# Thiazolidinones: novel insights from microwave synthesis, computational studies, and potentially bioactive hybrids

**DOI:** 10.3762/bjoc.21.203

**Published:** 2025-11-28

**Authors:** Luan A Martinho, Victor H J G Praciano, Guilherme D R Matos, Claudia C Gatto, Carlos Kleber Z Andrade

**Affiliations:** 1 Instituto de Química, Laboratório de Química Metodológica e Orgânica Sintética (LaQMOS), Universidade de Brasília, 70904-970, Brasília, DF, Brazil; 2 Instituto de Química, Laboratório de Modelagem de Sistemas Complexos (LMSC), Universidade de Brasília, Campus Universitário Asa Norte, 70904-970, Brasília, DF, Brazil; 3 Instituto de Química, Laboratório de Síntese Inorgânica e Cristalografia (LASIC), Universidade de Brasília, Campus Universitário Asa Norte, 70904-970, Brasília, DF, Brazil

**Keywords:** fluorescence, hybridization, imidazo[1,2-*a*]pyridines, Knoevenagel reaction, thiazolidinone

## Abstract

Various 5-arylidene derivatives were prepared via a Knoevenagel condensation-type reaction of aromatic/heteroaromatic aldehydes with rhodanine or thiazolidine-2,4-dione (TZD) catalyzed by EDA/AcOH under microwave heating. This convenient methodology is broad in scope (49 different products were obtained), delivers excellent yields (up to 99%), and requires low catalyst loading (10 mol %). This new approach was successfully applied to the synthesis of eight novel imidazo[1,2-*a*]pyridine–thiazolidinone hybrids in good to excellent yields (66–99%). A spectroscopic study of compounds **3n** and **4n** was carried out using torsion angle analysis and ^13^C NMR chemical shift calculations to evaluate the absence of expected signals in the NMR spectra of these compounds. Their photophysical properties were also assessed, confirming a preference for a fluorescence mechanism via an ICT (intramolecular charge transfer) process.

## Introduction

Heterocycles are compounds of significant interest to organic chemists as drug candidates due to their already widespread presence in many commercially available drugs [1]. These structures exhibit unique properties that influence pharmacokinetic and pharmacodynamic parameters, including lipophilicity, polarity, hydrogen-bonding capacity, and toxicological profiles [2–4]. Among them, five-membered multi-heterocyclic (FMMH) rings are privileged scaffolds for several synthetic or natural compounds [5].

**Figure 1:**
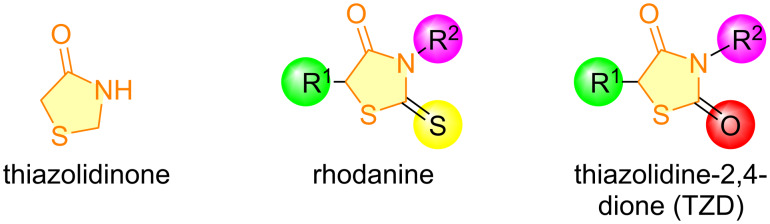
Structure of thiazolidinone derivatives.

**Figure 2:**
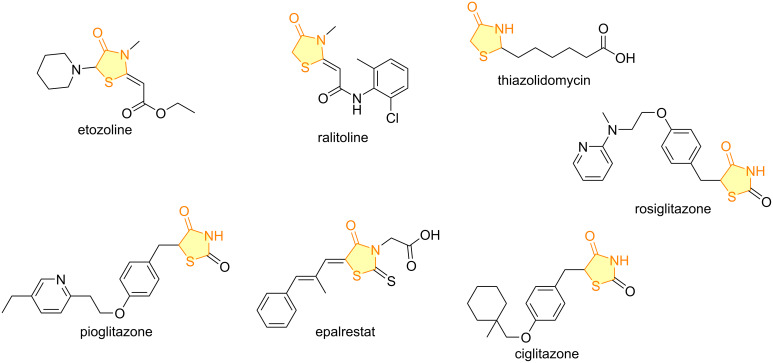
Selected examples of commercial drugs containing the thiazolidinone core.

Five-membered heterocycles include the thiazolidinone nucleus, characterized by two heteroatoms and a carbonyl group on the fourth carbon, as seen in compounds like rhodanine and thiazolidine-2,4-dione derivatives (Figure 1). These privileged moieties exhibit a broad spectrum of biological activities [6–8]. They are found in several medicinal compounds (Figure 2), including etozoline (antihypertensive), ralitoline (anticonvulsant), thiazolidomycin (antibacterial), and in the type II diabetes mellitus drugs pioglitazone, epalrestat, ciglitazone, and rosiglitazone [9–10].

Rhodanine (2-thioxothiazolidin-4-one) derivatives exhibit a wide range of pharmacological activities, including antiviral [11], antimalarial [12], antimicrobial [13], anti-inflammatory [14], anticancer [15], antidiabetic [16], antibacterial [17], and antifungal [18]. Some rhodanine-based derivatives act as inhibitors of hepatitis C virus (HCV) protease [19], UDP-*N*-acetylmuramate/ʟ-alanine ligase [20], histidine decarboxylase [21], aldose/aldehyde reductase [22], fungal protein mannosyl transferase 1 (PMT1) [23], metallo β-lactamase [24], cathepsin D [25], JNK-stimulating phosphatase-1 (JSP-1) [26], and anthrax lethal factor protease [27]. Thiazolidine-2,4-dione (TZD) derivatives also display diverse pharmacological activities, including antibacterial [28], antifungal [29], antidiabetic [30], antitubercular [31], antiparasitic [32], anti-inflammatory [33], antioxidant [34], cytotoxic [35], and antiproliferative [36].

The structures of these compounds allow the synthesis of a large collection of bioactive molecules due to their nucleophilic and electrophilic properties [37]. One of the forms of modifications is at the methylene group, particularly in the 5-arylidene derivatives (5-arylidene-2-thioxothiazolidin-4-one or 5-arylidenethiazolidine-2,4-dione), which have attracted the attention of medicinal chemists, and, consequently, several strategies have been created to synthesize these molecules [38]. The main methodology comes from the Knoevenagel condensation-type reaction between aromatic aldehydes with rhodanine or thiazolidine-2,4-dione [39]. Various protocols have been reported employing diverse catalyst systems and reaction conditions.

Common catalysts include inorganic bases such as sodium acetate (NaOAc) [40], urea/thiourea [41], NaOH [42], diammonium phosphate (DAP) [43], and tetrabutylammonium bromide (TBAB) [44]. Organic bases like morpholine [45], triethylamine [46], ethanolamine, and piperidine [47] and heterogeneous catalysts from Cu [48], Ti [49], and Zn [50] metal compounds have also been used. Common solvents are ethanol [51], toluene [52], acetic acid [53], or solvent-free processes [54]. Ionic liquids, such as [Bu_4_N][OH] [55], [bmim][OH] [56], and [Et_3_NH][HSO_4_] [57], and deep eutectic solvents (DES) [58] have been introduced to improve efficiency. Traditional methods for synthesizing these compounds, however, face several drawbacks, including long reaction times, harsh conditions, low to moderate yields, tedious work-up procedures, use of environmentally hazardous solvents, high catalyst loadings, toxic residues, and the need for specialized apparatus. To address these limitations, alternative methodologies have been explored. One notable approach is the use of microwave irradiation. Microwave-assisted synthesis has proven effective in reducing reaction times and improving yields [59–62].

Herein, we report the synthesis of 5-arylidene derivatives from rhodanine or thiazolidine-2,4-dione via the Knoevenagel condensation-type reaction using ethylenediamine (EDA) as catalyst in AcOH under microwave (μw) heating. This convenient methodology is broad in scope, provides the condensation products in high yields (up to 99%), with a reasonable catalyst loading (10 mol %) in only 30 minutes. This approach also enabled the efficient synthesis of eight novel imidazo[1,2-*a*]pyridine–thiazolidinone hybrids in high yields (up to 99%). A spectroscopic analysis of selected compounds was carried out, including torsion angle evaluation and ^13^C NMR chemical shift calculations, justifying the absence of expected signals in some cases. Photophysical studies indicated a fluorescence mechanism via intramolecular charge transfer (ICT).

## Results and Discussion

### Synthesis of 5-arylidene derivatives under microwave heating

To find the ideal reaction conditions for the synthesis of 5-arylidene derivatives, benzaldehyde (**1a**) and rhodanine (**2a**) were chosen as model substrates, and various parameters were systematically screened under microwave heating. Microwave-assisted reactions usually improve selectivities and yields, while significantly reduce reaction times. This approach has consistently yielded positive results in our research group (Table 1) [63].

**Table 1:** Optimization of reaction conditions for the synthesis of (*Z*)-5-benzylidene-2-thioxothiazolidin-4-one under microwave heating.^a^

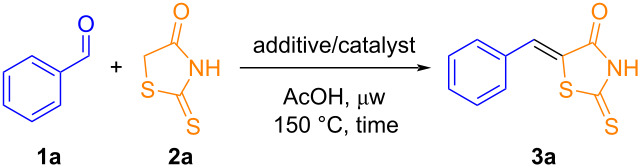			

Entry	Additive/catalyst	Time (min)	Yield (%)^b^

1	NaOAc (1.0 equiv)	30	32
2	NaOAc (1.0 equiv)	60	34
3	NaOAc/piperidine (1.0 equiv)	60	84
4	NaOAc/piperidine (1.0 equiv)	90	95
5	piperidine (1.0 equiv)	5	78
6	piperidine (1.0 equiv)	15	84
7	piperidine (1.0 equiv)	30	95
8	piperidine (1.0 equiv)	60	84
9	piperidine (1.0 equiv)	90	82
10	piperidine (1.0 equiv)	60	–^c^
11	pyridine (1.0 equiv)	30	6
12	Et_3_N (1.0 equiv)	30	24
13	EDA (1.0 equiv)	30	96
14	EDA (1.6 equiv)	30	96
15	EDA (0.80 equiv)	30	96
16	EDA (0.40 equiv)	30	96
17	EDA (0.20 equiv)	30	96
**18**	**EDA (0.10 equiv)**	**30**	**99**
19	EDA (0.10 equiv)	15	91
20	EDA 0.10 equiv)	10	89
21	EDA (0.10 equiv)	5	84
22	–	30	2

^a^Reaction conditions: benzaldehyde (0.50 mmol) and rhodanine (0.50 mmol) in 2.5 mL of AcOH. ^b^Isolated yields. ^c^Ethanol was used as a solvent (product was 2-amino-5-benzylidenethiazol-4-one).

Glacial acetic acid (AcOH) was initially selected as a hydrophilic (polar) protic solvent due to its high dipole moment, which makes it ideal for microwave reactions, as well as its ability to facilitate simple work-up procedures by simple addition of water [64]. Using sodium acetate (NaOAc) as an additive resulted in low reaction yields of the desired product **3a** (Table 1, entries 1 and 2). The addition of an aliphatic amine (piperidine) to the reaction medium significantly improved the yields (Table 1, entries 3 and 4). In the absence of NaOAc, only a slight decrease in yield was observed in different reaction times (Table 1, entries 5–9), highlighting the formation of product **3a** in an excellent yield of 95% in just 30 min (Table 1, entry 7).

**Scheme 1:**
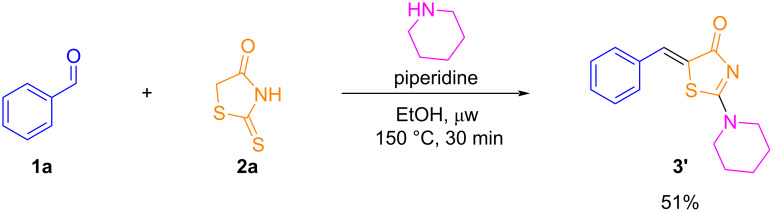
Multicomponent reaction of benzaldehyde, rhodanine, and piperidine in ethanol leading directly to a 2-amino-5-benzylidenethiazol-4-one.

Further adjustments in the reaction time were evaluated, but no improvements in yields were observed (Table 1, entry 9). Efforts were also made to replace AcOH by ethanol (Table 1, entry 10), however, the use of ethanol with piperidine was controversial. We found out that, during the reaction with benzaldehyde (**1a**) and rhodanine (**2a**), a multicomponent reaction was taking place by a substitution process, leading directly to 2-amino-5-benzylidenethiazol-4-one (**3’**) in moderate yield (Scheme 1). The implications and scope of this finding will be published in due course.

Switching to aromatic (pyridine) or tertiary amines (Et_3_N) resulted in low yields (Table 1, entries 11 and 12). In contrast, the use of ethylenediamine (EDA) stood out, delivering an excellent yield of 96% over different equimolar quantities (Table 1, entries 13–18). Interestingly, the yield was essentially quantitative for a lower catalyst loading of just 10 mol % in a reaction time of 30 minutes (Table 1, entry 18). Attempts to shorten the reaction time led to a slight decrease in yields (Table 1, entries 19–21). To confirm the critical role of ethylenediamine in the reaction mechanism, an experiment without its presence was conducted, resulting in only 2% of the desired product (Table 1, entry 22).

**Scheme 2:**
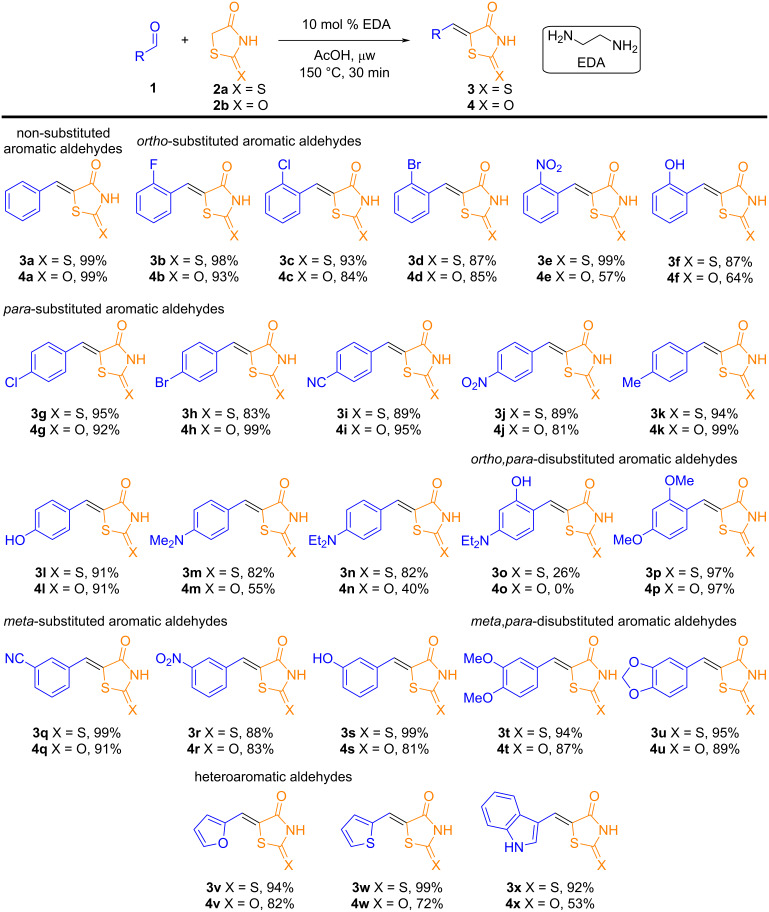
Substrate scope of the EDA-catalyzed Knoevenagel condensation reactions using a range of aromatic/heteroaromatic aldehydes. Reaction conditions: aromatic/heteroaromatic aldehyde (0.50 mmol), rhodanine (0.50 mmol) or thiazolidine-2,4-dione (1.00 mmol), and EDA (0.05 mmol, 10 mol %) in AcOH (2.50 mL), under microwave (µw) heating. The yields refer to isolated yields, and the structures were confirmed by FTIR, NMR, and HRMS analyses.

With the optimized reaction conditions established, this methodology was applied to a variety of aromatic aldehydes (Scheme 2). Aldehydes containing halides (–F, –Cl, –Br) or strong electron-withdrawing groups at the *ortho*-position provided excellent yields (87–99%) for the 5-benzylidene-2-thioxothiazolidin-4-one derivatives **3b–e**. The presence of an electron-donating group in *ortho*-position such as a hydroxy group generated compound **3f** in good yield (87%).

A broad scope was observed for *para*-substituted aldehydes, where both weak (**3g**, **3h**) and strong (**3i**, **3j**) electron-withdrawing groups gave good to excellent yields (83–95%). Similarly, aldehydes with electron-donating groups, such as –Me (**3k**), –OH (**3l**) or –NR_2_ (**3m**, **3n**), also resulted in good to excellent yields (82–94%) of products. However, disubstituted aldehydes containing a hydroxy group in the *ortho*-position resulted in significantly lower yields (26%) for the corresponding product (**3o**), a fact not observed for the presence of the –OMe group (**3p**). Aldehydes with substituents in the *meta*-position also delivered excellent results (**3q**–**s**) and aldehydes with OR groups in the *meta*,*para*-position (**3t**, **3u**) gave almost quantitative yields (up to 95%). Finally, the developed protocol was also shown to be effective for heterocyclic aldehydes such as furfural (**3v**), thiophene (**3w**) and indole (**3x**) carboxaldehydes.

**Scheme 3:**
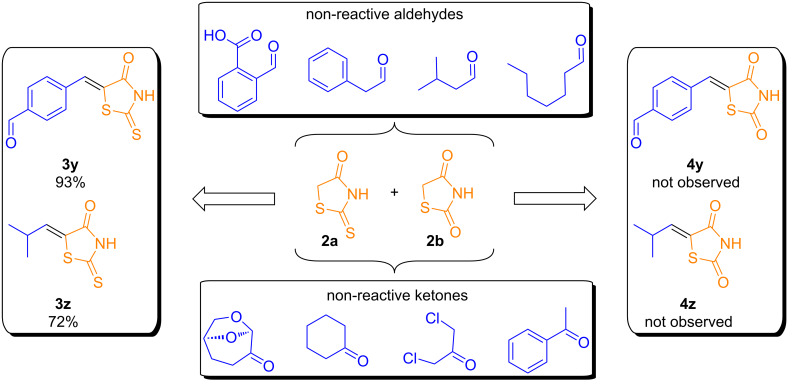
Limitations of the EDA-catalyzed Knoevenagel reactions for the synthesis of rhodanine or thiazolidine-2,4-dione derivatives.

Application of the optimized EDA-catalyzed Knoevenagel conditions to thiazolidine-2,4-dione (**2b**) afforded the expected product **4a** in moderate yield (Supporting Information File 1, Table S1). Adding excess thiazolidine improved the yield, although overall, 5-arylidenethiazolidine-2,4-dione derivatives were obtained in slightly lower yields than the corresponding rhodanine derivatives (Scheme 2). It is also important to highlight the limitations of our protocol (Scheme 3). Attempts to recover the catalyst were unsuccessful. Notably, with terephthalaldehyde, only the single condensation product (**3y**) was obtained in excellent yield for the condensation with rhodanine, no evidence of the double condensation product was observed. However, when TZDs were used, only the double condensation product was observed. Additionally, some substrates proved incompatible with this reaction protocol. 2-Carboxybenzaldehyde did not result in the recovery of any solid product by simple filtration, probably due to its high solubility in water. Use of aliphatic aldehydes presented a significant challenge, as only the product **3z** from isobutyraldehyde could be obtained. Other aliphatic aldehydes, such as phenylacetaldehyde, isovaleraldehyde, and heptanal, yielded only self-condensation products. A similar issue was observed with certain ketones, such as cyrene, cyclohexanone, 1,3-dichloroacetone, and acetophenone, since the condensation reaction with these substrates is not commonly reported in the literature [37,39]. The reaction of EDA in EtOH as solvent was investigated; however, a complex mixture of products was observed, with no evidence of formation of the Knoevenagel adduct. This outcome appears to result from both mono- and disubstitution processes involving sulfur-to-nitrogen displacement, likely promoted by the nucleophilic character of the amine. These limitations are likely due to the reaction conditions employed, including the high temperature and acidic environment, which may favor side reactions over the desired transformations.

The EDA-catalyzed Knoevenagel condensation reactions performed well even when a 10-fold increase in scale was applied (Supporting Information File 1, Scheme S1). The overall reaction profile was very much alike the smaller scales, affording the products in excellent yields (96–97%).

**Scheme 4:**
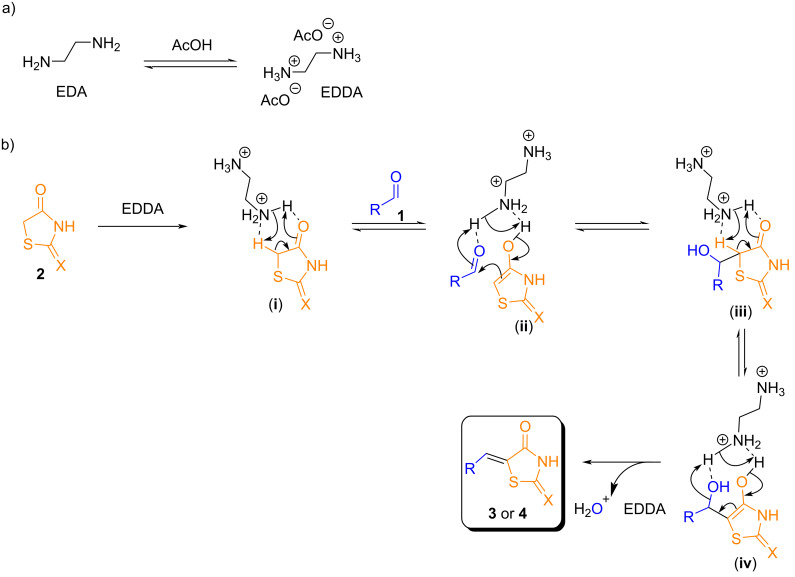
Plausible reaction mechanism for the EDA-catalyzed Knoevenagel condensation reactions.

A proposed mechanism involving the participation of ethylenediamine (EDA) is shown in Scheme 4. Initially, the basic EDA is protonated by acetic acid (AcOH), forming ethylenediamine diacetate (EDDA) in situ. The use of EDDA has been reported as a highly efficient catalyst for the synthesis of 5-arylidene-2,4-thiazolidinediones via the Knoevenagel condensation of aromatic aldehydes with 2,4-thiazolidinedione [65]. Nevertheless, the described solvent-free procedure (5 mol % of EDDA, at 80 °C for only 3 minutes) was not effective in our hands. Another similar protocol was reported by Gandini et al. [66] who used 50 mol % of EDDA at 80 °C for 30 minutes under microwave irradiation. Despite this later methodology looks very efficient, the method reported here enables the in situ generation of the catalyst, allowing for an expanded reaction scope with 2,4-TZD derivatives (23 examples) and also proving effective for condensation reactions involving rhodanine derivatives (26 examples). In addition, it requires a lower catalyst loading (10 mol %) and eliminates the need for further purification steps.

EDDA has also been employed in reactions for the synthesis of heterocyclic compounds such as tetrahydroquinolines [67], tetrahydrobenzo[*b*]pyrans [68], and pyrrolo[3,4-*c*]quinolinediones [69]. EDDA favors the enol formation of the thiazolidinone (**2**) through hydrogen donation from the protonated amino group, thus facilitating its removal and formation of enol **ii** (Scheme 4). This enol adds to the carbonyl group of the aromatic aldehyde **1**, activated by protonation of the oxygen atom through structure **ii**, forming the intermediate aldol **iii**. In the presence of EDDA, water elimination occurs in intermediate **iv**, yielding the Knoevenagel adducts **3** or **4**.

### Synthesis of novel imidazo[1,2-*a*]pyridine–thiazolidinone hybrids

To further demonstrate the effectiveness of our protocol, we explored the synthesis of novel imidazo[1,2-*a*]pyridine–thiazolidinone hybrid compounds, based on recent works from our group involving the chemistry of imidazo[1,2-*a*]pyridine derivatives synthesized via the Groebke–Blackburn–Bienaymé three-component reaction (GBB-3CR) [70–72]. These derivatives are of significant pharmacological and commercial interest, and are present in various commercial drugs, such as alpidem, miroprofen, necopidem, saripidem, zolimidine, and zolpidem [73].

**Scheme 5:**
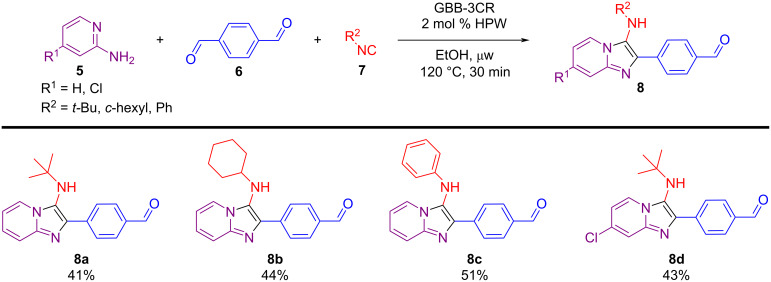
Substrate scope of the HPW-catalyzed GBB reactions.

**Scheme 6:**
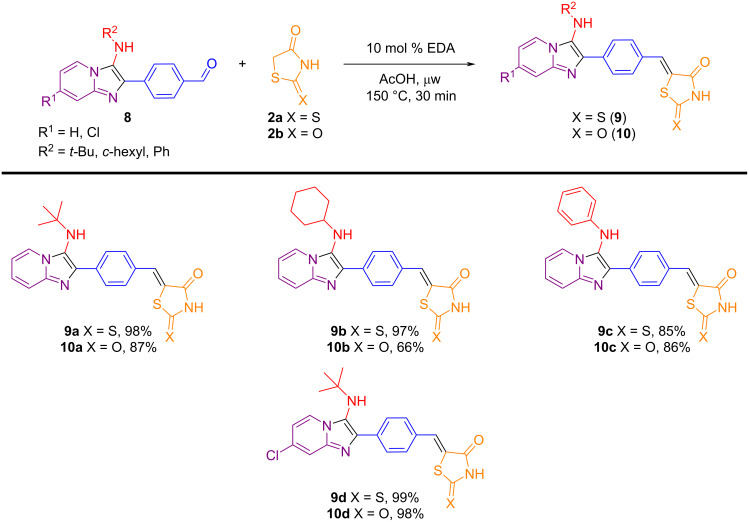
Synthesis of imidazo[1,2-*a*]pyridine-thiazolidinone hybrids by EDA-catalyzed Knoevenagel condensation reactions.

Using the EDA-catalyzed Knoevenagel condensation reaction methodology, hybrid compounds combining the imidazo[1,2-*a*]pyridine scaffold with the thiazolidine nucleus were synthesized. Initially, aldehyde derivatives of the GBB adducts **8** were prepared from 2-aminopyridines **5**, terephthalaldehyde (**6**), and isocyanides **7** using a green methodology that employed phosphotungstic acid (H_3_PW_12_O_40_, HPW) as a catalyst in ethanol under microwave (μw) heating (Scheme 5) [63]. These intermediates were then subjected to the previously optimized Knoevenagel condensation conditions with rhodanine or thiazolidine-2,4-dione (Scheme 6). Notably, the hybrid compounds **9a**–**d**, **10a**–**d** were obtained in good to excellent yields (66–99%) and may present several advantages, notably their potential to enhance bioactivity, expand structural diversity, and contribute to fluorescence properties. Comprehensive studies to fully evaluate these characteristics are in progress.

### Spectroscopic study of compounds **3n** and **4n**

**Figure 3:**
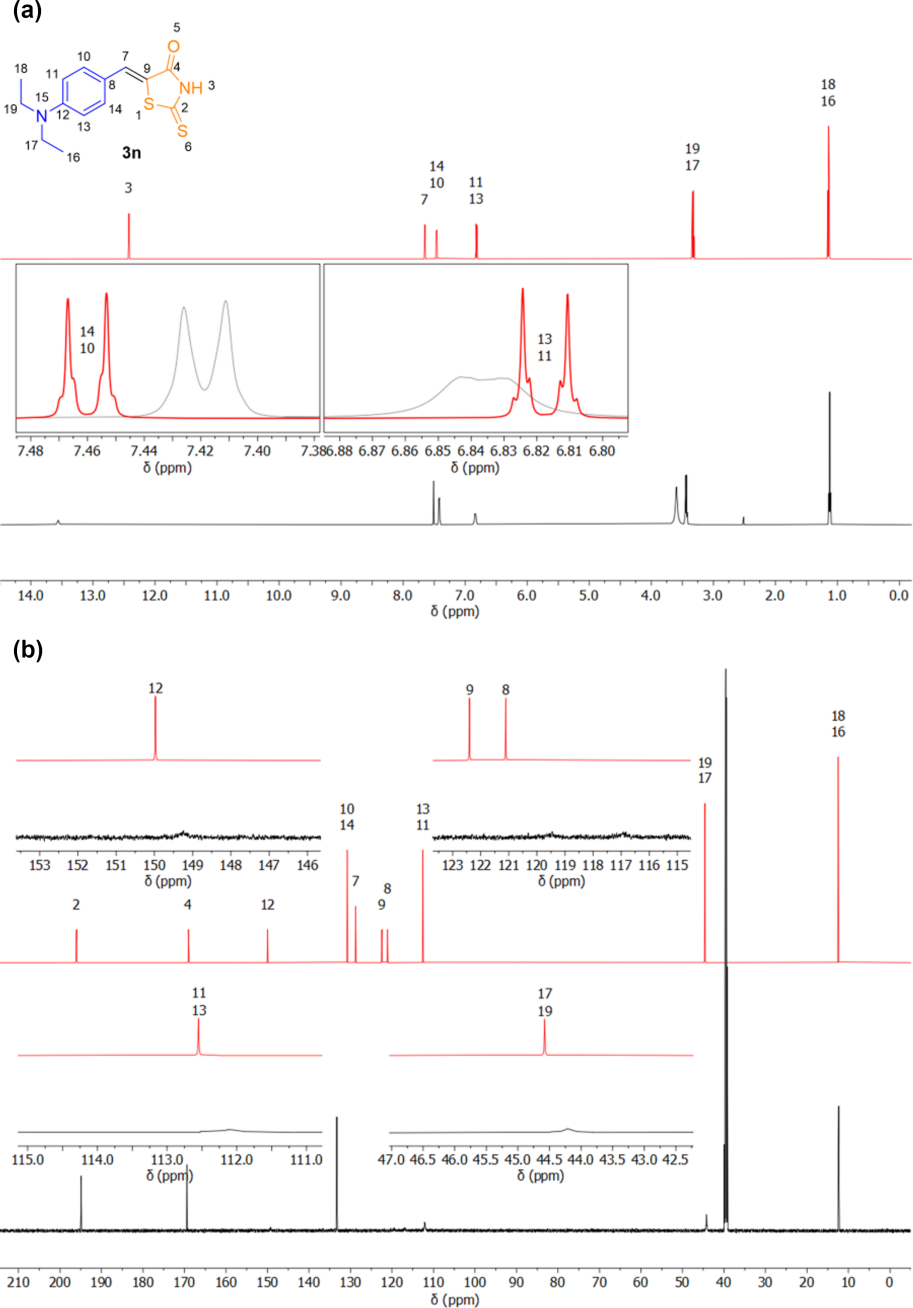
Overlay of predicted (red) and experimental (black) NMR spectra for compound **3n**: a) ^1^H NMR spectra (600 MHz, DMSO-*d*_6_) with expansions, and b) ^13^C NMR spectra (151 MHz, DMSO-*d*_6_) with expansions.

During the characterization of the compounds obtained in our study to determine the reaction scope, an anomaly was observed in the NMR spectra of products **3n** and **4n** derived from 4-diethylaminobenzaldehyde (see Supporting Information File 1). The spectra were acquired at 600 MHz for ^1^H NMR (320 scans) and 151 MHz for ^13^C NMR (20,480 scans), using an overnight experiment in DMSO-*d*_6_ at a concentration of 0.1 M. Specifically, the spectra showed either an absence of expected signals or a lack of signal multiplicity, which was apparent in both the ^1^H NMR and, more prominently, in the ^13^C NMR spectra. For instance, in compound **3n**, the aromatic (C-8 and C-12) and the olefinic (C-9) quaternary carbons, the methylene carbons attached to *N* (C-17, and C-19), and the *ortho*-*N* carbons displayed either absent or significantly weak signals, deviating from the expected patterns (Figure 3). This inconsistency made it challenging to rely on NMR as the primary method for confirming the structure of the expected products. To overcome this limitation, other analytical techniques, such as mass spectrometry and infrared (IR) spectroscopy, were employed. These methods successfully corroborated the formation of the desired products.

**Figure 4:**
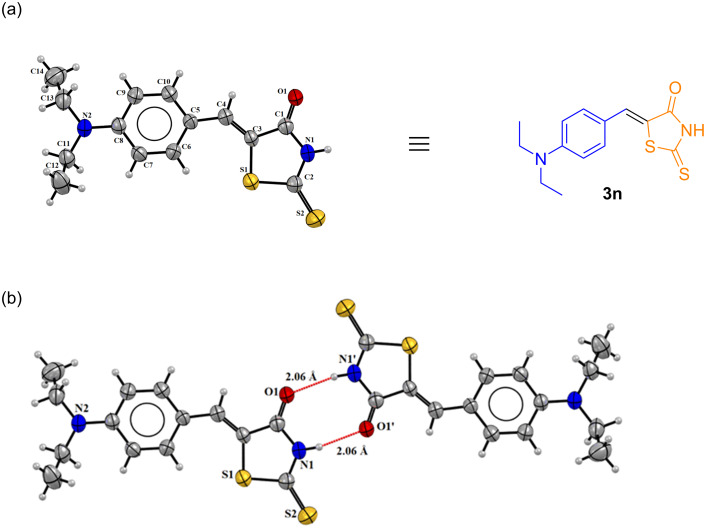
a) Molecular structure of **3n** with crystallographic labeling (50% probability displacement). b) Perspective views of intermolecular hydrogen bonds (dotted lines) of **3n**. (‘) symmetry operation: *−x* + 2, *−y* + 1, *−z +* 1.

More decisively, a single crystal X-ray analysis was performed. Compounds **3n** and **4n** were provided with suitable crystals for X-ray structural analysis resulting in new crystal structures, Figure 4 and Figure S250 in Supporting Information File 1, respectively. The single crystal XRD analysis confirmed the presence of the expected products and the stereochemistry of the newly created olefinic compound as being *Z*, as expected. Compound **3n** crystallizes in the monoclinic crystal system with four molecules in the asymmetric unit whereas **4n** crystallizes in the triclinic crystal system with two molecules in the asymmetric unit*.* The bond distances in **3n** C1=O1 is 1.229(4) Å and C2=S2 is 1.629(4) Å. For compound **4n**, the observed C=O bond length is shorter than that of compound **3n** (1.202(2) and 1.221(2) Å), respectively (Supporting Information File 1, Table S3). Intermolecular interactions are observed in both compounds forming dimer-like structures. The compounds show hydrogen bonds between N1–H1···O1’ with a distance of 2.06 Å in **3n** (Figure 4b) and 1.98 Å in **4n** (Figure S250b in Supporting Information File 1).

**Scheme 7:**
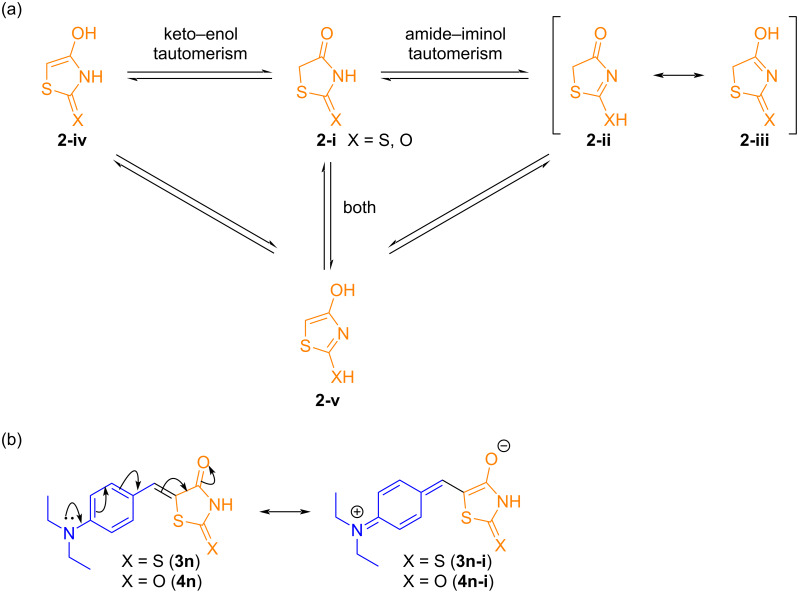
a) Tautomeric forms of thiazolidinones and b) resonance structures for compounds **3n** and **4n**.

The anomaly observed in the NMR spectra may be attributed to tautomerization within the structure of the products, likely induced by the presence of the 4-diethylamino (4-NEt_2_) group, a strong electron-donating substituent. The canonical structure of thiazolidines, due to the presence of one or two carbonyl groups, thiol groups, and α-hydrogens, allows the formation of multiple tautomers (Scheme 7a) [37,39]. Relative stability of these tautomers is ranked as follows: **2-i** > **2-ii** > **2-iii** > **2-iv** > **2-v** [74–76]. Therefore, compounds **3n** and **4n** may exist in equilibrium, with the enolic form being slightly more stable, which could explain the unusual behavior in their NMR spectra (Scheme 7b).

**Figure 5:**
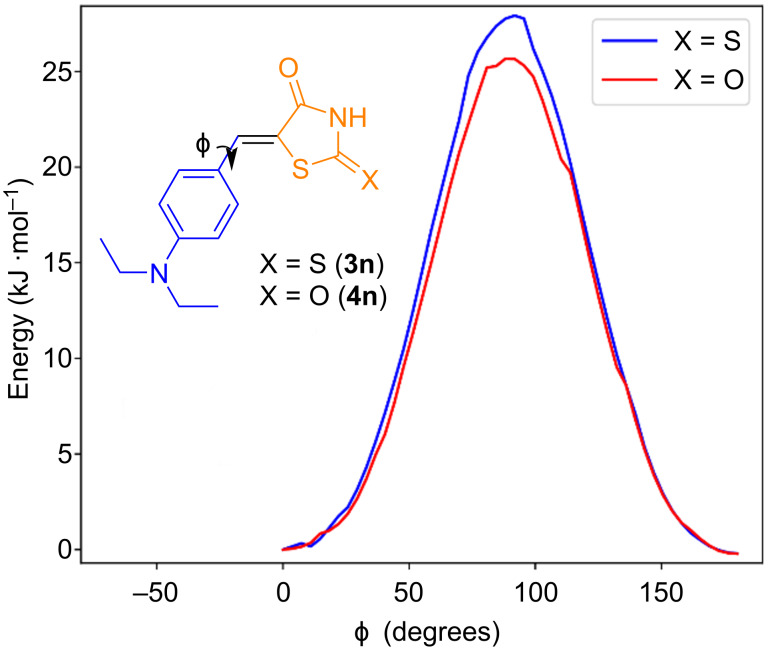
Molecular energy as a function of the torsion angle obtained from a relaxed dihedral scan at the M06-2X/def2-TZVPP level of theory.

A remarkable feature of the ^13^C NMR spectra of compounds **3n** and **4n** in DMSO is the attenuation/absence of peaks corresponding to the carbon atoms at the *ortho* and *meta* positions of the six-membered ring. It is known that quaternary carbon atoms have very long relaxation times, but this behavior is not expected from hydrogen-bonded carbon atoms in aromatic rings. Since peak intensities depend on spin-lattice relaxation times, the absence of peaks implies some degree of molecular rigidity, which could be related to two factors: the viscosity of DMSO and the conjugation between the six-membered and the five-membered rings. A relaxed dihedral scan – which allows a geometry optimization at every step – indicated that torsion barriers exceed 25 kJ/mol (Figure 5), indicating that the torsion angle is about twice as rigid as the torsion of a regular sigma bond in ethane (≈12 kJ/mol) [77–78].

**Figure 6:**
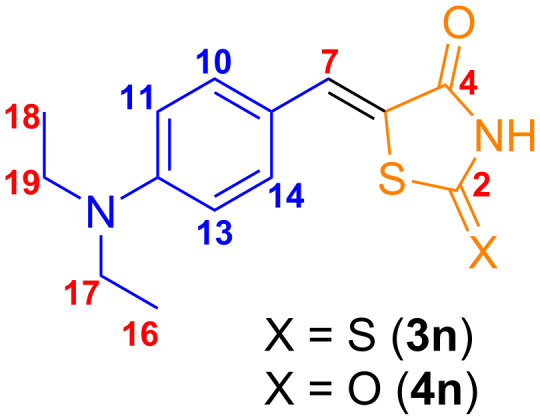
Identification of the carbon atoms used in the theoretical study of chemical shifts. In **red**, easily identifiable carbon atoms in the experimental spectrum. In **blue**, suppressed or absent carbon atoms bound to hydrogen atoms.

At first glance, it may seem that the carbon atoms in the *ortho* positions are equivalent to each other, as are the carbon atoms in the *meta* positions, which would result in two peaks in a ^13^C NMR spectrum. However, the torsional barrier and solvent viscosity stabilize the molecule in its most stable conformation, causing the nuclei on each side of the molecule to experience different local magnetic environments. In this case, four smaller peaks – each corresponding to a distinct carbon atom – should be observed. The absence of these peaks could be explained by longer relaxation times associated with the rigidity of the molecule. ^13^C NMR chemical shifts calculated at the B97-D/def2-TZVPP level in CPCM DMSO agreed with the assigned peaks with a mean absolute error of 3.30 ppm and 2.97 ppm for compounds **3n** and **4n**, respectively (Figure 6 and Table 2). Additional information can be found in Supporting Information File 1 (Tables S4 and S5). B97-D/def2-TZVPP calculations confirmed the hypothesis that the carbons labeled as 10, 11, 13, and 14 could be split in four peaks (Table 3) with chemical shift differences of ≈1 ppm and ≈8 ppm for the *ortho* and *meta* carbon atoms, respectively, with respect to the *N*-bonded carbon atom.

**Table 2:** Experimental and Boltzmann-weighted B97-D/def2-TZVPP chemical shifts (ppm) of molecules **3n** and **4n**.

	Molecule **3n** (X = S)		Molecule **4n** (X = O)	
Atom	Experimental	B97-D/def2-TZVPP	Experimental	B97-D/def2-TZVPP

18	12.8	14.4	12.5	14.3
19	44.7	51.5	45.0	51.3
7	133.0	133.6	132.9	133.6
2	195.4	192.3	168.6	170.2
4	169.9	167.1	167.9	165.2

**Table 3:** Boltzmann-weighted B97-D/def2-TZVPP chemical shifts (ppm) of suppressed or absent carbon atoms bound to hydrogen atoms (Figure 6) in molecules **3n** and **4n**.

Atom	Molecule **3n** (X = S)	Molecule **4n** (X = O)

13	114.8	113.9
14	130.8	129.7
11	113.9	113.0
10	138.8	138.1

Given that **3n** and **4n** contain donor–acceptor (DA)-type groups separated by a π-bond, the study of their photophysical properties has become particularly interesting. This structural arrangement often facilitates an internal charge transfer (ICT) process, which is common for such compounds [79]. The colorimetric properties of rhodanine-derived compound **3n** were used in the determination of silver [80] and gold [81] metals, and as a fluorescent sensor for detection of human serum albumin (HSA) [82]. Despite this, the TZD-derived compound **4n** had its photophysical properties neglected. Therefore, a study of the properties of both compounds was carried out. The spectroscopic data are summarized in Table 4.

**Table 4:** Photophysical properties of compounds **3n** and **4n**.^a^

Compd.	Φ*_f_*^b^	Solvent	λ_abs_ (nm)^c^	λ_em_ (nm)^d^	log ε (ε, M^−1^ cm^−1^)^e^	Stokes shift (cm^−1^)^f^

**3n**	0.00	CHCl_3_	480	535	4.70 (49897)	2,142
		EtOAc	462	539	4.75 (56801)	3,092
		CH_2_Cl_2_	479	540	4.77 (59261)	2,358
		DMSO	481	561	4.48 (29911)	2,965
		MeCN	470	555	4.70 (50337)	3,259
		MeOH	467	558	4.54 (34291)	3,492
		H_2_O	476	573	4.31 (20581)	3,556
**4n**	0.00	CHCl_3_	426	479	4.55 (35770)	2,597
		EtOAc	409	481	4.61 (40973)	3,660
		CH_2_Cl_2_	424	483	4.59 (38766)	2,881
		DMSO	423	497	4.49 (30877)	3,520
		MeCN	415	493	4.58 (38336)	3,812
		MeOH	415	486	4.52 (32807)	3,520
		H_2_O	415	524	4.19 (15488)	5,012

^a^Analyses were carried out at room temperature (10^−5^ M). ^b^Fluorescence quantum yields were measured with reference to quinine sulfate in 0.5 M H_2_SO_4_ (Φ_st_ = 0.546) and were carried out at room temperature. Excited at 366 nm. Compounds **3n** and **4n** were measured in acetonitrile (10^−5^ M) at room temperature. ^c^λ_abs_ = absorption maxima (nm). ^d^λ_em_ = emission maxima (nm). ^e^ε = molar absorptivity (M^−1^ cm^−1^). ^f^Stokes shifts difference between λ_em_ = emission maxima (cm^−1^) and λ_abs_ = absorption maxima (cm^−1^).

**Figure 7:**
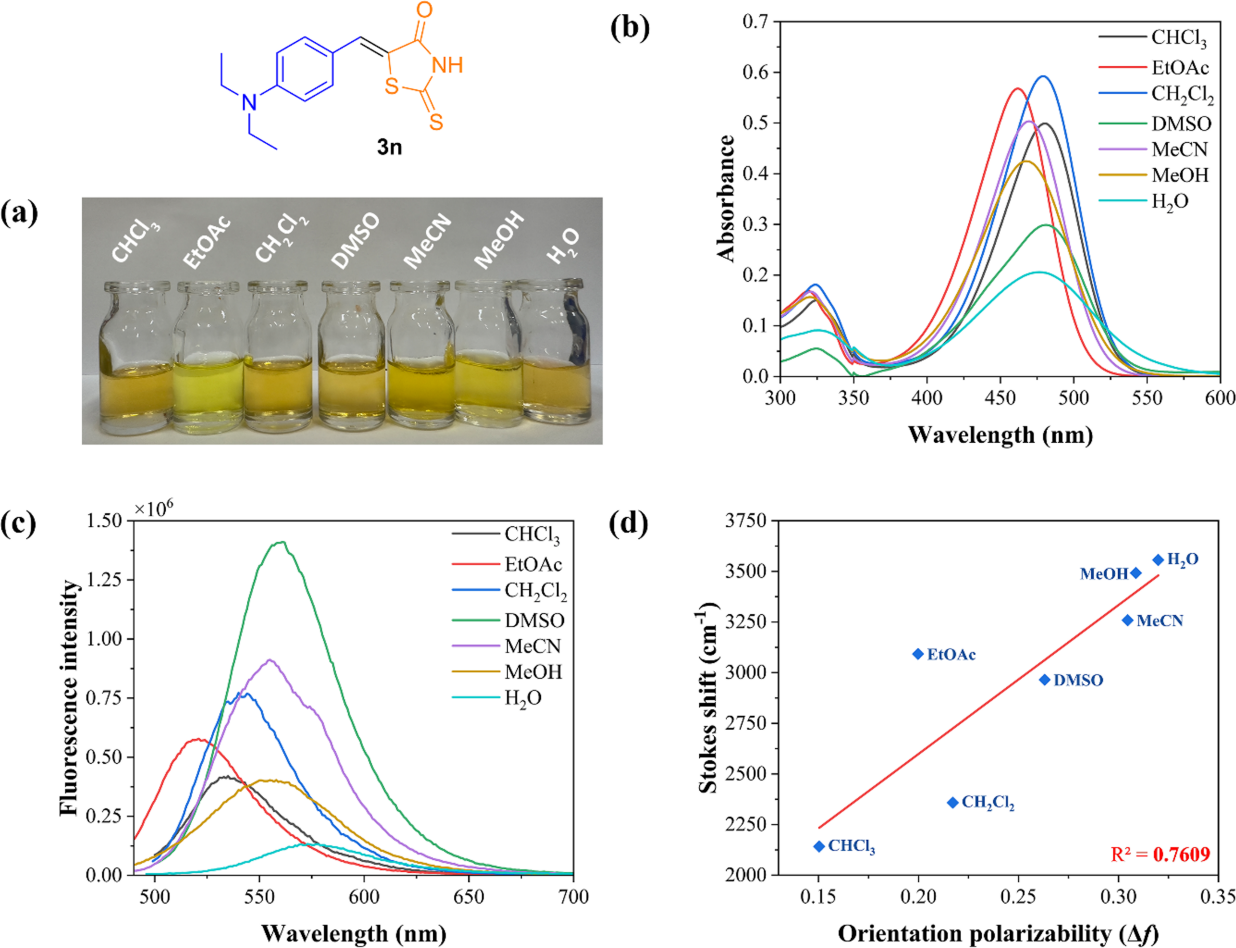
a) Visual impressions of the solvatochromic study in various solvents (10^−5^ M) after excitation with a natural light. b) UV–vis absorption spectra of **3n** in different solvents (10^−5^ M) at room temperature. c) Normalized emission spectra of **3n** in different solvents (10^−5^ M) at room temperature. d) Lippert–Mataga plot showing Stokes shift as a function of solvent orientation polarizability (Δ*_f_*) for compound **3n**.

We first investigated their fluorescence properties through the quantum yield Φ*_f_* by a comparative method with quinine sulfate, a standard of known quantum yield in 0.5 M H_2_SO_4_ (Φ_st_ = 0.546), with an excitation wavelength of 366 nm [83–85]. Nevertheless, no measurable quantum yield was observed for the fluorescence process. A solvatochromic study was conducted by measuring the UV–vis absorption and fluorescence spectra of compounds **3n** (Figure 7) and **4n** (Supporting Information File 1, Figure S251) in various solvents, including polar aprotic solvents (EtOAc, CH_2_Cl_2_, CHCl_3_, DMSO, and MeCN) and polar protic solvents (MeOH and H_2_O). However, the use of a non-polar solvent like hexane was not feasible due to the compounds' low solubility in this solvent.

The 5-arylidene derived from rhodanine (**3n**) exhibited an intense absorption band in the range of 462–481 nm, related to the π→π*-type transitions. Additionally, weaker, broad absorption bands were observed below 350 nm. In contrast, the thiazolidine-2,4-dione derivative **4n** displayed a blue shift, with maximum absorption band values ranging between 409–426 nm. In general, the solvatochromic effect of these compounds showed a blue shift in EtOAc, and a red shift in MeCN.

The fluorescence emission spectra of these compounds revealed a strong blue to green fluorescence emission, with the maximum emission peaks varying from 535 to 573 nm for compound **3n** and from 479 to 524 nm for compound **4n**. Therefore, the fluorescence emission spectra demonstrated a clear dependence on solvent polarity and the maximum emission wavelength λ_em_. Thus, a red shift in the emission bands was observed in solvents with higher polarity, indicating greater charge separation in the excited state, which is more stabilized in polar solvents, typical of compounds that undergo an ICT upon excitation [86]. The use of EtOAc showed a hypochromic effect with a decrease in fluorescence emission. Similar results for chlorinated solvents (CH_2_Cl_2_, CHCl_3_) were observed, however, with an increase in fluorescence emission. Interestingly, in DMSO, a bathochromic effect was observed alongside a significant increase in fluorescence intensity. In aqueous medium, a pronounced red shift was verified, likely due to stabilization of the excited state by water molecules through intramolecular hydrogen bonding.

**Scheme 8:**
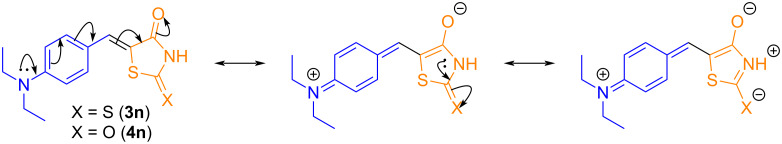
Proposed ICT-type mechanism for the fluorescence process, adapted from ref. [89].

By comparing the data for λ_abs_ and λ_em_, it is evident that the compounds exhibit significant Stokes shifts with values above 2,100 cm^−1^, reflecting their stability in the excited state [87–89]. The highest values of Stokes shifts were observed in polar protic solvents (MeOH, H_2_O) whereas lower values were recorded for chlorinated solvents (CH_2_Cl_2_, CHCl_3_). This trend matches with the Lippert–Mataga plots generated for these compounds to verify the relationship between the large values of Stokes shifts and the Reichardt polarity parameters. The graphs shown in Figure 7d and Figure S251d in Supporting Information File 1 provide data on the stabilization mechanism in the excited state in relation to the ICT process and the polarity of the solvent [90–91]. The graphs were generally linear, demonstrating that ICT-type mechanisms were favored for these compounds (Scheme 8) [92].

**Figure 8:**
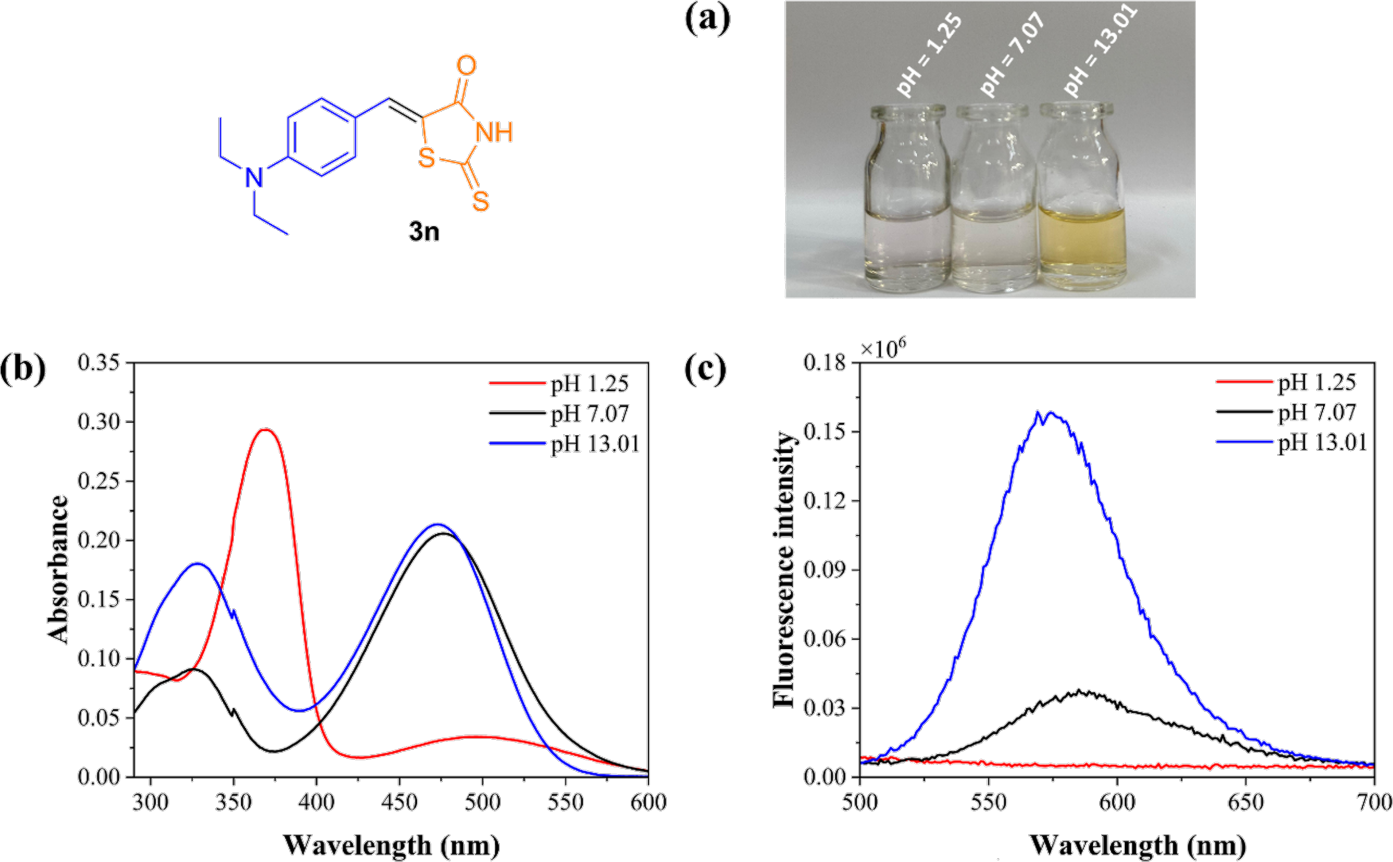
Photophysical study in aqueous solution under different pH values for compound **3n** (10^−5^ M) at room temperature. a) Visual impressions of the aqueous solution at various pH after excitation with a natural light. b) UV–vis absorption spectra. c) Normalized emission spectra.

**Scheme 9:**
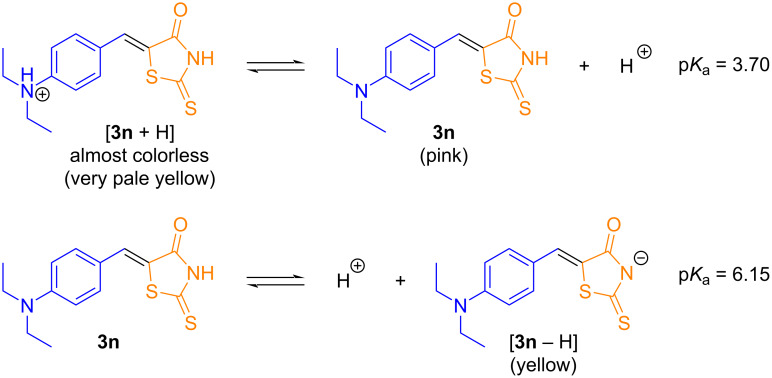
Two equilibria of compound **3n** in aqueous solutions, adapted from ref. [92–93].

Finally, an investigation of the influence of pH on the absorption and fluorescence spectra in aqueous solution was carried out under various pH values for compounds **3n** (Figure 8) and **4n** (Figure S252 in Supporting Information File 1), as changes in pH are known to significantly affect absorption and emission processes [93–94]. Compound **3n** is reported to have an ampholyte character existing in two equilibria in aqueous solutions (Scheme 9) [95–96]. Visually, the solutions lacked coloration under acidic pH compared to the neutral conditions. This observation was further analyzed quantitatively using UV–vis absorption spectroscopy. At acidic pH, only broad absorption bands at 370 nm and 325 nm were detected for compounds **3n** and **4n**, respectively. These results can be attributed to a phototautomerism effect in both the ground and excited states [97].

Additionally, fluorescence spectra under these conditions showed a suppressive effect. Under basic conditions, similar results were obtained for both products in comparison to the neutral medium for the maximum absorption bands at 473 and 403 nm, attributed to π→π*-type transitions. However, a notable increase in the broad absorption band around 350 nm was observed. This increase can be explained by deprotonation of the structures, which facilitates solvation effects by water molecules. The cause for the tautomerism effect at acidic pH may be due to the protonation of the basic site of the amino group present in the derivatives, thus hindering an ICT process (see Supporting Information File 1, Scheme S2).

**Figure 9:**
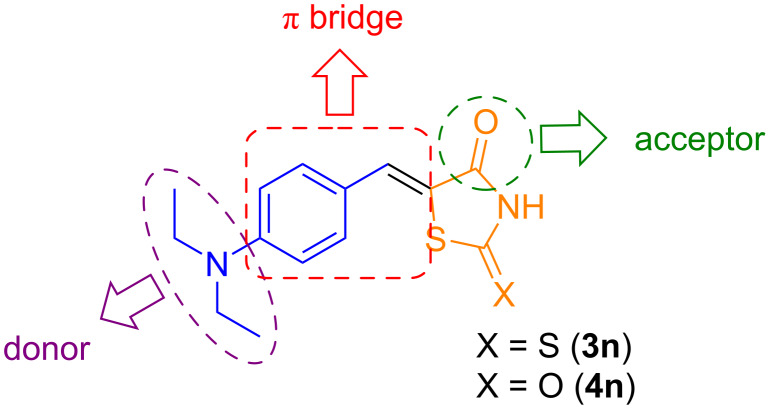
Molecular fragments associated with intramolecular charge transfer states.

The molecular structure of compounds **3n** and **4n** can be separated in three blocks: a donor region, a π bridge and an acceptor region (Figure 9) common to molecules with intramolecular charge transfer (ICT) states.

**Figure 10:**
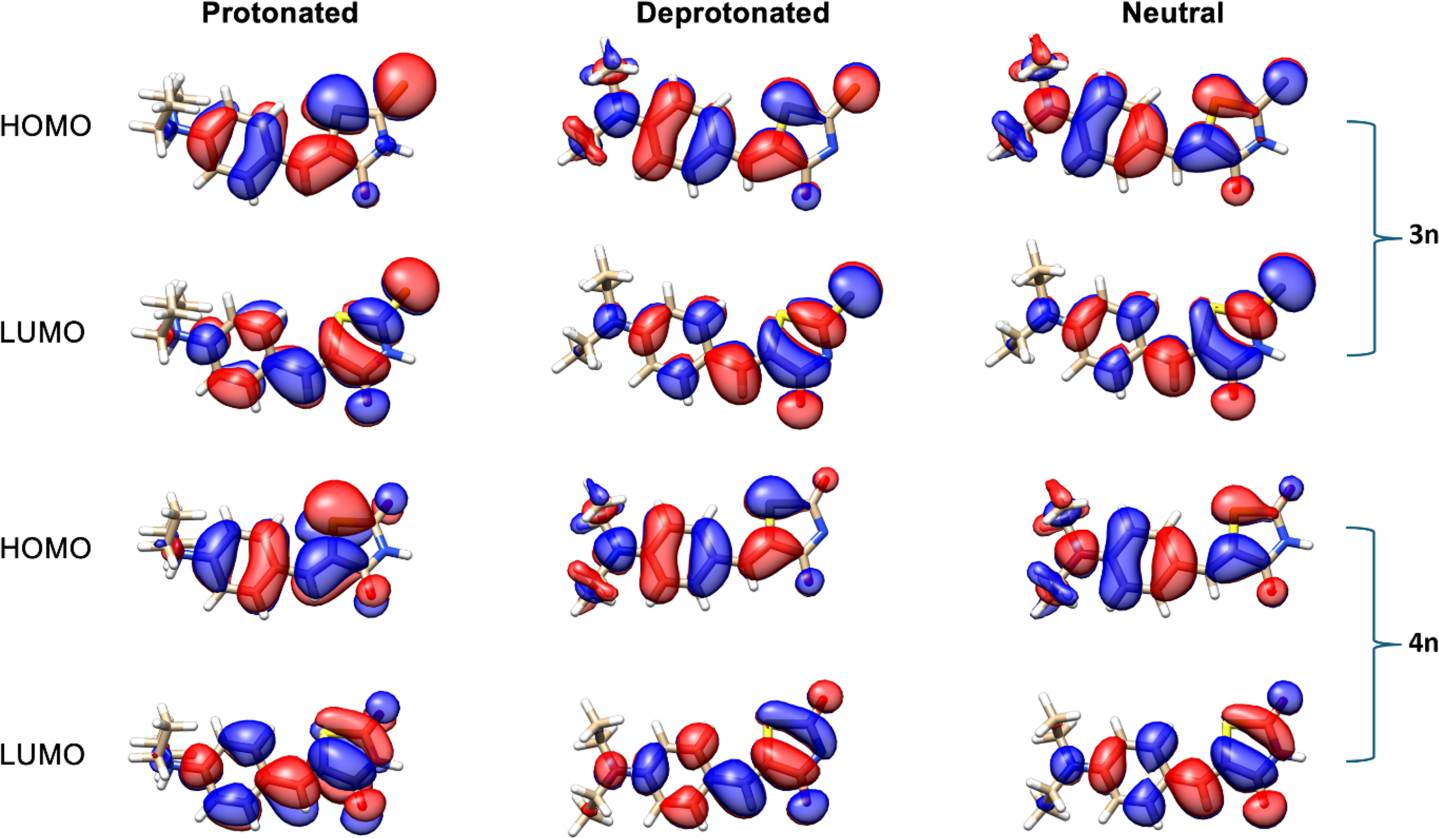
Frontier molecular orbitals of compounds **3n** and **4n** in three different states: protonated, deprotonated, and neutral.

M06-2X/def2-TZVPP single point calculations in CPCM water of compounds **3n** and **4n** in protonated, deprotonated, and neutral forms showed that there is a degree of charge transfer between a HOMO–LUMO excitation in their neutral and deprotonated forms [98]. Protonation of the nitrogen atom in the donor region shifts the electronic density towards the acceptor regions (Figure 10), which inhibits the intramolecular charge transfer upon excitation. It is important to note that protonation and deprotonation can significantly alter the HOMO–LUMO gap of a molecule by disrupting its electronic structure. In general, protonation of these compounds resulted in a lowering of the HOMO energy level, leading to an increase in the HOMO–LUMO gap and, consequently, greater molecular stability. In contrast, deprotonation led to an increase in the LUMO energy level, with little influence on the HOMO energy. Notably, the neutral forms of compounds **3n** and **4n** exhibited smaller HOMO–LUMO gaps compared to their protonated and deprotonated counterparts, suggesting higher reactivity (Table 5).

**Table 5:** Frontier molecular orbital (FMO) energies of compounds **3n** and **4n** in electron volts (eV).

	FMO energies (eV) of **3n**			FMO energies (eV) of **4n**		
	protonated	deprotonated	neutral	protonated	deprotonated	neutral

HOMO	−7.8279	−6.2428	−6.5021	−8.0059	−6.2145	−6.4983
LUMO	−2.1293	−1.1911	−1.7834	−1.7697	−0.6884	−1.3268
Δ*E*	5.6986	5.0517	4.7187	6.2362	5.5261	5.1715

## Conclusion

In summary, we have developed a straightforward approach to a Knoevenagel condensation-type reaction using ethylenediamine (EDA) as a catalyst in AcOH under microwave (μw) heating for the synthesis of 5-arylidene derivatives of rhodanine or thiazolidine-2,4-dione. Despite some minor limitations, such as the use of some ketones and aliphatic aldehydes, this convenient methodology is broad in scope (49 products were obtained), delivers excellent yields (up to 99%), and requires a low catalyst loading (10 mol %). The reactions are fast and can be applied to a wide range of aromatic and heteroaromatic aldehydes, with product isolation achieved by the simple addition of an aqueous HCl solution. This methodology was successfully applied to the synthesis of novel imidazo[1,2-*a*]pyridine–thiazolidinone hybrids in good to excellent yields (66–99%). A spectroscopic study of compounds **3n** and **4n** was conducted using torsion angle analysis and ^13^C NMR chemical shift calculations at the B97-D/def2-TZVPP level within the CPCM model for DMSO to investigate the absence of expected signals in the ^13^C NMR spectra, which can be attributed to longer relaxation times associated with the rigidity of the molecules. Furthermore, their photophysical properties were evaluated, confirming a preference for a fluorescence mechanism driven by an ICT (intramolecular charge transfer) process. The biological potential of these compounds is currently under investigation.

## Supporting Information

CCDC-2419877 and CCDC-2419878 contain the supplementary crystallographic data for this paper. These data can be obtained free of charge from the Cambridge Crystallographic Data Centre via http://www.ccdc.cam.ac.uk/structures.

File 1 Typical experimental procedures, FTIR, NMR and mass spectra of all compounds.

## Data Availability

All data that supports the findings of this study is available in the published article and/or the supporting information of this article.
