# Supplementary material for: Thiazolidinones: novel insights from microwave synthesis, computational studies, and potentially bioactive hybrids
Source: Beilstein J Org Chem. 2025 Nov 28;21:2618–36. doi: 10.3762/bjoc.21.203 (PMC12667731; doi:10.3762/bjoc.21.203)
Supplement: File 1 — Typical experimental procedures, FTIR, NMR and mass spectra of all compounds. [file Beilstein_J_Org_Chem-21-2618-s001.pdf]

BEILSTEIN JOURNAL OF ORGANIC CHEMISTRY

Supporting Information

for

Thiazolidinones: novel insights from microwave synthesis, computational studies, and potentially bioactive hybrids

Luan A. Martinho, Victor H. J. G. Praciano, Guilherme D. R. Matos, Claudia C. Gatto and Carlos Kleber Z. Andrade

Beilstein J. Org. Chem. **2025,** *21,* 2618–2636. doi:10.3762/bjoc.21.203

Typical experimental procedures, FTIR, NMR and mass spectra of all compounds

License and Terms: This is a supporting information file under the terms of the Creative Commons Attribution License (https://creativecommons.org/licenses/by/4.0). Please note that the reuse, redistribution and reproduction in particular requires that the author(s) and source are credited and that individual graphics may be subject to special legal provisions.
The license is subject to the *Beilstein Journal of Organic Chemistry* terms and conditions: (https://www.beilstein-journals.org/bjoc/terms)

Table of contents

1. Experimental section S2

1.1. General S2

1.2. Photophysical study S2

1.2.1. Quantum yield calculation S2

1.2.2. Absorbance and fluorescence analysis S3

1.2.3. Crystal structure determination S3

1.2.4. Computational methods S3

1.3. Experimental procedures S3

1.3.1. General synthetic procedure for the EDA-catalyzed Knoevenagel reactions under microwave heating S3

1.3.2. General synthetic procedure for the HPW-catalyzed gBB reactions under microwave heating S4

1.4. Synthesis and characterization S4

1.4.1. Synthesis and characterization of Knoevenagel adducts S4

1.4.2. Synthesis and characterization of imidazo[1,2-*a*]pyridines S20

1.4.3. Synthesis and characterization of imidazo[1,2-*a*]pyridine-thiazolidinone hybrids via Knoevenagel reaction S22

2. References S26

3. FTIR, ^{1}H and ^{13}C NMR, HRMS and HRMS/MS spectra for all compounds S28

4. Crystal structure determination of compounds **3n** and **4n** S153

5. Photophysical data S155

6. Benchmarking study S157

1. Experimental section

1.1. General

Unless otherwise stated, all reagents and solvents were purchased from Sigma-Aldrich Merck and used without further purification. Aldehydes, hexane, ethyl acetate and dichloromethane were distilled prior to their use.

All microwave-mediated reactions were performed on a Biotage® Initiator+ (Uppsala, Sweden) microwave reactor using a sealed vessel with simultaneous cooling, and media stirring, temperature detection via an internal fiber optic probe. The reagents consumption and products formation were confirmed by thin-layer chromatography (TLC) with UV fluorescent silica gel Merck 60 F254 plates and visualized by treatment with a 10% solution of phosphomolybdic acid in ethanol (PMA), followed by heating. Column chromatography was performed on silica gel (Supelco, pore size 60 Å, 230–400 mesh particle size, 40–63 µm particle size) and mixtures of hexane/ethyl acetate were used as eluents as described for each molecule.

FTIR spectra were obtained on a Jasco FT/IR - 4100 with TA DLaTGS as detector in the infrared region (4000–650 cm^{-1}) in attenuated total reflection (ATR) mode using baseline correction using asymmetrically reweighted penalized least squares smoothing [1]. The NMR spectra were recorded at 25 °C on a Bruker Ascend 600 spectrometer at 600 MHz for ^{1}H NMR and 151 MHz for ^{13}C NMR with TMS as an internal standard for residual ^{1}H and ^{13}C signals of $CDCl_3$ (δ 7.26 and δ 77.2 ppm) and DMSO-d_6 (δ 2.50 and δ 39.5 ppm) are referenced as solvent. HRMS experiments were performed on a Triple Tof 5600 Sciex by flow injection analysis using an Eksigent UltraLC 100 Sciex chromatograph set to a flow rate of 0.3 mL/min. A DuoSpray Ion Source (ESI) was used, and the MS spectra were acquired in positive or negative mode, employing external calibration, in the range of 50–1000 Da and 0.1% (v/v) of formic acid in acetonitrile or methanol as solvent**.** The melting points were measured with capillary in the LOGEN Scientific equipment (LS III Plus) and were not corrected. The stock solutions of the **3n** and **4n** products were prepared in DMSO at 10^{-2} M.

1.2. Photophysical study

1.2.1. Quantum yield calculation

For the determination of the quantum yield by the method of comparison with the quinine sulfate solution, this standard was used as it has a known fluorescence quantum yield (Φ_r = 0.546) [2-4]. The solution was prepared from quinine monohydrate and solubilized in H_2SO_4 solution (0.5 M), to obtain a concentration of 10^{-5} M. The stock solutions of the products were diluted in MeCN to obtain solutions with a concentration of 10^{-5} M. The analyses were performed in a Fluorolog-Horiba spectrofluorimeter at room temperature, using 10 mm standard cells, in emission mode, with an excitation source at 366 nm (reference value for quinine sulfate), reading with a wavelength scanning range of 386–700 nm at each 1.0 nm and 2.0 nm slit. The data were collected in the form of graphs corrected for lamp noise and the reading was lateral. With the data obtained, the curves were integrated and used in Equation 1. The refractive index of the quinine sulfate solution and the acetonitrile solvent are equal. The data were analyzed by the OriginPro graphics program (version 2025).

$$\Phi_f = \Phi_{st}\,(\frac{Grad_{exp}}{Grad_{st}}) \times (\frac{\eta_{exp}}{\eta_{st}})^2 \quad \text{Eq. 1}$$

in which Φ_{st} is the quantum yield described for the standard, the term grad refers to the gradient of the fluorescence integrated area and η is the refractive index of the solvent.

1.2.2. Absorbance and fluorescence analysis

UV–vis spectra were obtained on a UV–vis–NIR–Cary 5000 spectrophotometer at room temperature and 10 mm standard cells, using single-beam mode with prior blank reading. The chosen scanning range was 800 to 200 nm, with a data interval of 1.0 nm. Fluorescence emission measurements were performed at room temperature on a Fluorolog-Horiba spectrofluorimeter, in emission mode, with an excitation wavelength starting from the maximum absorption wavelength of each product, obtained from the absorption spectra, standard output: lateral, reading every 1 nm and slit of 2.0. Data were collected in the form of graphs corrected for lamp noise. Data were analyzed by the OriginPro graphics program (version 2025).

1.2.3. Crystal structure determination

Crystallographic analyses of the compounds were carried out with a Bruker CCD SMART APEX II, at 296 K, with graphite monochromated Mo Kα radiation (λ = 0.71073 Å). The crystal structures were solved by dual space using the SHELXT program and refined by full-matrix least-squares on F^2 using the SHELXL software [5]. Data from the unit cell was obtained by collecting three matrices, each with twelve images, and the refinement was carried out with anisotropic parameters, using the OLEX2 program [6]. Hydrogen atoms were located from the Fourier map and treated with the riding model. MERCURY [7] was used for the ORTEP plot and hydrogen-bonding pictures. Selected crystallographic data are resumed in Table S2 and selected bonds and angles are depicted in Table S3. Full crystallographic data for the compounds have been deposited with the CCDC numbers 2419877 and 2419878.

1.2.4. Computational methods

Molecules **3n** and **4n** were built using UCSF Chimera [8] and all quantum chemical calculations were run using ORCA 6.0.1 [9-12]. Molecular energy as a function of the torsion angle between the six-membered ring and the double bond was calculated at 50 equally spaced dihedral angles between 0 and 180 degrees at the M06-2X/def2-TZVPP level of theory [13,14]. A benchmarking study of ^{13}C NMR chemical shifts using the assigned chemical shifts of compounds **3n** and **4n** showed the DFT level of theory with the lowest mean absolute error B97-D/def2-TZVPP [15]. A conformer search was done using ORCA 6.0.1 gOAT program [16,17] at the gFN2-xTB level of theory [18]. A few of the lowest energy conformations were chosen for each molecule and their Boltzmann weights were obtained to allow the calculation of conformational energy-weighted ^{13}C NMR chemical shifts [19-21] in CPCM [22] DMSO. Lastly, acidic, basic and neutral forms of compounds **3n** and **4n** were studied at the M06-2X/def2-TZVPP in CPCM water to determine frontier molecular orbital shapes and energies.

1.3. Experimental procedures

1.3.1. General synthetic procedure for the EDA-catalyzed Knoevenagel reactions under microwave heating

A Biotage microwave reaction vial of 2.0–5.0 mL containing a mixture of aldehyde (0.50 mmol), rhodanine (0.50 mmol) or thiazolidine-2,4-dione (1.00 mmol), and ethylenediamine EDA (0.05 mmol, 10 mol %) in AcOH (2.5 mL) was introduced into in the cavity of a microwave reactor Biotage® Initiator+ and heated at 150 °C for 30 min under magnetic stirring. The reaction mixture was then cooled to room temperature, and reagents

consumption was confirmed by TLC analysis (mixture of ethyl acetate/hexane). Then, 5 mL of 2.5 M HCl were added to the reaction mixture and a solid precipitated. The resulting solid was collected by vacuum filtration and washed with 2.5 M HCl, cold water and ethanol. The expected product was isolated without requiring any further purification.

1.3.2. general synthetic procedure for the HPW-catalyzed gBB reactions under microwave heating

A Biotage microwave reaction vial of 0.5–2.0 mL containing a mixture of 2-aminopyridine (2.50 mmol), aldehyde (2.50 mmol), isocyanide (2.50 mmol), and phosphotungstic acid hydrate HPW (0.05 mmol, 2 mol %) in EtOH (2.5 mL) was introduced into in the cavity of a microwave reactor Biotage® Initiator+ and heated at 120 °C for 30 min under magnetic stirring. The reaction mixture was then cooled to room temperature, and reagents consumption was confirmed by TLC analysis (mixture of ethyl acetate/hexane). The reaction mixture was removed from the MW vial, concentrated under vacuum and the crude product was purified by silica gel column chromatography [23].

1.4. Synthesis and characterization

1.4.1. Synthesis and characterization of Knoevenagel adducts

(*Z*)-5-Benzylidene-2-thioxothiazolidin-4-one (**3a**)

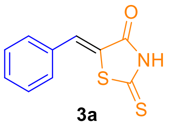

3a was obtained from benzaldehyde (0.50 mmol; 0.051 mL), rhodanine (0.50 mmol; 0.067 g), EDA (0.05 mmol; 0.003 mL) in AcOH (2.5 mL), in 99% yield (0.110 g) as a yellow solid (m.p.: 199-200 ºC; lit. m.p.: 200-202 ºC, *ref.* [24]).

FTIR (ATR): ν 3150, 3058, 2848, 1699, 1673, 1588, 1492, 1436, 1341, 1289, 1236, 1196, 1074, 1010, 921, 808, 761, 710, 676 cm^{-1}.

^{1}H NMR (600 MHz, DMSO-d_6): δ 13.85 (s, 1H), 7.65 (s, 1H), 7.63 – 7.58 (m, 2H), 7.58 – 7.52 (m, 2H), 7.52 – 7.48 (m, 1H) ppm.

^{13}C NMR (151 MHz, DMSO-d_6): δ 195.7, 169.4, 133.0, 131.7, 130.8, 130.5, 129.5, 125.5 ppm.

HRMS (ESI-QTOF) *m/z* calculated for $C_{10}H_6NOS_2^-$: 219.9896 $[M-H]^-$; found 219.9895.

(*Z*)-5-(2-Fluorobenzylidene)-2-thioxothiazolidin-4-one (**3b**)

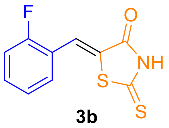

3b was obtained from 2-fluorobenzaldehyde (0.50 mmol; 0.053 mL), rhodanine (0.50 mmol; 0.067 g), EDA (0.05 mmol; 0.003 mL) in AcOH (2.5 mL), in 98% yield (0.117 g) as a yellow solid (m.p.: 187-188 ºC; lit. m.p.: 201-203 ºC, *ref.* [25]).

FTIR (ATR): ν 3147, 3052, 2839, 1692, 1608, 1591, 1480, 1455, 1427, 1309, 1296, 1208, 1157, 1068, 933, 796, 749, 709, 676 cm^{-1}.

^{1}H NMR (600 MHz, DMSO-d_6): δ 13.95 (s, 1H), 7.62 (s, 1H), 7.61 – 7.56 (m, 1H), 7.53 (td, *J* = 7.8, 1.7 Hz, 1H), 7.43 – 7.36 (m, 2H) ppm.

^{13}C NMR (151 MHz, DMSO-d_6): δ 195.5, 169.2, 160.7 (d, *J* = 252.7 Hz), 133.1 (d, *J* = 8.8 Hz), 129.4, 128.1, 125.6 (d, *J* = 3.5 Hz), 122.4 (d, *J* = 6.1 Hz), 120.9 (d, *J* = 12.0 Hz), 116.3 (d, *J* = 21.4 Hz) ppm.

HRMS (ESI-QTOF) *m/z* calculated for $C_{10}H_5FNOS_2^-$: 237.9802 $[M-H]^-$; found 237.9799.

(*Z*)-5-(2-Chlorobenzylidene)-2-thioxothiazolidin-4-one (**3c**)

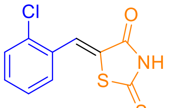

3c

3c was obtained from 2-chlorobenzaldehyde (0.50 mmol; 0.056 mL), rhodanine (0.50 mmol; 0.067 g), EDA (0.05 mmol; 0.003 mL) in AcOH (2.5 mL), in 93% yield (0.119 g) as a yellow solid (m.p.: 174-175 ºC; lit. m.p.: 177-178 ºC, *ref.* [26]).

FTIR (ATR): ν 3067, 2854, 1739, 1694, 1596, 1583, 1456, 1418, 1304, 1279, 1234, 1184, 1040, 997, 852, 749, 715, 670 cm^{-1}.

^1H NMR (600 MHz, DMSO-d_6): δ 13.97 (s, 1H), 7.76 (s, 1H), 7.69 – 7.61 (m, 1H), 7.55 – 7.48 (m, 3H) ppm.

^{13}C NMR (151 MHz, DMSO-d_6): δ 195.5, 169.1, 134.8, 132.1, 130.9, 130.5, 129.3, 129.1, 128.3, 126.1 ppm.

HRMS (ESI-QTOF) *m/z* calculated for $C_{10}H_5ClNOS_2^-$: 253.9507 [M−H]$^-$; found 253.9507.

(*Z*)-5-(2-Bromobenzylidene)-2-thioxothiazolidin-4-one (**3d**)

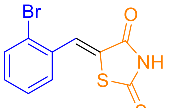

3d

3d was obtained from 2-bromobenzaldehyde (0.50 mmol; 0.058 mL), rhodanine (0.50 mmol; 0.067 g), EDA (0.05 mmol; 0.003 mL) in AcOH (2.5 mL), in 87% yield (0.131 g) as a yellow solid (m.p.: 178-180 ºC; lit. m.p.: 185-186 ºC, *ref.* [27]).

FTIR (ATR): ν 3078, 2845, 1727, 1604, 1581, 1447, 1427, 1275, 1228, 1189, 1001, 750, 714, 679 cm^{-1}.

^1H NMR (600 MHz, DMSO-d_6): δ 13.97 (s, 1H), 7.82 (dd, *J* = 8.1, 1.2 Hz, 1H), 7.73 (s, 1H), 7.60 – 7.51 (m, 2H), 7.43 (td, *J* = 7.6, 1.7 Hz, 1H) ppm.

^{13}C NMR (151 MHz, DMSO-d_6): δ 195.6, 169.1, 133.7, 132.6, 132.2, 129.4, 129.1, 128.9, 128.8, 125.7 ppm.

HRMS (ESI-QTOF) *m/z* calculated for $C_{10}H_5BrNOS_2^-$: 299.8980 [M−H]$^-$; found 299.8968.

(*Z*)-5-(2-Nitrobenzylidene)-2-thioxothiazolidin-4-one (**3e**)

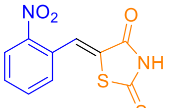

3e

3e was obtained from 2-nitrobenzaldehyde (0.50 mmol; 0.076 g), rhodanine (0.50 mmol; 0.067 g), EDA (0.05 mmol; 0.003 mL) in AcOH (2.5 mL), in 99% yield (0.133 g) as a yellow solid (m.p.: 198-200 ºC; lit. m.p.: 204-205 ºC, *ref.* [28]).

FTIR (ATR): ν 3068, 2934, 2850, 1750, 1692, 1602, 1567, 1531, 1419, 1338, 1299, 1227, 1182, 997, 863, 784, 749, 682 cm^{-1}.

^1H NMR (600 MHz, DMSO-d_6): δ 13.96 (s, 1H), 8.21 (dd, *J* = 8.3, 1.2 Hz, 1H), 7.92 – 7.86 (m, 2H), 7.77 – 7.69 (m, 2H) ppm.

^{13}C NMR (151 MHz, DMSO-d_6): δ 195.8, 168.6, 148.0, 134.6, 131.3, 130.3, 129.4, 128.8, 127.9, 125.5 ppm.

HRMS (ESI-QTOF) *m/z* calculated for $C_{10}H_5N_2O_3S_2^-$: 264.9747 [M−H]$^-$; found 264.9737.

(*Z*)-5-(2-Hydroxybenzylidene)-2-thioxothiazolidin-4-one (**3f**)

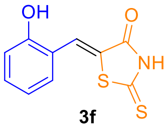

3f

3f was obtained from salicylaldehyde (0.50 mmol; 0.052 mL), rhodanine (0.50 mmol; 0.067 g), EDA (0.05 mmol; 0.003 mL) in AcOH (2.5 mL), in 87% yield (0.103 g) as an orange solid (m.p.: 215-217 °C; lit. m.p.: 177-178 °C, *ref.* [29]).

FTIR (ATR): ν 3501, 3433, 3092, 3038, 2844, 1685, 1568, 1458, 1431, 1338, 1250, 1212, 1190, 1151, 734, 683 cm^{-1}.

^{1}H NMR (600 MHz, DMSO-d_6): δ 13.74 (s, 1H), 10.69 (s, 1H), 7.86 (s, 1H), 7.38 – 7.30 (m, 2H), 7.00 – 6.94 (m, 2H) ppm.

^{13}C NMR (151 MHz, DMSO-d_6): δ 196.1, 169.6, 157.6, 132.9, 129.3, 127.3, 123.9, 120.0, 119.9, 116.3 ppm.

HRMS (ESI-QTOF) *m/z* calculated for $C_{10}H_6NO_2S_2^-$: 235.9845 $[M-H]^-$; found 235.9842.

(*Z*)-5-(4-Chlorobenzylidene)-2-thioxothiazolidin-4-one (**3g**)

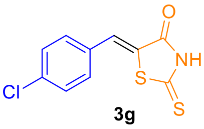

3g

3g was obtained from 4-chlorobenzaldehyde (0.50 mmol; 0.070 g), rhodanine (0.50 mmol; 0.067 g), EDA (0.05 mmol; 0.003 mL) in AcOH (2.5 mL), in 95% yield (0.122 g) as a yellow solid (m.p.: 222-223 °C; lit. m.p.: 225-226 °C, *ref.* [24]).

FTIR (ATR): ν 3666, 2980, 2900, 1702, 1596, 1557, 1485, 1402, 1226, 1180, 1085, 1007, 826, 801, 750, 721, 678 cm^{-1}.

^{1}H NMR (600 MHz, DMSO-d_6): δ 13.88 (s, 1H), 7.65 (s, 1H), 7.64 – 7.59 (m, 4H) ppm.

^{13}C NMR (151 MHz, DMSO-d_6): δ 195.4, 169.3, 135.4, 132.1, 131.9, 130.2, 129.5, 126.3 ppm.

HRMS (ESI-QTOF) *m/z* calculated for $C_{10}H_5ClNOS_2^-$: 253.9507 $[M-H]^-$; found 253.9501.

(*Z*)-5-(4-Bromobenzylidene)-2-thioxothiazolidin-4-one (**3h**)

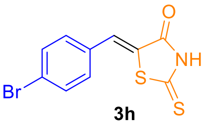

3h

3h was obtained from 4-bromobenzaldehyde (0.50 mmol; 0.092 g), rhodanine (0.50 mmol; 0.067 g), EDA (0.05 mmol; 0.003 mL) in AcOH (2.5 mL), in 83% yield (0.124 g) as a yellow solid (m.p.: 218-220 °C; lit. m.p.: 227-228 °C, *ref.* [27]).

FTIR (ATR): ν 3012, 3056, 2844, 1702, 1593, 1480, 1438, 1397, 1277, 1220, 1177, 1006, 917, 823, 799, 745, 717, 684 cm^{-1}.

^{1}H NMR (600 MHz, DMSO-d_6): δ 13.88 (s, 1H), 7.74 (d, J = 8.5 Hz, 2H), 7.62 (s, 1H), 7.54 (d, J = 8.5 Hz, 2H) ppm.

^{13}C NMR (151 MHz, DMSO-d_6): δ 195.4, 169.3, 132.5, 132.2, 132.2, 130.3, 126.4, 124.3 ppm.

HRMS (ESI-QTOF) *m/z* calculated for $C_{10}H_5BrNOS_2^-$: 299.8980 $[M-H]^-$; found 299.8961.

(*Z*)-4-((4-Oxo-2-thioxothiazolidin-5-ylidene)methyl)benzonitrile (**3i**)

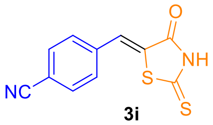

3i

3i was obtained from 4-formylbenzonitrile (0.50 mmol; 0.066 g), rhodanine (0.50 mmol; 0.067 g), EDA (0.05 mmol; 0.003 mL) in AcOH (2.5 mL), in 89% yield (0.109 g) as a yellow solid (m.p.: 285-286 °C; lit. m.p.: 286 °C (dec.), *ref.* [30]).

FTIR (ATR): ν 3657, 3112, 2980, 2900, 2233, 1709, 1596, 1441, 1410, 1227, 1189, 1065, 920, 828, 801, 672 cm^{-1}.

^1H NMR (600 MHz, DMSO-d_6): δ 13.98 (s, 1H), 7.98 (dd, *J* = 8.4, 1.8 Hz, 2H), 7.77 (dd, *J* = 8.4, 1.8 Hz, 2H), 7.69 (s, 1H) ppm.

^{13}C NMR (151 MHz, DMSO-d_6): δ 195.3, 169.3, 137.4, 133.1, 130.8, 129.2, 129.2, 118.4, 112.2 ppm.

HRMS (ESI-QTOF) *m/z* calculated for $C_{11}H_5N_2OS_2^-$: 244.9849 $[M+H]^+$; found 244.9850.

(*Z*)-5-(4-Nitrobenzylidene)-2-thioxothiazolidin-4-one (**3j**)

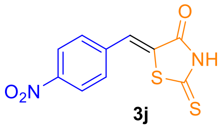

3j

3j was obtained from 4-nitrobenzaldehyde (0.50 mmol; 0.076 g), rhodanine (0.50 mmol; 0.067 g), EDA (0.05 mmol; 0.003 mL) in AcOH (2.5 mL), in 89% yield (0.118 g) as an orange solid (m.p.: 249-250 ºC; lit. m.p.: 249-250 ºC, *ref.* [31]).

FTIR (ATR): ν 3267, 3014, 1709, 1605, 1506, 1406, 1337, 1284, 1222, 1180, 999, 914, 843, 752, 703, 664 cm^{-1}.

^1H NMR (600 MHz, DMSO-d_6): δ 14.01 (s, 1H), 8.33 (d, *J* = 8.6 Hz, 2H), 7.84 (d, *J* = 8.6 Hz, 2H), 7.73 (s, 1H) ppm.

^{13}C NMR (151 MHz, DMSO-d_6): δ 195.3, 169.2, 147.5, 139.2, 131.3, 129.9, 128.6, 124.3 ppm.

HRMS (ESI-QTOF) *m/z* calculated for $C_{10}H_5N_2O_3S_2^-$: 264.9747 $[M-H]^-$; found 264.9743.

(*Z*)-5-(4-Methylbenzylidene)-2-thioxothiazolidin-4-one (**3k**)

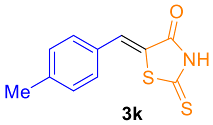

3k

3k was obtained from *p*-tolualdehyde (0.50 mmol; 0.059 mL), rhodanine (0.50 mmol; 0.067 g), EDA (0.05 mmol; 0.003 mL) in AcOH (2.5 mL), in 94% yield (0.111 g) as a yellow solid (m.p.: 215-217 ºC; lit. m.p.: 220-223 ºC, *ref.* [32]).

FTIR (ATR): ν 3138, 3046, 2841, 1686, 1587, 1508, 1430, 1310, 1290, 1224, 1198, 1180, 1068, 1016, 903, 806, 704, 675 cm^{-1}.

^1H NMR (600 MHz, DMSO-d_6): δ 13.80 (s, 1H), 7.60 (s, 1H), 7.49 (d, *J* = 8.0 Hz, 2H), 7.36 (d, *J* = 8.0 Hz, 2H), 2.36 (s, 3H) ppm.

^{13}C NMR (151 MHz, DMSO-d_6): δ 195.6, 169.4, 141.2, 131.8, 130.6, 130.2, 130.1, 124.3, 21.2 ppm.

HRMS (ESI-QTOF) *m/z* calculated for $C_{11}H_8NOS_2^-$: 234.0053 $[M-H]^-$; found 234.0049.

(*Z*)-5-(4-Hydroxybenzylidene)-2-thioxothiazolidin-4-one (**3l**)

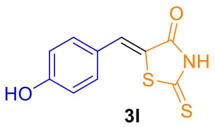

3l

3l was obtained from 4-hydroxybenzaldehyde (0.50 mmol; 0.061 g), rhodanine (0.50 mmol; 0.067 g), EDA (0.05 mmol; 0.003 mL) in AcOH (2.5 mL), in 91% yield (0.108 g) as an orange solid (m.p.: 268-270 °C; lit. m.p.: 184-185 °C, *ref.* [24]).

FTIR (ATR): ν 3389, 3046, 2941, 2844, 1693, 1568, 1513, 1427, 1225, 1168, 1011, 906, 839, 812, 743, 721, 687 cm^{-1}.

^{1}H NMR (600 MHz, DMSO-d_6): δ 13.69 (s, 1H), 10.43 (s, 1H), 7.56 (s, 1H), 7.46 (dd, *J* = 8.6, 2.2 Hz, 2H), 6.92 (dd, *J* = 8.6, 2.2 Hz, 2H) ppm.

^{13}C NMR (151 MHz, DMSO-d_6): δ 195.6, 169.5, 160.4, 133.1, 132.5, 124.0, 121.0, 116.6 ppm.

HRMS (ESI-QTOF) *m/z* calculated for $C_{10}H_6NO_2S_2^-$: 235.9845 $[M-H]^-$; found 235.9840.

(*Z*)-5-(4-(Dimethylamino)benzylidene)-2-thioxothiazolidin-4-one (**3m**)

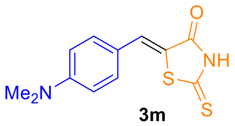

3m

3m was obtained from 4-(dimethylamino)benzaldehyde (0.50 mmol; 0.075 g), rhodanine (0.50 mmol; 0.067 g), EDA (0.05 mmol; 0.003 mL) in AcOH (2.5 mL), in 82% yield (0.109 g) as a purple solid (m.p.: 267-268 °C; lit. m.p.: 184-185 °C, *ref.* [33]).

FTIR (ATR): ν 3127, 3019, 2818, 1679, 1610, 1556, 1517, 1432, 1373, 1250, 1208, 1157, 1056, 801, 714, 675 cm^{-1}.

^{1}H NMR (600 MHz, DMSO-d_6): δ 13.55 (s, 1H), 7.51 (s, 1H), 7.44 – 7.38 (m, 2H), 6.84 – 6.78 (m, 2H), 3.03 (s, 6H) ppm.

^{13}C NMR (151 MHz, DMSO-d_6): δ 195.0, 169.4, 151.8, 133.3, 132.9, 119.8, 117.3, 112.2, 39.6 ppm.

HRMS (ESI-QTOF) *m/z* calculated for $C_{12}H_{11}N_2OS_2^-$: 263.0318 $[M-H]^-$; found 263.0328.

(*Z*)-5-(4-(Diethylamino)benzylidene)-2-thioxothiazolidin-4-one (**3n**)

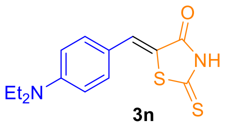

3n

3n was obtained from 4-(diethylamino)benzaldehyde (0.50 mmol; 0.089 g), rhodanine (0.50 mmol; 0.067 g), EDA (0.05 mmol; 0.003 mL) in AcOH (2.5 mL), in 82% yield (0.120 g) as a red solid (m.p.: 189-190 °C; lit. m.p.: 208 °C, *ref.* [34]).

FTIR (ATR): ν 3031, 2957, 2841, 1707, 1668, 1610, 1558, 1516, 1434, 1412, 1341, 1275, 1209, 1173, 1158, 1062, 1004, 808, 671 cm^{-1}.

^{1}H NMR (600 MHz, DMSO-d_6): δ 13.56 (s, 1H), 7.51 (s, 1H), 7.42 (d, *J* = 8.5 Hz, 2H), 6.84 (d, *J* = 8.5 Hz, 2H), 3.44 (q, *J* = 7.0 Hz, 4H), 1.12 (t, *J* = 7.0 Hz, 6H) ppm.

^{13}C NMR (151 MHz, DMSO-d_6): δ 194.9, 169.4, 133.3, 44.2, 12.4 ppm.

HRMS (ESI-QTOF) *m/z* calculated for $C_{14}H_{15}N_2OS_2^-$: 291.0631 $[M-H]^-$; found 291.0625.

(*Z*)-5-(4-(Diethylamino)-2-hydroxybenzylidene)-2-thioxothiazolidin-4-one (**3o**)

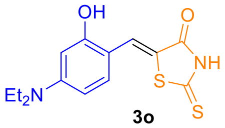

3o

3o was obtained from 4-(diethylamino)salicylaldehyde (0.50 mmol; 0.097 g), rhodanine (0.50 mmol; 0.067 g), EDA (0.05 mmol; 0.003 mL) in AcOH (2.5 mL), in 26% yield (0.040 g) as a yellow solid (m.p.: 234-235 ºC; lit. m.p.: 277 °C (dec.) ºC, *ref.* [30]).

FTIR (ATR): ν 3037, 2621, 2535, 2467, 1732, 1687, 1593, 1482, 1415, 1340, 1219, 1192, 1150, 1107, 1012, 919, 822, 791, 667, 650 cm^{-1}.

^{1}H NMR (600 MHz, DMSO-*d*$_6$): δ 13.45 (s, 1H), 7.80 (s, 1H), 7.15 (d, *J* = 8.9 Hz, 1H), 6.48 (s, 1H), 6.36 (s, 1H) 3.39 (q, *J* = 7.0 Hz, 4H), 1.12 (t, *J* = 7.0 Hz, 6H) ppm.

^{13}C NMR (151 MHz, DMSO-*d*$_6$): δ 195.1, 169.6, 160.0, 131.3, 128.4, 45.0, 12.4 ppm.

HRMS (ESI-QTOF) *m/z* calculated for $C_{14}H_{15}N_2O_2S_2^-$: 307.0580 [M−H]$^-$; found 307.0570.

(*Z*)-5-(2,4-Dimethoxybenzylidene)-2-thioxothiazolidin-4-one (**3p**)

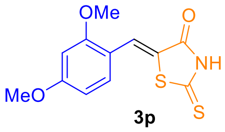

3p

3p was obtained from 2,4-dimethoxybenzaldehyde (0.50 mmol; 0.083 g), rhodanine (0.50 mmol; 0.067 g), EDA (0.05 mmol; 0.003 mL) in AcOH (2.5 mL), in 97% yield (0.137 g) as an orange solid (m.p.: 267-269 ºC; lit. m.p.: 271-272 ºC, *ref.* [35]).

FTIR (ATR): ν 3130, 3010, 2942, 2834, 1682, 1606, 1558, 1461, 1308, 1267, 1211, 1192, 1117, 1035, 1012, 826, 689 cm^{-1}.

^{1}H NMR (600 MHz, DMSO-*d*$_6$): δ 13.66 (s, 1H), 7.75 (s, 1H), 7.33 (dd, *J* = 8.7, 2.3 Hz, 1H), 6.72 (dt, *J* = 8.7, 2.3 Hz, 1H), 6.68 (d, *J* = 2.3 Hz, 1H), 3.91 (s, 3H), 3.86 (s, 3H) ppm.

^{13}C NMR (151 MHz, DMSO-*d*$_6$): δ 195.9, 169.6, 163.7, 160.1, 131.5, 127.1, 121.7, 114.3, 114.3, 107.0, 98.7, 56.0, 55.8 ppm.

HRMS (ESI-QTOF) *m/z* calculated for $C_{12}H_{10}NO_3S_2^-$: 280.0108 [M−H]$^-$; found 280.0100.

(*Z*)-3-((4-Oxo-2-thioxothiazolidin-5-ylidene)methyl)benzonitrile (**3q**)

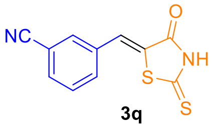

3q

3q was obtained from 3-formylbenzonitrile (0.50 mmol; 0.066 g), rhodanine (0.50 mmol; 0.067 g), EDA (0.05 mmol; 0.003 mL) in AcOH (2.5 mL), in 99% yield (0.123 g) as a yellow solid (m.p.: 280-281 ºC; lit. m.p.: not reported).

FTIR (ATR): ν 3245, 3072, 3015, 2839, 2229, 1723, 1703, 1604, 1475, 1415, 1283, 1243, 1188, 1061, 1003, 915, 797, 735, 674 cm^{-1}.

^{1}H NMR (600 MHz, DMSO-*d*$_6$): δ 13.94 (s, 1H), 8.07 (t, *J* = 1.5 Hz, 1H), 7.93 (dt, *J* = 8.0, 1.5 Hz, 1H), 7.85 (dt, *J* = 8.0, 1.5 Hz, 1H), 7.74 (t, *J* = 8.0 Hz, 1H), 7.66 (s, 1H) ppm.

^{13}C NMR (151 MHz, DMSO-*d*$_6$): δ 195.3, 169.2, 134.2, 134.2, 133.7, 133.6, 130.6, 129.1, 128.2, 118.1, 112.6 ppm.

HRMS (ESI-QTOF) *m/z* calculated for $C_{11}H_5N_2OS_2^-$: 244.9849 [M−H]$^-$; found 244.9846.

(*Z*)-5-(3-Nitrobenzylidene)-2-thioxothiazolidin-4-one (**3r**)

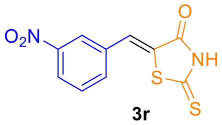
3r

3r was obtained from 3-nitrobenzaldehyde (0.50 mmol; 0.076 g), rhodanine (0.50 mmol; 0.067 g), EDA (0.05 mmol; 0.003 mL) in AcOH (2.5 mL), in 88% yield (0.117 g) as a yellow solid (m.p.: 255-256 ºC; lit. m.p.: 271-272 ºC, *ref.* [35]).

FTIR (ATR): ν 3232, 3011, 1693, 1596, 1525, 1401, 1344, 1223, 1169, 996, 916, 823, 739, 660, 796 cm^{-1}.

^{1}H NMR (600 MHz, DMSO-d_6): δ 13.98 (s, 1H), 8.42 (t, *J* = 2.2 Hz, 1H), 8.30 (dd, *J* = 8.0, 2.2 Hz, 1H), 7.99 (d, *J* = 8.0 Hz, 1H), 7.82 (t, *J* = 8.0 Hz, 1H), 7.78 (s, 1H) ppm.

^{13}C NMR (151 MHz, DMSO-d_6): δ 195.1, 169.2, 148.3, 135.7, 134.6, 131.0, 129.0, 128.5, 124.7, 124.6 ppm.

HRMS (ESI-QTOF) *m/z* calculated for $C_{10}H_5N_2O_3S_2^-$: 264.9747 $[M-H]^-$; found 264.9746.

(*Z*)-5-(3-Hydroxybenzylidene)-2-thioxothiazolidin-4-one (**3s**)

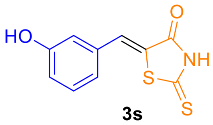
3s

3s was obtained from 3-hydroxybenzaldehyde (0.50 mmol; 0.061 g), rhodanine (0.50 mmol; 0.067 g), EDA (0.05 mmol; 0.003 mL) in AcOH (2.5 mL), in 99% yield (0.118 g) as a yellow solid (m.p.: 240-241 °C; lit. m.p.: 244 °C (dec.), *ref.* [36]).

FTIR (ATR): ν 3333, 2859, 1692, 1581, 1442, 1353, 1300, 1283, 1203, 1162, 1069, 1015, 900, 852, 776, 706, 672 cm^{-1}.

^{1}H NMR (600 MHz, DMSO-d_6): δ 13.83 (s, 1H), 9.89 (s, 1H), 7.55 (s, 1H), 7.35 (t, *J* = 7.9 Hz, 1H), 7.06 (dd, *J* = 7.9, 2.1 Hz, 1H), 6.98 (t, *J* = 2.1 Hz, 1H), 6.91 (dd, *J* = 7.9, 2.1 Hz, 1H) ppm.

^{13}C NMR (151 MHz, DMSO-d_6): δ 195.8, 169.4, 158.0, 134.2, 131.9, 130.6, 125.3, 121.9, 118.2, 116.2 ppm.

HRMS (ESI-QTOF) *m/z* calculated for $C_{10}H_6NO_2S_2^-$: 235.9845 $[M-H]^-$; found 235.9843.

(*Z*)-5-(3,4-Dimethoxybenzylidene)-2-thioxothiazolidin-4-one (**3t**)

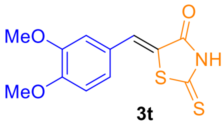
3t

3t was obtained from 3,4-dimethoxybenzaldehyde (0.50 mmol; 0.083 g), rhodanine (0.50 mmol; 0.067 g), EDA (0.05 mmol; 0.003 mL) in AcOH (2.5 mL), in 94% yield (0.132 g) as a yellow solid (m.p.: 225-226 ºC; lit. m.p.: 230 ºC, *ref.* [37]).

FTIR (ATR): ν 3142, 3005, 2965, 2841, 1683, 1683, 1578, 1507, 1439, 1320, 1248, 1194, 1161, 1065, 1015, 821, 800, 676 cm^{-1}.

^{1}H NMR (600 MHz, DMSO-d_6): δ 13.75 (s, 1H), 7.60 (s, 1H), 7.19 (dd, *J* = 8.3, 2.1 Hz, 1H), 7.16 (d, *J* = 2.1 Hz, 1H), 7.13 (d, *J* = 8.3 Hz, 1H), 3.84 (s, 3H), 3.83 (s, 3H) ppm.

^{13}C NMR (151 MHz, DMSO-d_6): δ 195.5, 169.4, 151.2, 149.1, 132.3, 125.7, 124.6, 122.3, 113.4, 112.2, 55.8, 55.6 ppm.

HRMS (ESI-QTOF) *m/z* calculated for $C_{12}H_{10}NO_3S_2^-$: 280.0108 $[M-H]^-$; found 280.0099.

(*Z*)-5-(Benzo[*d*][1,3]dioxol-5-ylmethylene)-2-thioxothiazolidin-4-one (**3u**)

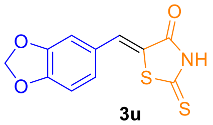

3u was obtained from piperonal (0.50 mmol; 0.076 g), rhodanine (0.50 mmol; 0.067 g), EDA (0.05 mmol; 0.003 mL) in AcOH (2.5 mL), in 95% yield (0.127 g) as a yellow solid (m.p.: 278-280 ºC; lit. m.p.: 278-279 ºC, *ref.* [26]).

FTIR (ATR): ν 3143, 3045, 2830, 1686, 1578, 1496, 1427, 1367, 1307, 1262, 1189, 1096, 1032, 917, 845, 822, 800, 678, 662 cm^{-1}.

^{1}H NMR (600 MHz, DMSO-d_6): δ 13.77 (s, 1H), 7.57 (s, 1H), 7.17 (dd, *J* = 8.2, 1.9 Hz, 1H), 7.13 – 7.09 (m, 2H), 6.15 (s, 2H) ppm.

^{13}C NMR (151 MHz, DMSO-d_6): δ 195.9, 169.9, 150.2, 148.8, 132.4, 127.6, 127.2, 123.4, 110.0, 109.8, 102.6 ppm.

HRMS (ESI-QTOF) *m/z* calculated for $C_{11}H_6NO_3S_2^-$: 263.9795 [M−H]$^-$; found 263.9791.

(*Z*)-5-(Furan-2-ylmethylene)-2-thioxothiazolidin-4-one (**3v**)

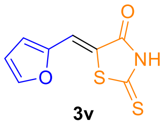

3v was obtained from furfural (0.50 mmol; 0.041 mL), rhodanine (0.50 mmol; 0.067 g), EDA (0.05 mmol; 0.003 mL) in AcOH (2.5 mL), in 94% yield (0.105 g) as a yellow solid (m.p.: 225-227 ºC; lit. m.p.: 228-229 ºC, *ref.* [26]).

FTIR (ATR): ν 3125, 3032, 2847, 1682, 1598, 1461, 1441, 1320, 1254, 1221, 1181, 1087, 1036, 1014, 939, 904, 880, 811, 758, 733, 693, 677 cm^{-1}.

^{1}H NMR (600 MHz, DMSO-d_6): δ 13.69 (s, 1H), 8.11 (d, *J* = 1.8 Hz, 1H), 7.49 (s, 1H), 7.18 (d, *J* = 3.6 Hz, 1H), 6.78 (dd, *J* = 3.6, 1.8 Hz, 1H) ppm.

^{13}C NMR (151 MHz, DMSO-d_6): δ 196.5, 169.0, 149.5, 148.3, 122.5, 119.9, 117.8, 114.0 ppm.

HRMS (ESI-QTOF) *m/z* calculated for $C_8H_4NO_2S_2^-$: 209.9689 [M−H]$^-$; found 209.9689.

(*Z*)-5-(Thiophen-2-ylmethylene)-2-thioxothiazolidin-4-one (**3w**)

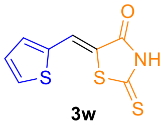

3w was obtained from thiophene-2-carbaldehyde (0.50 mmol; 0.047 mL), rhodanine (0.50 mmol; 0.067 g), EDA (0.05 mmol; 0.003 mL) in AcOH (2.5 mL), in 99% yield (0.112 g) as a yellow solid (m.p.: 208-210 ºC; lit. m.p.: 210 ºC, *ref.* [38]).

FTIR (ATR): ν 3130, 3058, 2838, 1677, 1577, 1433, 1410, 1361, 1304, 1223, 1186, 1063, 1016, 900, 854, 831, 786, 733, 707, 674 cm^{-1}.

^{1}H NMR (600 MHz, DMSO-d_6): δ 13.81 (s, 1H), 8.09 (d, *J* = 5.0 Hz, 1H), 7.93 (s, 1H), 7.72 (d, *J* = 3.8 Hz, 1H), 7.31 (dd, *J* = 5.0, 3.8 Hz, 1H) ppm.

^{13}C NMR (151 MHz, DMSO-d_6): δ 194.7, 169.1, 137.5, 135.5, 134.4, 129.4, 124.8, 123.0 ppm.

HRMS (ESI-QTOF) *m/z* calculated for $C_8H_4NOS_3^-$: 225.9460 [M−H]$^-$; found 225.9460.

(*Z*)-5-((1*H*-Indol-3-yl)methylene)-2-thioxothiazolidin-4-one (**3x**)

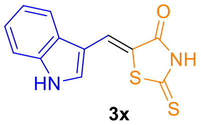

3x

3x was obtained from indole-3-carboxaldehyde (0.50 mmol; 0.073 g), rhodanine (0.50 mmol; 0.067 g), EDA (0.05 mmol; 0.003 mL) in AcOH (2.5 mL), in 92% yield (0.120 g) as an orange solid (m.p.: 287-288 ºC; lit. m.p.: 285-289 ºC, *ref.* [30]).

FTIR (ATR): ν 3258, 2980, 2846, 1674, 1559, 1508, 1432, 1291, 1207, 1138, 1010, 908, 761, 740, 683 cm^{-1}.

^{1}H NMR (600 MHz, DMSO-d_6): δ 13.56 (s, 1H), 12.30 (s, 1H), 7.94 (s, 1H), 7.92 (s, 1H), 7.82 (d, *J* = 3.0 Hz, 1H), 7.52 (d, *J* = 7.5 Hz, 1H), 7.27 (t, *J* = 7.5 Hz, 1H), 7.22 (t, *J* = 7.5 Hz, 1H) ppm.

^{13}C NMR (151 MHz, DMSO-d_6): δ 194.7, 169.1, 136.4, 130.1, 126.8, 124.8, 123.3, 121.4, 118.5, 117.9, 112.6, 110.9 ppm.

HRMS (ESI-QTOF) *m/z* calculated for $C_{12}H_7N_2OS_2^-$: 259.0005 $[M-H]^-$; found 259.0005.

(*Z*)-4-((4-Oxo-2-thioxothiazolidin-5-ylidene)methyl)benzaldehyde (**3y**)

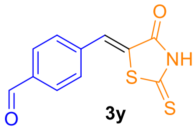

3y

3y was obtained from terephthalaldehyde (0.50 mmol; 0.067 g), rhodanine (0.50 mmol; 0.067 g), EDA (0.05 mmol; 0.003 mL) in AcOH (2.5 mL), in 93% yield (0.116 g) as an orange solid (m.p.: 280 ºC (dec.); lit. m.p.: not reported).

FTIR (ATR): ν 3083, 3021, 2846, 2940, 1712, 1678, 1656, 1595, 1448, 1290, 1231, 1192, 1061, 1003, 923, 860, 836, 812, 748, 700, 676 cm^{-1}.

^{1}H NMR (600 MHz, DMSO-d_6): δ 13.84 (s, 1H), 9.94 (s, 1H), 7.91 (d, *J* = 8.0 Hz, 2H), 7.69 (d, *J* = 8.0 Hz, 2H), 7.58 (s, 1H) ppm.

^{13}C NMR (151 MHz, DMSO-d_6): δ 195.6, 192.6, 169.4, 138.4, 136.5, 130.9, 130.2, 129.7 ppm.

HRMS (ESI-QTOF) *m/z* calculated for $C_{11}H_6NO_2S_2^-$: 247.9845 $[M-H]^-$; found 247.9847.

(*Z*)-5-(2-Methylpropylidene)-2-thioxothiazolidin-4-one (**3z**)

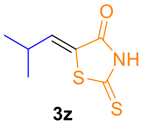

3z

3z was obtained from isobutyraldehyde (0.50 mmol; 0.061 mL), rhodanine (0.50 mmol; 0.067 g), EDA (0.05 mmol; 0.003 mL) in AcOH (2.5 mL), in 72% yield (0.068 g) as a yellow solid (m.p.: 89-90 ºC; lit. m.p.: not reported).

FTIR (ATR): ν 3147, 3041, 2963, 2843, 1693, 1616, 1430, 1336, 1301, 1207, 1101, 1067, 952, 804, 727, 670 cm^{-1}.

^{1}H NMR (600 MHz, DMSO-d_6): δ 13.48 (s, 1H), 6.56 (d, *J* = 9.5 Hz, 1H), 2.26 (dhept, *J* = 9.5, 6.7 Hz, 1H), 0.96 (d, *J* = 6.7 Hz, 6H) ppm.

^{13}C NMR (151 MHz, DMSO-d_6): δ 196.1, 168.1, 143.1, 127.1, 31.7, 20.9 ppm.

HRMS (ESI-QTOF) *m/z* calculated for $C_7H_8NOS_2^-$: 186.0053 $[M-H]^-$; found 186.0050.

(*Z*)-5-Benzylidenethiazolidine-2,4-dione (**4a**)

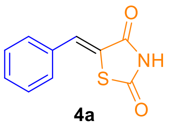

4a

4a was obtained from benzaldehyde (0.50 mmol; 0.051 mL), thiazolidine-2,4-dione (1.00 mmol; 0.117 g), EDA (0.05 mmol; 0.003 mL) in AcOH (2.5 mL), in 99% yield (0.101 g) as a white solid (m.p.: 240-242 °C; lit. m.p.: 242-244 °C, *ref.* [24]).

FTIR (ATR): ν 3114, 3008, 2779, 1736, 1683, 1606, 1593, 1491, 1446, 1332, 1296, 1284, 1164, 1019, 919, 809, 759, 680 cm^{-1}.

^{1}H NMR (600 MHz, DMSO-d_6): δ 12.64 (s, 1H), 7.80 (s, 1H), 7.60 (d, J = 7.5 Hz, 2H), 7.54 (t, J = 7.5 Hz, 2H), 7.51 – 7.46 (m, 1H) ppm.

^{13}C NMR (151 MHz, DMSO-d_6): δ 167.9, 167.3, 133.0, 131.8, 130.5, 130.0, 129.3, 123.6 ppm.

HRMS (ESI-QTOF) *m/z* calculated for $C_{10}H_6NO_2S^-$: 204.0125 [M−H]$^-$; found 204.0123.

(*Z*)-5-(2-Fluorobenzylidene)thiazolidine-2,4-dione (**4b**)

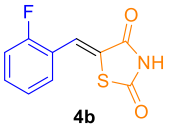

4b

4b was obtained from 2-fluorobenzaldehyde (0.50 mmol; 0.053 mL), thiazolidine-2,4-dione (1.00 mmol; 0.117 g), EDA (0.05 mmol; 0.003 mL) in AcOH (2.5 mL), in 93% yield (0.103 g) as a white solid (m.p.: 223-224 °C; lit. m.p.: 225.7-226.8 °C, *ref.* [39]).

FTIR (ATR): ν 3143, 3000, 2779, 1735, 1683, 1608, 1482, 1454, 1334, 1289, 1234, 1191, 1152, 1104, 1020, 907, 849, 802, 753, 685 cm^{-1}.

^{1}H NMR (600 MHz, DMSO-d_6): δ 12.73 (s, 1H), 7.79 (s, 1H), 7.60 – 7.52 (m, 2H), 7.42 – 7.36 (m, 2H) ppm.

^{13}C NMR (151 MHz, DMSO-d_6): δ 167.6, 167.0, 160.5 (d, J = 251.9 Hz), 132.7 (d, J = 8.8 Hz), 128.8, 126.3, 125.4 (d, J = 3.4 Hz), 122.7 (d, J = 6.4 Hz), 120.9 (d, J = 11.7 Hz), 116.2 (d, J = 21.4 Hz) ppm.

HRMS (ESI-QTOF) *m/z* calculated for $C_{10}H_5FNO_2S^-$: 222.0031 [M−H]$^-$; found 222.0029.

(*Z*)-5-(2-Chlorobenzylidene)thiazolidine-2,4-dione (**4c**)

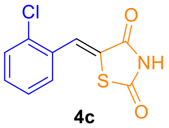

4c

4c was obtained from 2-chlorobenzaldehyde (0.50 mmol; 0.056 mL), thiazolidine-2,4-dione (1.00 mmol; 0.117 g), EDA (0.05 mmol; 0.003 mL) in AcOH (2.5 mL), in 84% yield (0.101 g) as a white solid (m.p.: 190-191 °C; lit. m.p.: 210 °C, *ref.* [41]).

FTIR (ATR): ν 3143, 3049, 2980, 2771, 1734, 1712, 1682, 1605, 1463, 1432, 1319, 1216, 1163, 1038, 1009, 940, 894, 768, 749, 694 cm^{-1}.

^{1}H NMR (600 MHz, DMSO-d_6): δ 12.77 (s, 1H), 7.93 (s, 1H), 7.65 (dd, J = 7.3, 1.9 Hz, 1H), 7.58 (dd, J = 7.3, 1.9 Hz, 1H), 7.55 – 7.48 (m, 2H) ppm.

^{13}C NMR (151 MHz, DMSO-d_6): δ 167.6, 167.0, 134.4, 131.8, 131.0, 130.3, 128.9, 128.1, 127.3, 126.7 ppm.

HRMS (ESI-QTOF) *m/z* calculated for $C_{10}H_5ClNO_2S^-$: 237.9735 [M−H]$^-$; found 237.9735.

(*Z*)-5-(2-Bromobenzylidene)thiazolidine-2,4-dione (**4d**)

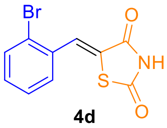

4d

4d was obtained from 2-bromobenzaldehyde (0.50 mmol; 0.058 mL), thiazolidine-2,4-dione (1.00 mmol; 0.117 g), EDA (0.05 mmol; 0.003 mL) in AcOH (2.5 mL), in 85% yield (0.121 g) as a white solid (m.p.: 160-161 ºC; lit. m.p.: 152 °CºC, *ref.* [41]).

FTIR (ATR): ν 3117, 3050, 2980, 2762, 1738, 1697, 1604, 1458, 1428, 1313, 1277, 1164, 1023, 1004, 896, 774, 749, 693 cm^{-1}.

^{1}H NMR (600 MHz, DMSO-d_6): δ 12.76 (s, 1H), 7.89 (s, 1H), 7.81 (dd, *J* = 8.0, 0.9 Hz, 1H), 7.60 – 7.54 (m, 2H), 7.41 (ddd, *J* = 8.0, 5.5, 3.5 Hz, 1H) ppm.

^{13}C NMR (151 MHz, DMSO-d_6): δ 167.6, 166.9, 133.6, 132.7, 132.0, 129.5, 129.0, 128.6, 127.2, 125.3 ppm.

HRMS (ESI-QTOF) *m/z* calculated for $C_{10}H_5BrNO_2S^-$: 283.9209 [M−H]$^-$; found 283.9195.

(*Z*)-5-(2-Nitrobenzylidene)thiazolidine-2,4-dione (**4e**)

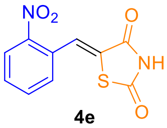

4e

4e was obtained from 2-nitrobenzaldehyde (0.50 mmol; 0.076 g), thiazolidine-2,4-dione (1.00 mmol; 0.117 g), EDA (0.05 mmol; 0.003 mL) in AcOH (2.5 mL), in 57% yield (0.071 g) as a beige off-white solid (m.p.: 181-182 ºC; lit. m.p.: 195-197 ºC, *ref.* [40]).

FTIR (ATR): ν 3157, 3103, 3048, 2765, 1734, 1719, 1698, 1609, 1570, 1519, 1439, 1342, 1315, 1213, 1162, 1076, 1006, 868, 839, 786, 750, 689 cm^{-1}.

^{1}H NMR (600 MHz, DMSO-d_6): δ 12.78 (s, 1H), 8.21 (dd, *J* = 7.9, 1.3 Hz, 1H), 8.02 (s, 1H), 7.90 (td, *J* = 7.9, 1.3 Hz, 1H), 7.77 – 7.70 (m, 2H) ppm.

^{13}C NMR (151 MHz, DMSO-d_6): δ 167.6, 166.6, 147.9, 134.5, 131.0, 129.2, 128.9, 128.5, 128.2, 125.4 ppm.

HRMS (ESI-QTOF) *m/z* calculated for $C_{10}H_5N_2O_4S^-$: 248.9976 [M−H]$^-$; found 248.9967.

(*Z*)-5-(2-Hydroxybenzylidene)thiazolidine-2,4-dione (**4f**)

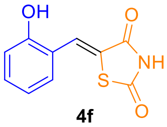

4f

4f was obtained from salicylaldehyde (0.50 mmol; 0.052 mL), thiazolidine-2,4-dione (1.00 mmol; 0.117 g), EDA (0.05 mmol; 0.003 mL) in AcOH (2.5 mL), in 64% yield (0.070 g) as a yellow solid (m.p.: 239-240 ºC; lit. m.p.: 235-237 ºC, *ref.* [42]).

FTIR (ATR): ν 3407, 3126, 2990, 2788, 1721, 1668, 1590, 1455, 1327, 1246, 1193, 1166, 1015, 808, 751, 738, 687 cm^{-1}.

^{1}H NMR (600 MHz, DMSO-d_6): δ 12.51 (s, 1H), 10.50 (s, 1H), 8.03 (s, 1H), 7.36 – 7.29 (m, 2H), 7.00 – 6.92 (m, 2H) ppm.

^{13}C NMR (151 MHz, DMSO-d_6): δ 168.2, 167.5, 157.3, 132.2, 128.3, 127.0, 121.9, 119.9, 119.7, 116.1 ppm.

HRMS (ESI-QTOF) *m/z* calculated for $C_{10}H_6NO_3S^-$: 220.0074 [M−H]$^-$; found 220.0074.

(*Z*)-5-(4-Chlorobenzylidene)thiazolidine-2,4-dione (**4g**)

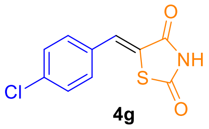

4g

4g was obtained from 4-chlorobenzaldehyde (0.50 mmol; 0.070 g), thiazolidine-2,4-dione (1.00 mmol; 0.117 g), EDA (0.05 mmol; 0.003 mL) in AcOH (2.5 mL), in 92% yield (0.110 g) as a white solid (m.p.: 225-227 °C; lit. m.p.: 225-228 °C, *ref.* [24]).

FTIR (ATR): ν 3144, 3051, 2765, 1718, 1609, 1586, 1487, 1401, 1324, 1168, 1086, 1011, 898, 816, 766, 698 cm^{-1}.

^{1}H NMR (600 MHz, DMSO-d_6): δ 12.67 (s, 1H), 7.79 (s, 1H), 7.64 – 7.57 (m, 4H) ppm.

^{13}C NMR (151 MHz, DMSO-d_6): δ 167.7, 167.2, 135.0, 132.0, 131.7, 130.5, 129.4, 124.4 ppm.

HRMS (ESI-QTOF) *m/z* calculated for $C_{10}H_5ClNO_2S^-$: 237.9735 [M−H]$^-$; found 237.9735.

(*Z*)-5-(4-Bromobenzylidene)thiazolidine-2,4-dione (**4h**)

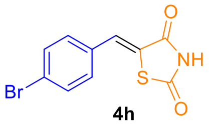

4h

4h was obtained from 4-bromobenzaldehyde (0.50 mmol; 0.092 g), thiazolidine-2,4-dione (1.00 mmol; 0.117 g), EDA (0.05 mmol; 0.003 mL) in AcOH (2.5 mL), in 99% yield (0.142 g) as a white solid (m.p.: 237-238 °C; lit. m.p.: 241.5-242.5 °C, *ref.* [43]).

FTIR (ATR): ν 3143, 3051, 2766, 1716, 1609, 1484, 1397, 1323, 1166, 1070, 1008, 898, 814, 768, 696, 660 cm^{-1}.

^{1}H NMR (600 MHz, DMSO-d_6): δ 12.67 (s, 1H), 7.78 (s, 1H), 7.77 – 7.72 (m, 2H), 7.57 – 7.52 (m, 2H) ppm.

^{13}C NMR (151 MHz, DMSO-d_6): δ 167.6, 167.2, 132.3, 132.3, 132.3, 131.8, 130.5, 124.4, 123.9 ppm.

HRMS (ESI-QTOF) *m/z* calculated for $C_{10}H_5BrNO_2S^-$: 283.9209 [M−H]$^-$; found 283.9206.

(*Z*)-4-((2,4-Dioxothiazolidin-5-ylidene)methyl)benzonitrile (**4i**)

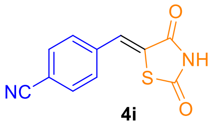

4i

4i was obtained from 4-formylbenzonitrile (0.50 mmol; 0.066 g), thiazolidine-2,4-dione (1.00 mmol; 0.117 g), EDA (0.05 mmol; 0.003 mL) in AcOH (2.5 mL), in 95% yield (0.110 g) as a yellow solid (m.p.: 258-260 °C; lit. m.p.: not reported).

FTIR (ATR): ν 3123, 3011, 2758, 2226, 1742, 1686, 1597, 1501, 1413, 1320, 1282, 1211, 1148, 1020, 916, 810, 686 cm^{-1}.

^{1}H NMR (600 MHz, DMSO-d_6): δ 12.78 (s, 1H), 7.99 (d, J = 8.2 Hz, 2H), 7.85 (s, 1H), 7.78 (d, J = 8.2 Hz, 2H) ppm.

^{13}C NMR (151 MHz, DMSO-d_6): δ 167.4, 167.1, 137.5, 133.0, 130.4, 129.6, 127.3, 118.4, 112.0 ppm.

HRMS (ESI-QTOF) *m/z* calculated for $C_{11}H_5N_2O_2S^-$: 229.0077 [M−H]$^-$; found 229.0077.

(*Z*)-5-(4-Nitrobenzylidene)thiazolidine-2,4-dione (**4j**)

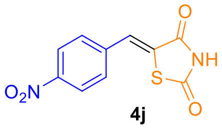

4j

4j was obtained from 4-nitrobenzaldehyde (0.50 mmol; 0.076 g), thiazolidine-2,4-dione (1.00 mmol; 0.117 g), EDA (0.05 mmol; 0.003 mL) in AcOH (2.5 mL), in 81% yield (0.101 g) as a white solid (m.p.: 239-240 ºC; lit. m.p.: 240-241 ºC, *ref.* [44]).

FTIR (ATR): ν 3251, 3027, 2980, 1743, 1711, 1608, 1590, 1507, 1334, 1308, 1135, 1106, 1008, 919, 868, 843, 755, 731, 678, 662 cm^{-1}.

^{1}H NMR (600 MHz, DMSO-d_6): δ 12.81 (s, 1H), 8.36 – 8.31 (m, 2H), 7.89 (s, 1H), 7.88 – 7.83 (m, 2H) ppm.

^{13}C NMR (151 MHz, DMSO-d_6): δ 167.4, 167.0, 147.4, 139.4, 130.9, 129.1, 128.0, 124.2 ppm.

HRMS (ESI-QTOF) *m/z* calculated for $C_{10}H_5N_2O_4S^-$: 248.9976 [M−H]$^-$; found 248.9974.

(Z)-5-(4-Methylbenzylidene)thiazolidine-2,4-dione (**4k**)

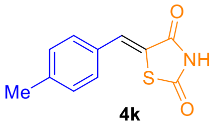

4k

4k was obtained from *p*-tolualdehyde (0.50 mmol; 0.059 mL), thiazolidine-2,4-dione (1.00 mmol; 0.117 g), EDA (0.05 mmol; 0.003 mL) in AcOH (2.5 mL), in 99% yield (0.109 g) as a white solid (m.p.: 226-228 ºC; lit. m.p.: 228.9-229.2 ºC, *ref.* [43]).

FTIR (ATR): ν 3157, 3046, 2980, 2767, 1736, 1683, 1597, 1508, 1326, 1287, 1153, 1022, 902, 806, 687 cm^{-1}.

^{1}H NMR (600 MHz, DMSO-d_6): δ 12.68 (s, 1H), 7.86 (s, 1H), 7.60 (dd, *J* = 8.2, 1.9 Hz, 2H), 7.45 (d, *J* = 8.0 Hz, 2H), 2.47 (s, 3H) ppm.

^{13}C NMR (151 MHz, DMSO-d_6): δ 167.9, 167.4, 140.7, 131.9, 130.3, 130.1, 129.9, 122.3, 21.1 ppm.

HRMS (ESI-QTOF) *m/z* calculated for $C_{11}H_8NO_2S^-$: 218.0281 [M−H]$^-$; found 218.0288.

(*Z*)-5-(4-Hydroxybenzylidene)thiazolidine-2,4-dione (**4l**)

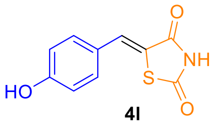

4l

4l was obtained from 4-hydroxybenzaldehyde (0.50 mmol; 0.061 g), thiazolidine-2,4-dione (1.00 mmol; 0.117 g), EDA (0.05 mmol; 0.003 mL) in AcOH (2.5 mL), in 91% yield (0.101 g) as a yellow solid (m.p.: 288-290 ºC; lit. m.p.: 282-285 ºC, *ref.* [45]).

FTIR (ATR): ν 3399, 3122, 2980, 2788, 1717, 1672, 1571, 1508, 1278, 1337, 1210, 1153, 1022, 899, 822, 693 cm^{-1}.

^{1}H NMR (600 MHz, DMSO-d_6): δ 12.46 (s, 1H), 10.30 (s, 1H), 7.71 (s, 1H), 7.49 – 7.43 (m, 2H), 6.95 – 6.89 (m, 2H) ppm.

^{13}C NMR (151 MHz, DMSO-d_6): δ 168.1, 167.5, 159.9, 132.4, 132.3, 123.9, 119.0, 116.3 ppm.

HRMS (ESI-QTOF) *m/z* calculated for $C_{10}H_6NO_3S^-$: 220.0074 [M−H]$^-$; found 220.0079.

(*Z*)-5-(4-(Dimethylamino)benzylidene)thiazolidine-2,4-dione (**4m**)

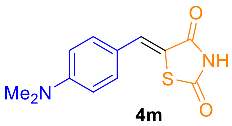

4m was obtained from 4-(dimethylamino)benzaldehyde (0.50 mmol; 0.075 g), thiazolidine-2,4-dione (1.00 mmol; 0.117 g), EDA (0.05 mmol; 0.003 mL) in AcOH (2.5 mL), in 55% yield (0.068 g) as an orange solid (m.p.: 280-281 ºC; lit. m.p.: 280-281 ºC, *ref*. [46]).

FTIR (ATR): ν 3096, 3000, 2765, 1723, 1679, 1612, 1558, 1519, 1381, 1338, 1300, 1188, 1137, 1030, 945, 901, 804, 689 cm^{-1}.

^{1}H NMR (600 MHz, DMSO-d_6): δ 12.20 (s, 1H), 7.55 (s, 1H), 7.32 – 7.27 (m, 2H), 6.72 – 6.67 (m, 2H), 2.90 (s, 6H) ppm.

^{13}C NMR (151 MHz, DMSO-d_6): δ 168.1, 167.5, 151.4, 132.9, 132.1, 119.8, 115.6, 112.0, 39.6 ppm.

HRMS (ESI-QTOF) *m/z* calculated for $C_{12}H_{11}N_2O_2S^-$: 247.0547 [M−H]$^-$; found 247.0552.

(Z)-5-(4-(Diethylamino)benzylidene)thiazolidine-2,4-dione (**4n**)

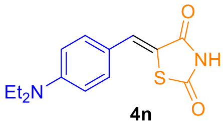

4n was obtained from 4-(diethylamino)benzaldehyde (0.50 mmol; 0.089 g), thiazolidine-2,4-dione (1.00 mmol; 0.117 g), EDA (0.05 mmol; 0.003 mL) in AcOH (2.5 mL), in 40% yield (0.056 g) as an orange solid (m.p.: 188-190 ºC; lit. m.p.: not reported).

FTIR (ATR): ν 3121, 3096, 3029, 2969, 2759, 1722, 1672, 1614, 1570, 1520, 1412, 1333, 1303, 1272, 1193, 1145, 1011, 914, 811, 792, 684 cm^{-1}.

^{1}H NMR (600 MHz, DMSO-d_6): δ 12.37 (s, 1H), 7.68 (s, 1H), 7.47 (s, 2H), 6.90 (s, 2H), 3.45 (q, *J* = 7.0 Hz, 4H), 1.11 (t, *J* = 7.0 Hz, 6H) ppm.

^{13}C NMR (151 MHz, DMSO-d_6): δ 168.1, 167.5, 132.4, 12.0 ppm.

HRMS (ESI-QTOF) *m/z* calculated for $C_{14}H_{15}N_2O_2S^-$: 275.0860 [M−H]$^-$; found 275.0865.

(*Z*)-5-(2,4-Dimethoxybenzylidene)thiazolidine-2,4-dione (**4p**)

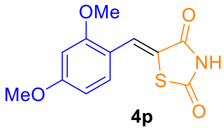

4p was obtained from 2,4-dimethoxybenzaldehyde (0.50 mmol; 0.083 g), thiazolidine-2,4-dione (1.00 mmol; 0.117 g), EDA (0.05 mmol; 0.003 mL) in AcOH (2.5 mL), in 97% yield (0.129 g) as a yellow solid (m.p.: 242-244 ºC; lit. m.p.: 240-242 ºC, *ref*. [47]).

FTIR (ATR): ν 3100, 2976, 2948, 2842, 2780, 1766, 1717, 1683, 1608, 1569, 1461, 1353, 1313, 1264, 1211, 1162, 1117, 1020, 921, 823, 794, 696 cm^{-1}.

^{1}H NMR (600 MHz, DMSO-d_6): δ 12.54 (s, 1H), 8.03 (s, 1H), 7.45 (d, *J* = 8.6 Hz, 1H), 6.81 (dd, *J* = 8.6, 2.4 Hz, 1H), 6.79 (d, *J* = 2.4 Hz, 1H), 4.00 (s, 3H), 3.95 (s, 3H) ppm.

^{13}C NMR (151 MHz, DMSO-d_6): δ 168.2, 167.5, 163.1, 159.8, 130.1, 126.5, 119.9, 114.3, 106.5, 98.6, 55.9, 55.6 ppm.

HRMS (ESI-QTOF) *m/z* calculated for $C_{12}H_{10}NO_4S^-$: 264.0336 [M−H]$^-$; found 264.0345.

(*Z*)-3-((2,4-Dioxothiazolidin-5-ylidene)methyl)benzonitrile (**4q**)

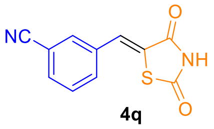

4q

4q was obtained from 3-formylbenzonitrile (0.50 mmol; 0.066 g), thiazolidine-2,4-dione (1.00 mmol; 0.117 g), EDA (0.05 mmol; 0.003 mL) in AcOH (2.5 mL), in 91% yield (0.105 g) as a white solid (m.p.: 238-240 ºC; lit. m.p.: not reported).

FTIR (ATR): ν 3126, 2997, 2775, 2233, 1739, 1685, 1605, 1415, 1327, 1171, 1150, 1022, 914, 802, 676 cm^{-1}.

^{1}H NMR (600 MHz, DMSO-d_6): δ 12.74 (s, 1H), 8.05 (t, *J* = 1.5 Hz, 1H), 7.92 (dt, *J* = 8.0, 1.5 Hz, 1H), 7.87 (dt, *J* = 8.0, 1.5 Hz, 1H), 7.81 (s, 1H), 7.73 (t, *J* = 8.0 Hz, 1H) ppm.

^{13}C NMR (151 MHz, DMSO-d_6): δ 167.4, 167.1, 134.3, 133.9, 133.3, 133.3, 130.5, 129.4, 126.2, 118.1, 112.5 ppm.

HRMS (ESI-QTOF) *m/z* calculated for $C_{11}H_5N_2O_2S^-$: 229.0077 [M−H]$^-$; found 229.0077.

(Z)-5-(3-Nitrobenzylidene)thiazolidine-2,4-dione (**4r**)

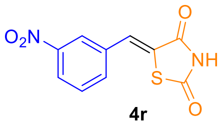

4r

4r was obtained from 3-nitrobenzaldehyde (0.50 mmol; 0.076 g), thiazolidine-2,4-dione (1.00 mmol; 0.117 g), EDA (0.05 mmol; 0.003 mL) in AcOH (2.5 mL), in 83% yield (0.104 g) as a white solid (m.p.: 186-188 ºC; lit. m.p.: 178-179 ºC, *ref*. [46]).

FTIR (ATR): ν 3159, 3044, 2765, 1745, 1688, 1606, 1533, 1353, 1326, 1291, 1154, 1019, 929, 803, 741, 701, 668 cm^{-1}.

^{1}H NMR (600 MHz, DMSO-d_6): δ 12.78 (s, 1H), 8.44 (t, *J* = 2.2 Hz, 1H), 8.29 (ddd, *J* = 8.0, 2.2, 1.0 Hz, 1H), 8.01 (dt, *J* = 8.0, 1.0 Hz, 1H), 7.95 (s, 1H), 7.82 (t, *J* = 8.0 Hz, 1H) ppm.

^{13}C NMR (151 MHz, DMSO-d_6): δ 167.3, 167.0, 148.2, 135.4, 134.8, 130.9, 129.4, 126.6, 124.4, 124.3 ppm.

HRMS (ESI-QTOF) *m/z* calculated for $C_{10}H_5N_2O_4S^-$: 248.9976 [M−H]$^-$; found 248.9981.

(*Z*)-5-(3-Hydroxybenzylidene)thiazolidine-2,4-dione (**4s**)

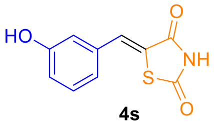

4s

4s was obtained from 3-hydroxybenzaldehyde (0.50 mmol; 0.061 g), thiazolidine-2,4-dione (1.00 mmol; 0.117 g), EDA (0.05 mmol; 0.003 mL) in AcOH (2.5 mL), in 81% yield (0.090 g) as a white solid (m.p.: 264-265 ºC; lit. m.p.: 273-274 ºC, *ref*. [48]).

FTIR (ATR): ν 3295, 3165, 1746, 1683, 1614, 1585, 1449, 1361, 1337, 1305, 1281, 1231, 1178, 1143, 1022, 993, 958, 905, 857, 791, 734, 678 cm^{-1}.

^{1}H NMR (600 MHz, DMSO-d_6): δ 12.60 (s, 1H), 9.84 (s, 1H), 7.70 (s, 1H), 7.33 (t, *J* = 8.0 Hz, 1H), 7.04 (dt, *J* = 8.0, 1.0 Hz, 1H), 6.98 (t, *J* = 2.2 Hz, 1H), 6.89 (ddd, *J* = 8.0, 2.2, 1.0 Hz, 1H) ppm.

^{13}C NMR (151 MHz, DMSO-d_6): δ 168.0, 167.3, 157.9, 134.2, 132.0, 130.4, 123.3, 121.3, 117.7, 115.9 ppm.

HRMS (ESI-QTOF) *m/z* calculated for $C_{10}H_6NO_3S^-$: 220.0074 [M−H]$^-$; found 220.0079.

(*Z*)-5-(3,4-Dimethoxybenzylidene)thiazolidine-2,4-dione (**4t**)

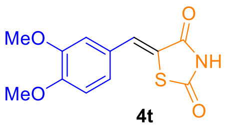

4t

4t was obtained from 3,4-dimethoxybenzaldehyde (0.50 mmol; 0.083 g), thiazolidine-2,4-dione (1.00 mmol; 0.117 g), EDA (0.05 mmol; 0.003 mL) in AcOH (2.5 mL), in 87% yield (0.115 g) as a light yellow solid (m.p.: 211-213 ºC; lit. m.p.: 208-210 ºC, *ref*. [49]).

FTIR (ATR): ν 3208, 2932, 2841, 1734, 1697, 1578, 1510, 1439, 1301, 1259, 1135, 1011, 951, 903, 790, 769, 682 cm^{-1}.

^{1}H NMR (600 MHz, DMSO-d_6): δ 12.52 (s, 1H), 7.75 (s, 1H), 7.21 – 7.16 (m, 2H), 7.15 – 7.10 (m, 1H), 3.84 (s, 3H), 3.82 (s, 3H) ppm.

^{13}C NMR (151 MHz, DMSO-d_6): δ 167.9, 167.4, 150.8, 148.9, 132.2, 125.7, 123.7, 120.4, 113.3, 112.1, 55.7, 55.5 ppm.

HRMS (ESI-QTOF) *m/z* calculated for $C_{12}H_{10}NO_4S^-$: 264.0336 $[M-H]^-$; found 264.0343.

(*Z*)-5-(Benzo[*d*][1,3]dioxol-5-ylmethylene)thiazolidine-2,4-dione (**4u**)

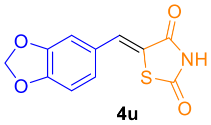

4u

4u was obtained from piperonal (0.50 mmol; 0.076 g), thiazolidine-2,4-dione (1.00 mmol; 0.117 g), EDA (0.05 mmol; 0.003 mL) in AcOH (2.5 mL), in 89% yield (0.111 g) as a yellow solid (m.p.: 228-229 °C; lit. m.p.: 235-236 °C, *ref*. [50]).

FTIR (ATR): ν 3130, 2981, 2911, 2737, 1732, 1692, 1583, 1492, 1371, 1321, 1262, 1151, 1094, 1035, 922, 826, 804, 723, 686, 650 cm^{-1}.

^{1}H NMR (600 MHz, DMSO-d_6): δ 12.54 (s, 1H), 7.72 (s, 1H), 7.15 (dd, *J* = 8.0, 1.8 Hz, 1H), 7.13 (d, *J* = 1.8 Hz, 1H), 7.09 (d, *J* = 8.0 Hz, 1H), 6.14 (s, 2H) ppm.

^{13}C NMR (151 MHz, DMSO-d_6): δ 167.8, 167.3, 149.3, 148.2, 131.9, 127.2, 125.9, 120.9, 109.2, 109.1, 102.0 ppm.

HRMS (ESI-QTOF) *m/z* calculated for $C_{11}H_6NO_4S^-$: 248.0023 $[M-H]^-$; found 248.0029.

(*Z*)-5-(Furan-2-ylmethylene)thiazolidine-2,4-dione (**4v**)

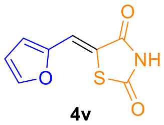

4v

4v was obtained from furfural (0.50 mmol; 0.041 mL), thiazolidine-2,4-dione (1.00 mmol; 0.117 g), EDA (0.05 mmol; 0.003 mL) in AcOH (2.5 mL), in 82% yield (0.080 g) as a grey solid (m.p.: 233-235 °C; lit. m.p.: 234-236 °C, *ref*. [34]).

FTIR (ATR): ν 3128, 3033, 2795, 1680, 1607, 1543, 1470, 1339, 1284, 1218, 1166, 1148, 1020, 928, 885, 824, 754, 684 cm^{-1}.

^{1}H NMR (600 MHz, DMSO-d_6): δ 12.47 (s, 1H), 8.05 (d, *J* = 1.8 Hz, 1H), 7.62 (s, 1H), 7.10 (d, *J* = 3.5 Hz, 1H), 6.75 (dd, *J* = 3.5, 1.8 Hz, 1H) ppm.

^{13}C NMR (151 MHz, DMSO-d_6): δ 168.6, 167.0, 149.2, 147.5, 120.4, 118.6, 118.5, 113.5 ppm.

HRMS (ESI-QTOF) *m/z* calculated for $C_8H_4NO_3S^-$: 193.9917 $[M-H]^-$; found 193.9925.

(*Z*)-5-(Thiophen-2-ylmethylene)thiazolidine-2,4-dione (**4w**)

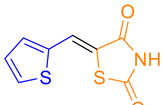
4w

4w was obtained from thiophene-2-carbaldehyde (0.50 mmol; 0.047 mL), thiazolidine-2,4-dione (1.00 mmol; 0.117 g), EDA (0.05 mmol; 0.003 mL) in AcOH (2.5 mL), in 72% yield (0.077 g) as a brown solid (m.p.: 230-232 ºC; lit. m.p.: 222 ºC, *ref.* [51]).

FTIR (ATR): ν 3129, 2980, 2790, 1734, 1672, 1592, 1315, 1153, 1078, 1046, 885, 824, 696, 794 cm^{-1}.

^{1}H NMR (600 MHz, DMSO-d_6): δ 12.57 (s, 1H), 8.07 (s, 1H), 8.02 (d, *J* = 5.0 Hz, 1H), 7.68 (d, *J* = 5.0 Hz, 1H), 7.30 (t, *J* = 5.0 Hz, 1H) ppm.

^{13}C NMR (151 MHz, DMSO-d_6): δ 167.1, 167.0, 137.3, 134.6, 133.1, 128.9, 125.2, 121.0 ppm.

HRMS (ESI-QTOF) *m/z* calculated for $C_8H_4NO_2S_2^-$: 209.9689 [M−H]$^-$; found 209.9694.

(*Z*)-5-((1*H*-Indol-3-yl)methylene)thiazolidine-2,4-dione (**4x**)

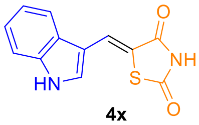
4x

4x was obtained from indole-3-carboxaldehyde (0.50 mmol; 0.073 g), thiazolidine-2,4-dione (1.00 mmol; 0.117 g), EDA (0.05 mmol; 0.003 mL) in AcOH (2.5 mL), in 53% yield (0.065 g) as a yellow solid (m.p.: 290 ºC (dec.); lit. m.p.: 280-282 ºC, *ref.* [52]).

FTIR (ATR): ν 3219, 3112, 2971, 1715, 1686, 1572, 1514, 1352, 1290, 1222, 1160, 1138, 1030, 951, 879, 767, 734, 682 cm^{-1}.

^{1}H NMR (600 MHz, DMSO-d_6): δ 12.31 (s, 1H), 12.14 (s, 1H), 8.07 (s, 1H), 7.90 (d, *J* = 8.0 Hz, 1H), 7.75 (d, *J* = 3.0 Hz, 1H), 7.52 (d, *J* = 8.0 Hz, 1H), 7.26 (t, *J* = 7.5 Hz, 1H), 7.21 (t, *J* = 7.5 Hz, 1H) ppm.

^{13}C NMR (151 MHz, DMSO-d_6): δ 167.7, 167.3, 136.2, 128.6, 126.8, 124.5, 123.1, 121.0, 118.3, 116.2, 112.4, 110.4 ppm.

HRMS (ESI-QTOF) *m/z* calculated for $C_{12}H_7N_2O_2S^-$: 243.0234 [M−H]$^-$; found 243.0241.

1.4.2. Synthesis and characterization of imidazo[1,2-*a*]pyridines

4-(3-(*tert*-Butylamino)imidazo[1,2-*a*]pyridin-2-yl)benzaldehyde (**8a**)

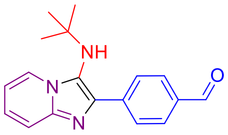
8a

8a was obtained from 2-aminopyridine (2.50 mmol; 0.235 g), terephthalaldehyde (2.50 mmol; 0.335 g), HPW (0.144 g; 2 mol %), *tert*-butyl isocyanide (2.50 mmol; 0.283 mL), in EtOH (2.5 mL) and in 41% yield (0.301 g) as a light yellow solid (m.p.: 135-137 ºC; lit. m.p.: 140-142 ºC, *ref.* [53]) after silica gel column chromatography (30% ethyl acetate/hexane to 50% ethyl acetate/hexane). R_f = 0.52 (50% ethyl acetate/hexane).

FTIR (ATR): ν 3325, 2959, 2832, 2737, 1684, 1601, 1545, 1498, 1438, 1388, 1361, 1332, 1197, 1169, 1098, 1074, 908, 833, 755, 737, 687 cm^{-1}.

^{1}H NMR (600 MHz, DMSO-d_6): δ 10.01 (s, 1H), 8.43 (t, *J* = 7.9 Hz, 3H), 7.94 (d, *J* = 7.9 Hz, 2H), 7.50 (d, *J* = 9.0 Hz, 1H), 7.25 – 7.19 (m, 1H), 6.91 (t, *J* = 6.6 Hz, 1H), 4.79 (s, 1H), 1.02 (s, 9H) ppm.

^{13}C NMR (151 MHz, DMSO-d_6): δ 192.6, 141.5, 141.4, 136.4, 134.5, 129.3, 127.9, 125.6, 124.7, 124.4, 116.9, 111.5, 56.1, 30.0 ppm.

HRMS (ESI-QTOF) *m/z* calculated for $C_{18}H_{20}N_3O^+$: 294.1601 [M+H]$^+$; found 294.1616.

4-(3-(Cyclohexylamino)imidazo[1,2-*a*]pyridin-2-yl)benzaldehyde (**8b**)

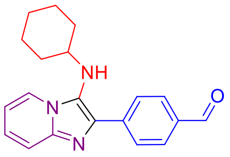

8b

8b was obtained from 2-aminopyridine (2.50 mmol; 0.235 g), terephthalaldehyde (2.50 mmol; 0.335 g), HPW (0.144 g; 2 mol %), cyclohexyl isocyanide (2.50 mmol; 0.311 mL), in EtOH (2.5 mL) in 44% yield (0.350 g) as a light-yellow solid (m.p.: 186-188 ºC; lit. m.p.: 140-142 ºC, *ref.* [53]) after silica gel column chromatography (30% ethyl acetate/hexane to 50% ethyl acetate/hexane). R_f = 0.43 (50% ethyl acetate/hexane).

FTIR (ATR): ν 3291, 3226, 3037, 3099, 3070, 2927, 2850, 2727, 1685, 1599, 1557, 1444, 1333, 1302, 1211, 1167, 1097, 840, 753, 738, 687 cm^{-1}.

^{1}H NMR (600 MHz, $CDCl_3$): δ 10.04 (s, 1H), 8.29 (dt, *J* = 8.4, 1.9 Hz, 2H), 8.13 – 8.06 (m, 1H), 7.98 – 7.93 (m, 2H), 7.55 (dt, *J* = 9.1, 1.2 Hz, 1H), 7.17 (ddd, *J* = 9.1, 6.8, 1.2 Hz, 1H), 6.81 (ddd, *J* = 6.8, 5.8, 1.2 Hz, 1H), 3.12 (d, *J* = 4.9 Hz, 1H), 3.02 – 2.94 (m, 1H), 1.86 – 1.80 (m, 2H), 1.74 – 1.67 (m, 2H), 1.65 – 1.56 (m, 1H), 1.31 – 1.22 (m, 2H), 1.23 – 1.11 (m, 3H) ppm.

^{13}C NMR (151 MHz, $CDCl_3$): δ 192.0, 142.0, 140.8, 135.2, 134.9, 130.1, 130.0, 127.2, 126.3, 124.6, 122.7, 117.8, 112.0, 57.1, 34.3, 25.7, 24.8 ppm.

HRMS (ESI-QTOF) *m/z* calculated for $C_{20}H_{22}N_3O^+$: 320.1757 $[M+H]^+$; found 320.1774.

4-(3-(Phenylamino)imidazo[1,2-*a*]pyridin-2-yl)benzaldehyde (**8c**)

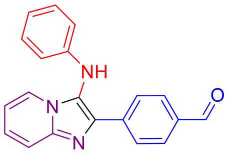

8c

8c was obtained from 2-aminopyridine (2.50 mmol; 0.235 g), terephthalaldehyde (2.50 mmol; 0.335 g), HPW (0.144 g; 2 mol %), phenyl isocyanide (2.50 mmol; 0.265 mL), in EtOH (2.5 mL) in 51% yield (0.400 g) as a yellow solid (m.p.: 169-171 ºC; *unpublished compound*) after silica gel column chromatography (30% ethyl acetate/hexane to 60% ethyl acetate/hexane). R_f = 0.31 (50% ethyl acetate/hexane).

FTIR (ATR): ν 3206, 3084, 3052, 2979, 2911, 1689, 1600, 1496, 1429, 1383, 1340, 1302, 1247, 1207, 838, 755, 732, 684 cm^{-1}.

^{1}H NMR (600 MHz, $CDCl_3$): δ 9.95 (s, 1H), 8.17 (d, *J* = 8.3 Hz, 2H), 7.85 – 7.80 (m, 3H), 7.64 (dd, *J* = 9.2, 1.1 Hz, 1H), 7.28 – 7.19 (m, 3H), 6.89 (t, *J* = 7.5 Hz, 1H), 6.78 (td, *J* = 6.7, 1.1 Hz, 1H), 6.60 (d, *J* = 7.5 Hz, 2H), 5.80 (s, 1H) ppm.

^{13}C NMR (151 MHz, $CDCl_3$): δ 191.9, 144.2, 143.0, 139.4, 137.8, 135.3, 130.0, 130.0, 127.3, 125.7, 122.9, 120.2, 119.5, 117.9, 113.5, 112.7 ppm.

HRMS (ESI-QTOF) *m/z* calculated for $C_{20}H_{16}N_3O^+$: 314.1288 $[M+H]^+$; found 314.1307.

4-(3-(*tert*-Butylamino)-7-chloroimidazo[1,2-*a*]pyridin-2-yl)benzaldehyde (**8d**)

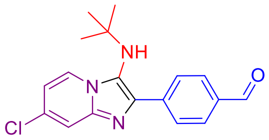

8d

8d was obtained from 2-amino-4-chloropyridine (2.50 mmol; 0.321 g), terephthalaldehyde (2.50 mmol; 0.335 g), HPW (0.144 g; 2 mol %), *tert*-butyl isocyanide (2.50 mmol; 0.283 mL), in EtOH (2.5 mL) in 43% yield (0.352 g) as a light yellow solid (m.p.: 144-146 ºC; *unpublished compound*) after silica gel column chromatography (20% ethyl acetate/hexane to 40% ethyl acetate/hexane). R_f = 0.50 (30% ethyl acetate/hexane).

FTIR (ATR): ν 3669, 3291, 2971, 2901, 1681, 1603, 1386, 1335, 1304, 1210, 1168, 1062, 929, 842, 785, 741 cm^{-1}.

^{1}H NMR (600 MHz, CDCl$_3$): δ 10.04 (s, 1H), 8.17 – 8.12 (m, 3H), 7.95 (dt, *J* = 8.3, 2.0 Hz, 2H), 7.54 (dd, *J* = 2.0, 0.8 Hz, 1H), 6.79 (dd, *J* = 7.3, 2.0 Hz, 1H), 3.09 (s, 1H), 1.06 (s, 9H) ppm.

^{13}C NMR (151 MHz, CDCl$_3$): δ 192.0, 142.0, 141.0, 138.8, 135.3, 131.2, 129.8, 128.4, 128.4, 124.8, 123.9, 116.4, 113.5, 56.8, 30.4 ppm.

HRMS (ESI-QTOF) *m/z* calculated for $C_{18}H_{19}ClN_3O^+$: 328.1211 [M+H]$^+$; found 328.1231.

1.4.3. Synthesis and characterization of imidazo[1,2-*a*]pyridine–thiazolidinone hybrids via Knoevenagel reaction

(*Z*)-5-(4-(3-(*tert*-Butylamino)imidazo[1,2-*a*]pyridin-2-yl)benzylidene)-2-thioxothiazolidin-4-one (**9a**)

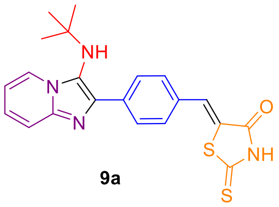

9a

9a was obtained from **8a** product (0.50 mmol; 0.147 g), rhodanine (0.50 mmol; 0.067 g), EDA (0.05 mmol; 0.003 mL) in AcOH (2.5 mL), in 98% yield (0.200 g) as a yellow solid (m.p.: 260-262 ºC; *unpublished compound*).

FTIR (ATR): ν 2954, 2930, 2782, 1704, 1651, 1593, 1439, 1285, 1233, 1200, 1177, 1055, 1004, 903, 766, 668 cm^{-1}.

^{1}H NMR (600 MHz, DMSO-*d*$_6$): δ 8.90 (d, *J* = 6.9 Hz, 1H), 8.35 (d, *J* = 8.3 Hz, 2H), 7.97 – 7.88 (m, 2H), 7.78 (d, *J* = 8.3 Hz, 2H), 7.72 (s, 1H), 7.48 (t, *J* = 6.9 Hz, 1H), 5.39 (s, 1H), 1.05 (s, 9H) ppm.

^{13}C NMR (151 MHz, DMSO-*d*$_6$): δ 195.5, 169.4, 137.7, 133.7, 130.7, 130.5, 128.8, 126.6, 126.1, 116.2, 112.4, 56.7, 29.8 ppm.

HRMS (ESI-QTOF) *m/z* calculated for $C_{21}H_{21}N_4OS_2^+$: 409.1151 [M+H]$^+$; found 409.1158.

(*Z*)-5-(4-(3-(Cyclohexylamino)imidazo[1,2-*a*]pyridin-2-yl)benzylidene)-2-thioxothiazolidin-4-one (**9b**)

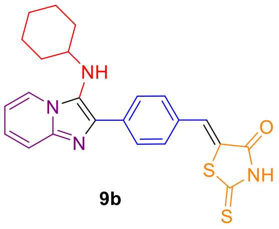

9b

9b was obtained from **8b** product (0.50 mmol; 0.160 g), rhodanine (0.50 mmol; 0.067 g), EDA (0.05 mmol; 0.003 mL) in AcOH (2.5 mL), in 97% yield (0.119 g) as a yellow solid (m.p.: 247-248 ºC; *unpublished compound*).

FTIR (ATR): ν 3186, 2928, 1712, 1653, 1591, 1508, 1432, 1287, 1232, 1185, 1052, 764, 674 cm^{-1}.

^{1}H NMR (600 MHz, DMSO-*d*$_6$): δ 8.79 (d, *J* = 6.8 Hz, 1H), 8.32 (d, *J* = 8.2 Hz, 2H), 7.94 – 7.86 (m, 2H), 7.80 (d, *J* = 8.2 Hz, 2H), 7.70 (s, 1H), 7.48 (t, *J* = 6.8 Hz, 1H), 5.59 (s, 1H), 2.94 – 2.86 (m, 1H), 1.83 – 1.77 (m, 2H), 1.66 – 1.60 (m, 2H), 1.51 – 1.47 (m, 1H), 1.34 – 1.25 (m, 2H), 1.13 – 1.05 (m, 3H) ppm.

^{13}C NMR (151 MHz, DMSO-*d*$_6$): δ 195.4, 169.4, 137.2, 133.4, 131.0, 130.5, 127.9, 127.5, 126.4, 125.4, 116.4, 112.3, 56.6, 33.3, 25.2, 24.5 ppm.

HRMS (ESI-QTOF) *m/z* calculated for $C_{23}H_{23}N_4OS_2^+$: 435.1308 [M+H]$^+$; found 435.1315.

(*Z*)-5-(4-(3-(Phenylamino)imidazo[1,2-*a*]pyridin-2-yl)benzylidene)-2-thioxothiazolidin-4-one (**9c**)

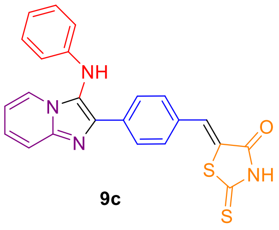

9c

9c was obtained from **8c** product (0.50 mmol; 0.157 g), rhodanine (0.50 mmol; 0.067 g), EDA (0.05 mmol; 0.003 mL) in AcOH (2.5 mL), in 85% yield (0.182 g) as a yellow solid (m.p.: 209-211 ºC; *unpublished compound*).

FTIR (ATR): ν 3177, 2817, 1710, 1651, 1591, 1496, 1427, 1286, 1234, 1180, 1058, 1009, 911, 833, 750, 677 cm^{-1}.

^{1}H NMR (600 MHz, DMSO-*d*$_6$): δ 8.83 (s, 1H), 8.36 (d, *J* = 6.8 Hz, 1H), 8.16 (d, *J* = 8.4 Hz, 2H), 8.02 (d, *J* = 9.0 Hz, 1H), 7.96 – 7.90 (m, 1H), 7.75 (d, *J* = 8.4 Hz, 2H), 7.66 (s, 1H), 7.43 (t, *J* = 6.8 Hz, 1H), 7.19 (t, *J* = 7.8 Hz, 2H), 6.83 (t, *J* = 7.3 Hz, 1H), 6.74 (d, *J* = 7.8 Hz, 2H) ppm.

^{13}C NMR (151 MHz, DMSO-*d*$_6$): δ 195.5, 169.4, 144.0, 138.7, 133.9, 131.1, 130.4, 129.7, 127.5, 126.7, 125.0, 121.4, 119.8, 116.7, 113.7, 113.4 ppm.

HRMS (ESI-QTOF) *m/z* calculated for $C_{23}H_{17}N_4OS_2^+$: 429.0838 [M+H]$^+$; found 429.0848.

(*Z*)-5-(4-(3-(*tert*-Butylamino)-7-chloroimidazo[1,2-*a*]pyridin-2-yl)benzylidene)-2-thioxothiazolidin-4-one (**9d**)

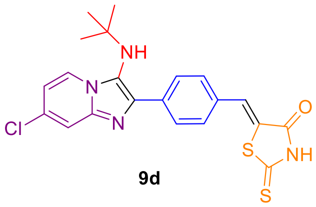

9d

9d was obtained from **8d** product (0.50 mmol; 0.164 g), rhodanine (0.50 mmol; 0.067 g), EDA (0.05 mmol; 0.003 mL) in AcOH (2.5 mL), in 99% yield (0.220 g) as an orange solid (m.p.: 220-222 ºC; *unpublished compound*).

FTIR (ATR): ν 3083, 2971, 2857, 2723, 2687, 2648, 1731, 1714, 1649, 1591, 1450, 1421, 1286, 1183, 1068, 1005, 934, 815, 684 cm^{-1}.

^{1}H NMR (600 MHz, DMSO-*d*$_6$): δ 8.82 (dd, *J* = 7.3, 0.8 Hz, 1H), 8.29 (d, *J* = 8.5 Hz, 2H), 7.99 (dd, *J* = 2.1, 0.8 Hz, 1H), 7.76 (d, *J* = 8.5 Hz, 2H), 7.70 (s, 1H), 7.48 (dd, *J* = 7.3, 2.1 Hz, 1H), 1.03 (s, 9H) ppm.

^{13}C NMR (151 MHz, DMSO-*d*$_6$): δ 195.5, 169.4, 138.2, 136.9, 133.6, 130.7, 130.6, 129.9, 128.7, 127.0, 126.5, 126.4, 116.8, 112.1, 56.7, 29.8 ppm.

HRMS (ESI-QTOF) *m/z* calculated for $C_{21}H_{20}ClN_4OS_2^+$: 443.0762 [M+H]$^+$; found 443.0788.

(*Z*)-5-(4-(3-(*tert*-Butylamino)imidazo[1,2-*a*]pyridin-2-yl)benzylidene)thiazolidine-2,4-dione (**10a**)

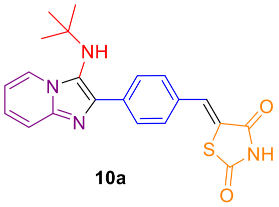

10a

10a was obtained from **8a** product (0.50 mmol; 0.147 g), thiazolidine-2,4-dione (1.00 mmol; 0.117 g), EDA (0.05 mmol; 0.003 mL) in AcOH (2.5 mL), in 87% yield (0.172 g) as a yellow solid (m.p.: 237-238 ºC; *unpublished compound*).

FTIR (ATR): ν 3372, 2970, 2714, 1739, 1697, 1649, 1596, 1509, 1283, 1140, 1011, 897, 825, 769, 681 cm^{-1}.

^{1}H NMR (600 MHz, DMSO-*d*$_6$): δ 12.61 (s, 1H), 8.77 (dd, *J* = 7.0, 1.4 Hz, 1H), 8.19 – 8.13 (m, 2H), 7.84 – 7.80 (m, 2H), 7.74 (s, 1H), 7.67 (d, *J* = 8.3 Hz, 2H), 7.38 (ddd, *J* = 7.0, 4.6, 3.4 Hz, 1H), 5.22 (s, 1H), 0.91 (s, 7H) ppm.

^{13}C NMR (151 MHz, DMSO-d_6): δ 167.7, 167.3, 137.5, 134.0, 133.3, 130.7, 130.3, 129.5, 128.8, 128.2, 126.2, 126.1, 124.8, 116.5, 112.2, 56.6, 29.8 ppm.

HRMS (ESI-QTOF) *m/z* calculated for $C_{21}H_{21}N_4O_2S^+$: 393.1380 $[M+H]^+$; found 393.1387.

(*Z*)-5-(4-(3-(Cyclohexylamino)imidazo[1,2-*a*]pyridin-2-yl)benzylidene)thiazolidine-2,4-dione (**10b**)

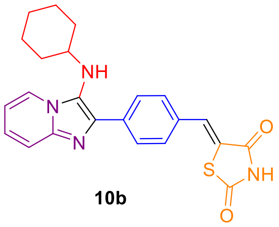

10b

10b was obtained from **8b** product (0.50 mmol; 0.160 g), thiazolidine-2,4-dione (1.00 mmol; 0.117 g), EDA (0.05 mmol; 0.003 mL) in AcOH (2.5 mL), in 66% yield (0.138 g) as a yellow solid (m.p.: 252-254 ºC; *unpublished compound*).

FTIR (ATR): ν 3230, 2926, 2849, 2741, 1744, 1698, 1599, 1514, 1288, 1152, 1014, 903, 833, 763, 687 cm^{-1}.

^{1}H NMR (600 MHz, DMSO-d_6): δ 8.89 (d, *J* = 6.8 Hz, 1H), 8.45 – 8.40 (m, 2H), 8.43 (d, *J* = 8.5 Hz, 2H), 8.05 – 7.97 (m, 2H), 7.96 (s, 1H), 7.90 (d, *J* = 8.5 Hz, 2H), 7.58 (td, *J* = 6.8, 1.3 Hz, 1H), 5.68 (s, 1H), 3.04 – 2.97 (m, 1H), 1.93 – 1.88 (m, 2H), 1.78 – 1.72 (m, 2H), 1.62 – 1.58 (m, 1H), 1.45 – 1.36 (m, 2H), 1.26 – 1.16 (m, 3H) ppm.

^{13}C NMR (151 MHz, DMSO-d_6): δ 167.7, 167.3, 137.3, 133.5, 132.6, 130.7, 130.5, 129.3, 127.7, 127.4, 125.4, 124.5, 116.3, 112.4, 56.5, 33.3, 25.2, 24.5 ppm.

HRMS (ESI-QTOF) *m/z* calculated for $C_{23}H_{23}N_4O_2S^+$: 419.1536 $[M+H]^+$; found 419.1549.

(*Z*)-5-(4-(3-(Phenylamino)imidazo[1,2-*a*]pyridin-2-yl)benzylidene)thiazolidine-2,4-dione (**10c**)

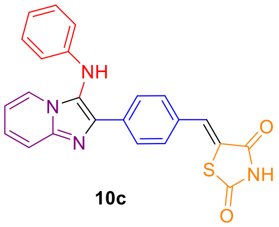

10c

10c was obtained from **8c** product (0.50 mmol; 0.157 g), thiazolidine-2,4-dione (1.00 mmol; 0.117 g), EDA (0.05 mmol; 0.003 mL) in AcOH (2.5 mL), in 86% yield (0.178 g) as a dark-yellow solid (m.p.: 200 ºC (dec.); *unpublished compound*).

FTIR (ATR): ν 3104, 2924, 2744, 1735, 1708, 1654, 1598, 1497, 1414, 1323, 1289, 1248, 1152, 1016, 902, 832, 746, 690 cm^{-1}.

^{1}H NMR (600 MHz, DMSO-d_6): δ 8.67 (s, 1H), 8.24 (d, *J* = 6.8 Hz, 1H), 8.17 (d, *J* = 8.1 Hz, 2H), 7.91 (d, *J* = 9.0 Hz, 1H), 7.80 (s, 1H), 7.75 (t, *J* = 8.1 Hz, 1H), 7.71 (d, *J* = 8.1 Hz, 2H), 7.28 (t, *J* = 6.8 Hz, 1H), 7.19 (t, *J* = 8.1 Hz, 2H), 6.81 (t, *J* = 6.8 Hz, 1H), 6.68 (d, *J* = 8.1 Hz, 2H) ppm.

^{13}C NMR (151 MHz, DMSO-d_6): δ 167.8, 167.3, 144.4, 139.7, 133.4, 130.9, 130.5, 129.7, 127.3, 124.4, 124.3, 120.9, 119.5, 115.4, 114.6, 113.5 ppm.

HRMS (ESI-QTOF) *m/z* calculated for $C_{23}H_{17}N_4O_2S^+$: 413.1067 $[M+H]^+$; found 413.1067.

(*Z*)-5-(4-(3-(*tert*-Butylamino)-7-chloroimidazo[1,2-*a*]pyridin-2-yl)benzylidene)thiazolidine-2,4-dione (**10d**)

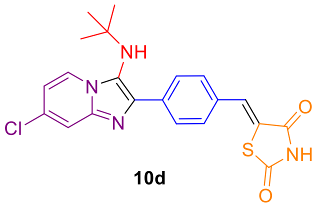

10d

10d was obtained from **8d** product (0.50 mmol; 0.164 g), thiazolidine-2,4-dione (1.00 mmol; 0.117 g), EDA (0.05 mmol; 0.003 mL) in AcOH (2.5 mL), in 98% yield (0.210 g) as a yellow solid (m.p.: >300 ºC; *unpublished compound*).

FTIR (ATR): ν 3367, 3271, 3087, 3031, 2965, 1744, 1703, 1651, 1595, 1519, 1424, 1281, 1196, 1142, 1073, 1004, 903, 807, 680 cm^{-1}.

^{1}H NMR (600 MHz, DMSO-*d*$_6$): δ 8.82 (d, *J* = 7.3 Hz, 1H), 8.28 – 8.24 (m, 2H), 8.00 (d, *J* = 2.0 Hz, 1H), 7.84 (s, 1H), 7.77 – 7.73 (m, 2H), 7.49 (dd, *J* = 7.3, 2.0 Hz, 1H), 1.02 (s, 9H) ppm.

^{13}C NMR (151 MHz, DMSO-*d*$_6$): δ 167.7, 167.3, 138.0, 137.2, 133.8, 130.8, 130.3, 129.7, 128.7, 127.1, 126.3, 124.6, 117.0, 111.9, 56.7, 29.7 ppm.

HRMS (ESI-QTOF) *m/z* calculated for $C_{21}H_{20}ClN_4O_2S^+$: 427.0990 [M+H]$^+$; found 427.0992.

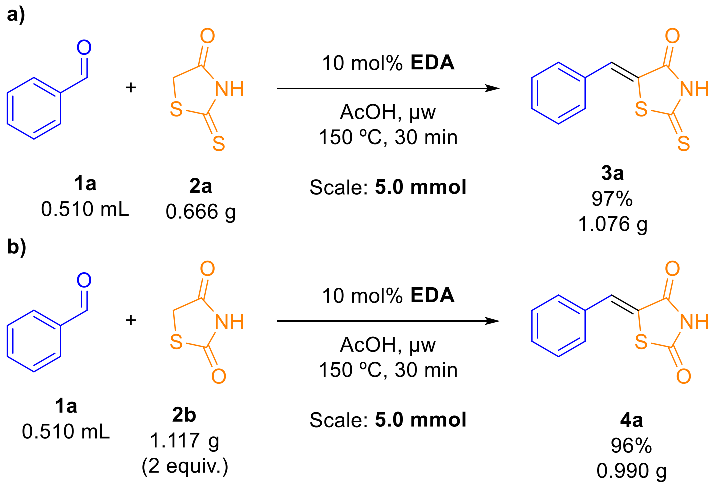

Scheme S1. 10-Fold scale-up of the EDA-catalyzed Knoevenagel condensation reactions under microwave (μw) heating.

Table S1: Optimization of reaction conditions for the synthesis of (*Z*)-5-benzylidenethiazolidine-2,4-dione **4a** under microwave heating[a].

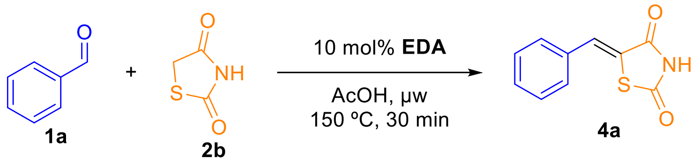

Entry	**2b** (equiv)	Yield (%)[b]
1	1.0	74
2	1.2	75
3	1.5	79
4	1.8	92
**5**	**2.0**	**99**

[a]Reaction conditions: benzaldehyde (0.50 mmol), thiazolidine-2,4-dione (0.50 mmol) and EDA (0.05 mmol, 10 mol %) in AcOH (2.5 mL). [b]Isolated yields.

2. References

1. Baek, S.-J.; Park, A.; Ahn, Y.-J.; Choo, J. *Analyst* **2015**, *140*, 250–257. doi:10.1039/C4AN01061B
2. Lagorio, M. g. *Methods Appl. Fluoresc.* **2020**, *8*, 043001. doi:10.1088/2050-6120/aba69c
3. Rhys Williams, A. T.; Winfield, S. A.; Miller, J. N. *Analyst* **1983**, *108*, 1067–1071. doi:10.1039/an9830801067
4. Brouwer, A. M. *Pure Appl. Chem.* **2011**, *83*, 2213–2228. doi:10.1351/PAC-REP-10-09-31
5. Sheldrick, g. M. *Acta Crystallogr. C Struct. Chem.* **2015**, *71*, 3–8. doi:10.1107/S2053229614024218
6. Dolomanov, O. V.; Bourhis, L. J.; gildea, R. J.; Howard, J. A. K.; Puschmann, H. *J. Appl. Crystallogr.* **2009**, *42*, 339–341. doi:10.1107/S0021889808042726
7. Macrae, C. F.; Sovago, I.; Cottrell, S. J.; galek, P. T. A.; McCabe, P.; Pidcock, E.; Platings, M.; Shields, g. P.; Stevens, J. S.; Towler, M.; Wood, P. A. *J. Appl. Crystallogr.* **2020**, *53*, 226–235. doi:10.1107/S1600576719014092
8. Pettersen, E. F.; goddard, T. D.; Huang, C. C.; Couch, g. S.; greenblatt, D. M.; Meng, E. C.; Ferrin, T. E. *J. Comput. Chem.* **2004**, *25*, 1605–1612. doi:10.1002/jcc.20084
9. Neese, F. *Wiley Interdiscip. Rev. Comput. Mol. Sci.* **2012**, *2*, 73–78. doi:10.1002/wcms.81
10. Neese, F. *Wiley Interdiscip. Rev. Comput. Mol. Sci.* **2018**, *8*, e1327. doi:10.1002/wcms.1327
11. Neese, F.; Wennmohs, F.; Becker, U.; Riplinger, C. *J. Chem. Phys.* **2020**, *152*, 224108. doi:10.1063/5.0004608
12. Neese, F. *Wiley Interdiscip. Rev. Comput. Mol. Sci.* **2022**, *12*, e1606. doi:10.1002/wcms.1606
13. Zhao, Y.; Truhlar, D. g. *Theor. Chem. Acc.* **2008**, *120*, 215–241. doi:10.1007/s00214-007-0310-x
14. Weigend, F.; Ahlrichs, R. *Phys. Chem. Chem. Phys.* **2005**, *7*, 3297. doi:10.1039/b508541a
15. Grimme, S. *J. Comput. Chem.* **2006**, *27*, 1787–1799. doi:10.1002/jcc.20495
16. Wales, D. J.; Doye, J. P. K. *J. Phys. Chem. A* **1997**, *101*, 5111–5116. doi:10.1021/jp970984n
17. Goedecker, S. *J. Chem. Phys.* **2004**, *120*, 9911–9917. doi:10.1063/1.1724816
18. Bannwarth, C.; Ehlert, S.; grimme, S. *J. Chem. Theory Comput.* **2019**, *15*, 1652–1671. doi:10.1021/acs.jctc.8b01176
19. Ditchfield, R. *J. Chem. Phys.* **1972**, *56*, 5688–5691. doi:10.1063/1.1677088
20. Ditchfield, R. *Mol. Phys.* **1974**, *27*, 789–807. doi:10.1080/00268977400100711
21. Hansen, A. E.; Bouman, T. D. *J. Chem. Phys.* **1985**, *82*, 5035–5047. doi:10.1063/1.448625
22. Barone, V.; Cossi, M. *J. Phys. Chem. A* **1998**, *102*, 1995–2001. doi:10.1021/jp9716997
23. Martinho, L. A.; Andrade, C. K. Z. *Beilstein J. Org. Chem.* **2024**, *20*, 628–637. doi:10.3762/bjoc.20.55
24. Gadekar, S. P.; Dipake, S. S.; gaikwad, S. T.; Lande, M. K. *Res. Chem. Intermed.* **2018**, *44*, 7509–7518. doi:10.1007/s11164-018-3570-2
25. Brown, F. C.; Bradsher, C. K.; Bond, S. M. *Ind. Eng. Chem.* **1953**, *45*, 1030–1033. doi:10.1021/ie50521a047
26. Zhou, J.; Song, Y.; Zhu, F.; Zhu, Y. *Synth. Commun.* **2006**, *36*, 3297–3303. doi:10.1080/00397910600941166
27. Opletalova, V.; Dolezel, J.; Kralova, K.; Pesko, M.; Kunes, J.; Jampilek, J. *Molecules* **2011**, *16*, 5207–5227. doi:10.3390/molecules16065207
28. Jadav, S. S.; Sinha, B. N.; Hilgenfeld, R.; Pastorino, B.; de Lamballerie, X.; Jayaprakash, V. *Eur. J. Med. Chem.* **2015**, *89*, 172–178. doi:10.1016/j.ejmech.2014.10.042
29. Mackie, A.; Misra, A. L. *J. Chem. Soc.* **1954**, 3919–3922. doi:10.1039/jr9540003919

30. Pinson, J.; Schmidt-Kittler, O.; Zhu, J.; Jennings, I. g.; Kinzler, K. W.; Vogelstein, B.; Chalmers, D. K.; Thompson, P. E. *ChemMedChem* **2011**, *6*, 514–522. doi:10.1002/cmdc.201000467
31. Le, Z. g.; Ni, K.; guo, L. T.; Xie, Z. B. *Adv. Mat. Res.* **2013**, *830*, 111–114. doi:10.4028/www.scientific.net/AMR.830.111
32. Veisi, H.; Vafajoo, Z.; Maleki, B.; Maghsoodlou, M. T. *Phosphorus Sulfur Silicon Relat. Elem.* **2013**, *188*, 672–677. doi:10.1080/10426507.2012.717134
33. Wang, H.; Zeng, J. *J. Chem. Res.* **2009**, *2009*, 374–376. doi:10.3184/030823409X460696
34. Saxena, g. C. A. N. A. K. S. S. D. S. *Orient. J. Chem.* **2008**, *24*, 347–351
35. Campaigne, E.; Kreighbaum, W. *J. Org. Chem.* **1961**, *26*, 1326–1327. doi:10.1021/jo01063a628
36. Taniyama, H.; Yusa, T.; Tabuchi, T.; Uchida, H. *J. Pharm. Soc. Jpn.***1956**, *76*, 154–157. doi:10.1248/yakushi1947.76.2_154
37. Voeller, F. Substituted Pyruvic Acid Oximes. DE956947, 1957.
38. Elgemeie, g. E. H.; Riad, B. Y.; Nawwar, g. A.; Elgamal, S. *Arch. Pharm.* **1987**, *320*, 223–228. doi:10.1002/ardp.19873200307
39. Sun, H.-S.; Wang, J.-Q.; gu, D.-W.; guo, C.; Shen, L.-J. *Int. Res. J. Pure. Appl. Chem.* **2016**, *11*, 1–8. doi:10.9734/IRJPAC/2016/25957
40. Tryambake, Pravin. T. *Asian J. Chem.* **2017**, *29*, 2401–2405. doi:10.14233/ajchem.2017.20695
41. Das, P.; Das, P.; Mondal, S.; Ray, S. *ChemistrySelect* **2024**, *9*, e202303973. doi:10.1002/slct.202303973
42. Rani, R.; Kumar, g.; Paul, K.; Luxami, V. *J. Lumin.* **2016**, *180*, 292–300. doi:10.1016/j.jlumin.2016.08.041
43. Tilekar, K.; Upadhyay, N.; Schweipert, M.; Hess, J. D.; Macias, L. H.; Mrowka, P.; Meyer-Almes, F.-J.; Aguilera, R. J.; Iancu, C. V.; Choe, J.; Ramaa, C. S. *Eur. J. Pharm. Sci.* **2020**, *154*, 105512. doi:10.1016/j.ejps.2020.105512
44. Swathi, N. ; R. Y. ; S. C. V. S. ; S. K. *Int. J. Pharm. Pharm. Sci.* **2012**, *4*, 632–637
45. Purohit, S. S. ; A. A. S. J. *Int. J. Pharm. Pharm. Sci.* **2012**, *4*, 273–276
46. Veisi, H.; Naeimi, A.; Maleki, B.; Ashrafi, S. S.; Sedrpoushan, A. *Org. Prep. Proced. Int.* **2015**, *47*, 309–315. doi:10.1080/00304948.2015.1052321
47. Fazylov, S. D.; Nurkenov, O. A.; Amerkhanova, Sh. K.; Tolepbek, I. S. *Russ. J. gen. Chem.* **2013**, *83*, 1792–1793. doi:10.1134/S1070363213090284
48. Mohammadi, A.; Safarnejad, M. *Spectrochim. Acta A Mol. Biomol. Spectrosc.* **2014**, *126*, 105–111. doi:10.1016/j.saa.2014.02.010
49. Jawale, D. V.; Pratap, U. R.; Lingampalle, D. L.; Mane, R. A. *Chin. J. Chem.* **2011**, *29*, 942–946. doi:10.1002/cjoc.201190192
50. de Paiva, R.; da Silva, J.; Moreira, H.; Pinto, O.; Camargo, L.; Naves, P.; Camargo, A.; Ribeiro, L.; Ramos, L. *J. Braz. Chem. Soc.* **2018**, *30*, 164–172. doi:10.21577/0103-5053.20180167
51. Metwally, N. H.; Rateb, N. M.; Zohdi, H. F. g*reen Chem. Lett. Rev.* **2011**, *4*, 225–228. doi:10.1080/17518253.2010.544330
52. Lafayette, E. A.; de Almeida, S. M. V.; Cavalcanti Santos, R. V.; de Oliveira, J. F.; Amorim, C. A. da C.; da Silva, R. M. F.; Pitta, M. g. da R.; Pitta, I. da R.; de Moura, R. O.; de Carvalho Júnior, L. B.; de Melo Rêgo, M. J. B.; de Lima, M. do C. A. *Eur. J. Med. Chem.* **2017**, *136*, 511–522. doi:10.1016/j.ejmech.2017.05.012
53. Shahrisa, A.; Esmati, S. *Synlett* **2013**, *24*, 595–602. doi:10.1055/s-0032-1318221

3. FTIR, ^{1}H and ^{13}C NMR, HRMS and HRMS/MS spectra for all compounds

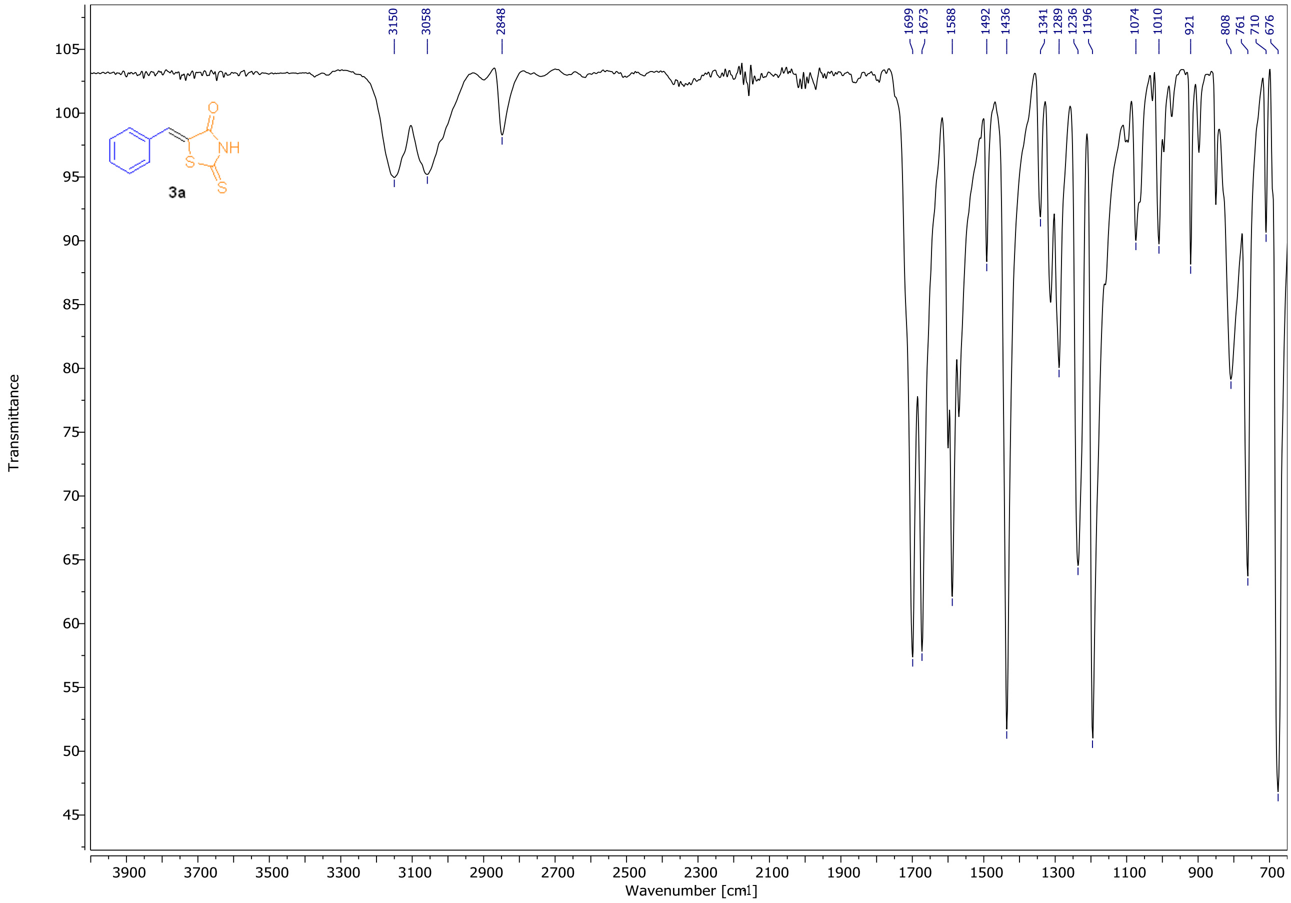

Figure S2. FTIR (ATR) of compound **3a**.

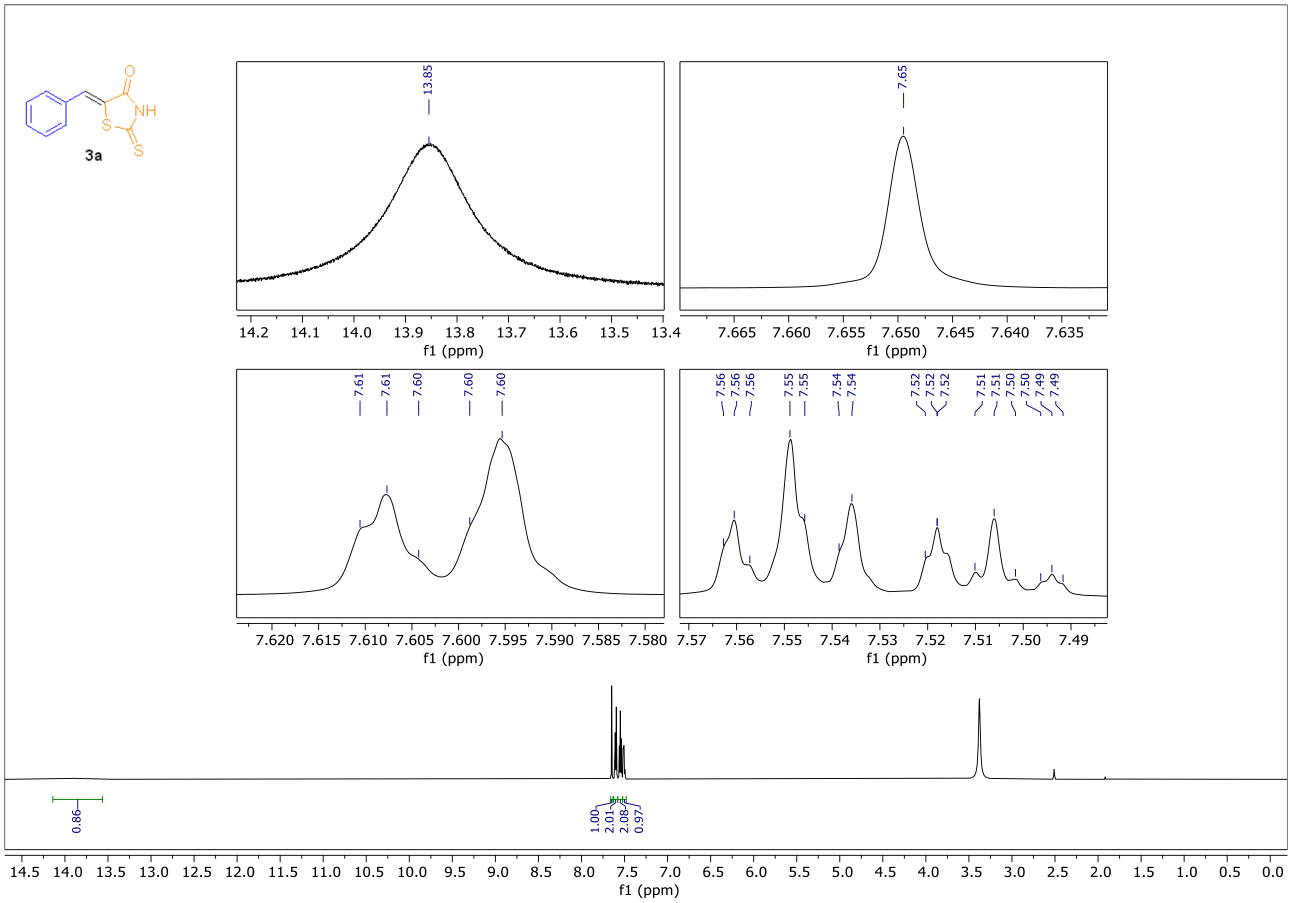

Figure S3. ^{1}H NMR spectrum (600 MHz, DMSO-d_6) of compound **3a**.

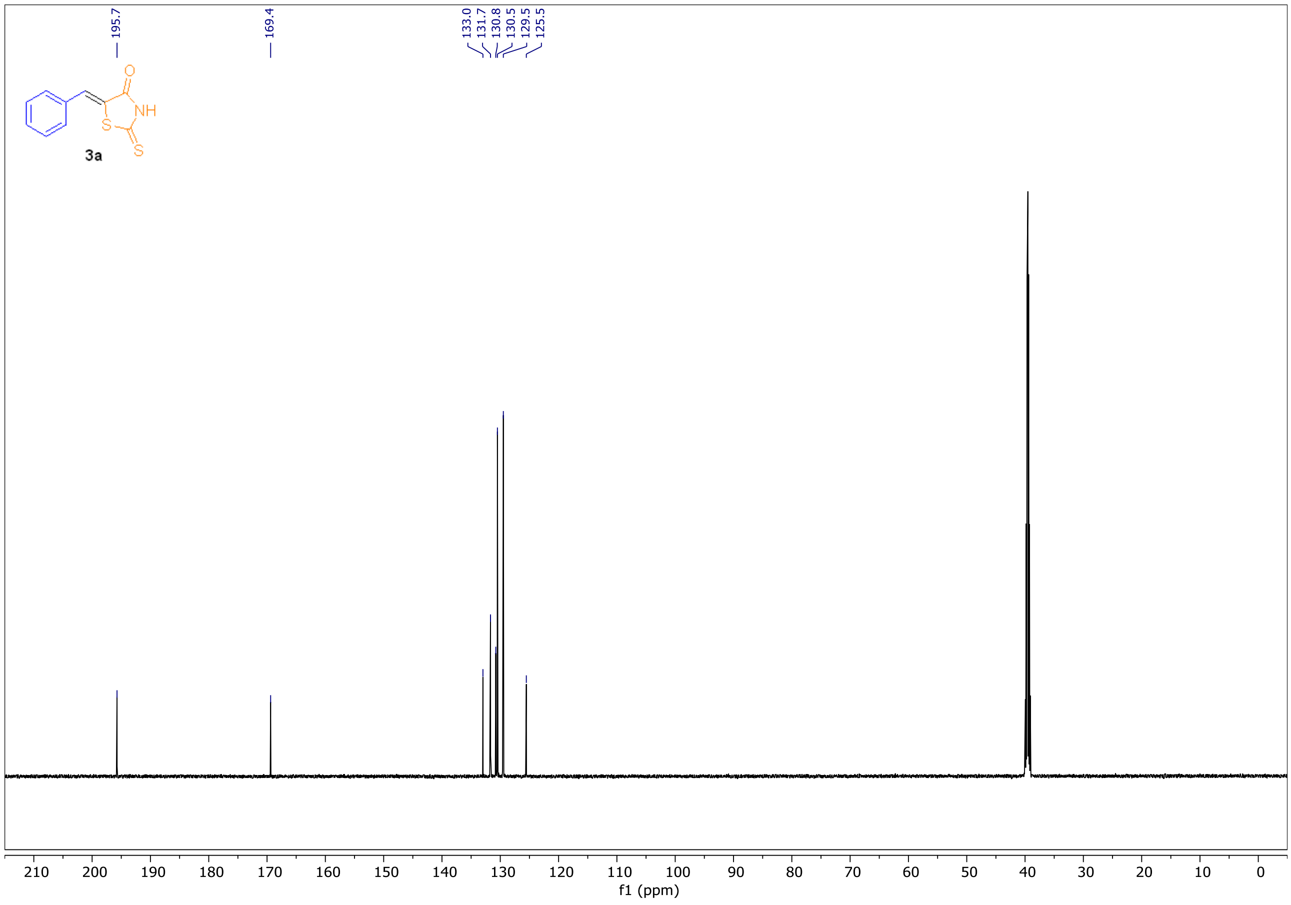

Figure S4. ^{13}C NMR spectrum (151 MHz, DMSO-d_6) of compound **3a**.

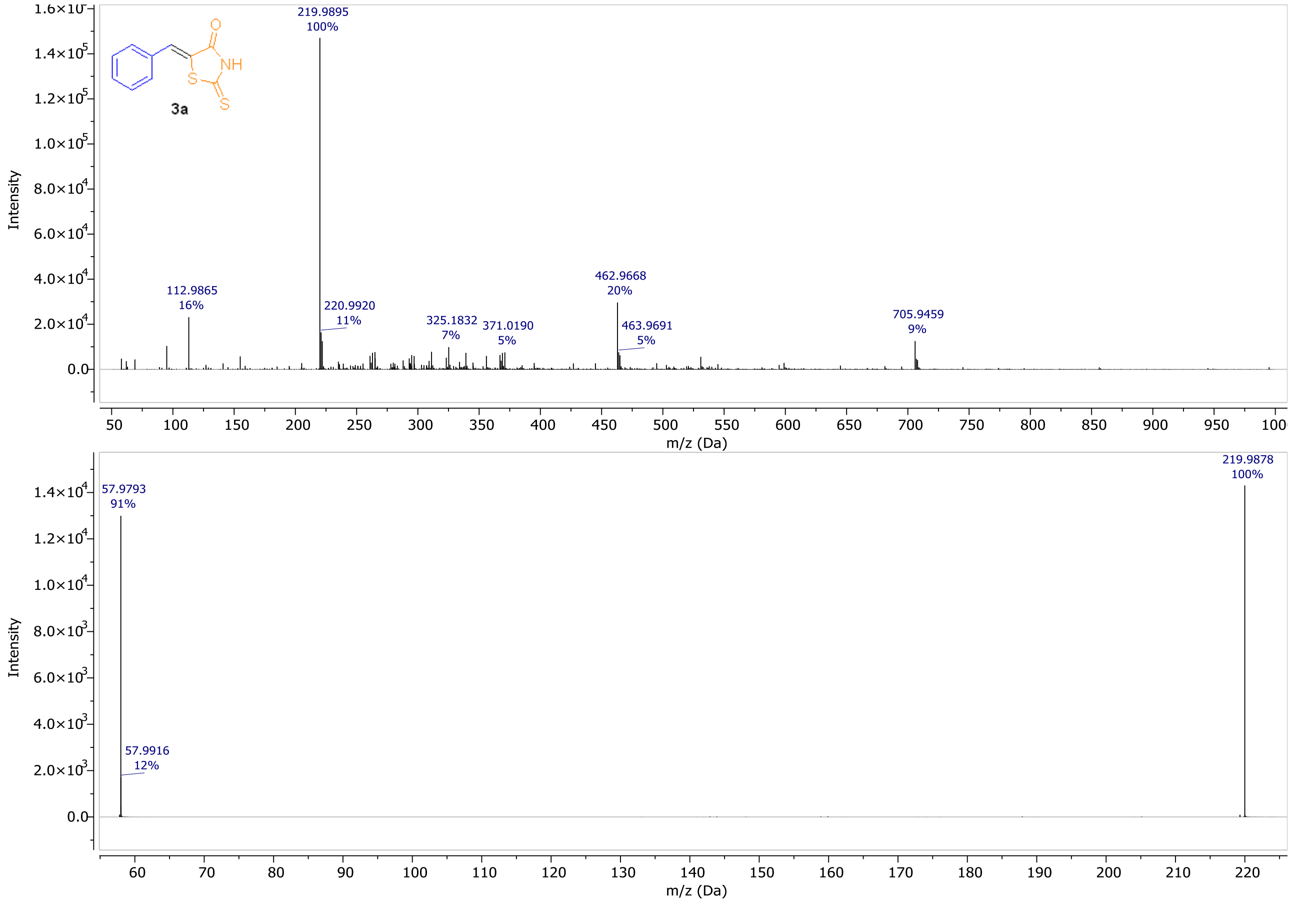

Figure S5. HRMS (ESI-QTOF) of compound **3a** and HRMS/MS for $[M-H]^-$.

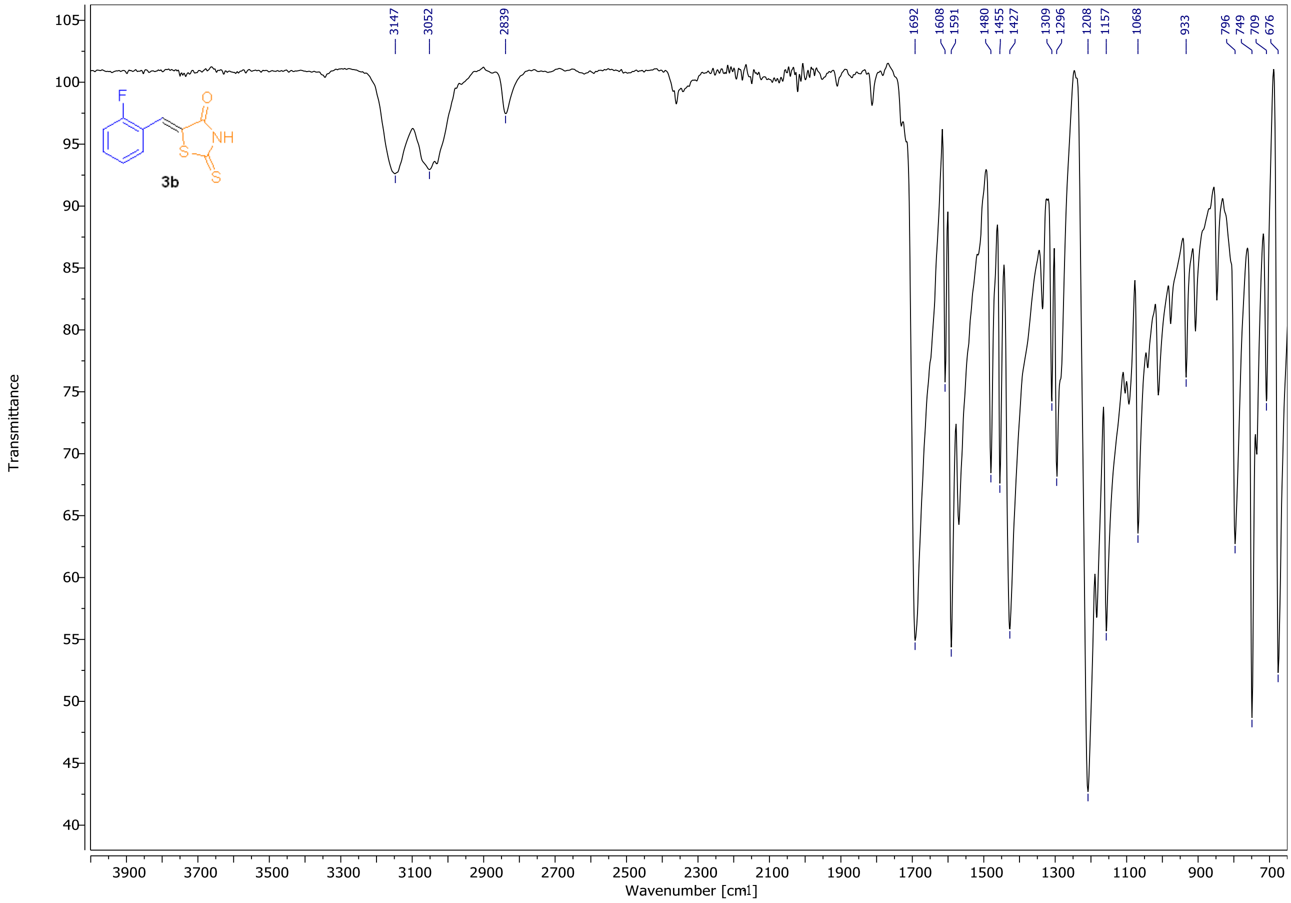

Figure S6. FTIR (ATR) of compound **3b**.

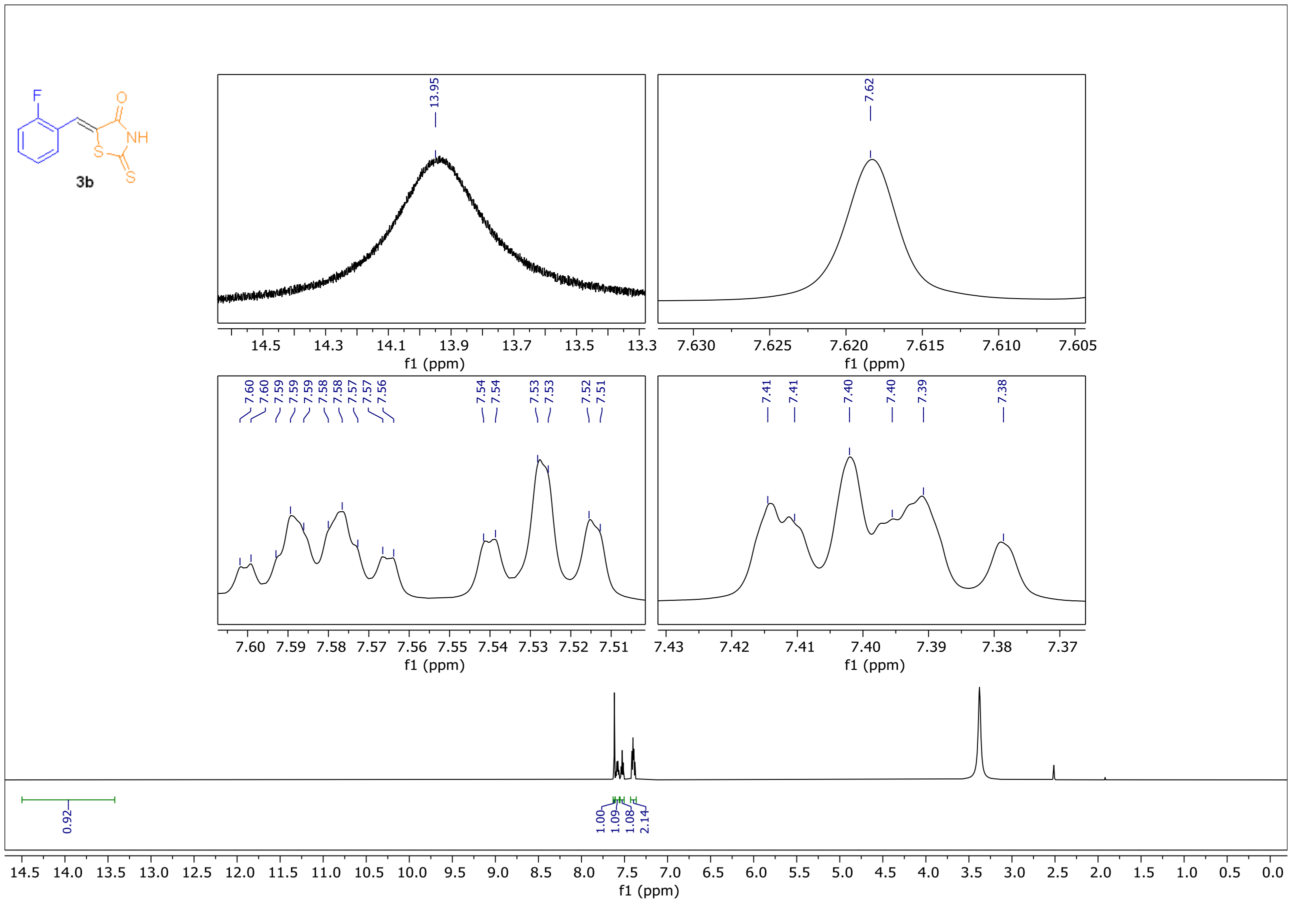

Figure S7. ^{1}H NMR spectrum (600 MHz, DMSO-*d*6) of compound **3b**.

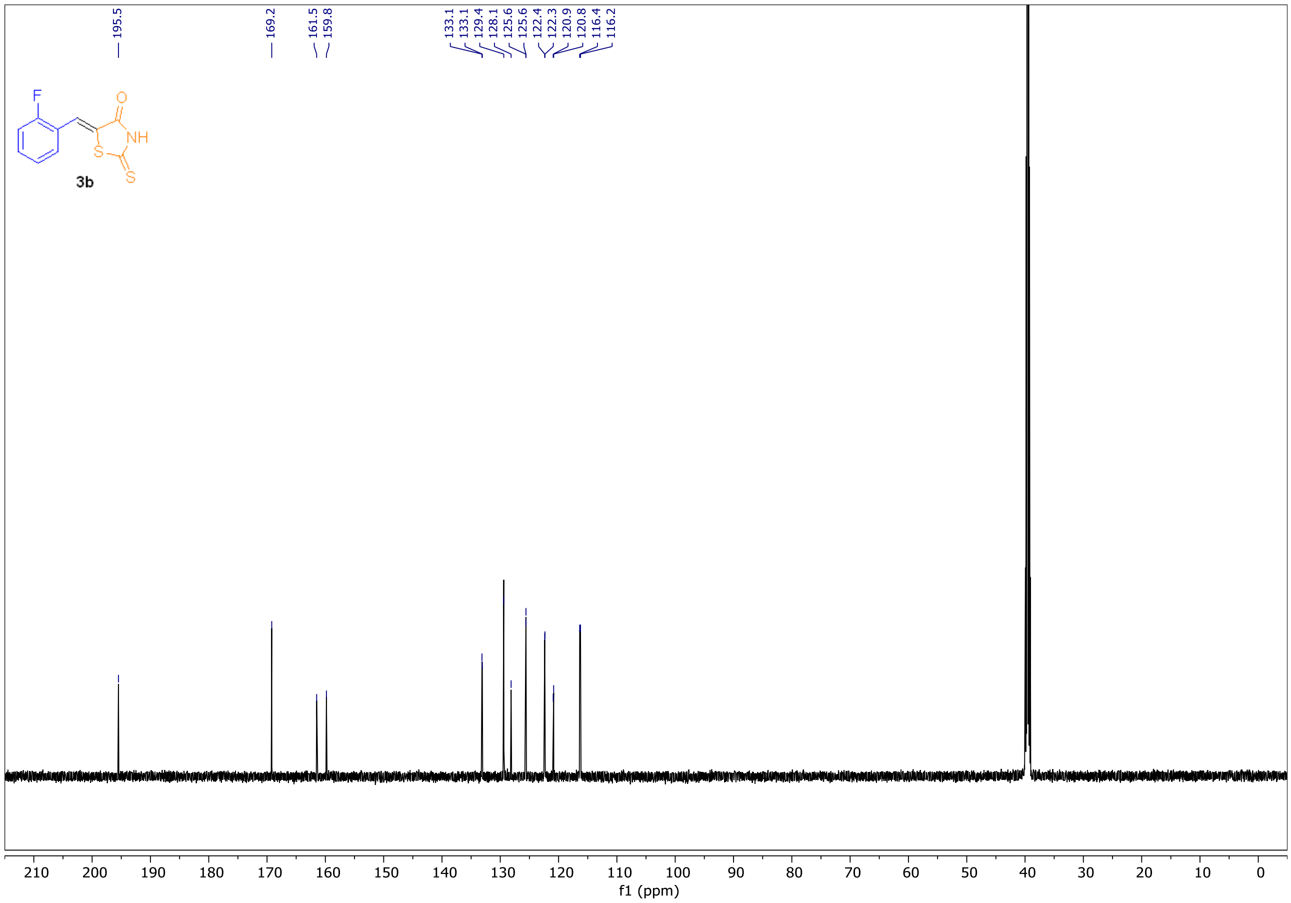

Figure S8. ^{13}C NMR spectrum (151 MHz, DMSO-d_6) of compound **3b**.

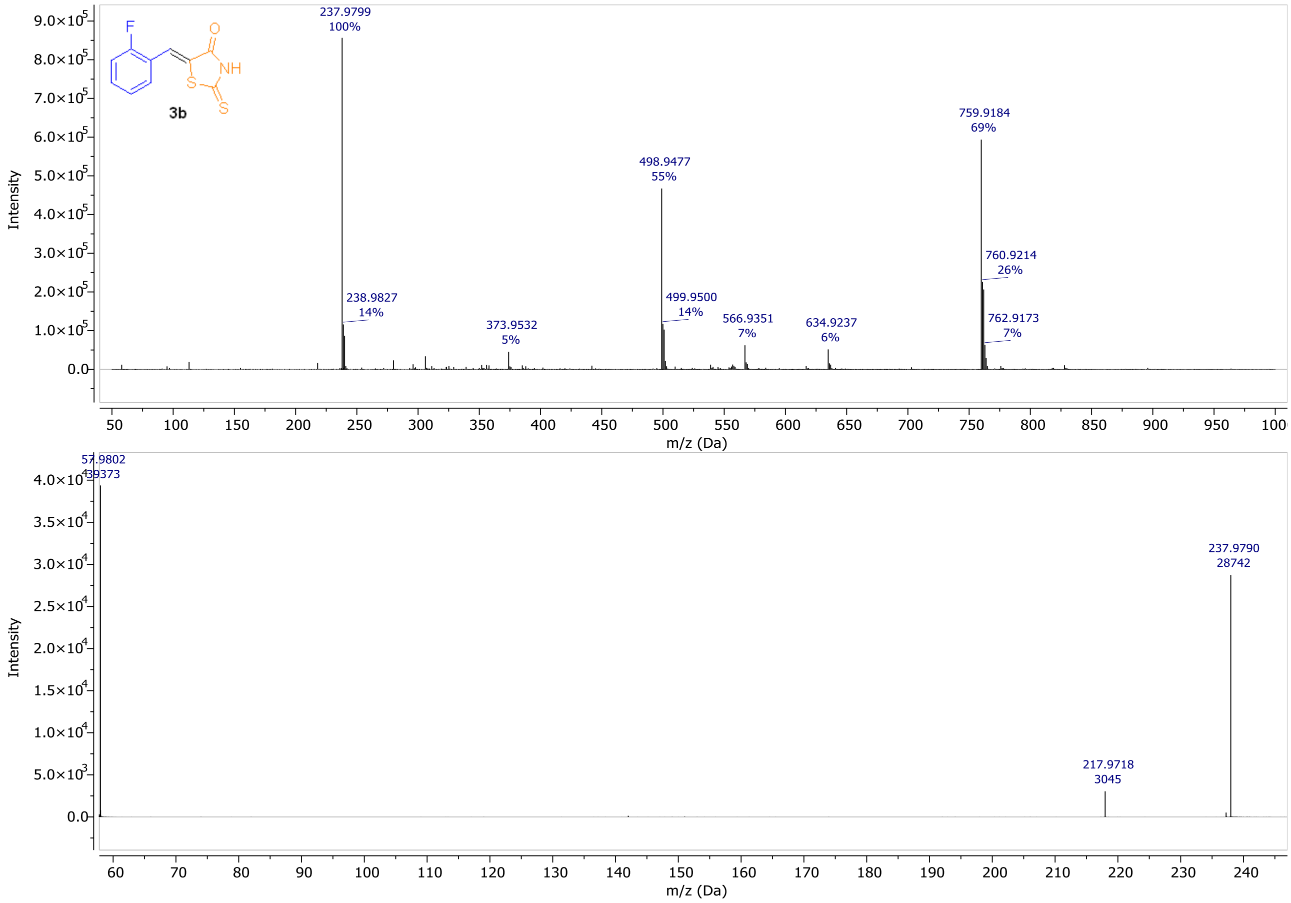

Figure S9. HRMS (ESI-QTOF) of compound **3b** and HRMS/MS for [M−H]⁻.

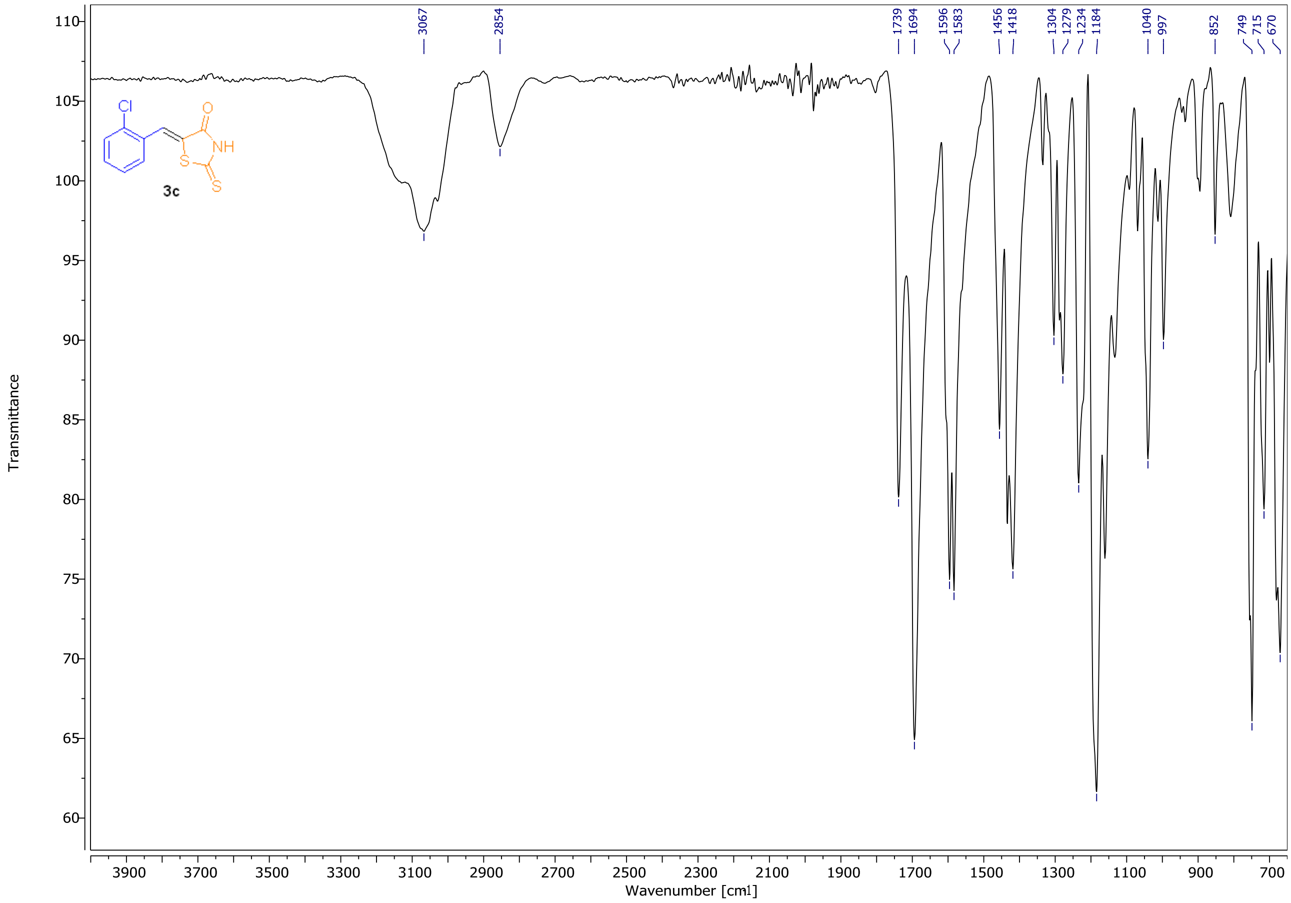

Figure S10. FTIR (ATR) of compound **3c**.

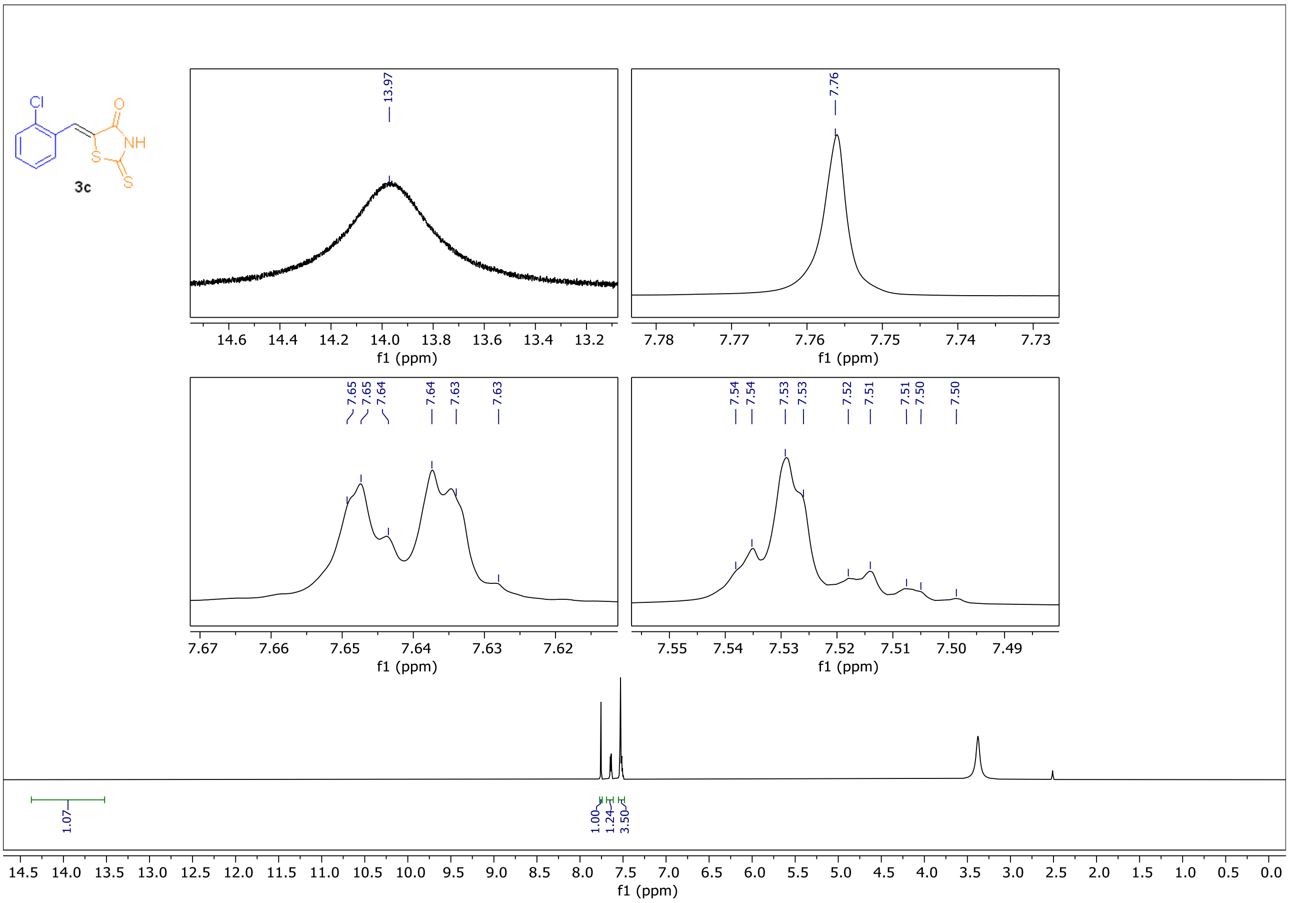

Figure S11. ^{1}H NMR spectrum (600 MHz, DMSO-d_6) of compound **3c**.

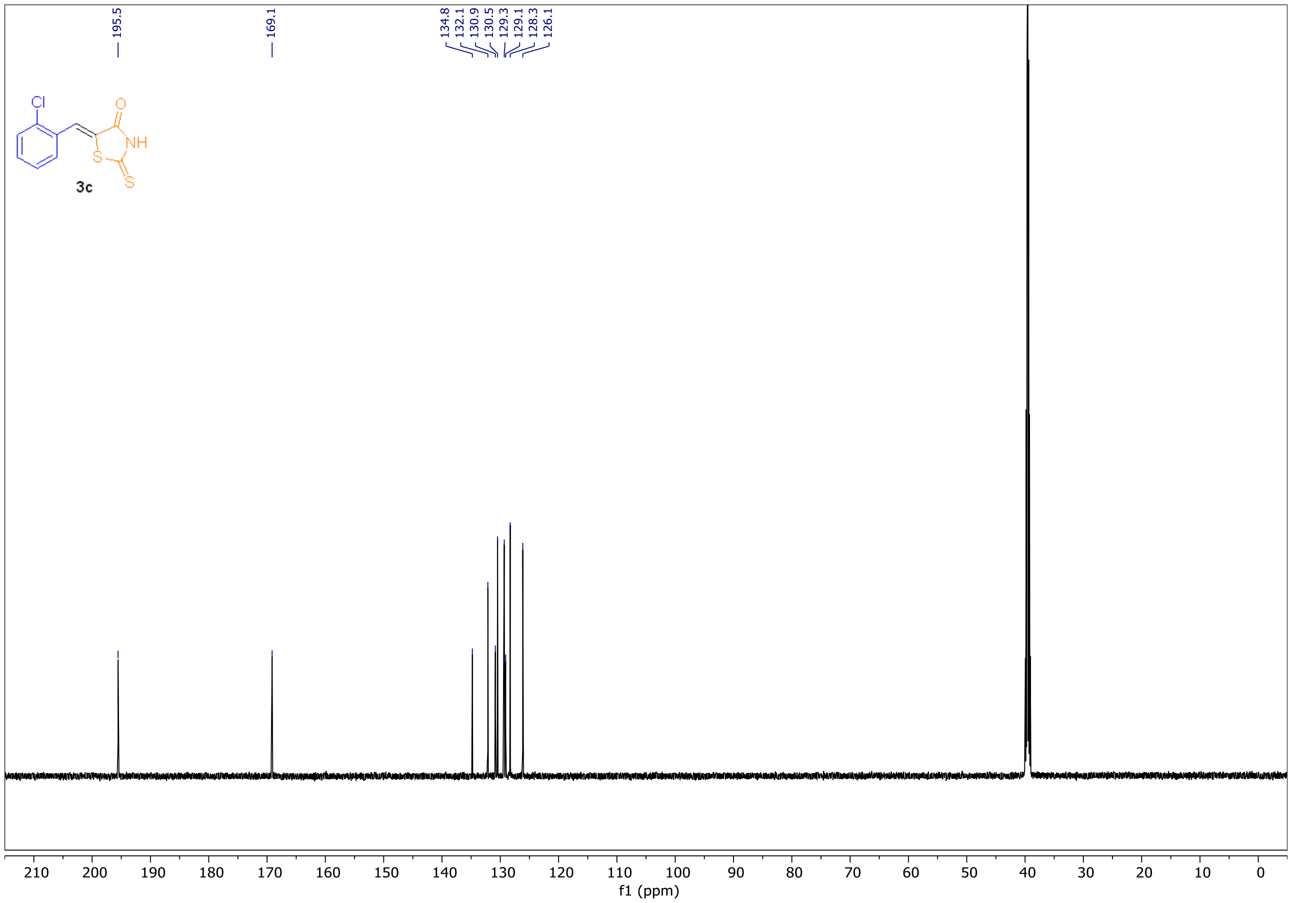

Figure S12. ^{13}C NMR spectrum (151 MHz, DMSO-d_6) of compound **3c**.

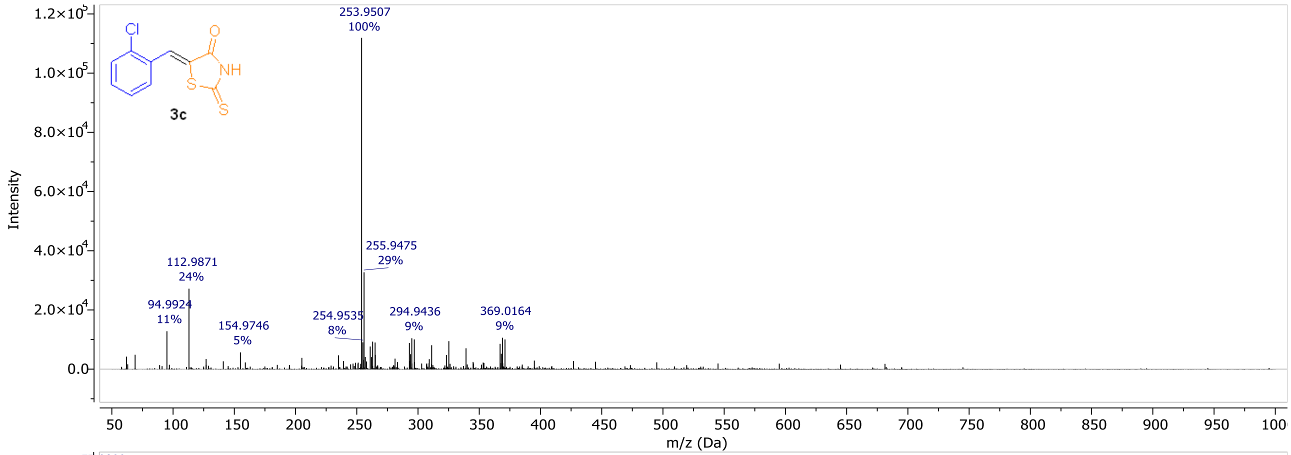

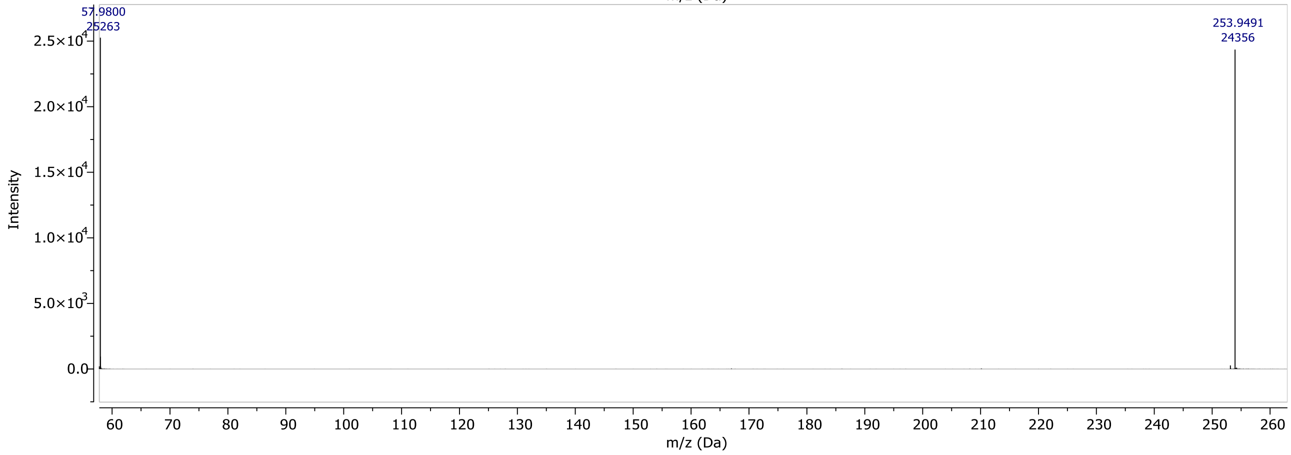

Figure S13. HRMS (ESI-QTOF) of compound **3c** and HRMS/MS for [M−H]⁻.

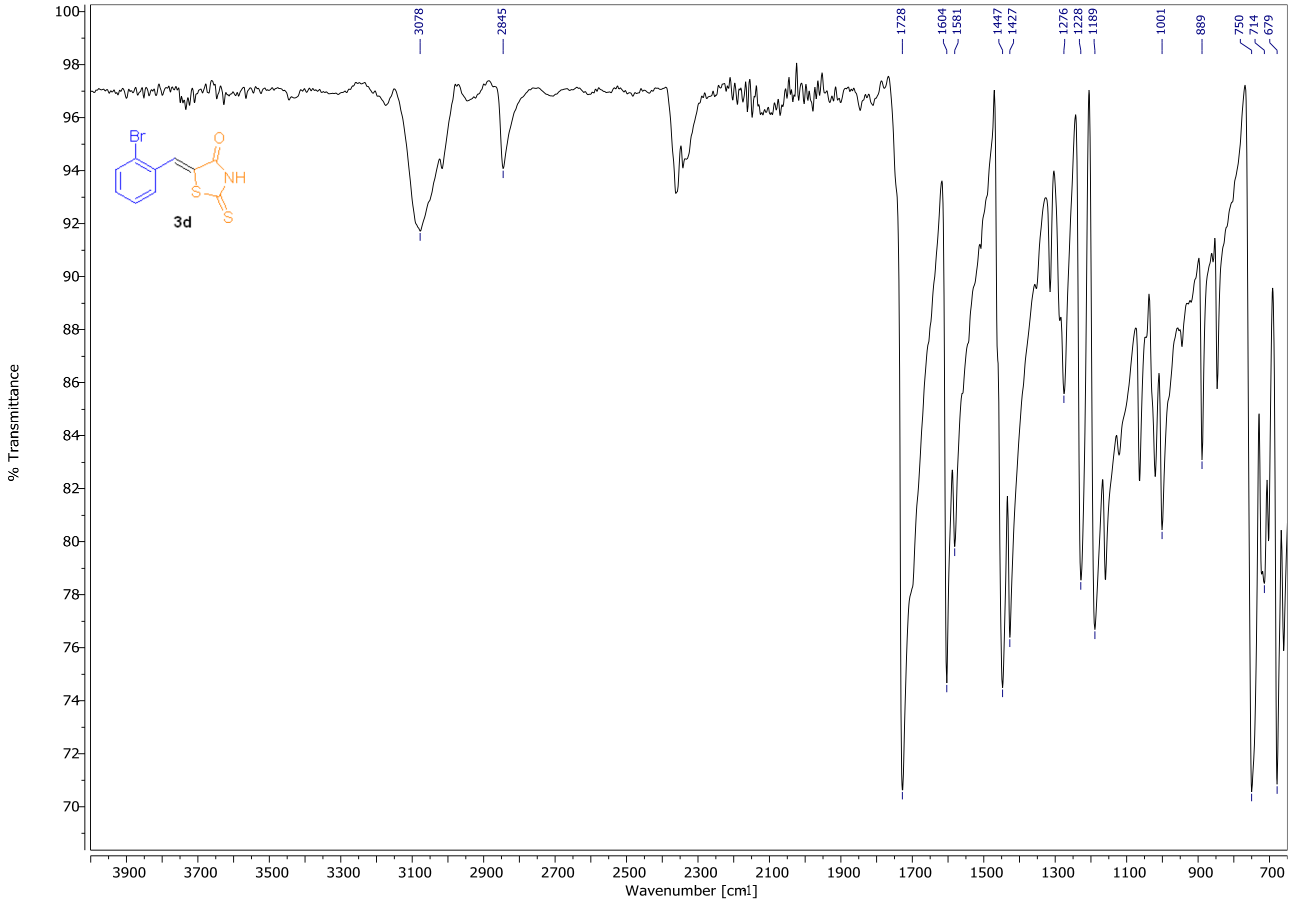

Figure S14. FTIR (ATR) of compound **3d**.

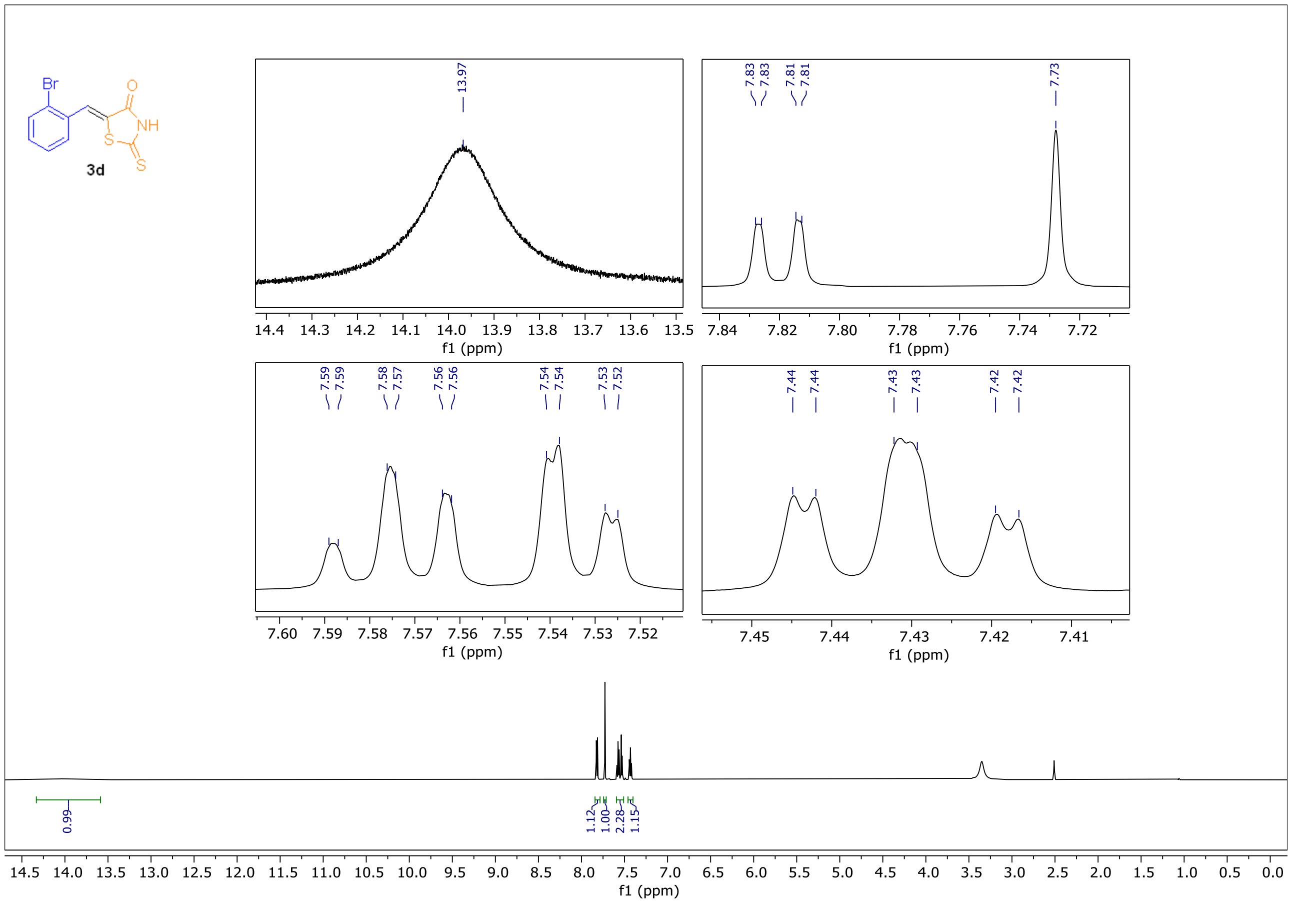

Figure S15. ^{1}H NMR spectrum (600 MHz, DMSO-d_6) of compound **3d**.

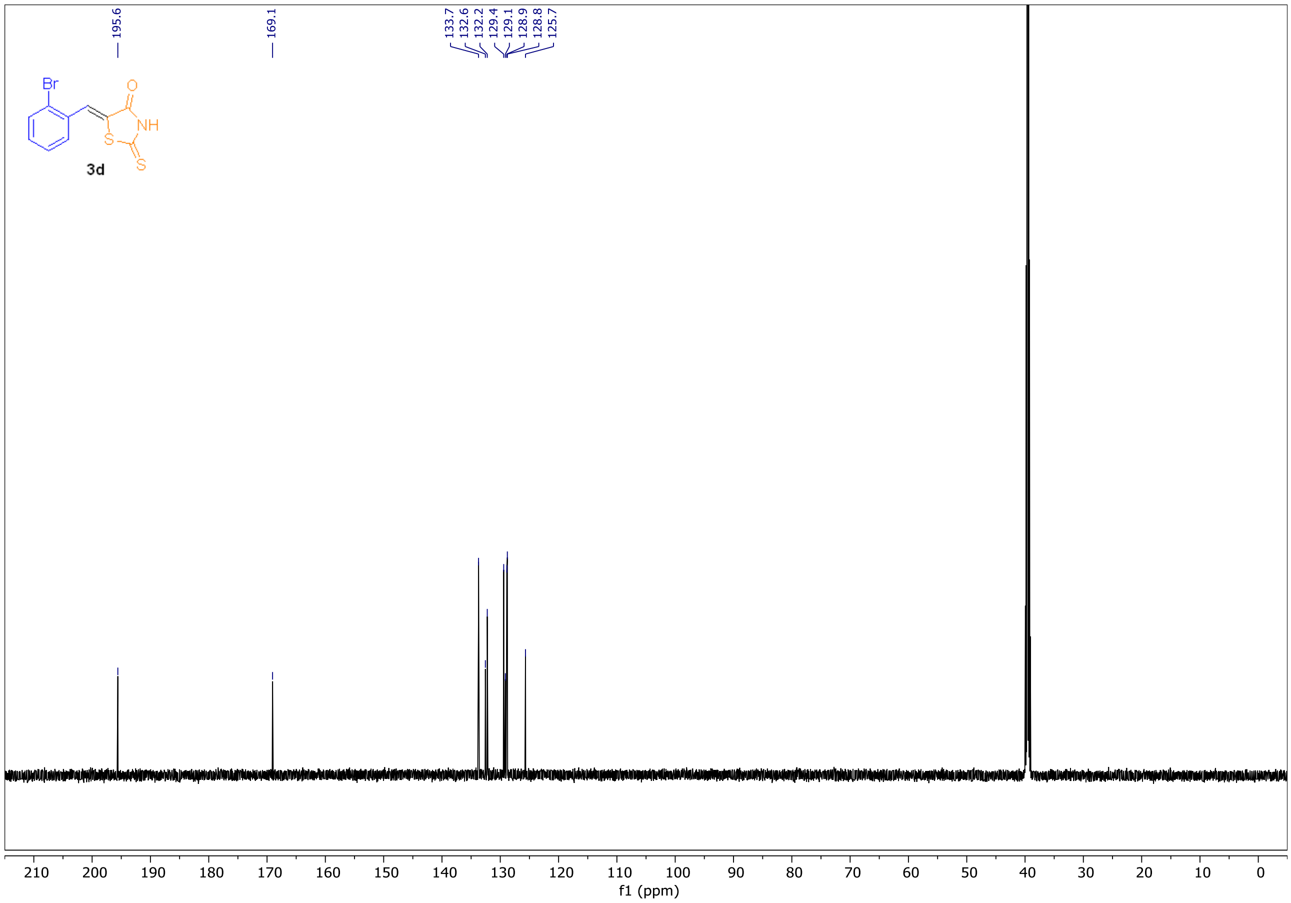

Figure S16. ^{13}C NMR spectrum (151 MHz, DMSO-d_6) of compound **3d**.

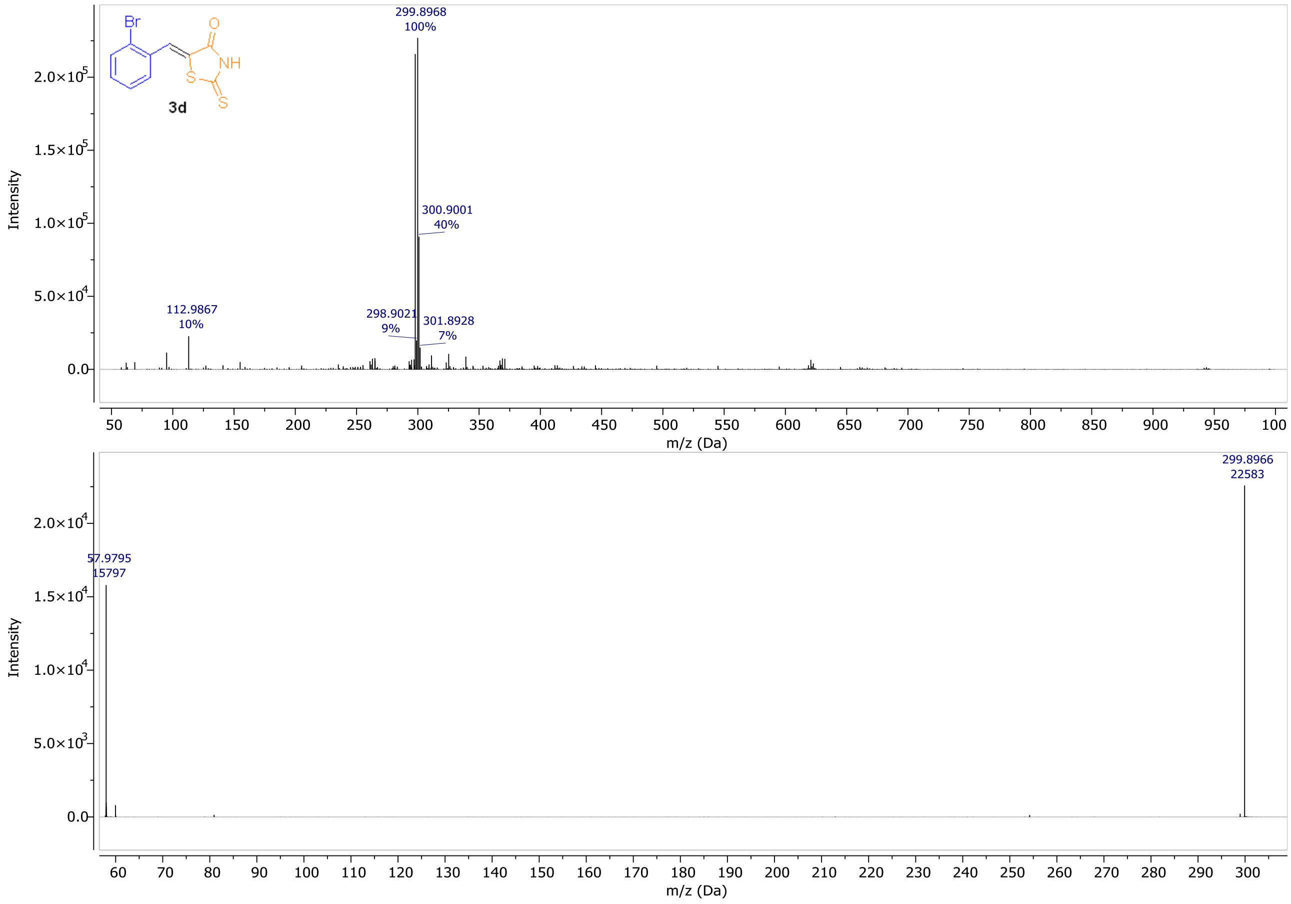

Figure S17. HRMS (ESI-QTOF) of compound **3d** and HRMS/MS for [M−H]⁻.

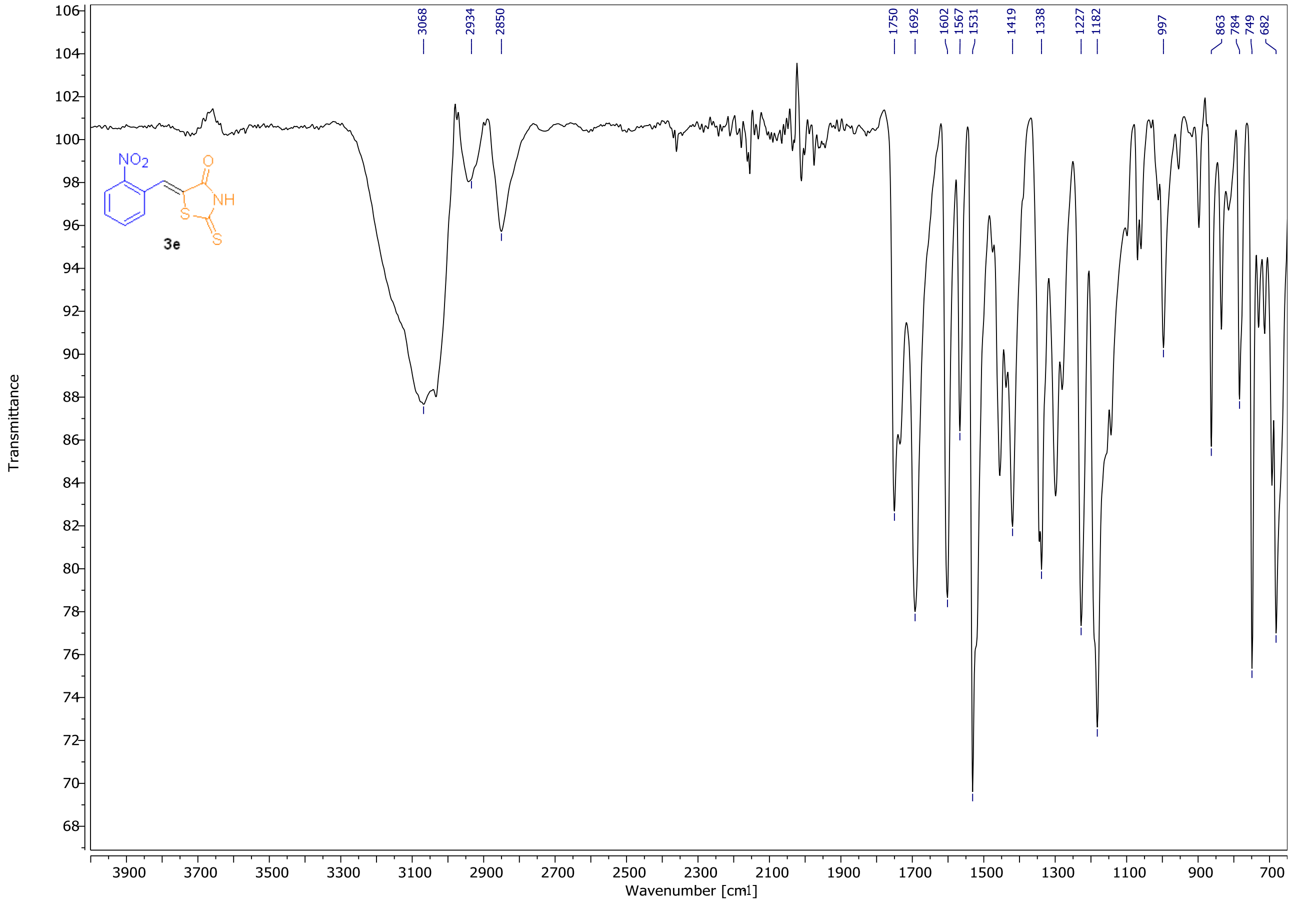

Figure S18. FTIR (ATR) of compound **3e**.

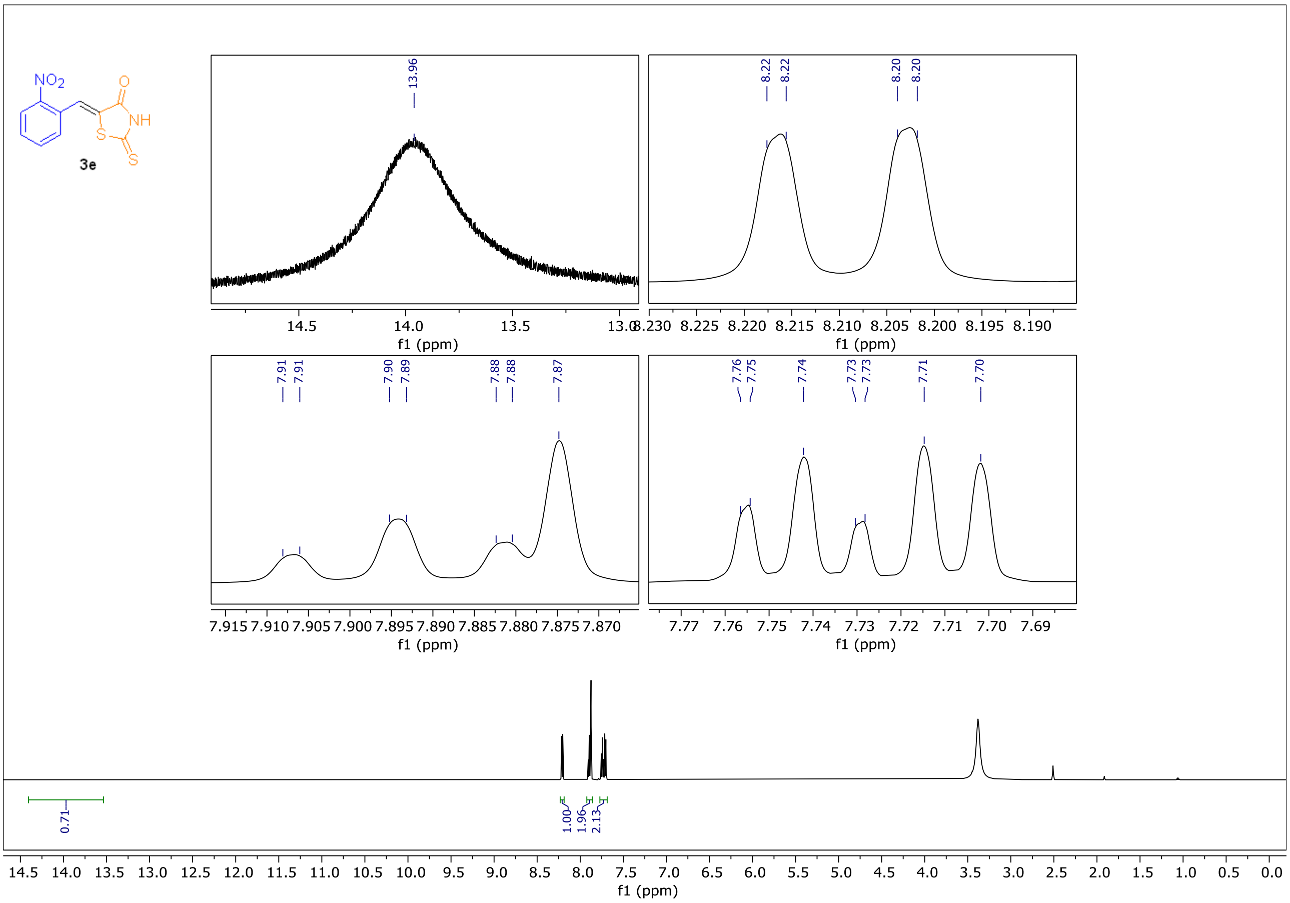

Figure S19. ^{1}H NMR spectrum (600 MHz, DMSO-d_6) of compound **3e**.

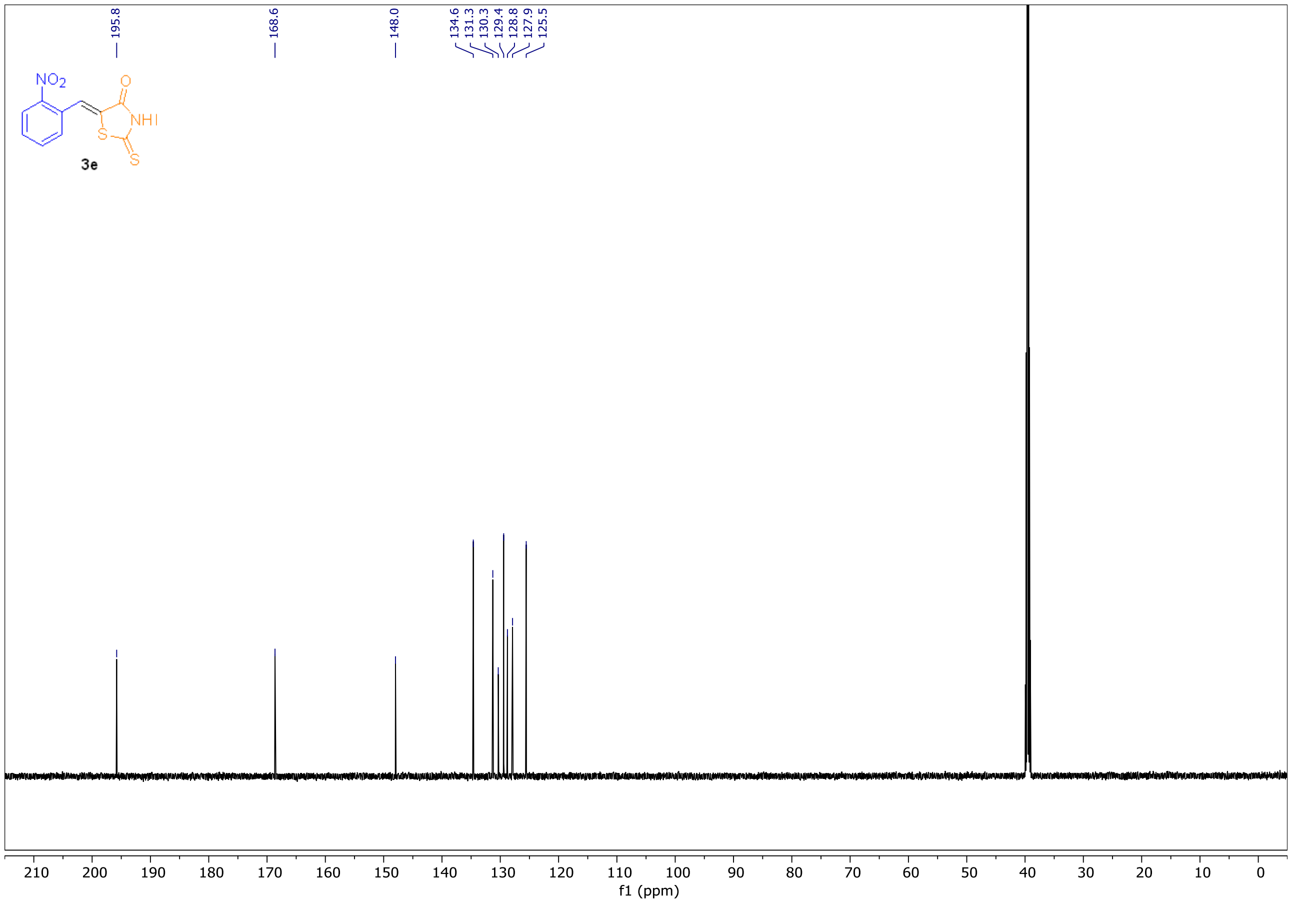

Figure S20. ^{13}C NMR spectrum (151 MHz, DMSO-d_6) of compound **3e**.

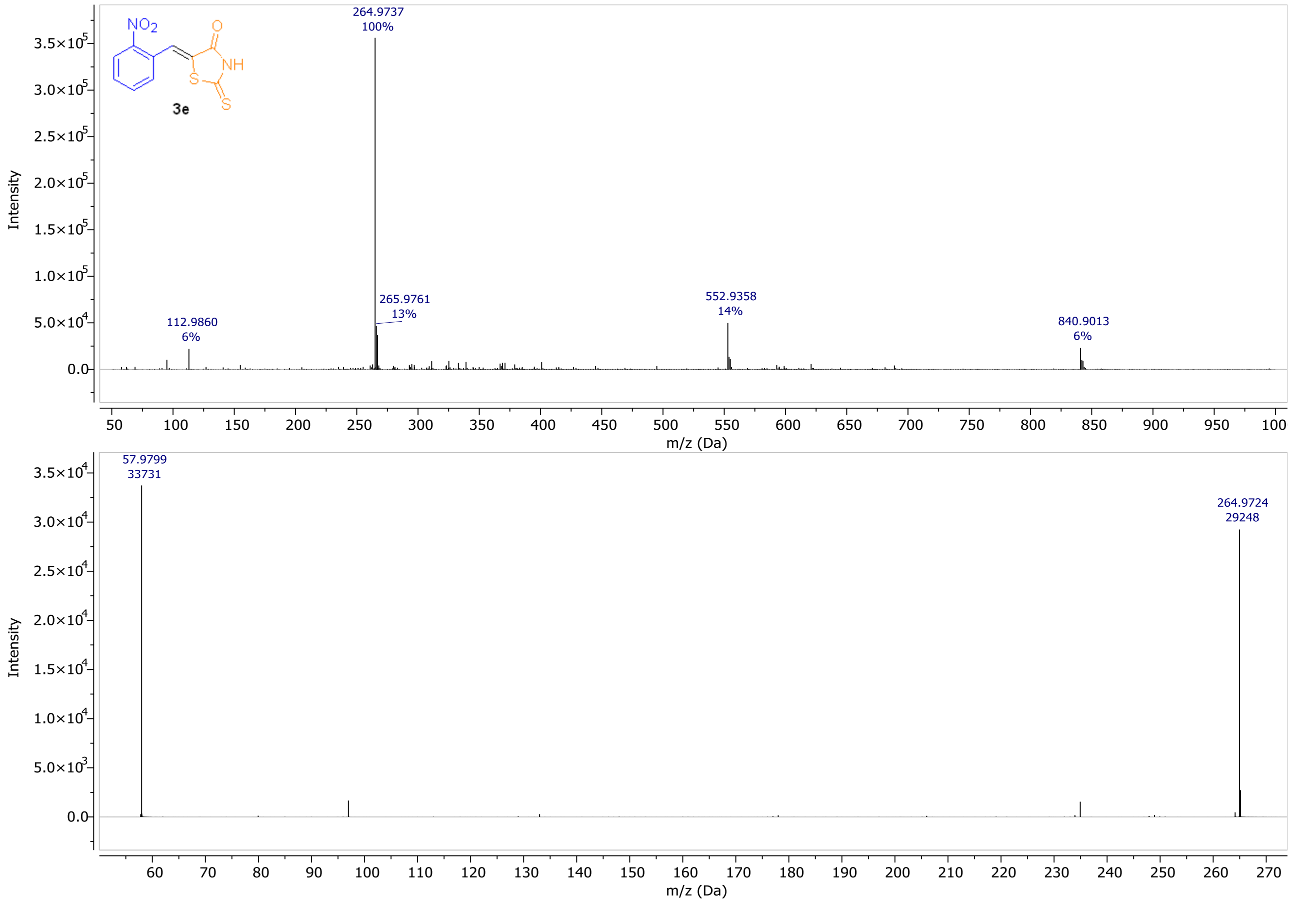

Figure S21. HRMS (ESI-QTOF) of compound **3e** and HRMS/MS for [M−H]⁻.

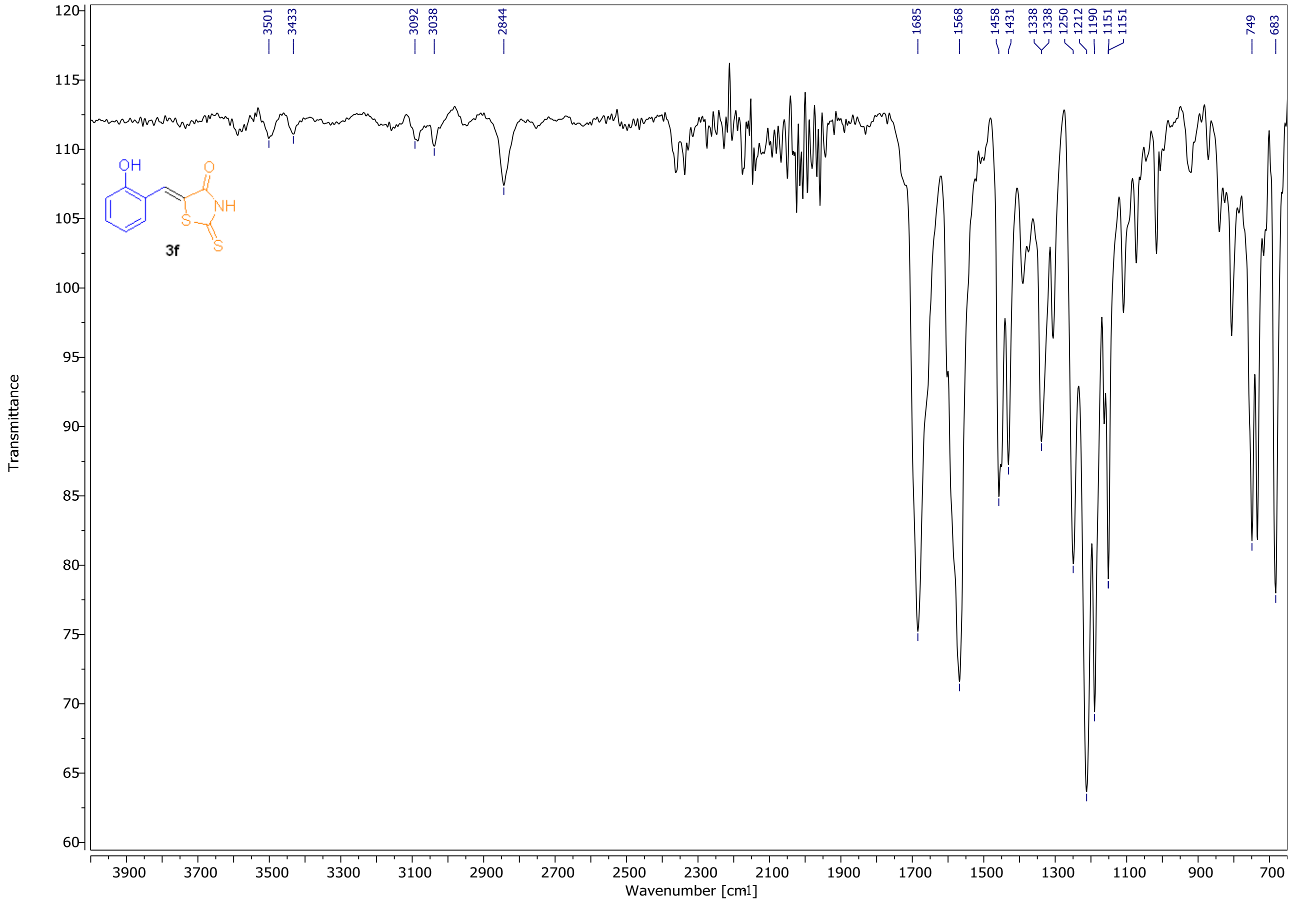

Figure S22. FTIR (ATR) of compound **3f**.

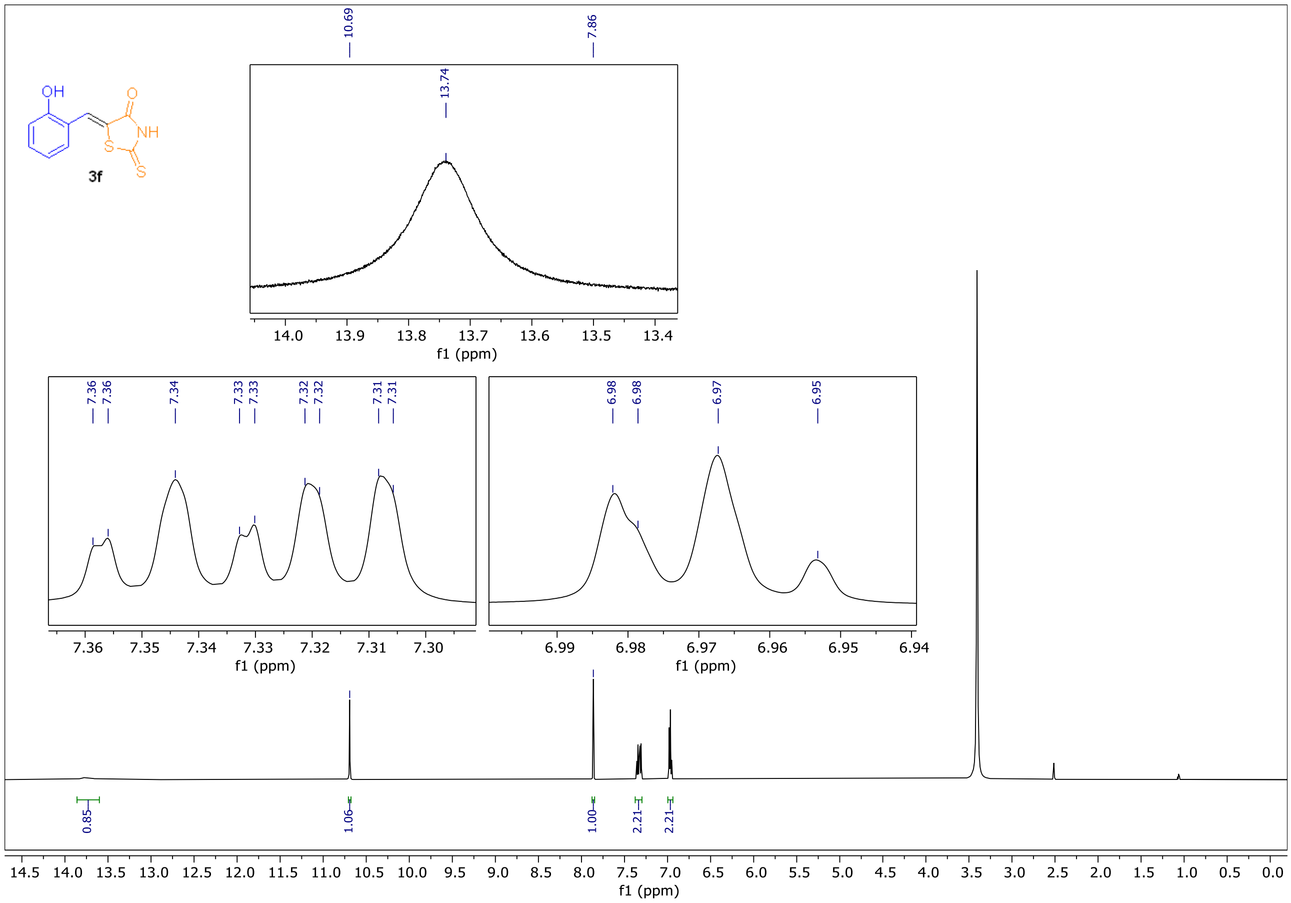

Figure S23. ^{1}H NMR spectrum (600 MHz, DMSO-d_6) of compound **3f**.

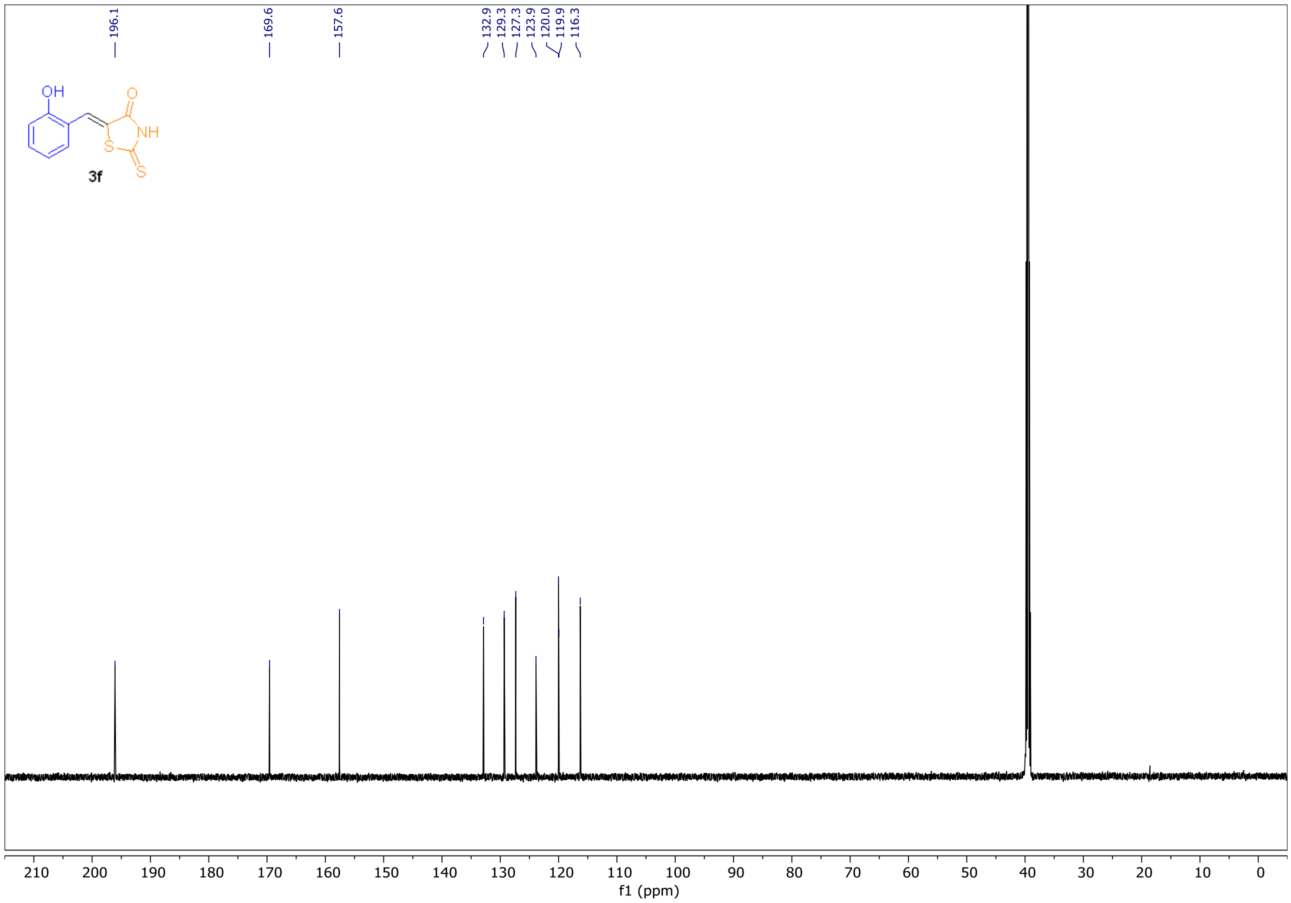

Figure S24. ^{13}C NMR spectrum (151 MHz, DMSO-d_6) of compound **3f**.

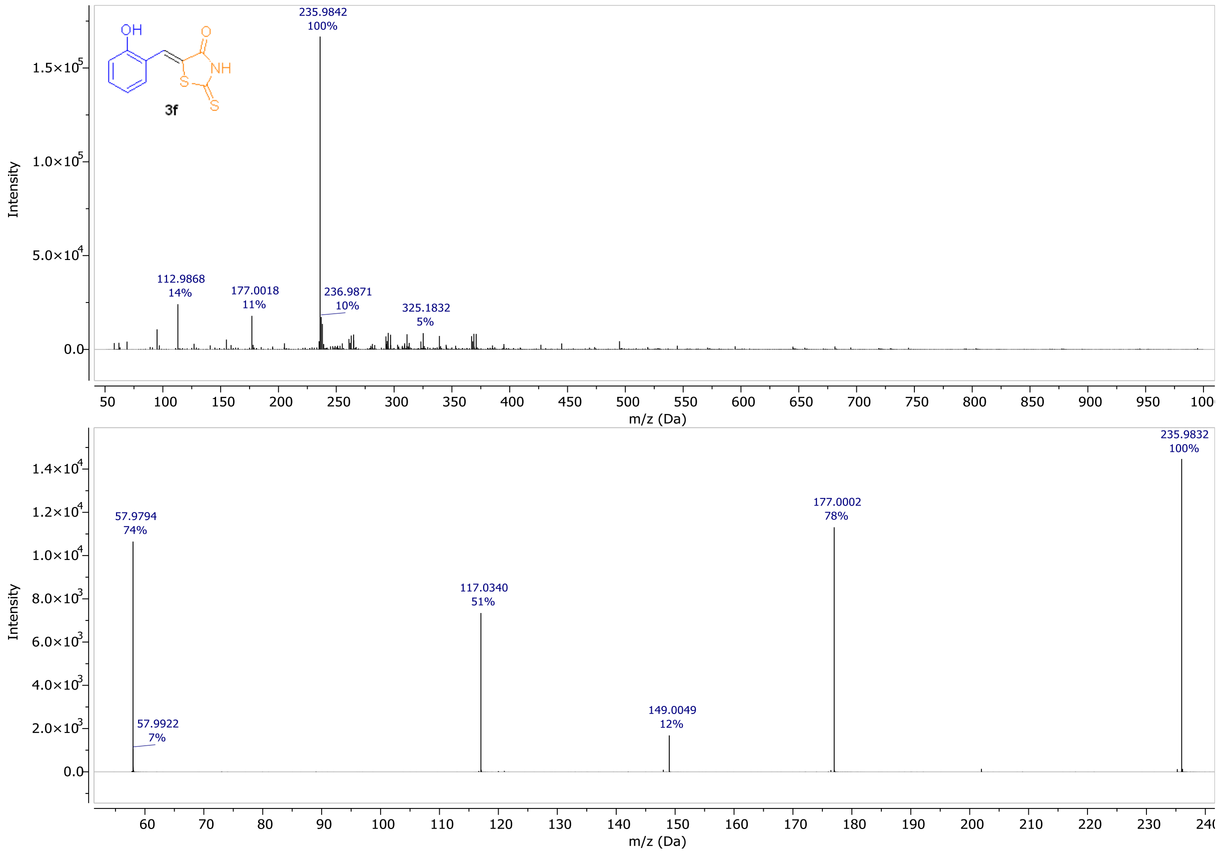

Figure S25. HRMS (ESI-QTOF) of compound **3f** and HRMS/MS for [M−H]$^-$.

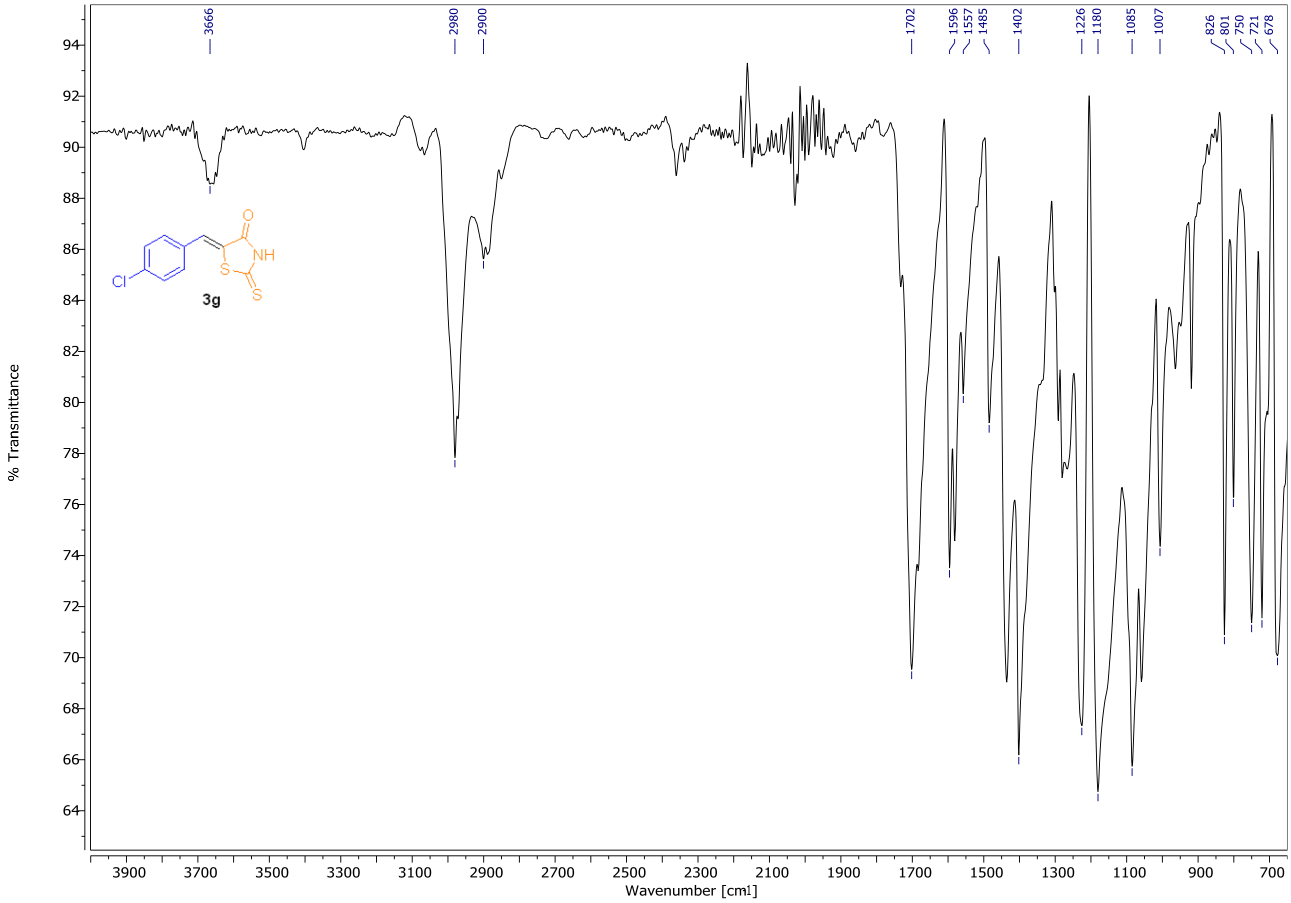

Figure S26. FTIR (ATR) of compound **3g**.

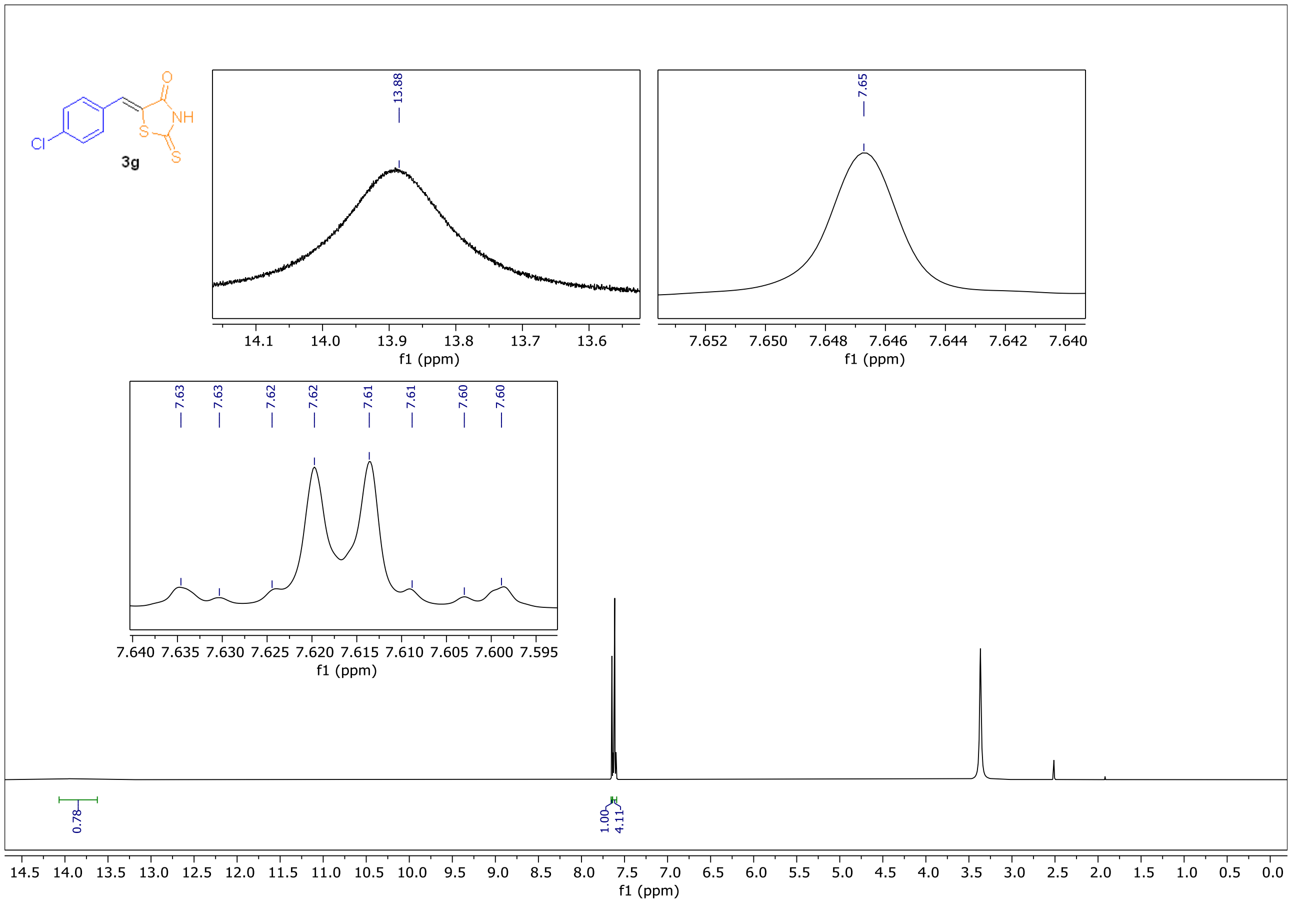

Figure S27. ^{1}H NMR spectrum (600 MHz, DMSO-d_6) of compound **3g**.

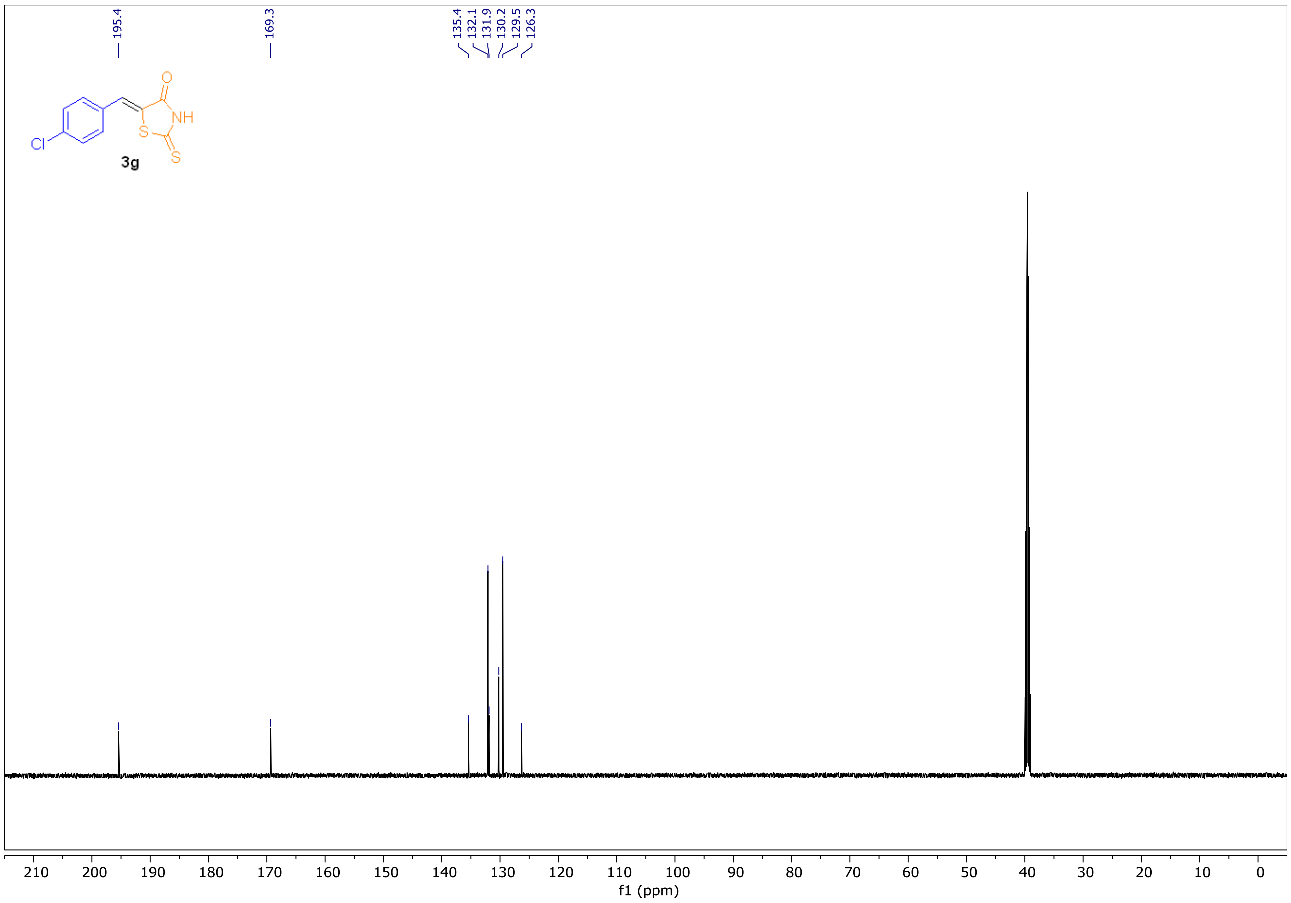

Figure S28. ^{13}C NMR spectrum (151 MHz, DMSO-d_6) of compound **3g**.

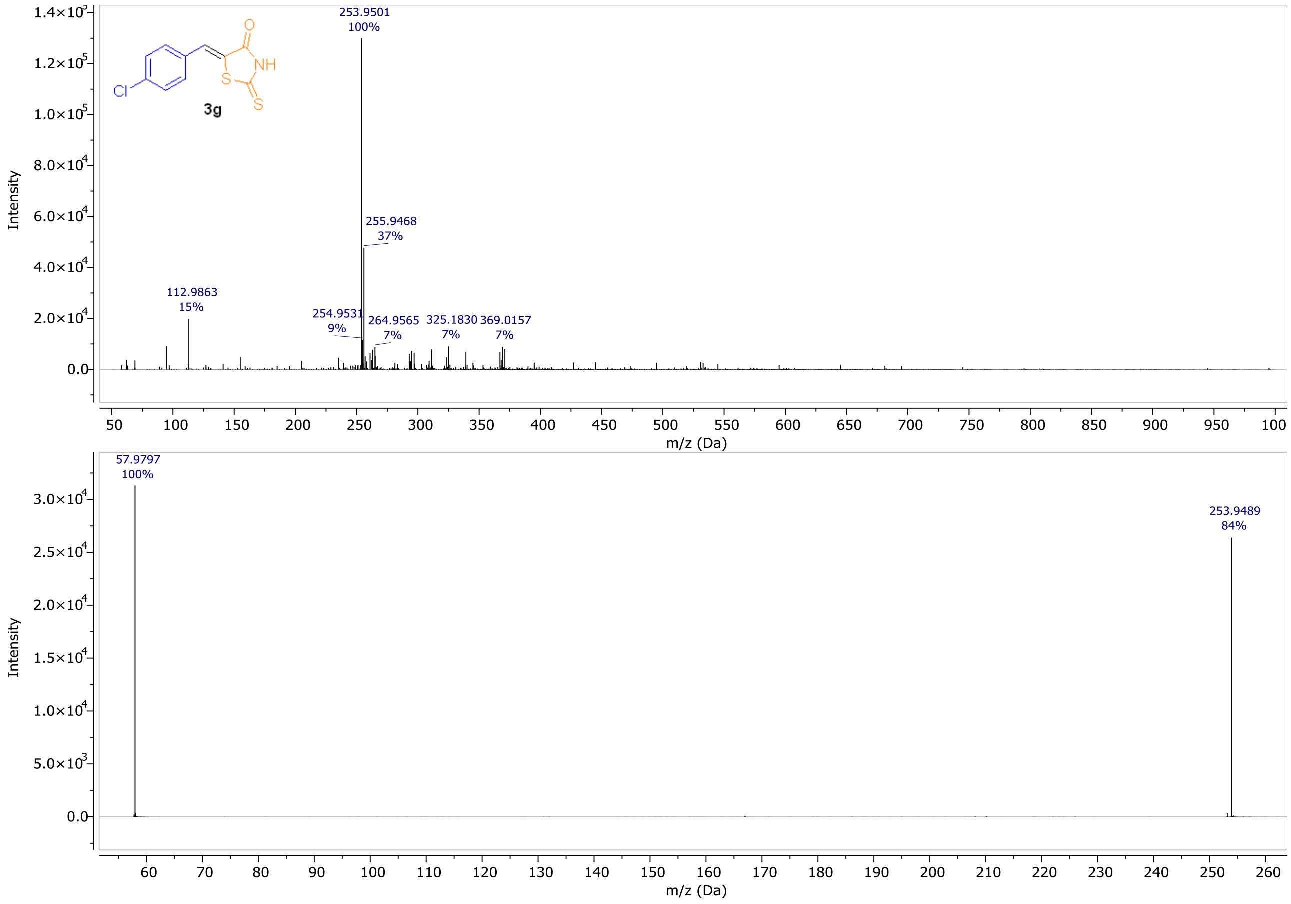

Figure S29. HRMS (ESI-QTOF) of compound **3g** and HRMS/MS for [M−H]$^-$.

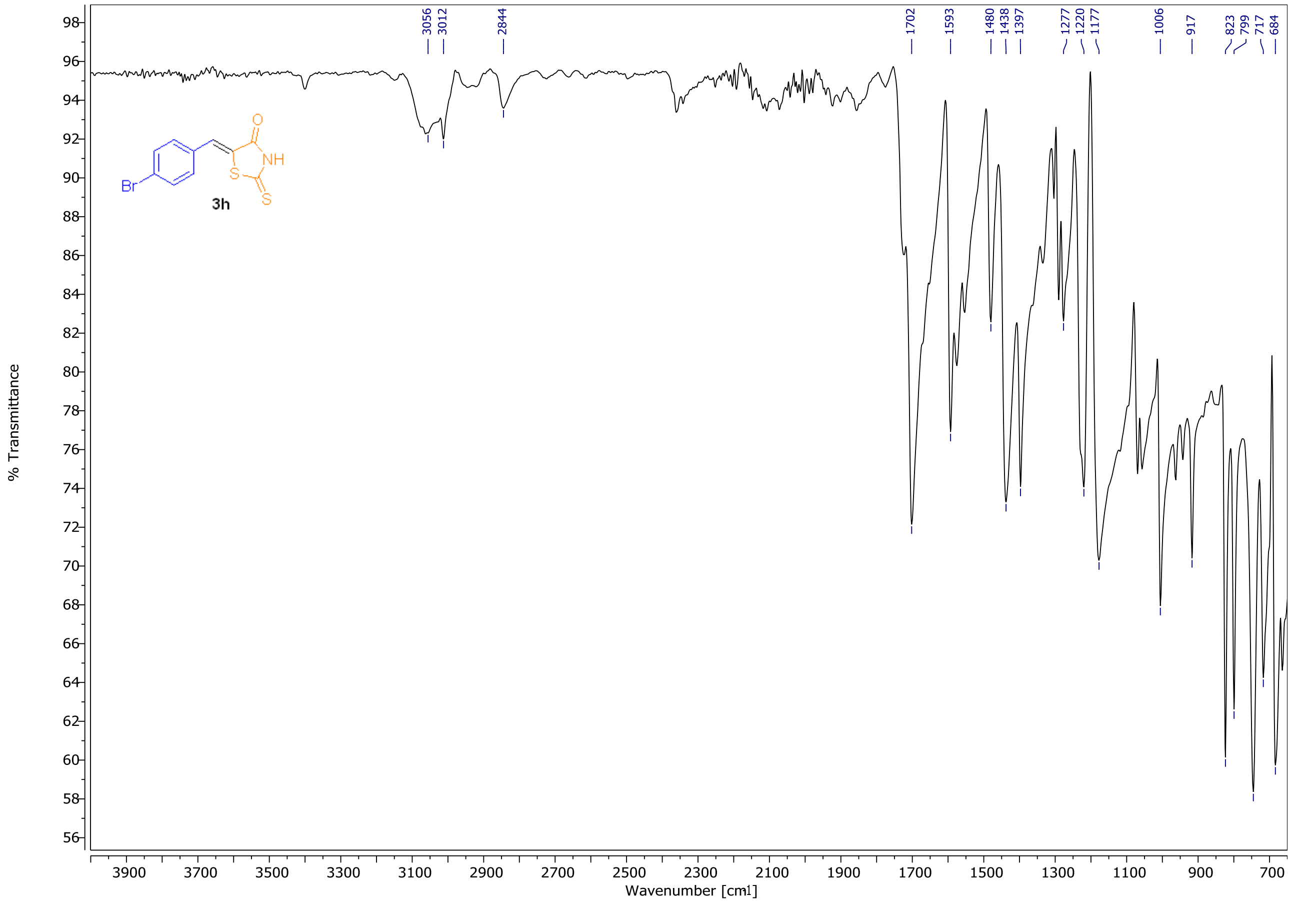

Figure S30. FTIR (ATR) of compound **3h**.

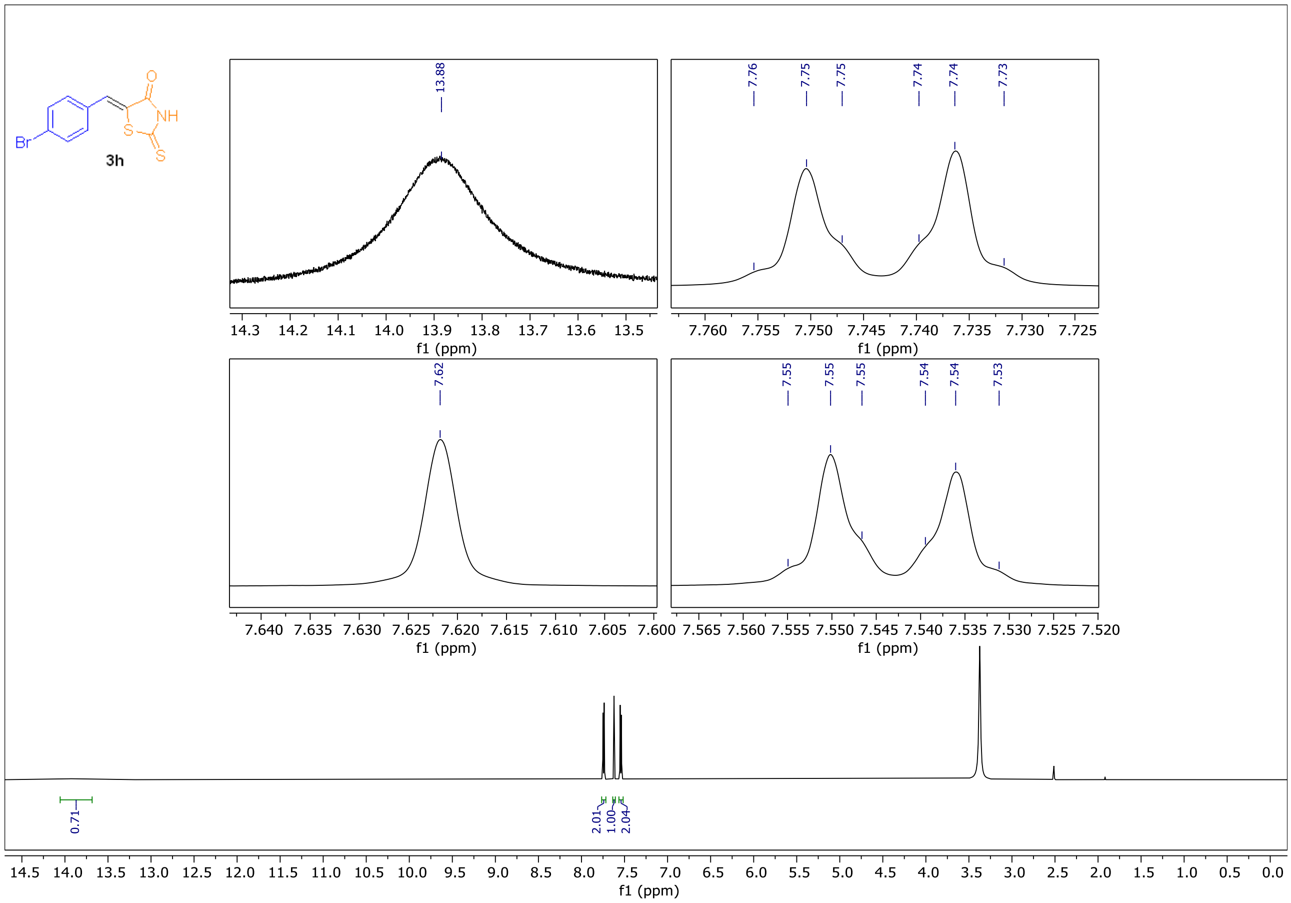

Figure S31. ^{1}H NMR spectrum (600 MHz, DMSO-d_6) of compound **3h**.

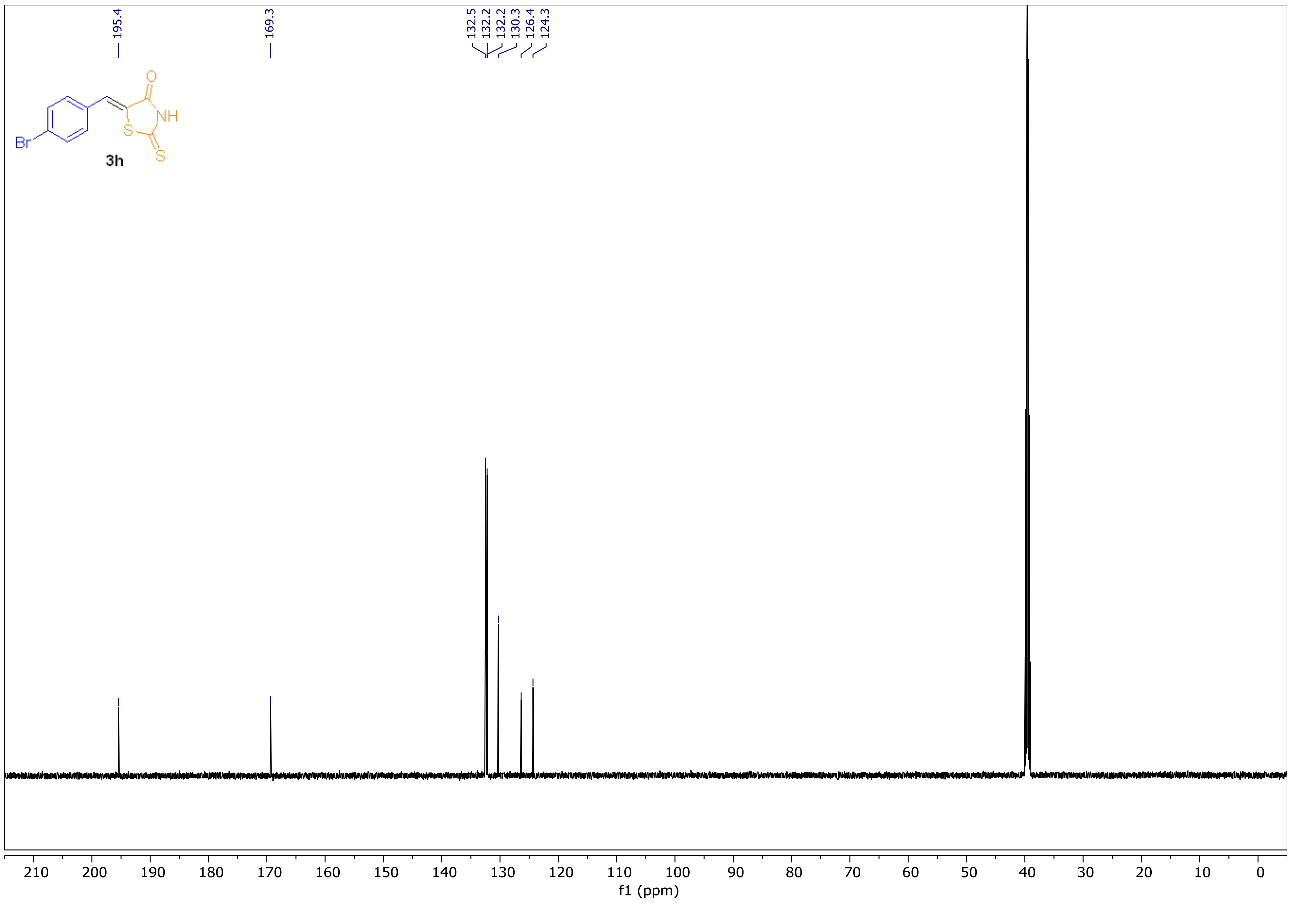

Figure S32. ^{13}C NMR spectrum (151 MHz, DMSO-d_6) of compound **3h**.

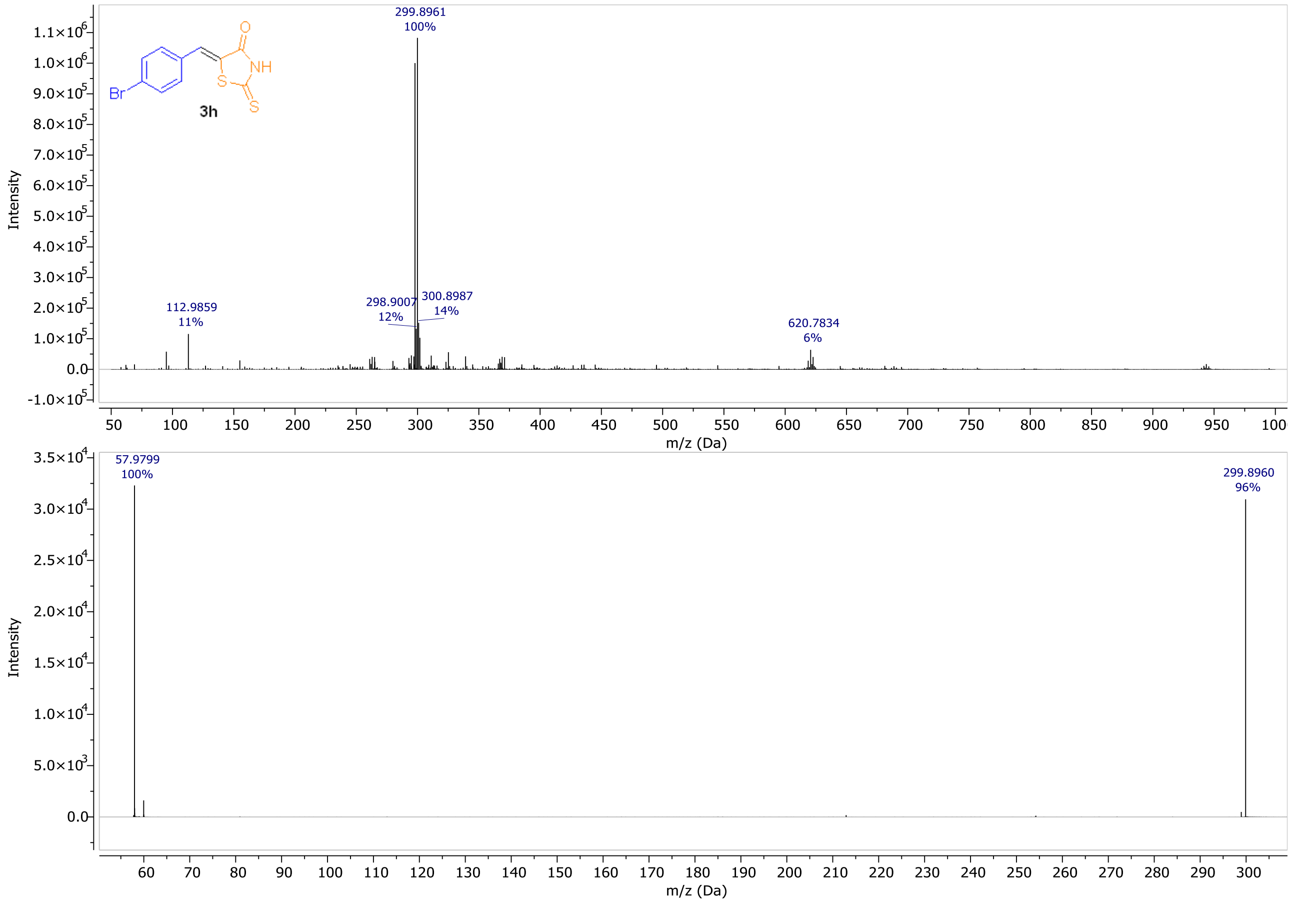

Figure S33. HRMS (ESI-QTOF) of compound **3h** and HRMS/MS for [M−H]⁻.

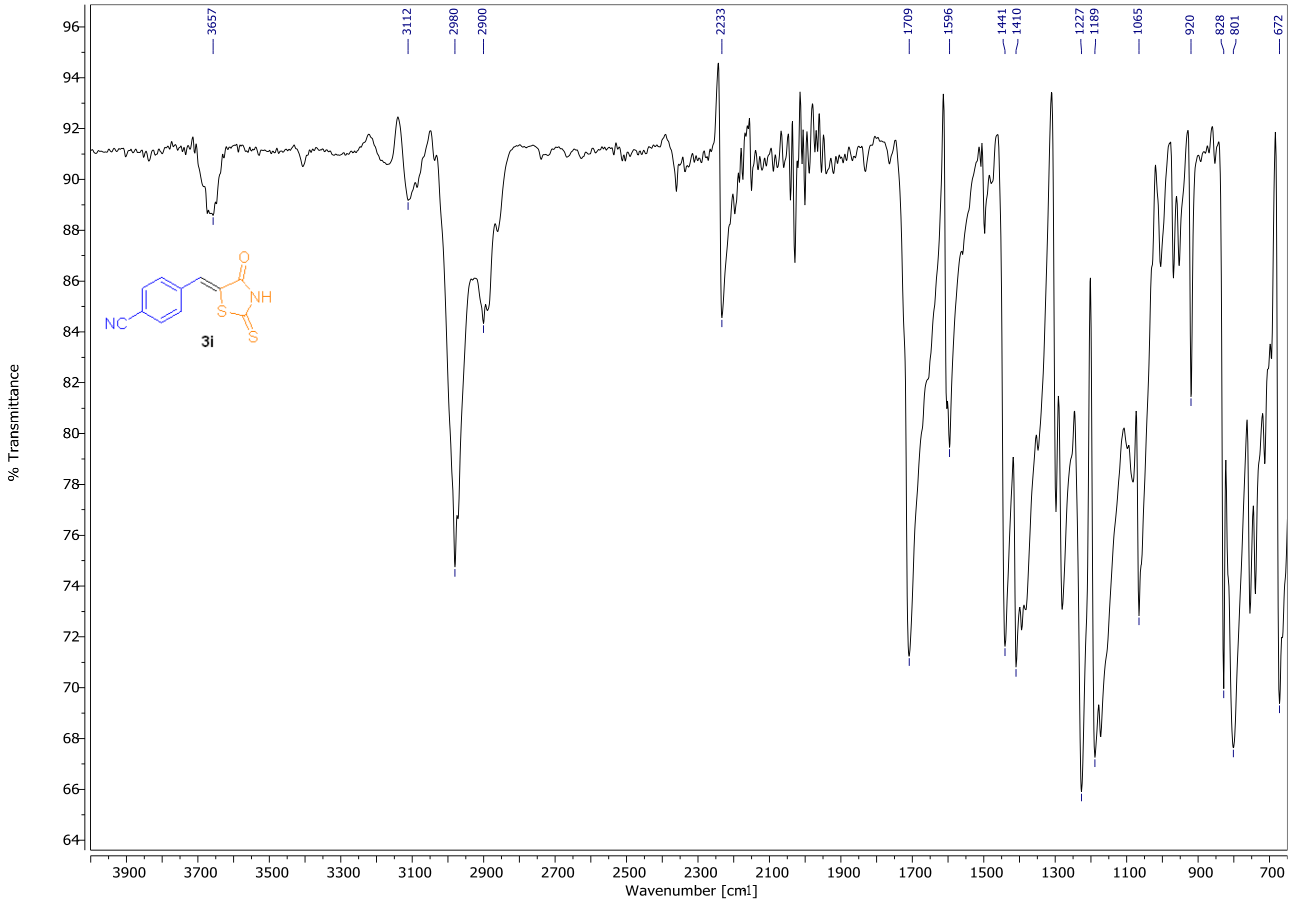

Figure S34. FTIR (ATR) of compound **3i**.

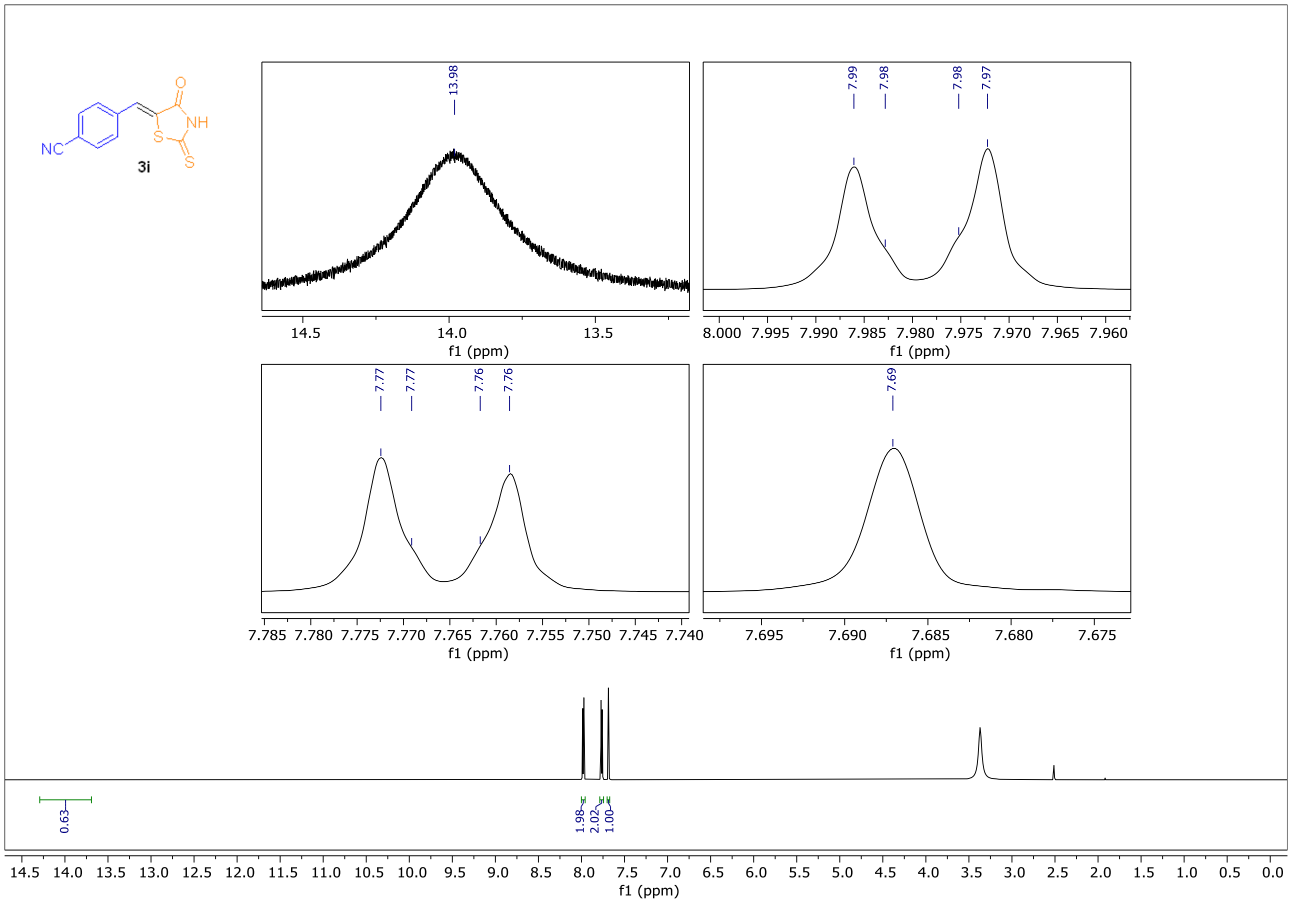

Figure S35. ^{1}H NMR spectrum (600 MHz, DMSO-d_6) of compound **3i**.

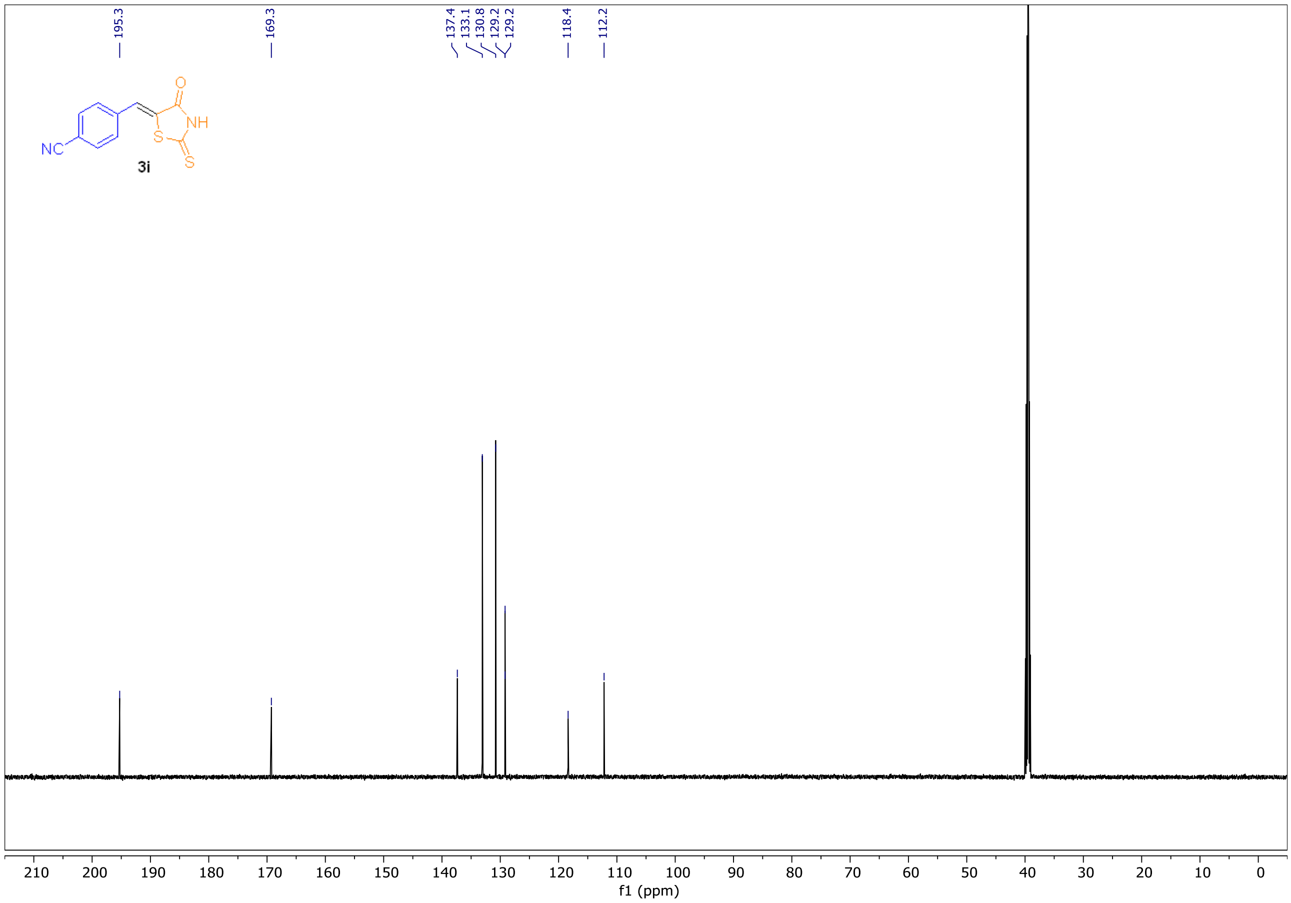

Figure S36. ^{13}C NMR spectrum (151 MHz, DMSO-d_6) of compound **3i**.

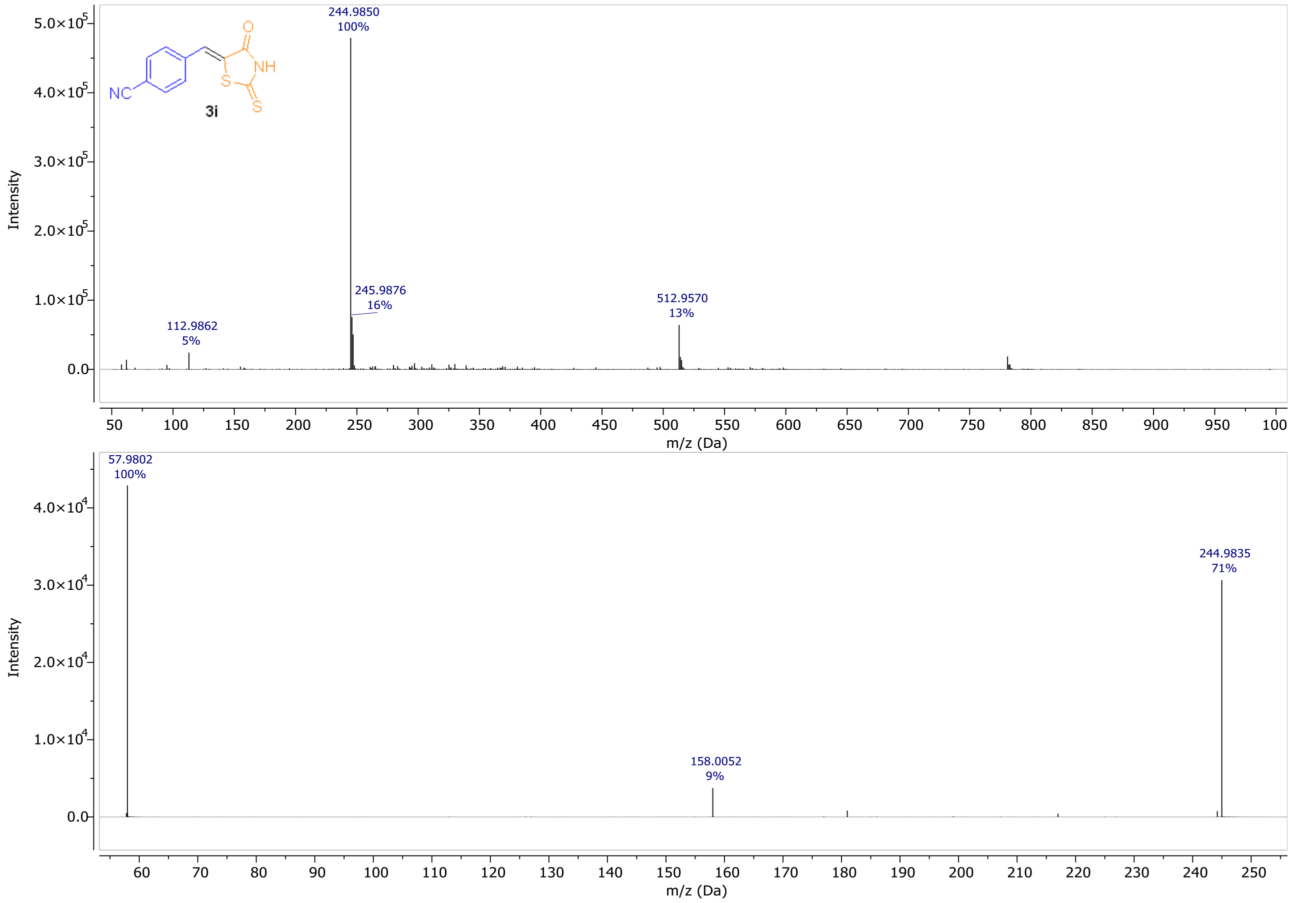

Figure S37. HRMS (ESI-QTOF) of compound **3i** and HRMS/MS for [M−H]$^-$.

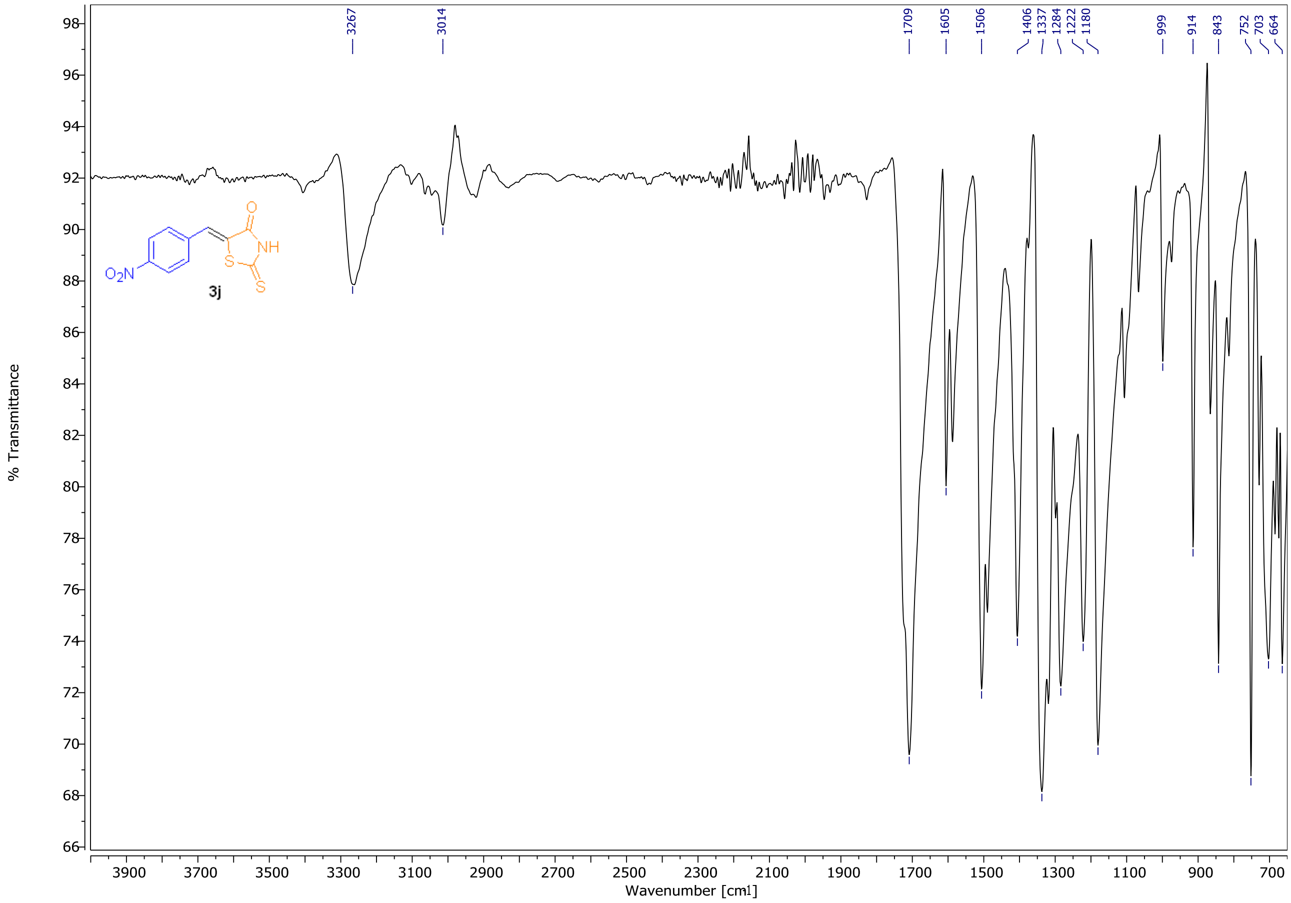

Figure S38. FTIR (ATR) of compound **3j**.

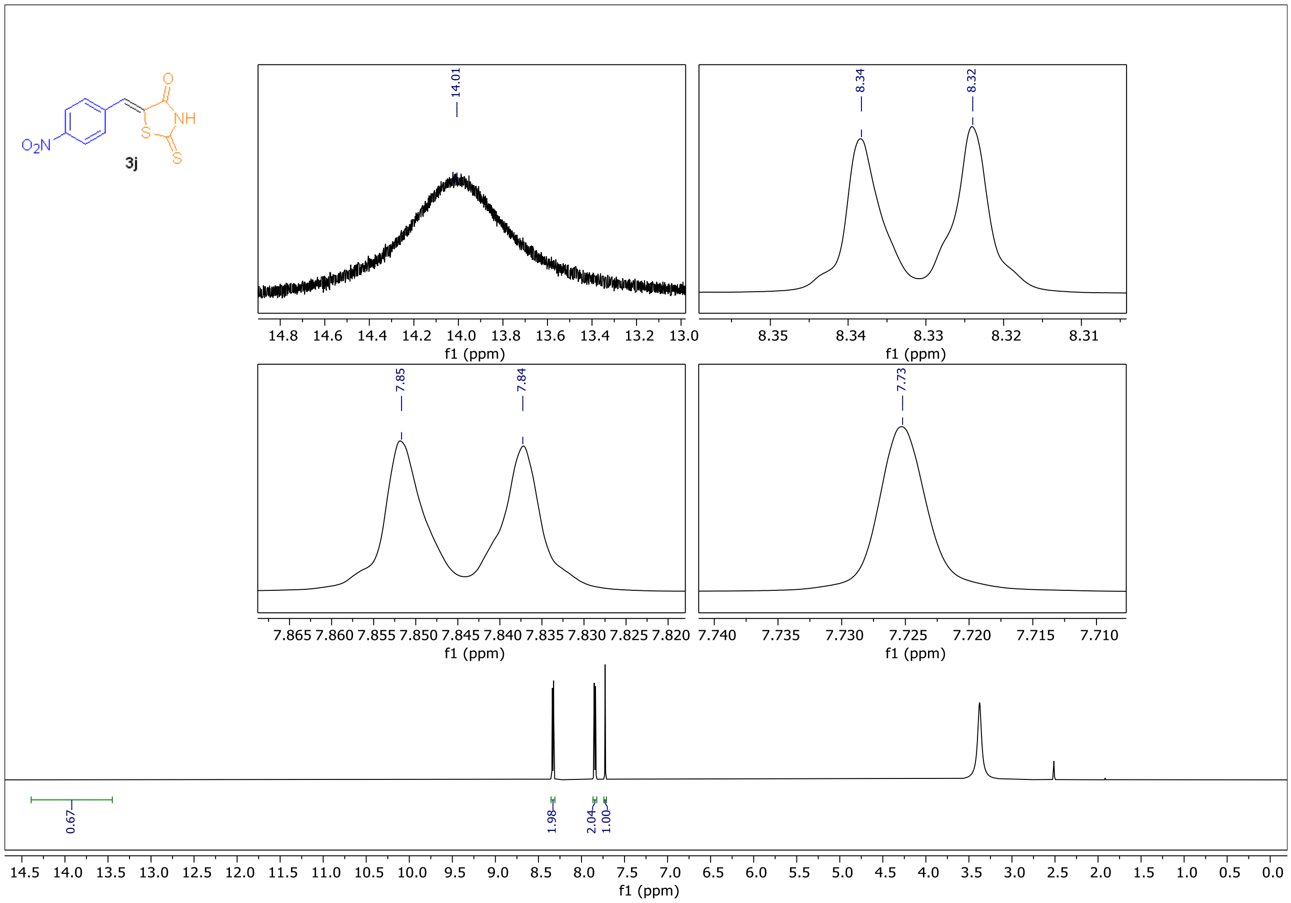

Figure S39. ^{1}H NMR spectrum (600 MHz, DMSO-d_6) of compound **3j**.

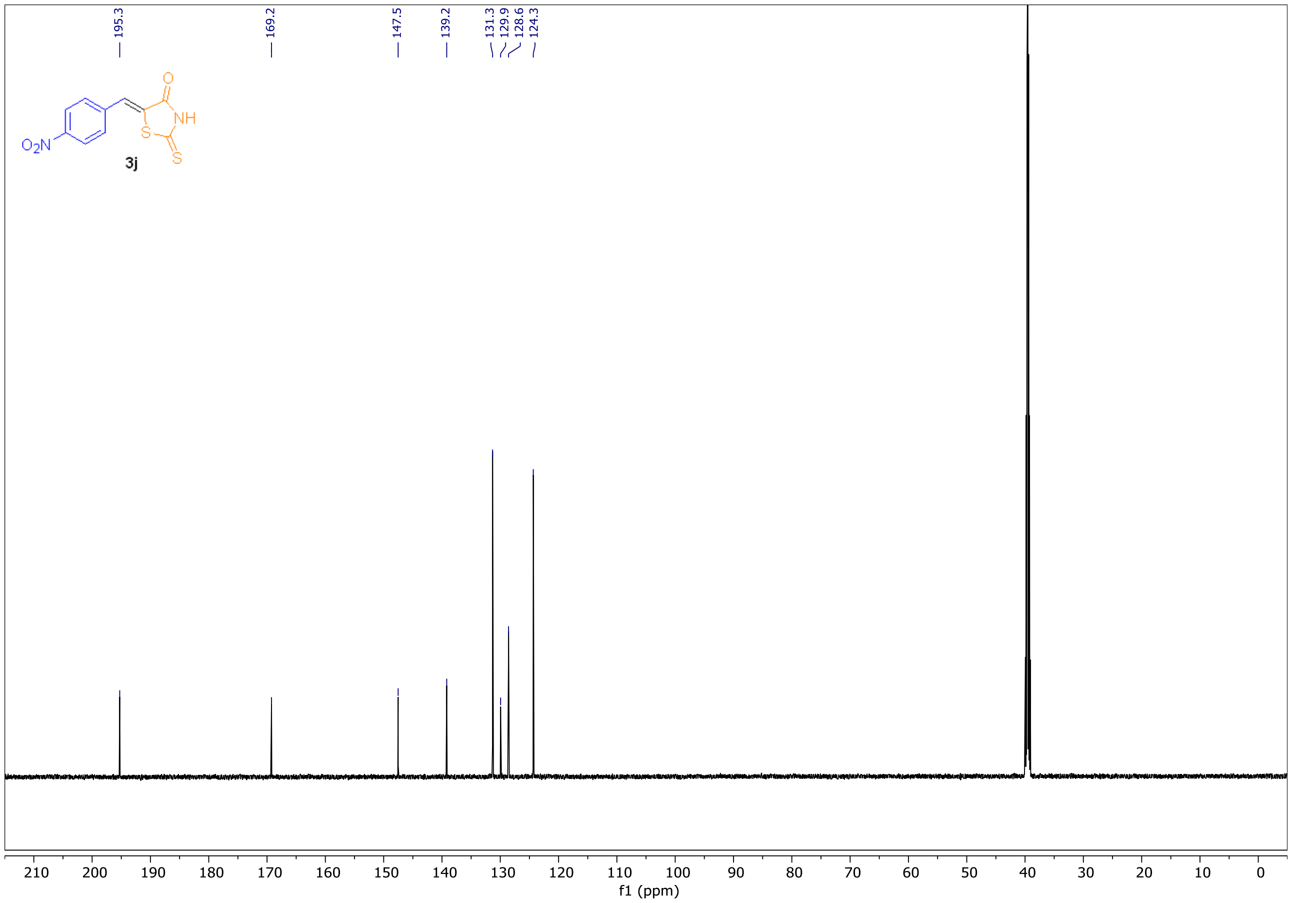

Figure S40. ^{13}C NMR spectrum (151 MHz, DMSO-d_6) of compound **3j**.

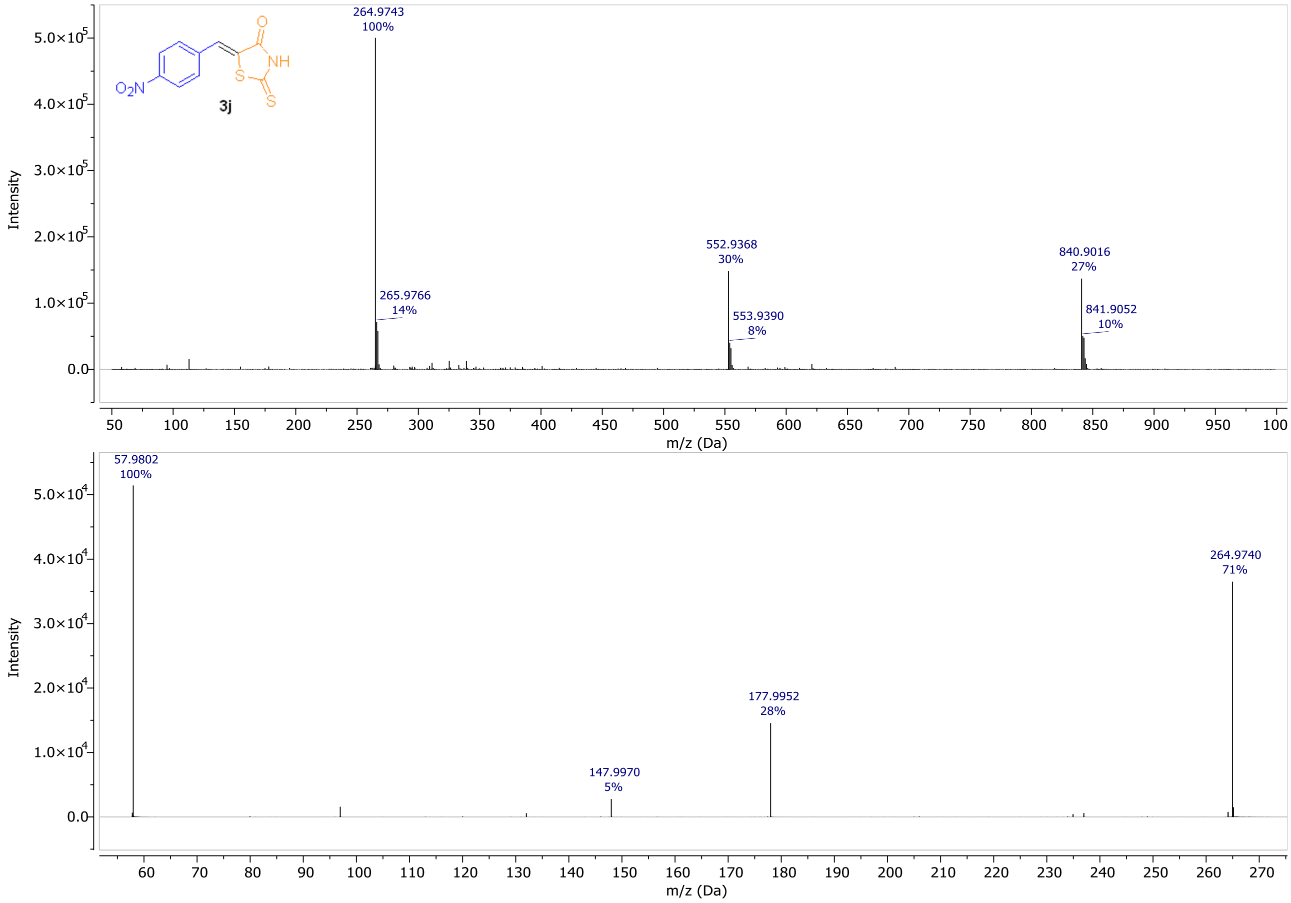

Figure S41. HRMS (ESI-QTOF) of compound **3j** and HRMS/MS for [M−H]⁻.

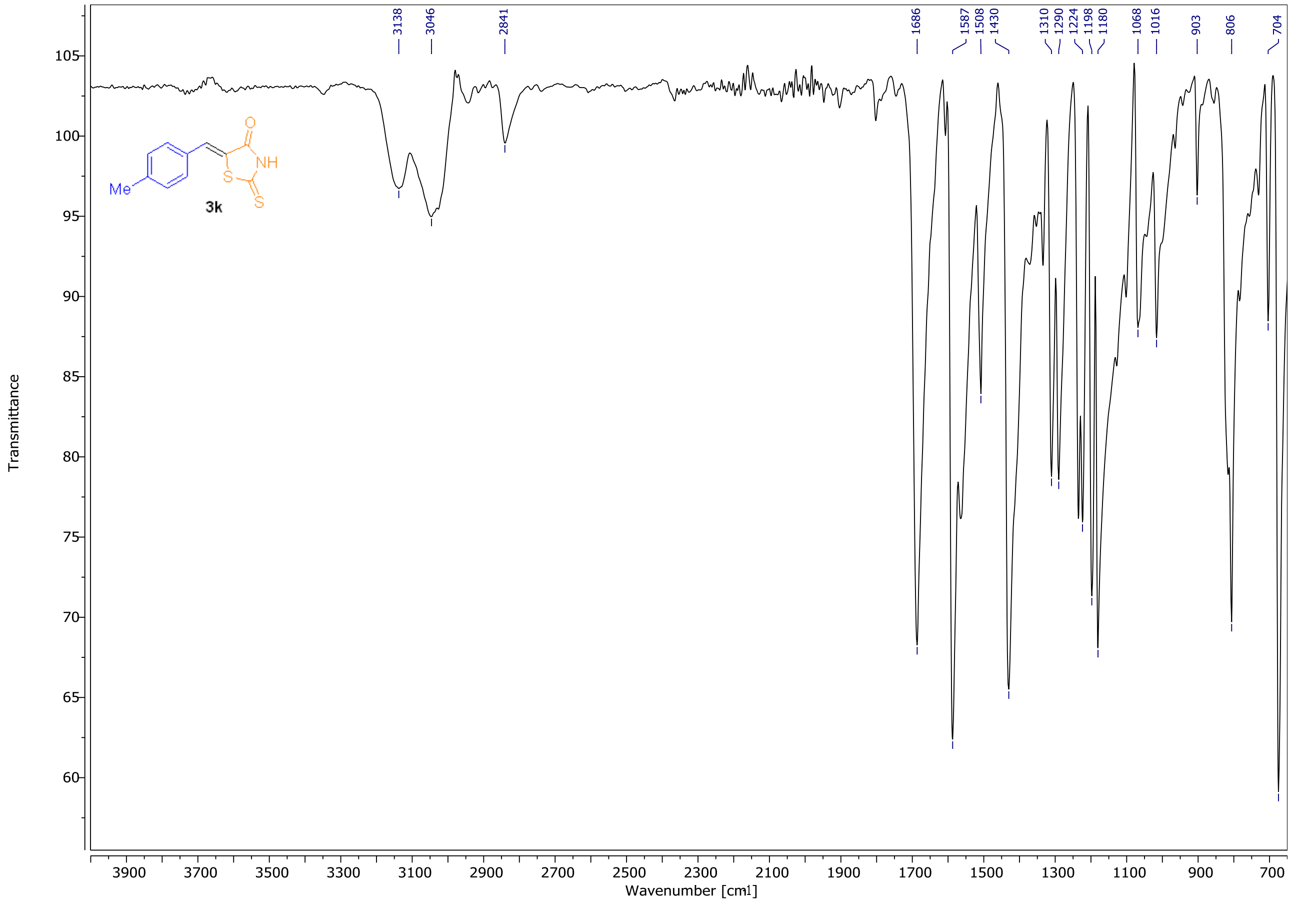

Figure S42. FTIR (ATR) of compound **3k**.

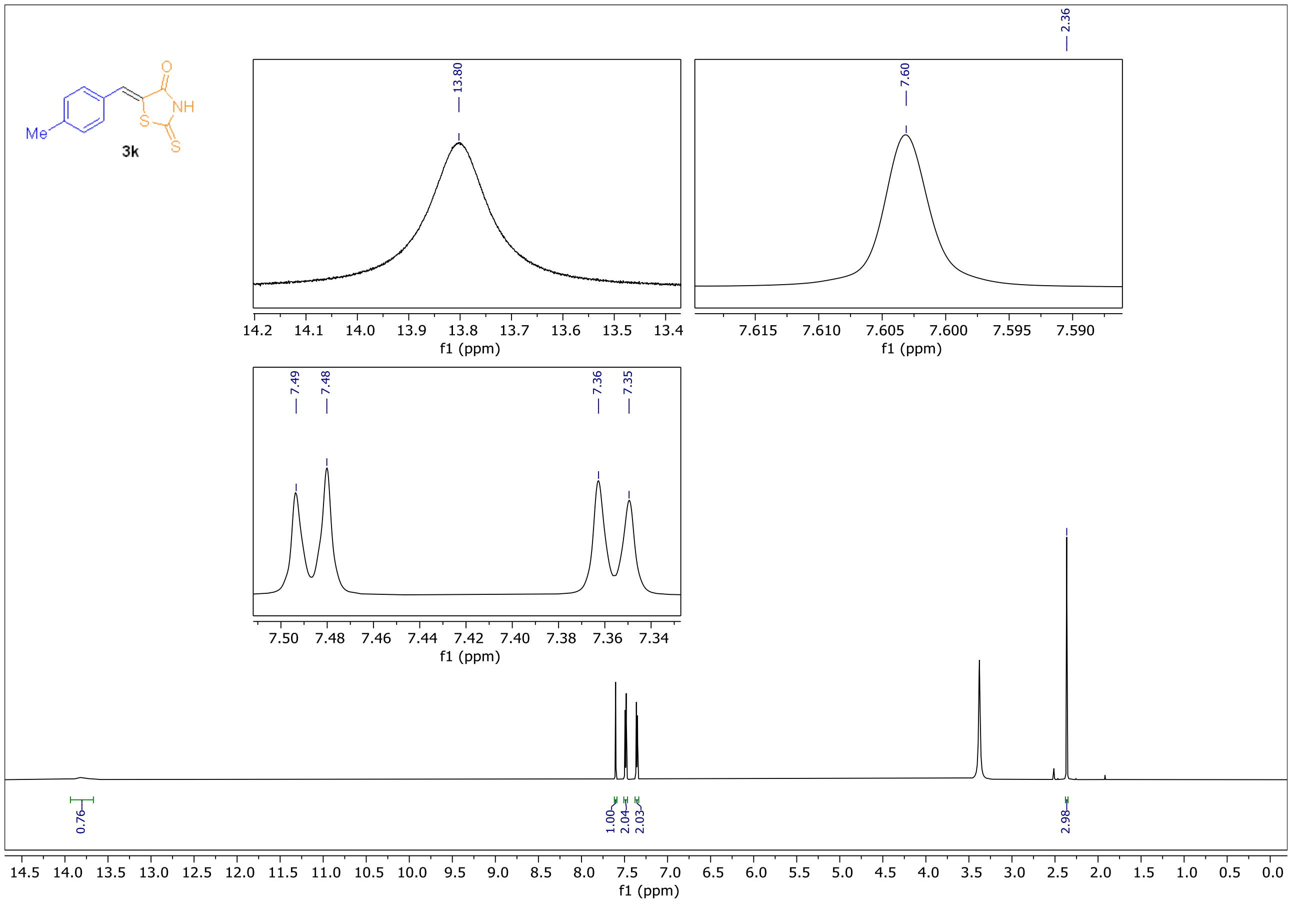

Figure S43. ^{1}H NMR spectrum (600 MHz, DMSO-d_6) of compound **3k**.

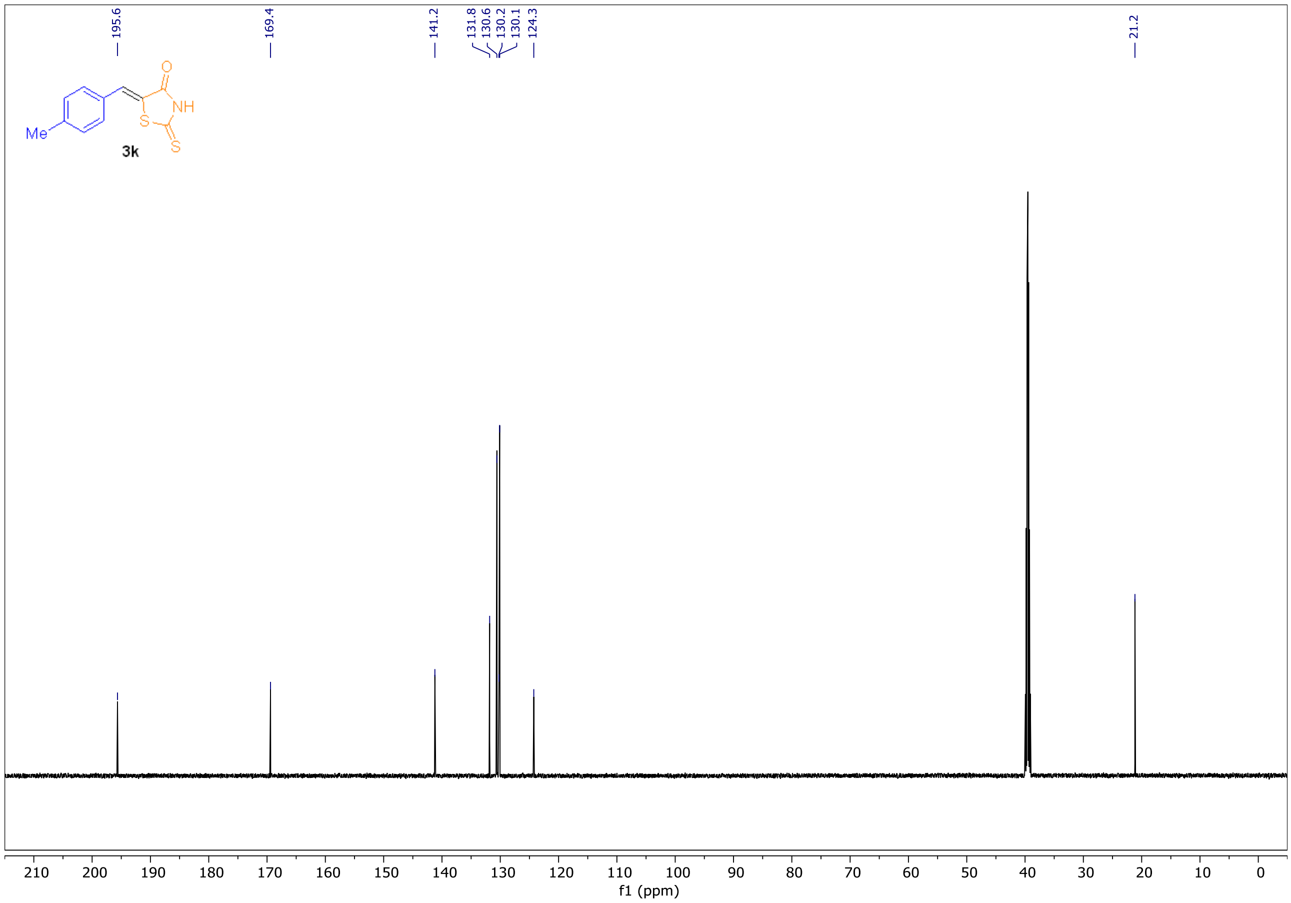

Figure S44. ^{13}C NMR spectrum (151 MHz, DMSO-d_6) of compound **3k**.

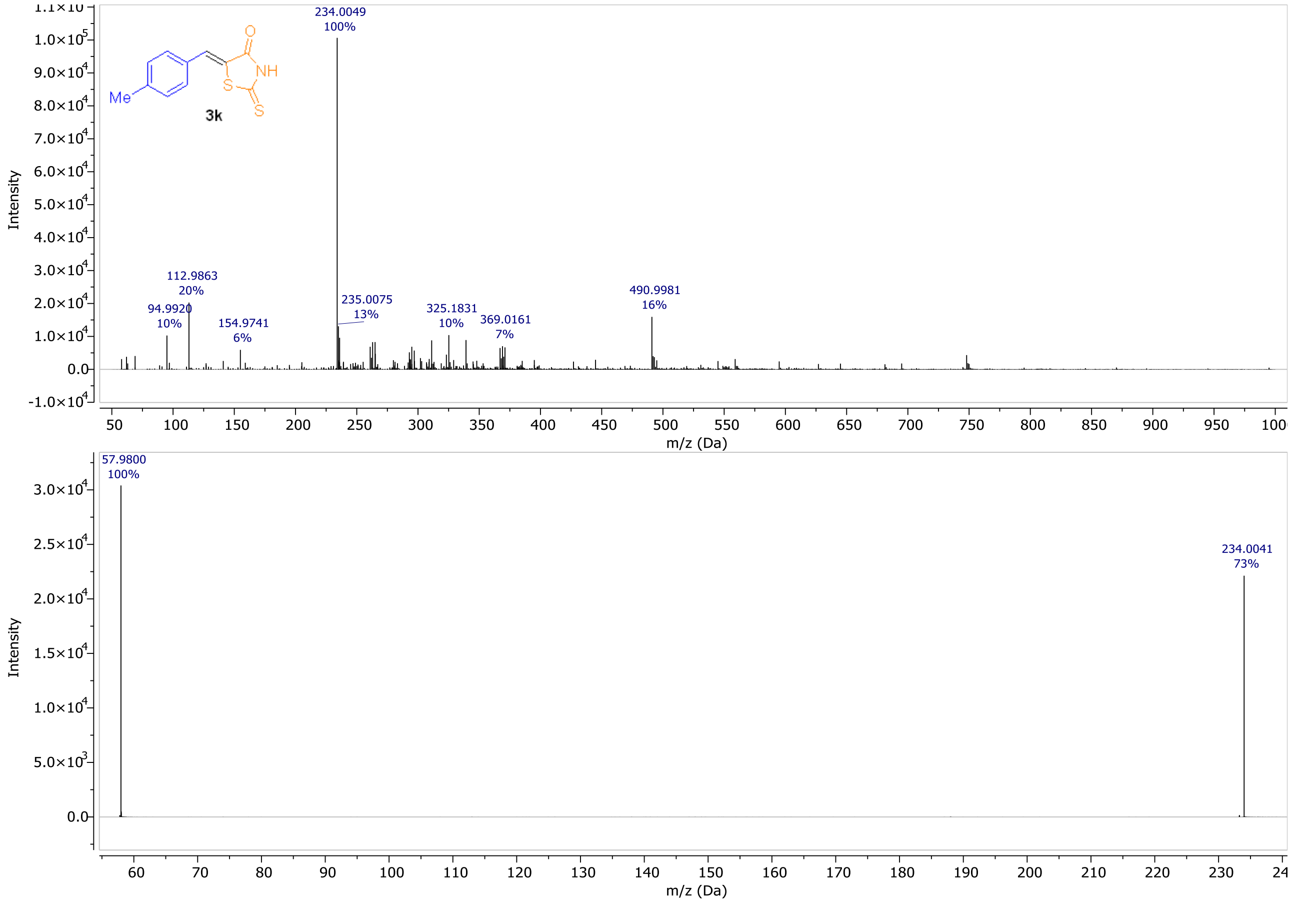

Figure S45. HRMS (ESI-QTOF) of compound **3k** and HRMS/MS for [M−H]⁻.

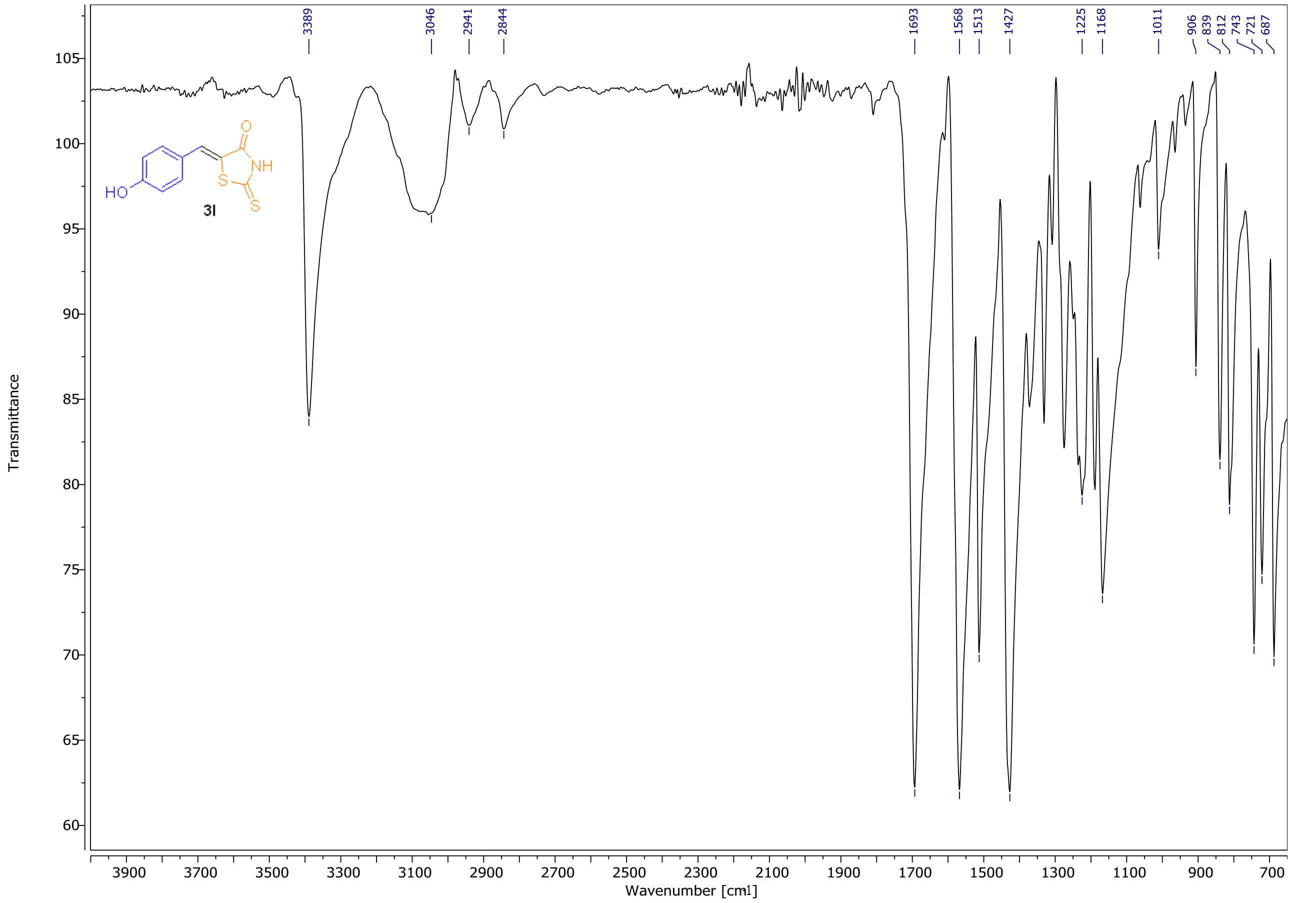

Figure S46. FTIR (ATR) of compound **3l**.

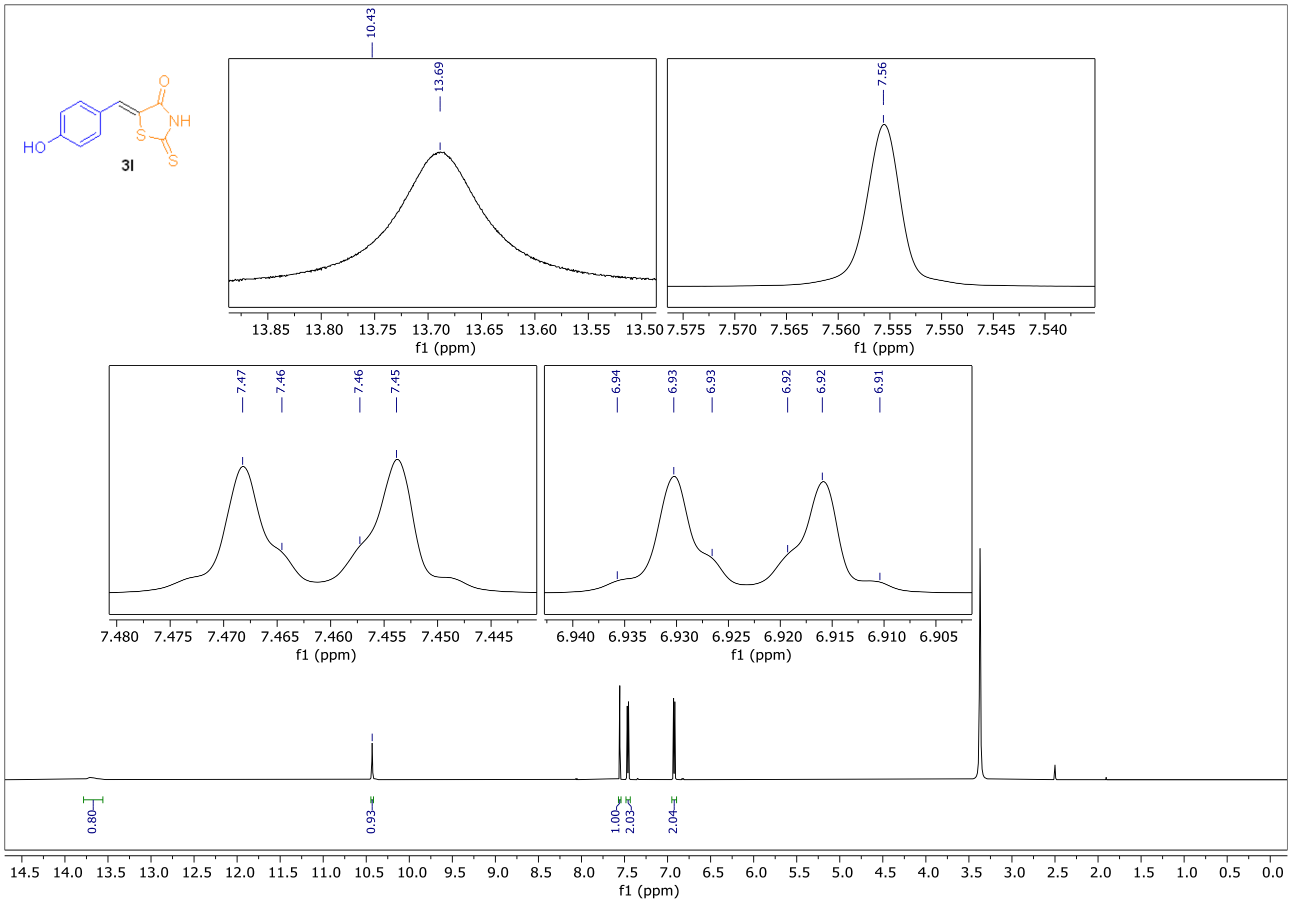

Figure S47. ^{1}H NMR spectrum (600 MHz, DMSO-d_6) of compound **3l**.

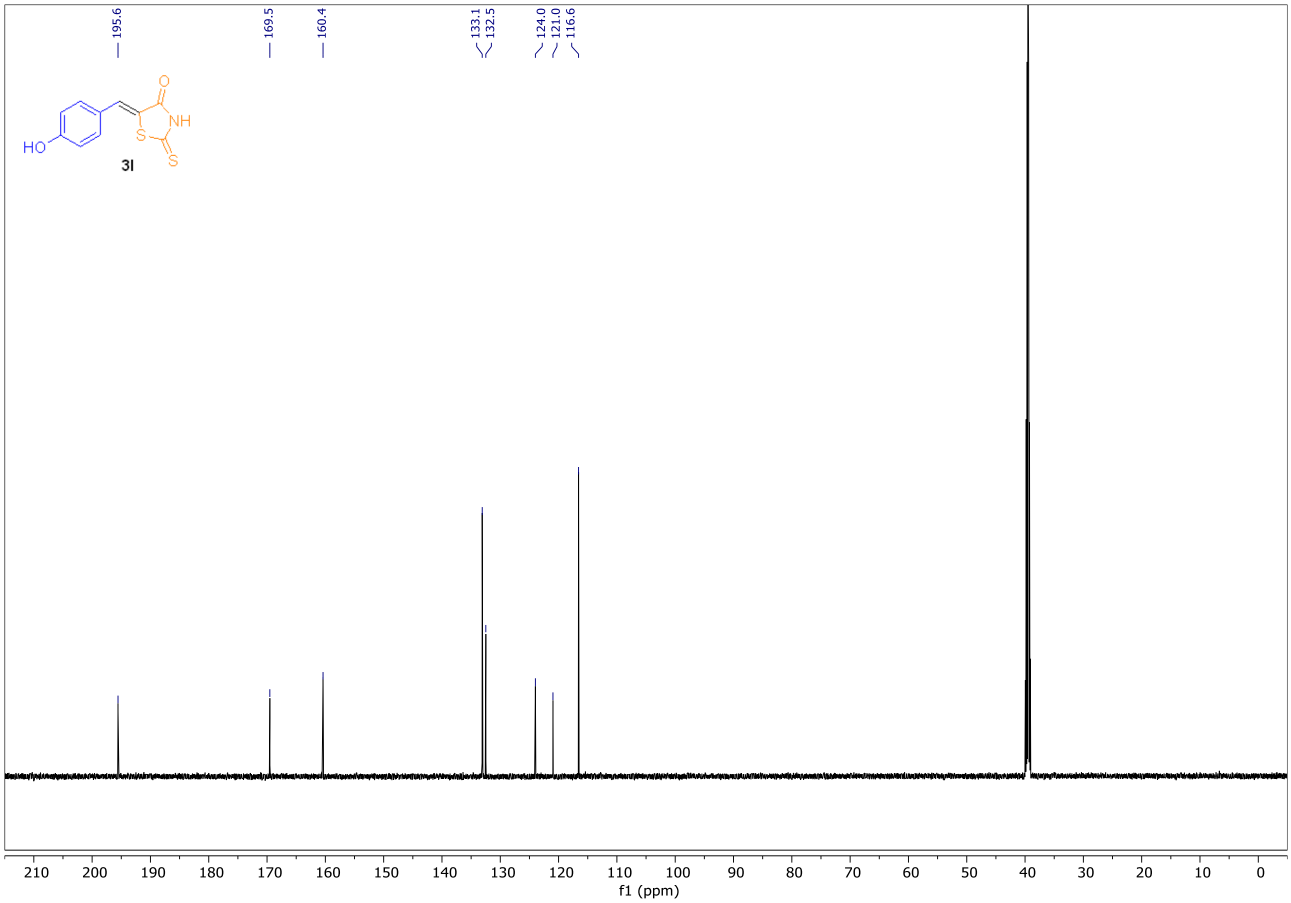

Figure S48. ^{13}C NMR spectrum (151 MHz, DMSO-d_6) of compound **3l**.

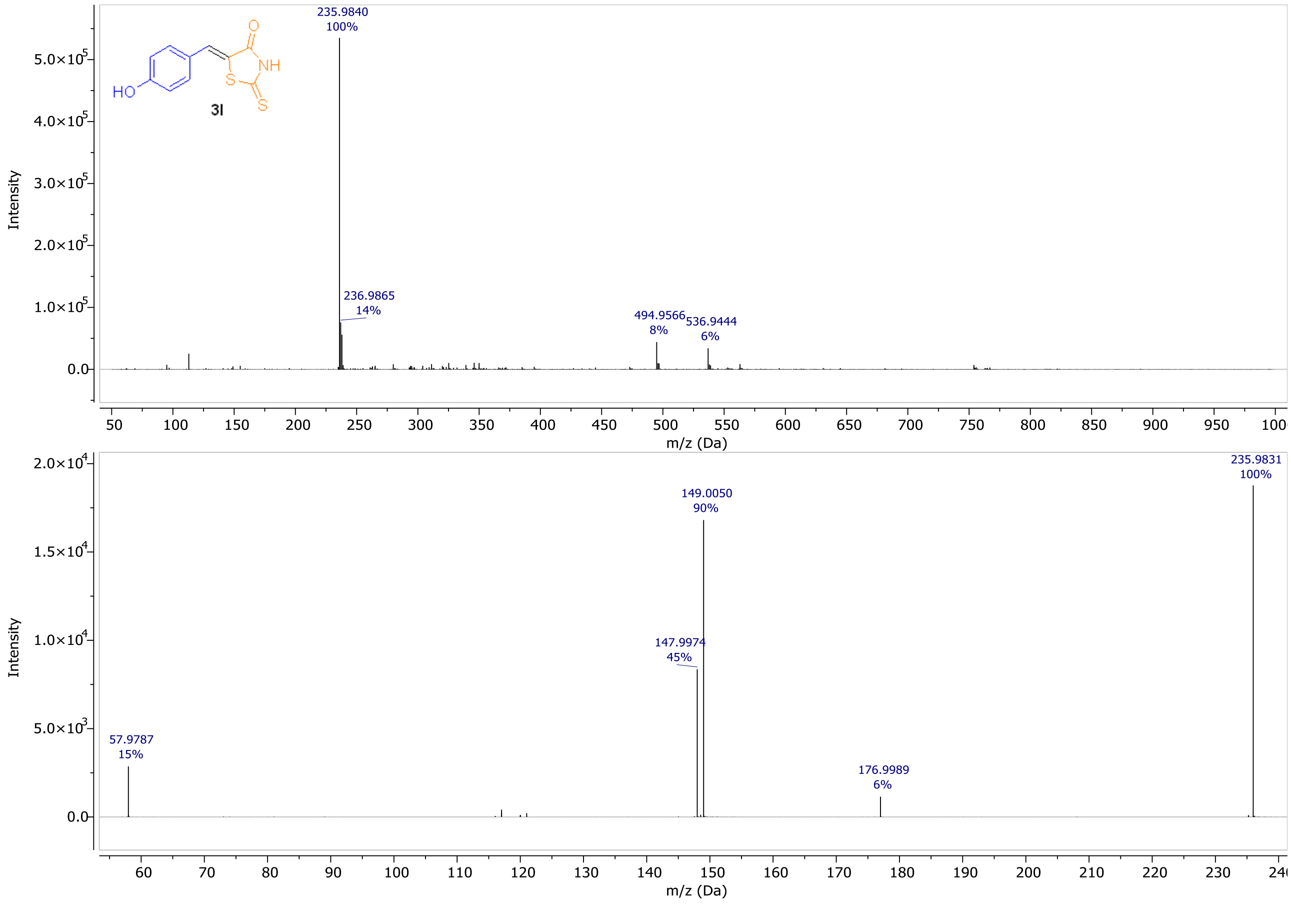

Figure S49. HRMS (ESI-QTOF) of compound **3l** and HRMS/MS for [M−H]$^-$.

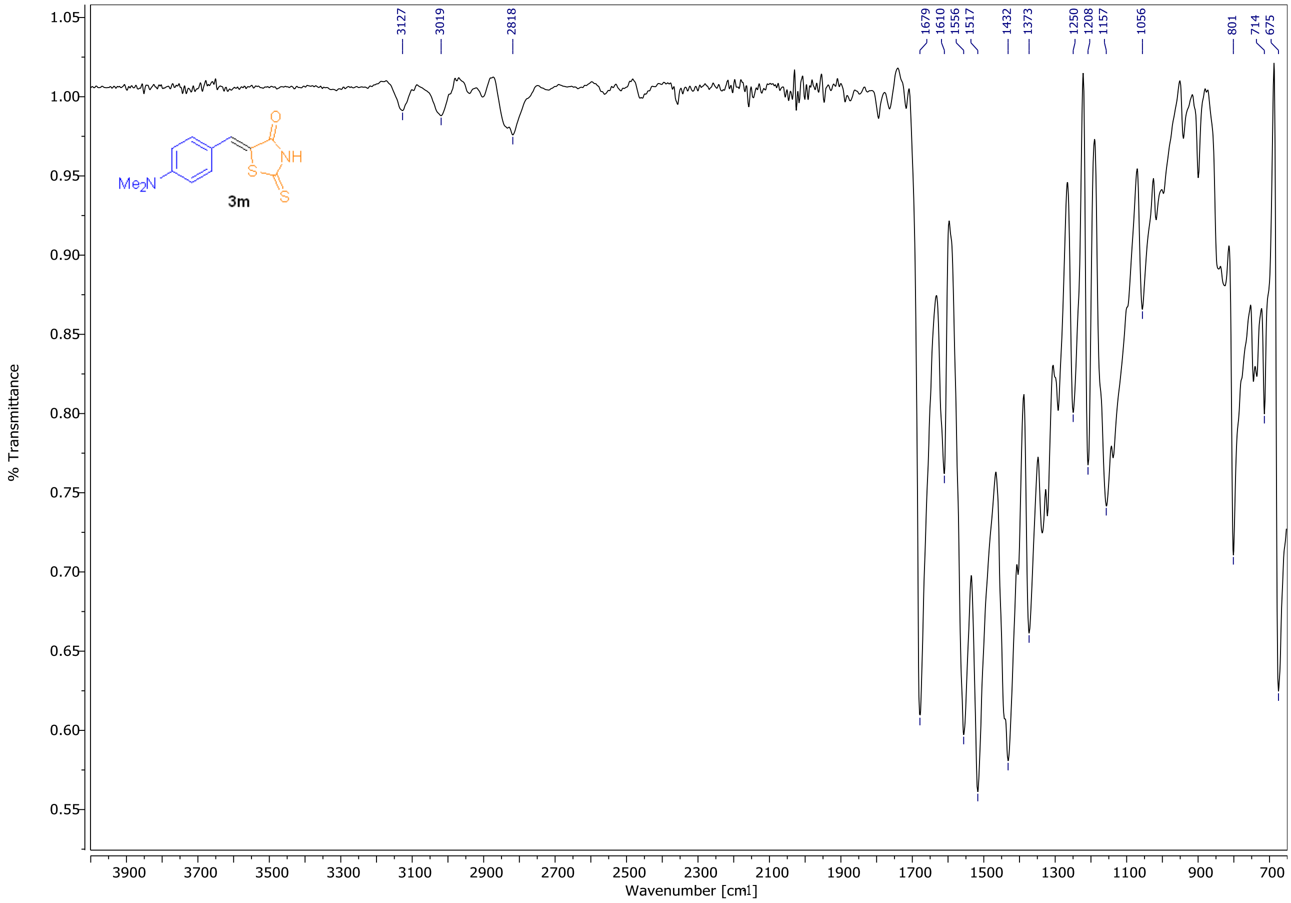

Figure S50. FTIR (ATR) of compound **3m**.

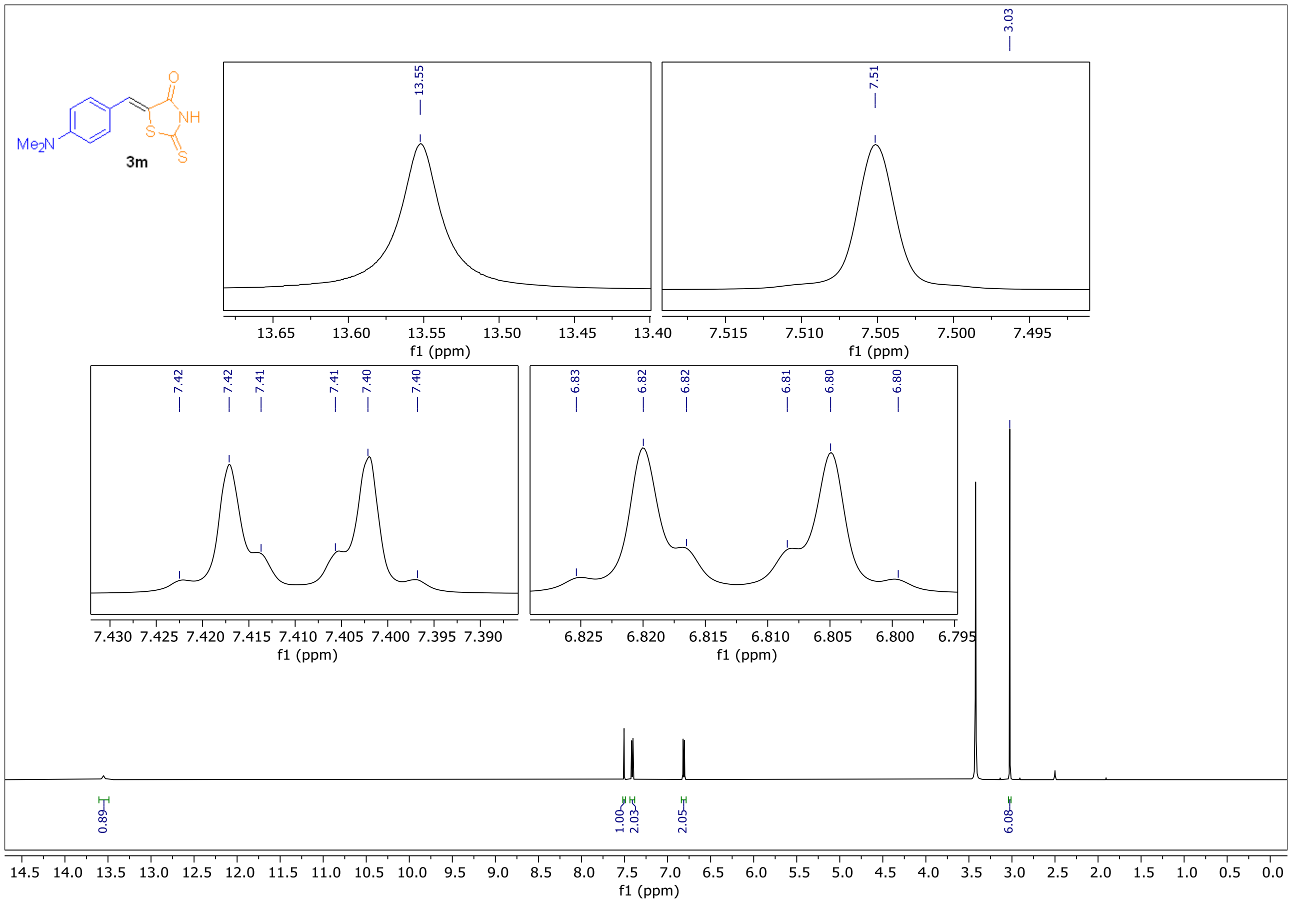

Figure S51. ^{1}H NMR spectrum (600 MHz, DMSO-d_6) of compound **3m**.

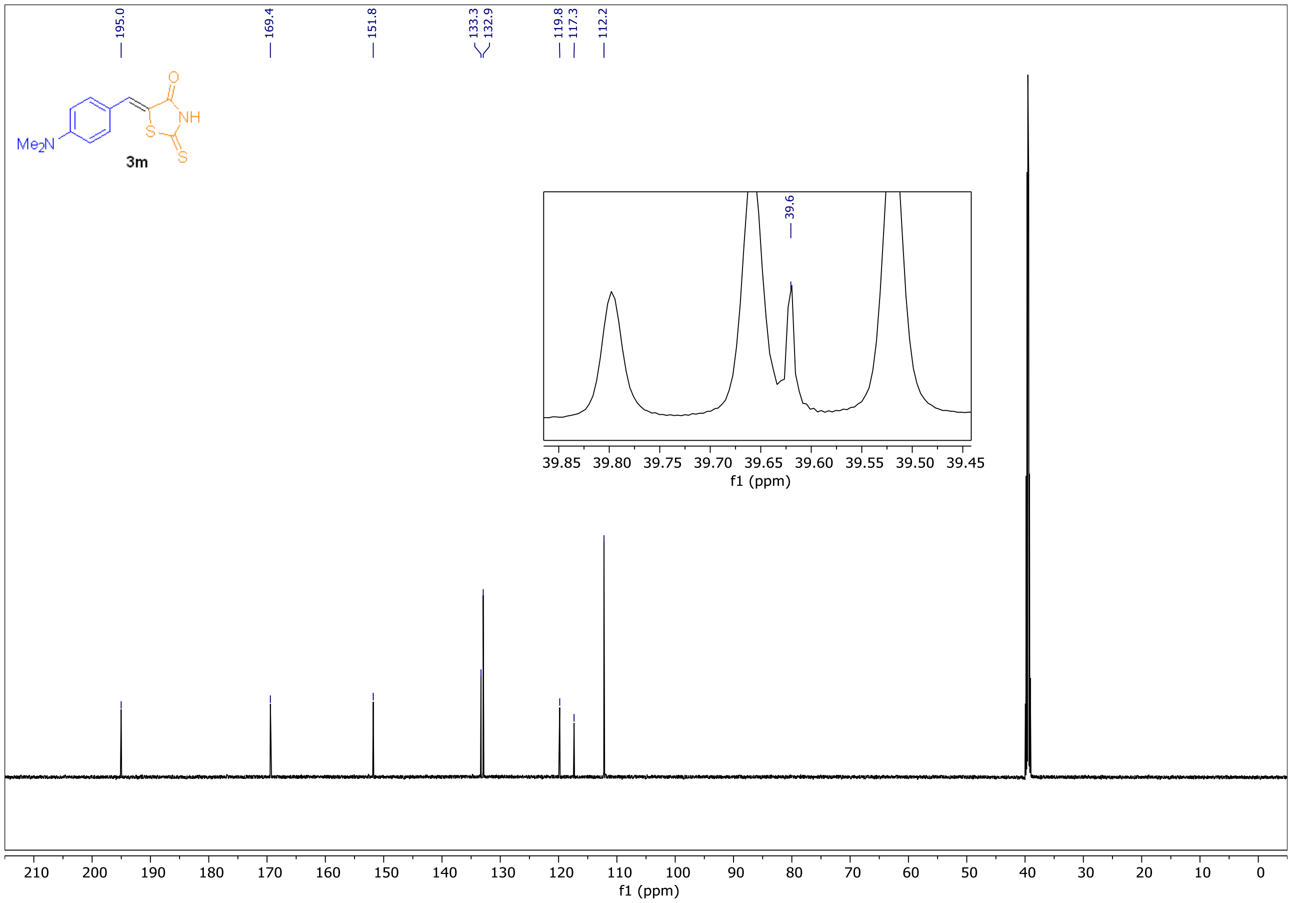

Figure S52. ^{13}C NMR spectrum (151 MHz, DMSO-d_6) of compound **3m**.

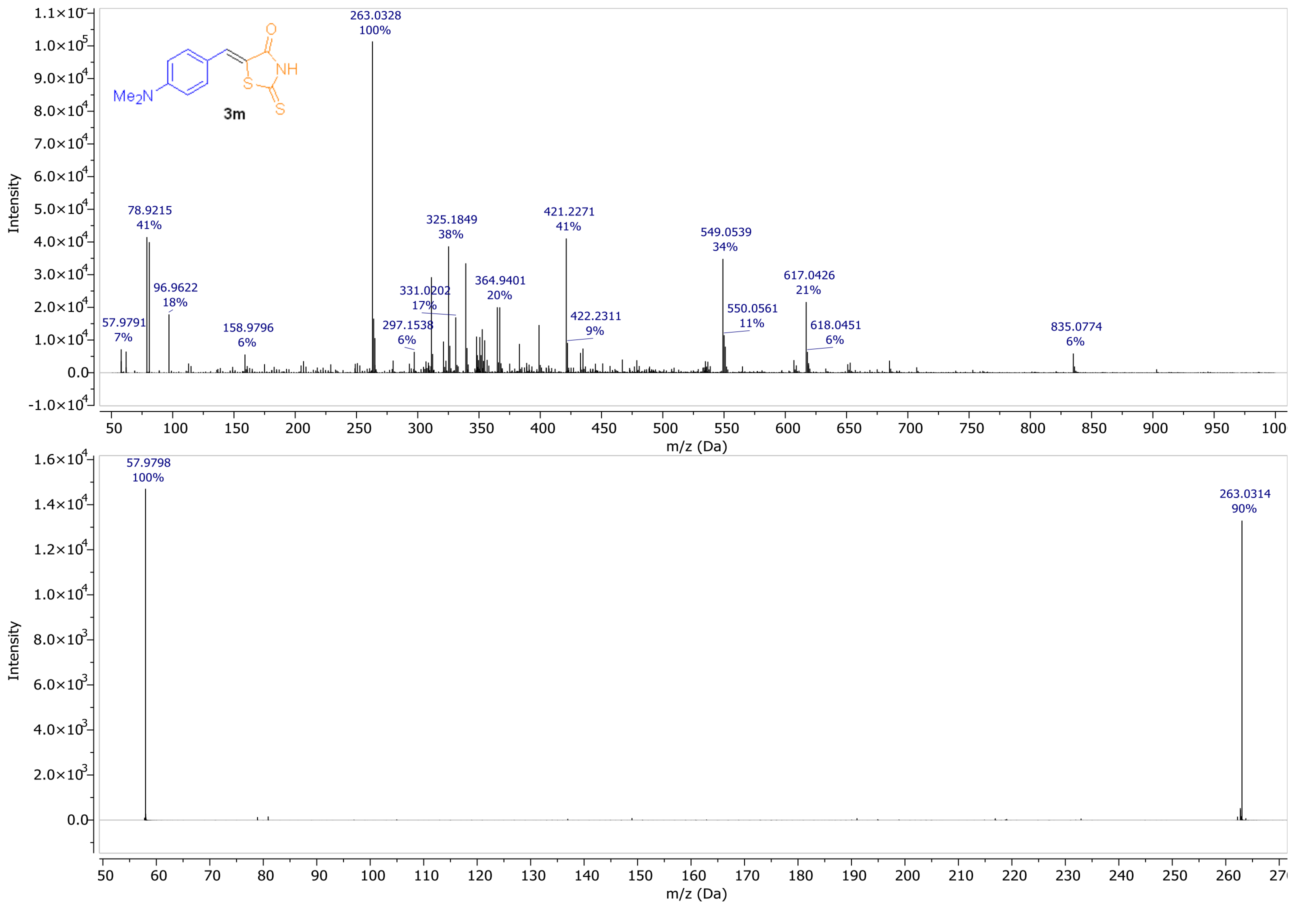

Figure S53. HRMS (ESI-QTOF) of compound **3m** and HRMS/MS for [M−H]$^-$.

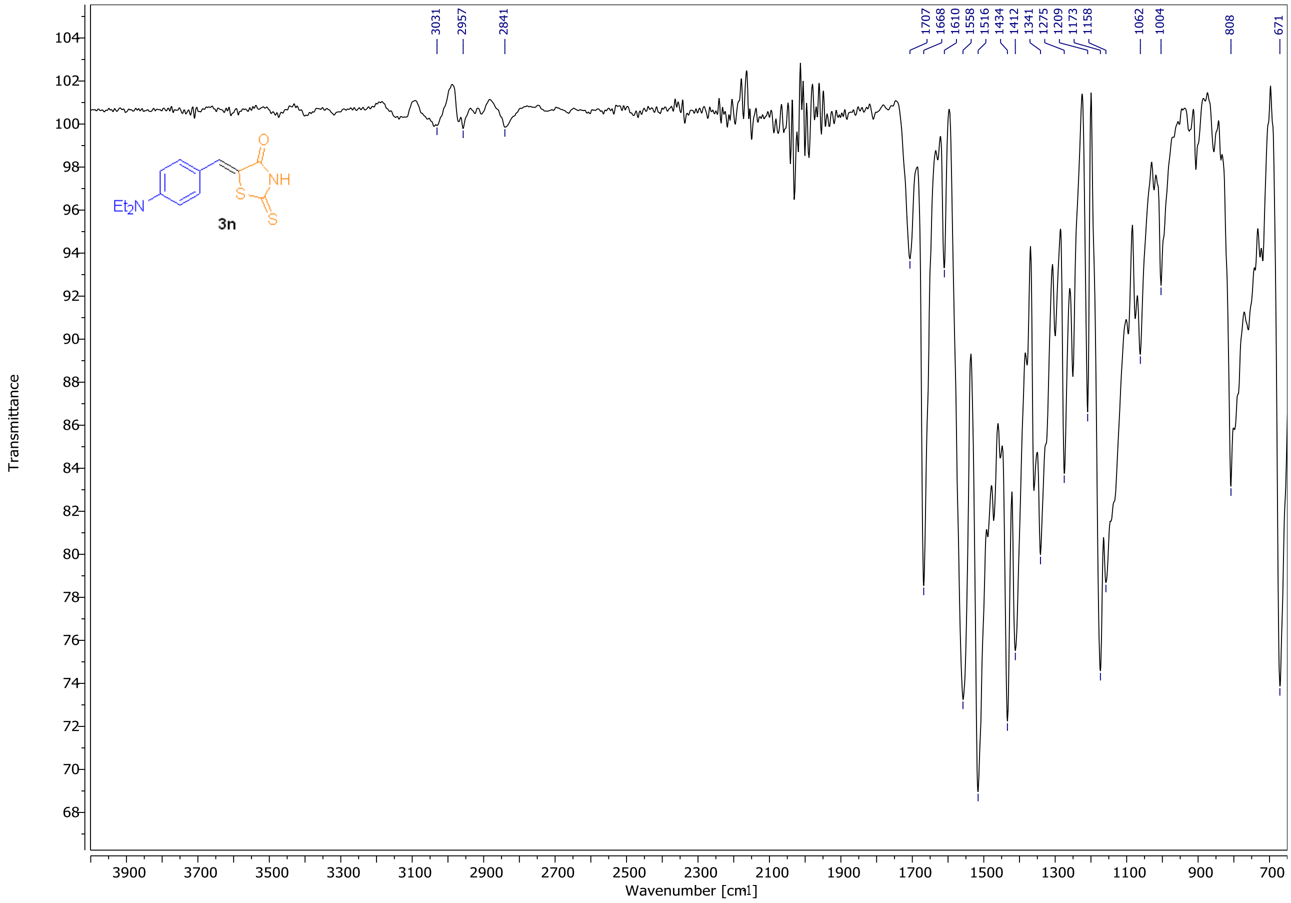

Figure S54. FTIR (ATR) of compound **3n**.

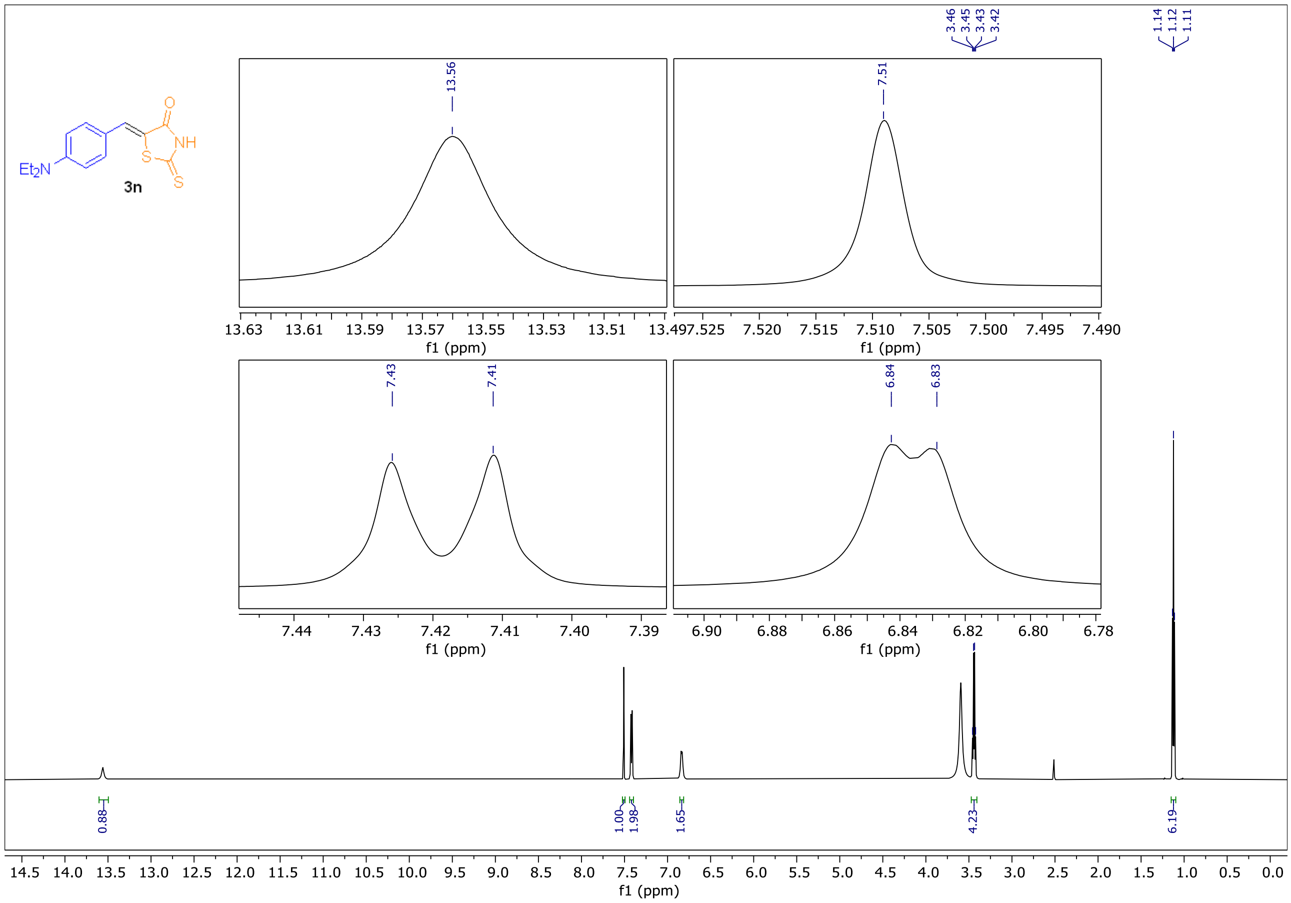

Figure S55. ^{1}H NMR spectrum (600 MHz, DMSO-d_6) of compound **3n**.

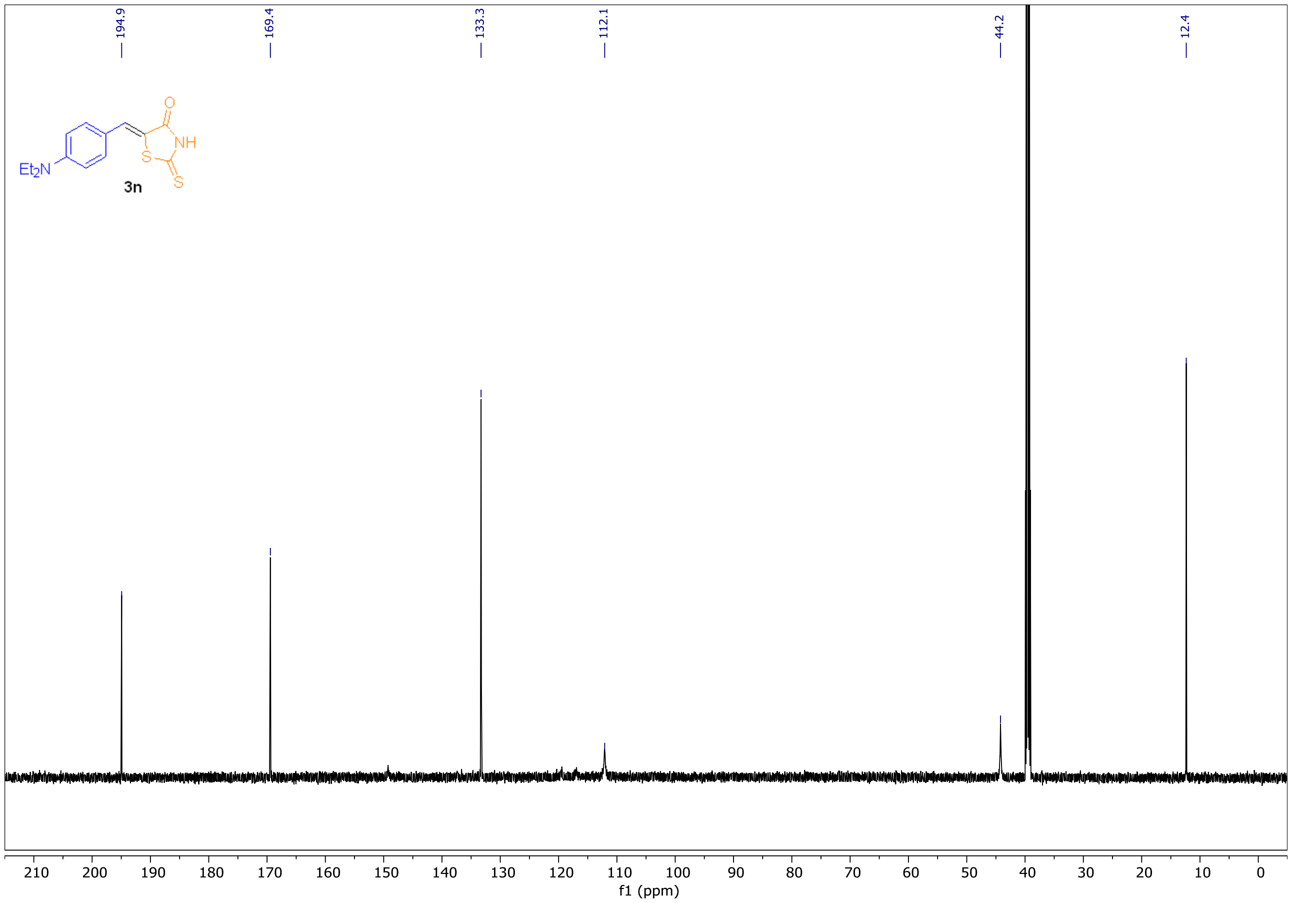

Figure S56. ^{13}C NMR spectrum (151 MHz, DMSO-*d*$_6$) of compound **3n**.

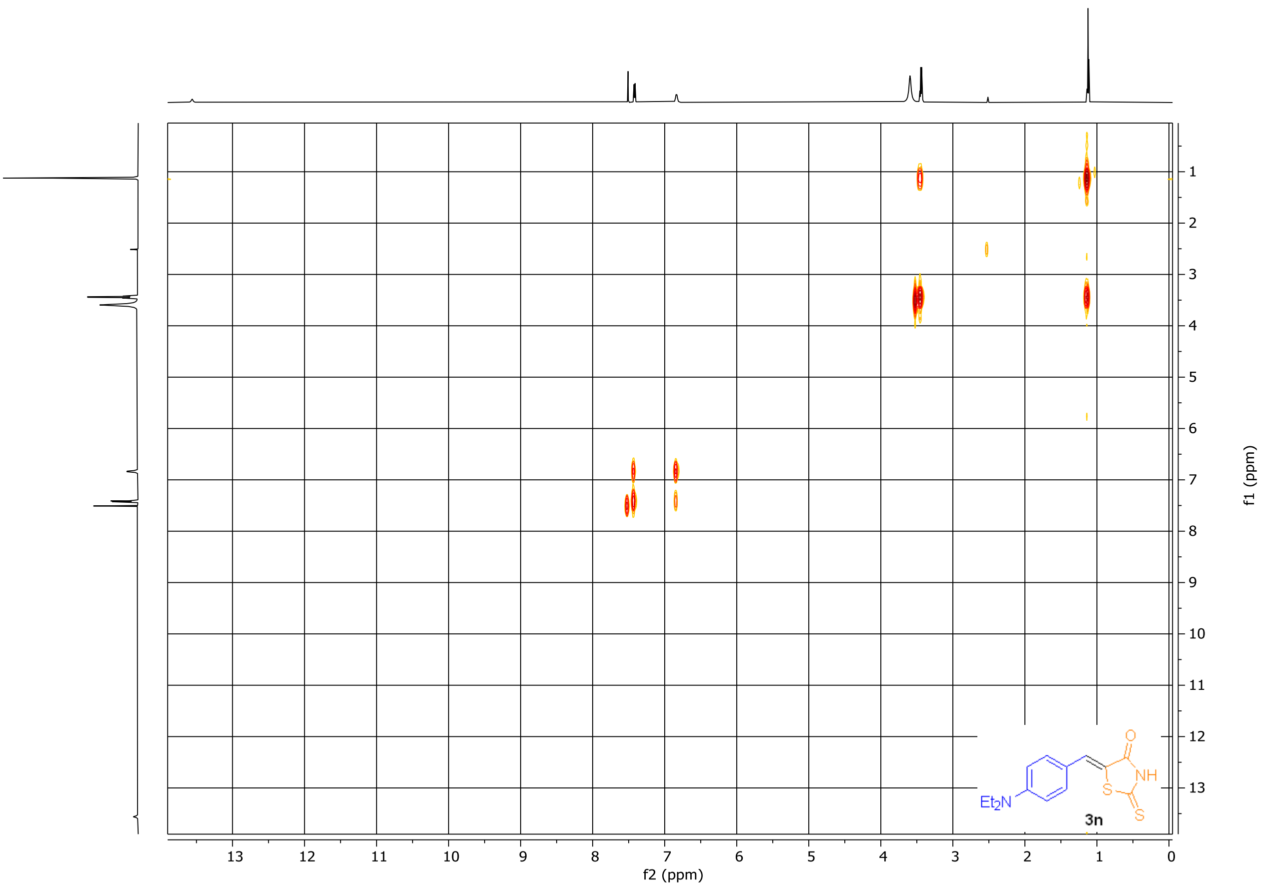

Figure S57. 2D COSY NMR spectrum (600 MHz, DMSO-*d*$_6$) of compound **3n**.

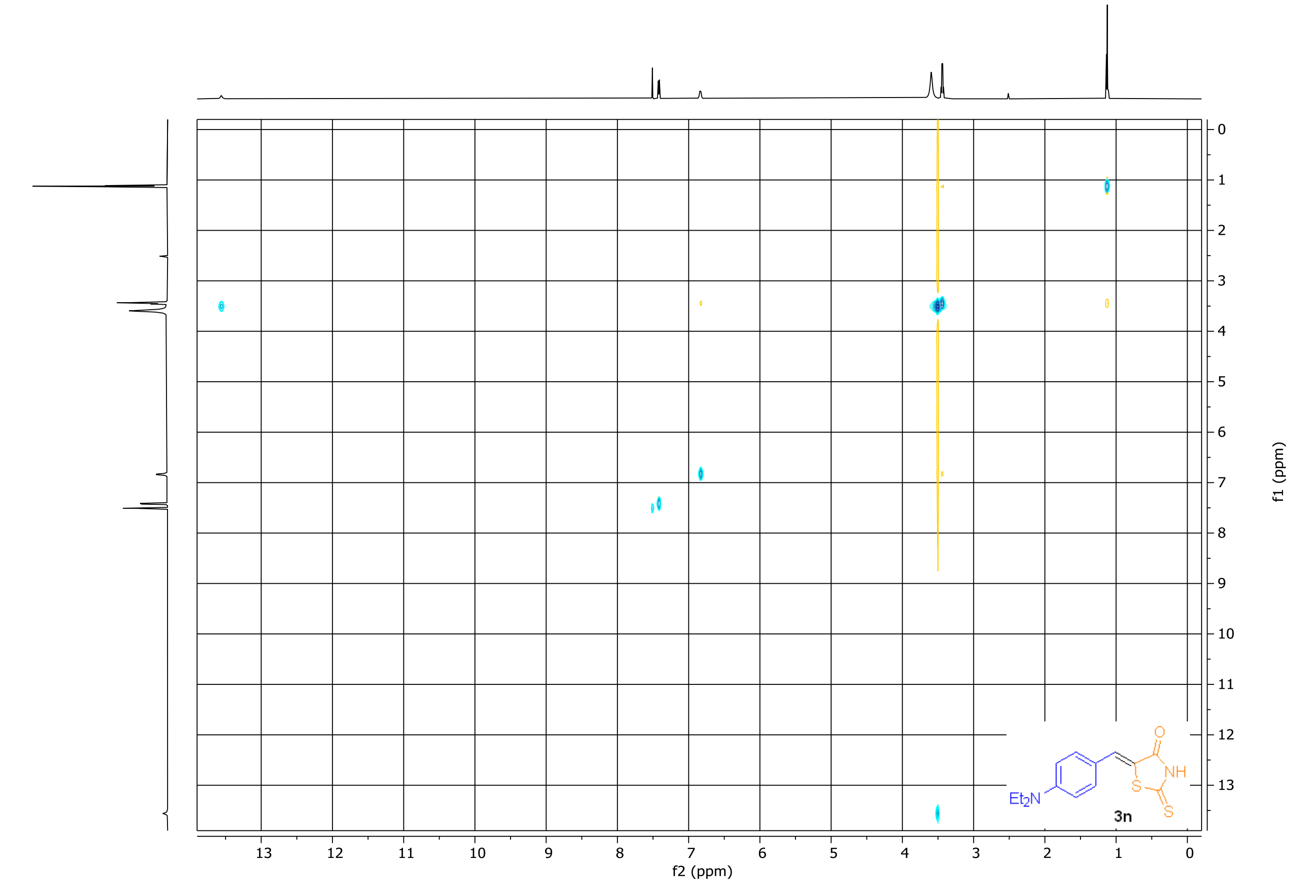

Figure S58. 2D NOESY NMR spectrum (600 MHz, DMSO-d_6) of compound **3n**.

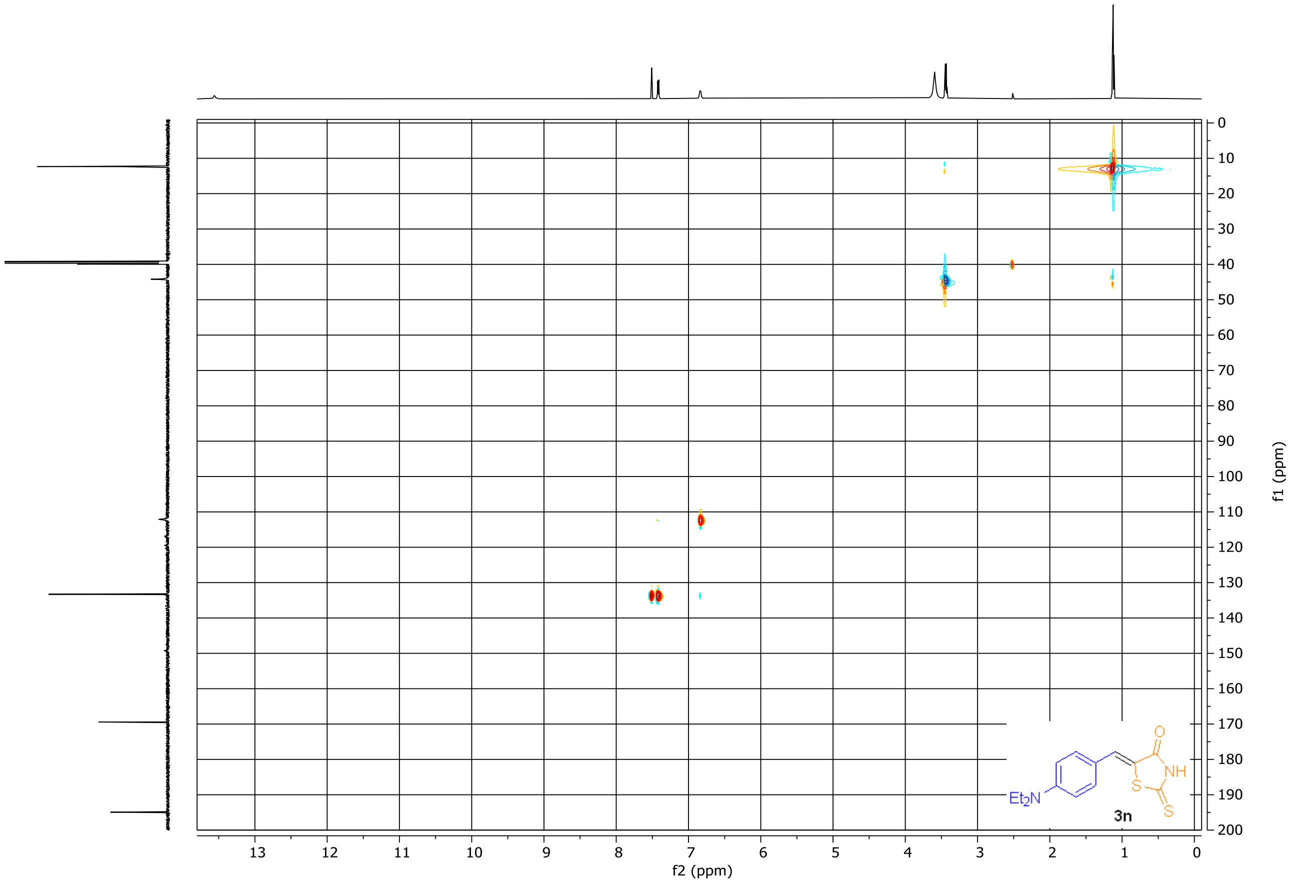

Figure S59. 2D HSQC NMR spectrum (600 MHz, DMSO-d_6) of compound **3n**.

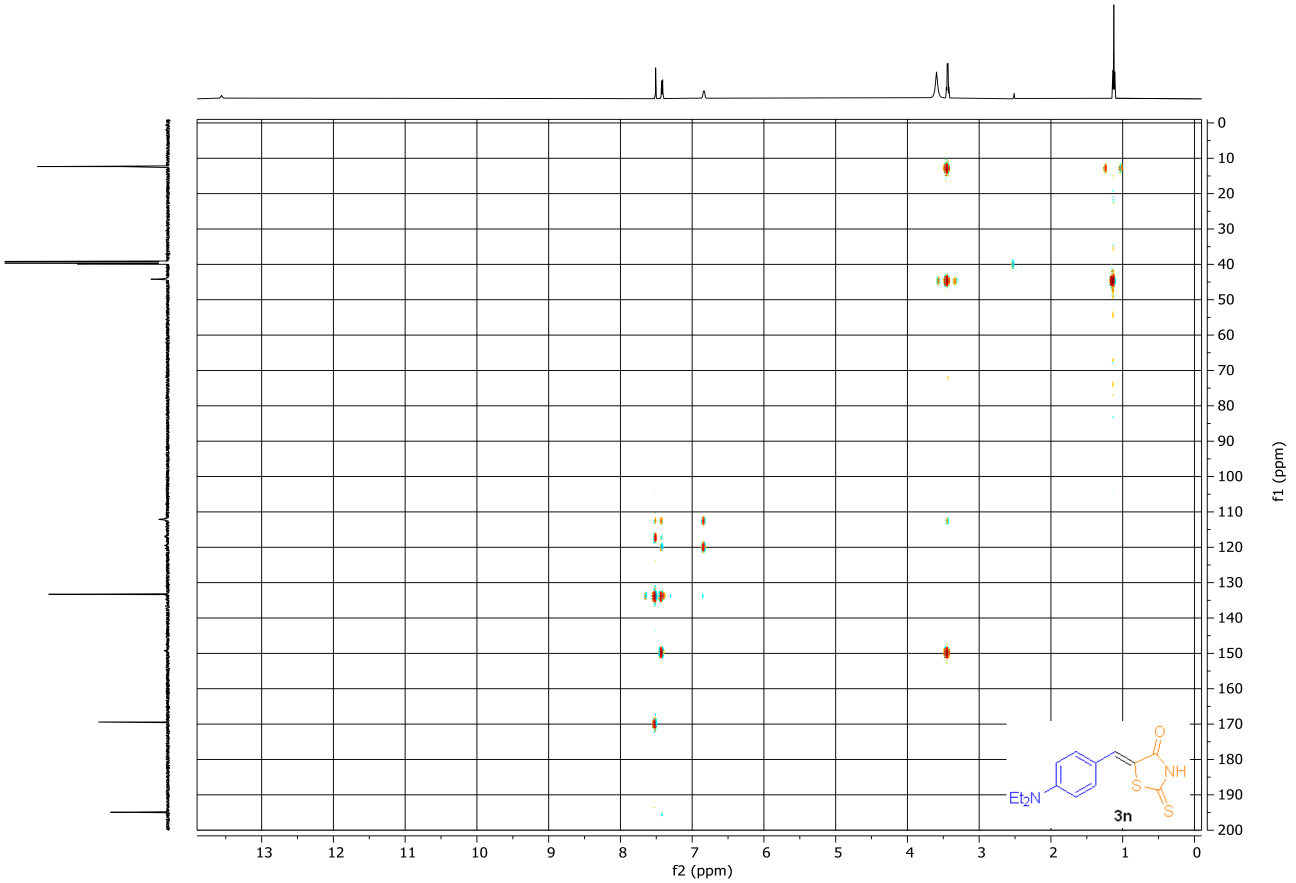

Figure S60. 2D HMBC NMR spectrum (600 MHz, DMSO-d_6) of compound **3n**.

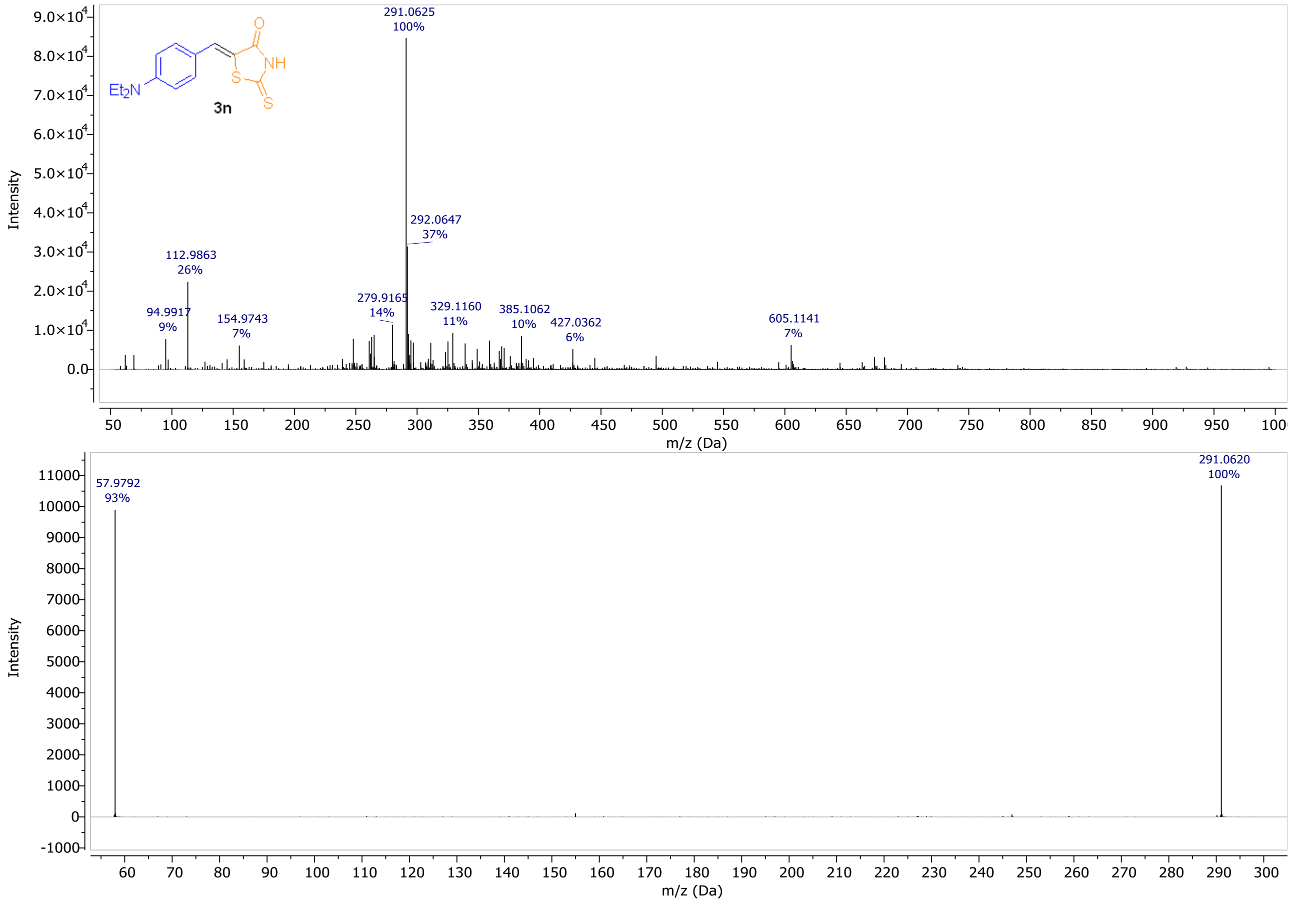

Figure S61. HRMS (ESI-QTOF) of compound **3n** and HRMS/MS for $[M-H]^-$.

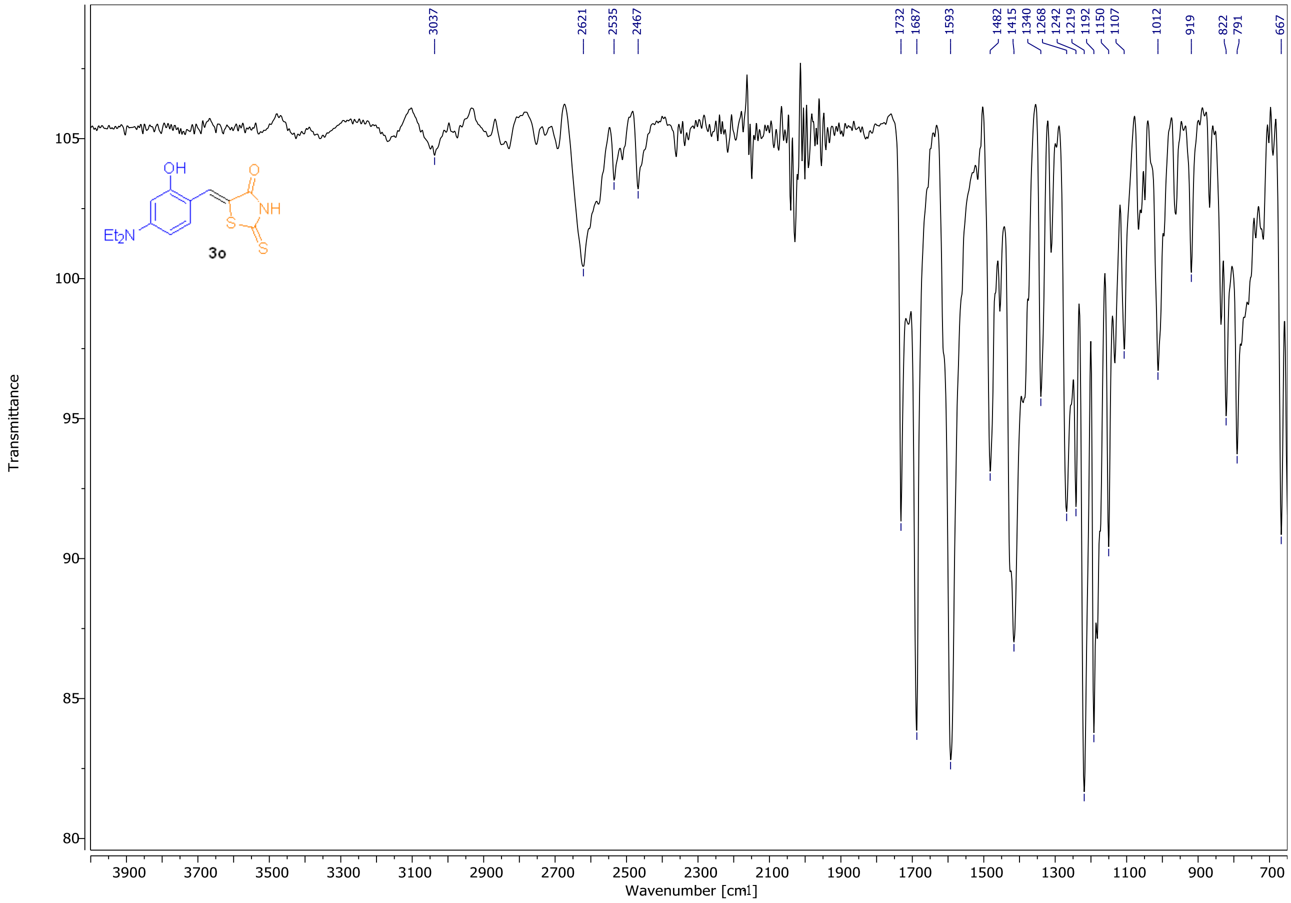

Figure S62. FTIR (ATR) of compound **3o**.

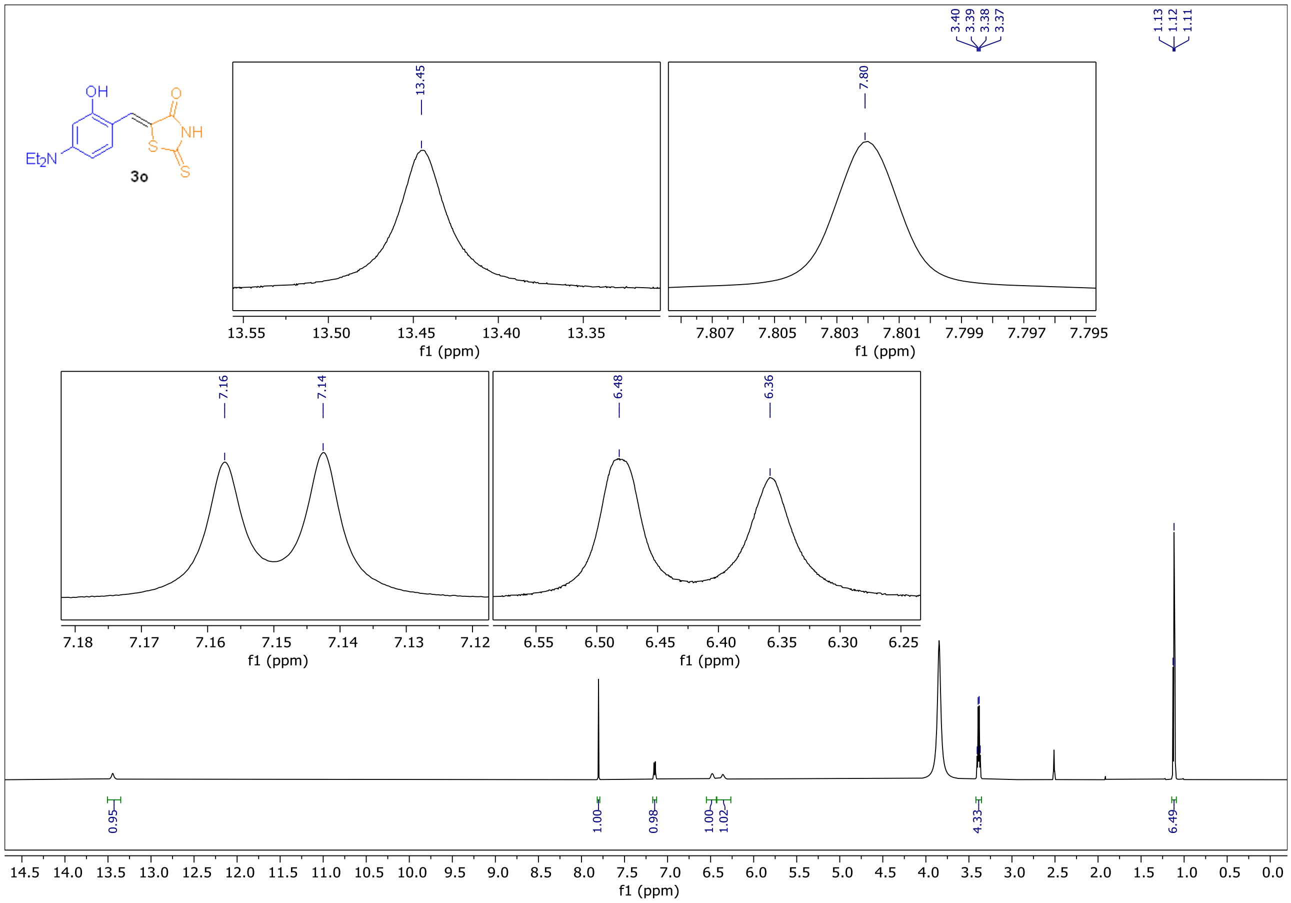

Figure S63. ^{1}H NMR spectrum (600 MHz, DMSO-d_6) of compound **3o**.

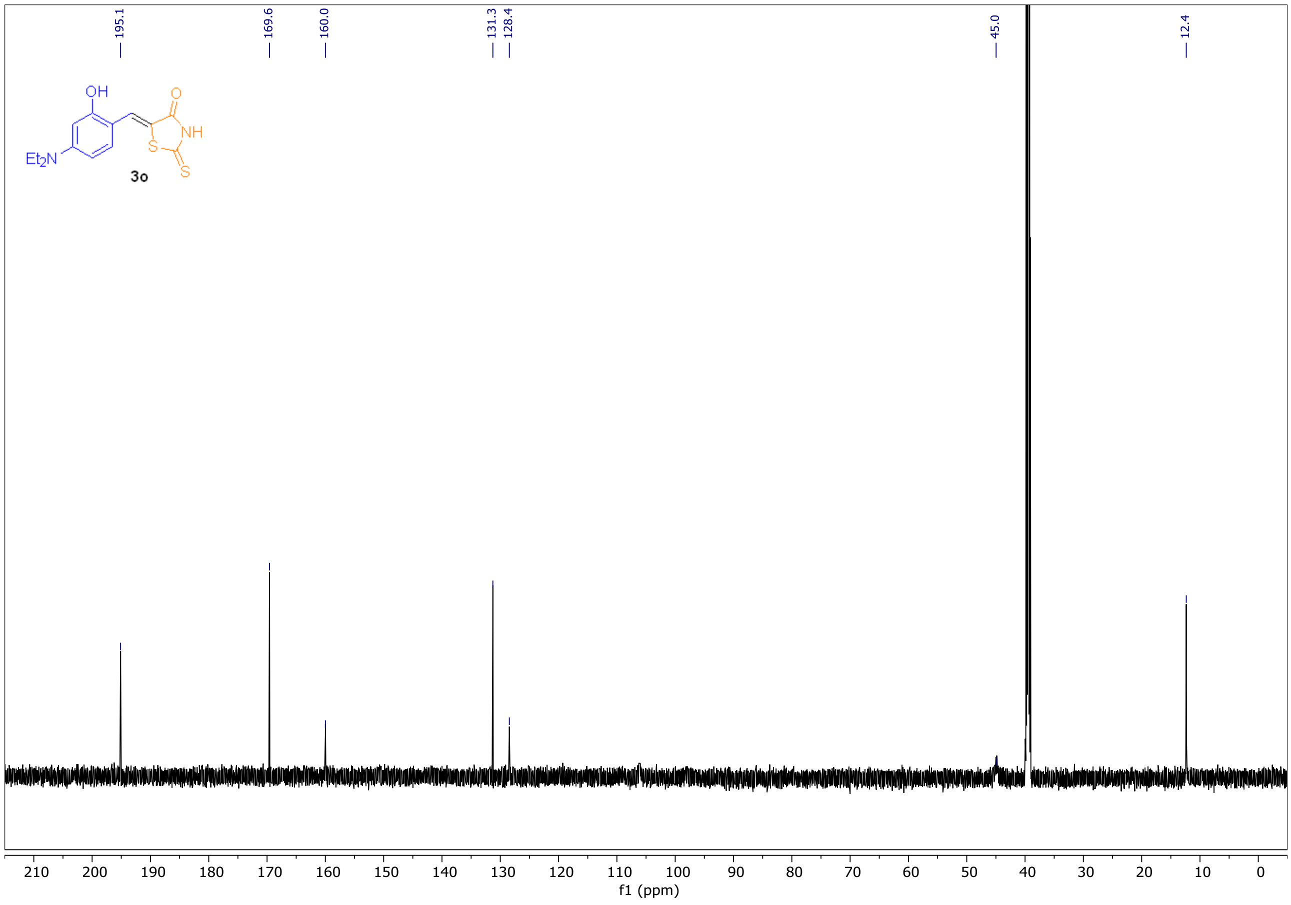

Figure S64. ^{13}C NMR spectrum (151 MHz, DMSO-*d*$_6$) of compound **3o**.

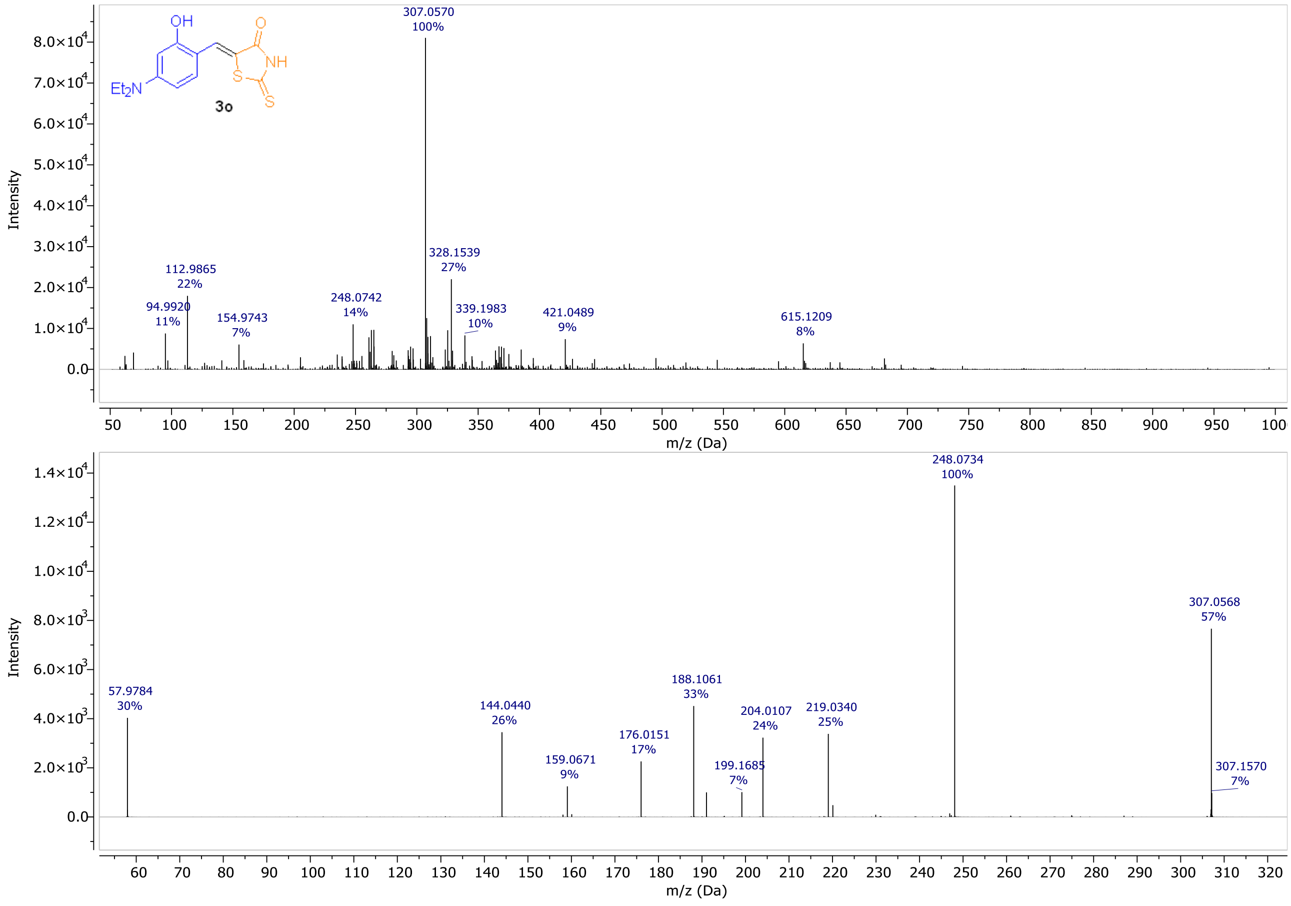

Figure S65. HRMS (ESI-QTOF) of compound **3o** and HRMS/MS for $[M-H]^-$.

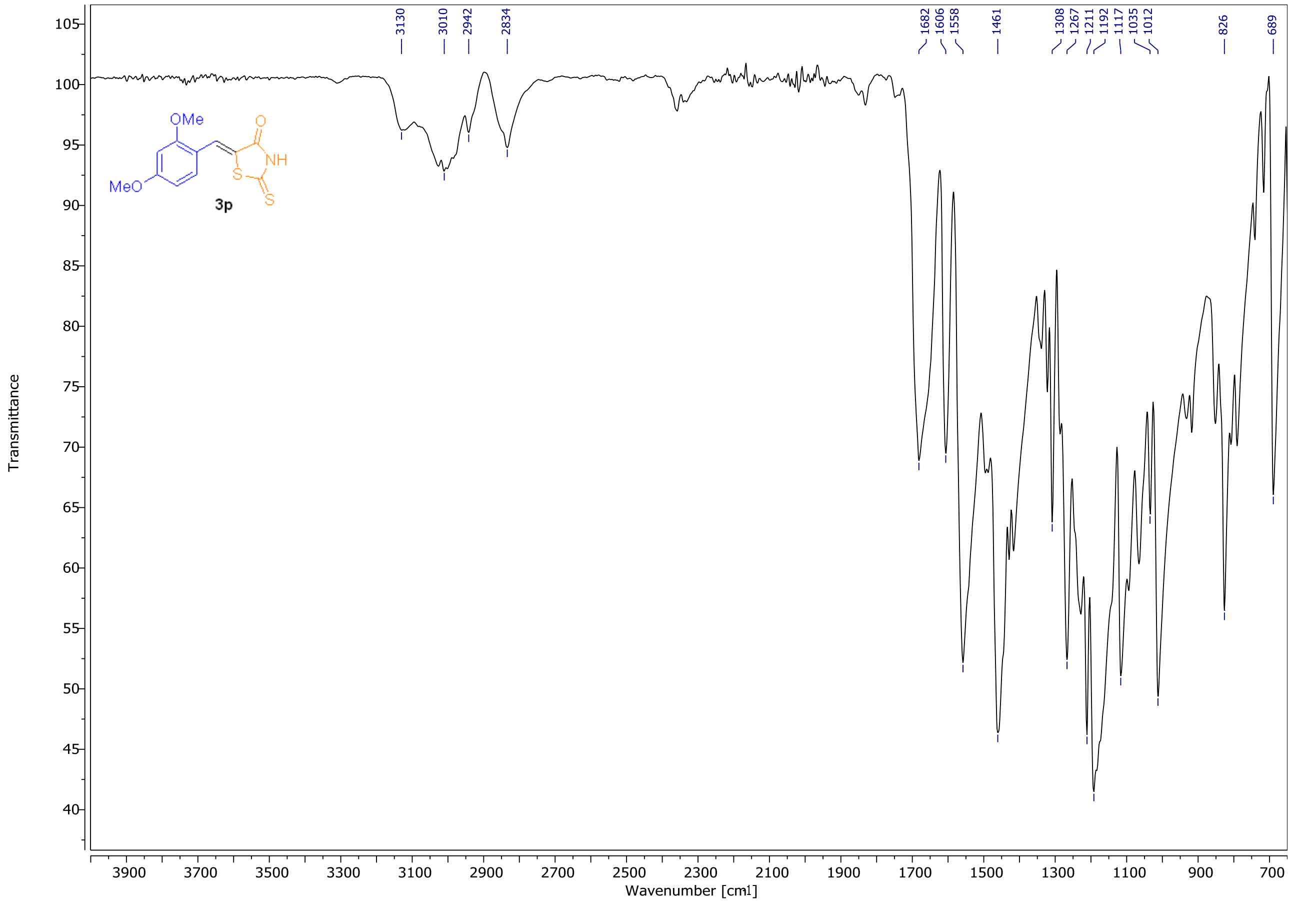

Figure S66. FTIR (ATR) of compound **3p**.

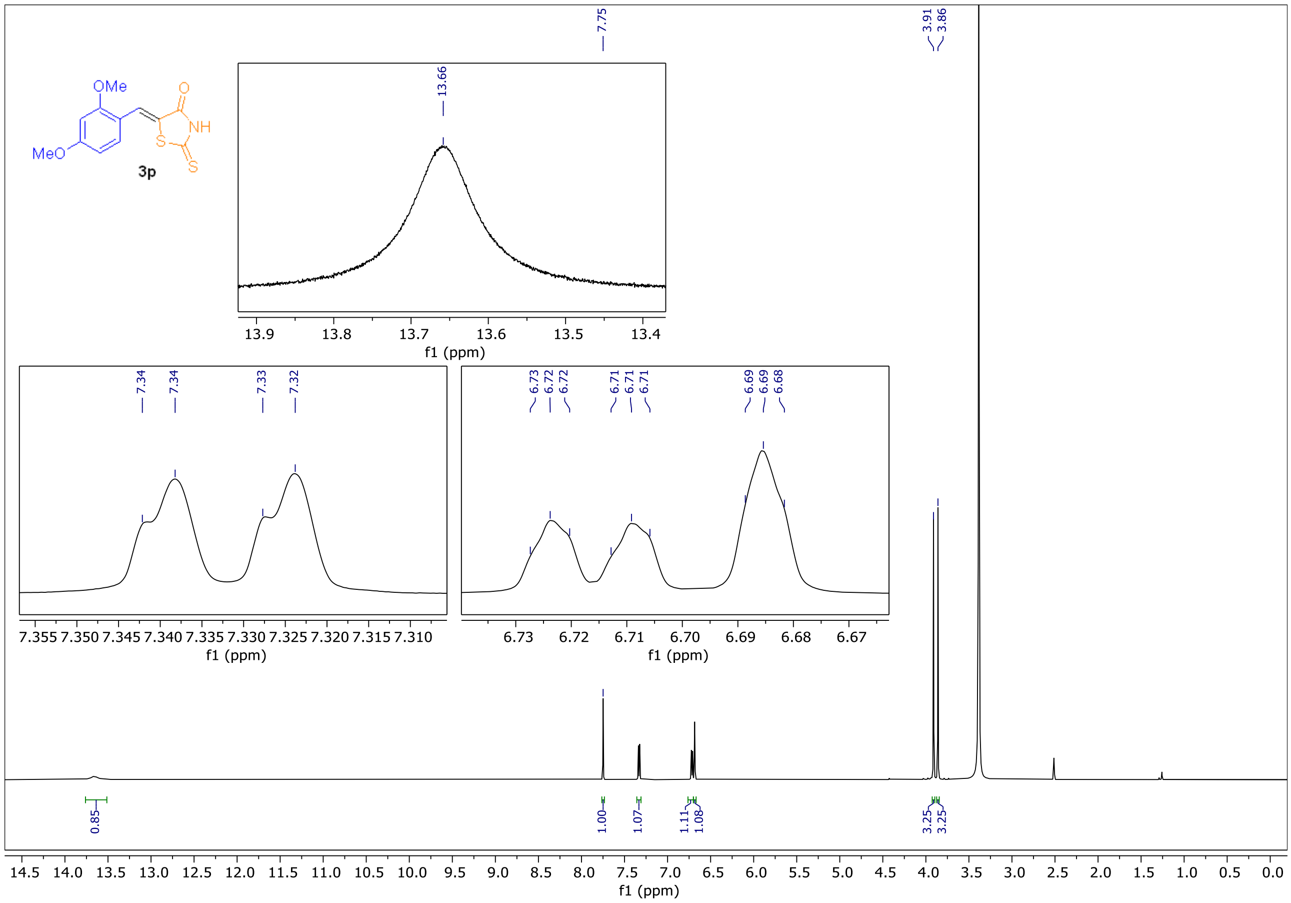

Figure S67. ^{1}H NMR spectrum (600 MHz, DMSO-d_6) of compound **3p**.

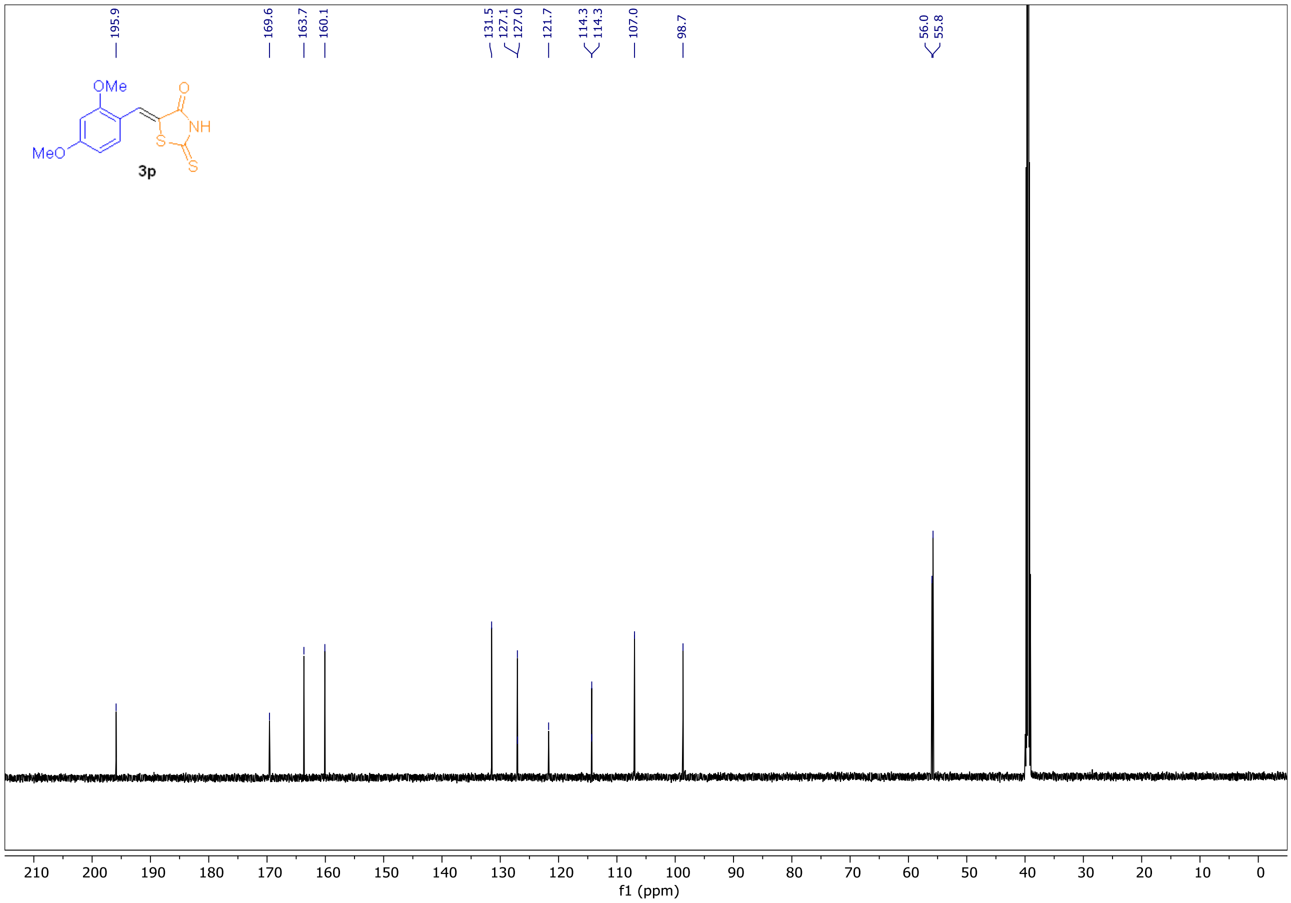

Figure S68. ^{13}C NMR spectrum (151 MHz, DMSO-d_6) of compound **3p**.

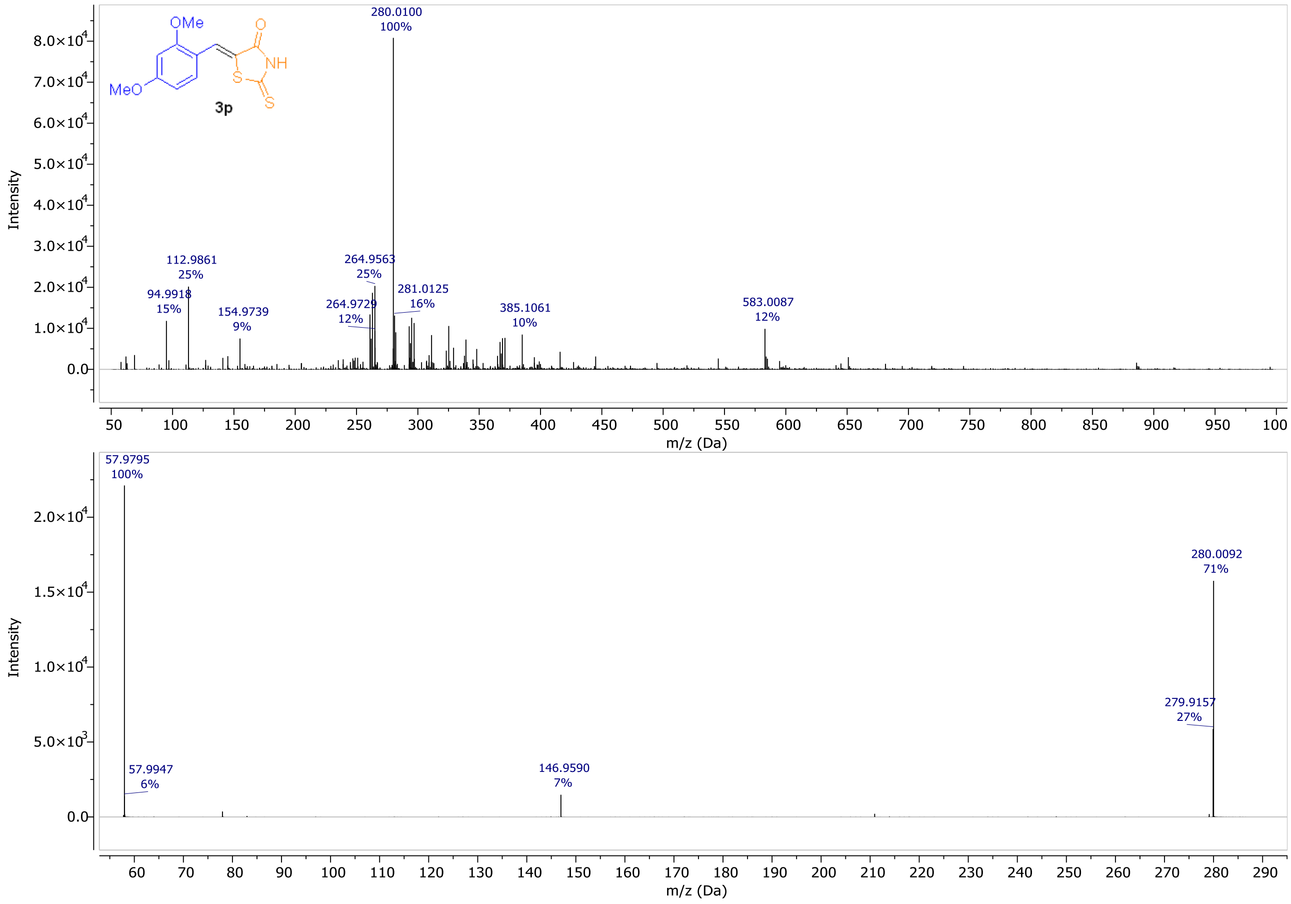

Figure S69. HRMS (ESI-QTOF) of compound **3p** and HRMS/MS for [M−H]⁻.

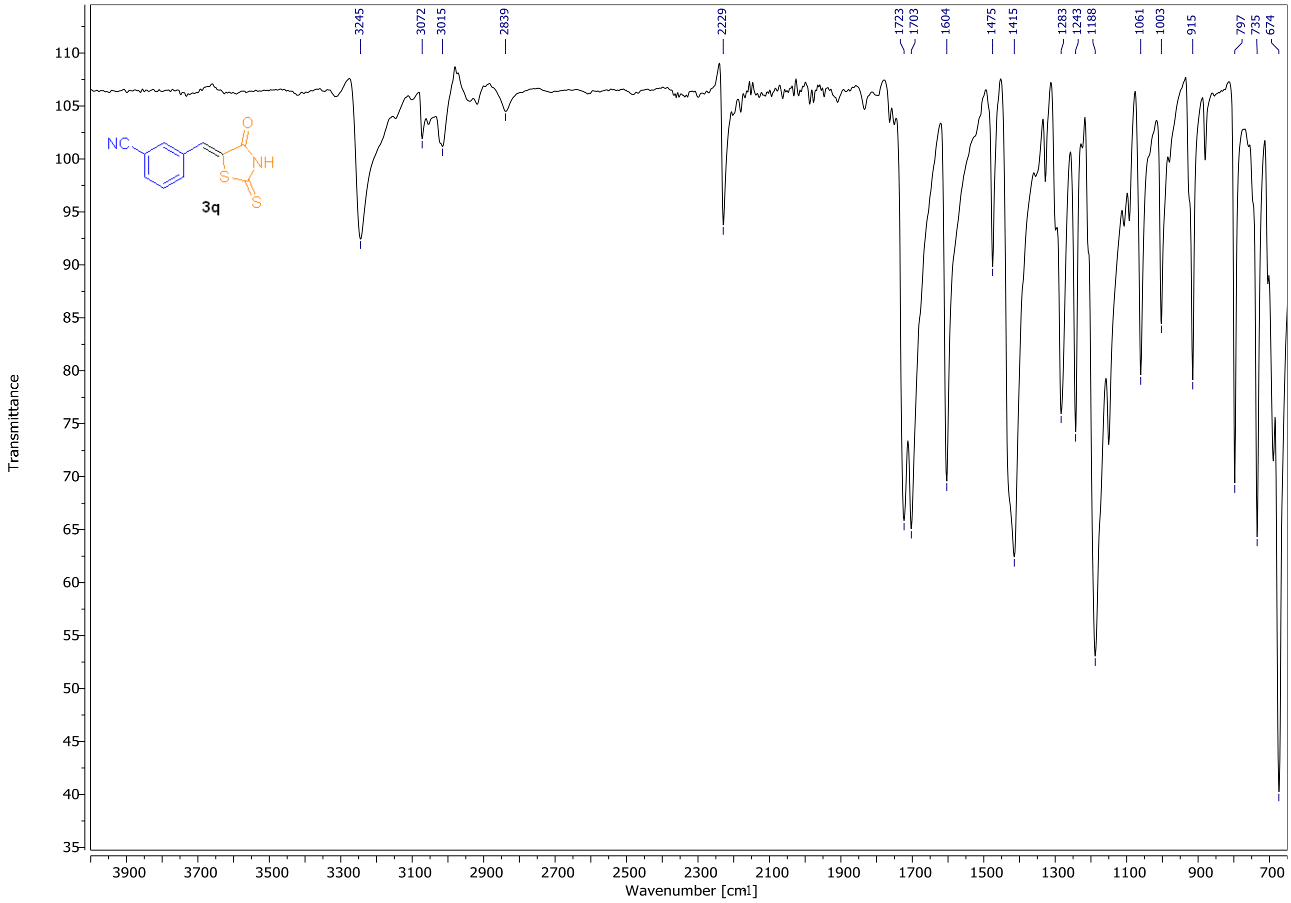

Figure S70. FTIR (ATR) of compound **3q**.

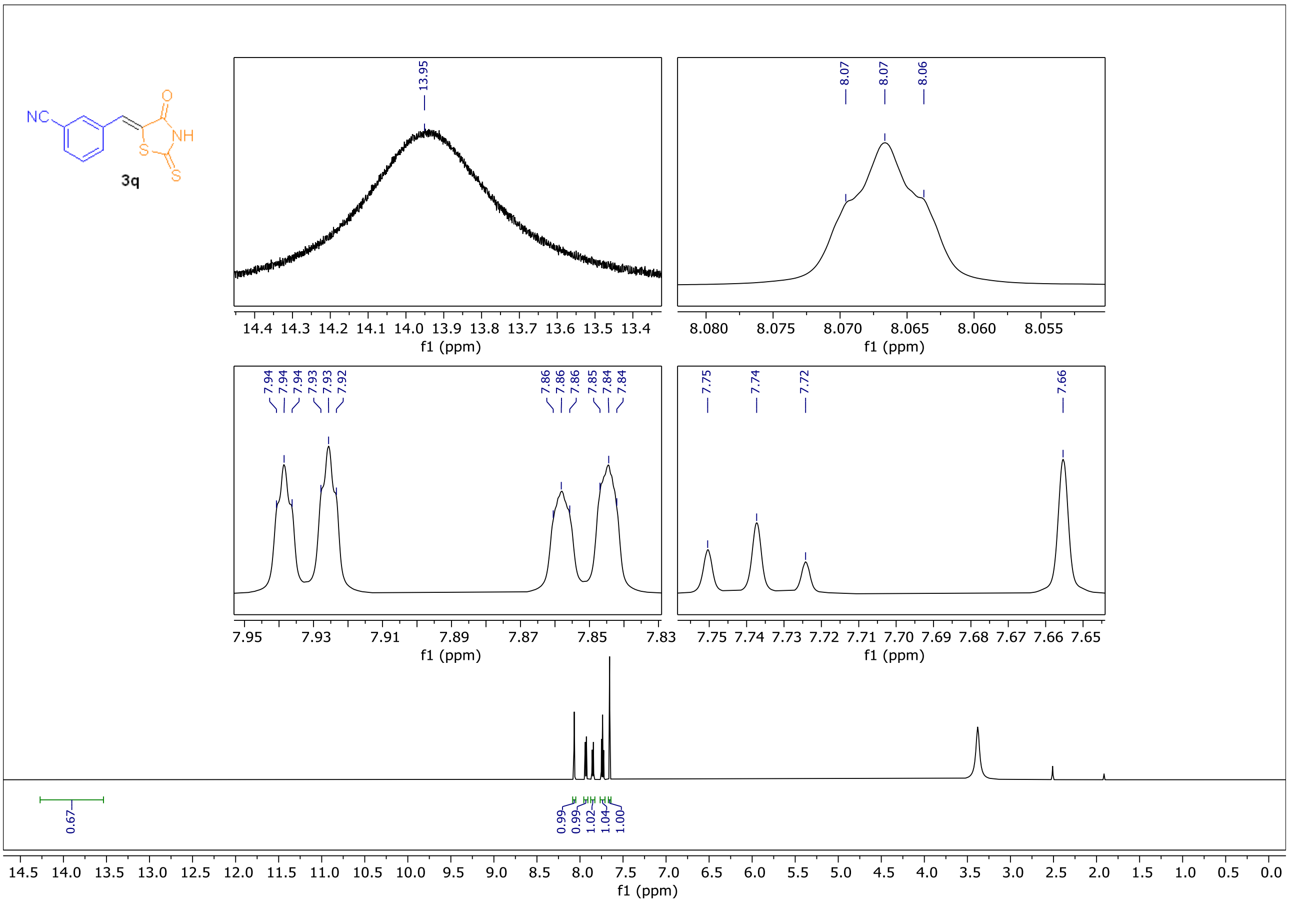

Figure S71. ^{1}H NMR spectrum (600 MHz, DMSO-d_6) of compound **3q**.

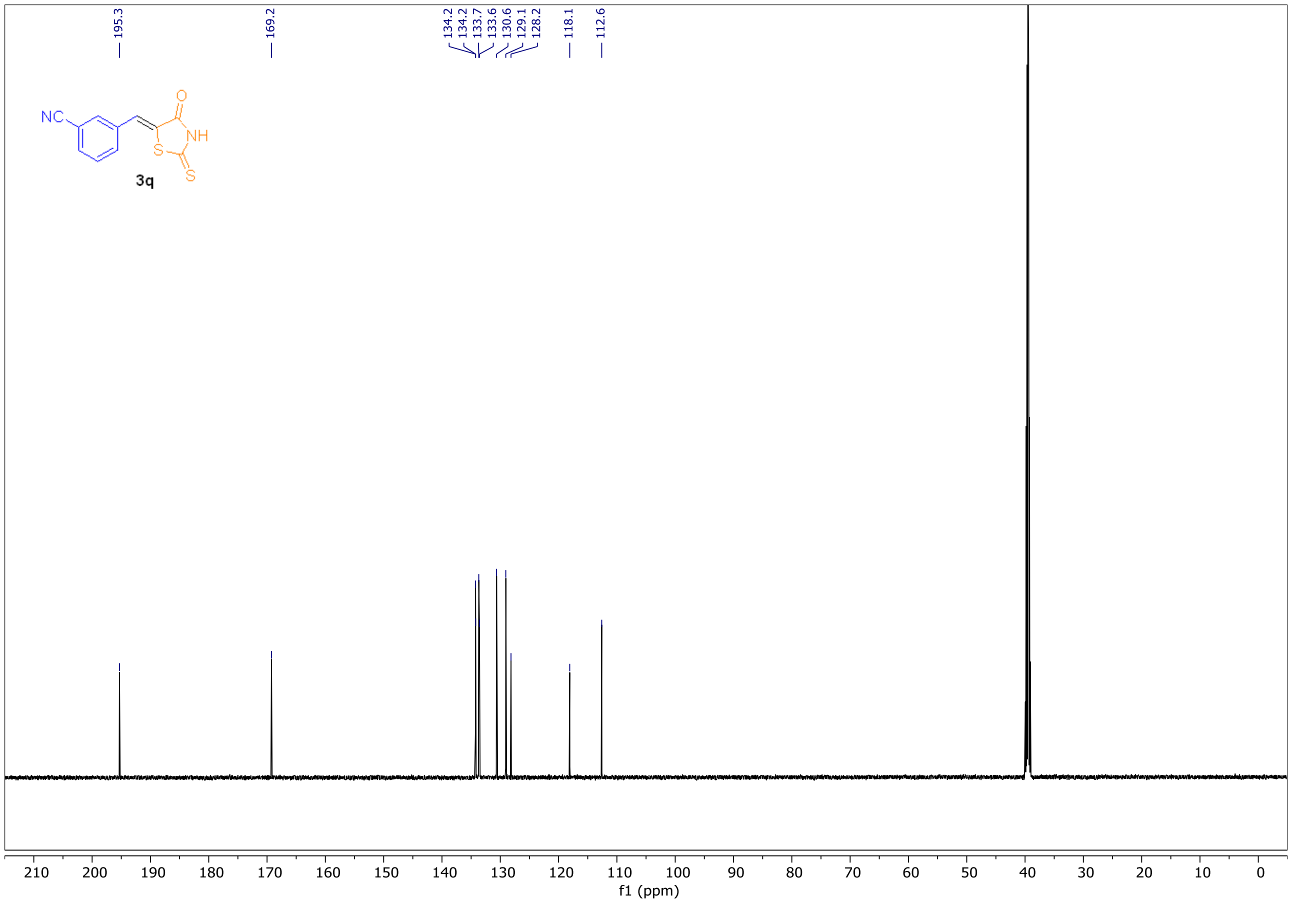

Figure S72. ^{13}C NMR spectrum (151 MHz, DMSO-d_6) of compound **3q**.

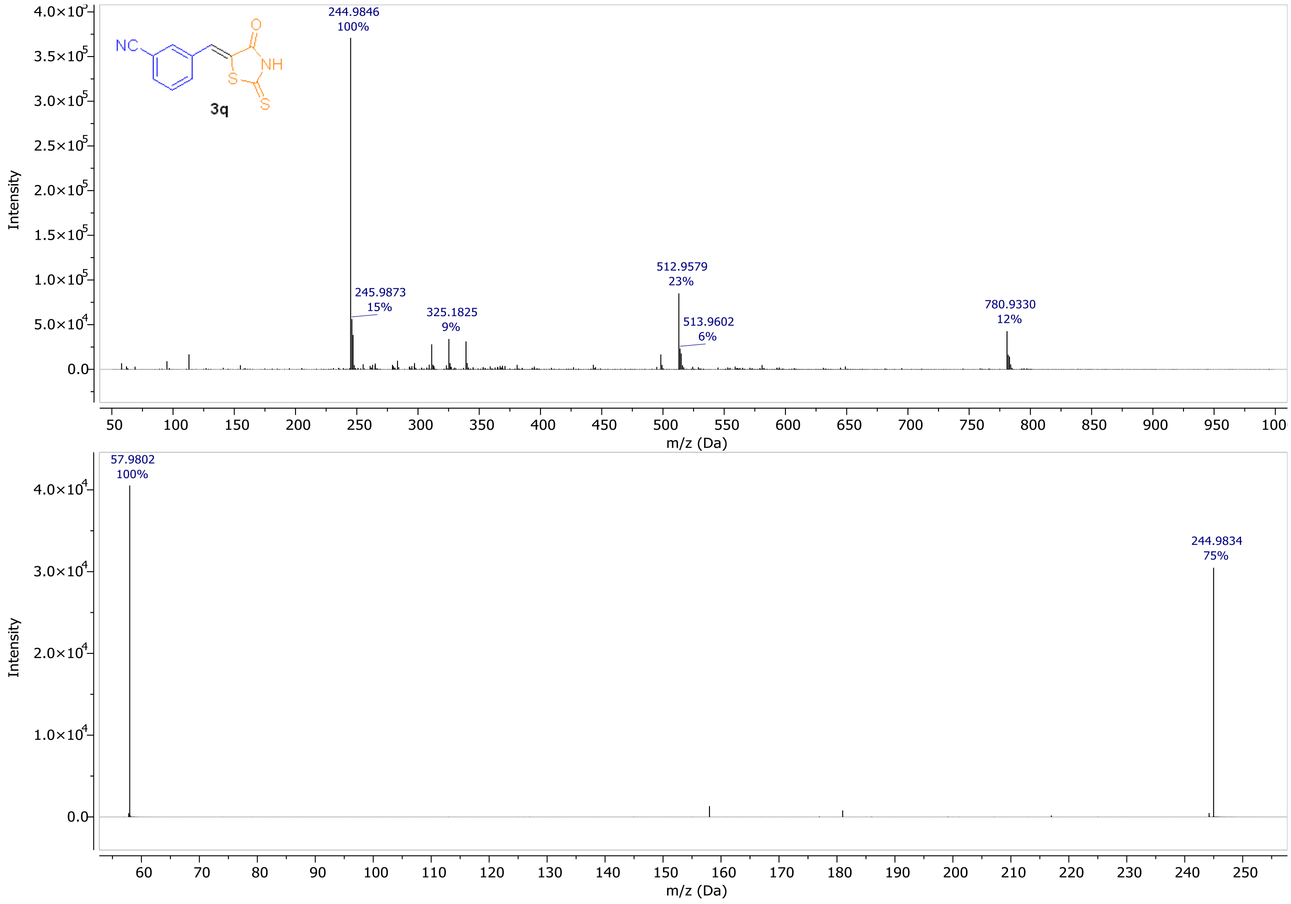

Figure S73. HRMS (ESI-QTOF) of compound **3q** and HRMS/MS for [M−H]⁻.

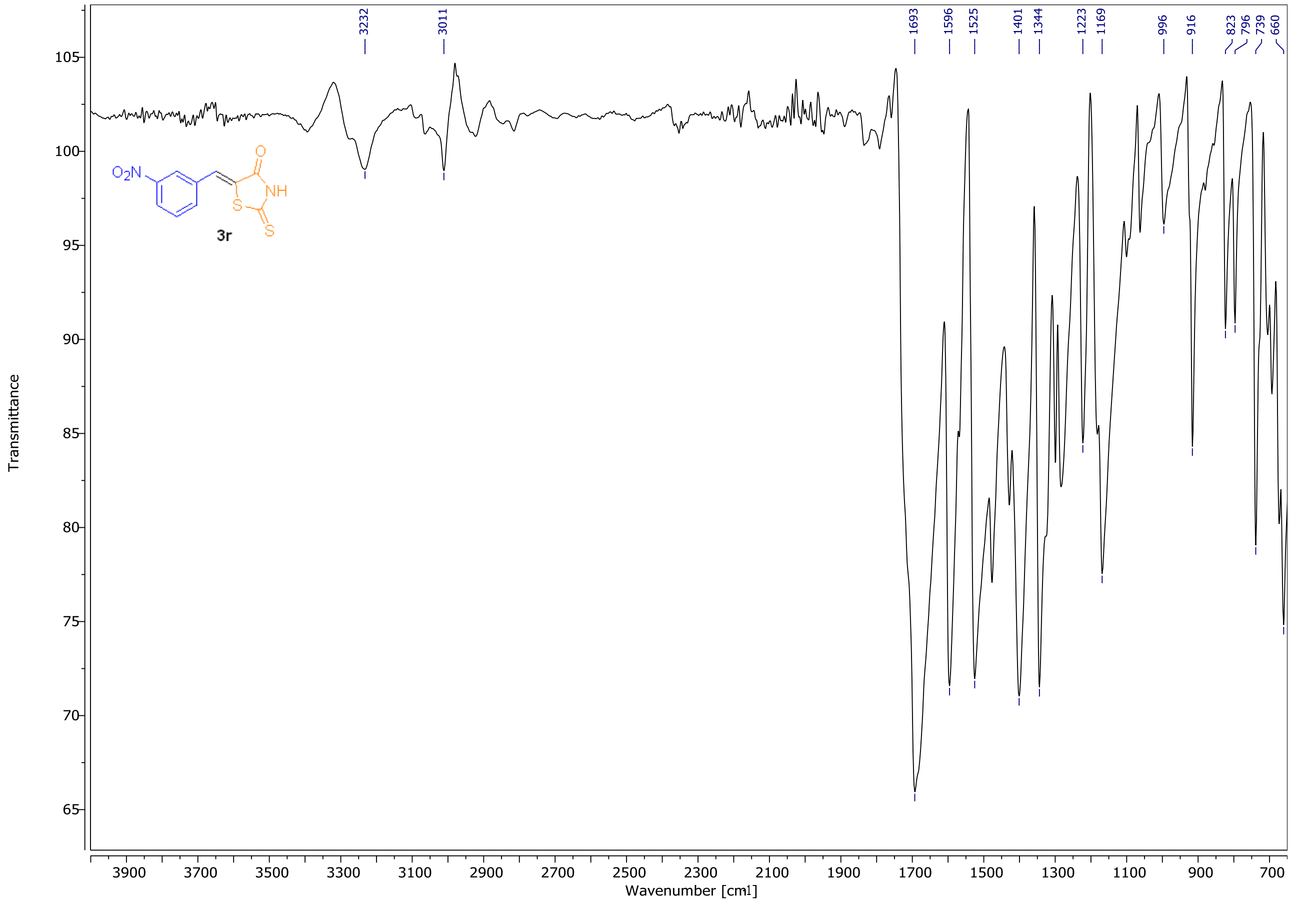

Figure S74. FTIR (ATR) of compound **3r**.

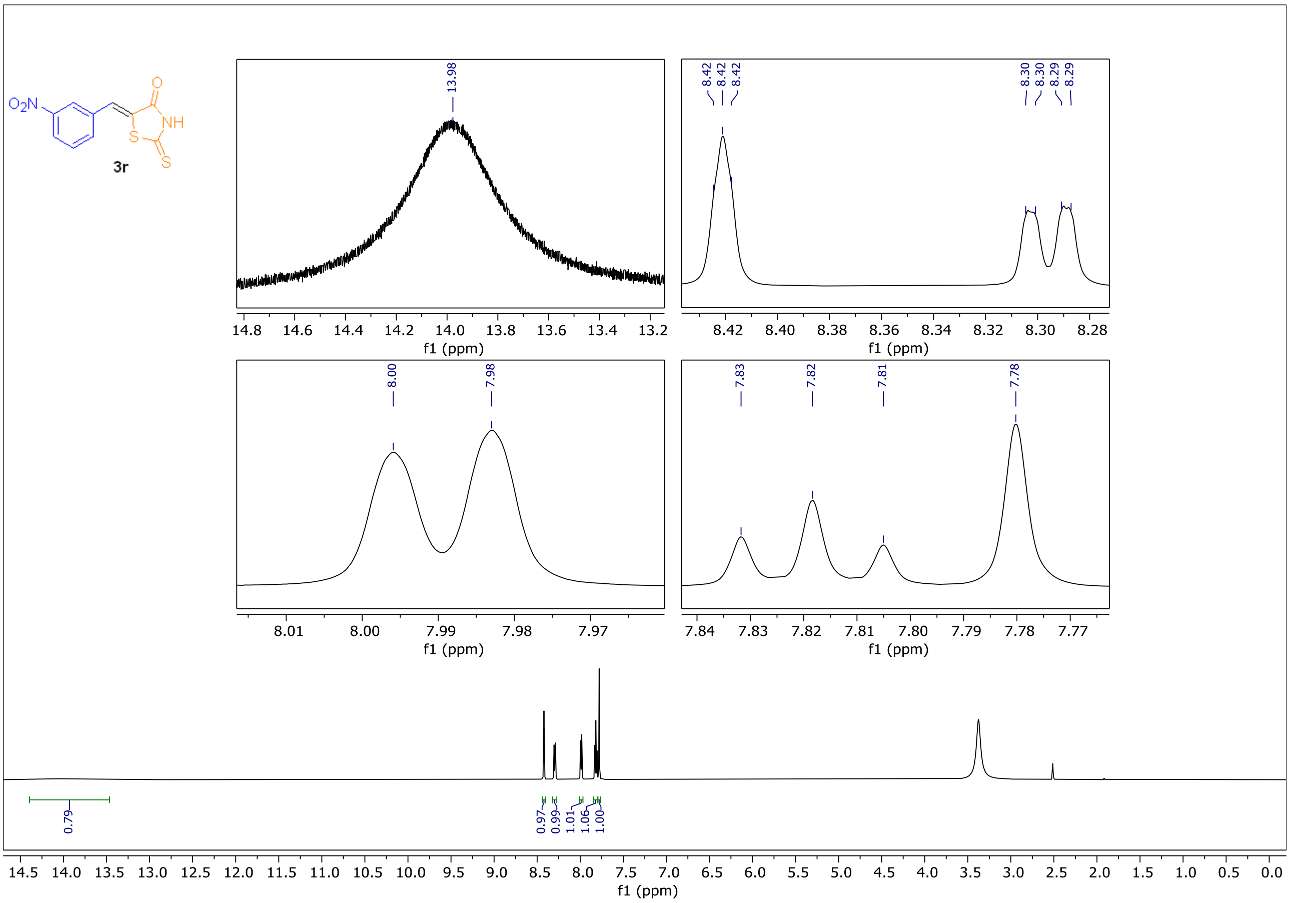

Figure S75. ^{1}H NMR spectrum (600 MHz, DMSO-d_6) of compound **3r**.

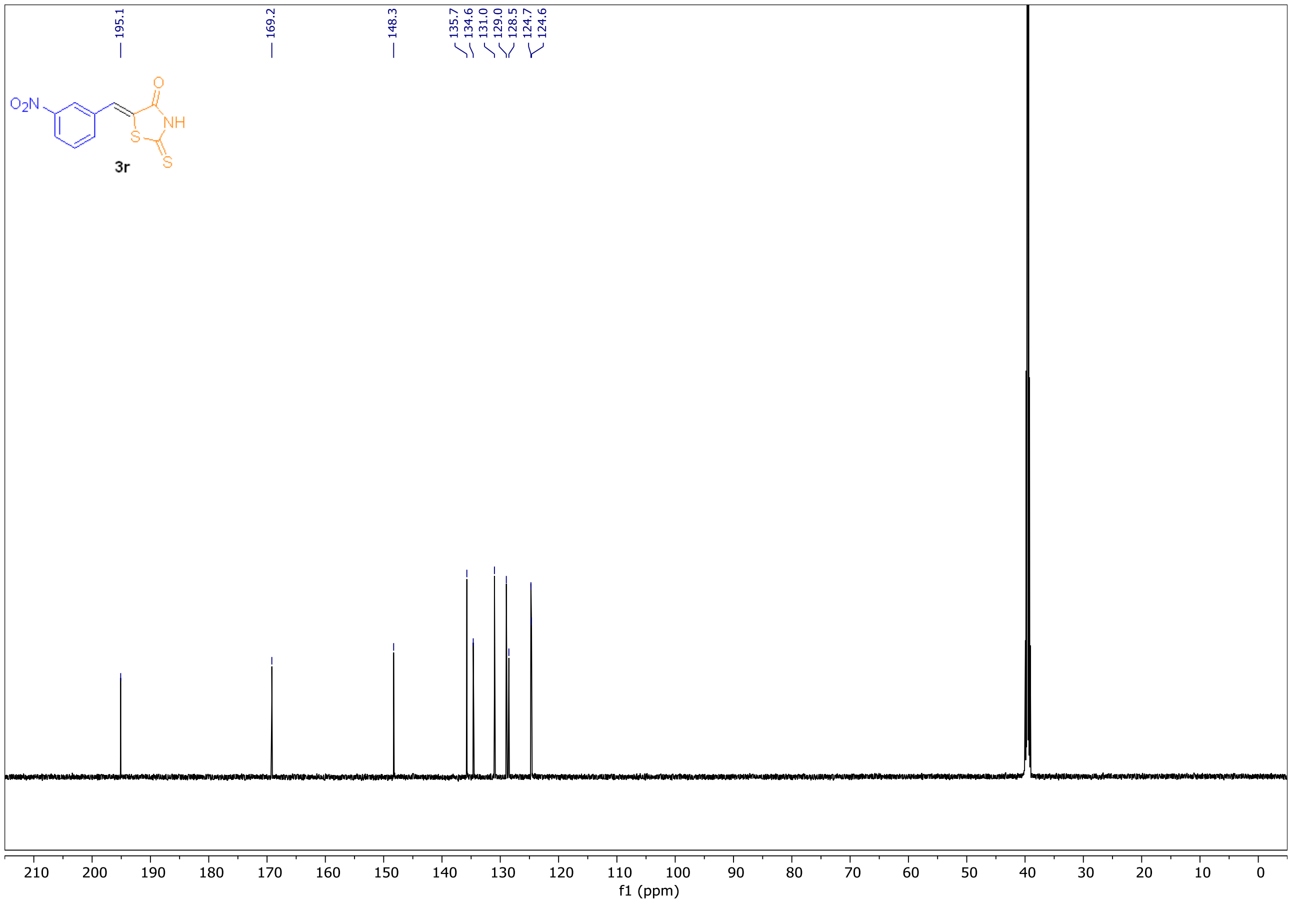

Figure S76. ^{13}C NMR spectrum (151 MHz, DMSO-d_6) of compound **3r**.

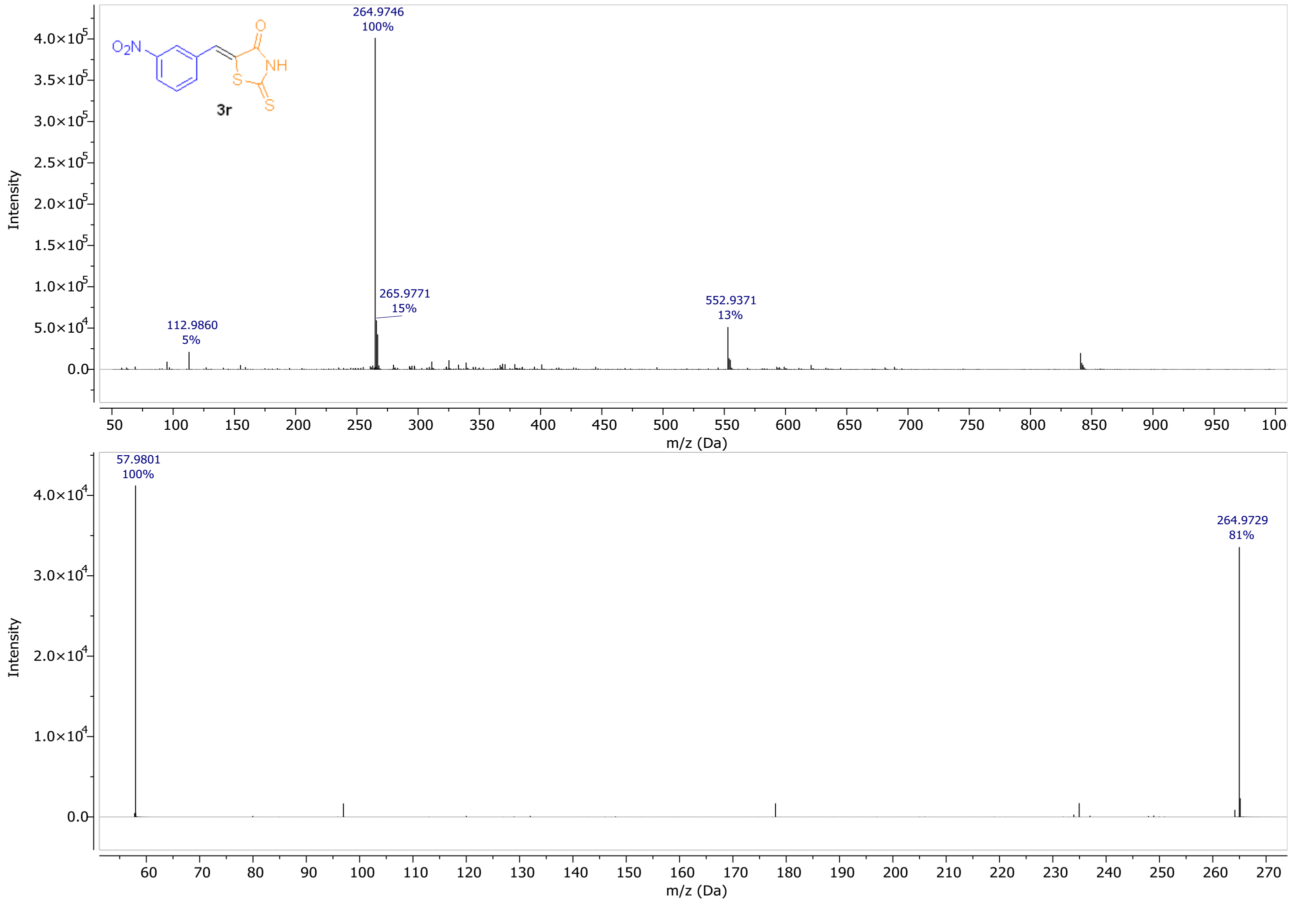

Figure S77. HRMS (ESI-QTOF) of compound **3r** and HRMS/MS for $[M-H]^-$.

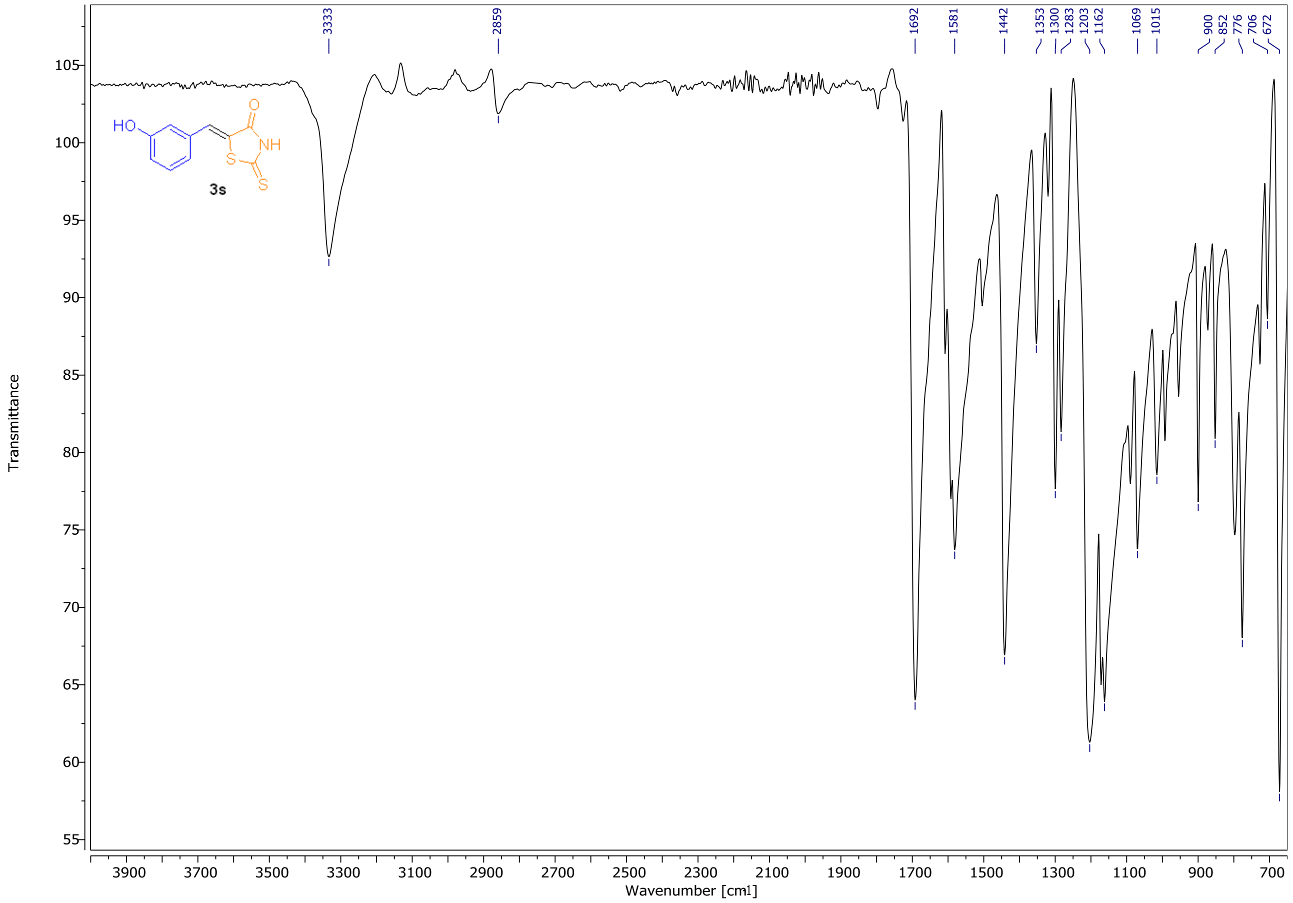

Figure S78. FTIR (ATR) of compound **3s**.

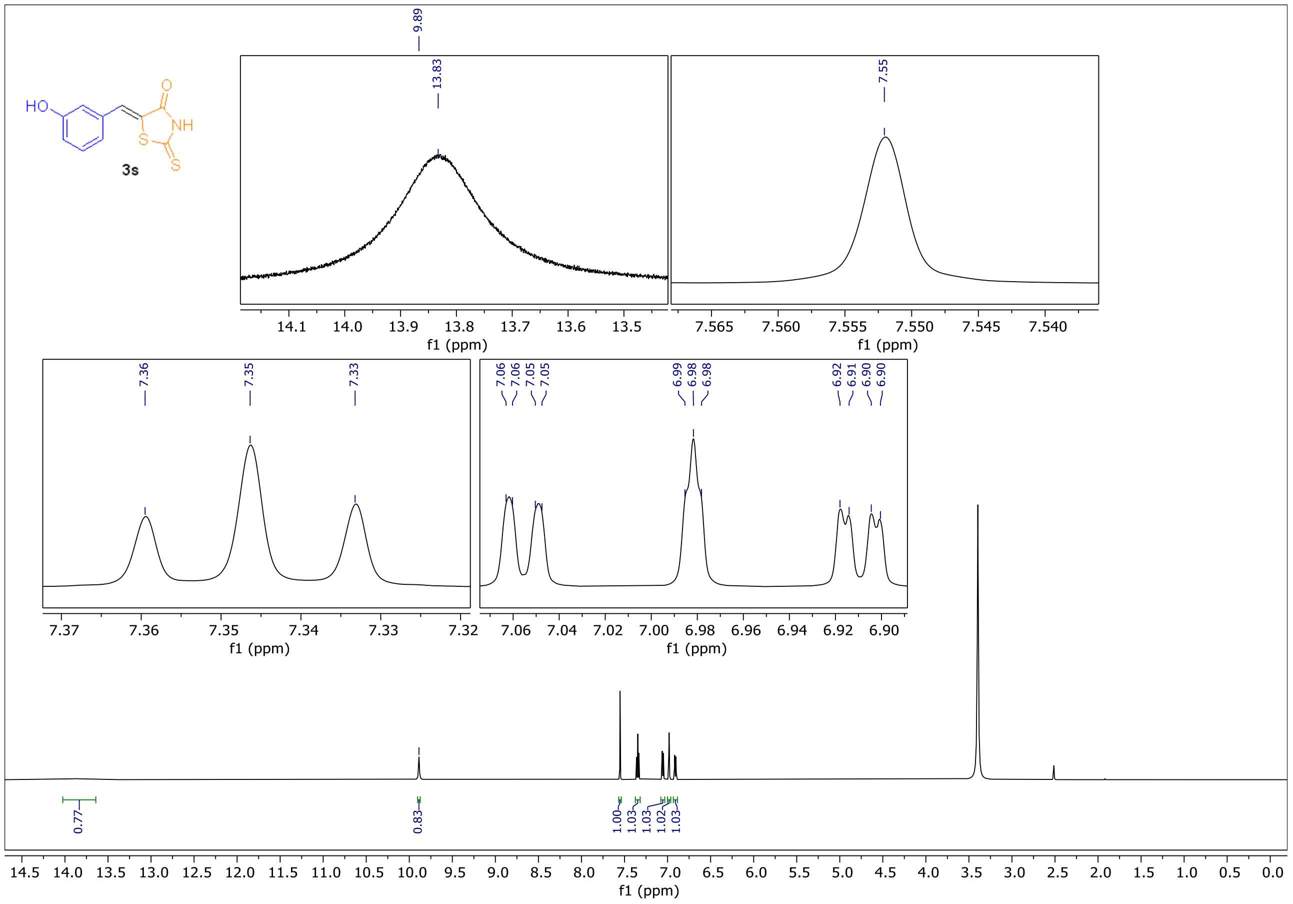

Figure S79. ^{1}H NMR spectrum (600 MHz, DMSO-d_6) of compound **3s**.

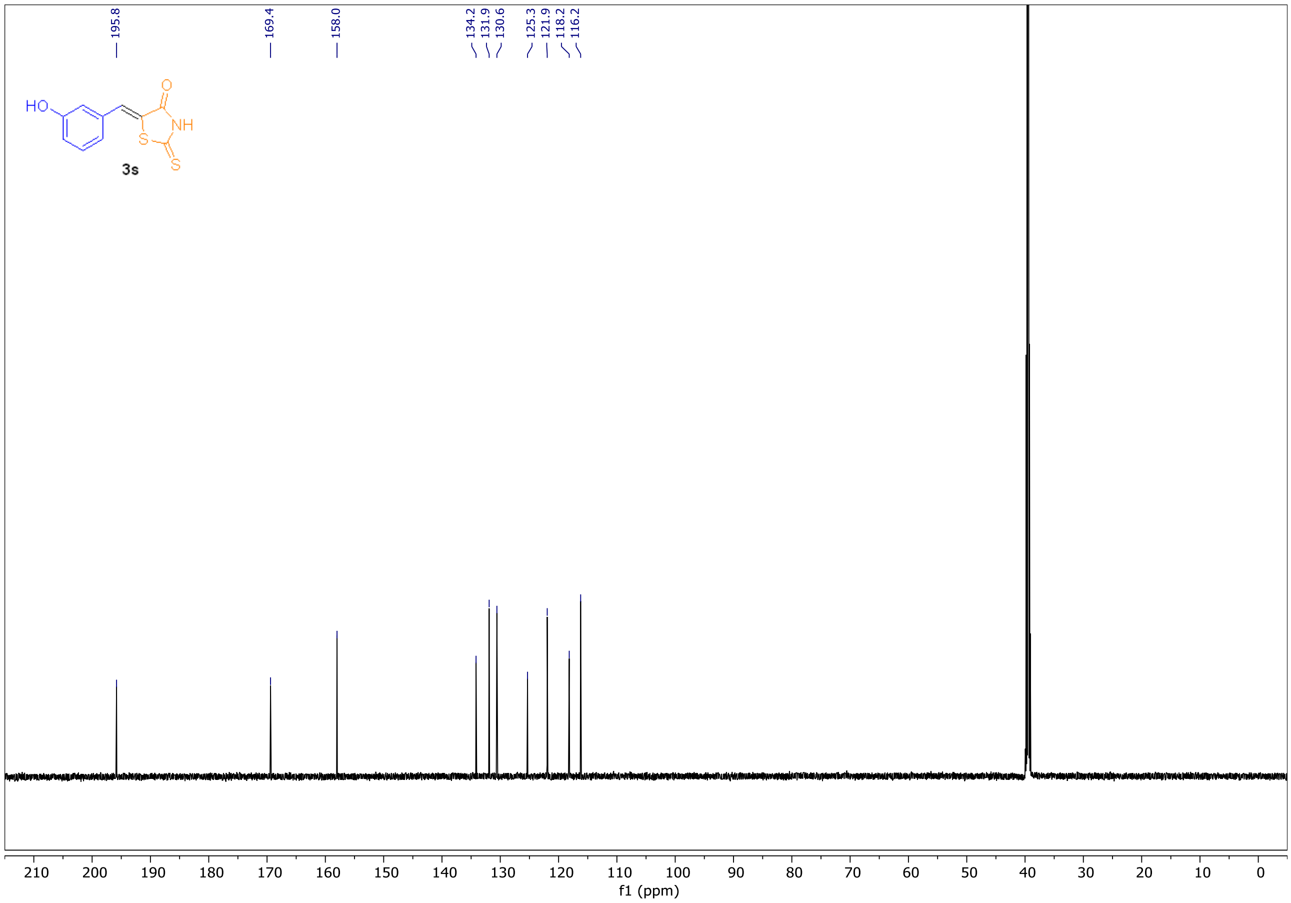

Figure S80. ^{13}C NMR spectrum (151 MHz, DMSO-d_6) of compound **3s**.

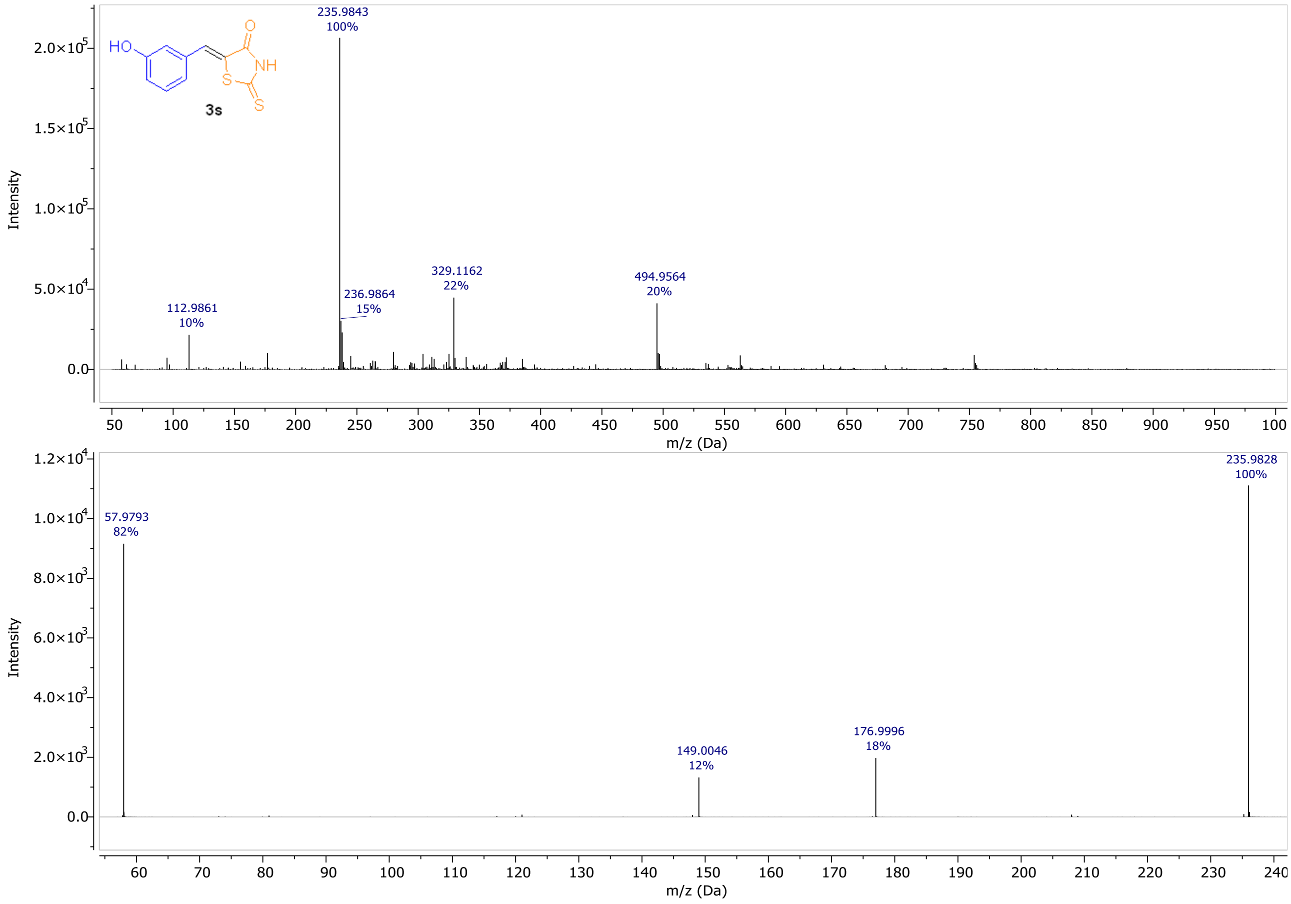

Figure S81. HRMS (ESI-QTOF) of compound **3s** and HRMS/MS for $[M-H]^-$.

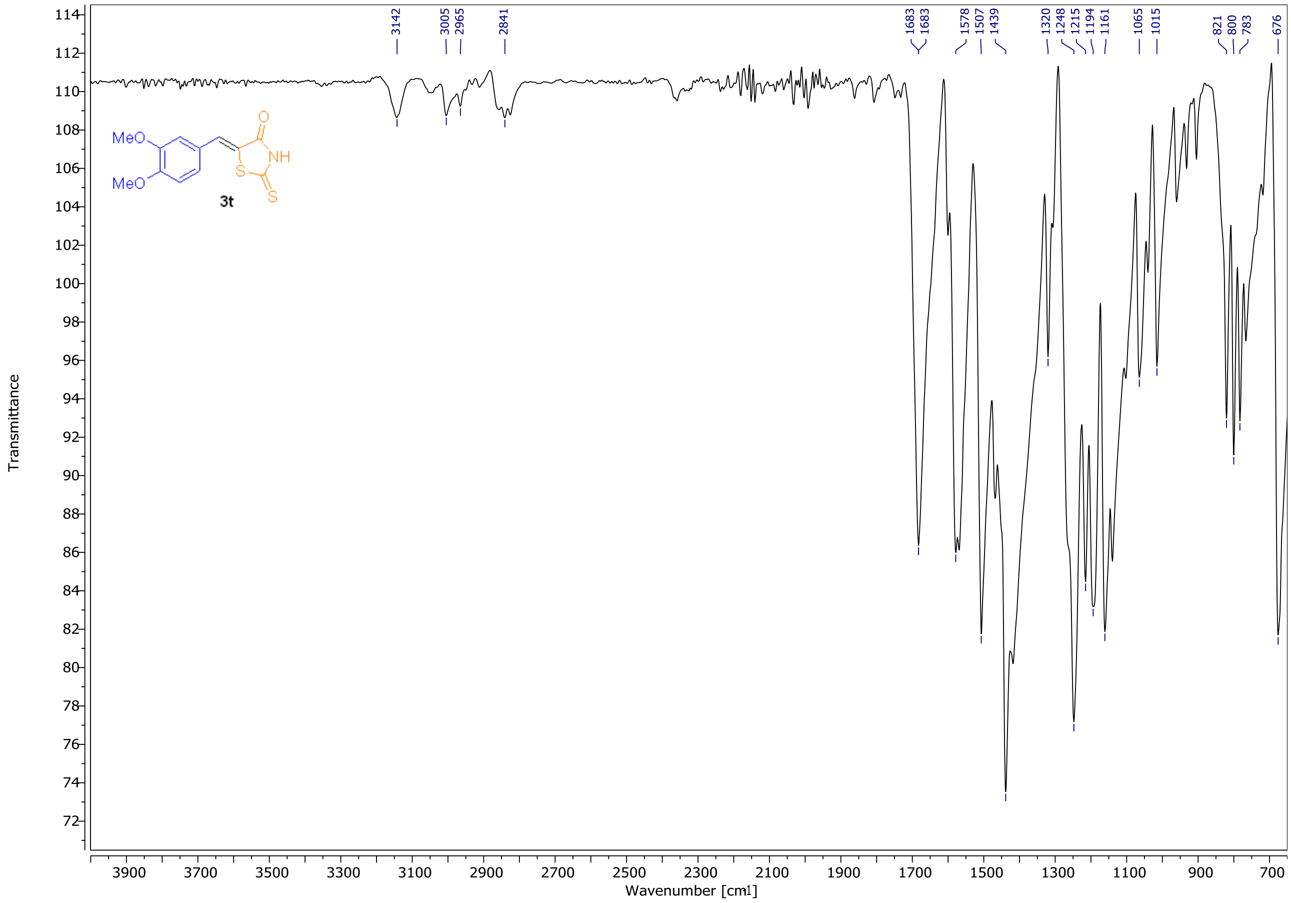

Figure S82. FTIR (ATR) of compound **3t**.

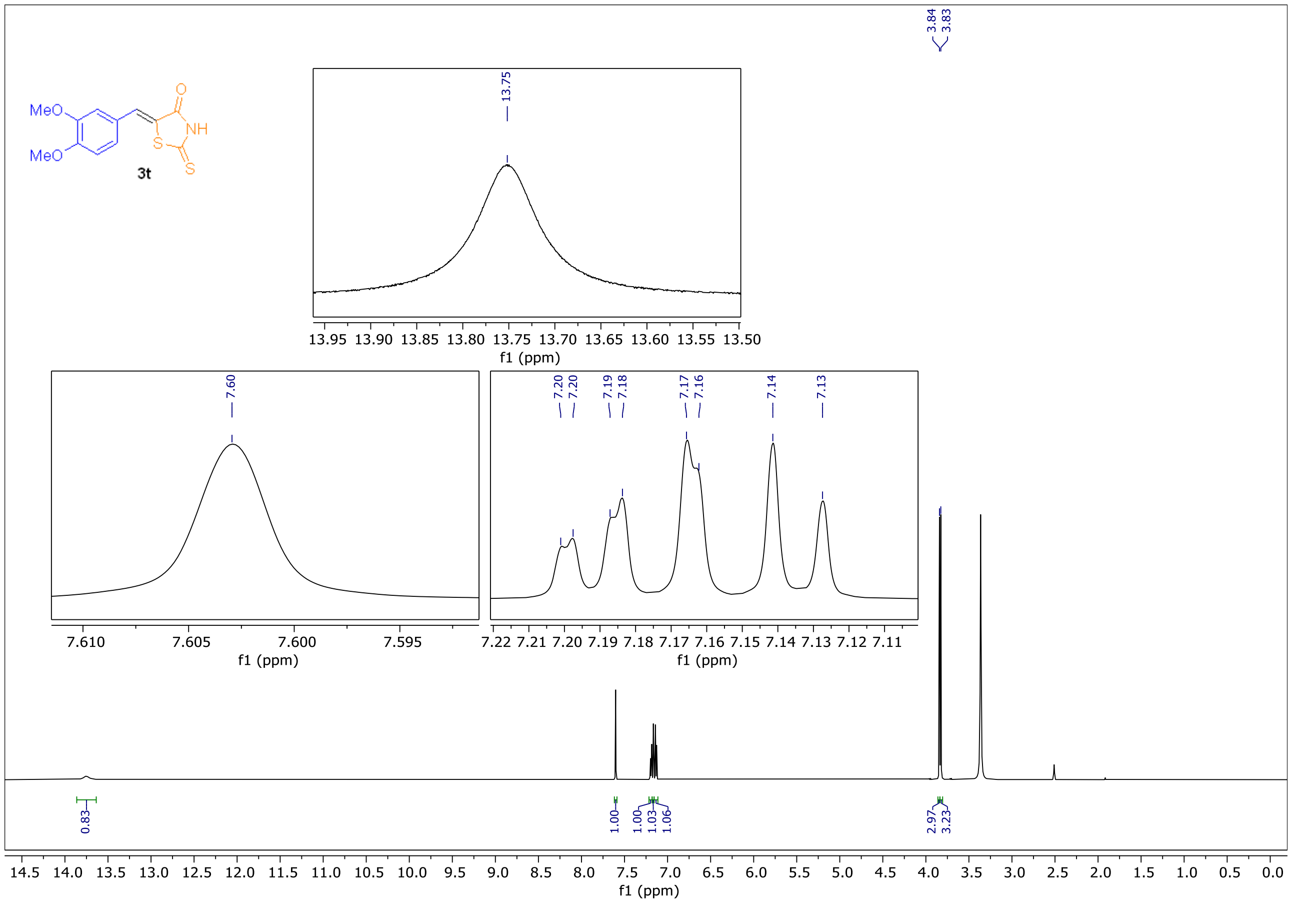

Figure S83. ^{1}H NMR spectrum (600 MHz, DMSO-d_6) of compound **3t**.

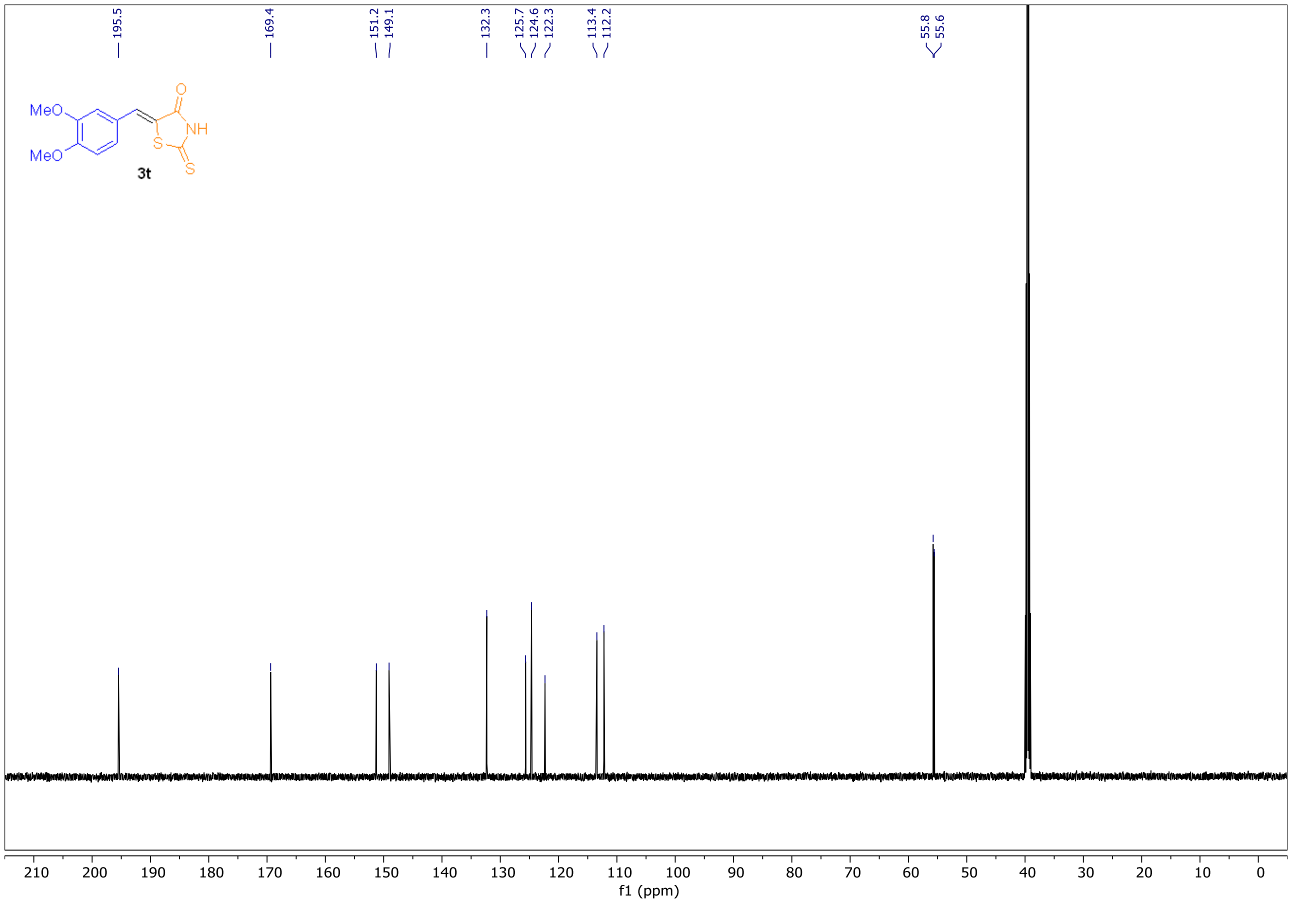

Figure S84. ^{13}C NMR spectrum (151 MHz, DMSO-d_6) of compound **3t**.

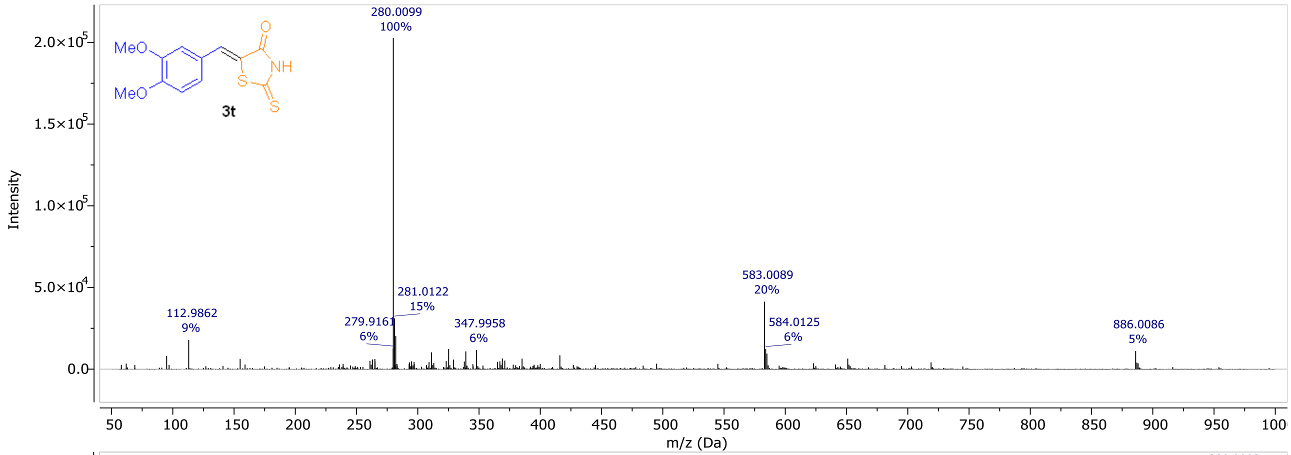

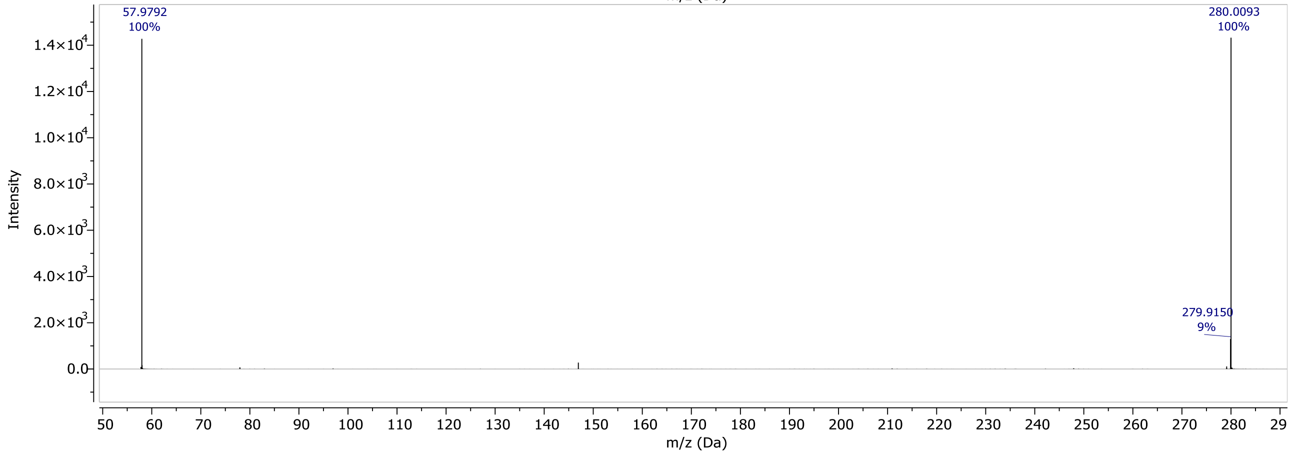

Figure S85. HRMS (ESI-QTOF) of compound **3t** and HRMS/MS for [M−H]⁻.

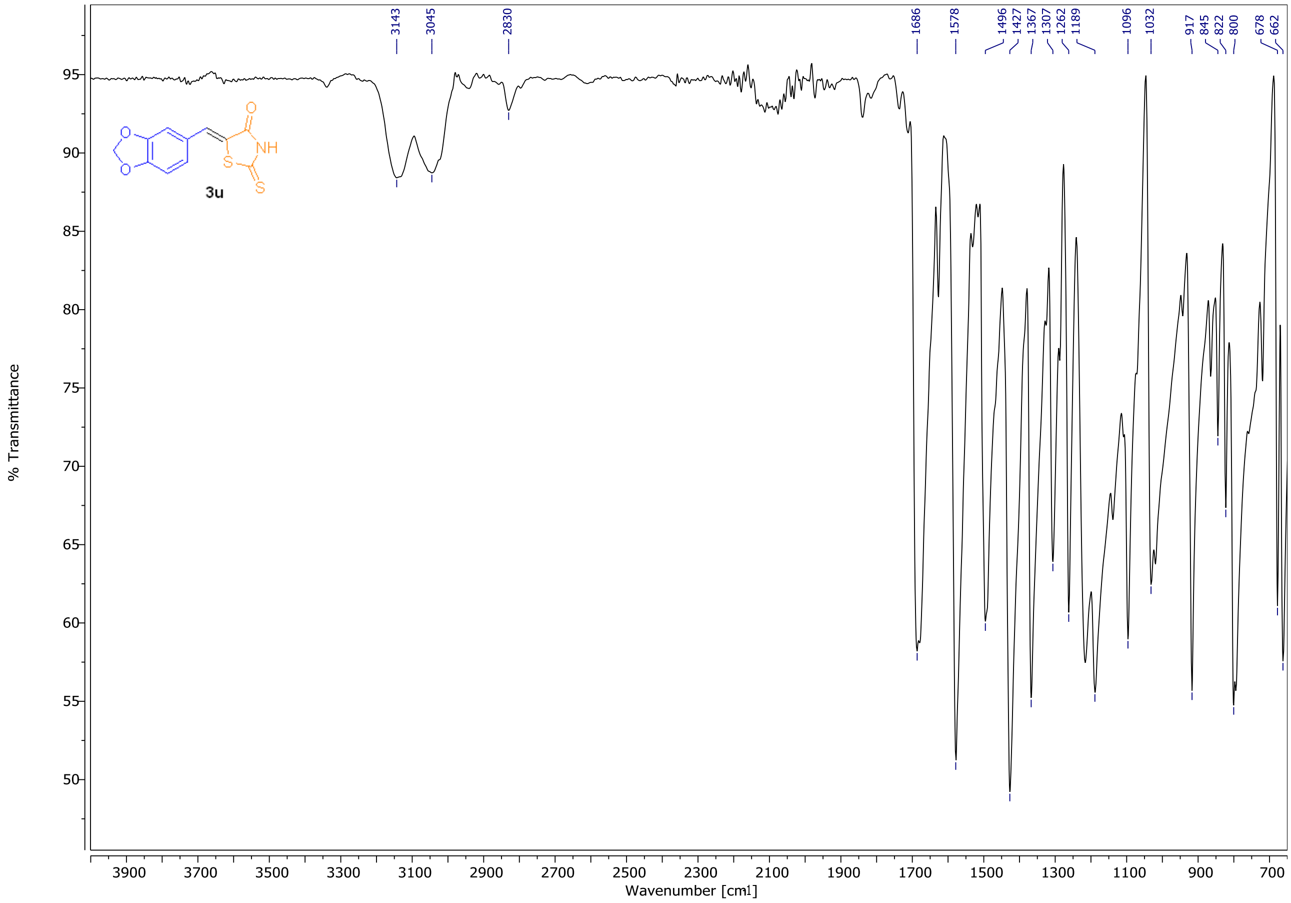

Figure S86. FTIR (ATR) of compound **3u**.

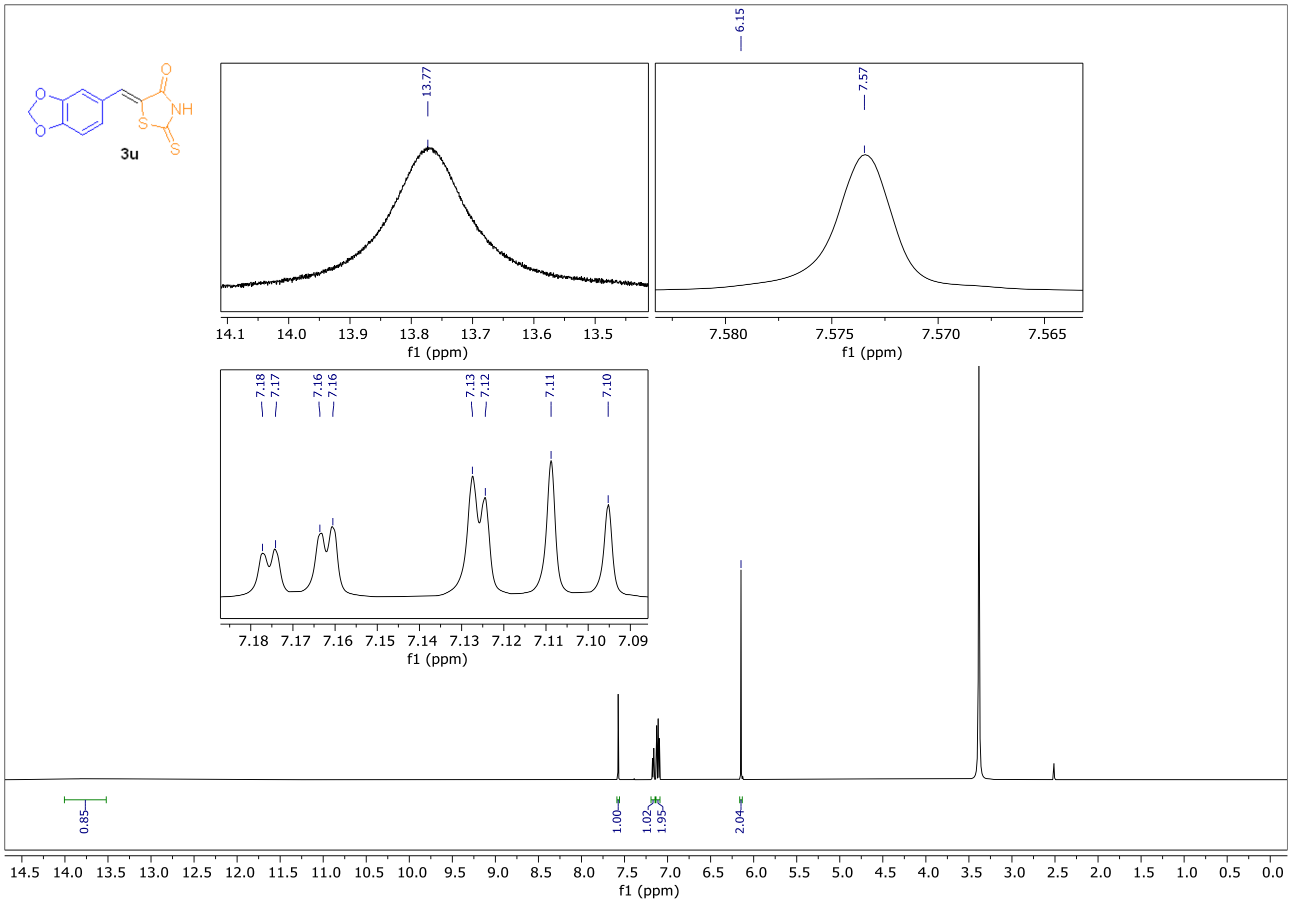

Figure S87. ^{1}H NMR spectrum (600 MHz, DMSO-d_6) of compound **3u**.

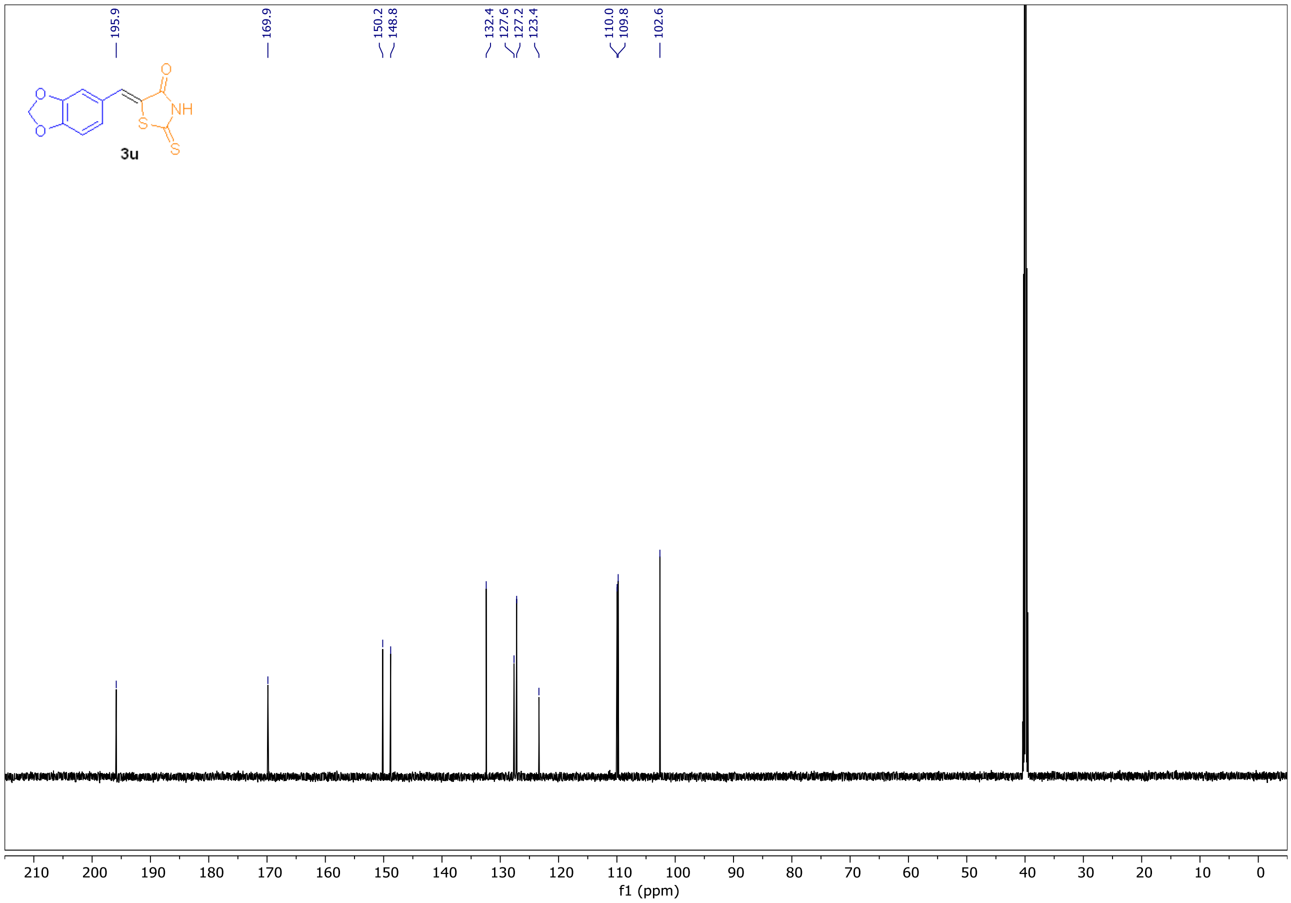

Figure S88. ^{13}C NMR spectrum (151 MHz, DMSO-d_6) of compound **3u**.

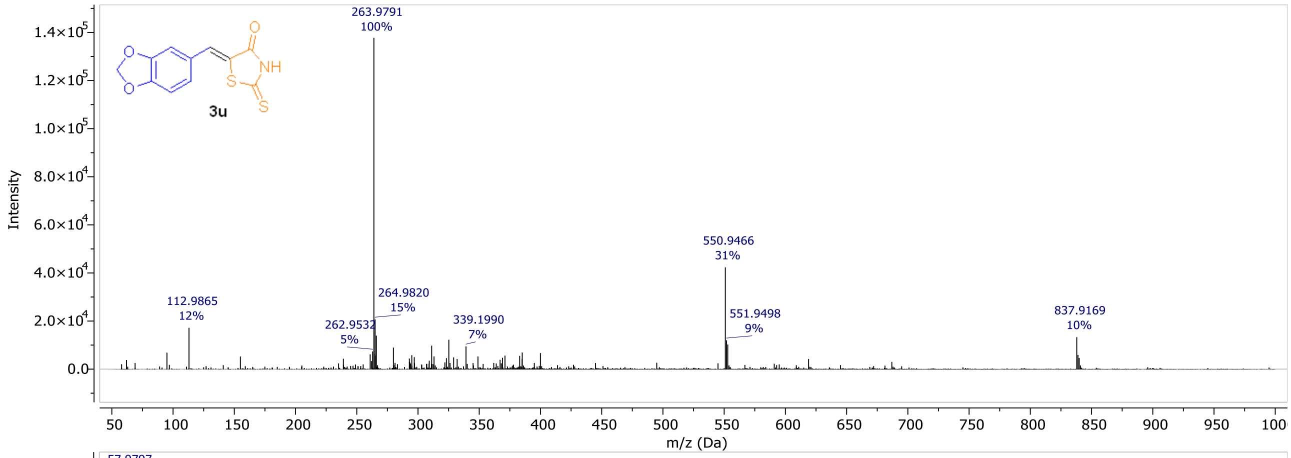

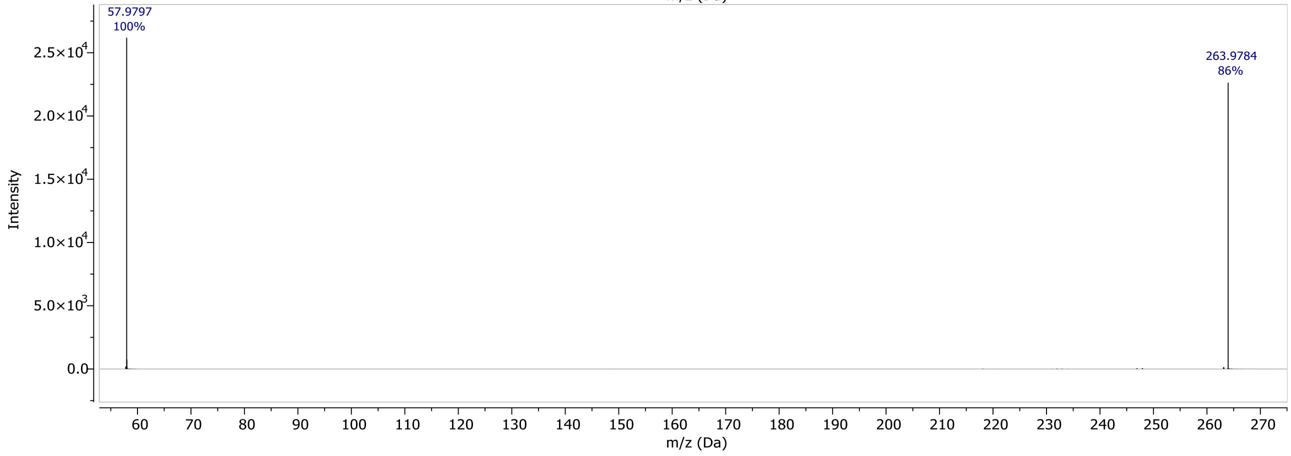

Figure S89. HRMS (ESI-QTOF) of compound **3u** and HRMS/MS for [M−H]$^-$.

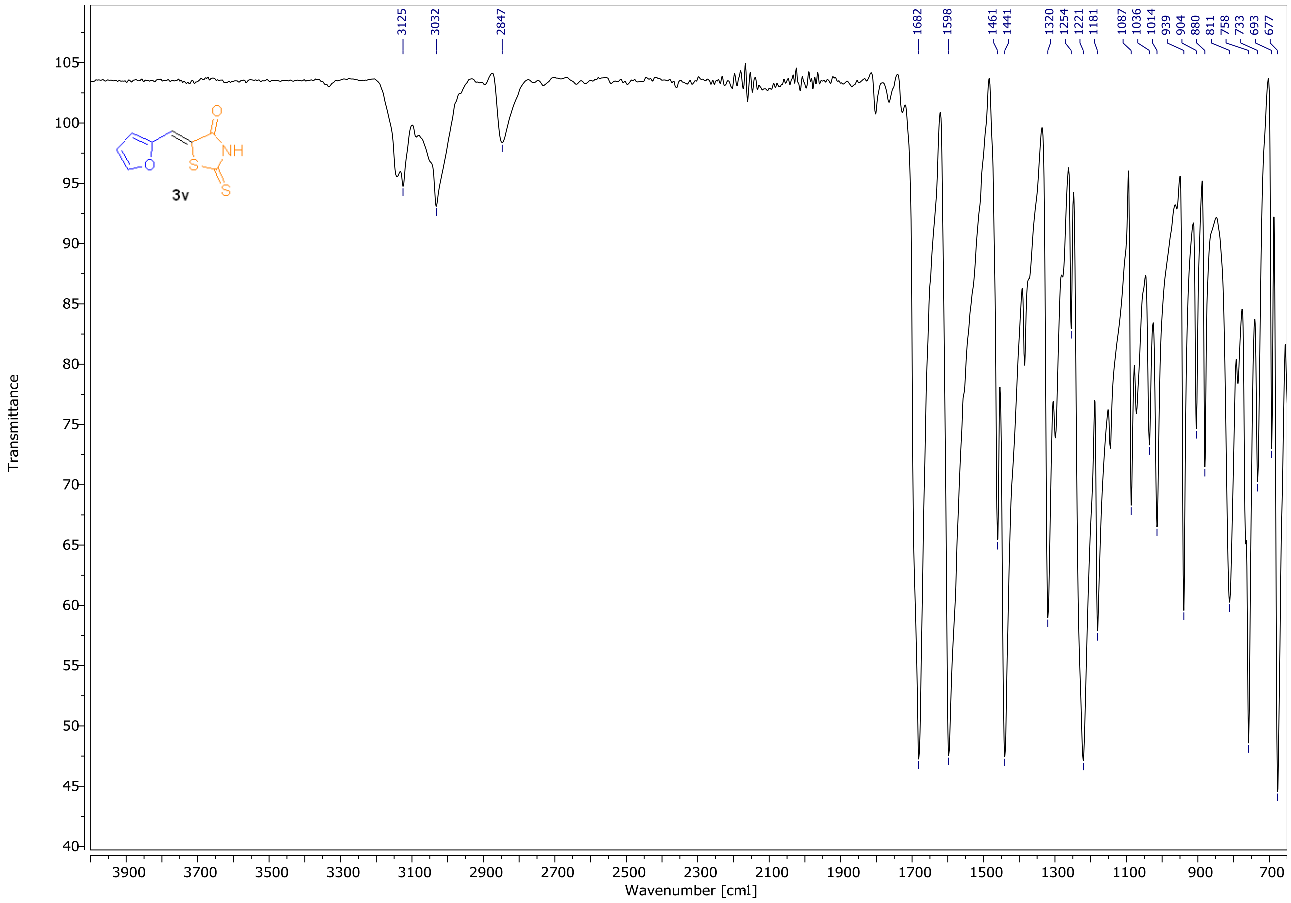

Figure S90. FTIR (ATR) of compound **3v**.

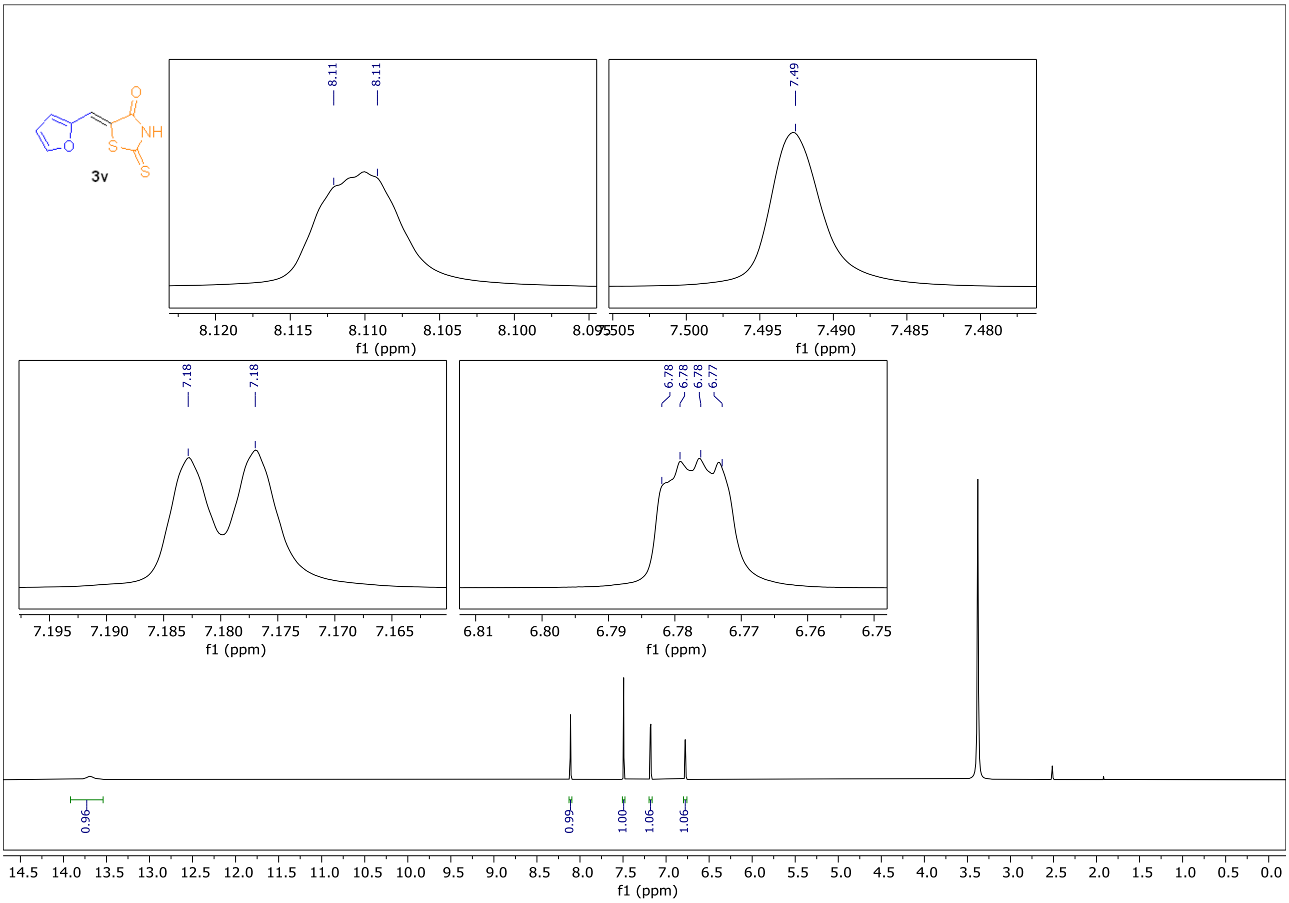

Figure S91. ^{1}H NMR spectrum (600 MHz, DMSO-d_6) of compound **3v**.

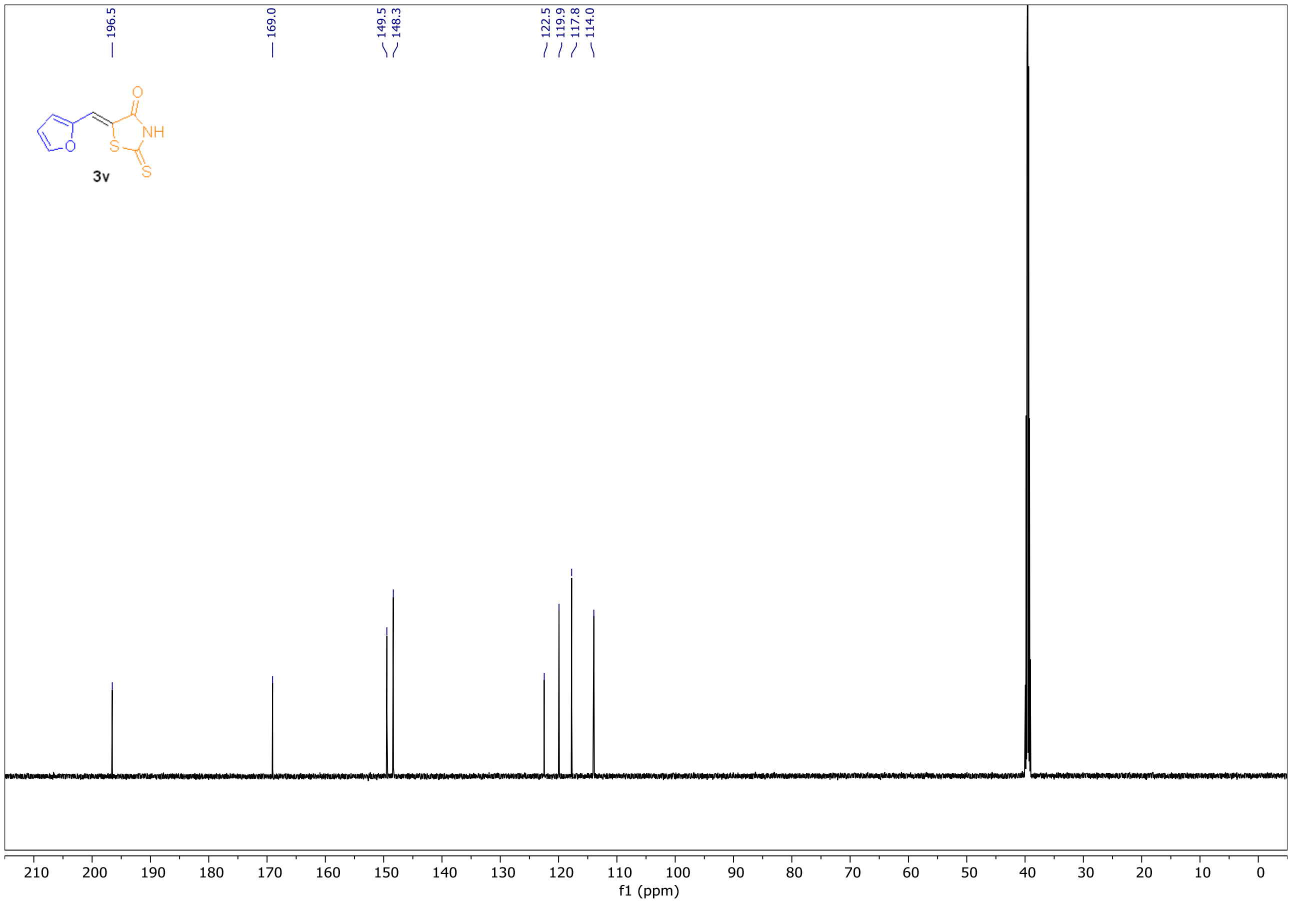

Figure S92. ^{13}C NMR spectrum (151 MHz, DMSO-d_6) of compound **3v**.

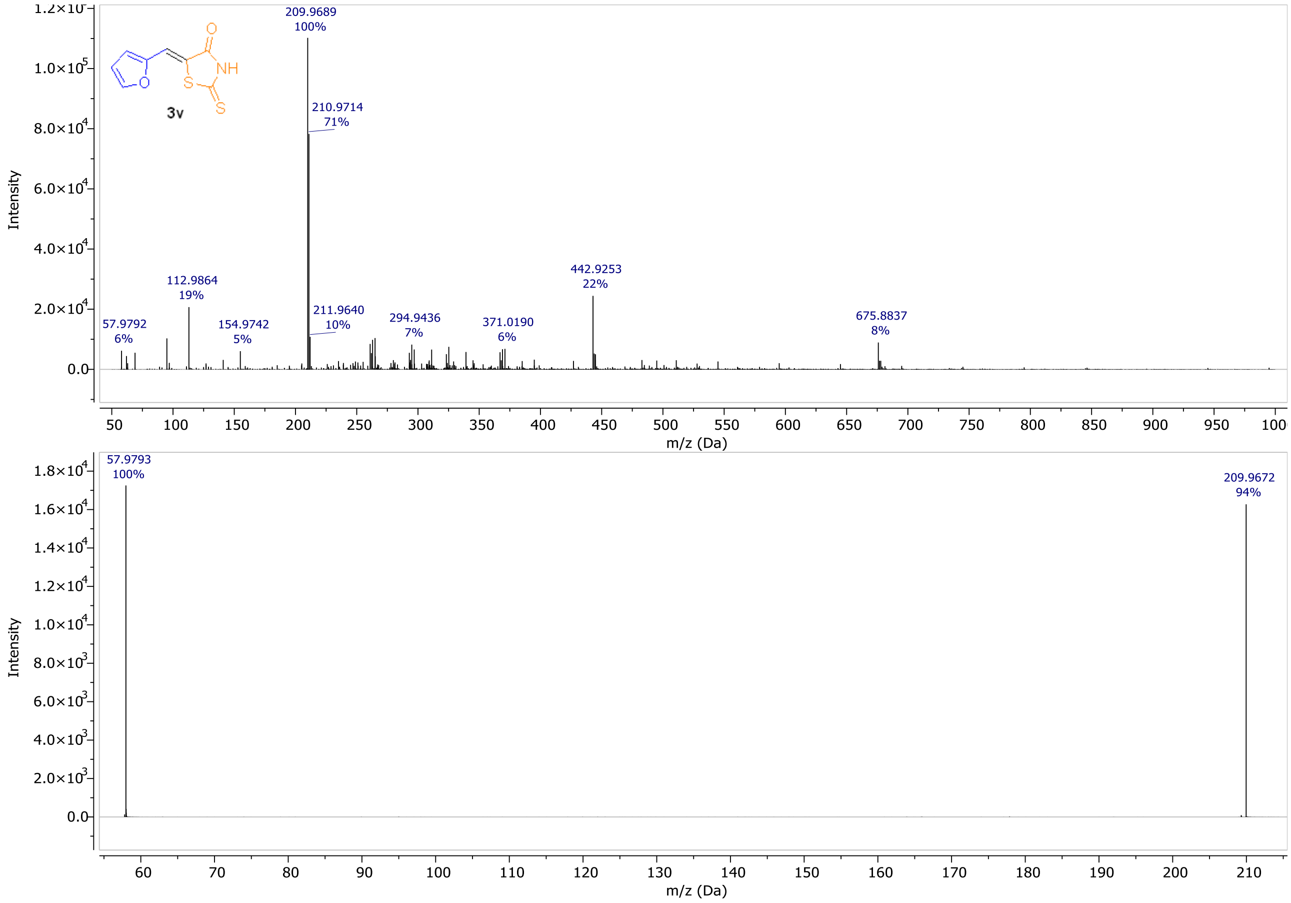

Figure S93. HRMS (ESI-QTOF) of compound **3v** and HRMS/MS for [M−H]$^-$.

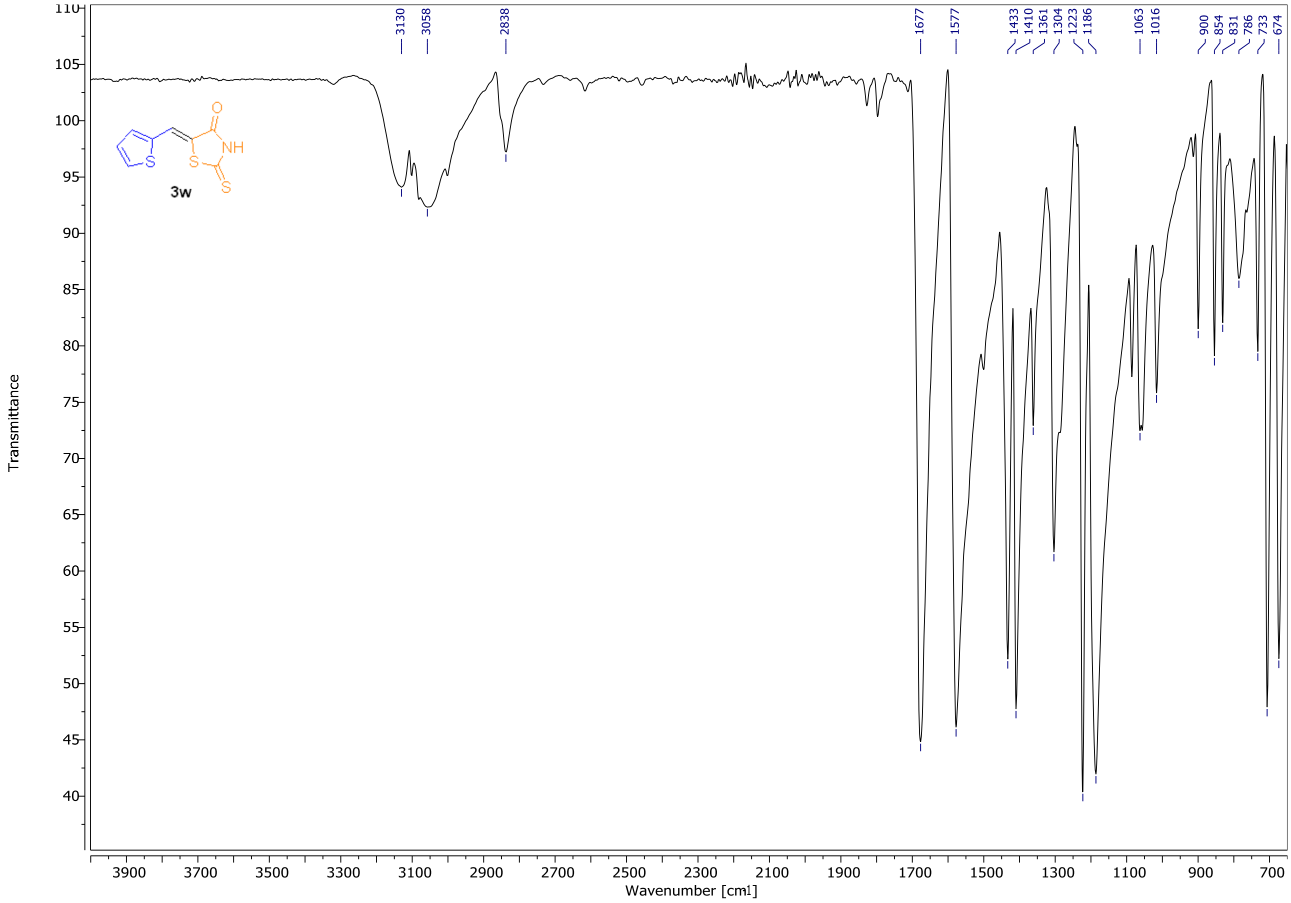

Figure S94. FTIR (ATR) of compound **3w**.

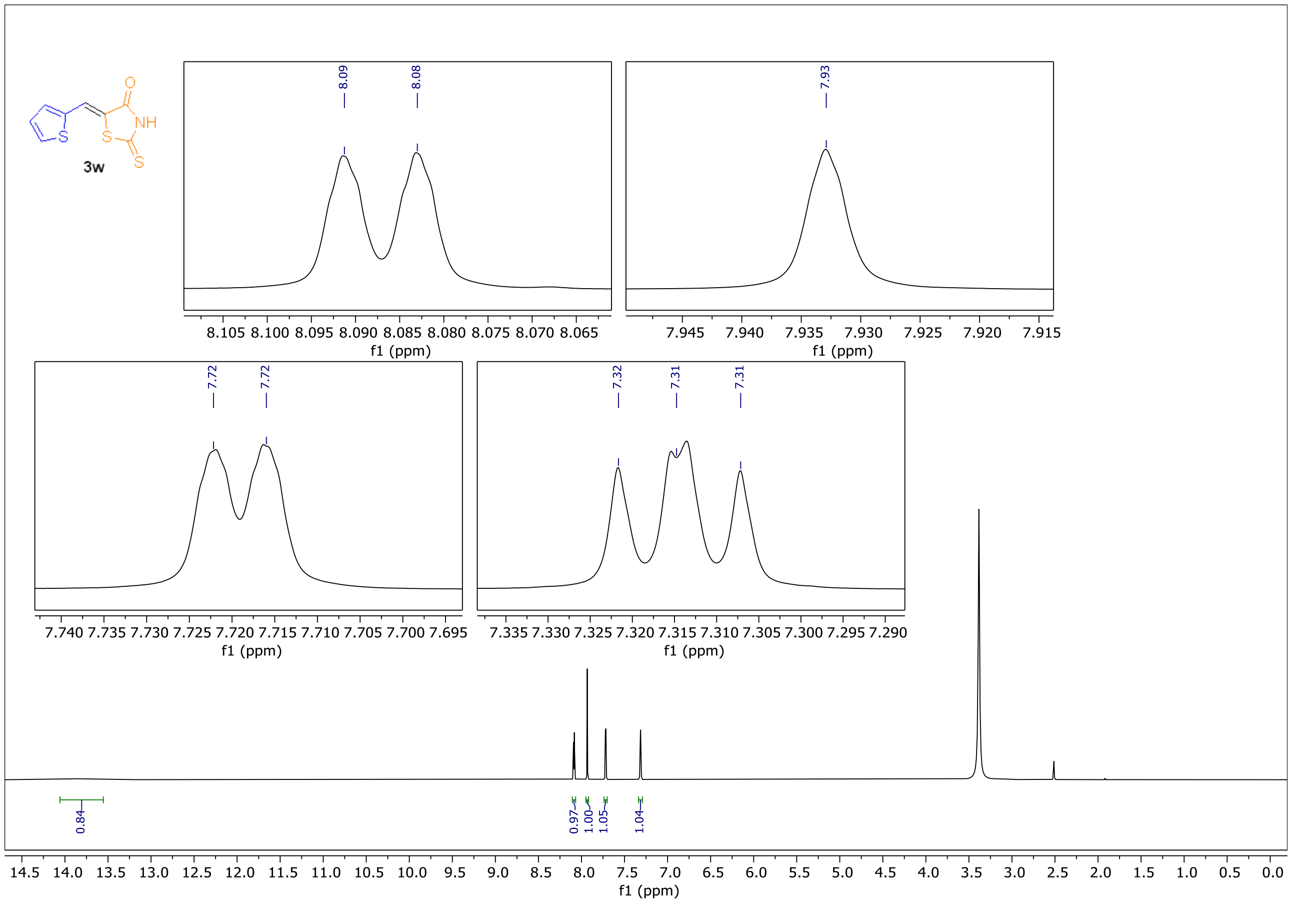

Figure S95. ^{1}H NMR spectrum (600 MHz, DMSO-d_6) of compound **3w**.

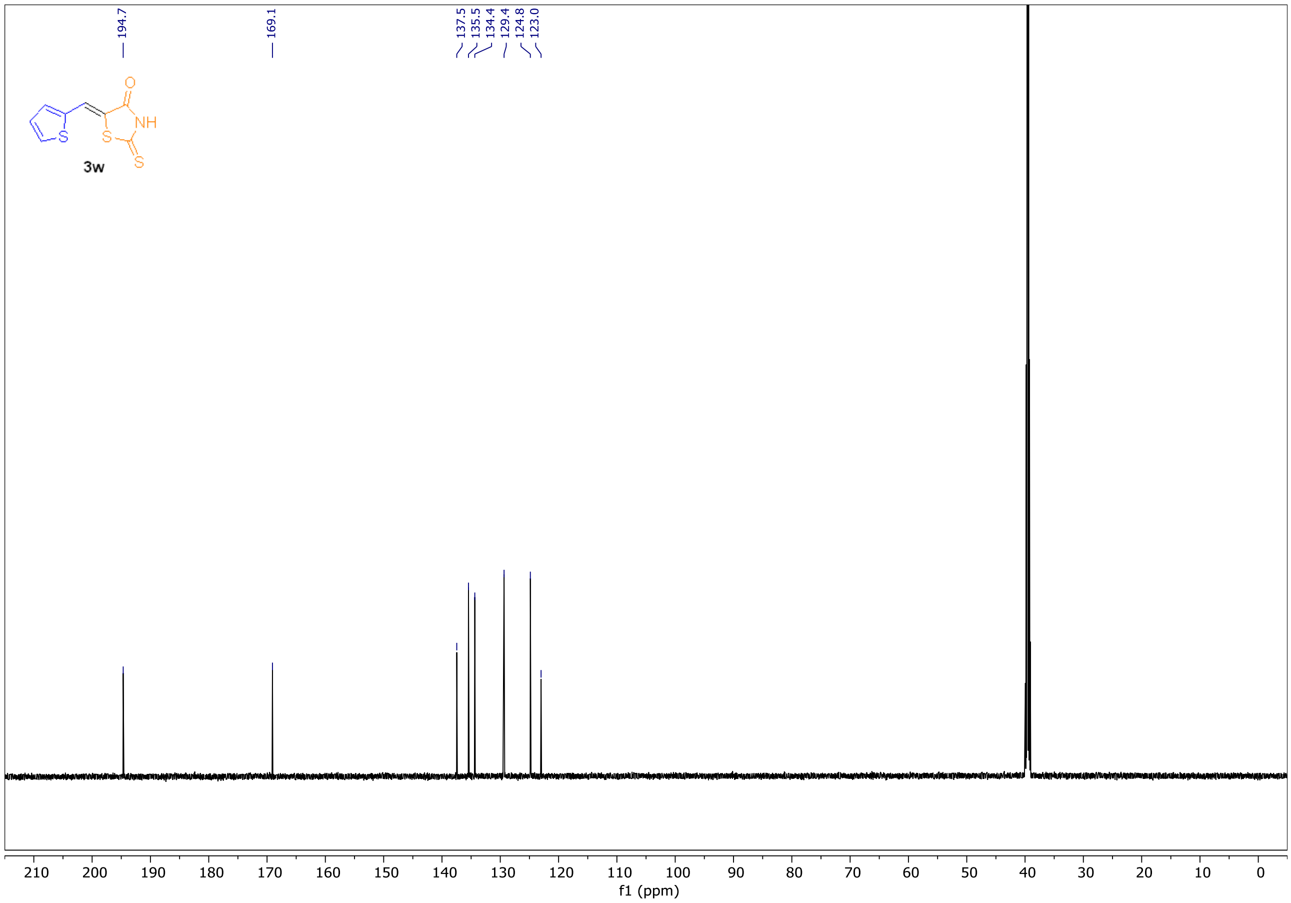

Figure S96. ^{13}C NMR spectrum (151 MHz, DMSO-d_6) of compound **3w**.

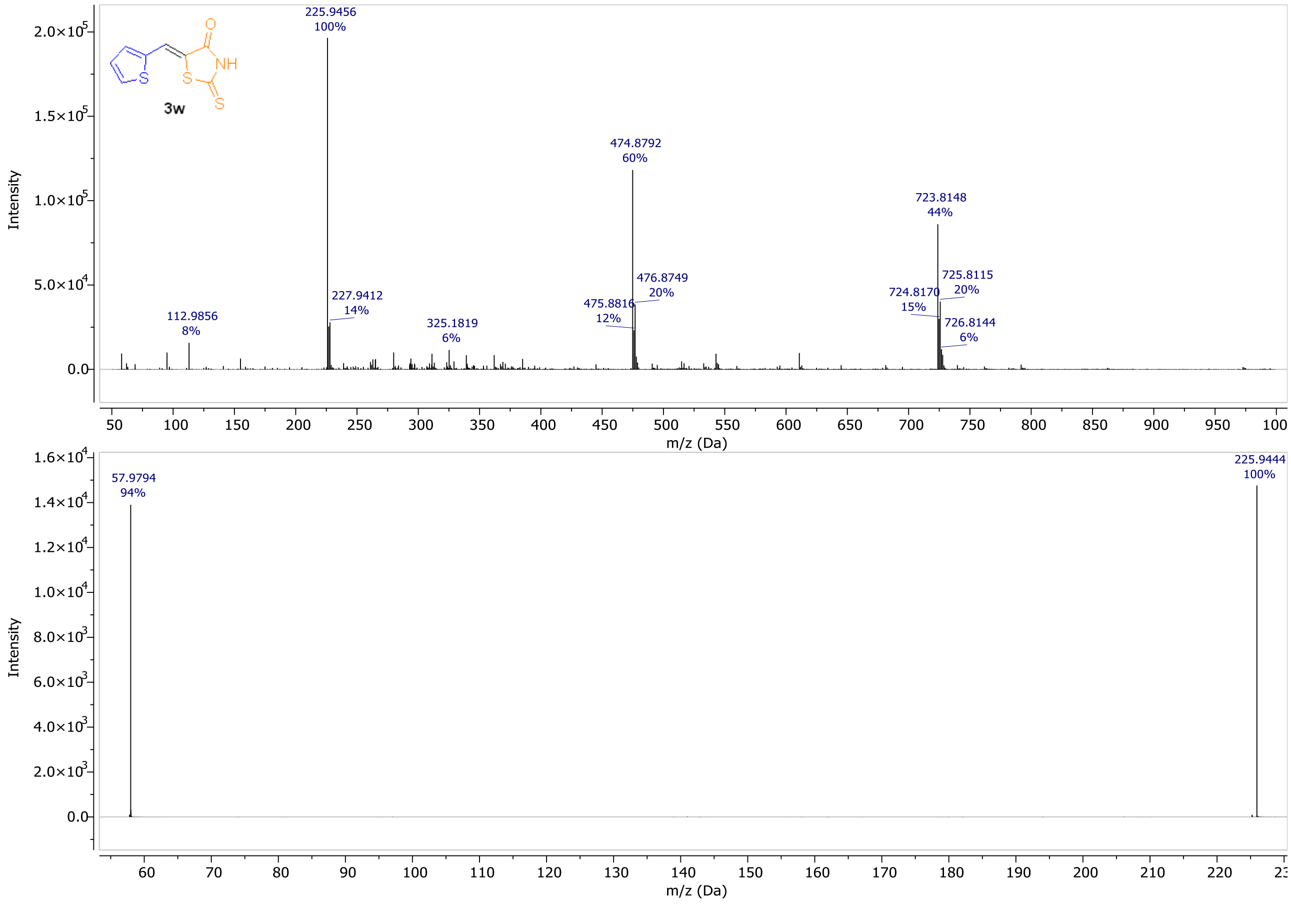

Figure S97. HRMS (ESI-QTOF) of compound **3w** and HRMS/MS for [M−H]⁻.

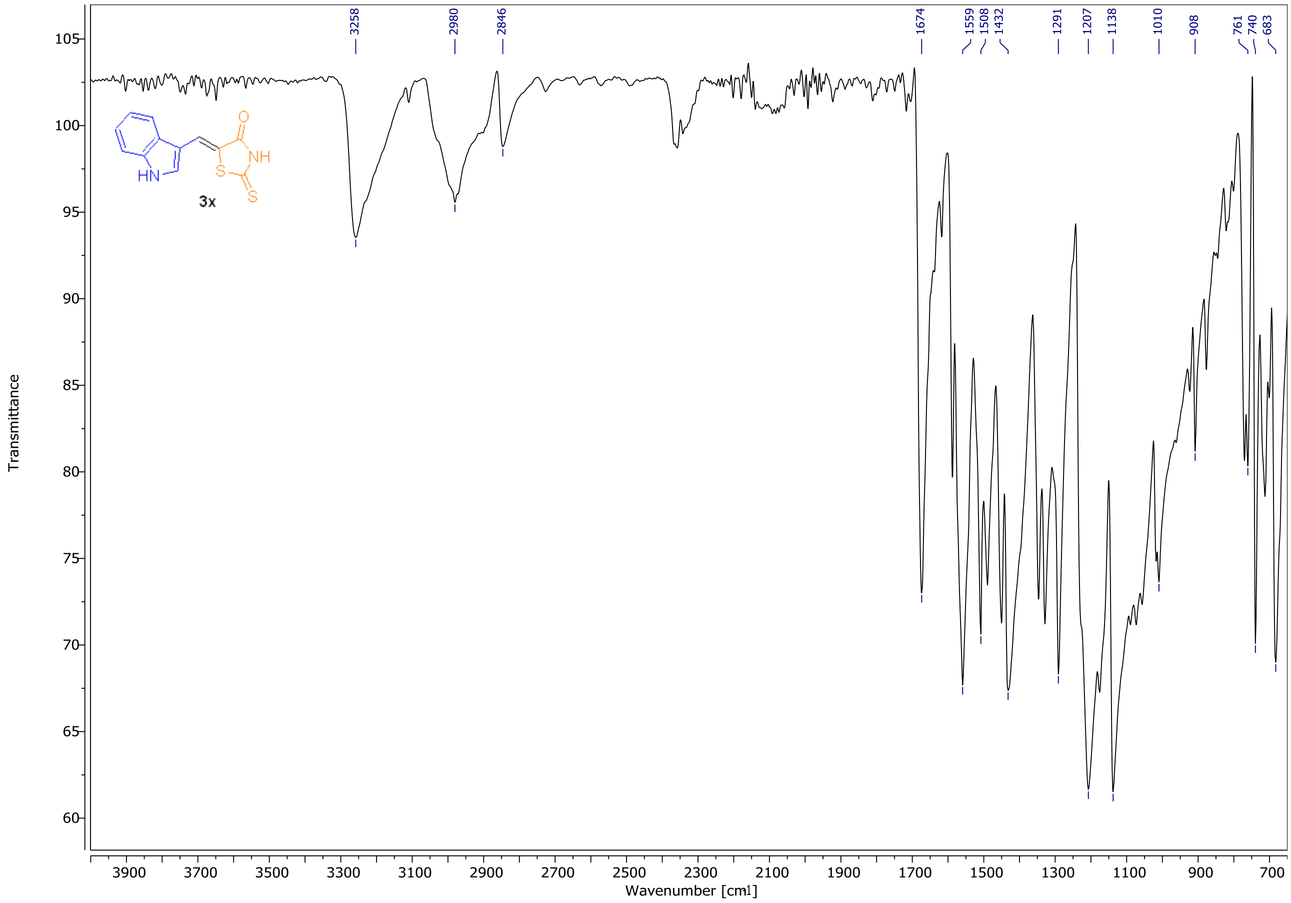

Figure S98. FTIR (ATR) of compound **3x**.

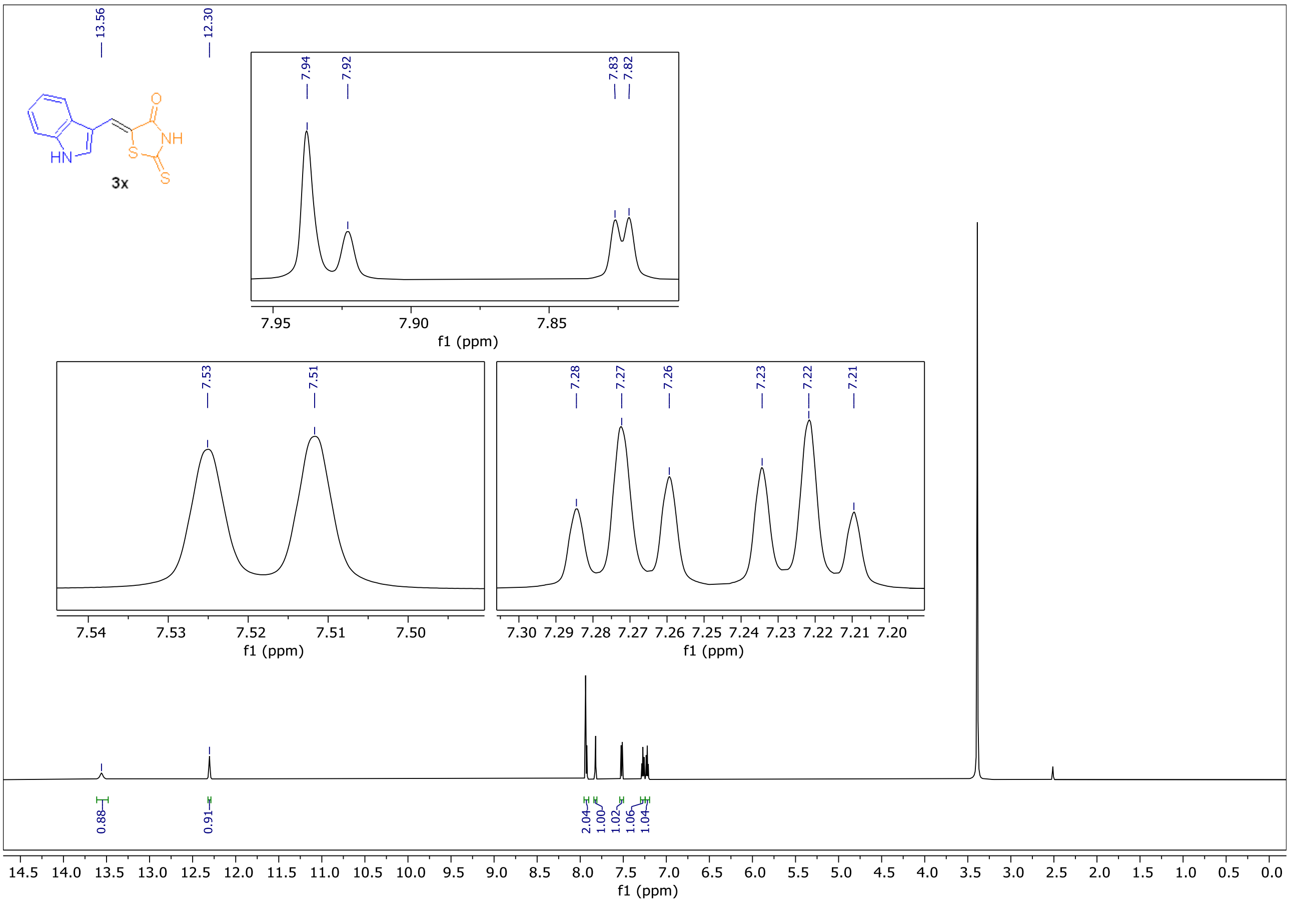

Figure S99. ^{1}H NMR spectrum (600 MHz, DMSO-d_6) of compound **3x**.

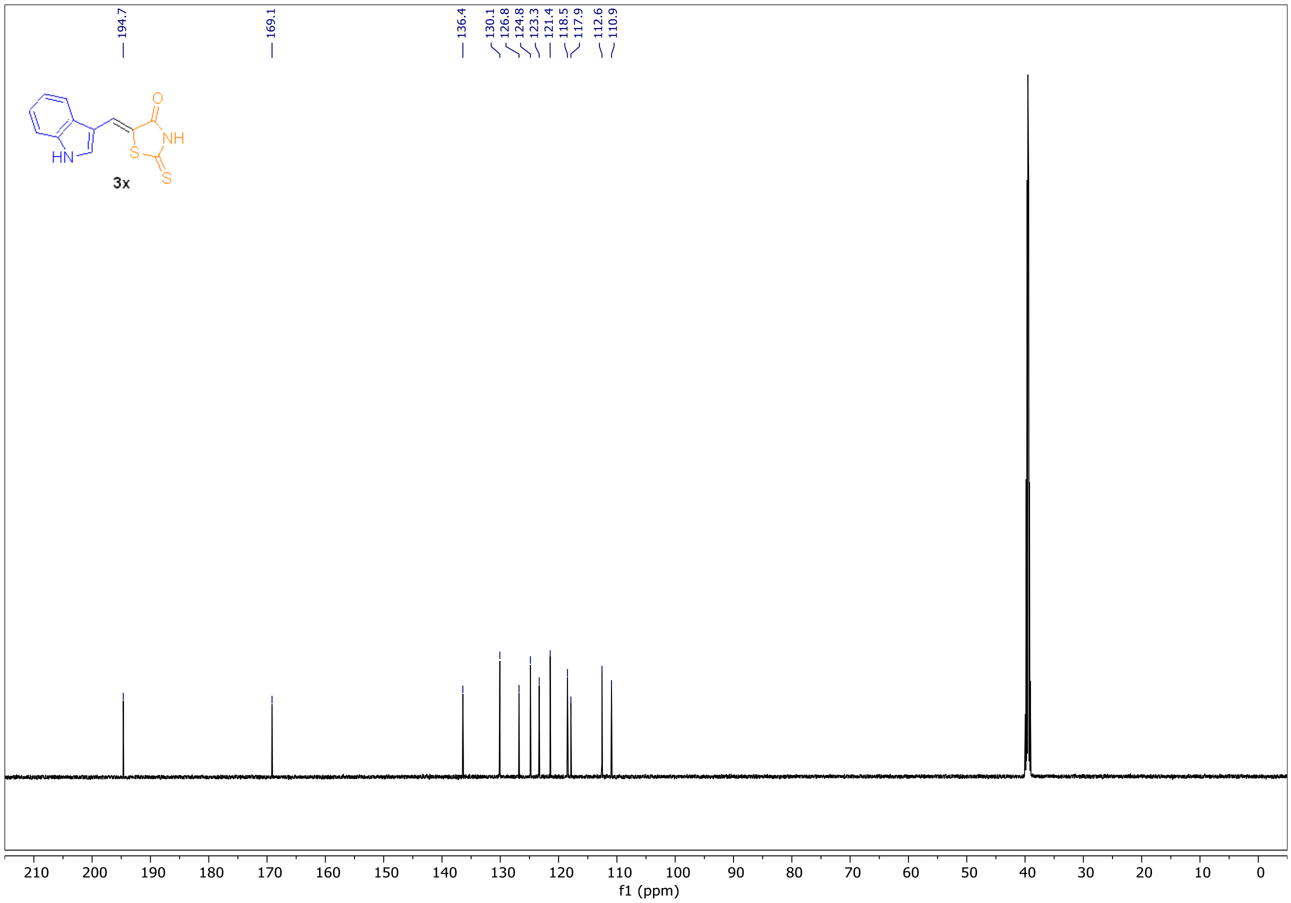

Figure S100. ^{13}C NMR spectrum (151 MHz, DMSO-d_6) of compound **3x**.

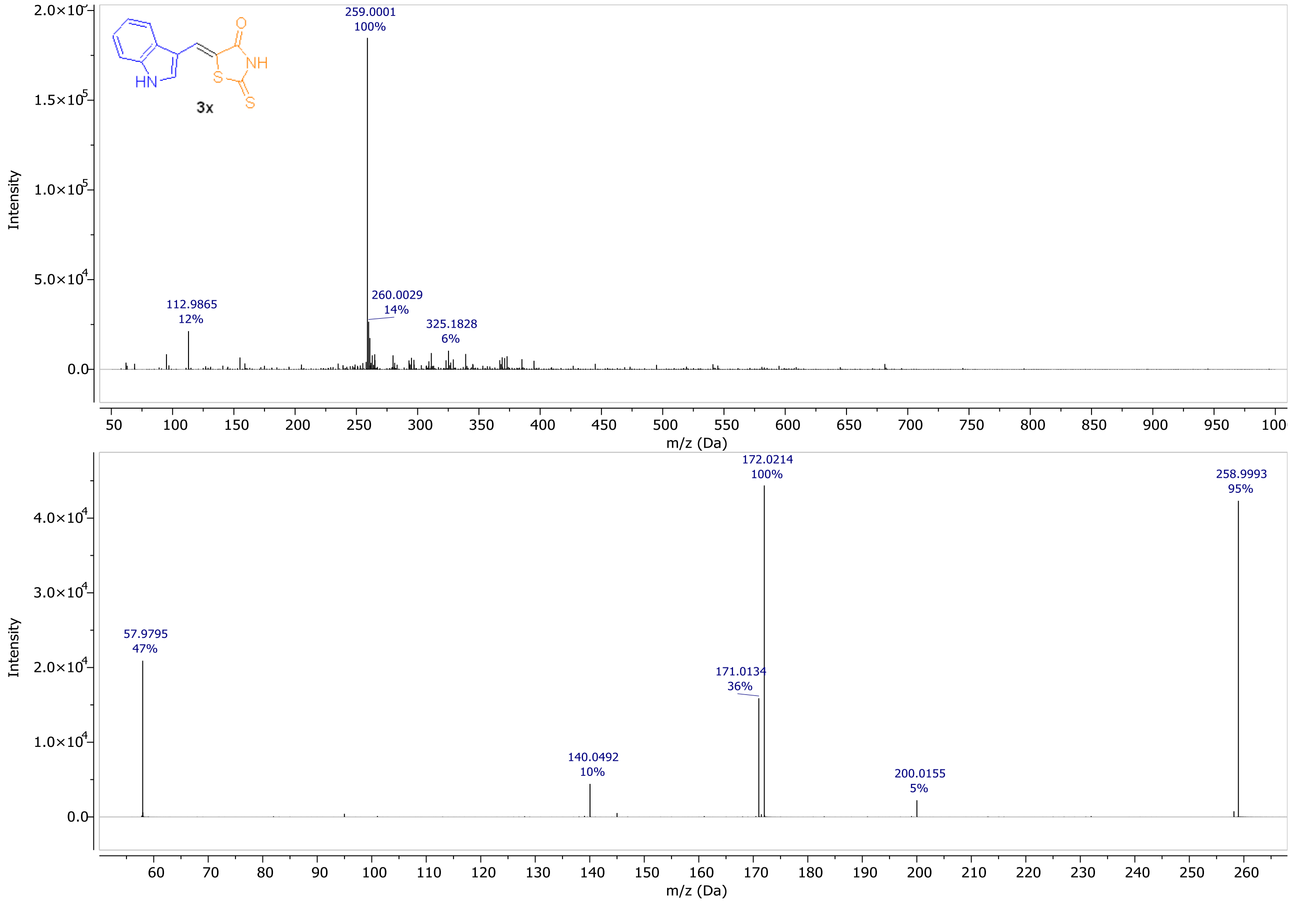

Figure S101. HRMS (ESI-QTOF) of compound **3x** and HRMS/MS for [M−H]⁻.

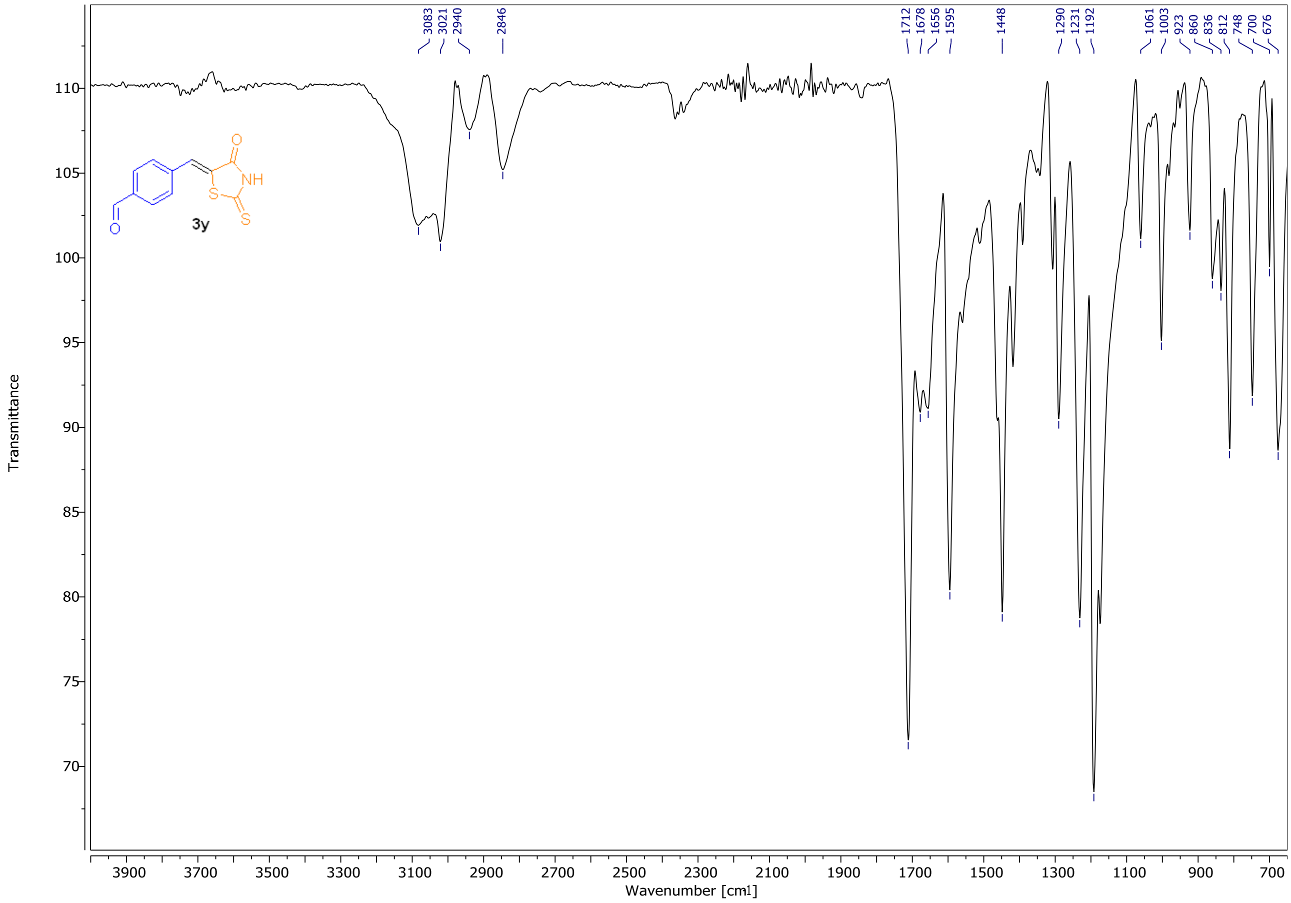

Figure S102. FTIR (ATR) of compound **3y**.

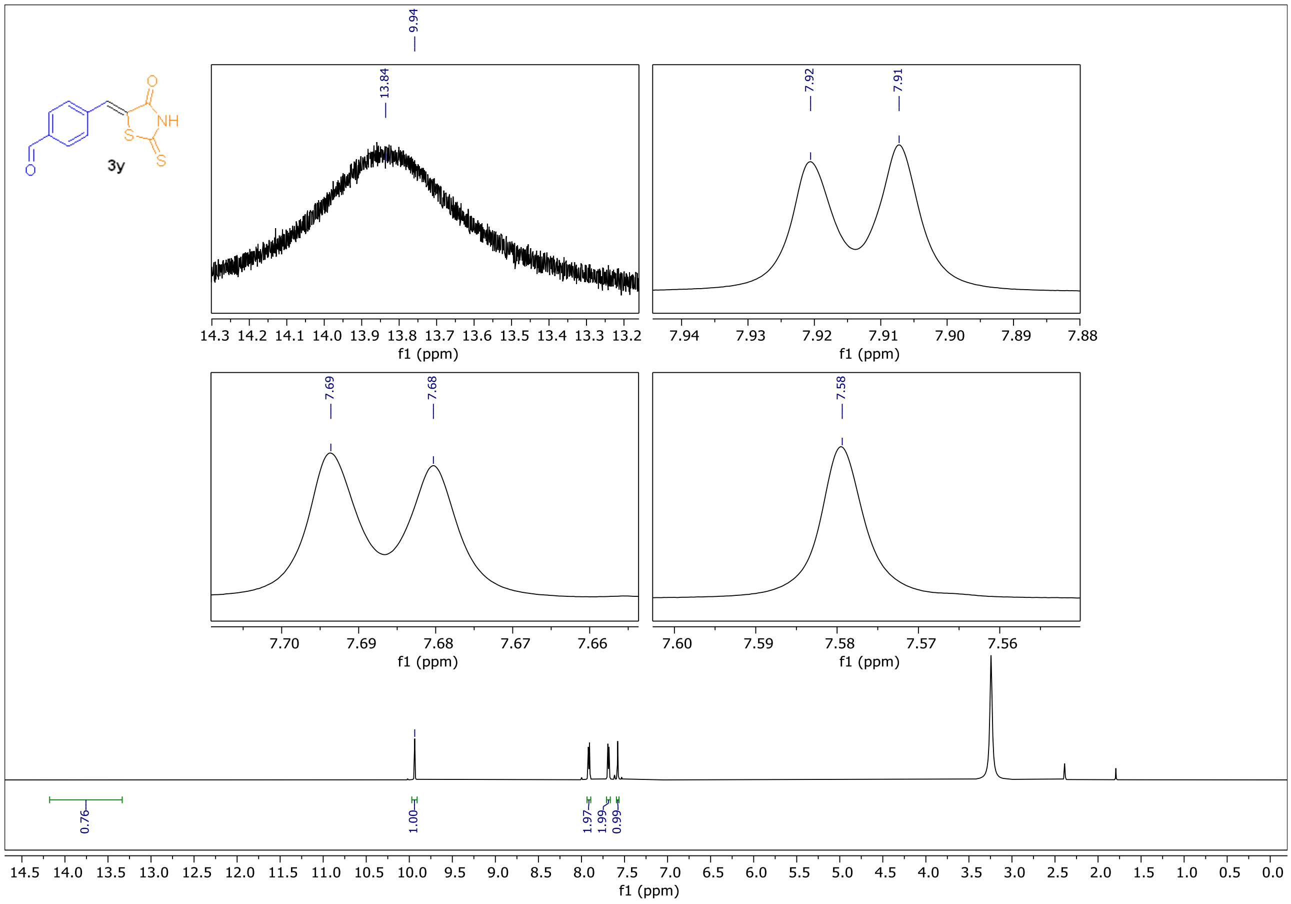

Figure S103. ^{1}H NMR spectrum (600 MHz, DMSO-d_6) of compound **3y**.

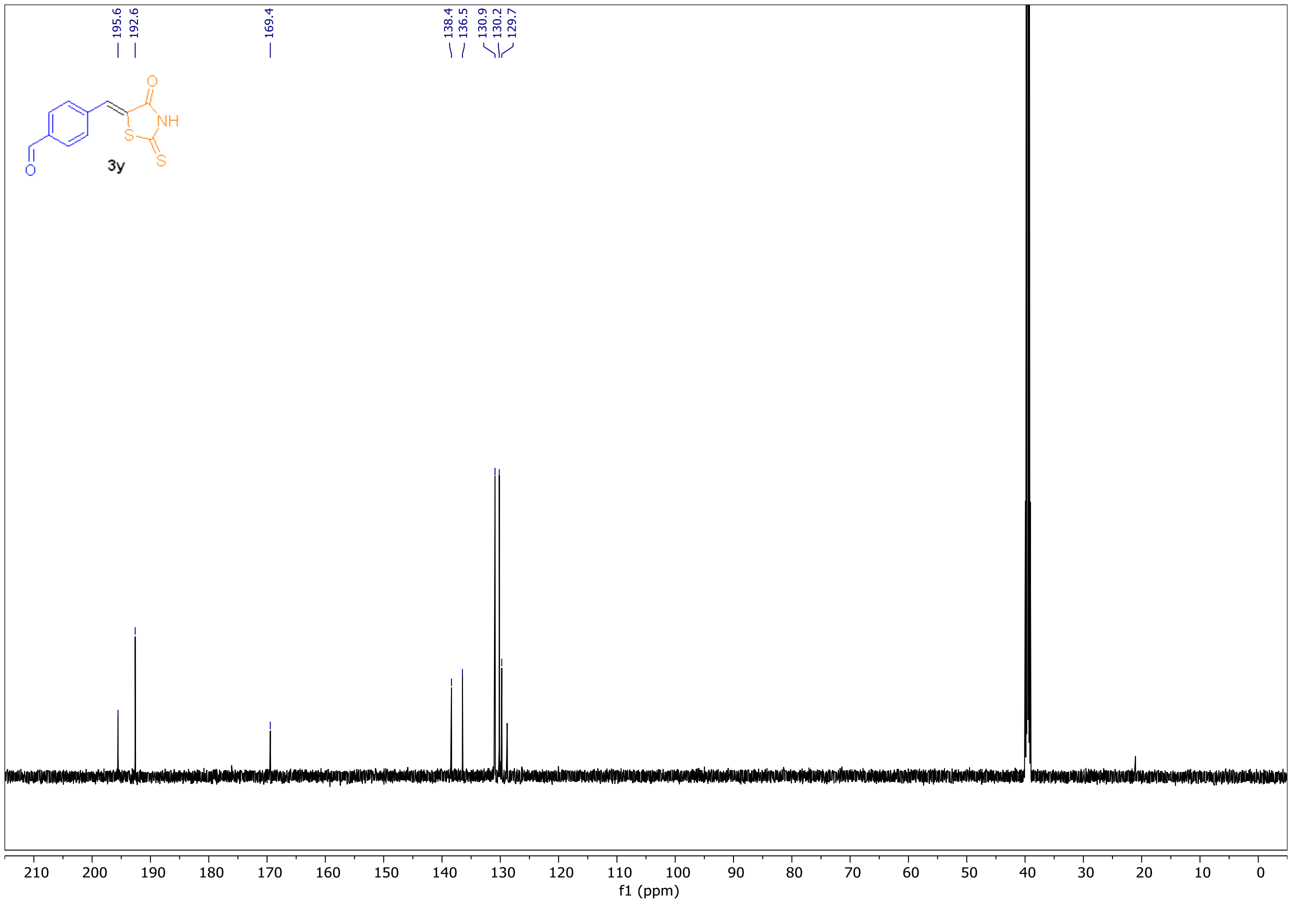

Figure S104. ^{13}C NMR spectrum (151 MHz, DMSO-d_6) of compound **3y**.

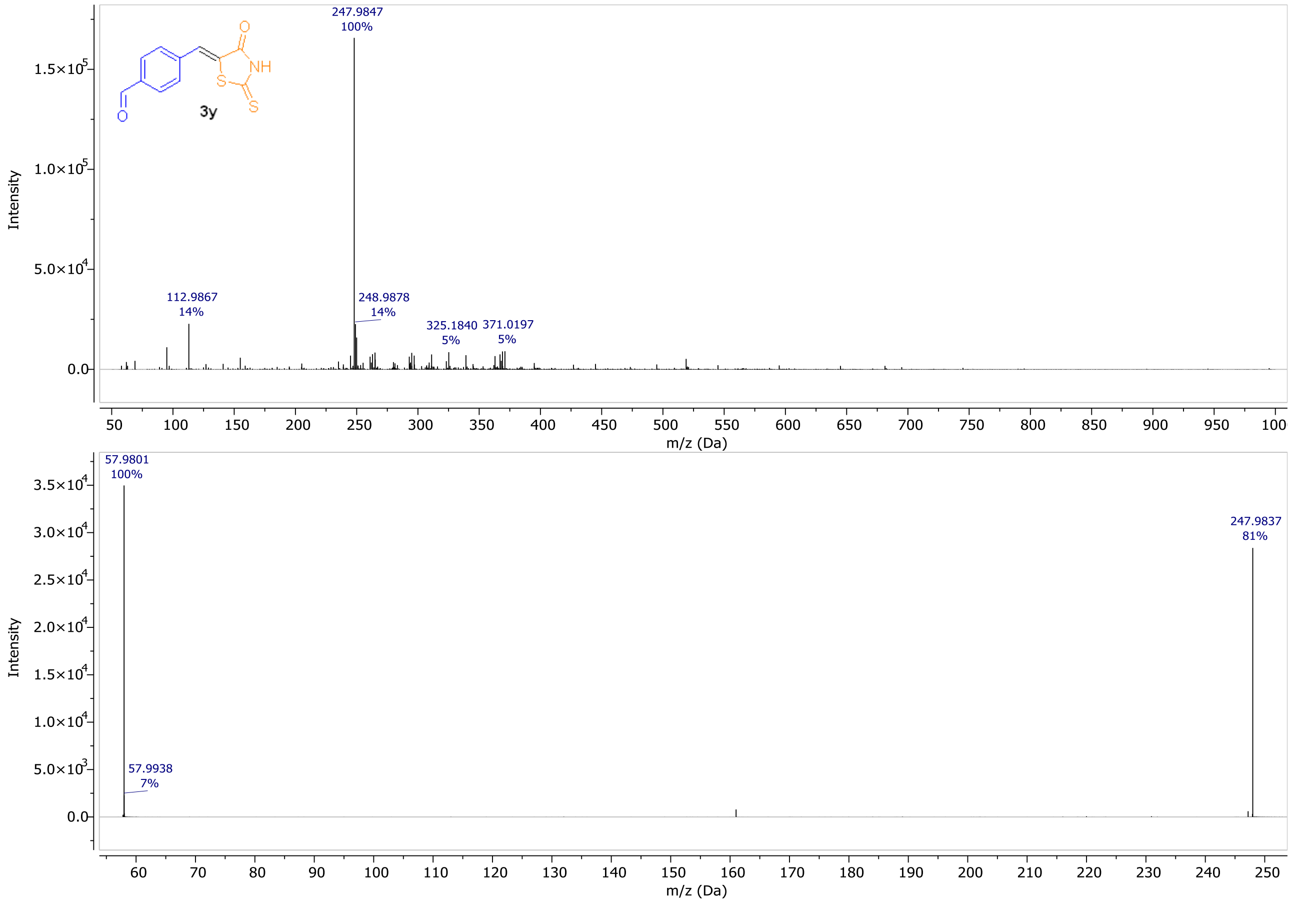

Figure S105. HRMS (ESI-QTOF) of compound **3y** and HRMS/MS for $[M-H]^-$.

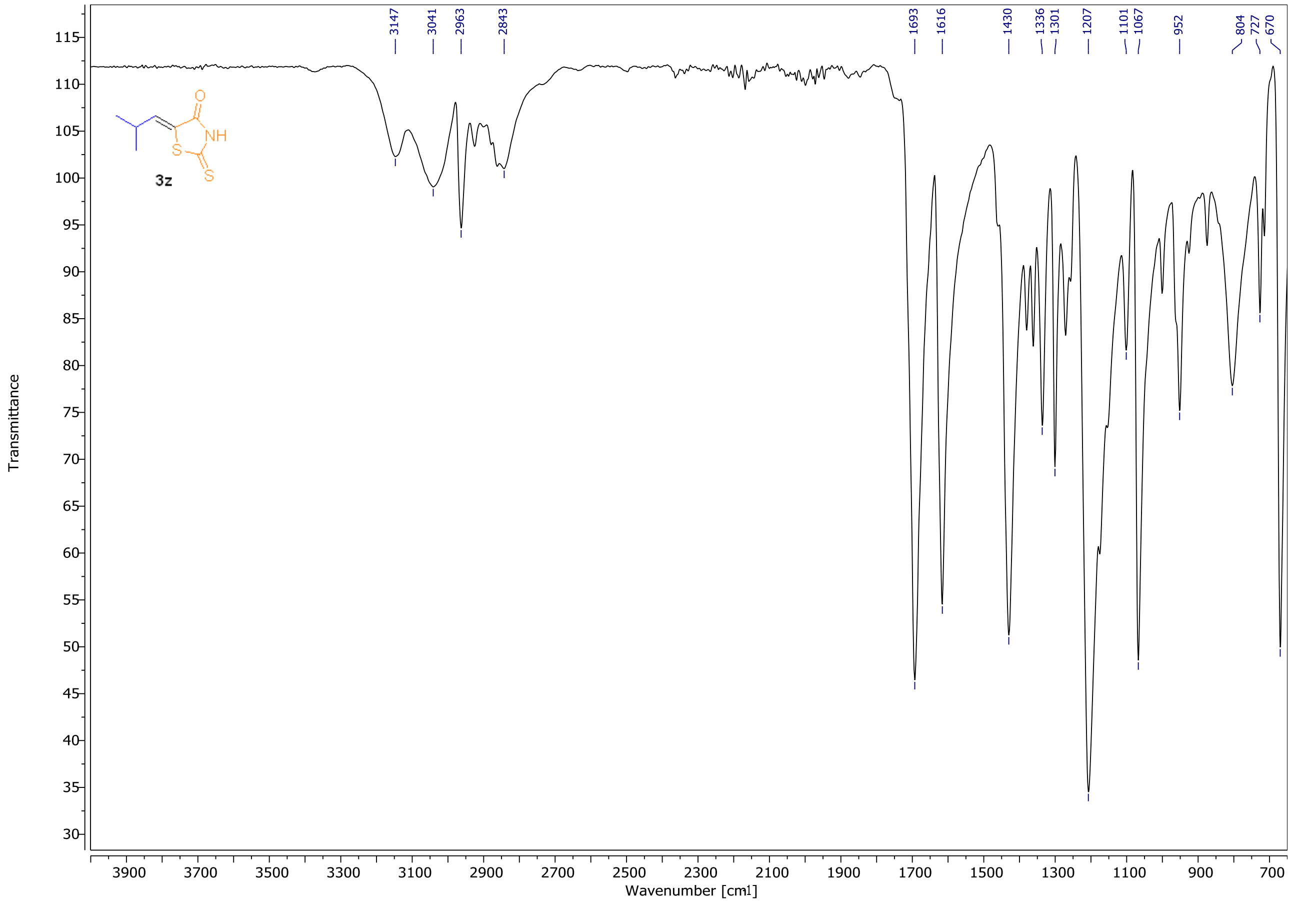

Figure S106. FTIR (ATR) of compound **3z**.

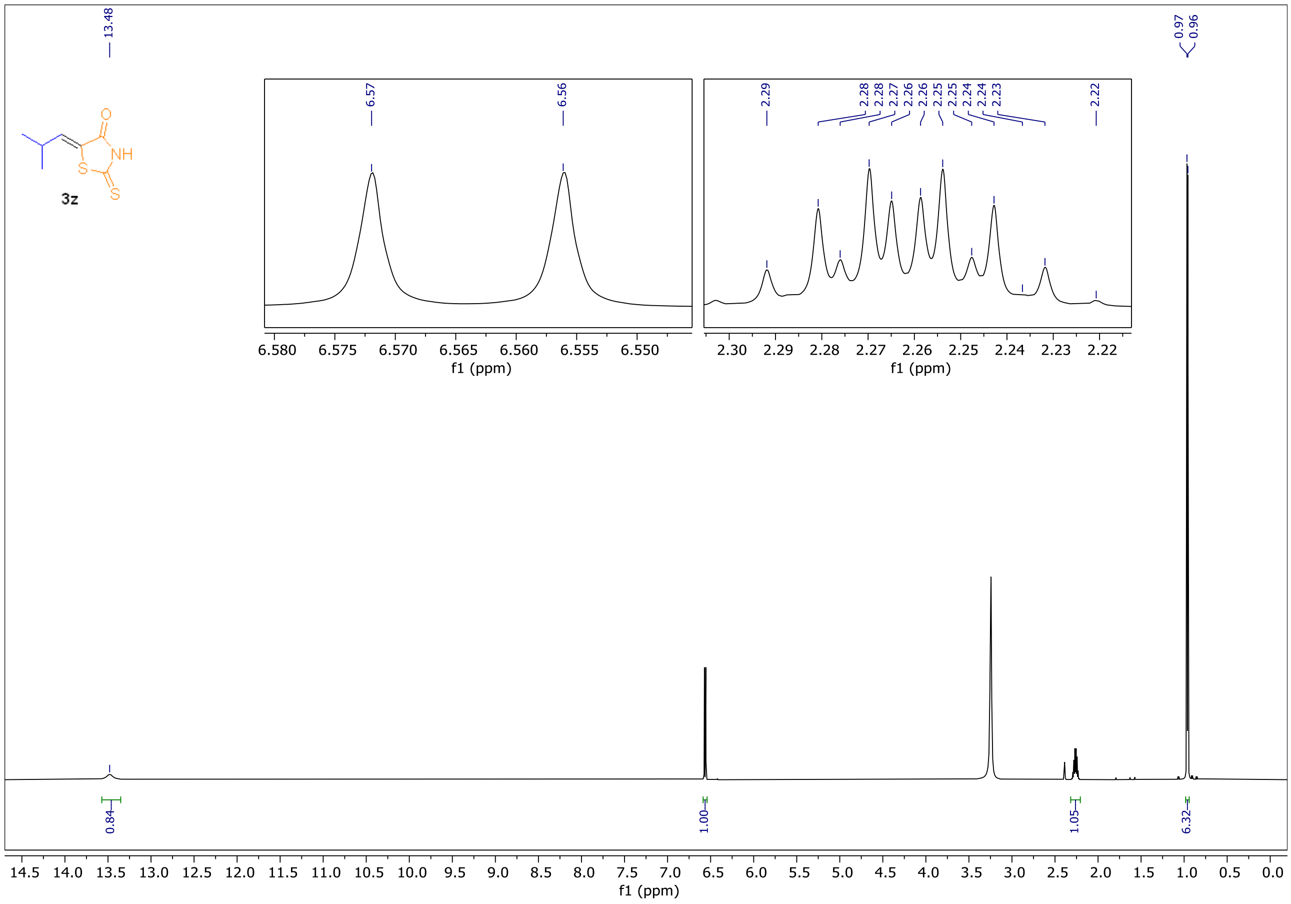

Figure S107. ^{1}H NMR spectrum (600 MHz, DMSO-d_6) of compound **3z**.

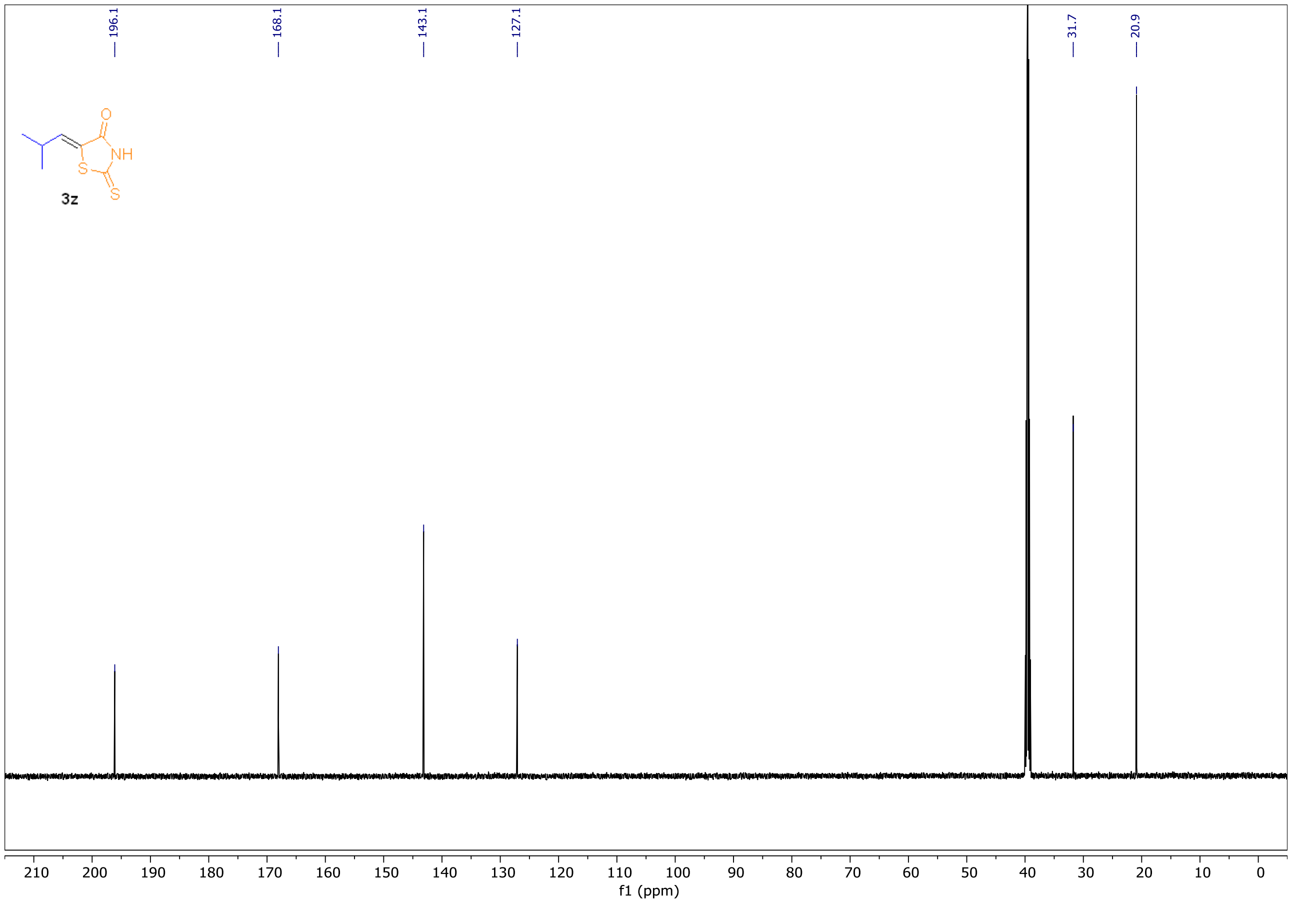

Figure S108. ^{13}C NMR spectrum (151 MHz, DMSO-d_6) of compound **3z**.

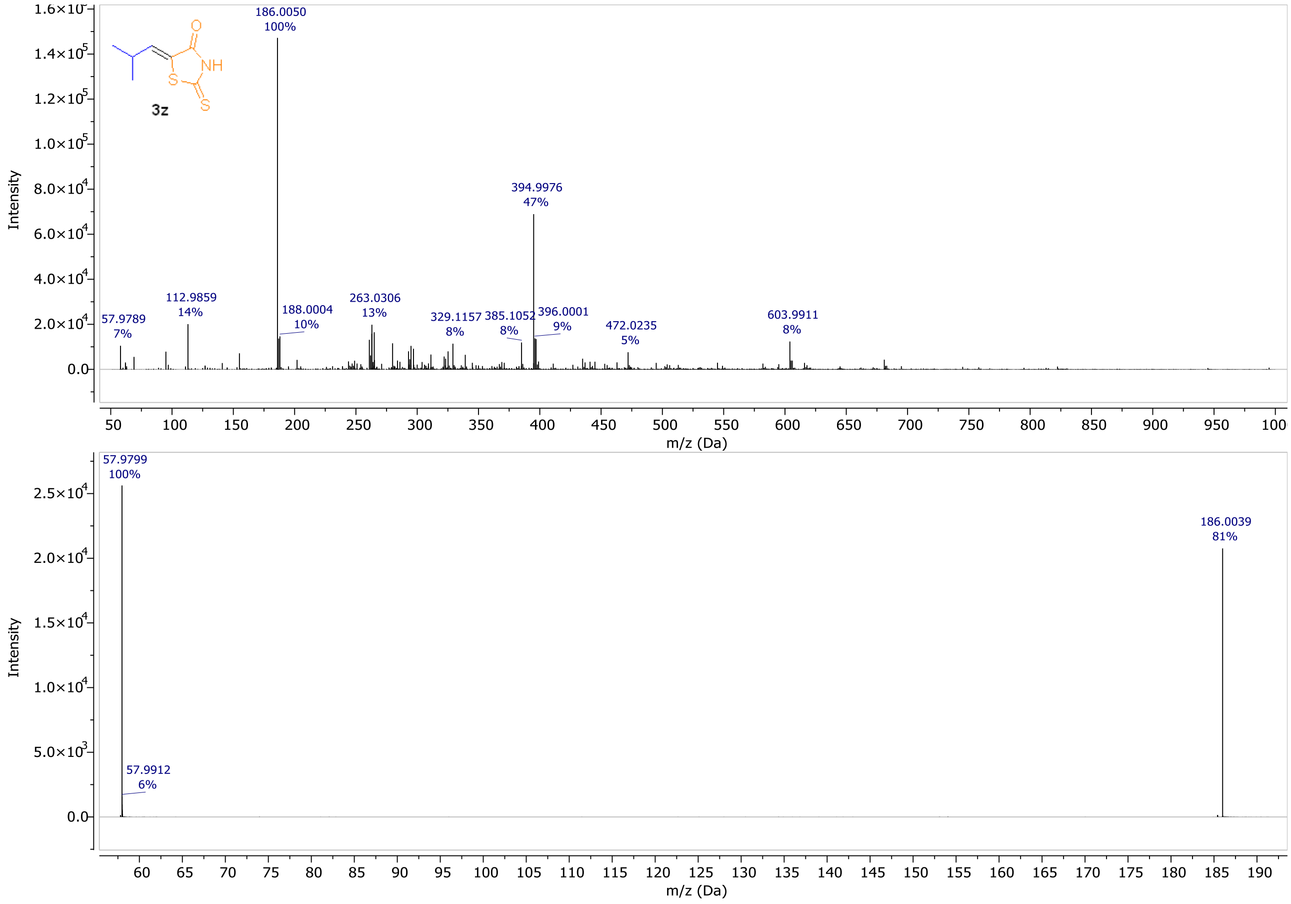

Figure S109. HRMS (ESI-QTOF) of compound **3z** and HRMS/MS for [M−H]$^-$.

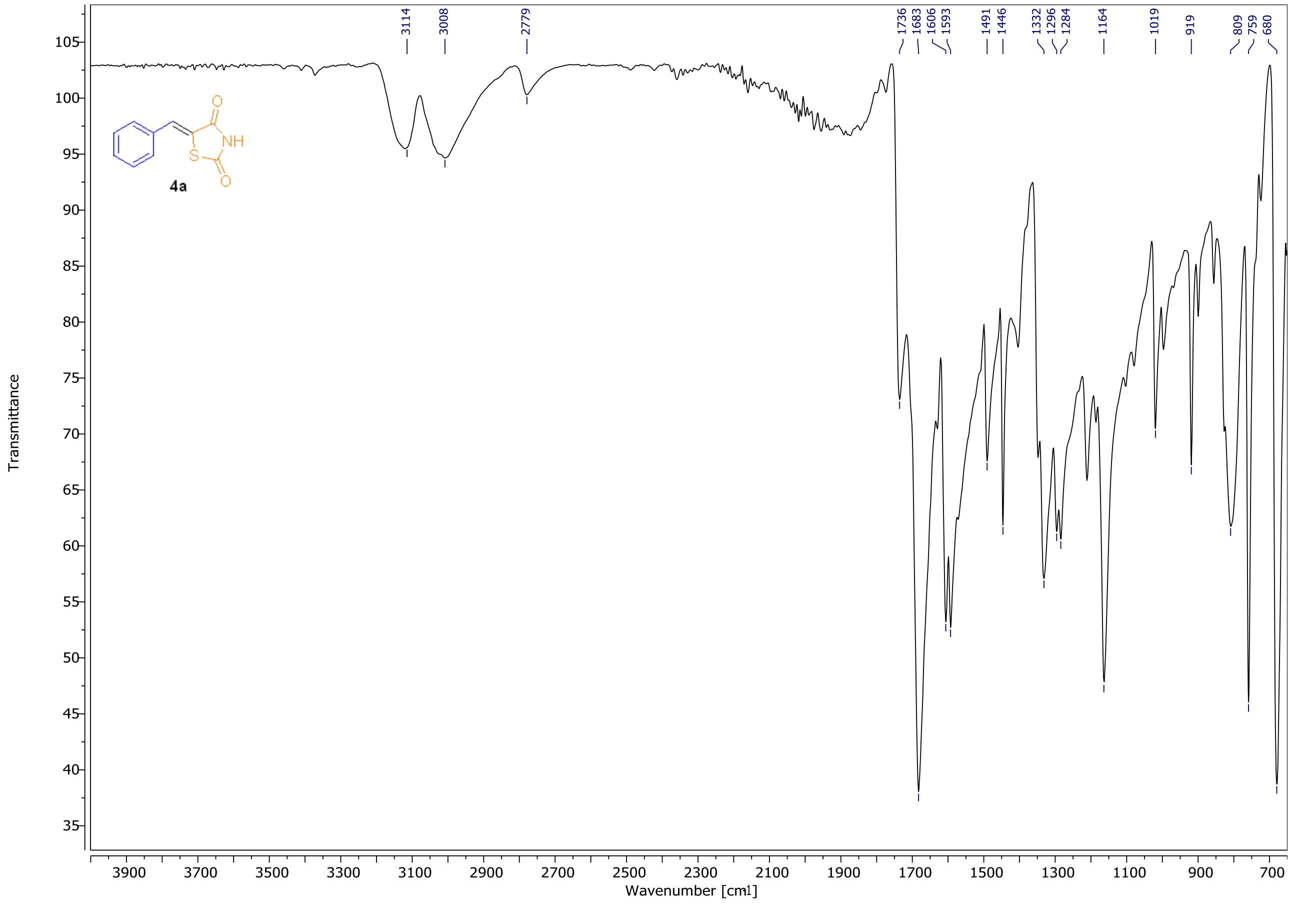

Figure S110. FTIR (ATR) of compound **4a**.

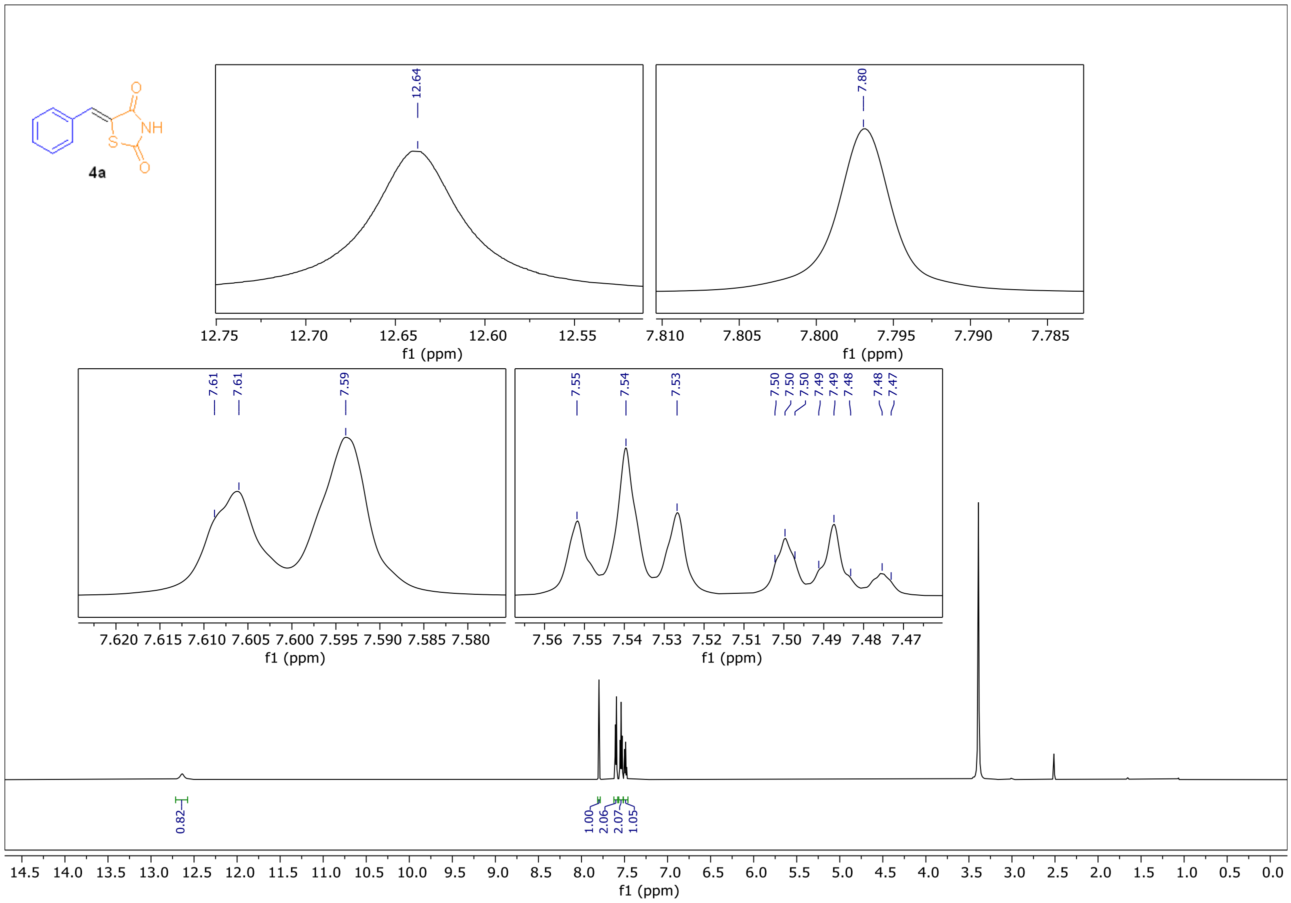

Figure S111. ^{1}H NMR spectrum (600 MHz, DMSO-d_6) of compound **4a**.

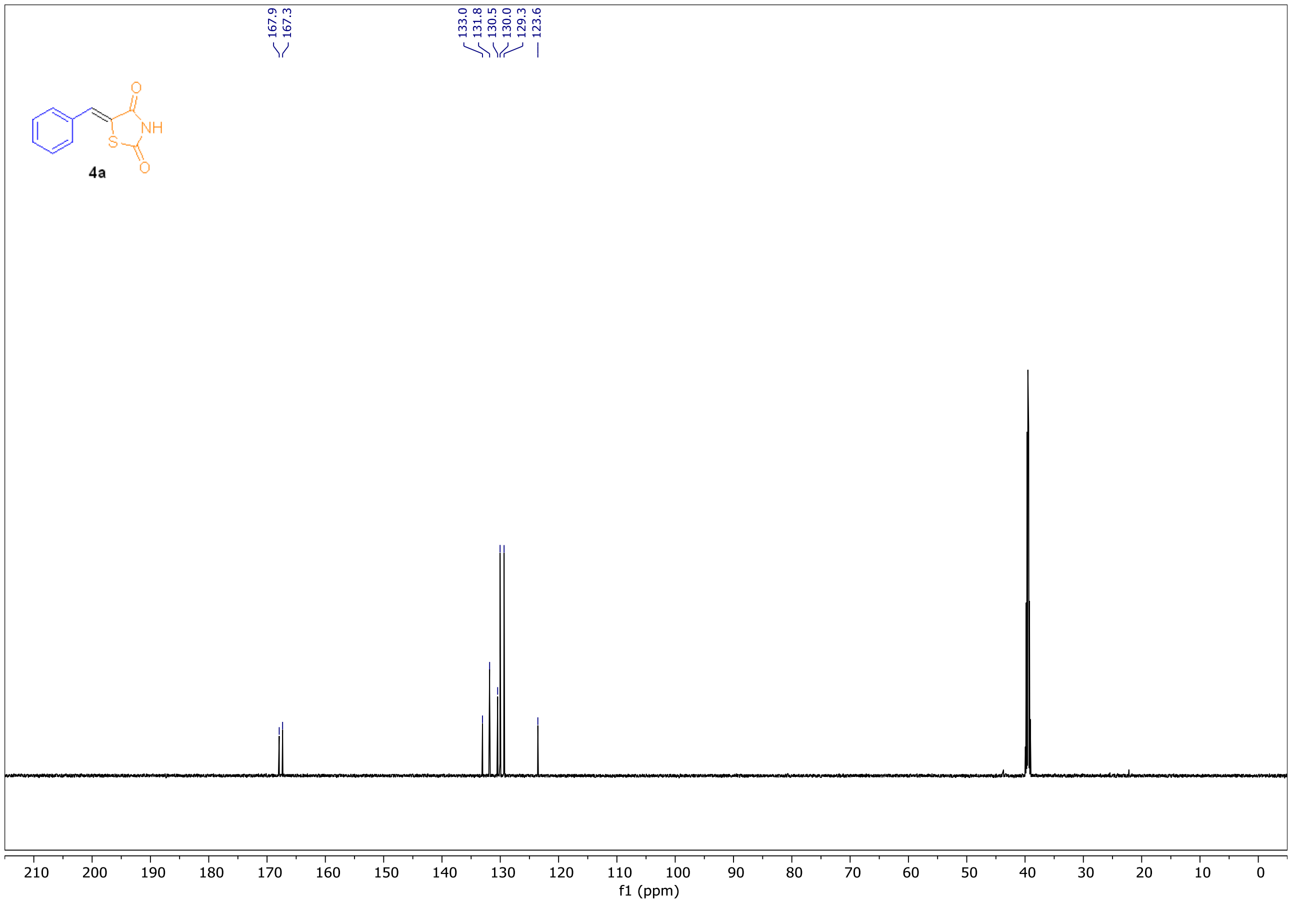

Figure S112. ^{13}C NMR spectrum (151 MHz, DMSO-d_6) of compound **4a**.

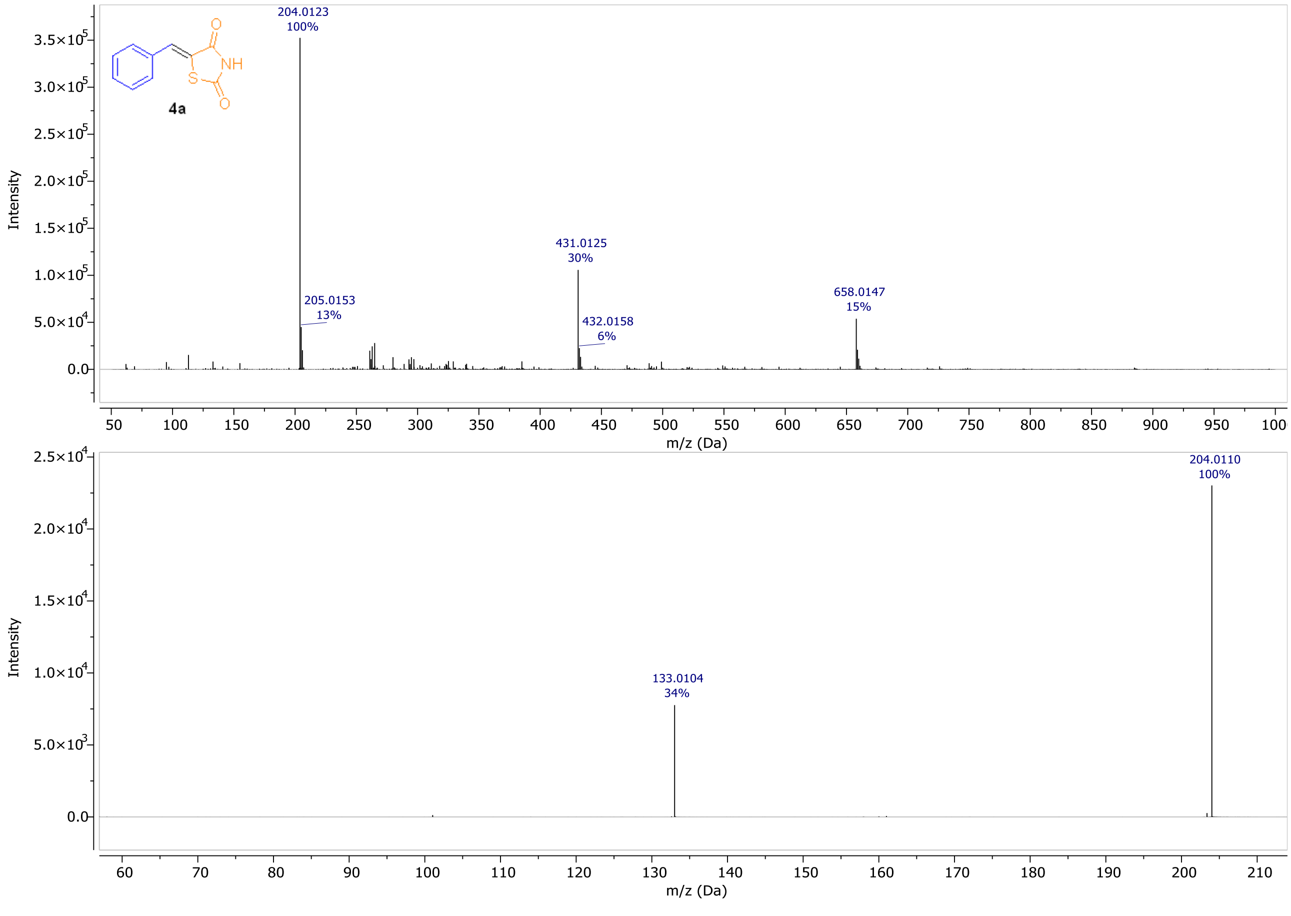

Figure S113. HRMS (ESI-QTOF) of compound **4a** and HRMS/MS for [M−H]$^-$.

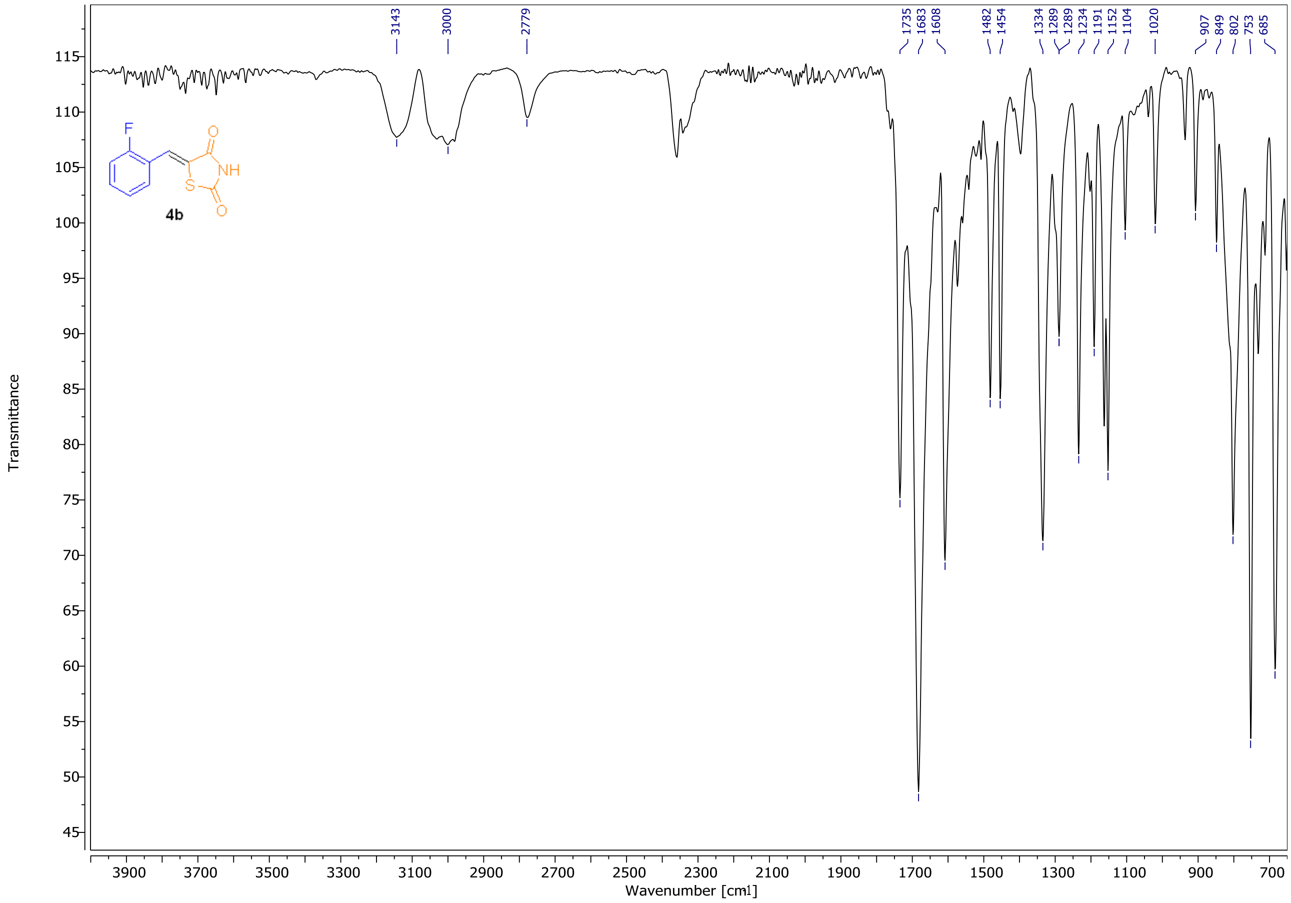

Figure S114. FTIR (ATR) of compound **4b**.

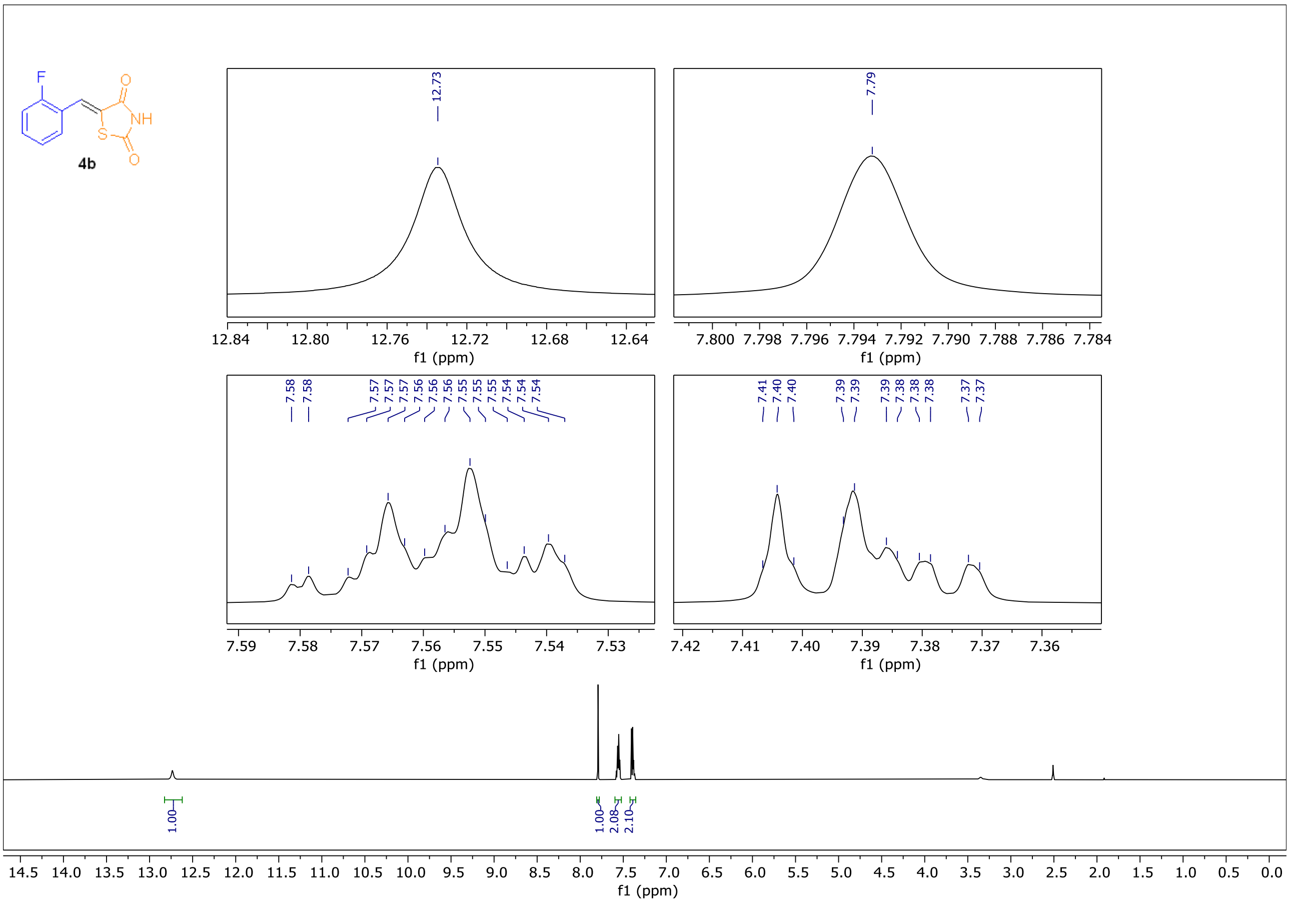

Figure S115. ^{1}H NMR spectrum (600 MHz, DMSO-d_6) of compound **4b**.

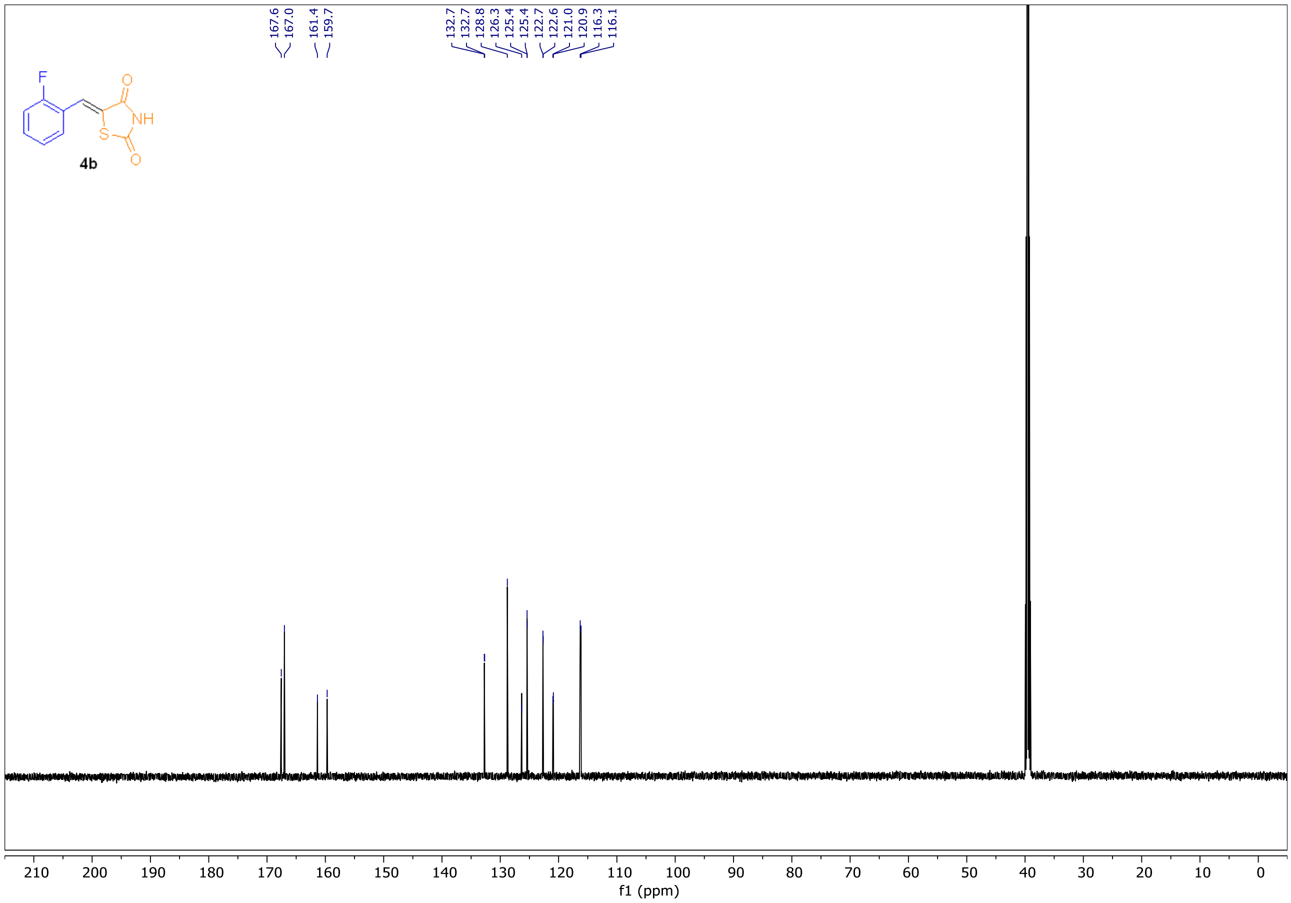

Figure S116. ^{13}C NMR spectrum (151 MHz, DMSO-d_6) of compound **4b**.

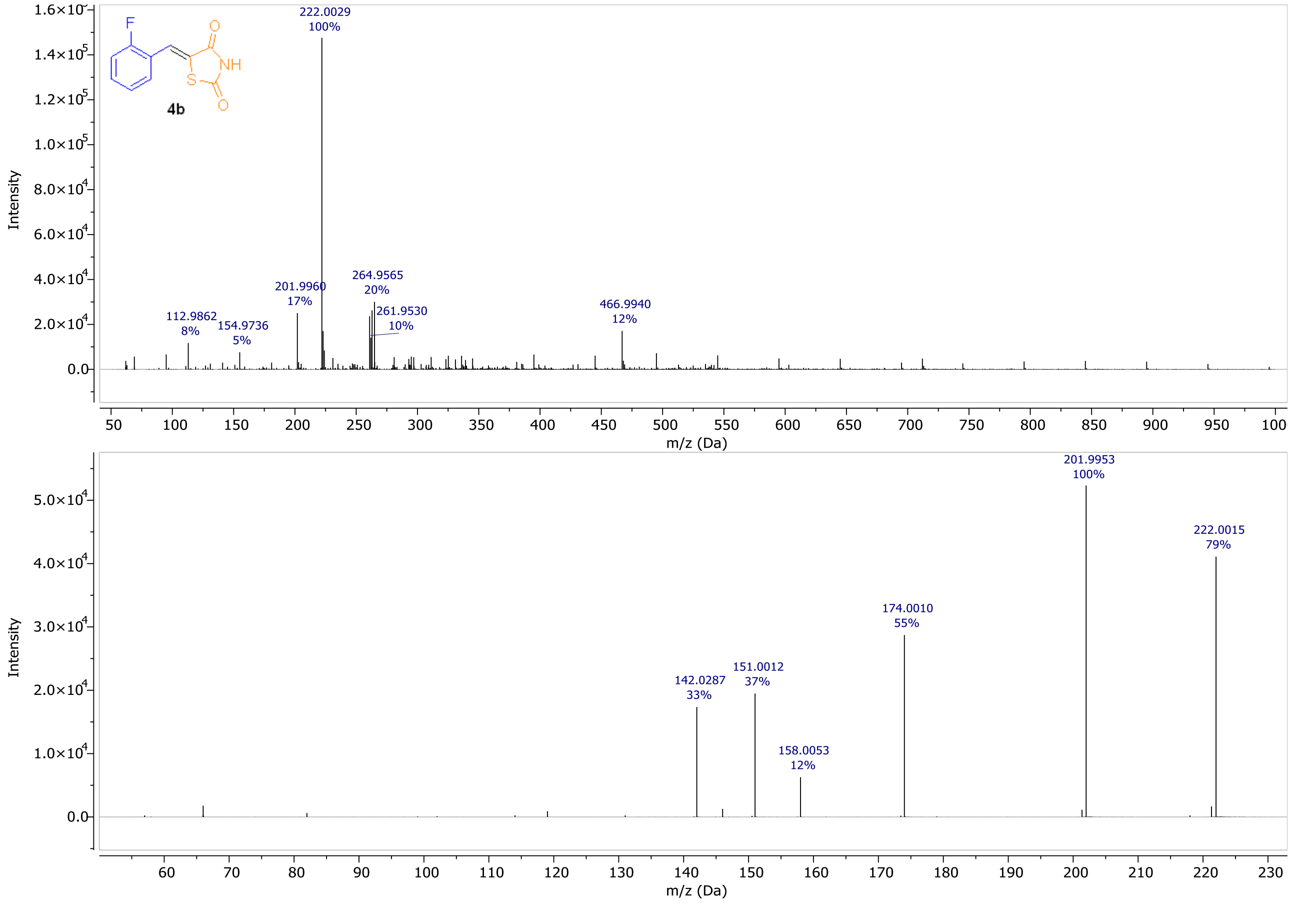

Figure S117. HRMS (ESI-QTOF) of compound **4b** and HRMS/MS for [M−H]⁻.

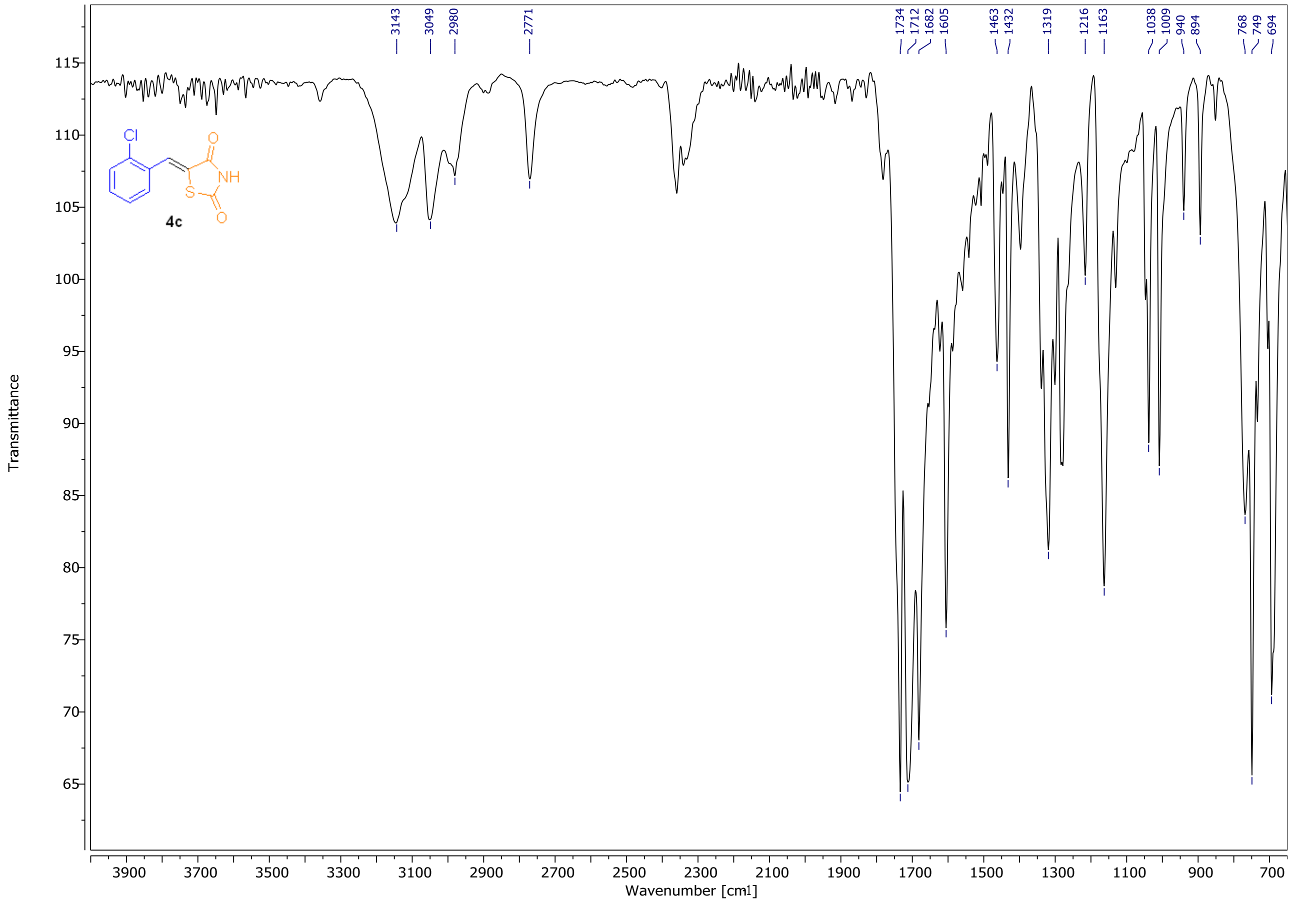

Figure S118. FTIR (ATR) of compound **4c**.

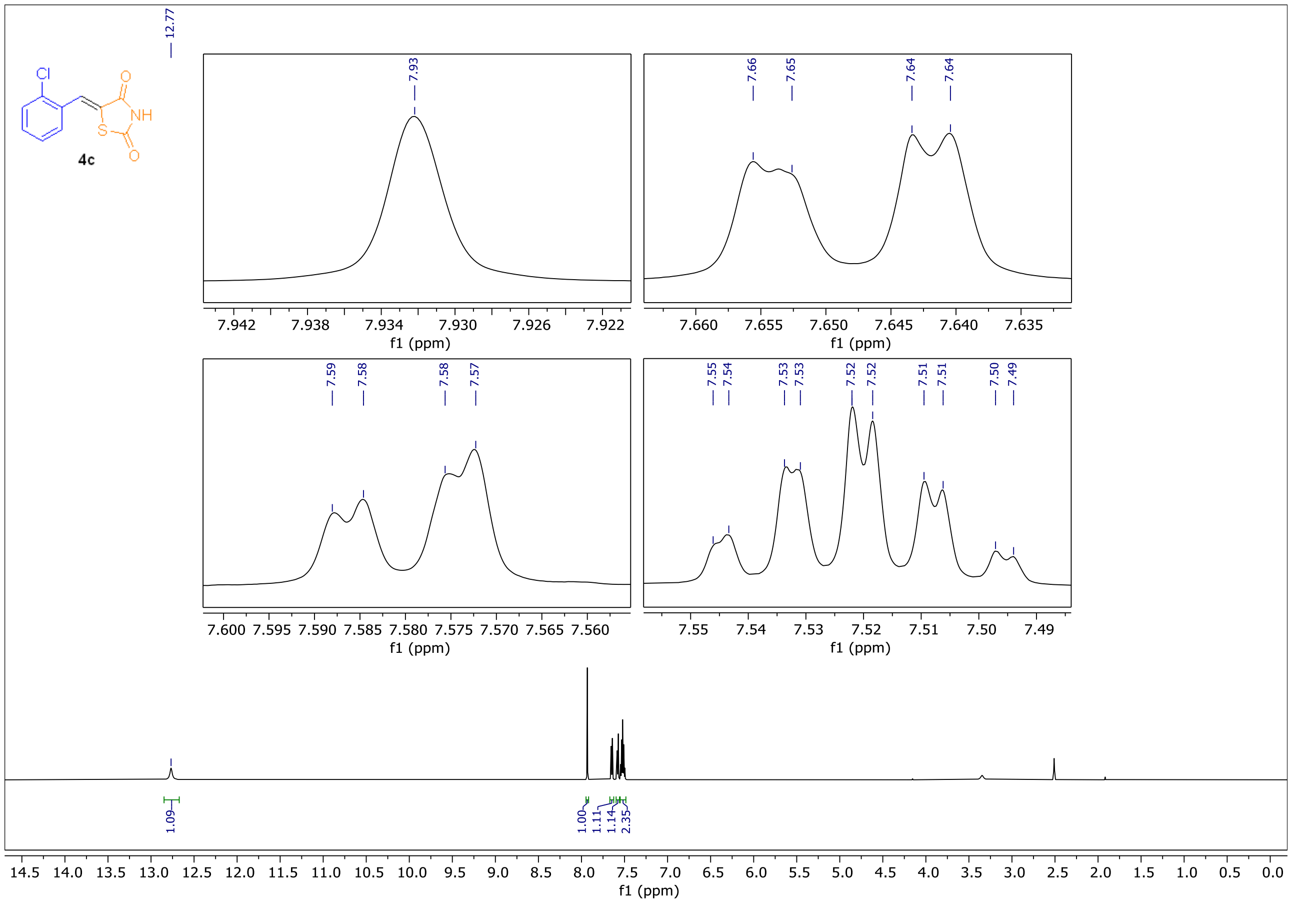

Figure S119. ^{1}H NMR spectrum (600 MHz, DMSO-d_6) of compound **4c**.

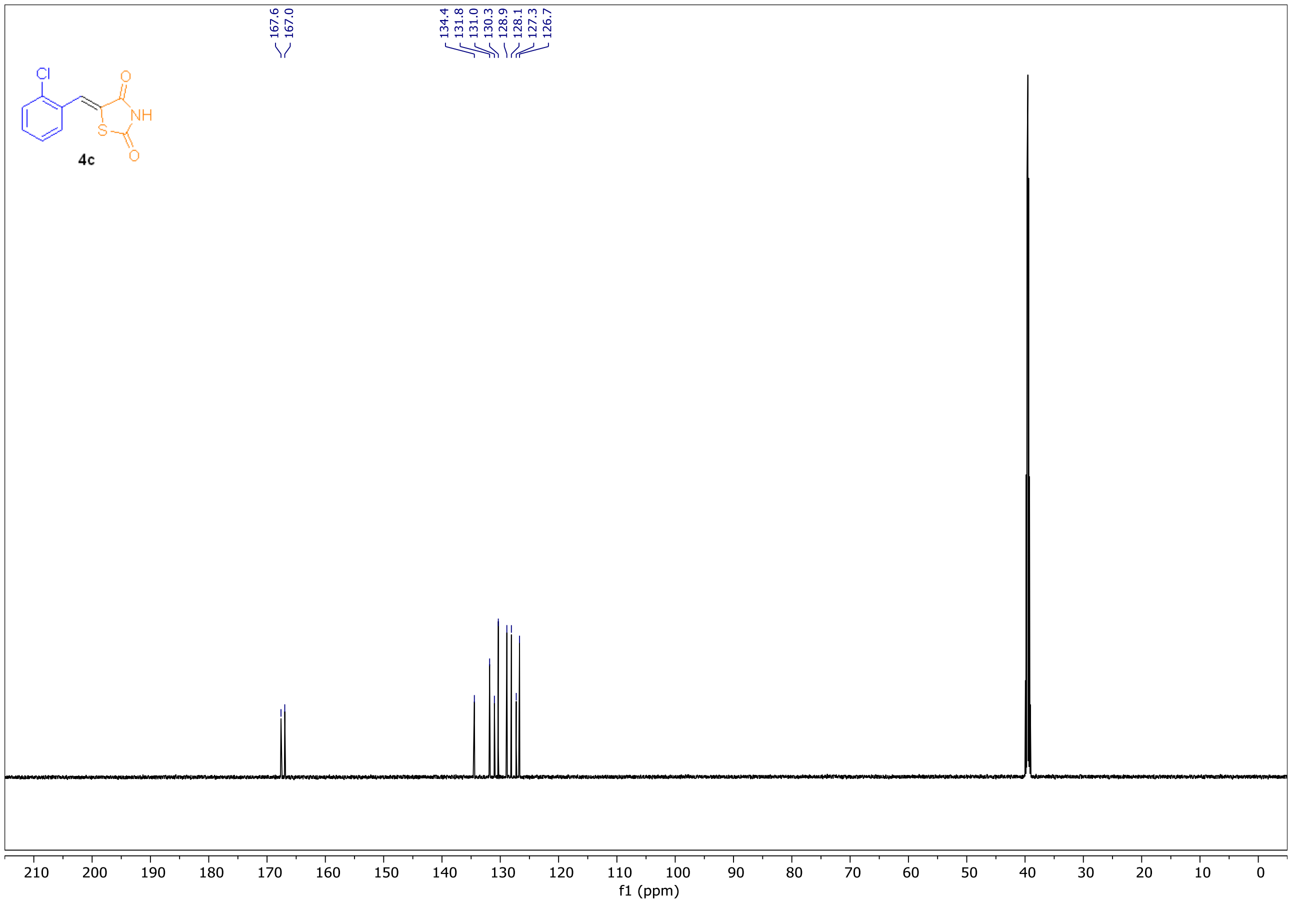

Figure S120. ^{13}C NMR spectrum (151 MHz, DMSO-d_6) of compound **4c**.

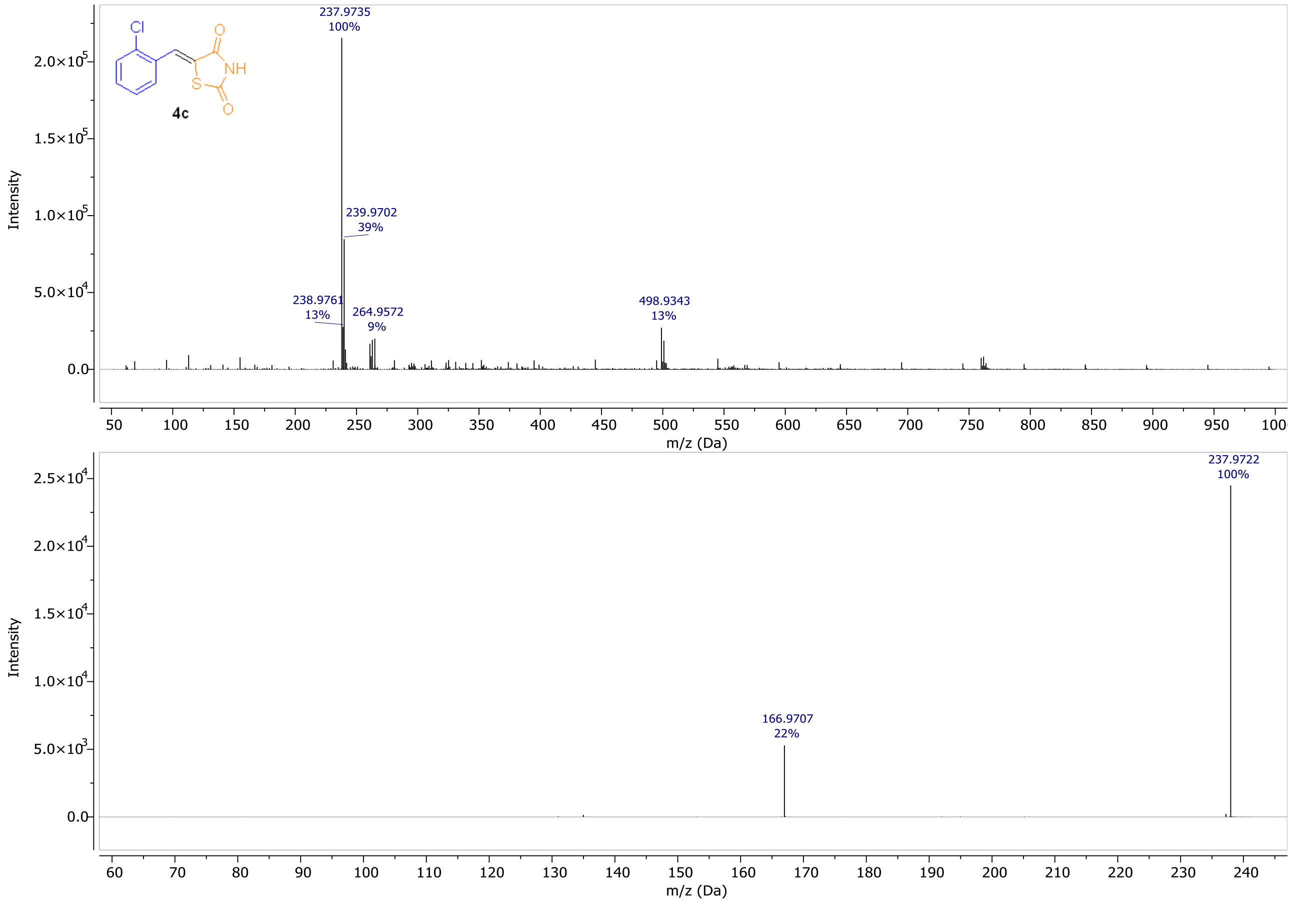

Figure S121. HRMS (ESI-QTOF) of compound **4c** and HRMS/MS for [M−H]⁻.

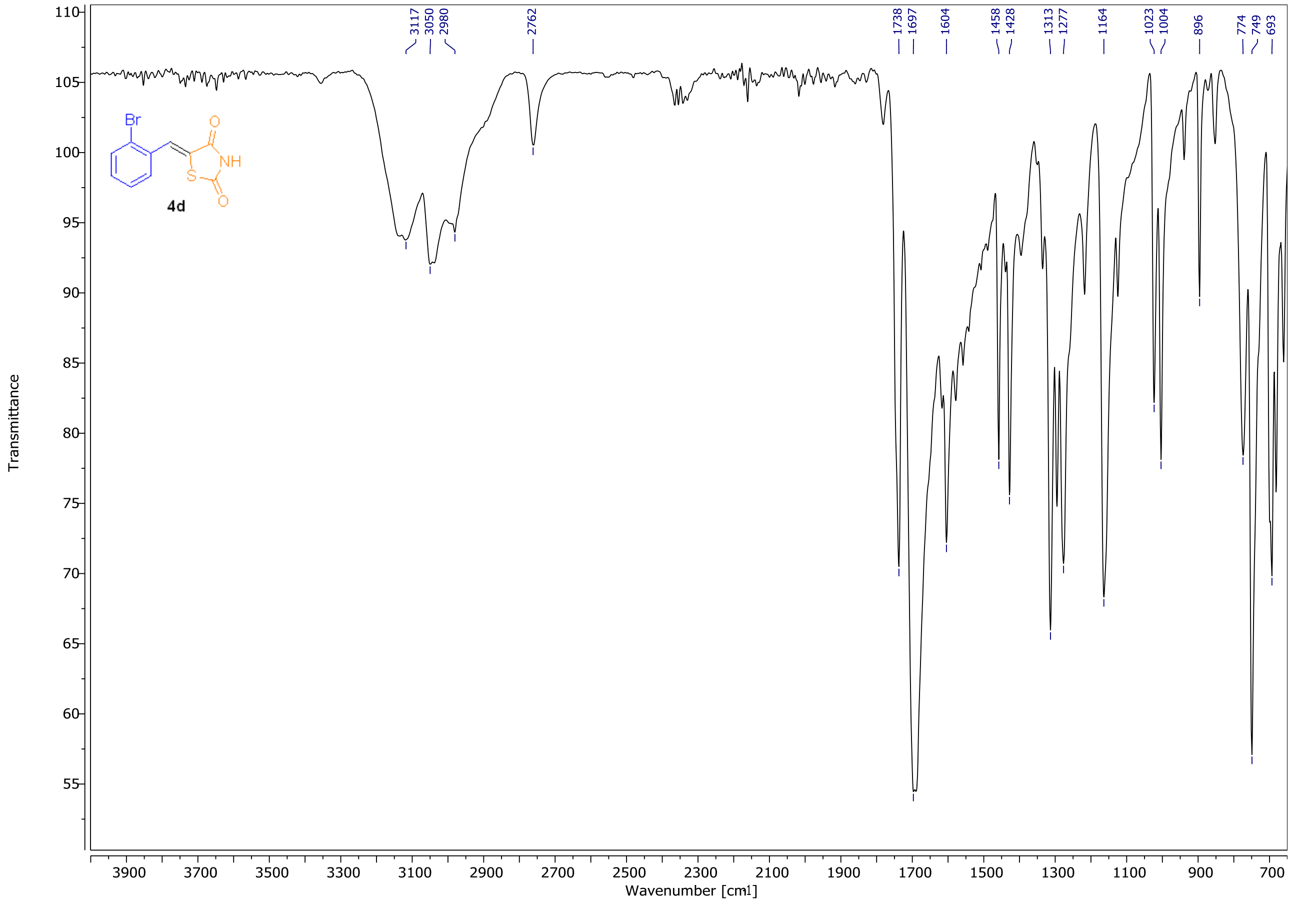

Figure S122. FTIR (ATR) of compound **4d**.

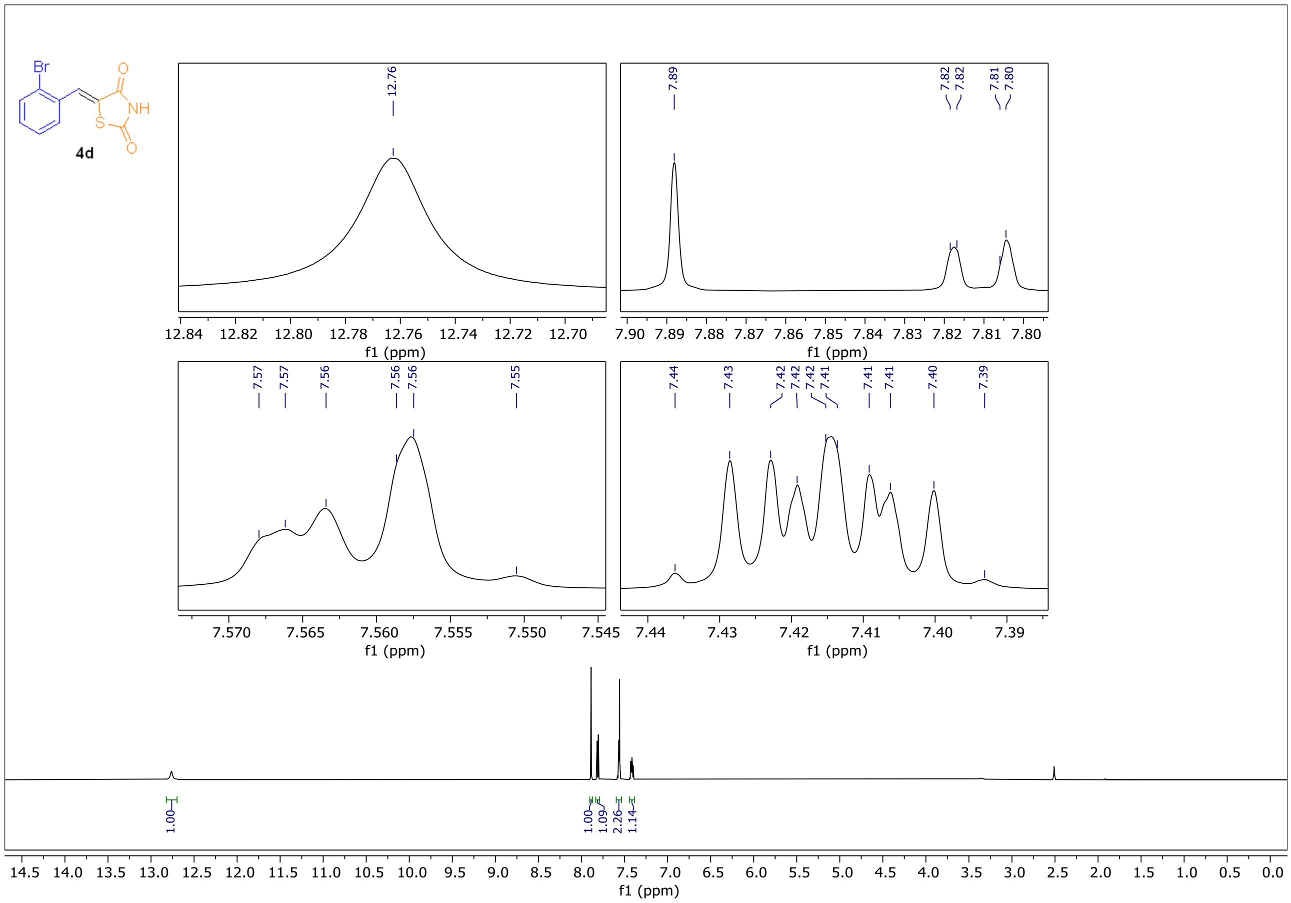

Figure S123. ^{1}H NMR spectrum (600 MHz, DMSO-d_6) of compound **4d**.

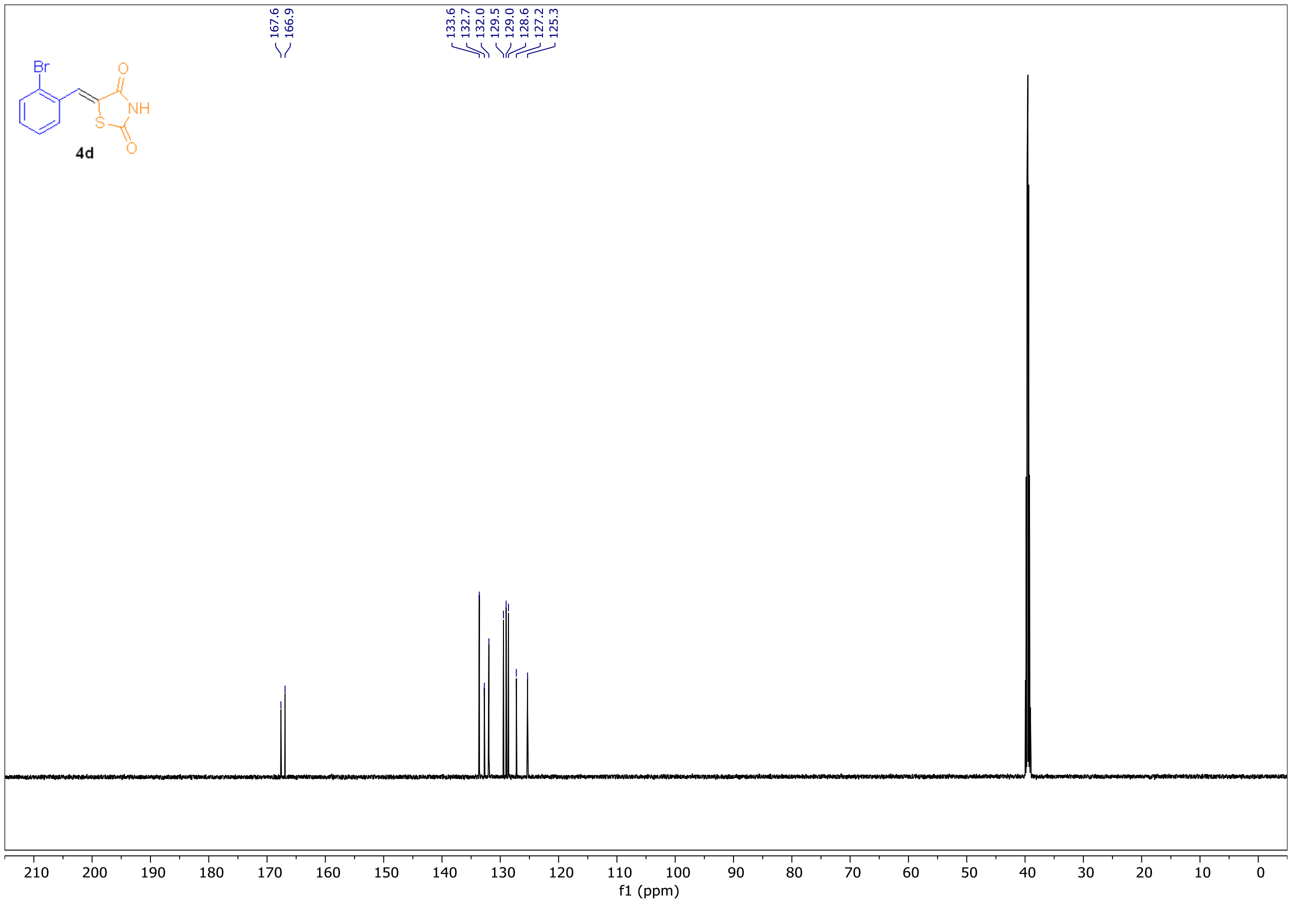

Figure S124. ^{13}C NMR spectrum (151 MHz, DMSO-d_6) of compound **4d**.

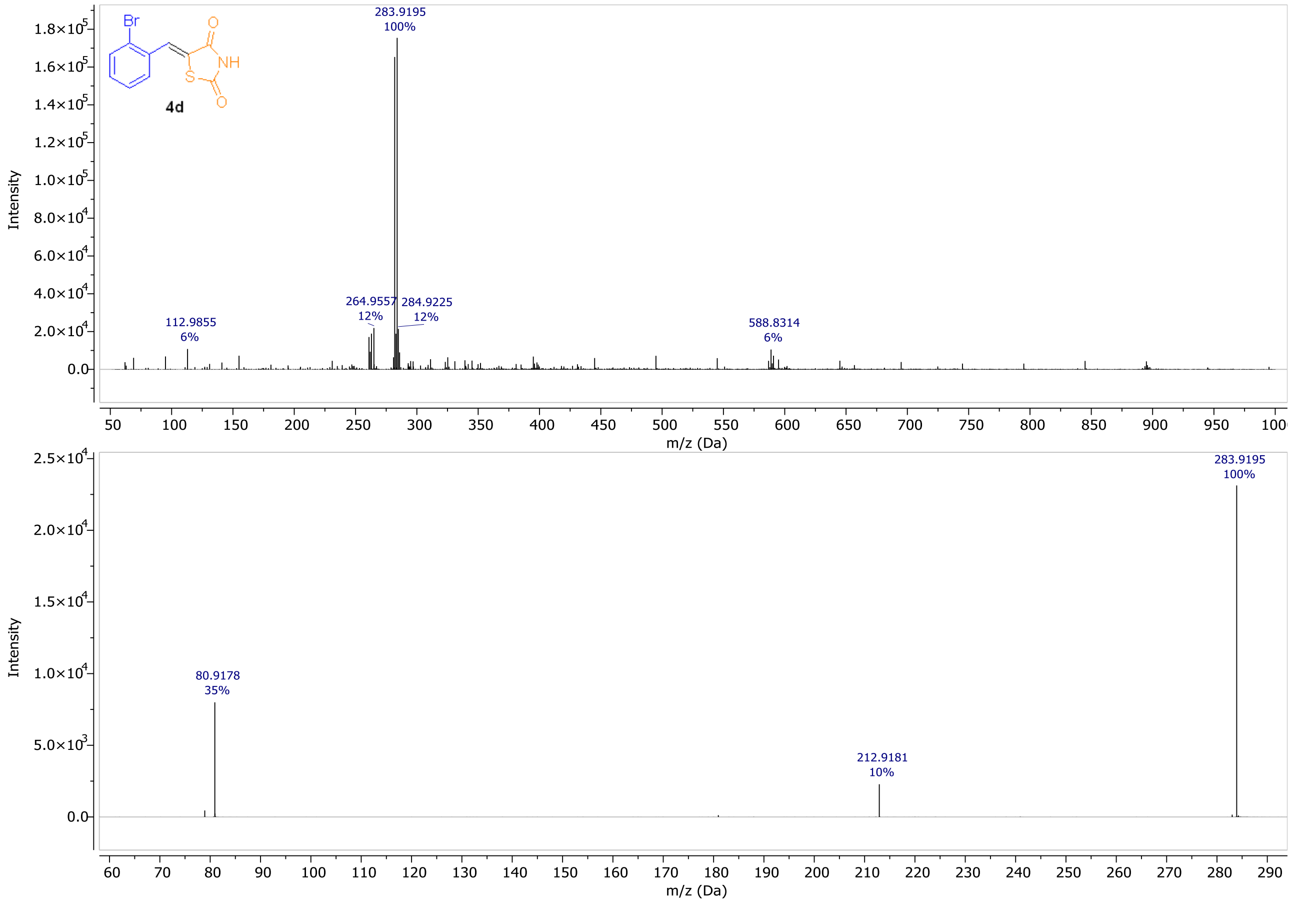

Figure S125. HRMS (ESI-QTOF) of compound **4d** and HRMS/MS for $[M-H]^-$.

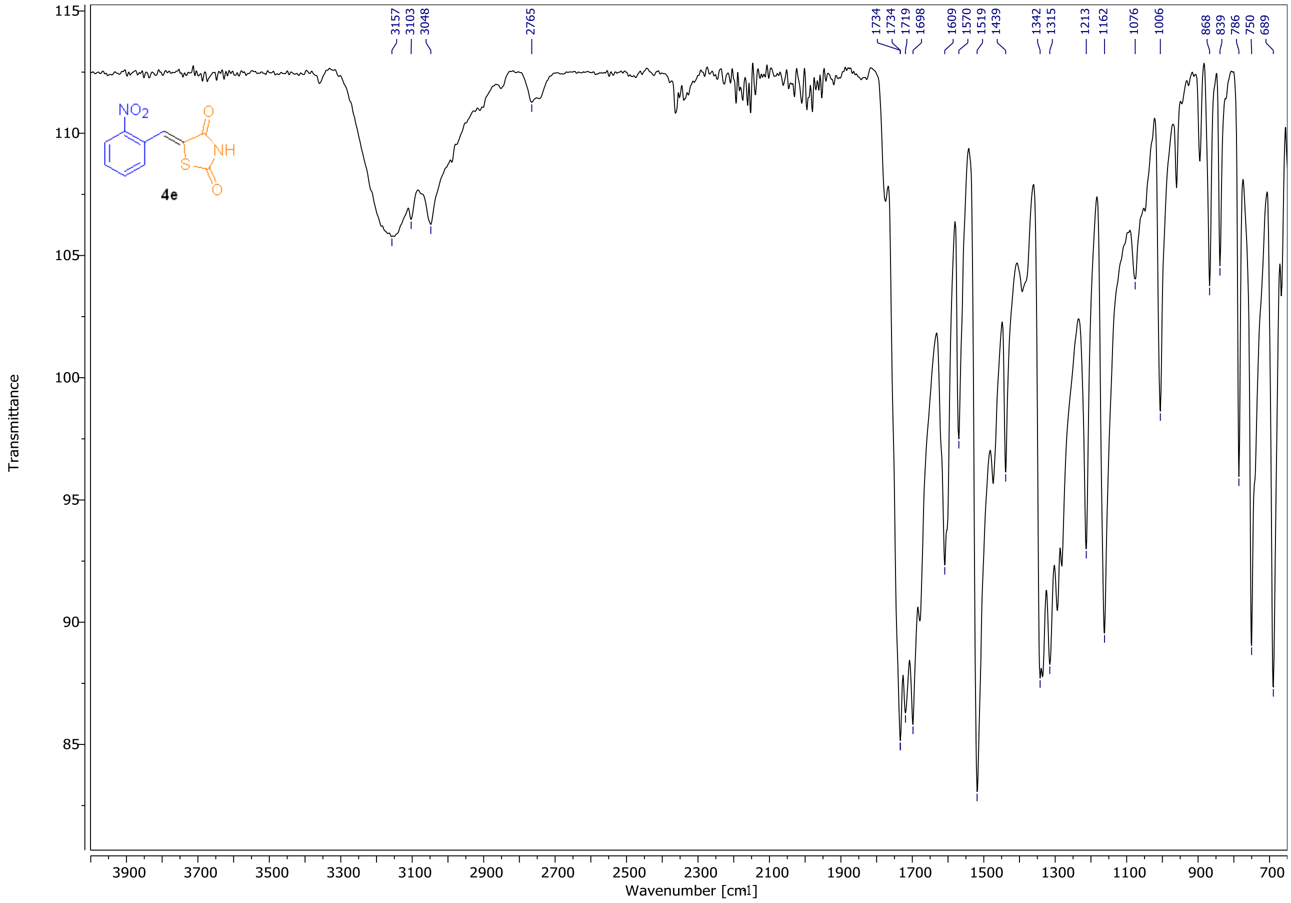

Figure S126. FTIR (ATR) of compound **4e**.

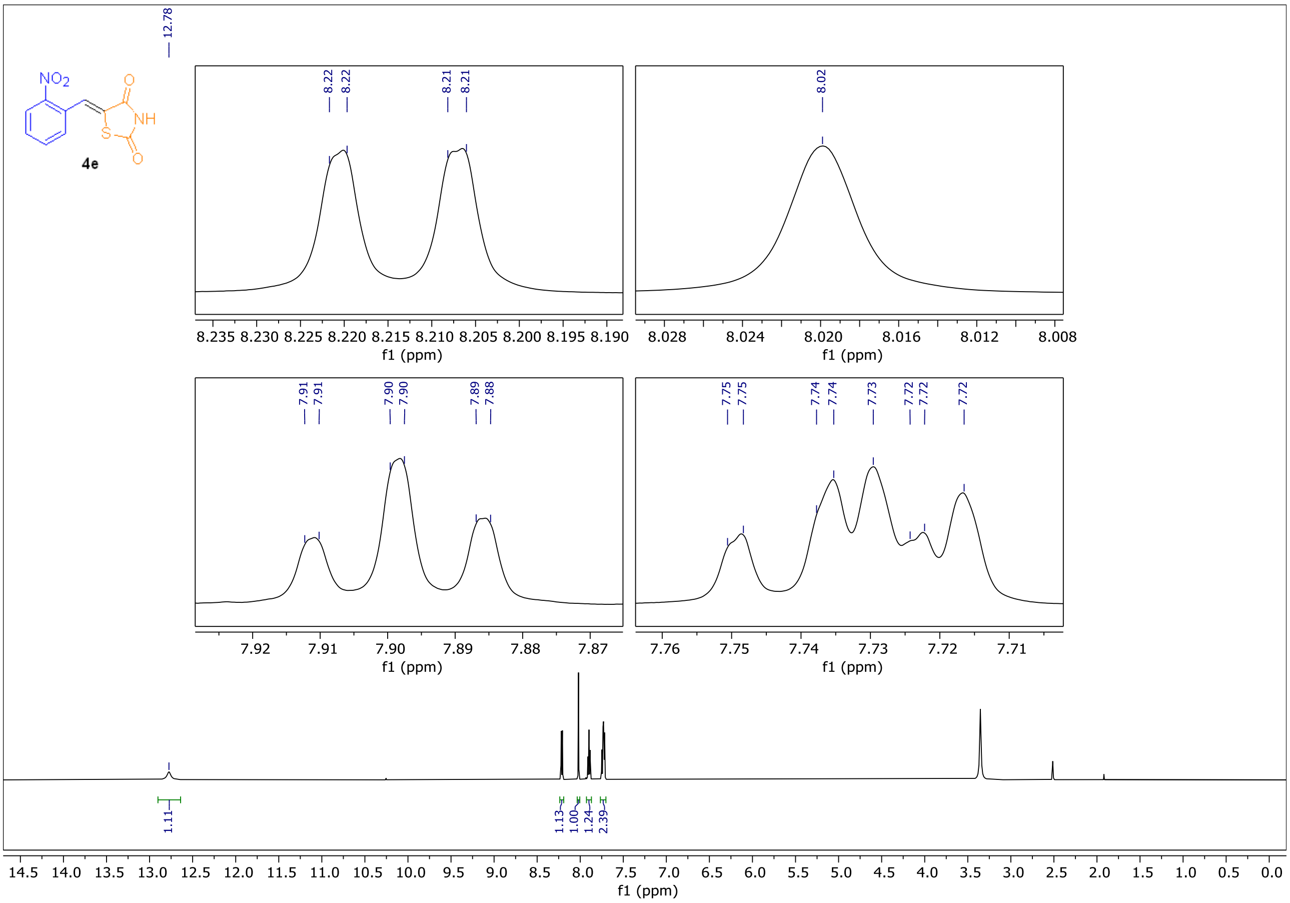

Figure S127. ^{1}H NMR spectrum (600 MHz, DMSO-d_6) of compound **4e**.

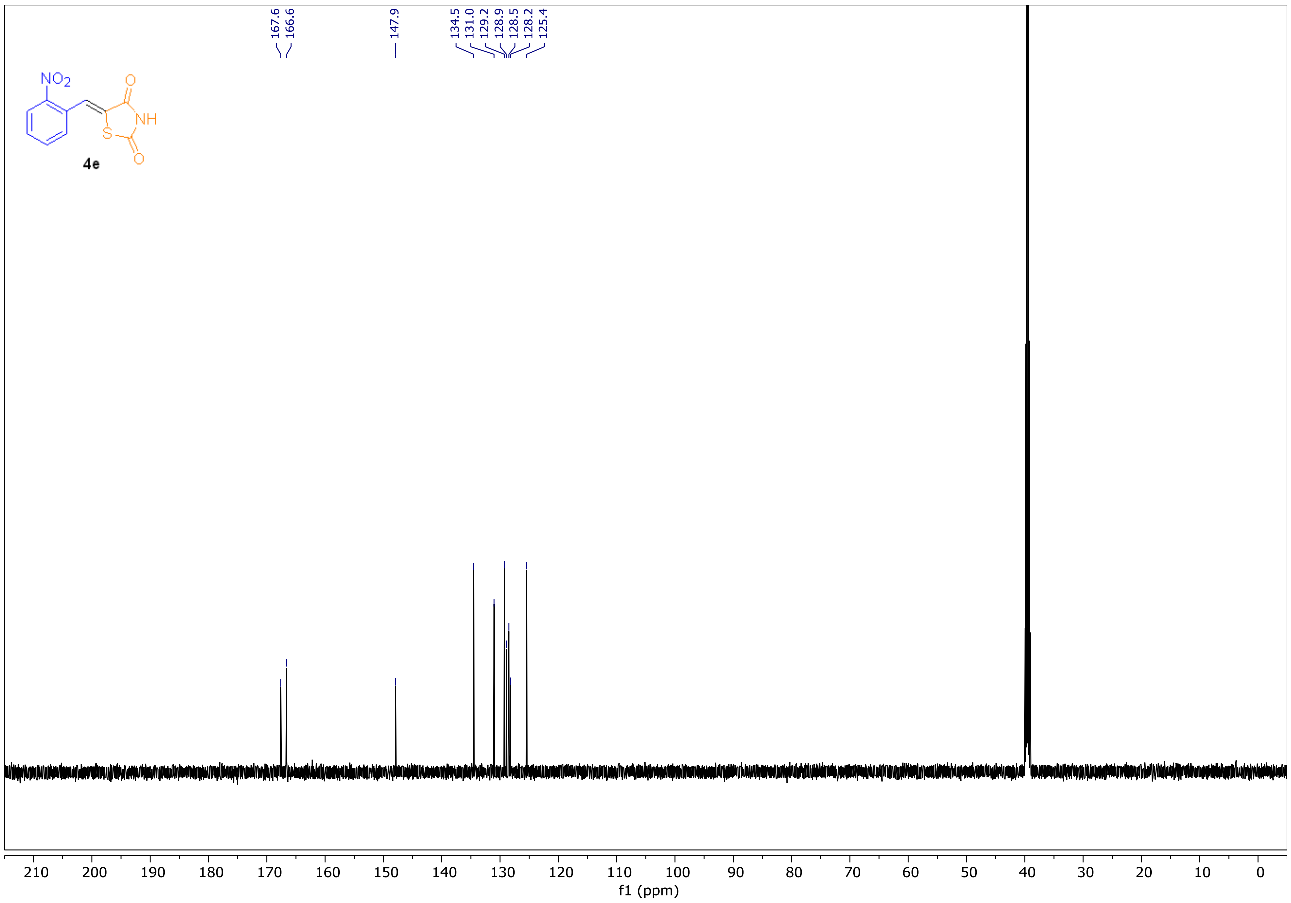

Figure S128. ^{13}C NMR spectrum (151 MHz, DMSO-d_6) of compound **4e**.

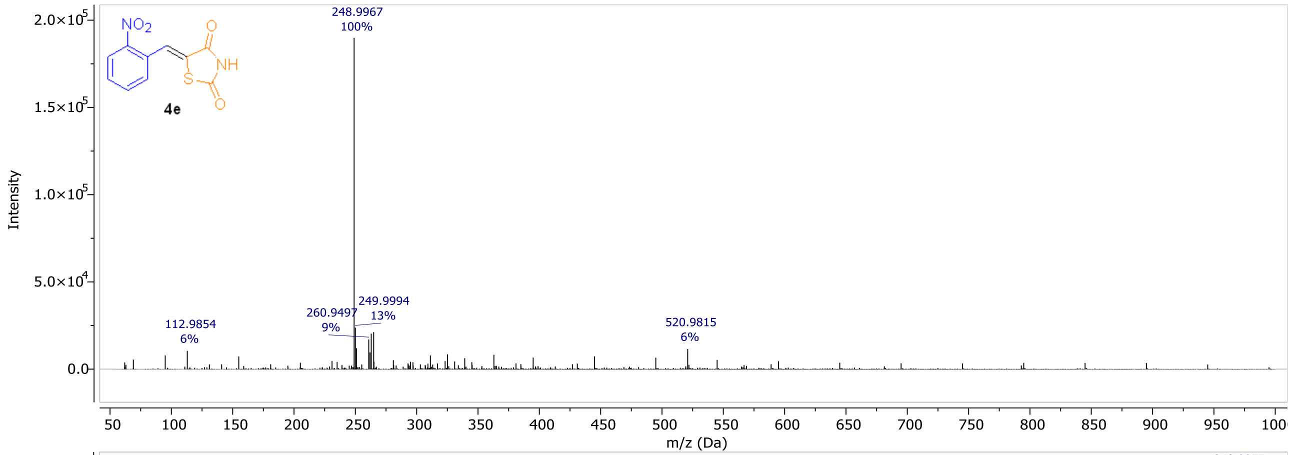

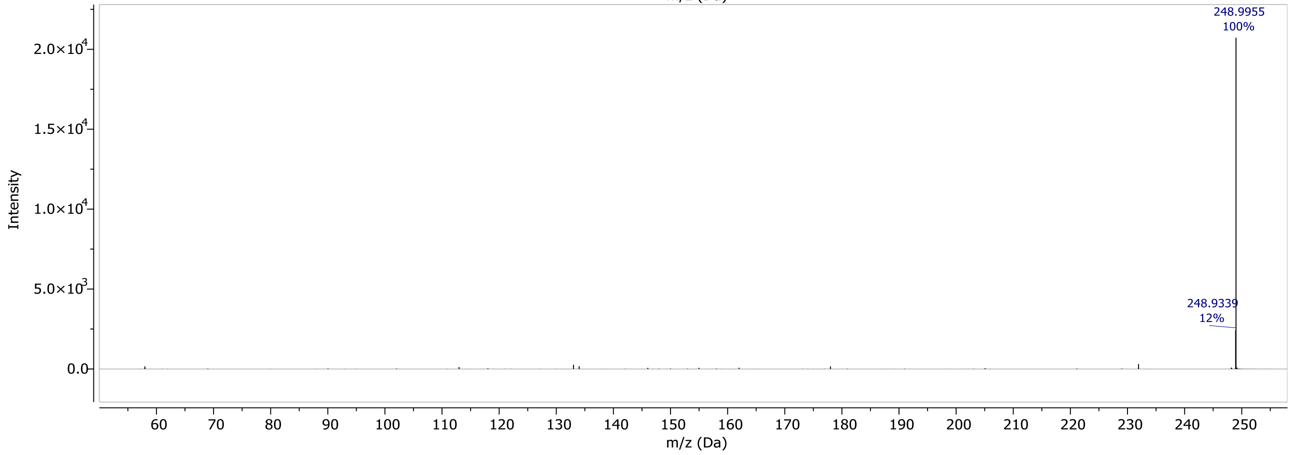

Figure S129. HRMS (ESI-QTOF) of compound **4e** and HRMS/MS for [M−H]$^{-}$.

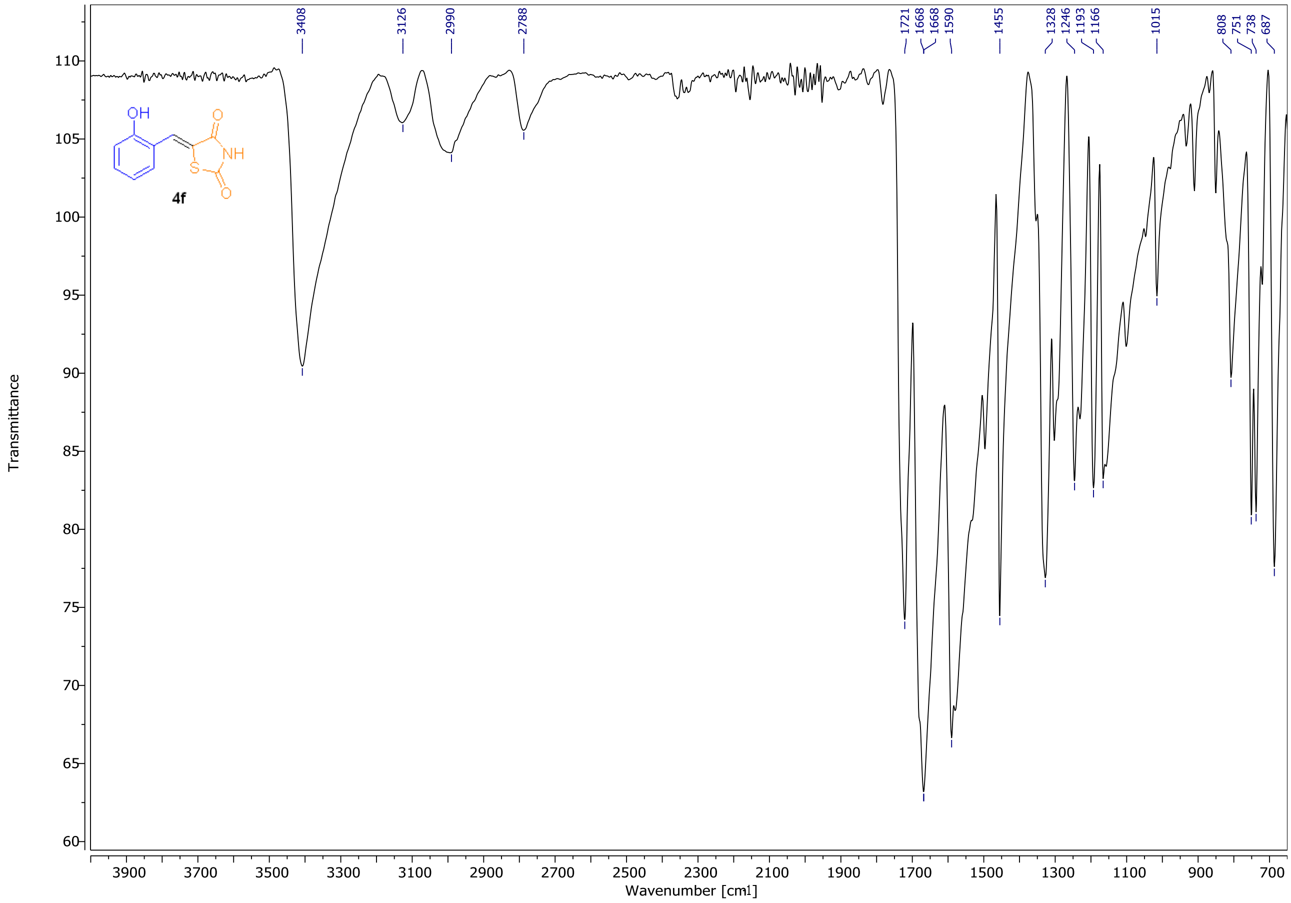

Figure S130. FTIR (ATR) of compound **4f**.

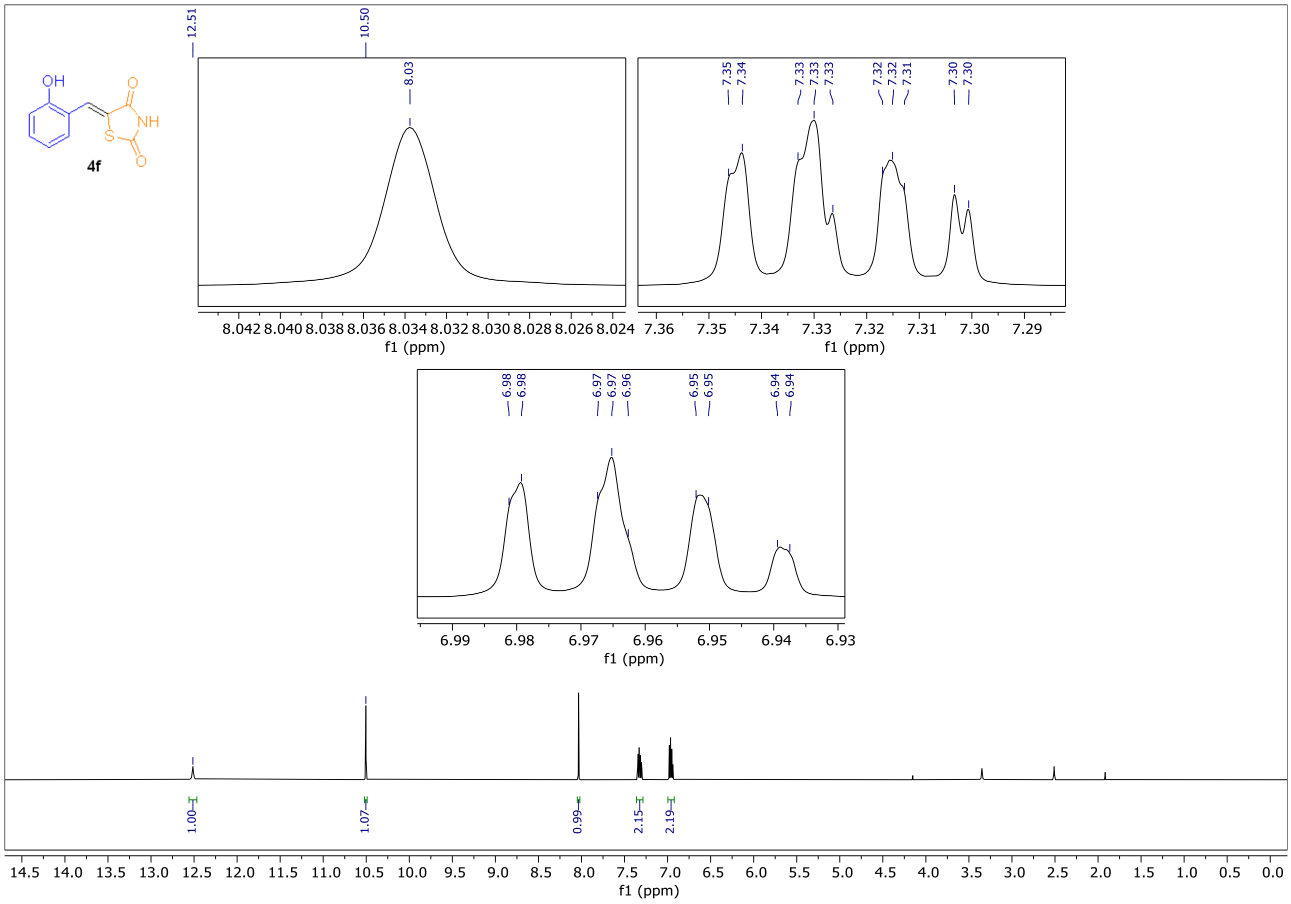

Figure S131. ^{1}H NMR spectrum (600 MHz, DMSO-d_6) of compound **4f**.

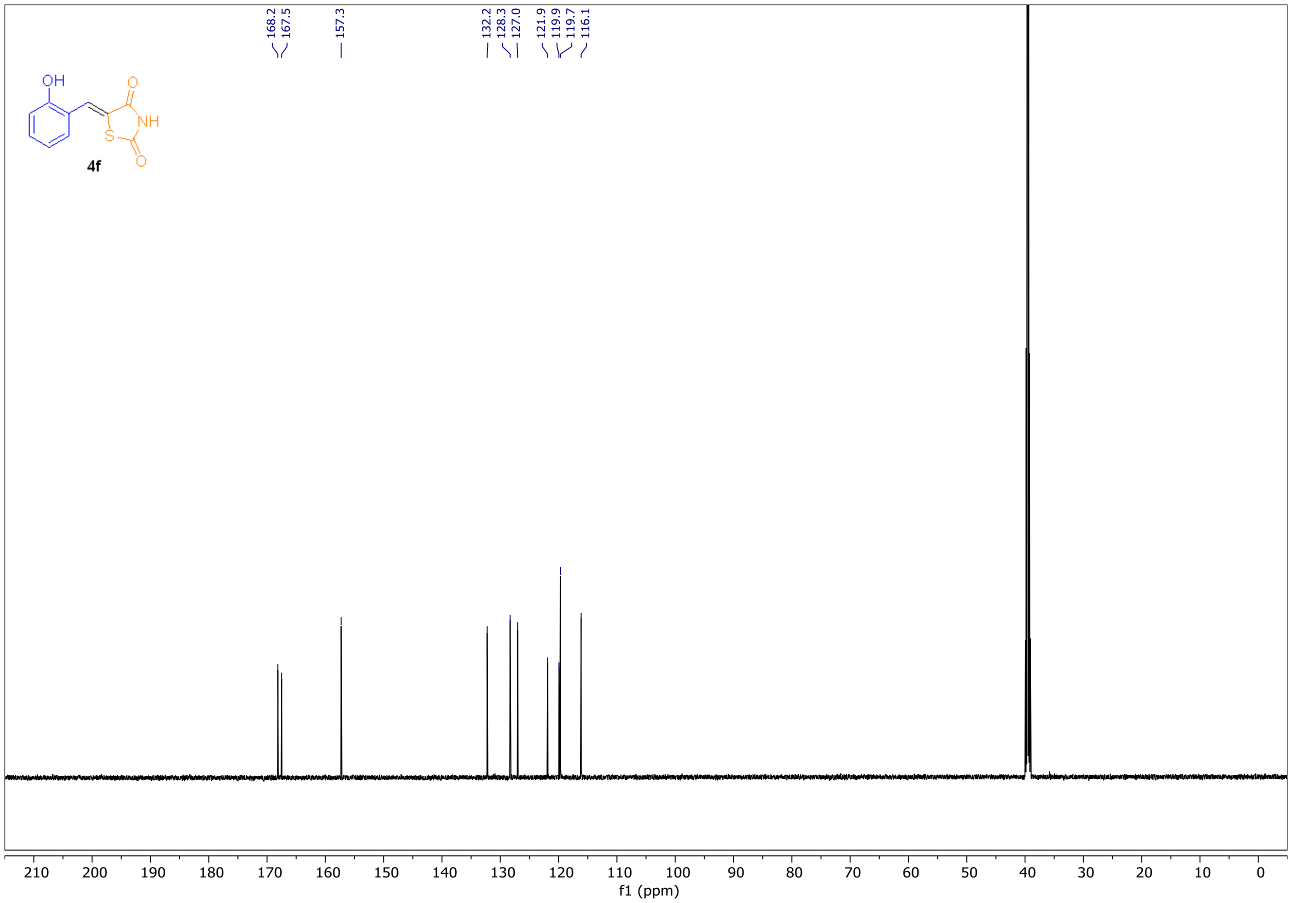

Figure S132. ^{13}C NMR spectrum (151 MHz, DMSO-d_6) of compound **4f**.

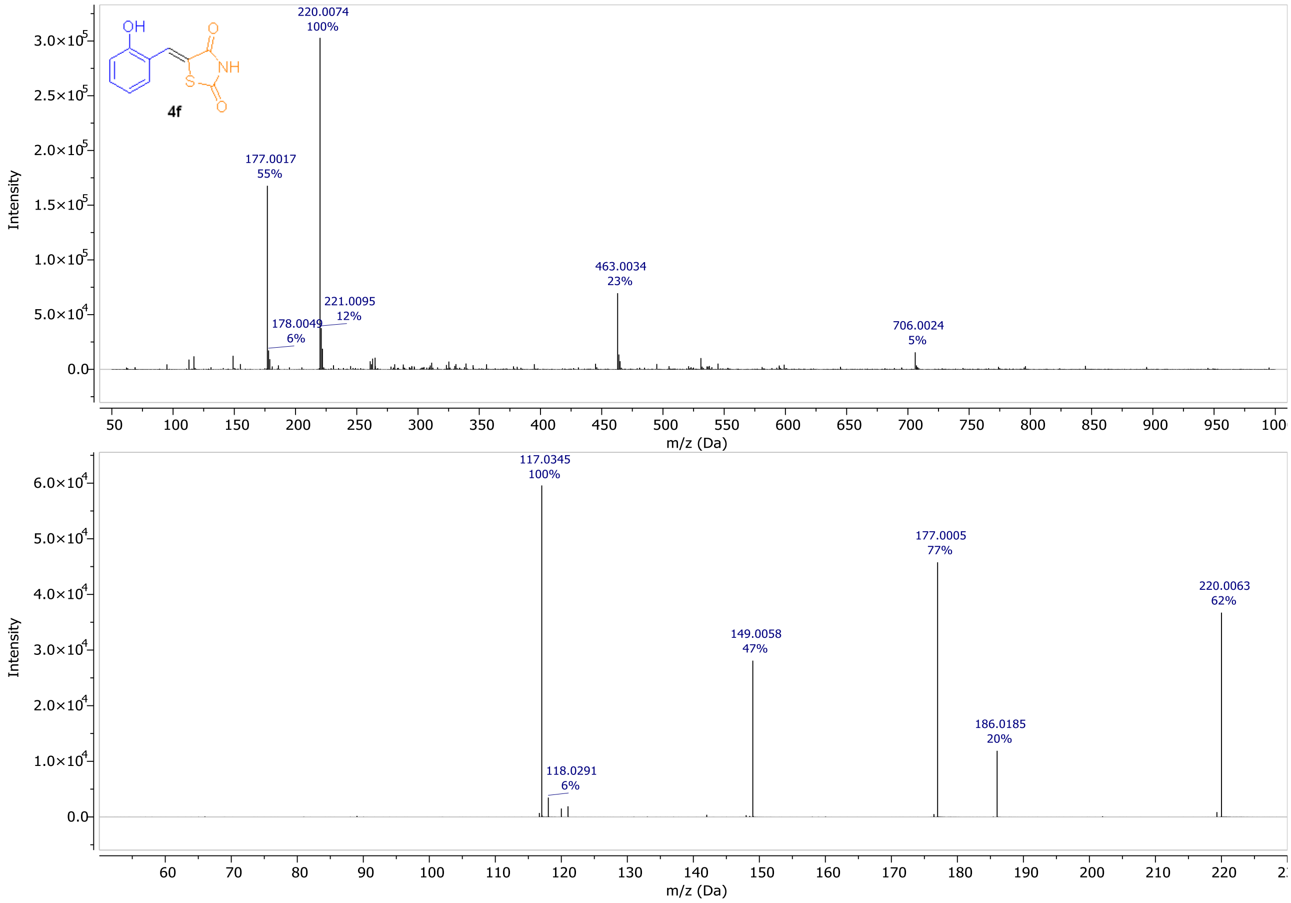

Figure S133. HRMS (ESI-QTOF) of compound **4f** and HRMS/MS for [M−H]⁻.

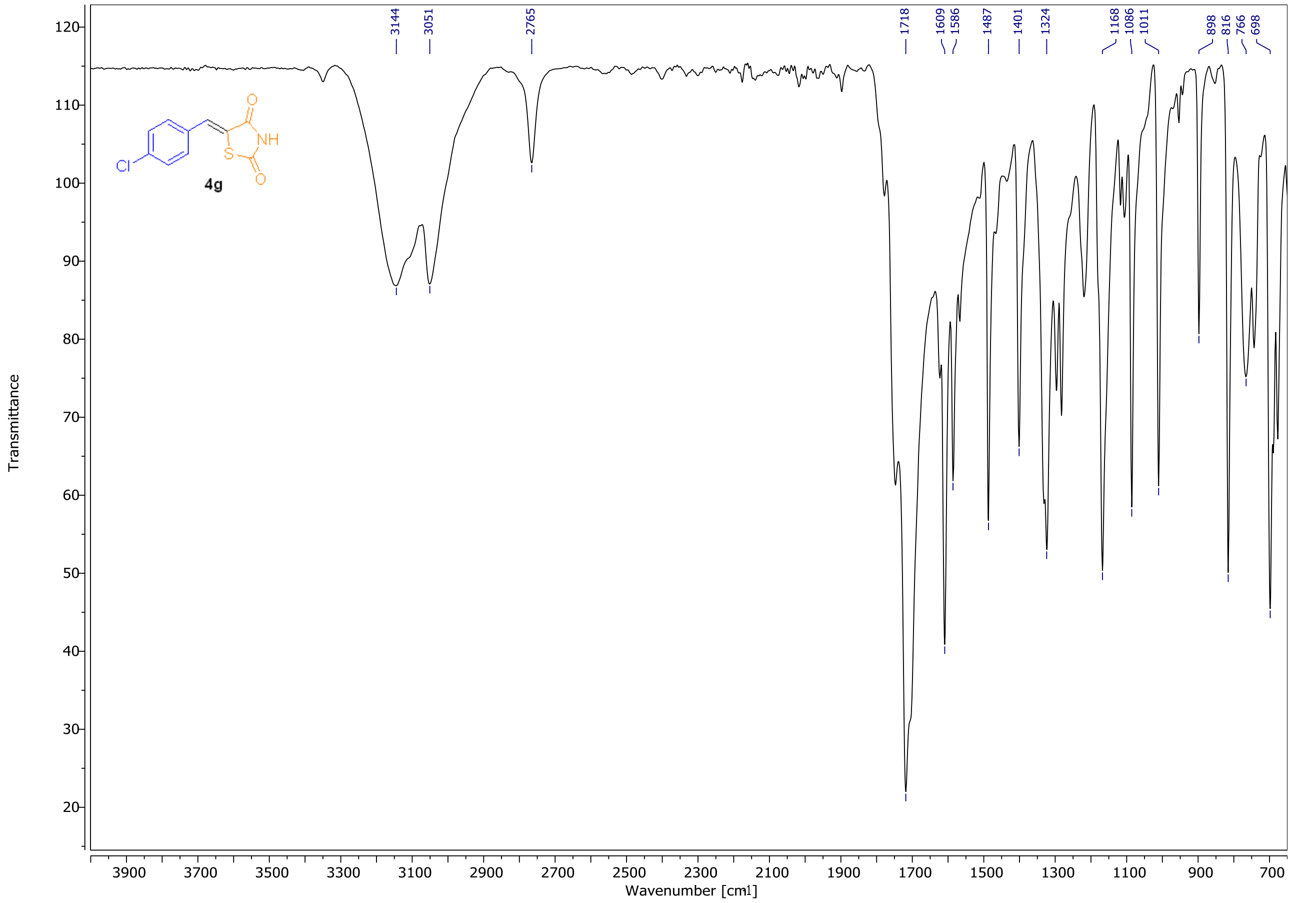

Figure S134. FTIR (ATR) of compound **4g**.

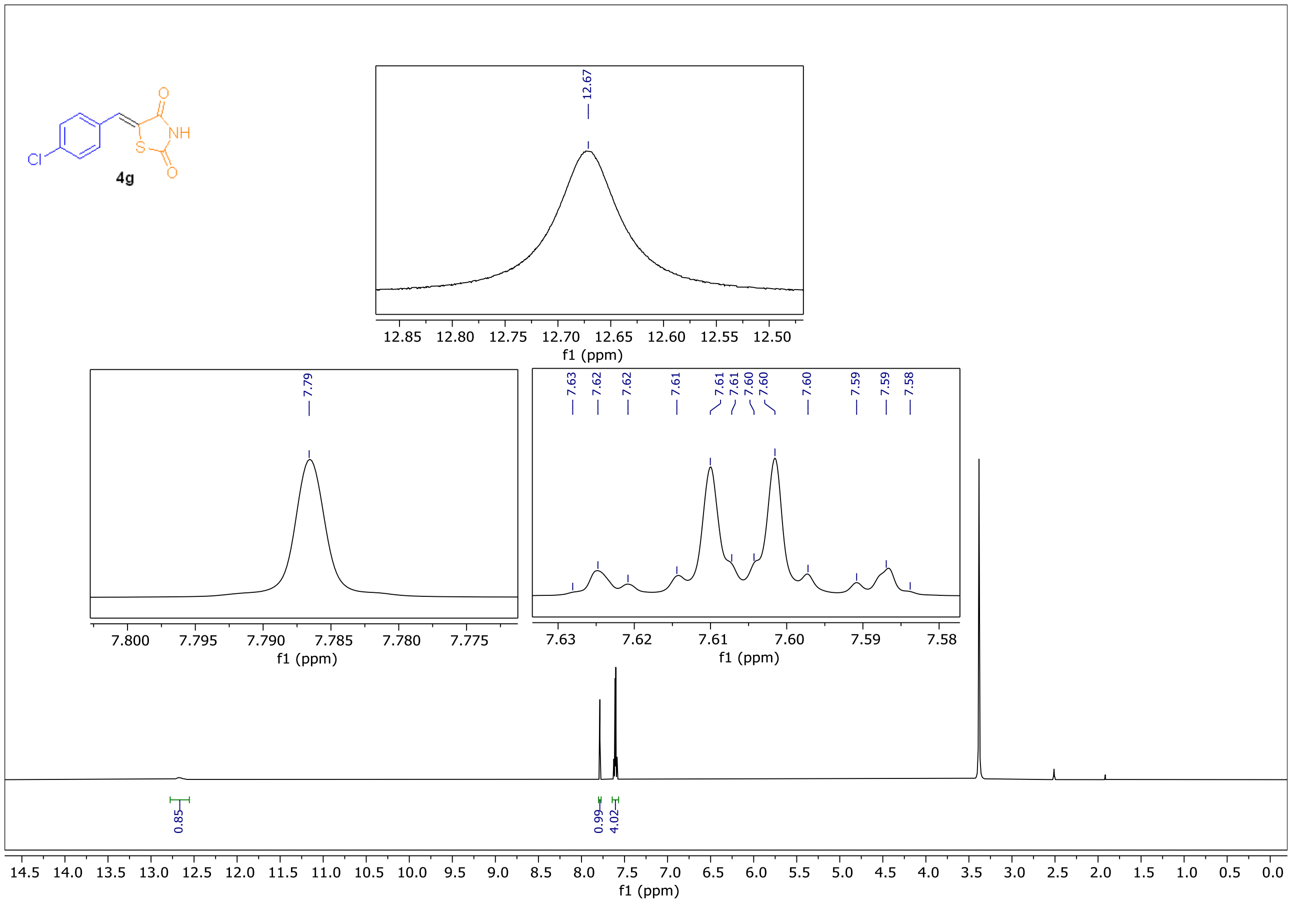

Figure S135. ^{1}H NMR spectrum (600 MHz, DMSO-*d*6) of compound **4g**.

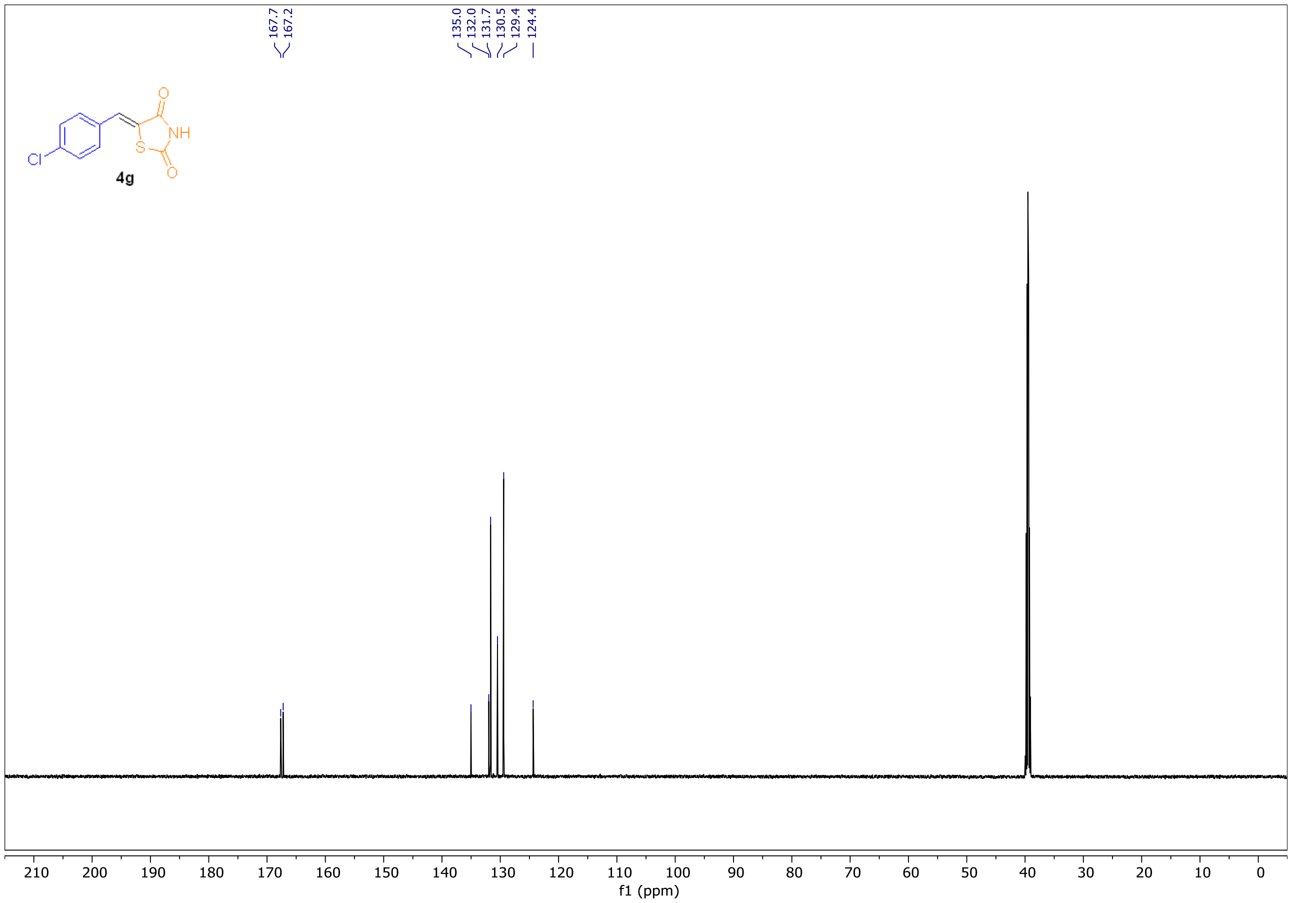

Figure S136. ^{13}C NMR spectrum (151 MHz, DMSO-d_6) of compound **4g**.

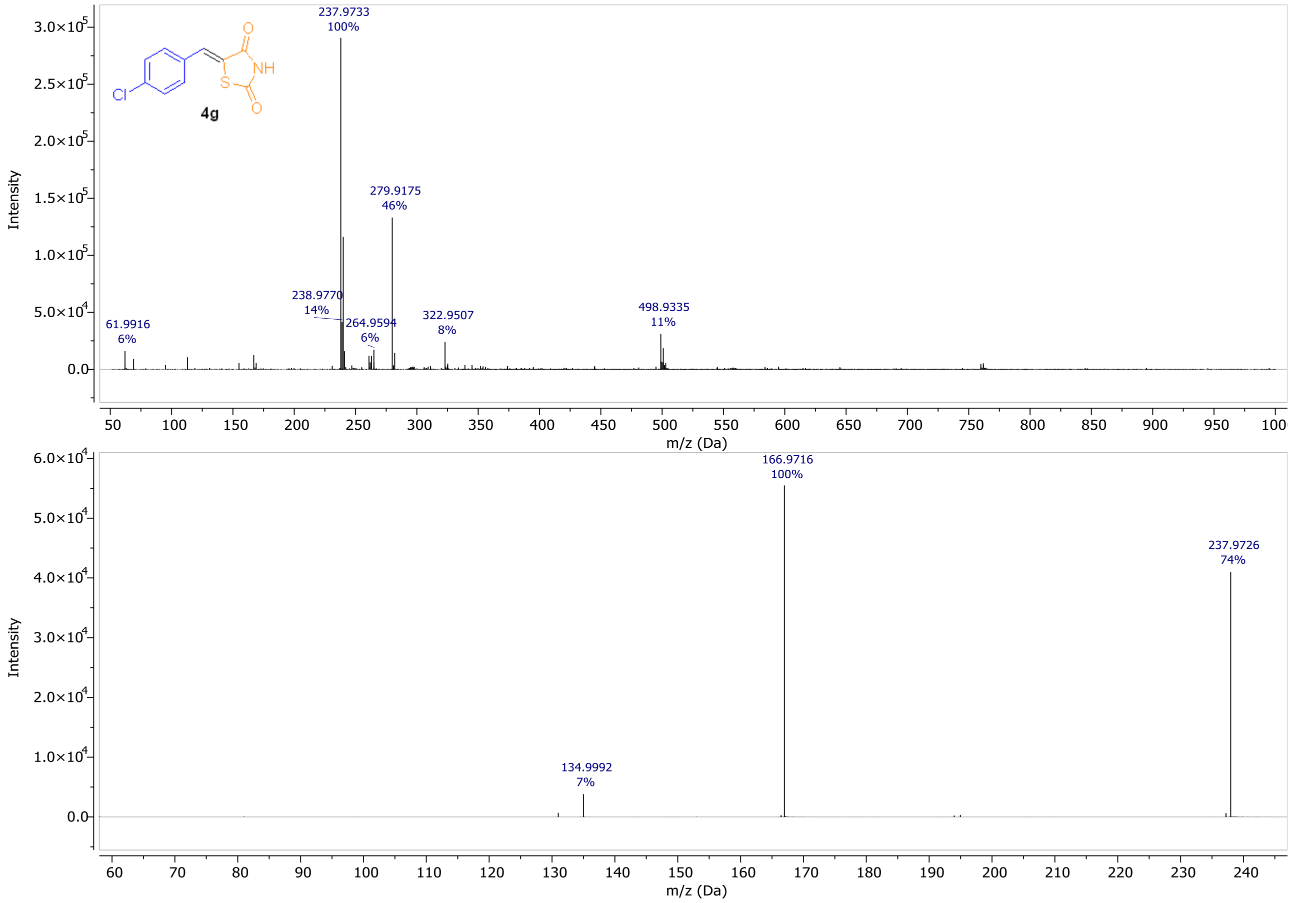

Figure S137. HRMS (ESI-QTOF) of compound **4g** and HRMS/MS for [M−H]⁻.

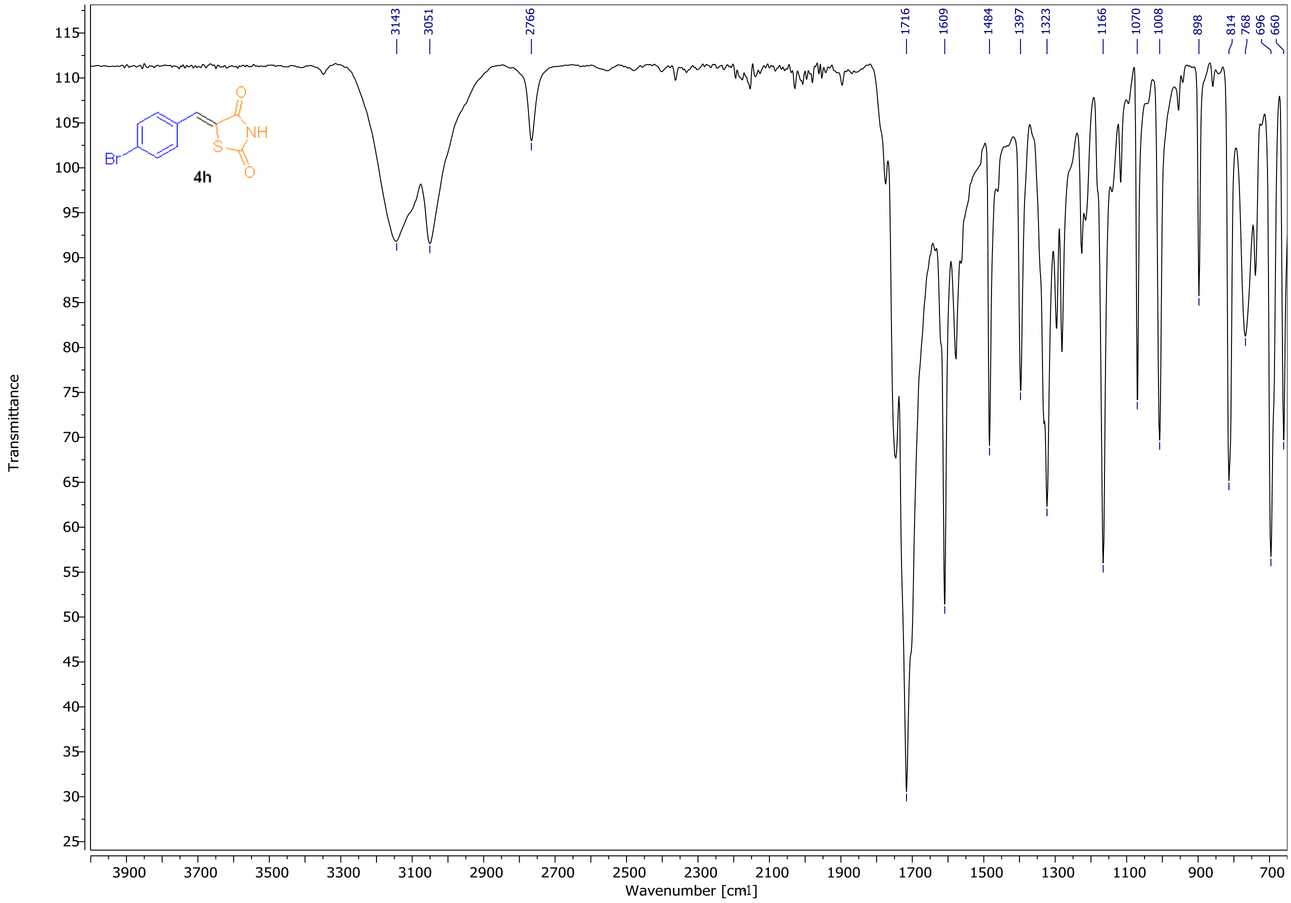

Figure S138. FTIR (ATR) of compound **4h**.

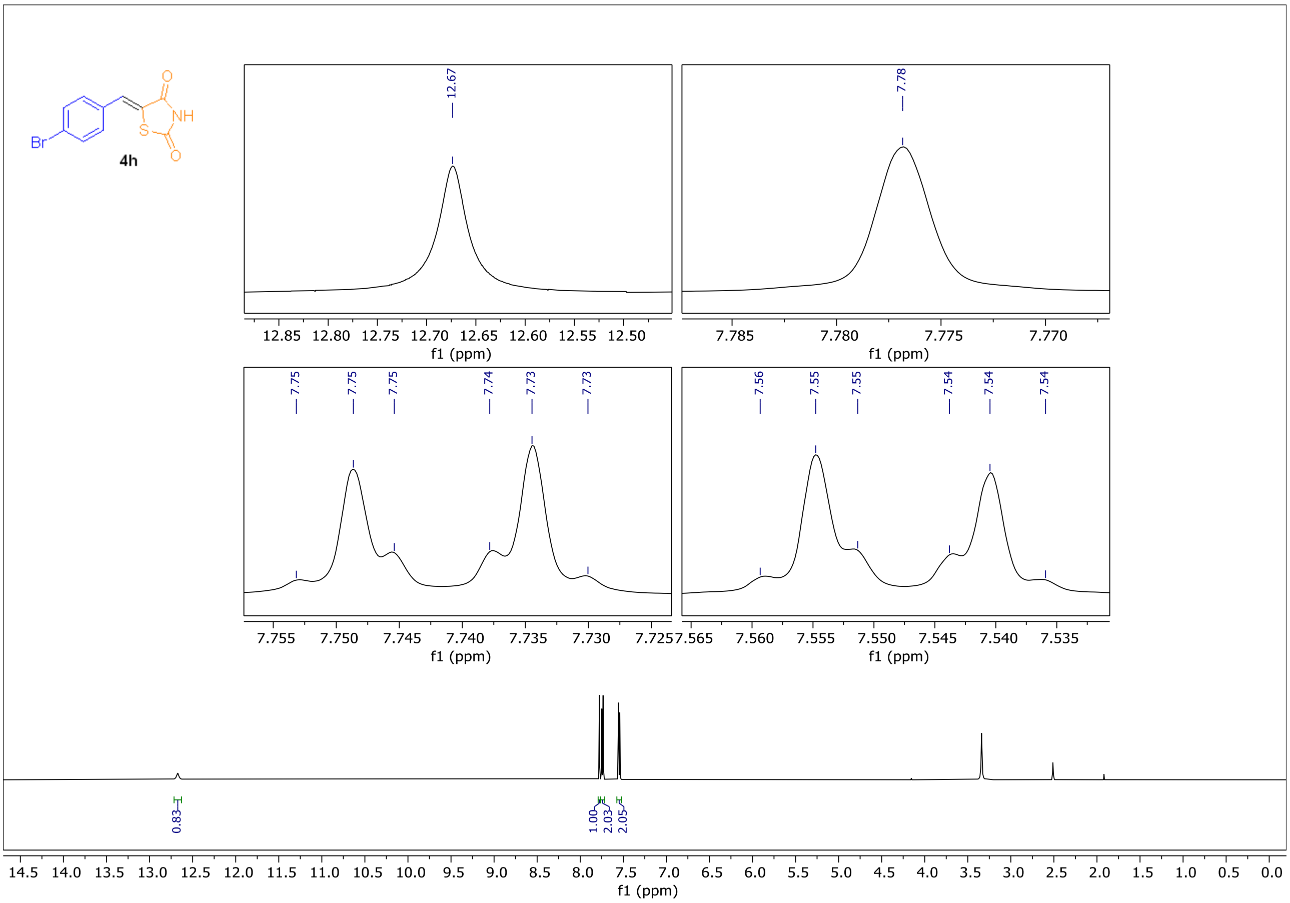

Figure S139. ^{1}H NMR spectrum (600 MHz, DMSO-d_6) of compound **4h**.

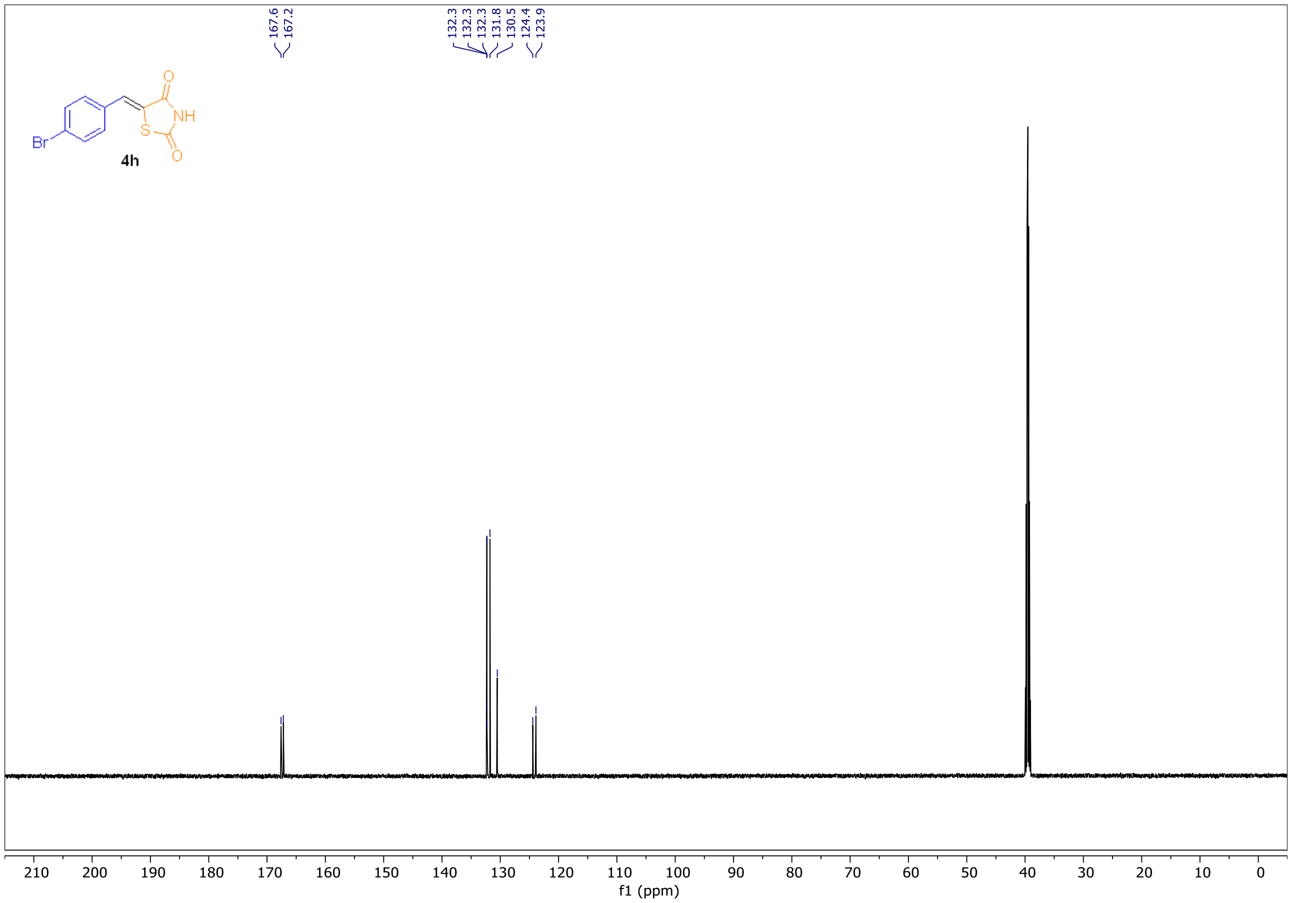

Figure S140. ^{13}C NMR spectrum (151 MHz, DMSO-d_6) of compound **4h**.

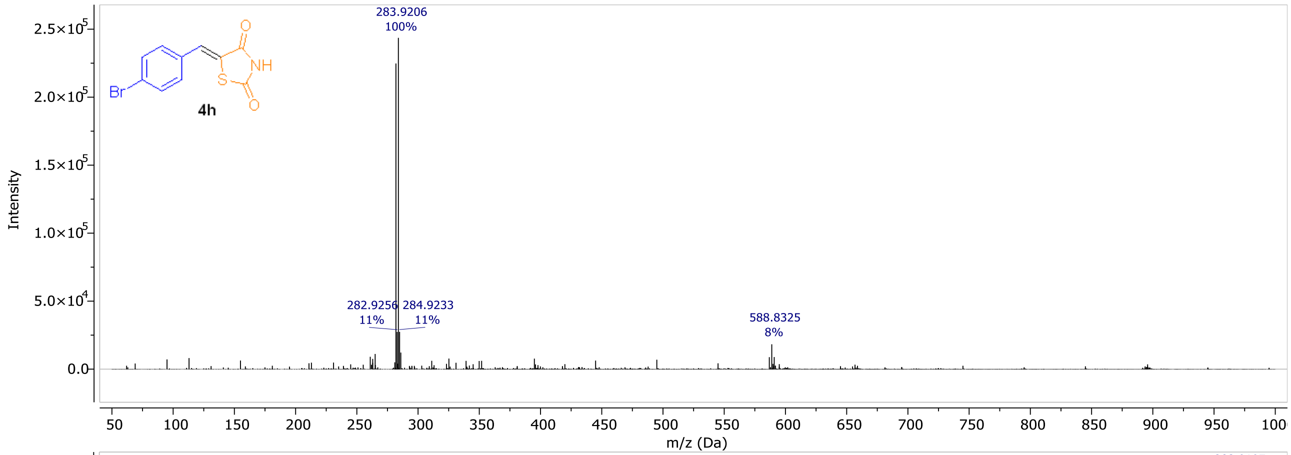

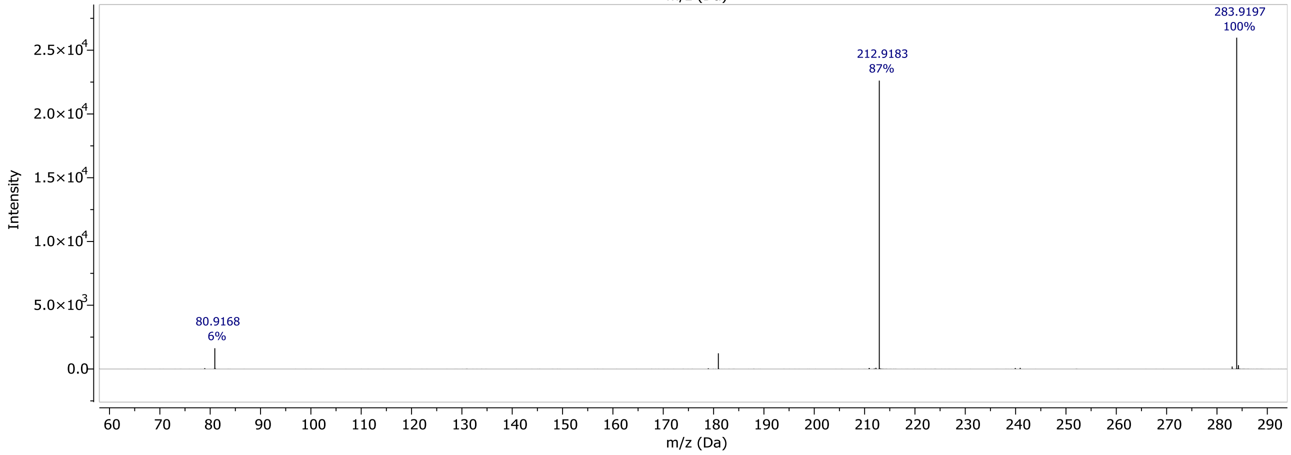

Figure S141. HRMS (ESI-QTOF) of compound **4h** and HRMS/MS for $[M-H]^-$.

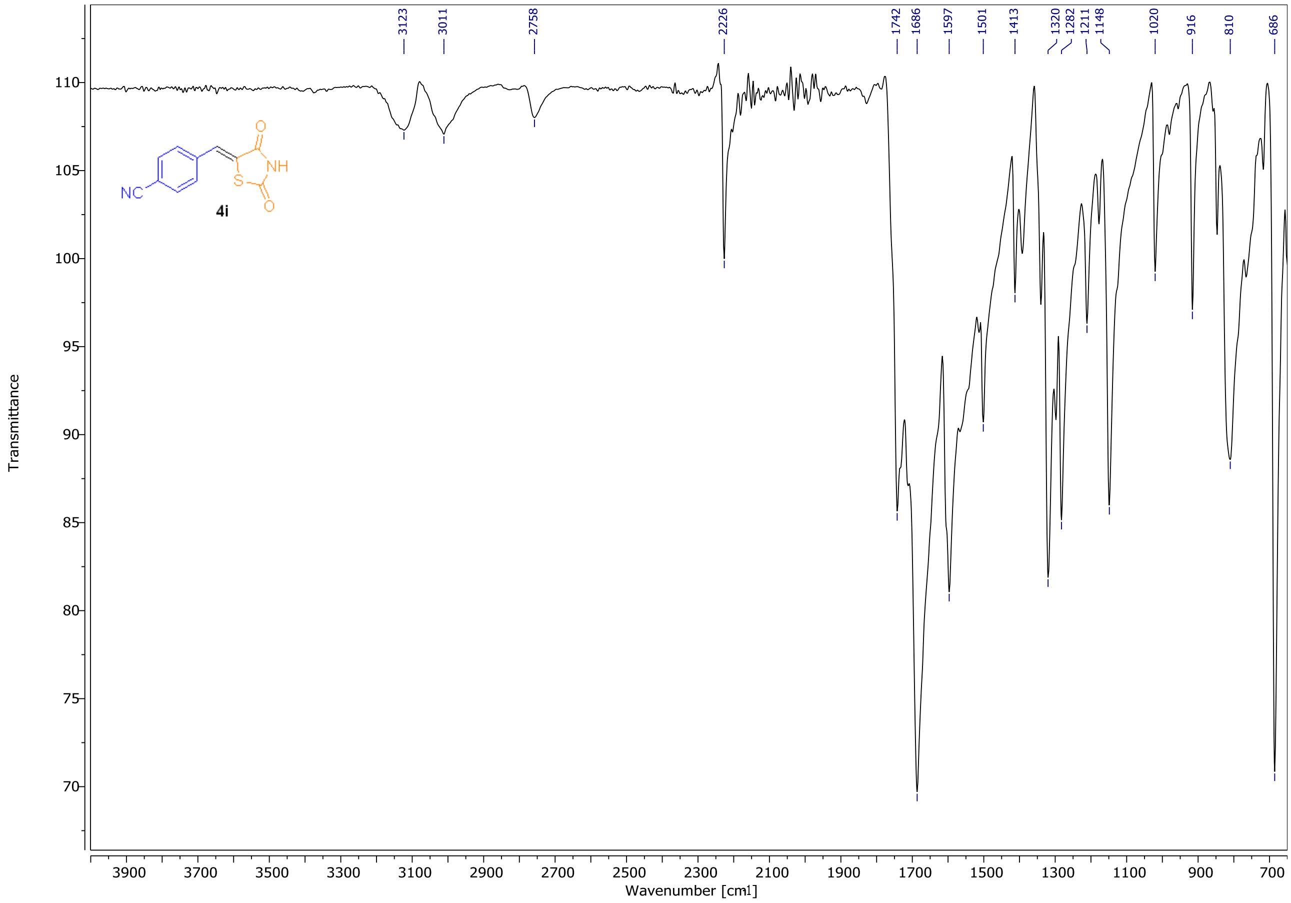

Figure S142. FTIR (ATR) of compound **4i**.

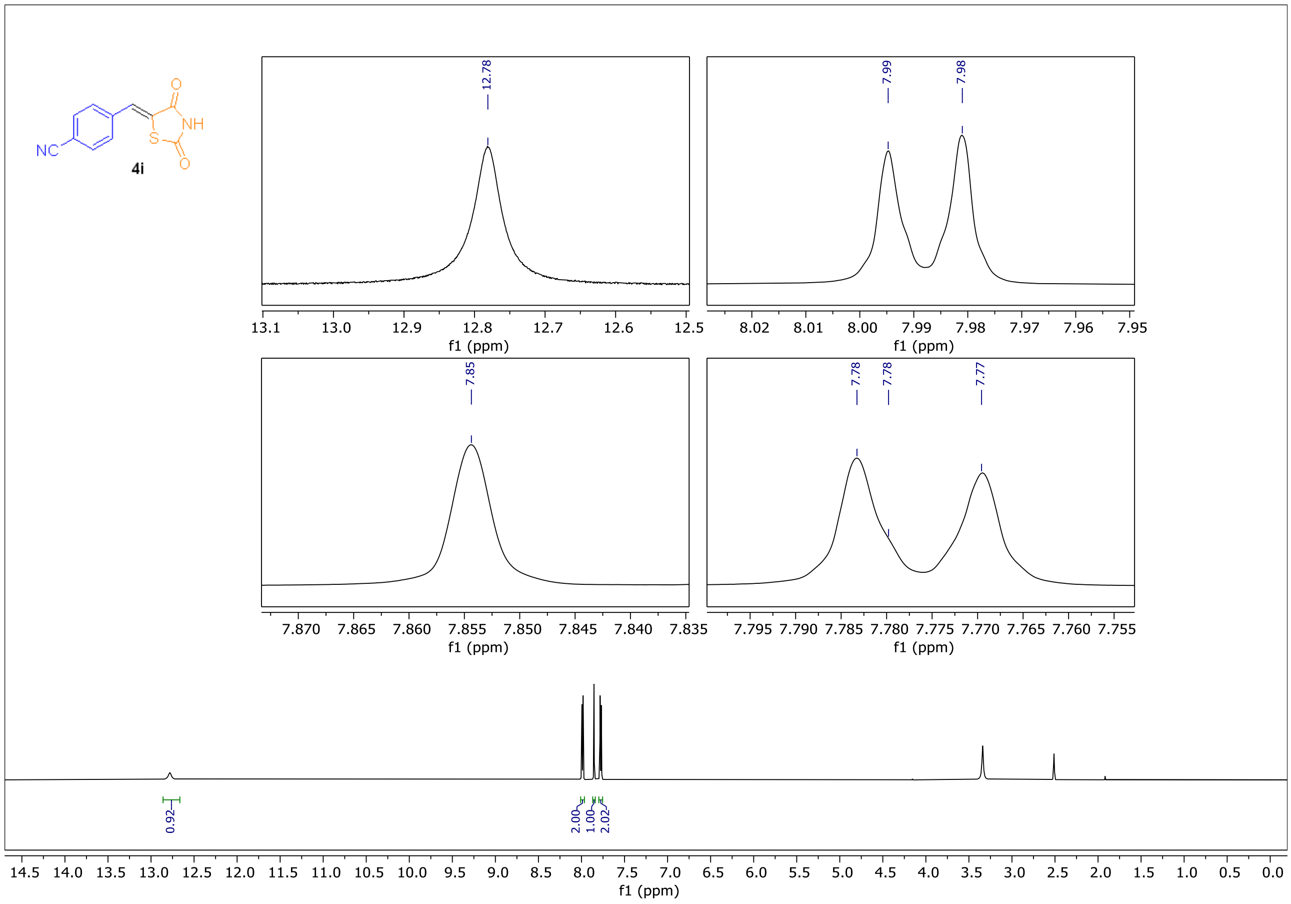

Figure S143. ^{1}H NMR spectrum (600 MHz, DMSO-d_6) of compound **4i**.

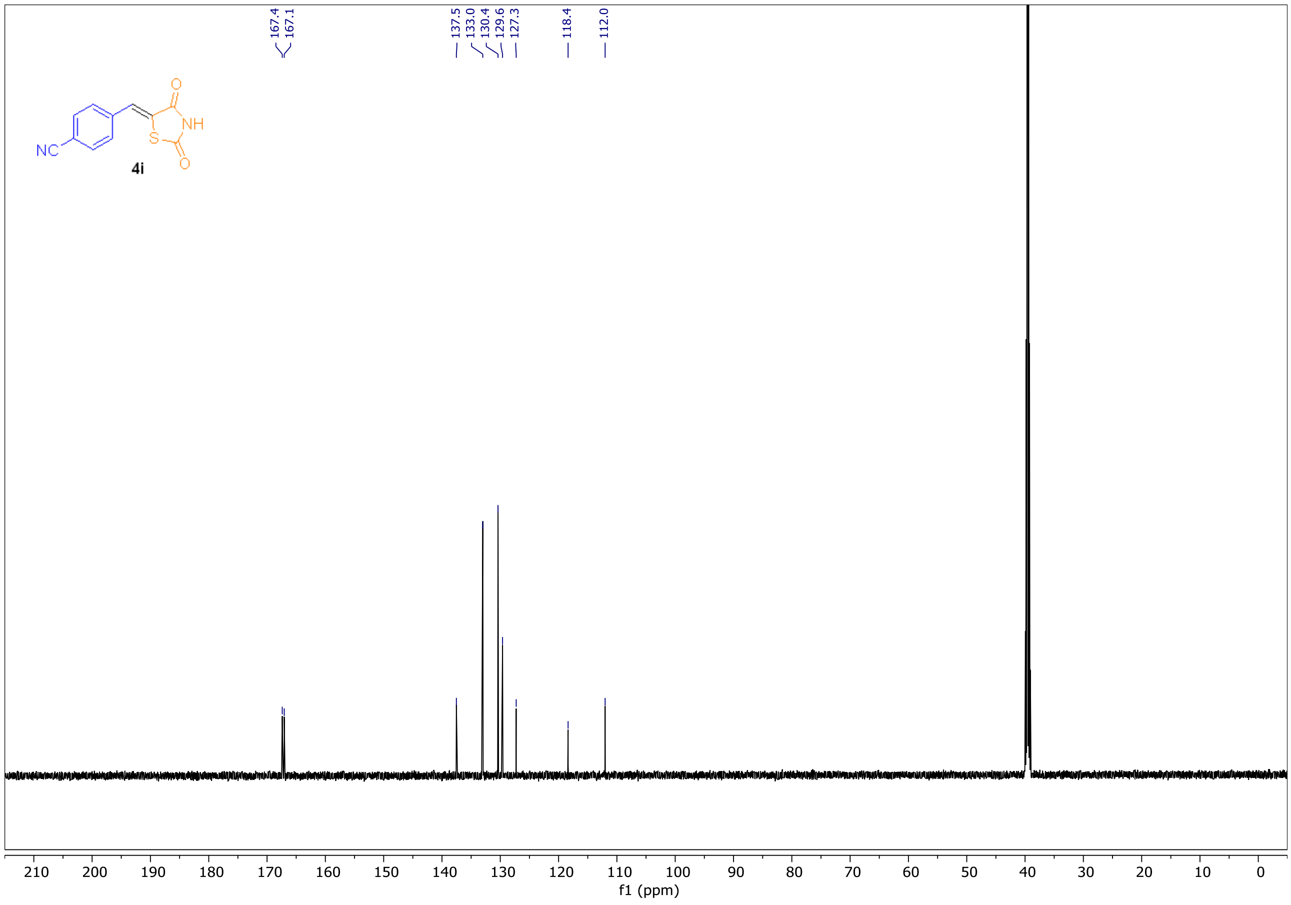

Figure S144. ^{13}C NMR spectrum (151 MHz, DMSO-d_6) of compound **4i**.

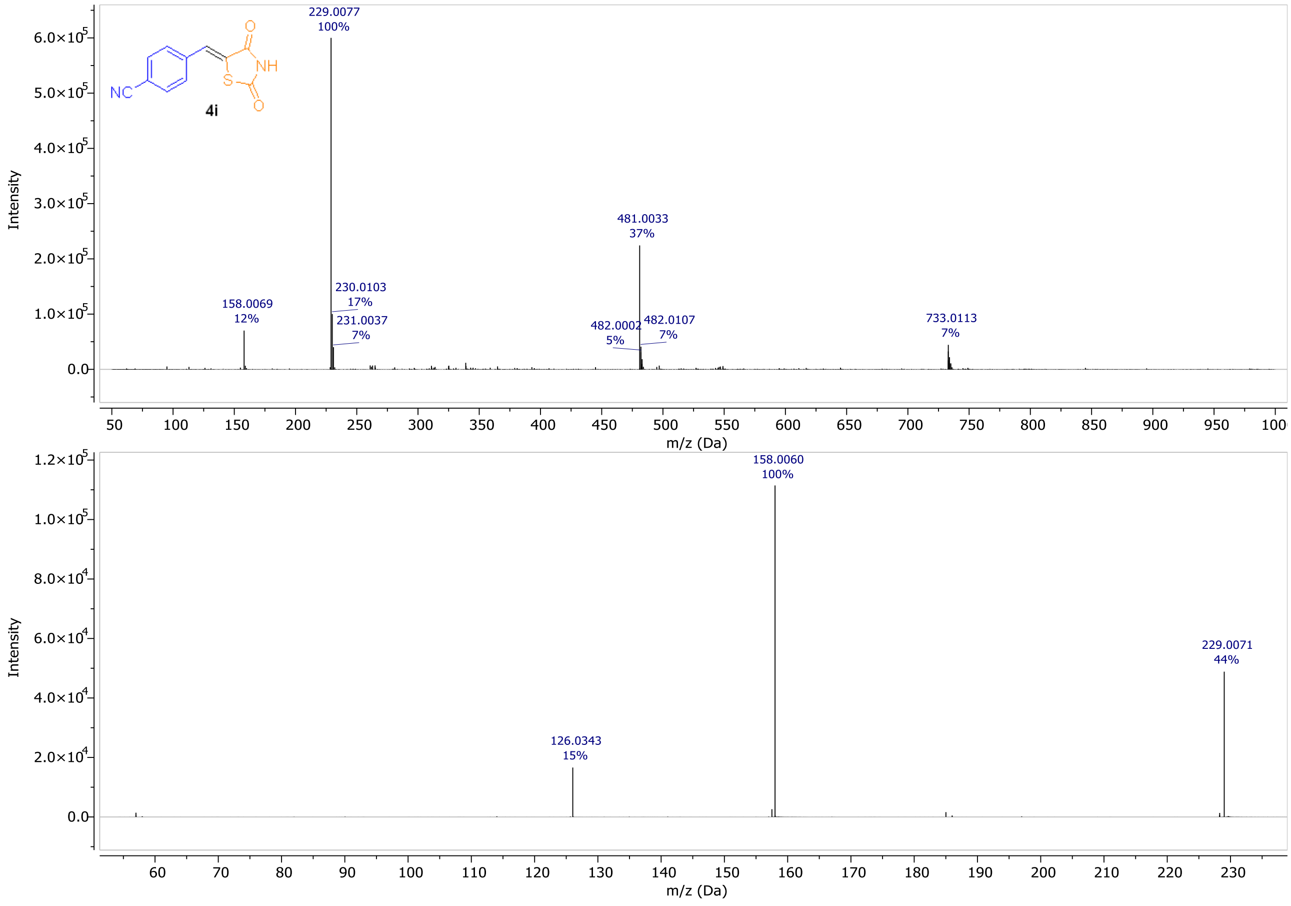

Figure S145. HRMS (ESI-QTOF) of compound **4i** and HRMS/MS for [M−H]⁻.

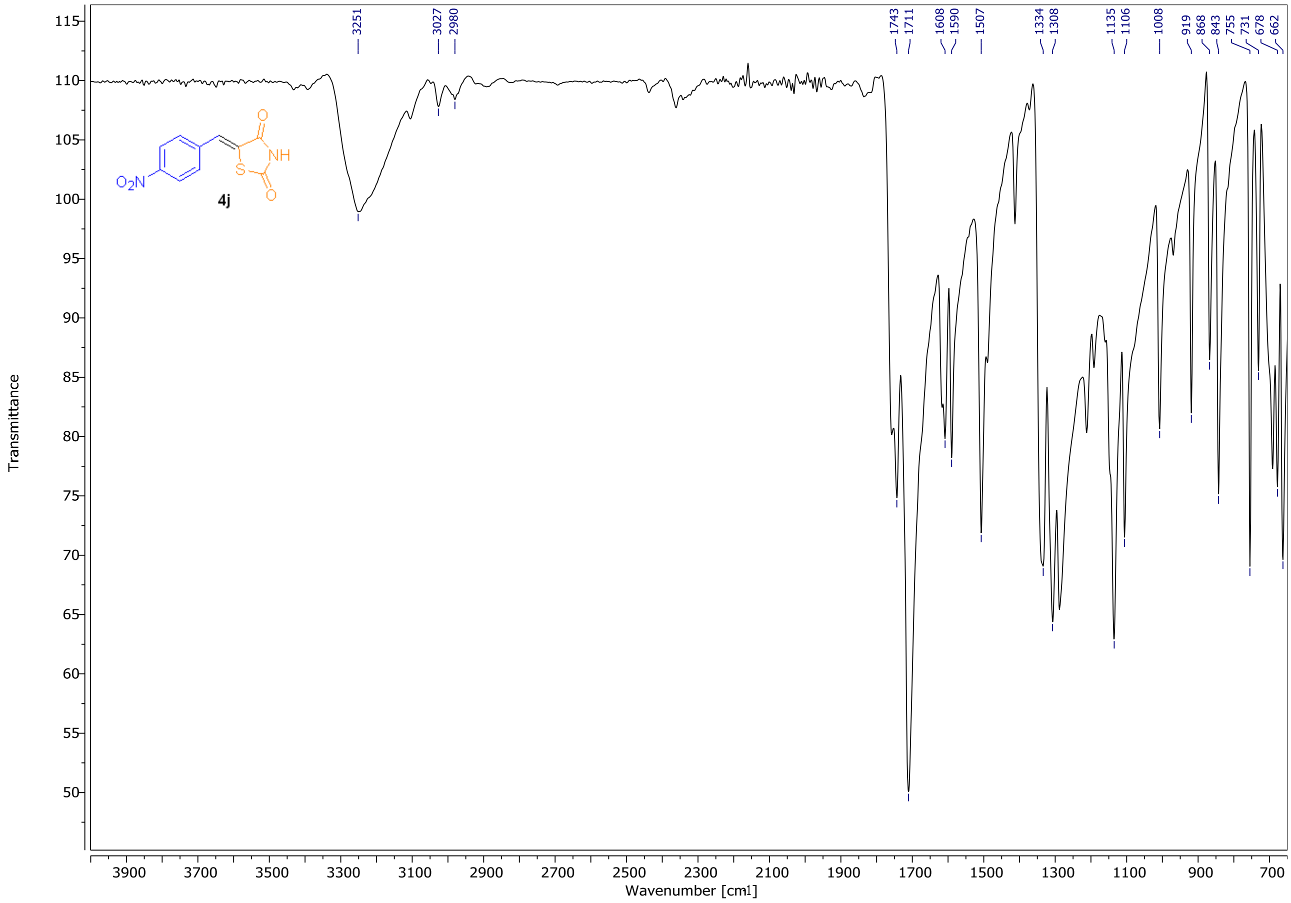

Figure S146. FTIR (ATR) of compound **4j**.

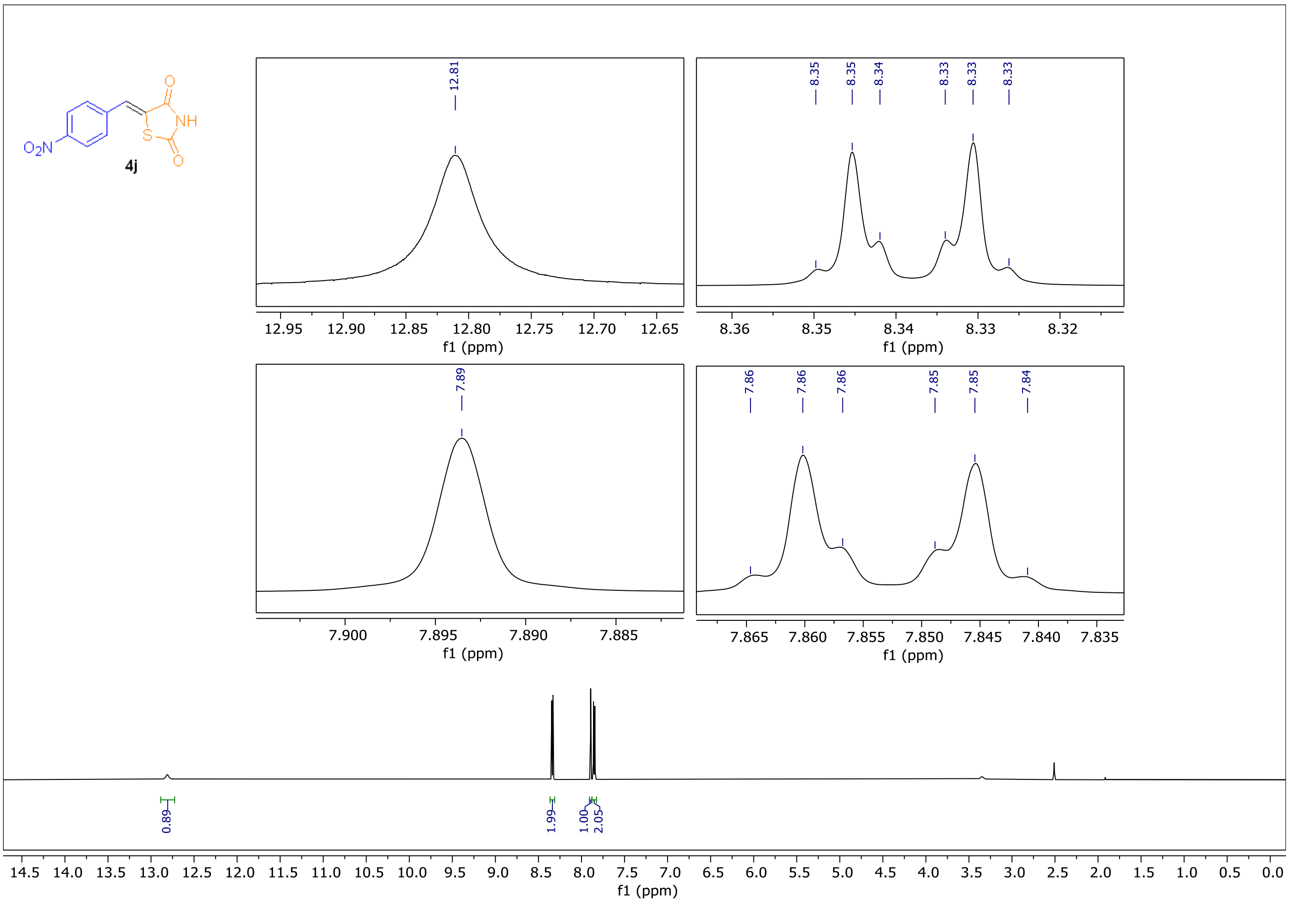

Figure S147. ^{1}H NMR spectrum (600 MHz, DMSO-d_6) of compound **4j**.

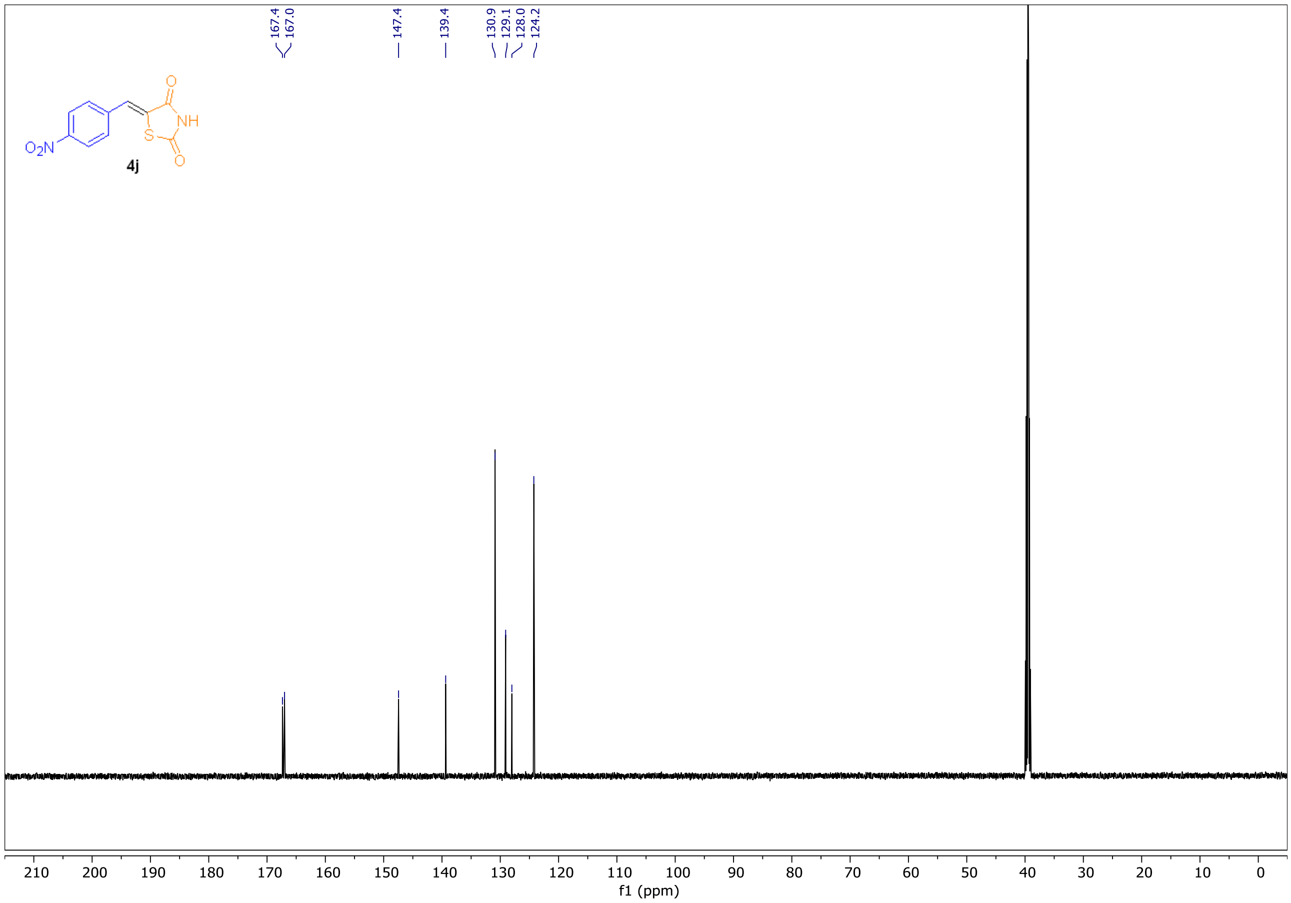

Figure S148. ^{13}C NMR spectrum (151 MHz, DMSO-d_6) of compound **4j**.

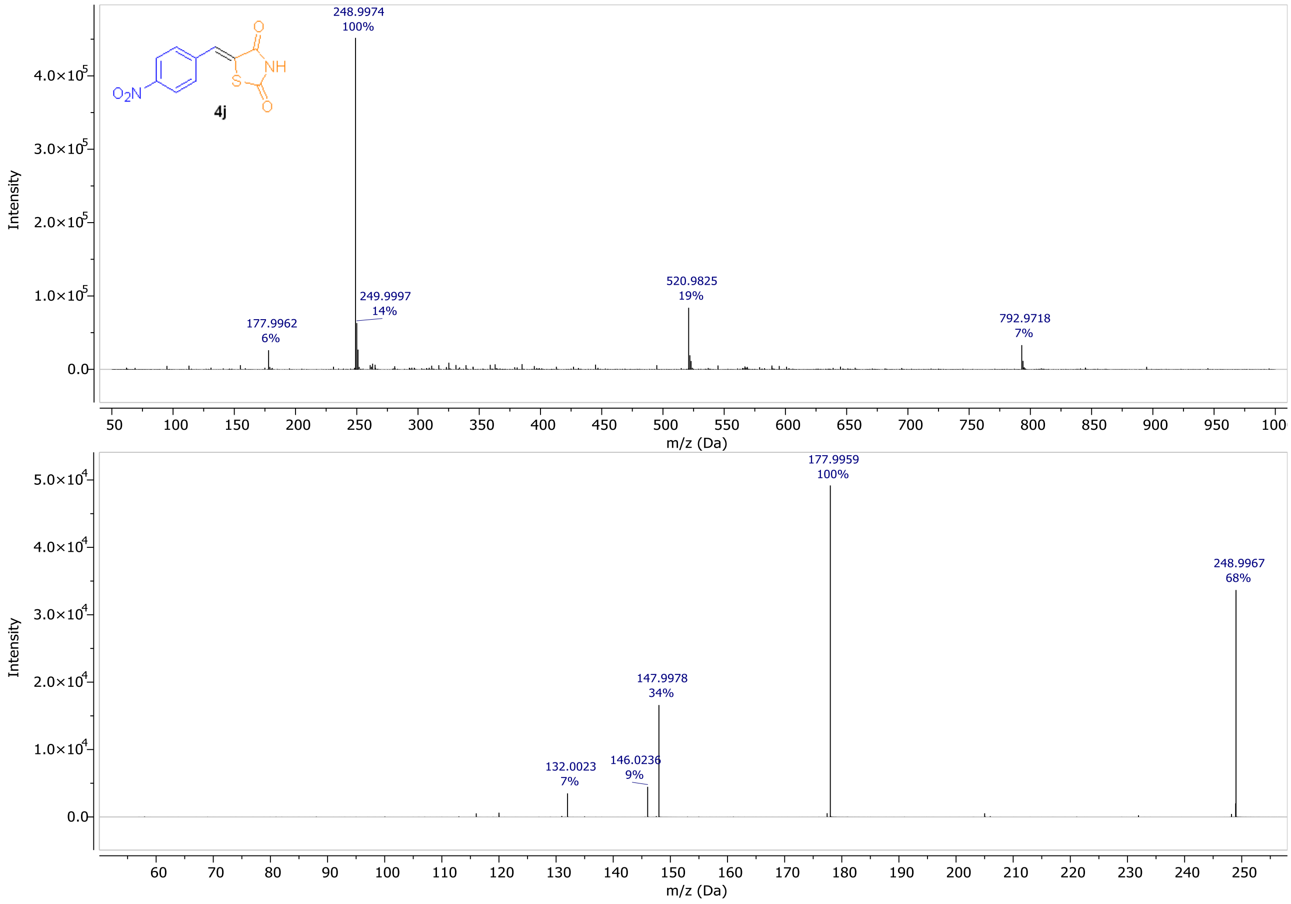

Figure S149. HRMS (ESI-QTOF) of compound **4j** and HRMS/MS for [M−H]⁻.

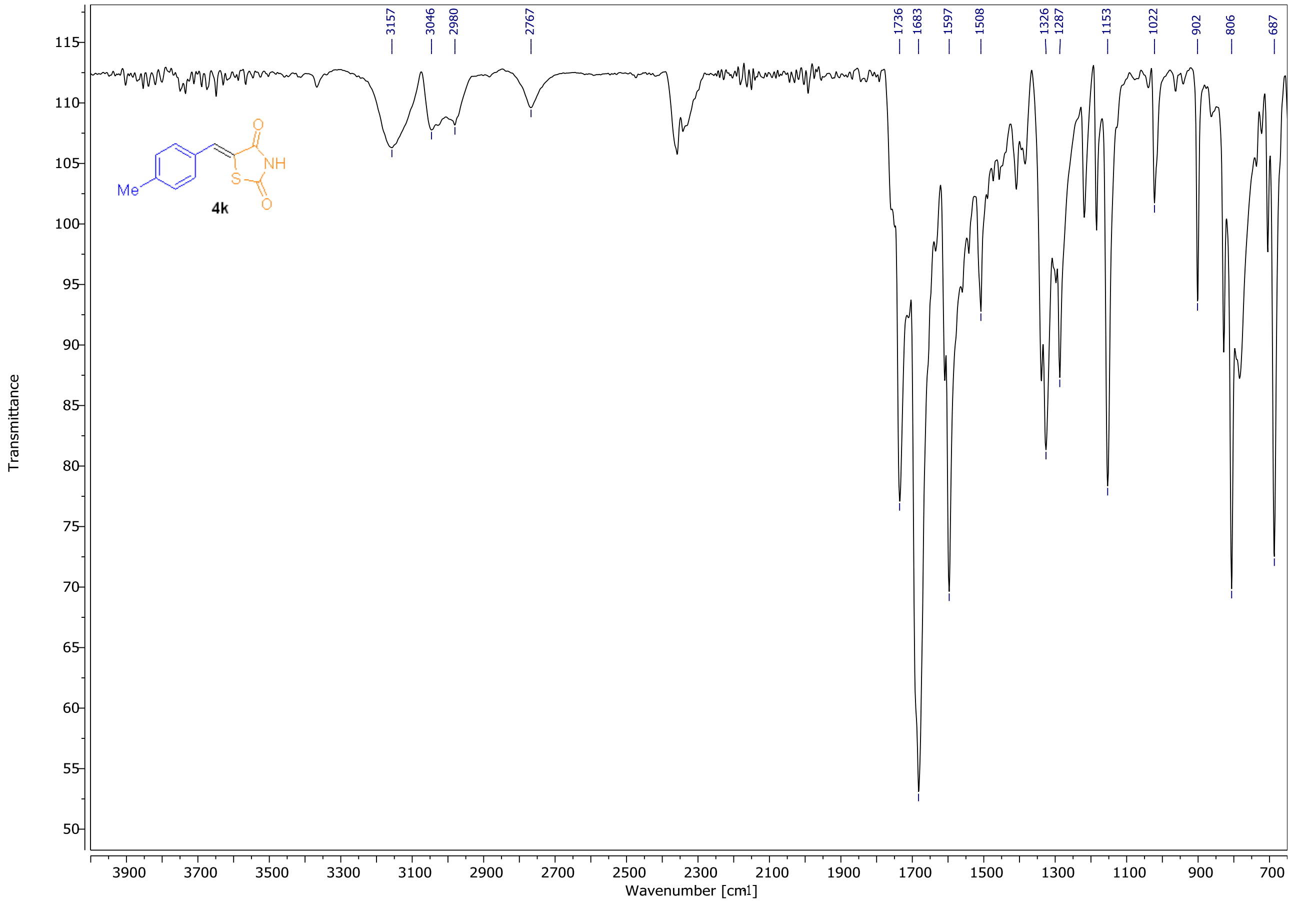

Figure S150. FTIR (ATR) of compound **4k**.

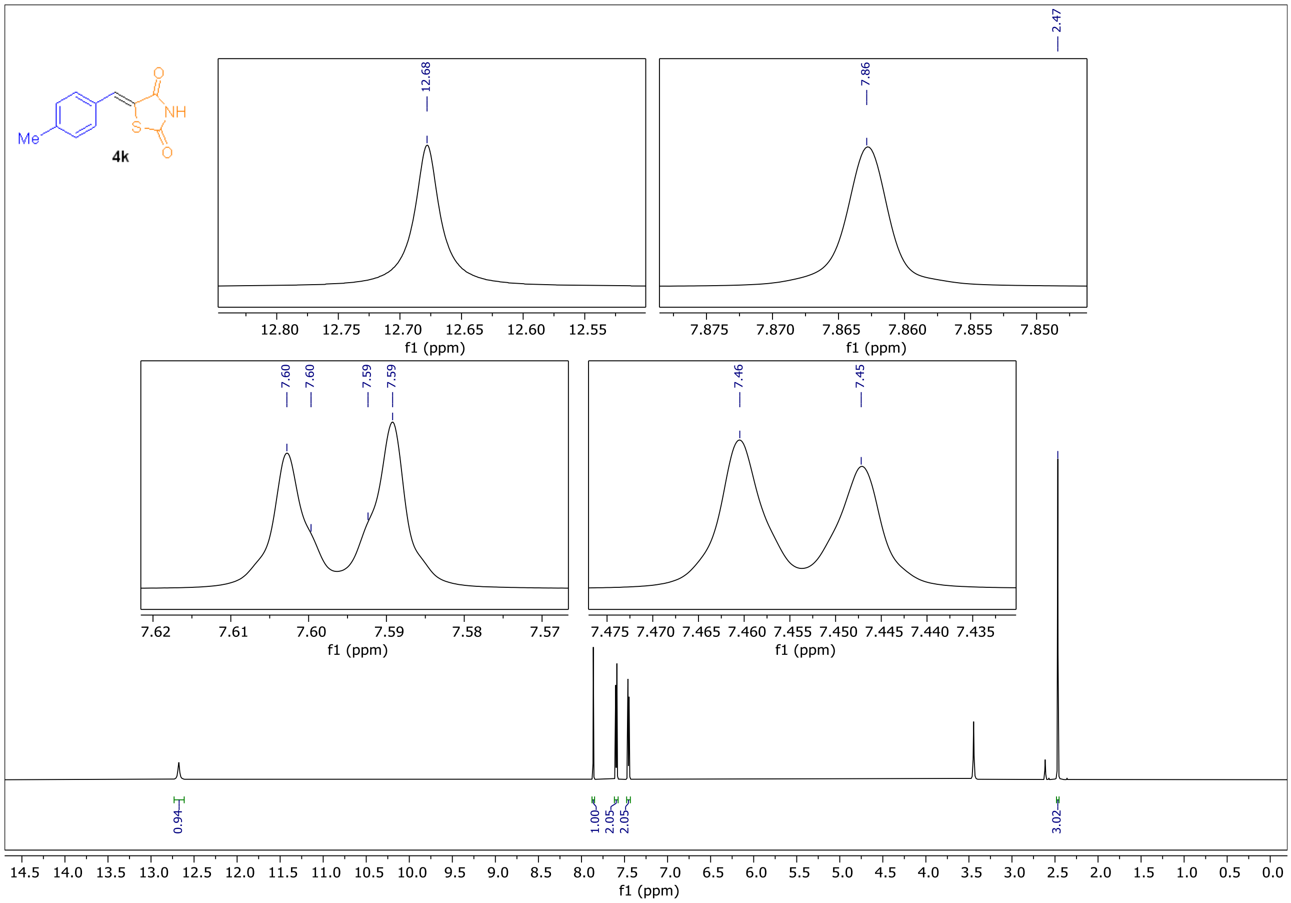

Figure S151. ^{1}H NMR spectrum (600 MHz, DMSO-d_6) of compound **4k**.

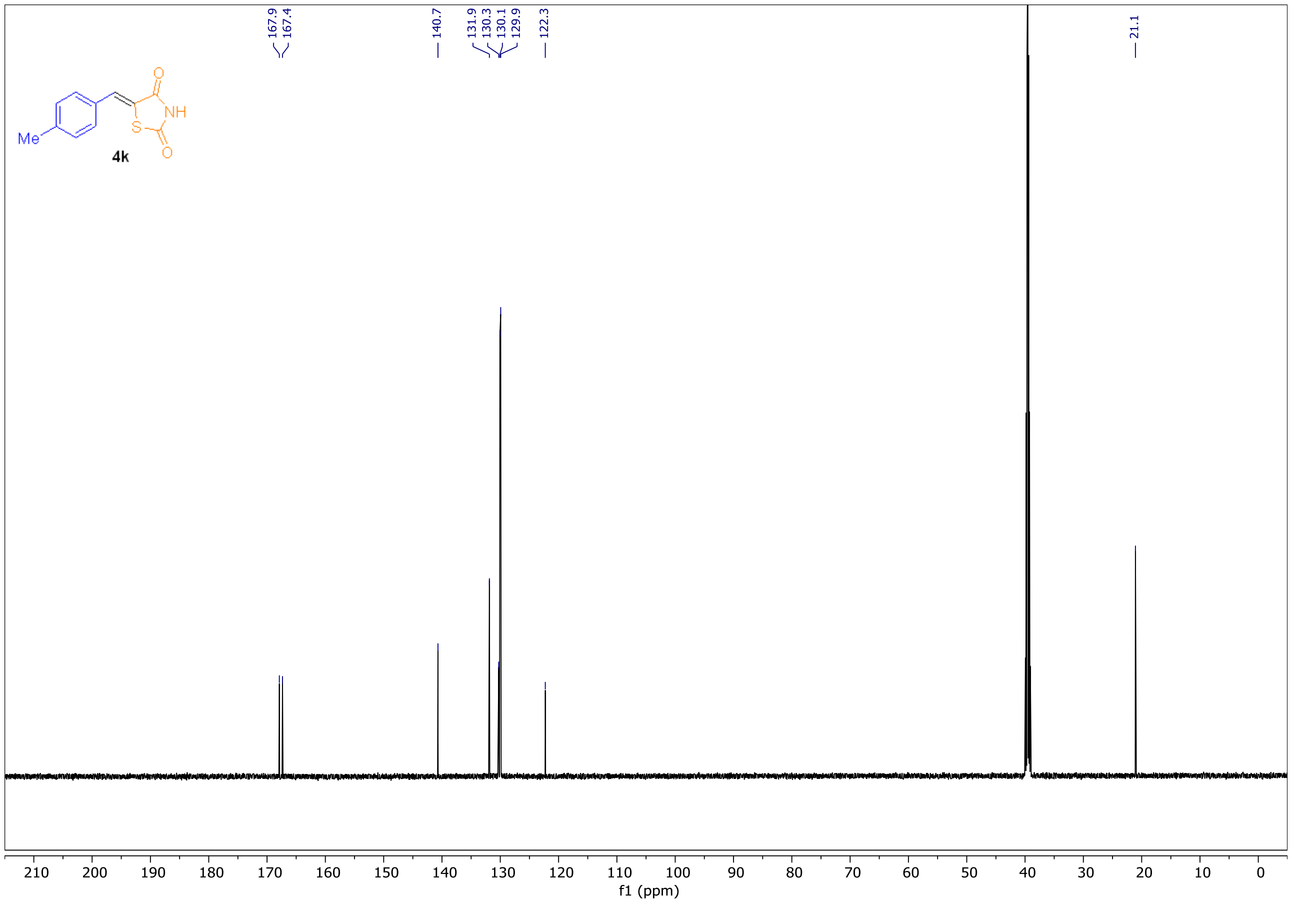

Figure S152. ^{13}C NMR spectrum (151 MHz, DMSO-d_6) of compound **4k**.

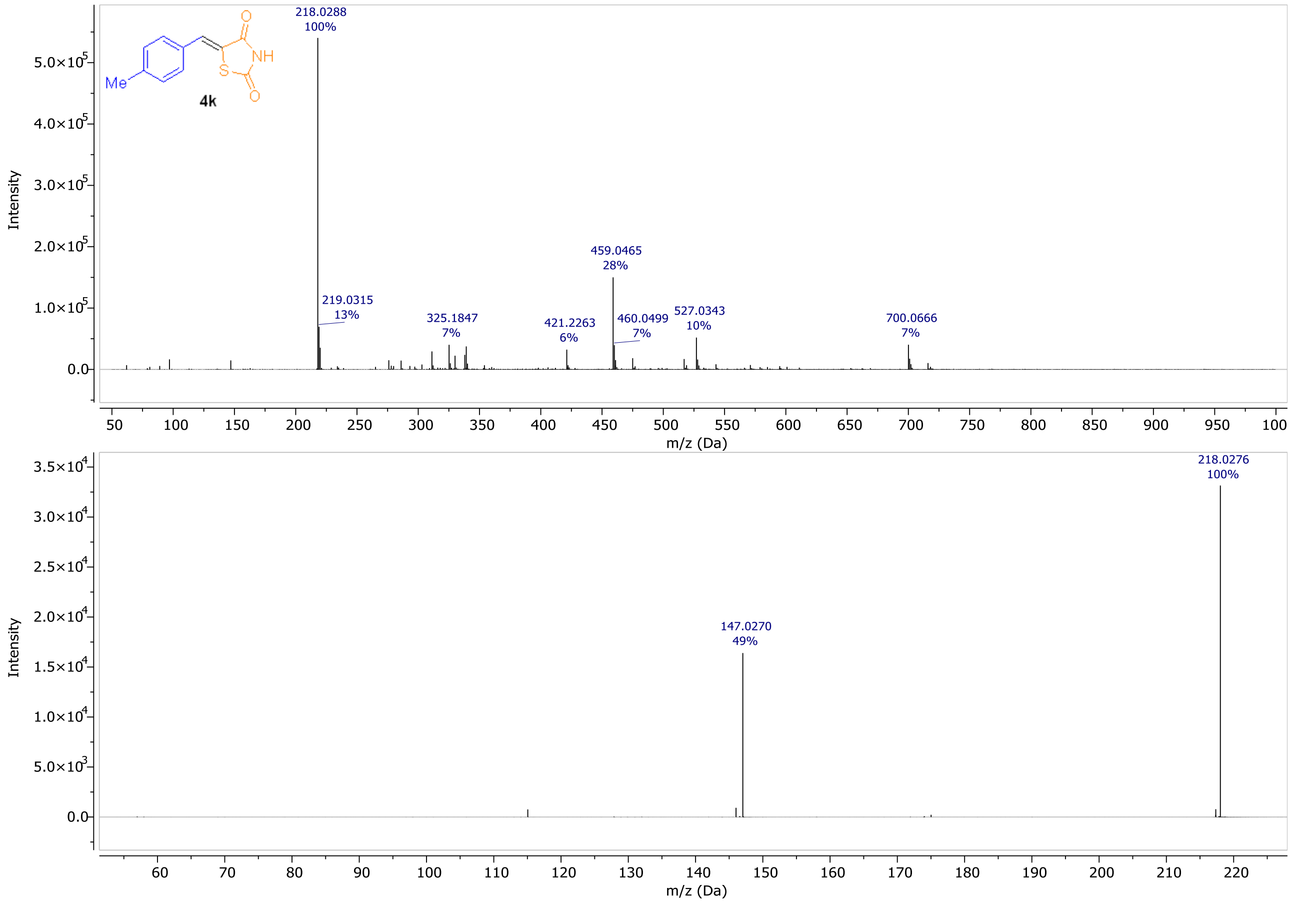

Figure S153. HRMS (ESI-QTOF) of compound **4k** and HRMS/MS for [M−H]⁻.

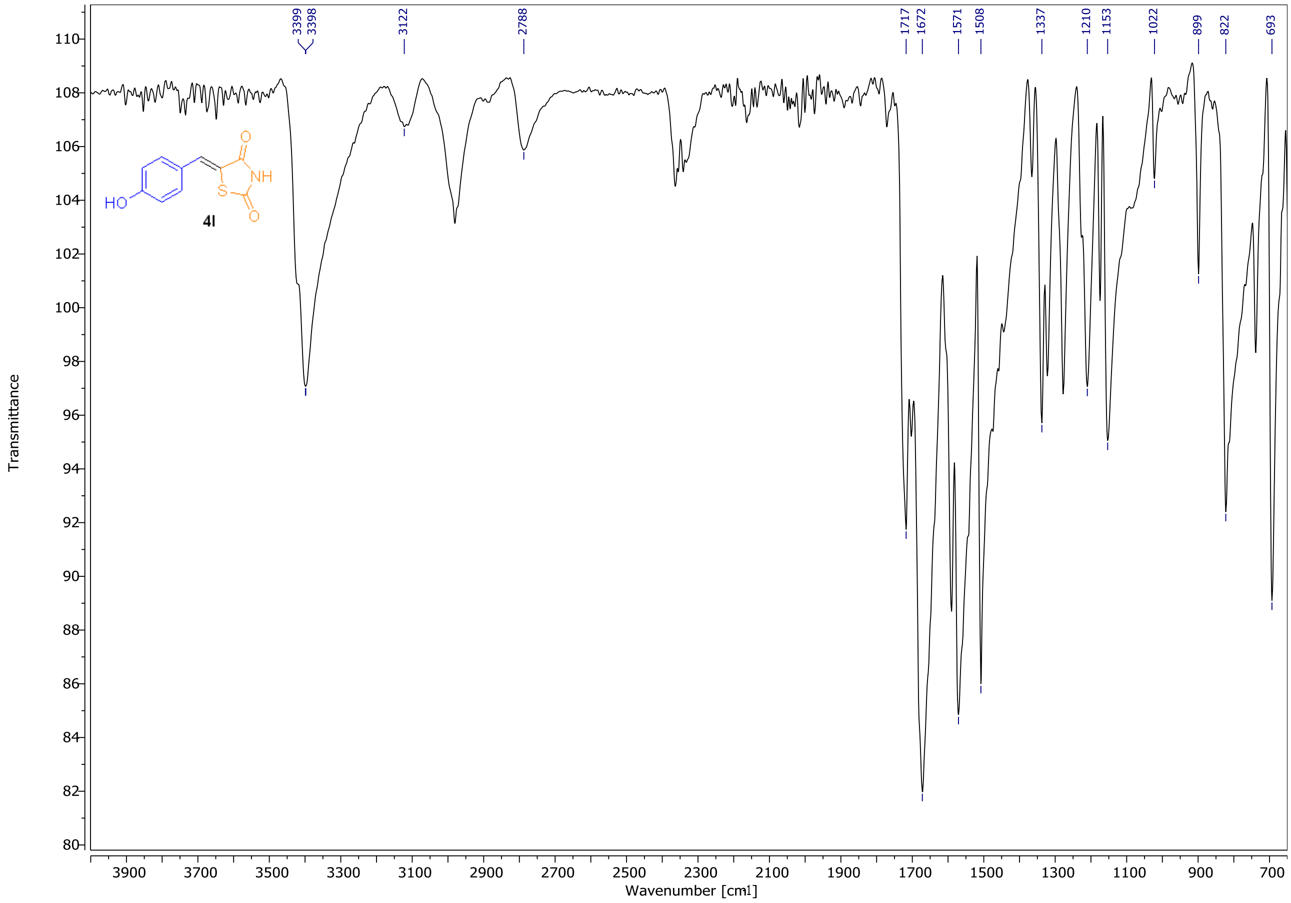

Figure S154. FTIR (ATR) of compound **4l**.

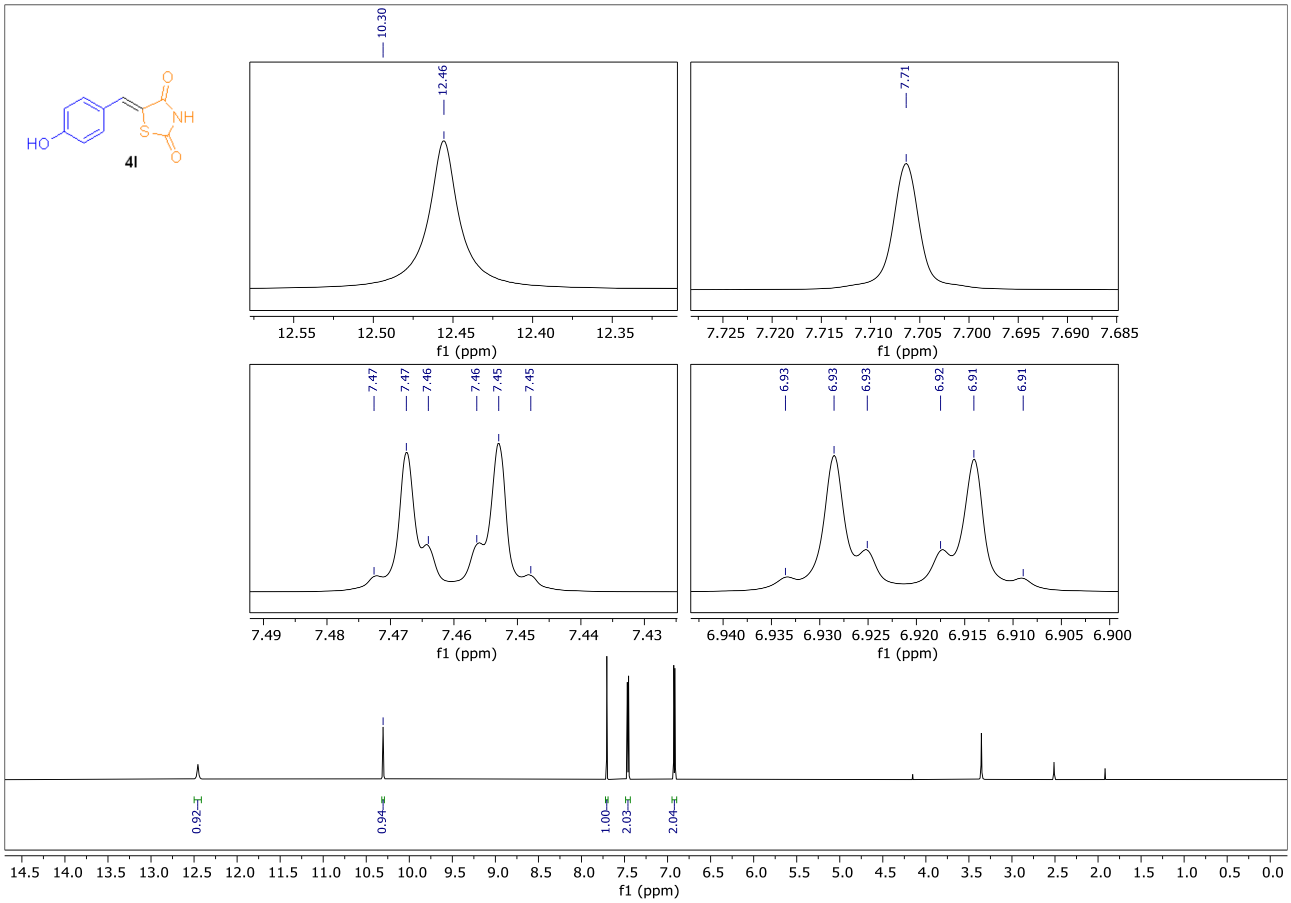

Figure S155. ^{1}H NMR spectrum (600 MHz, DMSO-d_6) of compound **4l**.

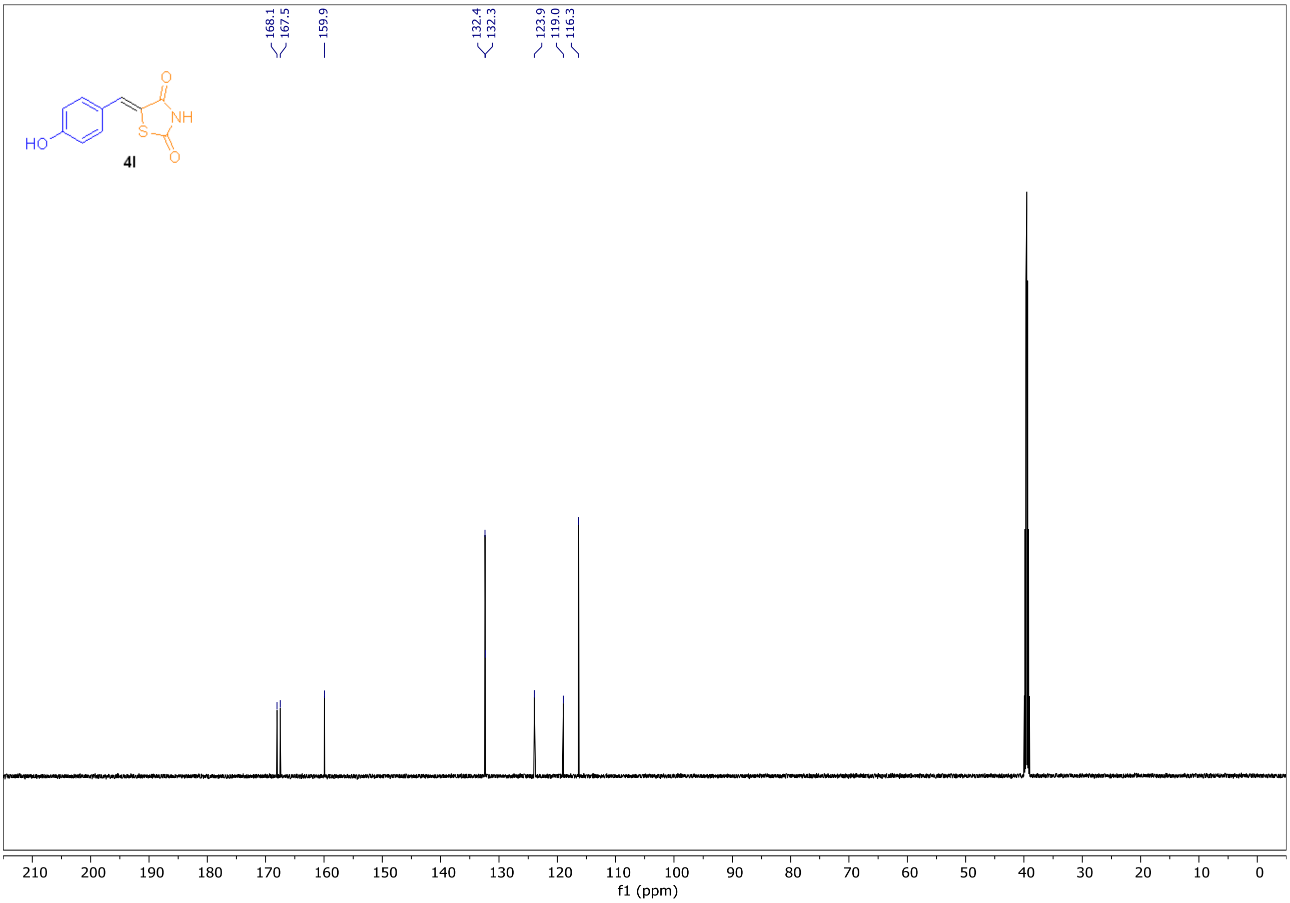

Figure S156. ^{13}C NMR spectrum (151 MHz, DMSO-d_6) of compound **4l**.

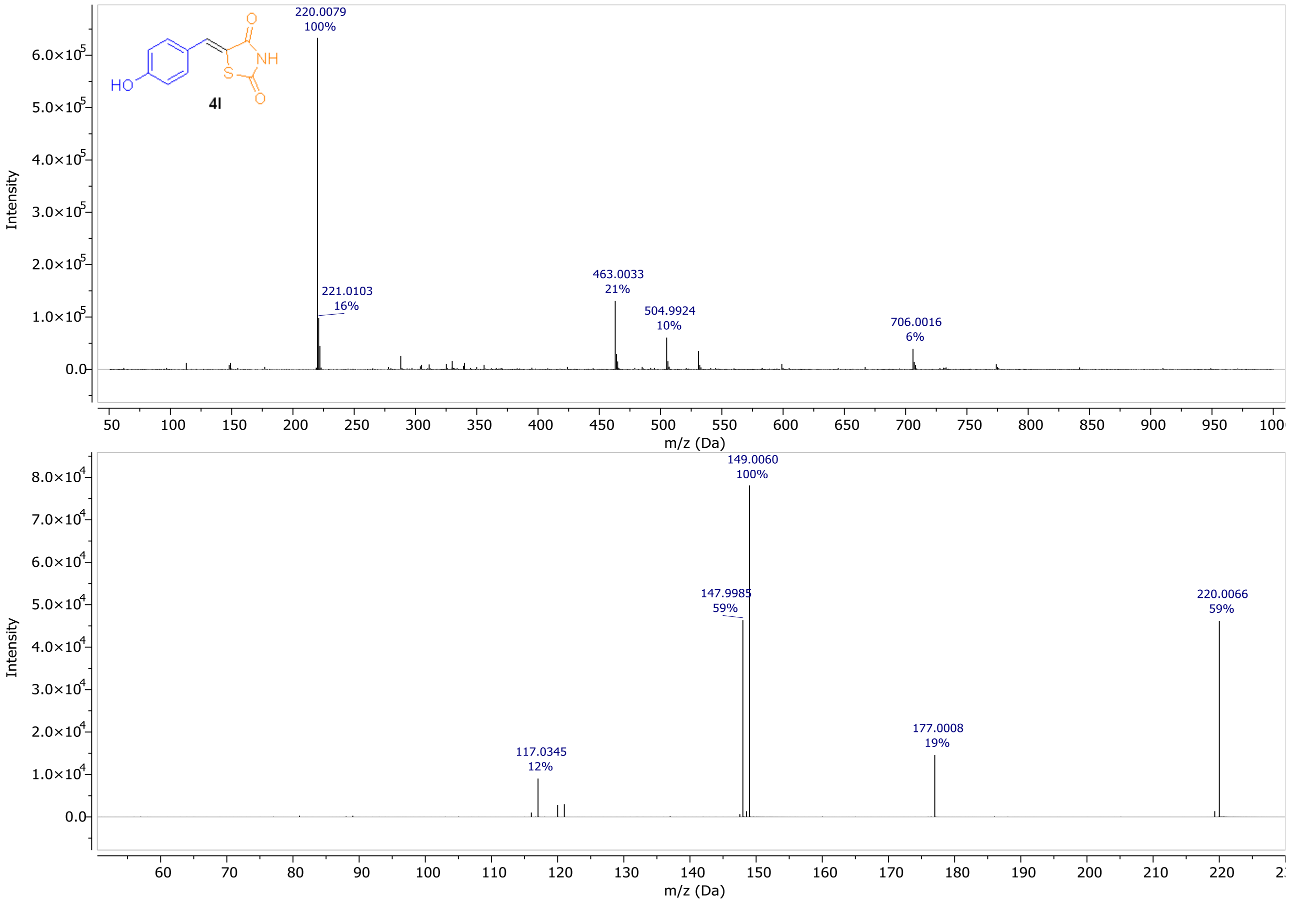

Figure S157. HRMS (ESI-QTOF) of compound **4l** and HRMS/MS for [M−H]⁻.

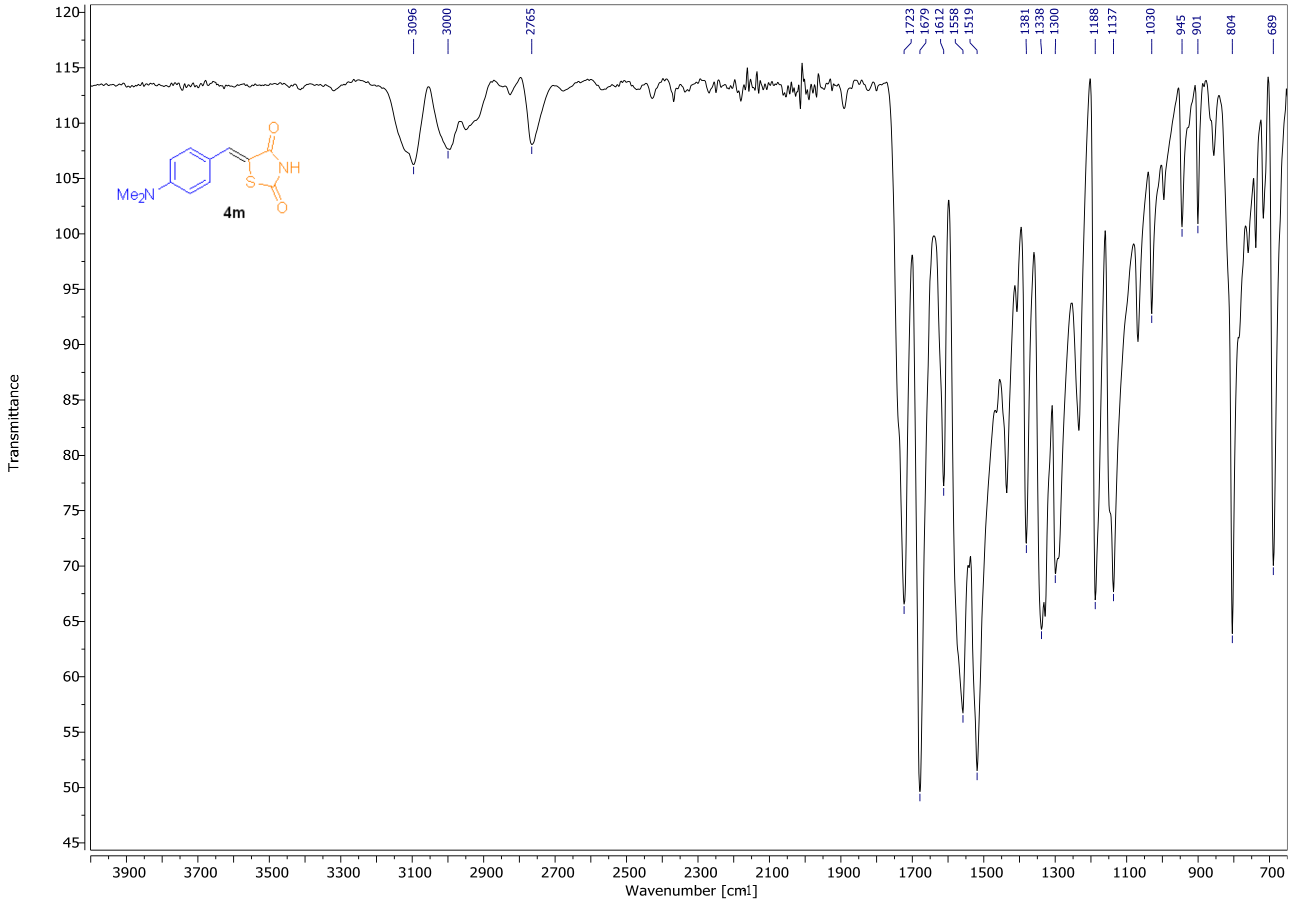

Figure S158. FTIR (ATR) of compound **4m**.

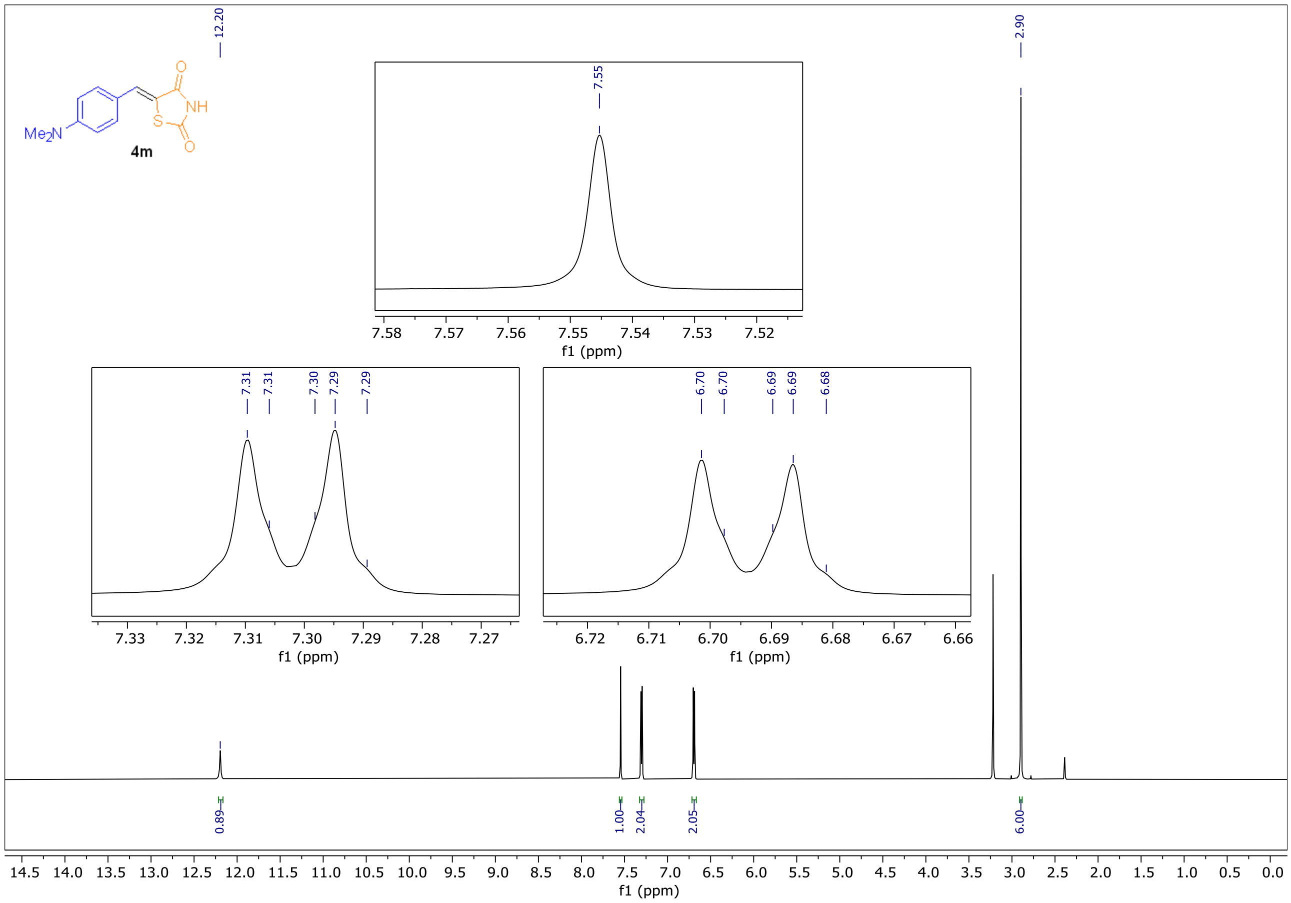

Figure S159. ^{1}H NMR spectrum (600 MHz, DMSO-d_6) of compound **4m**.

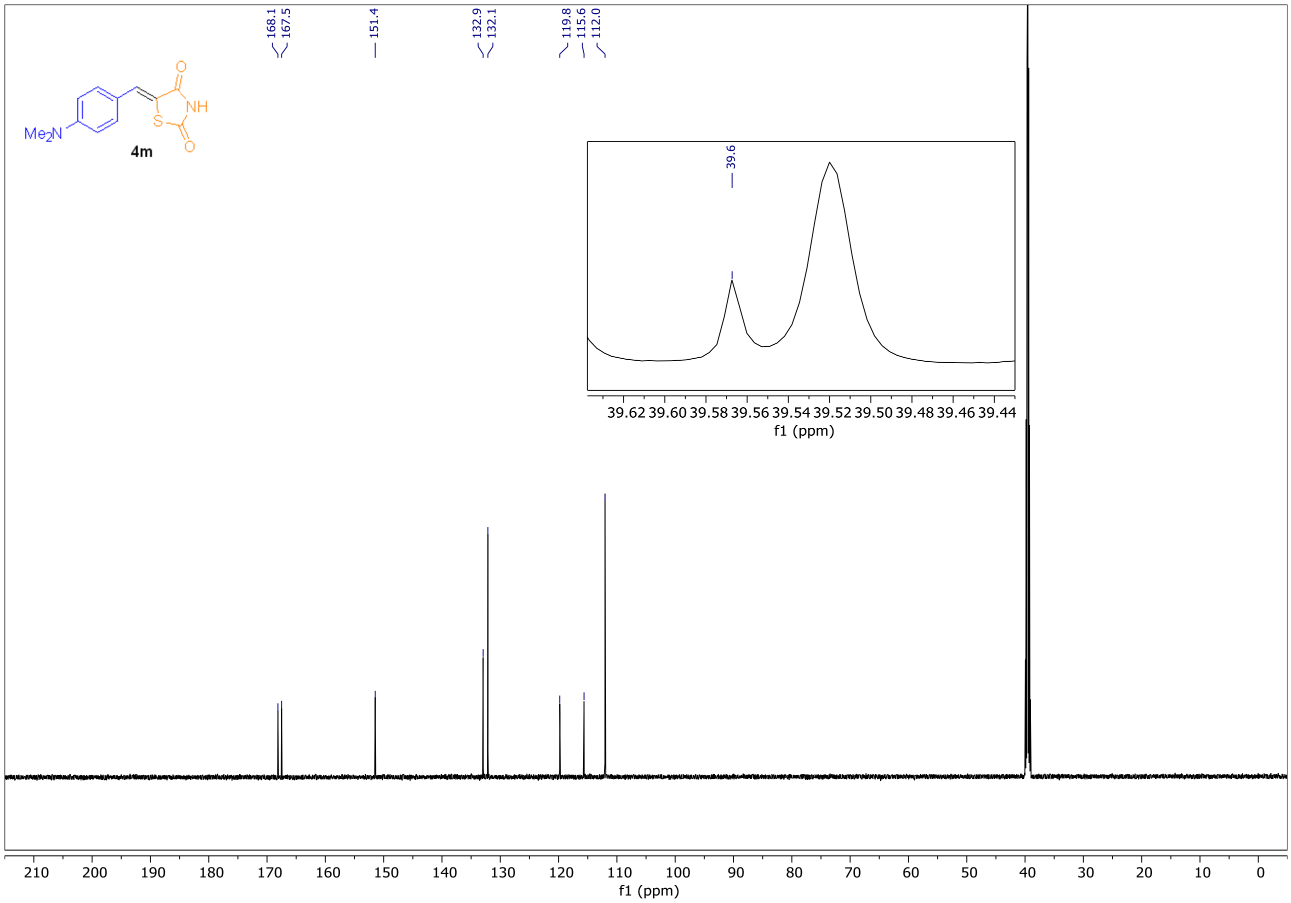

Figure S160. ^{13}C NMR spectrum (151 MHz, DMSO-d_6) of compound **4m**.

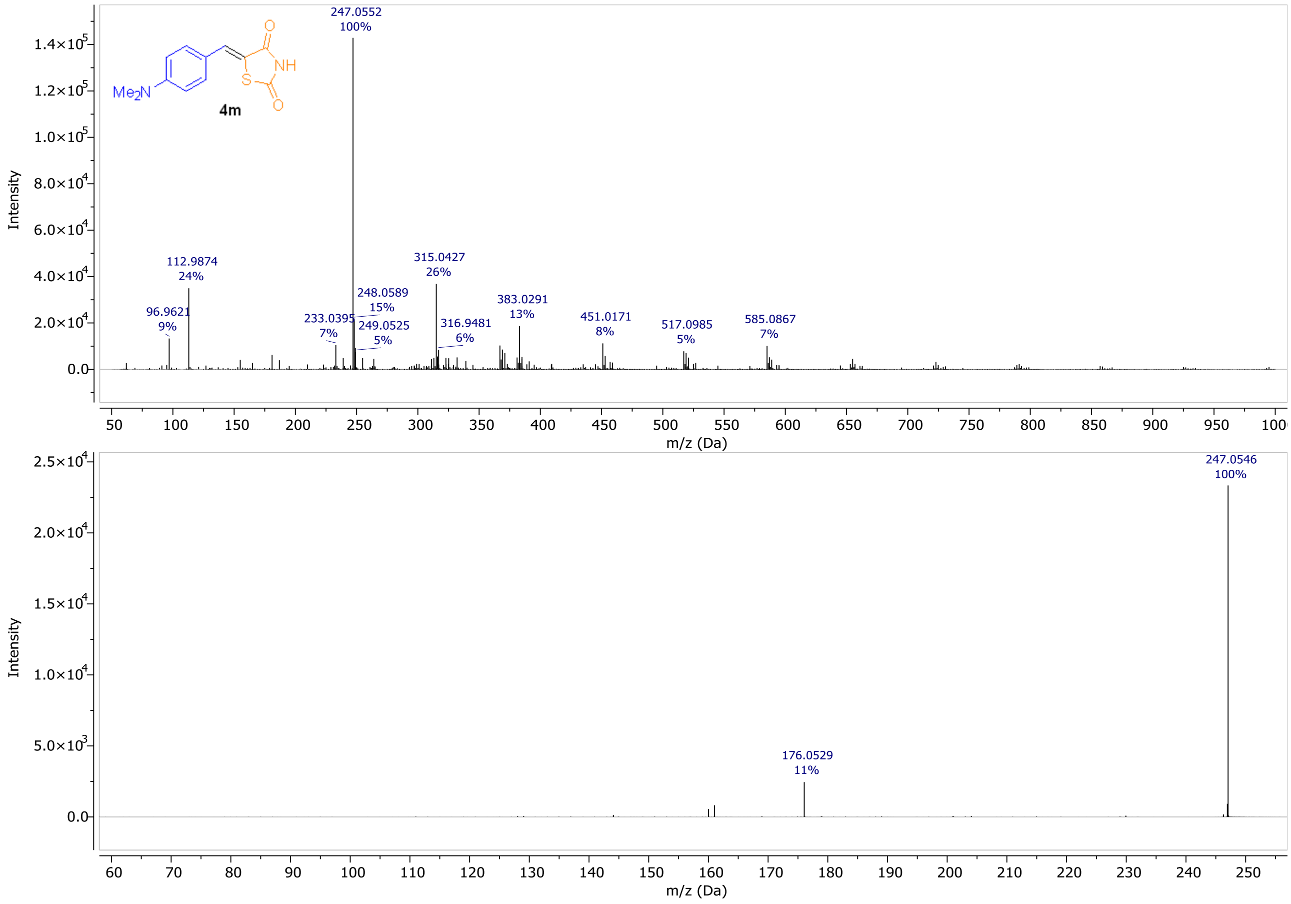

Figure S161. HRMS (ESI-QTOF) of compound **4m** and HRMS/MS for [M−H]⁻.

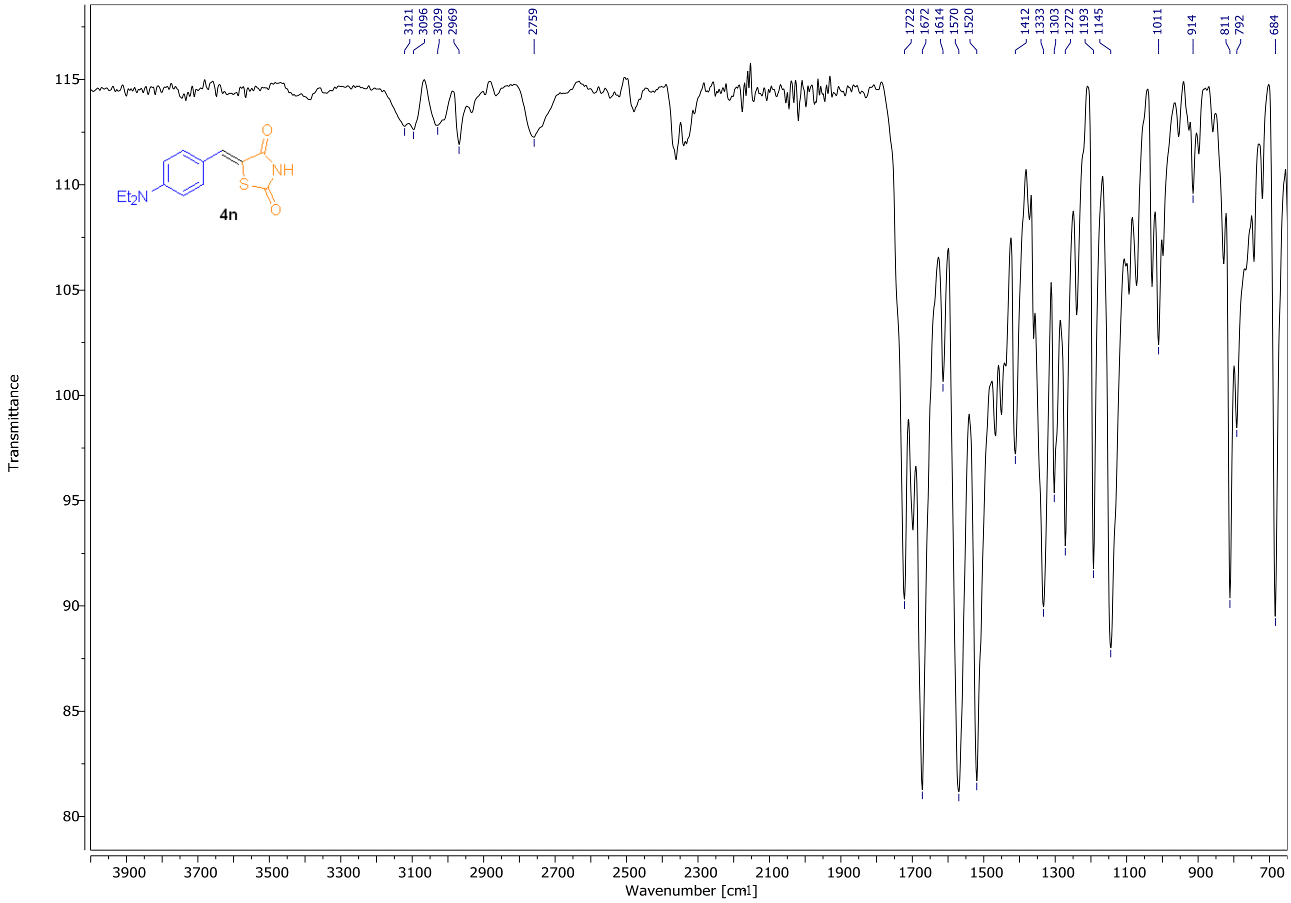

Figure S162. FTIR (ATR) of compound **4n**.

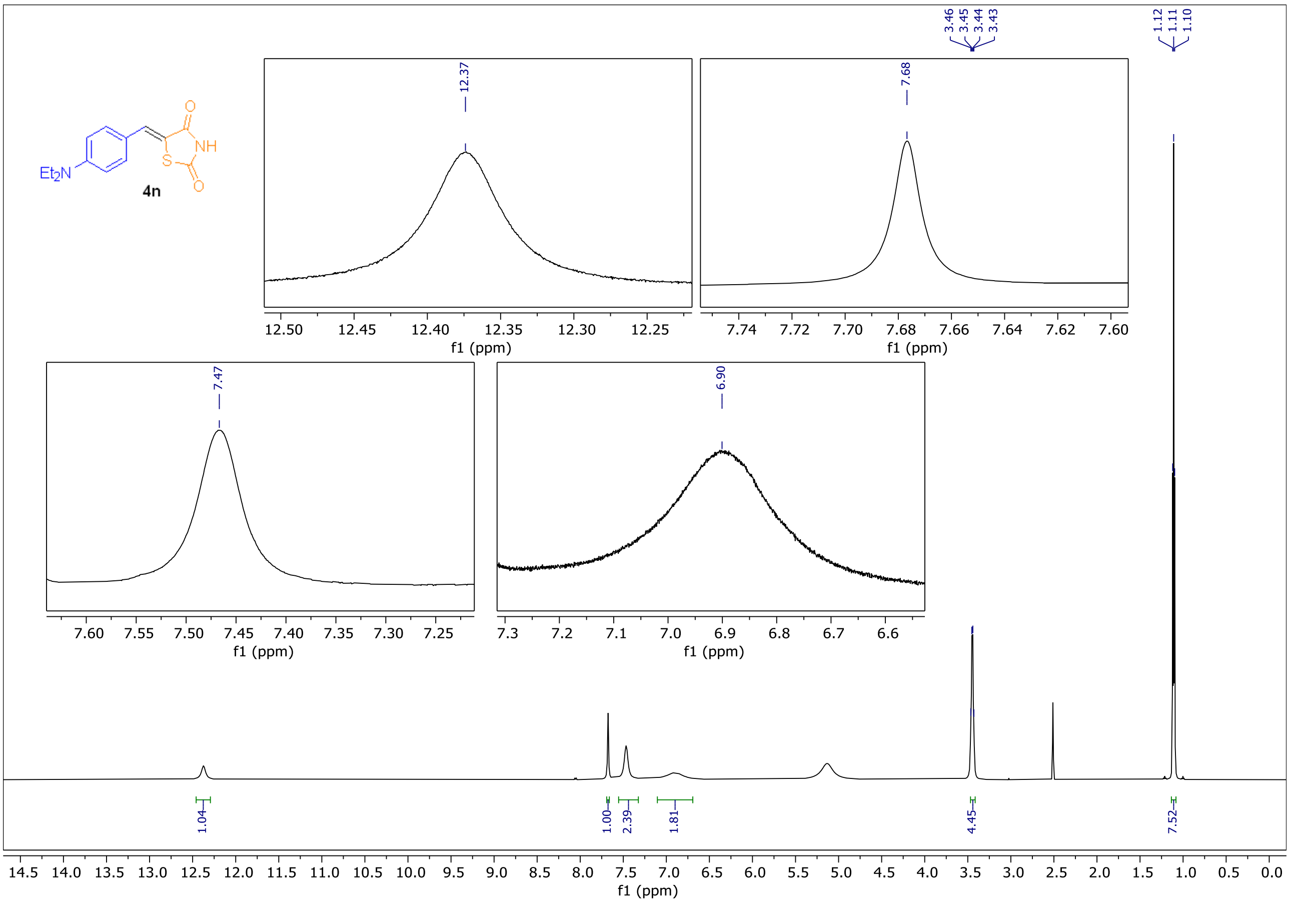

Figure S163. ^{1}H NMR spectrum (600 MHz, DMSO-d_6) of compound **4n**.

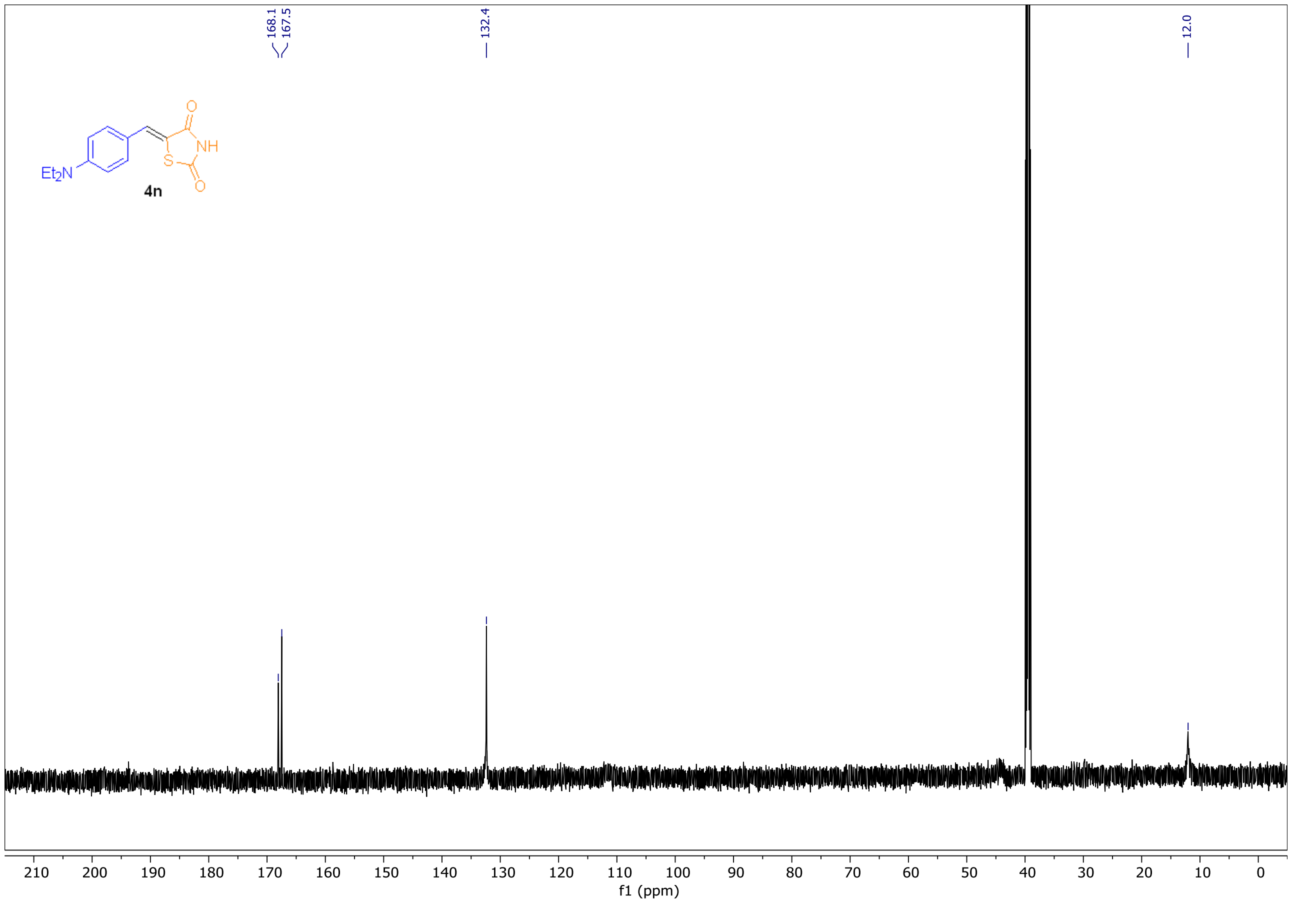

Figure S164. ^{13}C NMR spectrum (151 MHz, DMSO-d_6) of compound **4n**.

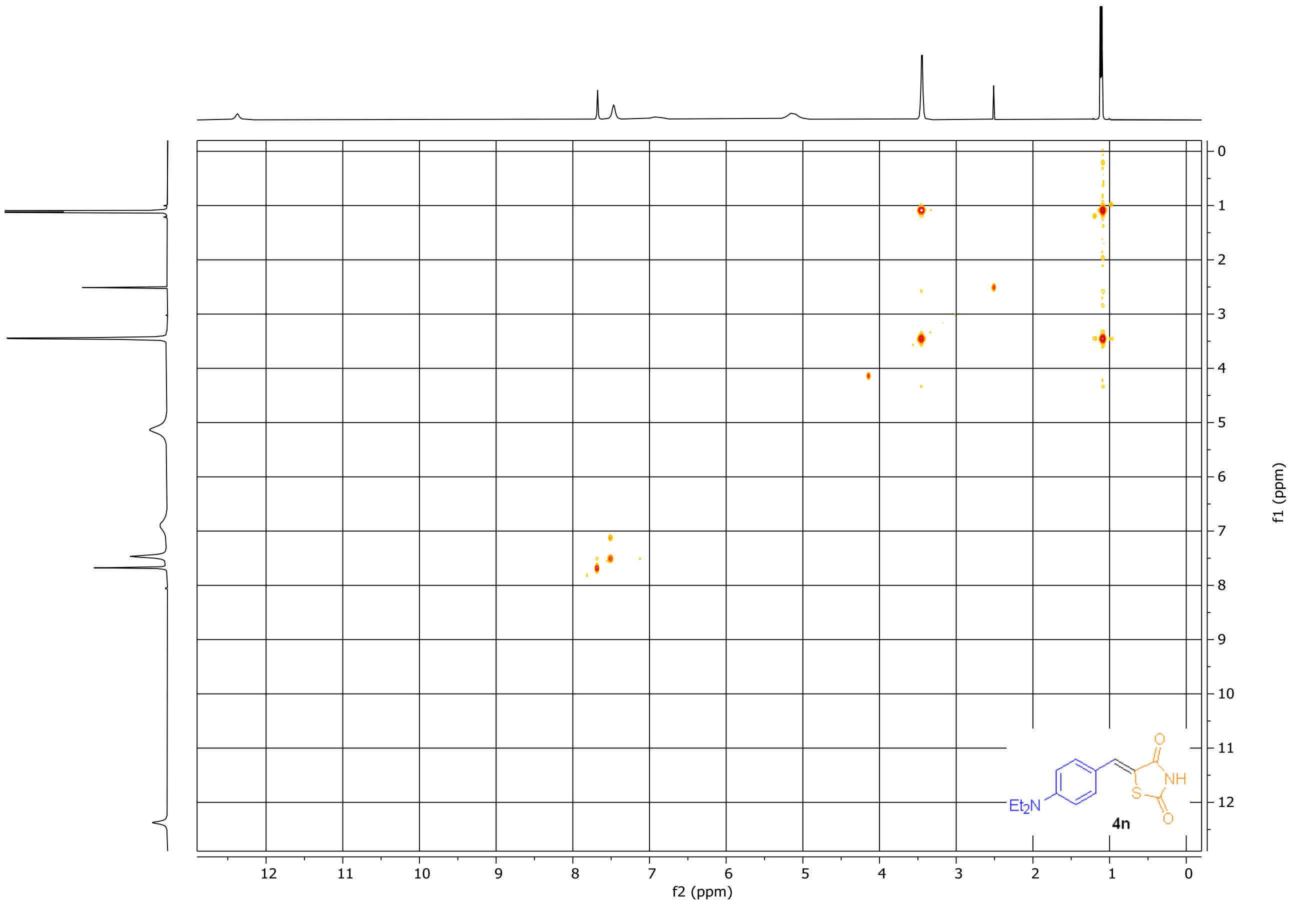

Figure S165. 2D COSY NMR spectrum (600 MHz, DMSO-d_6) of compound **4n**.

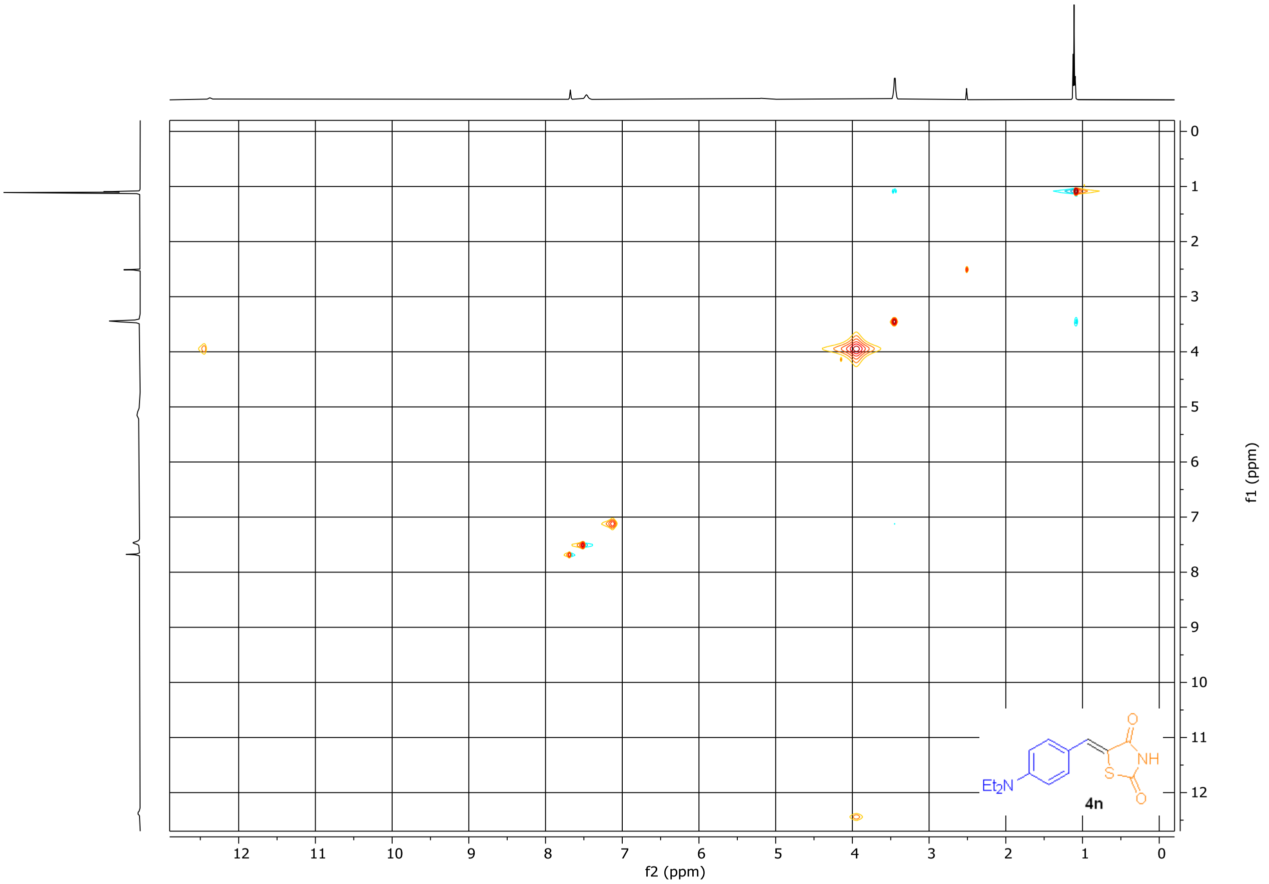

Figure S166. 2D NOESY NMR spectrum (600 MHz, DMSO-d_6) of compound **4n**.

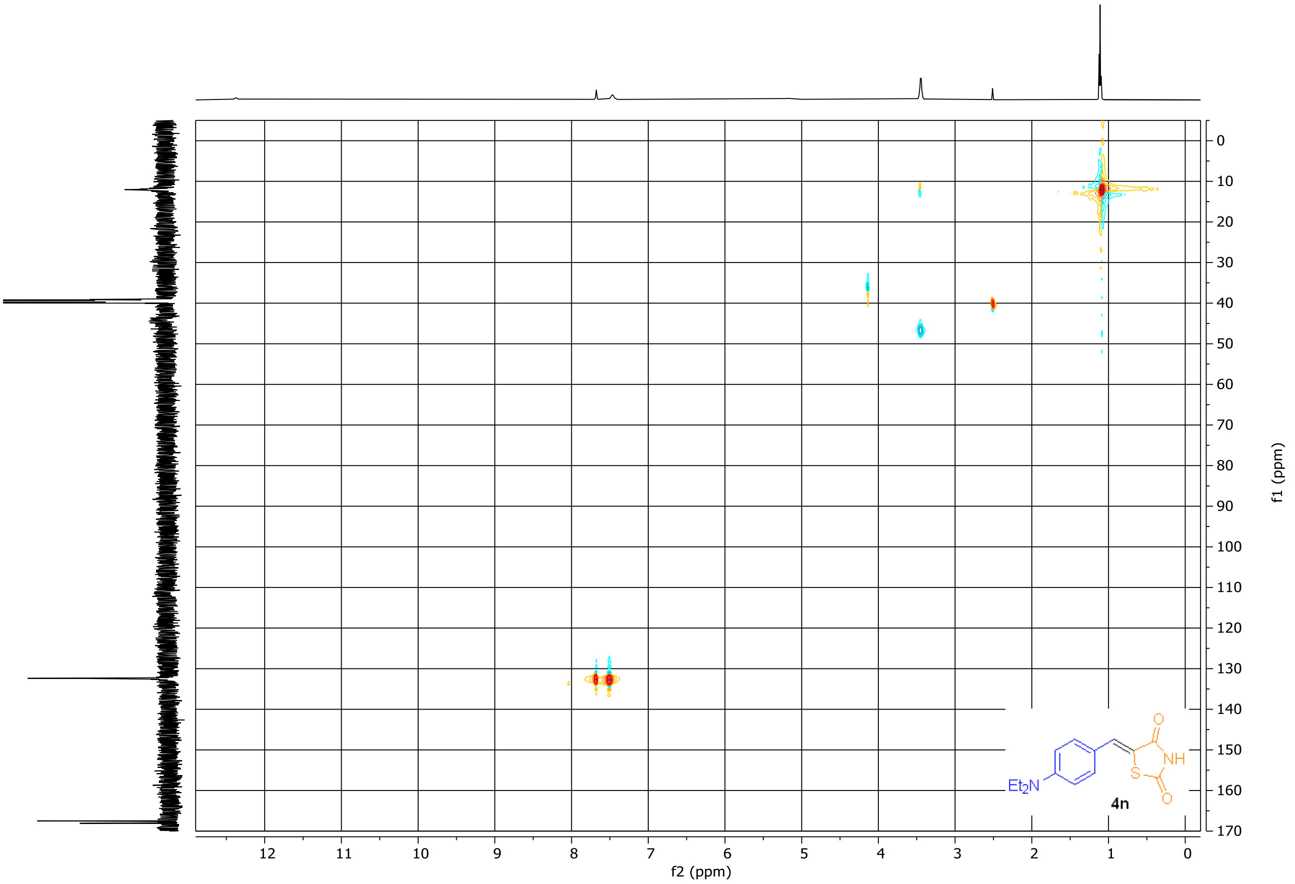

Figure S167. 2D HSQC NMR spectrum (600 MHz, DMSO-d_6) of compound **4n**.

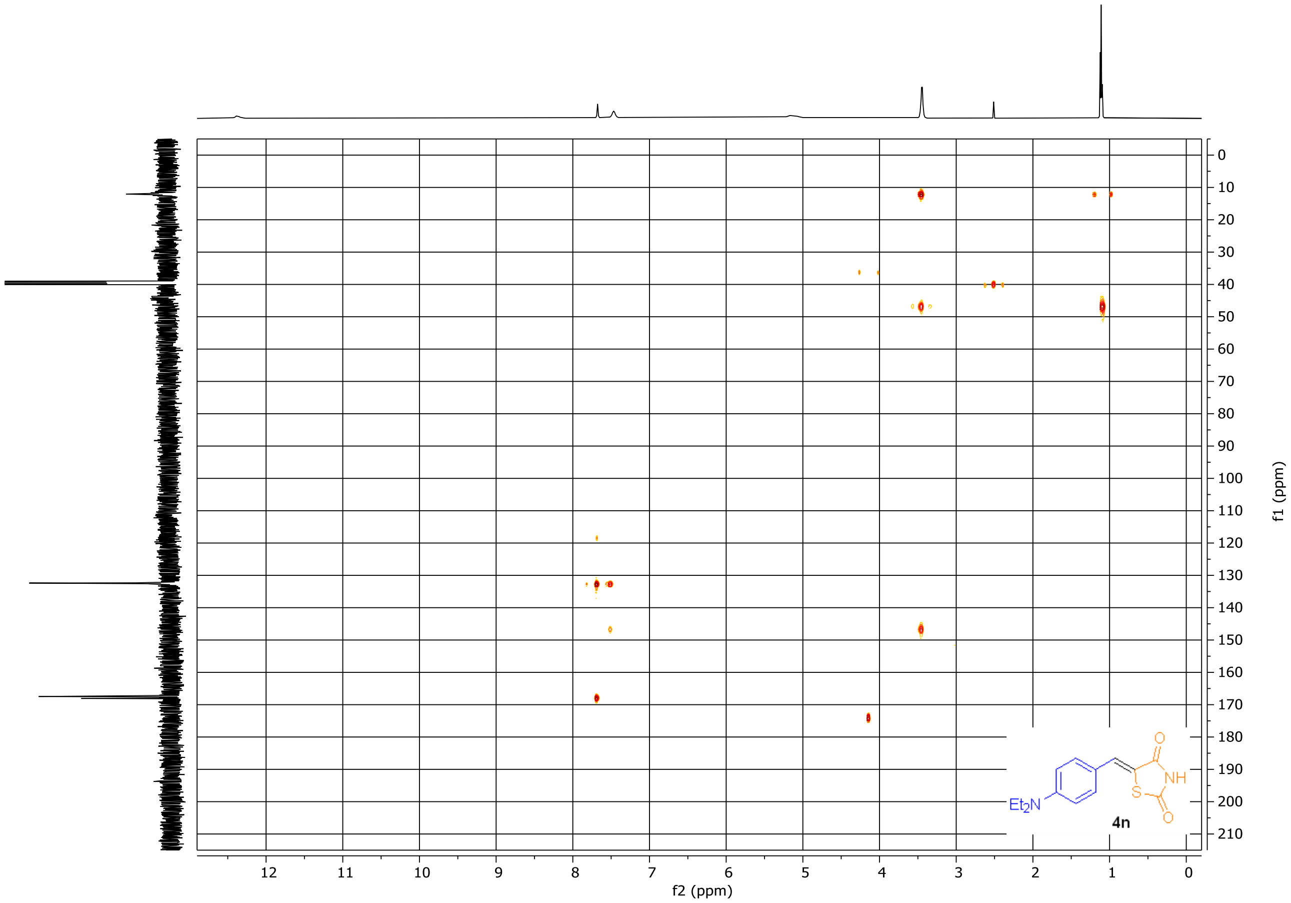

Figure S168. 2D HMBC NMR spectrum (600 MHz, DMSO-d_6) of compound **4n**.

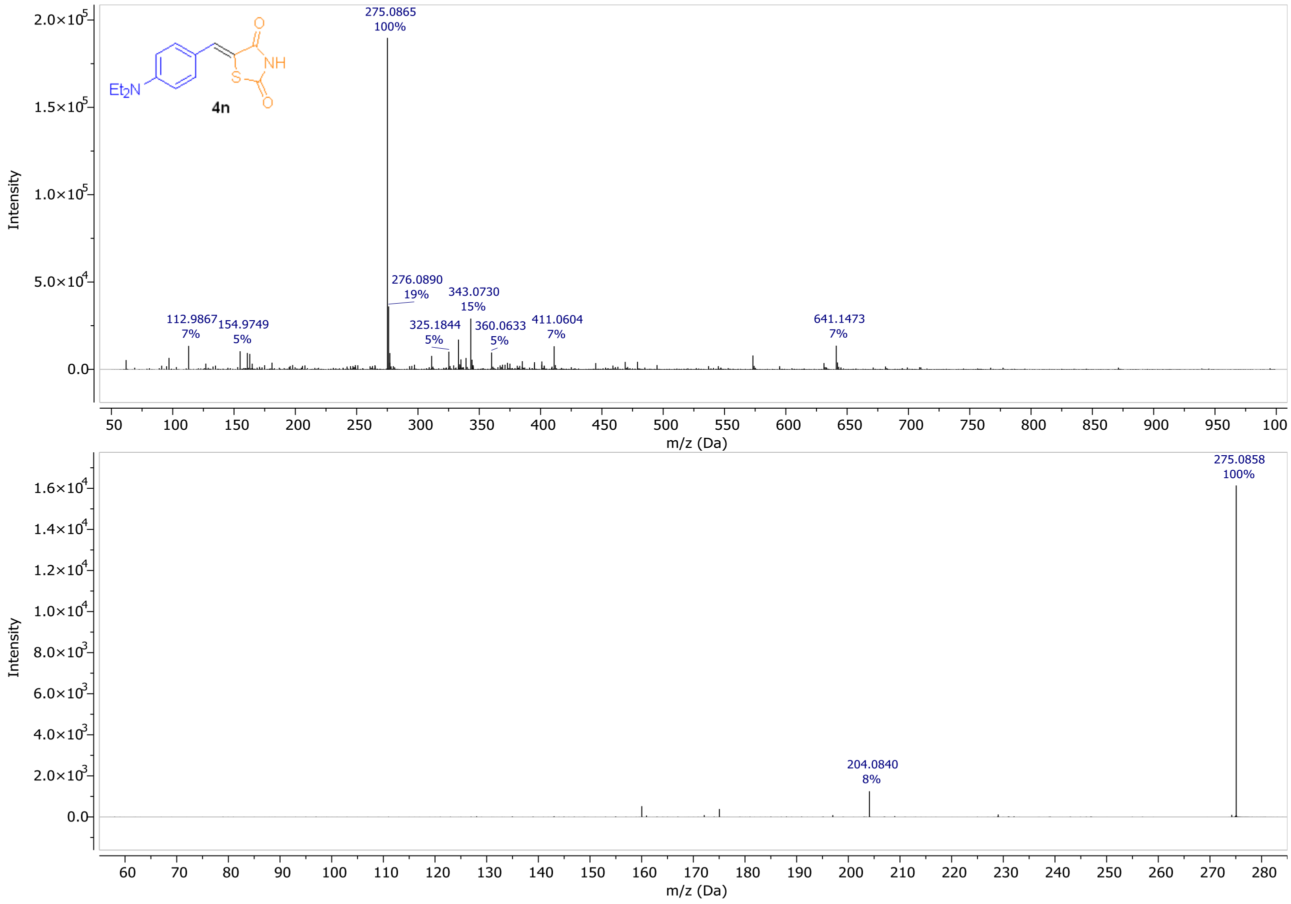

Figure S169. HRMS (ESI-QTOF) of compound **4n** and HRMS/MS for $[M-H]^-$.

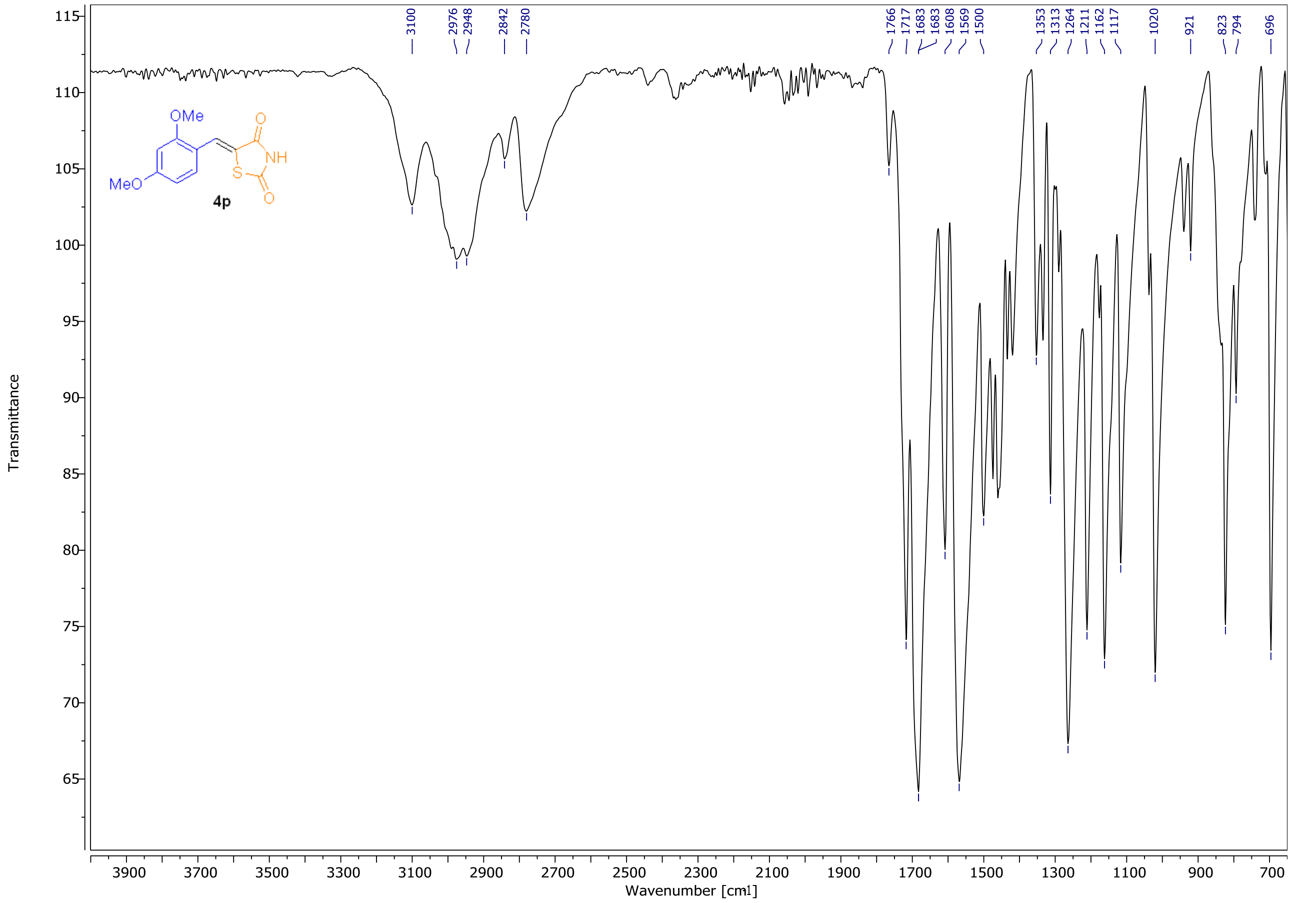

Figure S170. FTIR (ATR) of compound **4p**.

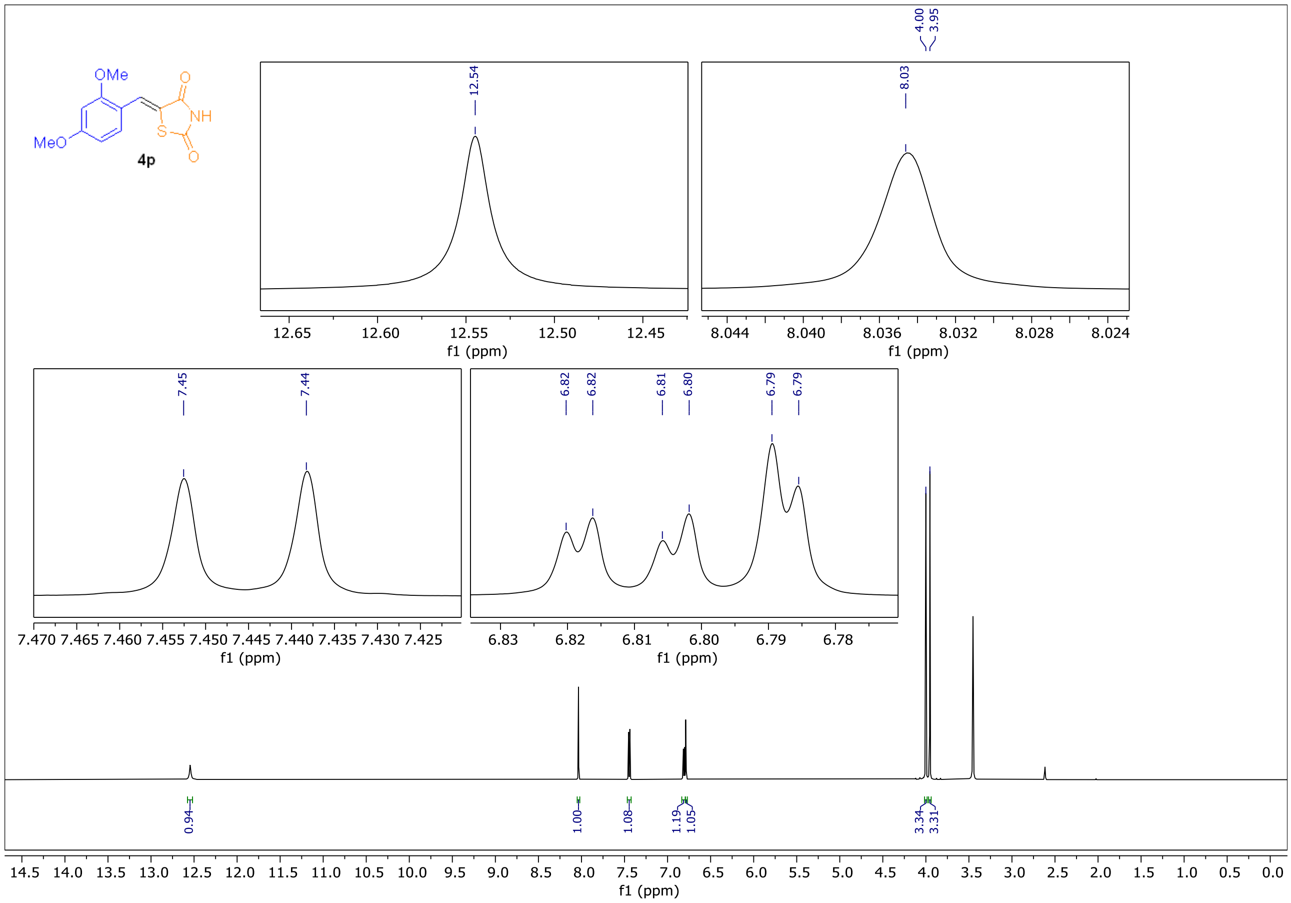

Figure S171. ^{1}H NMR spectrum (600 MHz, DMSO-d_6) of compound **4p**.

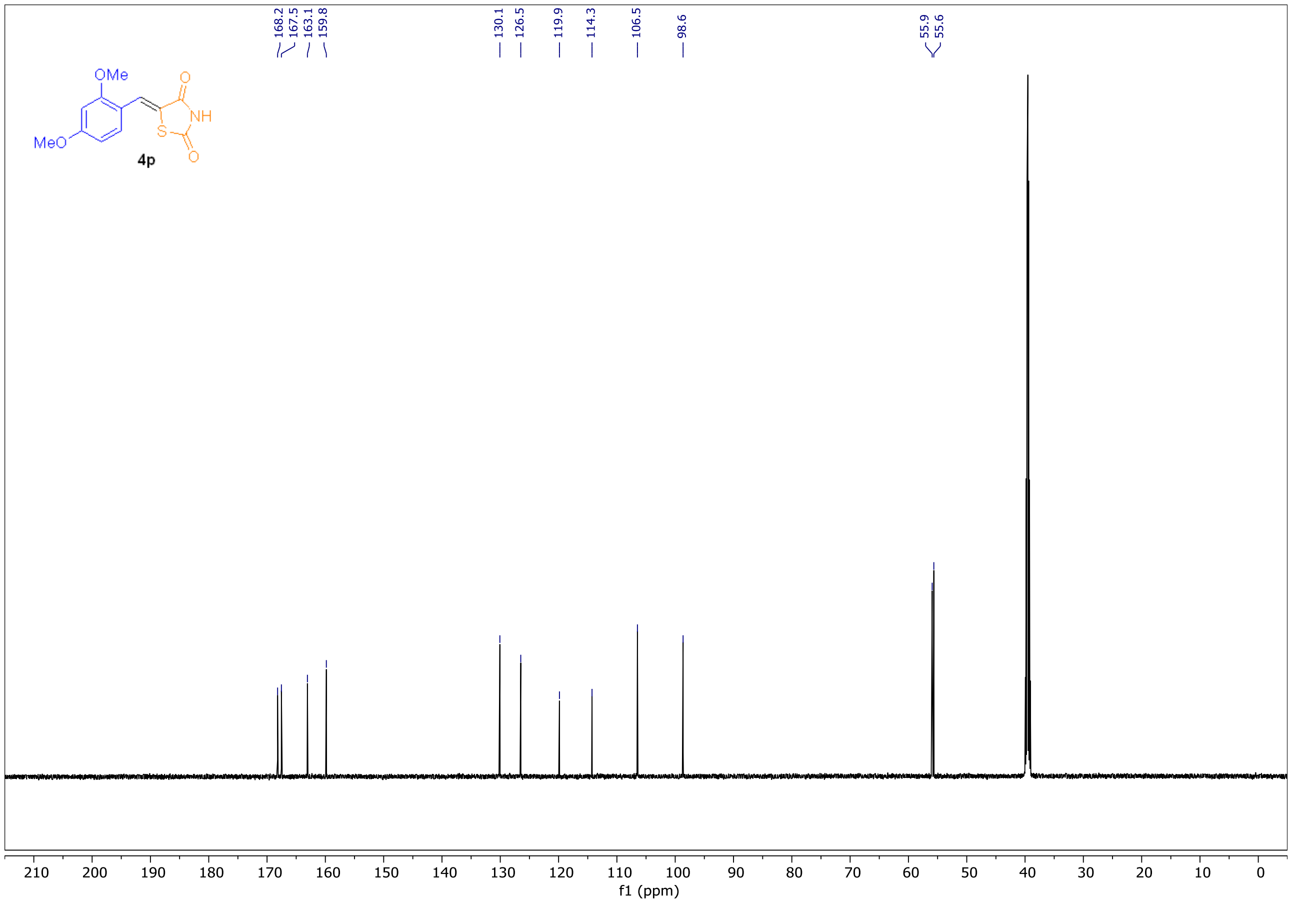

Figure S172. ^{13}C NMR spectrum (151 MHz, DMSO-d_6) of compound **4p**.

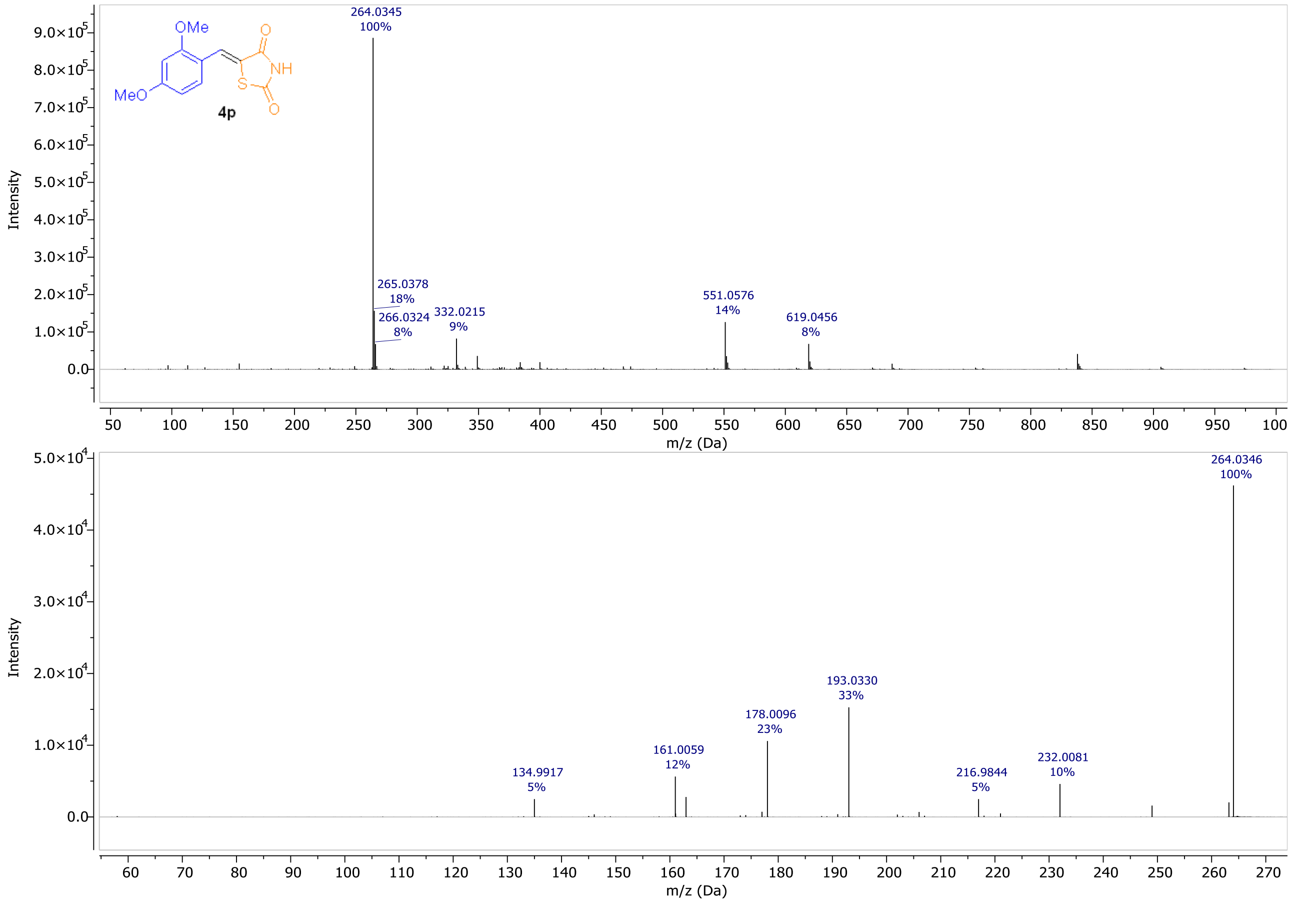

Figure S173. HRMS (ESI-QTOF) of compound **4p** and HRMS/MS for [M−H]⁻.

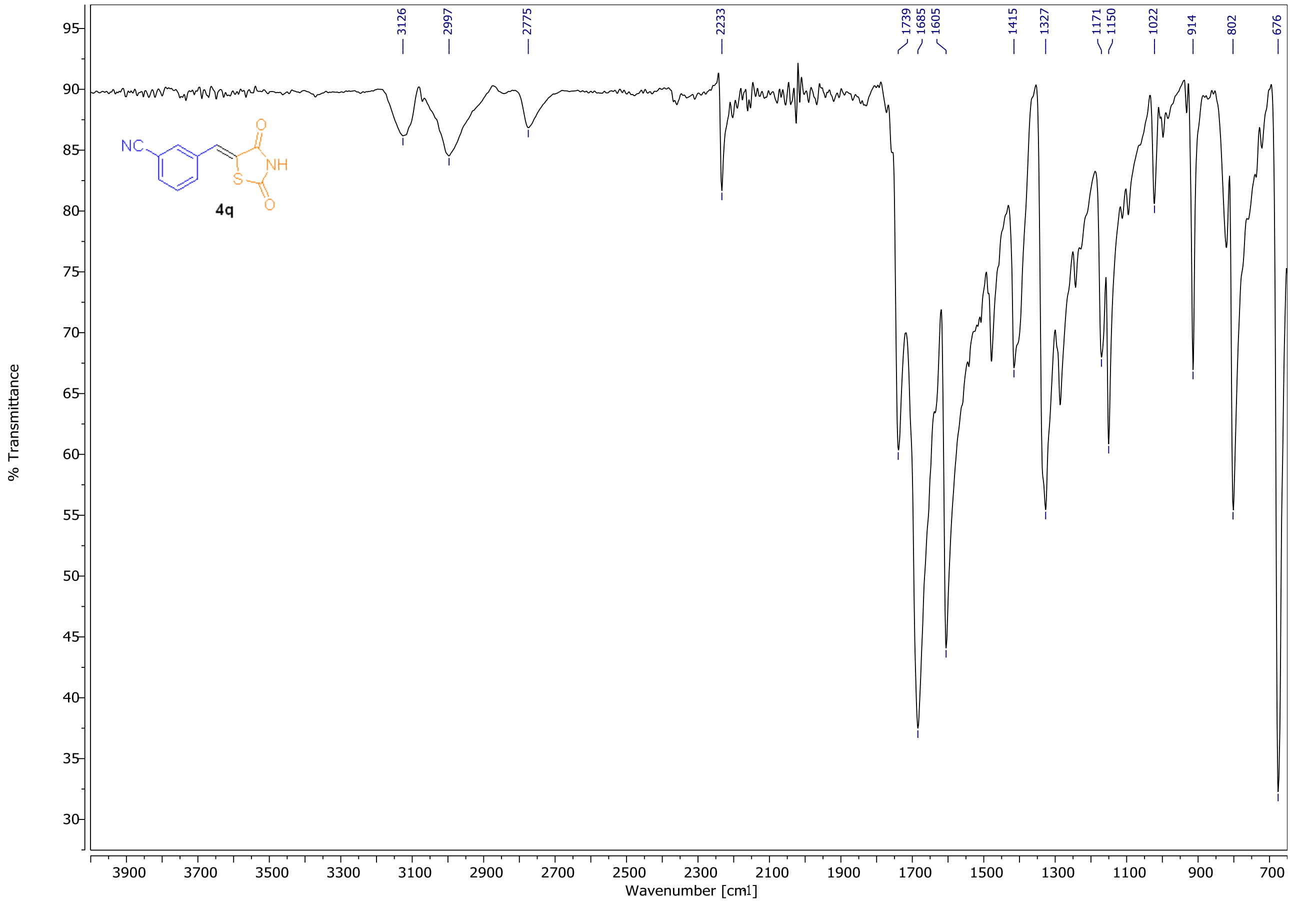

Figure S174. FTIR (ATR) of compound **4q**.

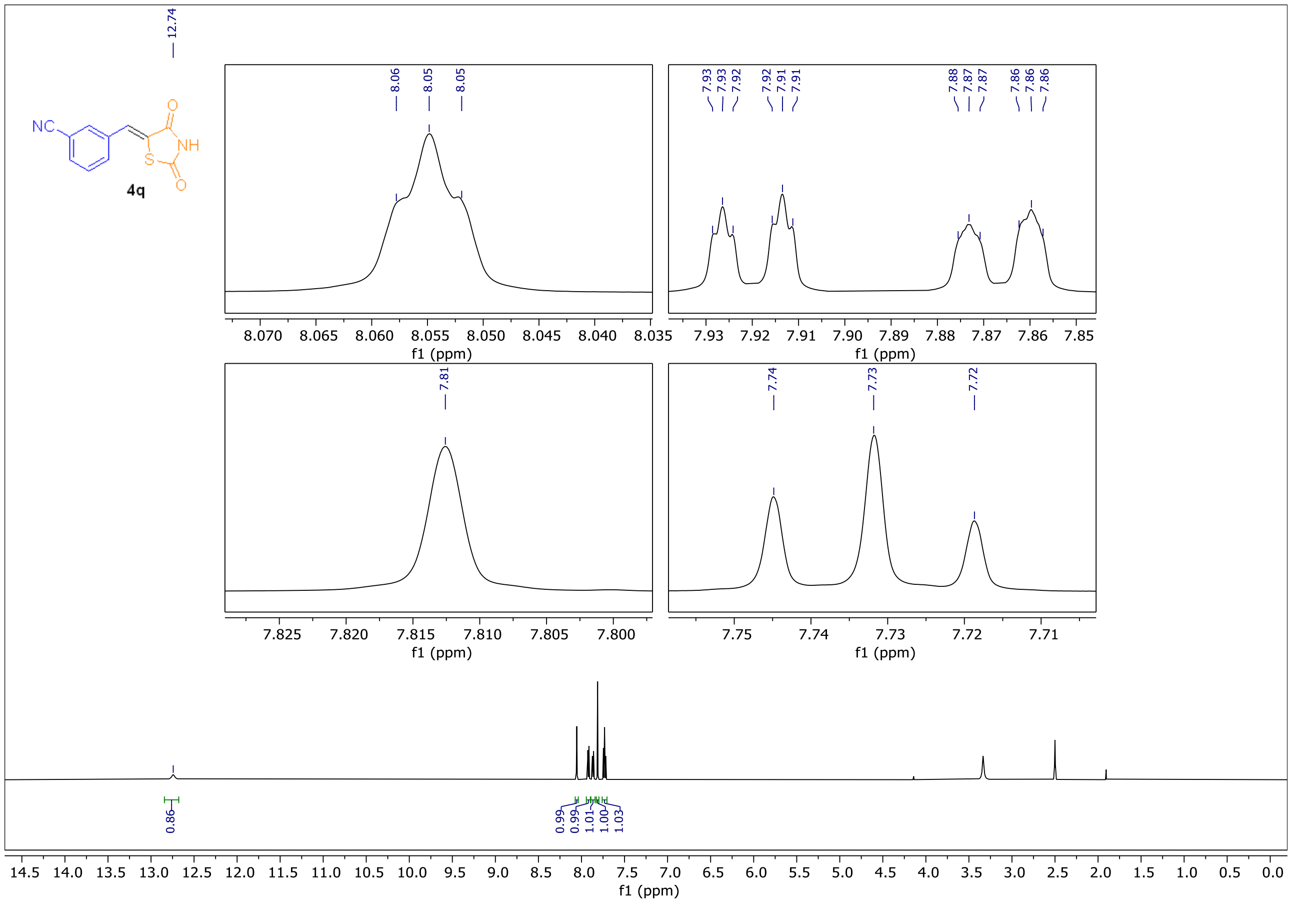

Figure S175. ^{1}H NMR spectrum (600 MHz, DMSO-d_6) of compound **4q**.

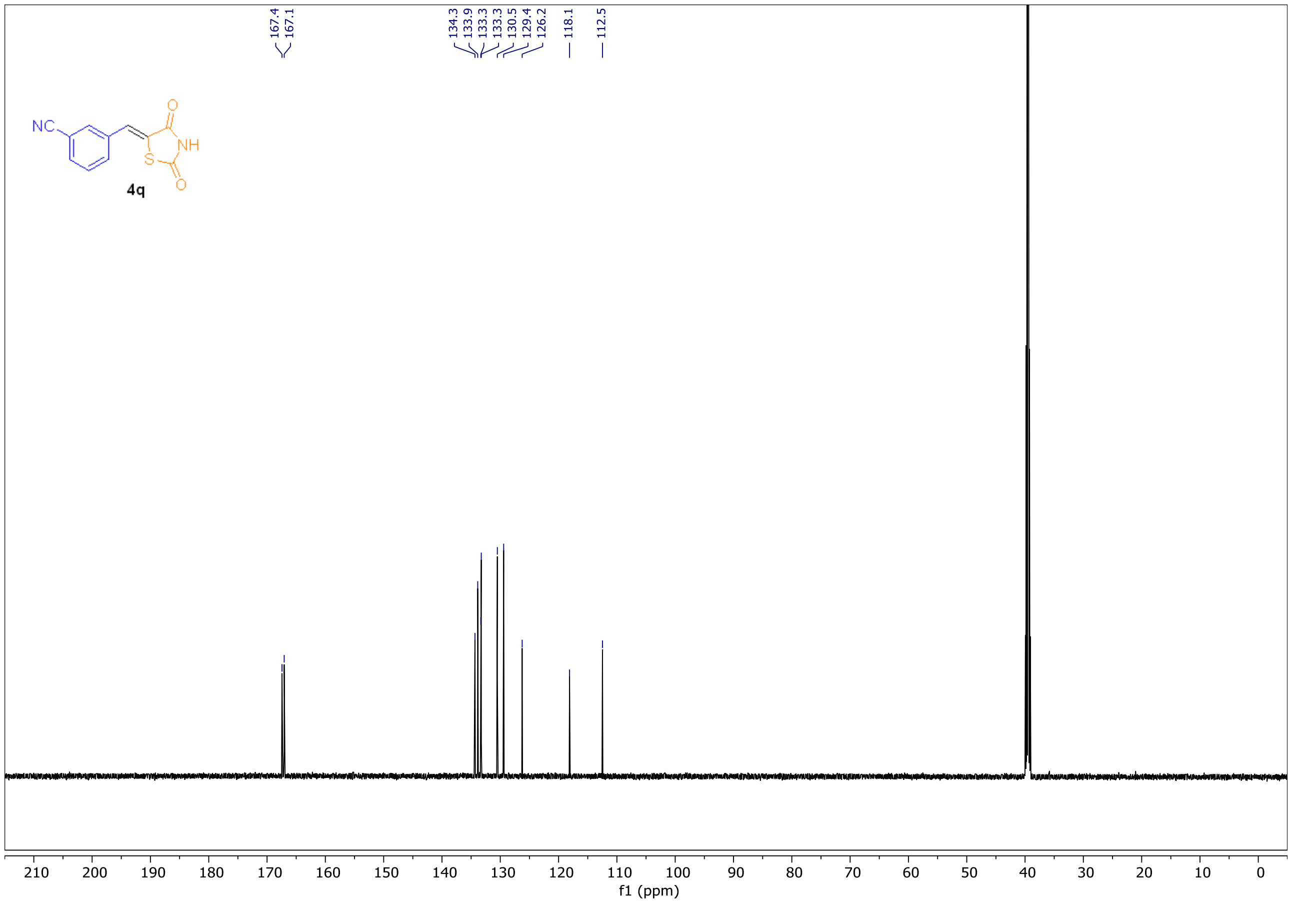

Figure S176. ^{13}C NMR spectrum (151 MHz, DMSO-d_6) of compound **4q**.

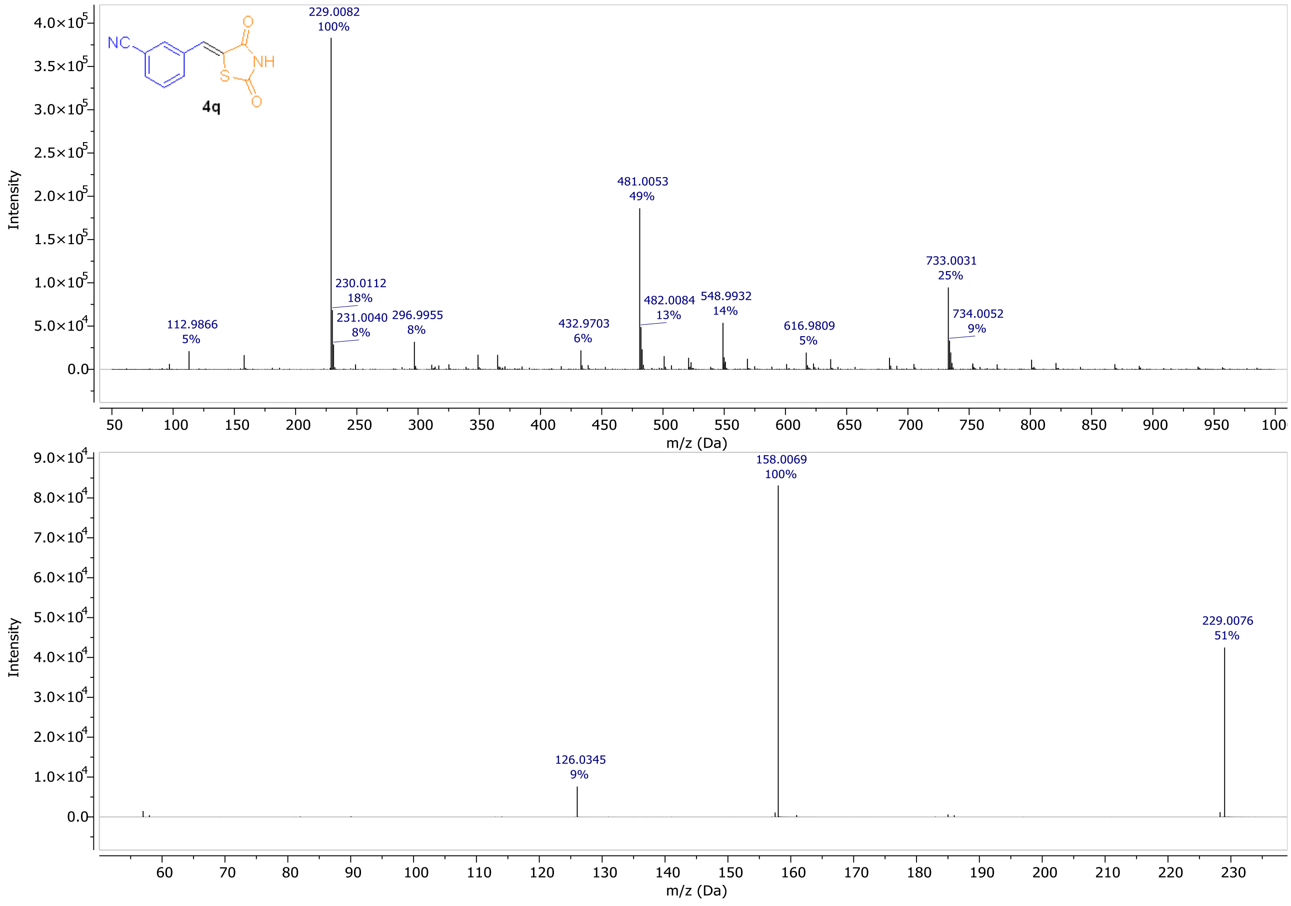

Figure S177. HRMS (ESI-QTOF) of compound **4q** and HRMS/MS for [M−H]$^-$.

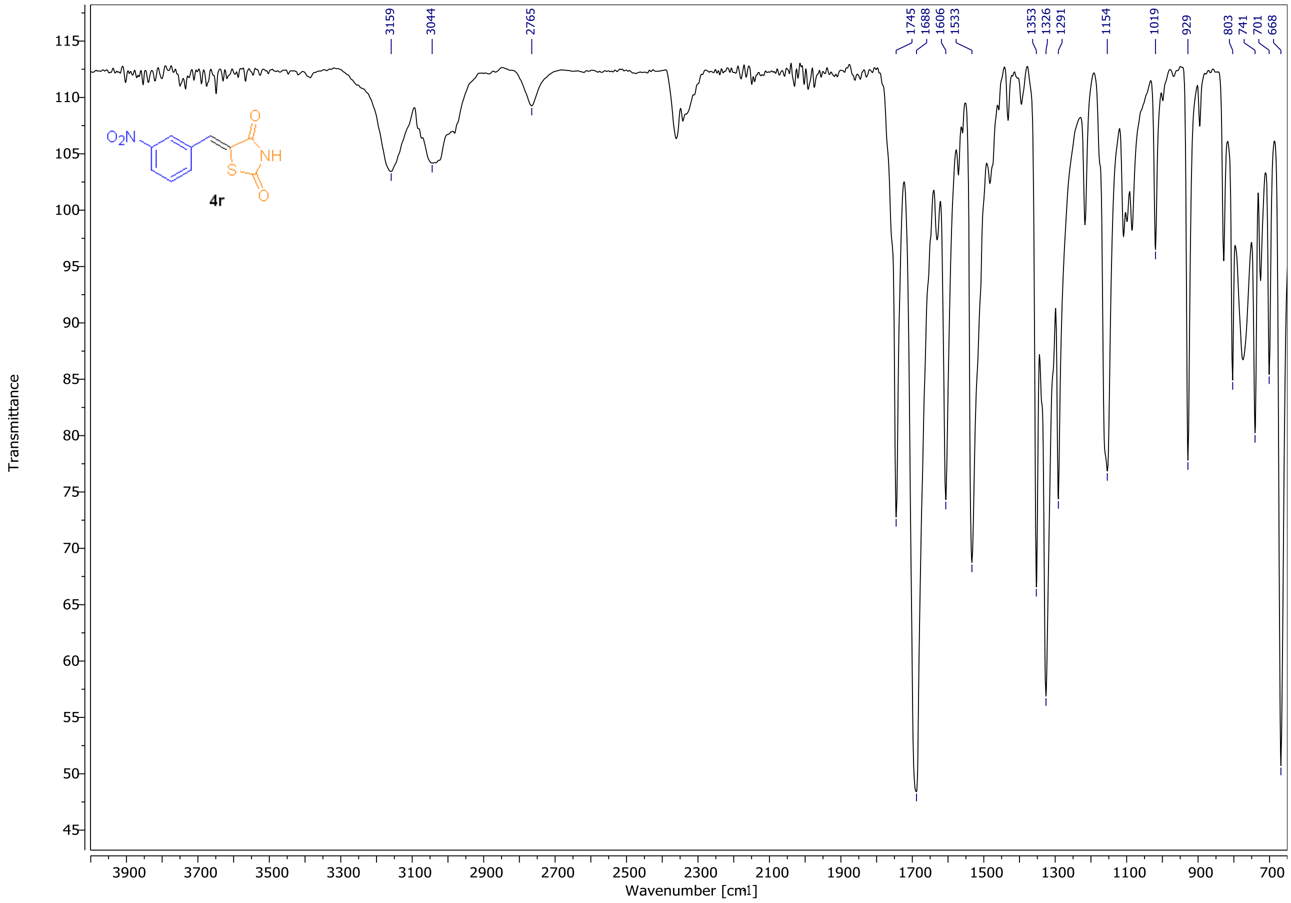

Figure S178. FTIR (ATR) of compound **4r**.

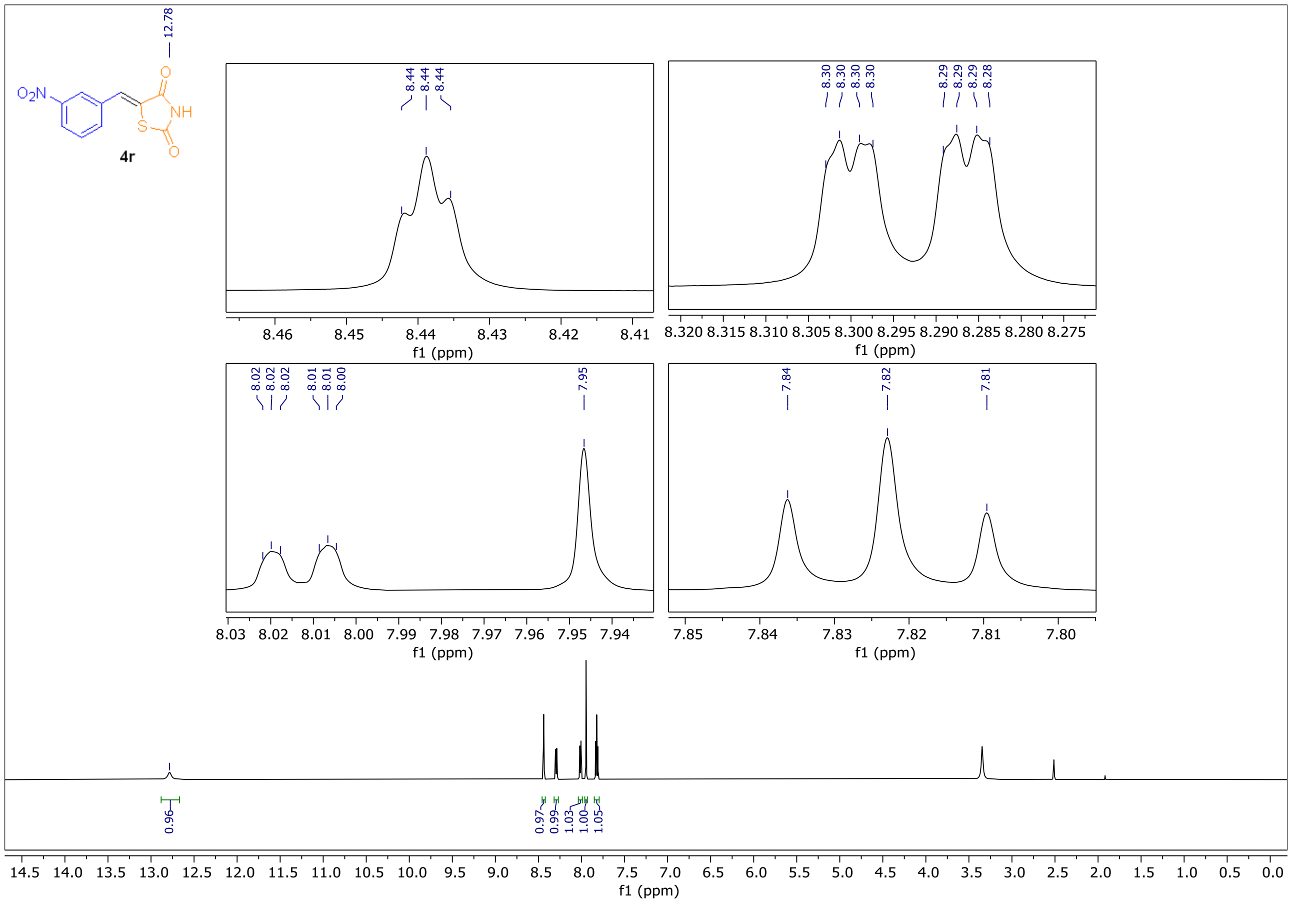

Figure S179. ^{1}H NMR spectrum (600 MHz, DMSO-d_6) of compound **4r**.

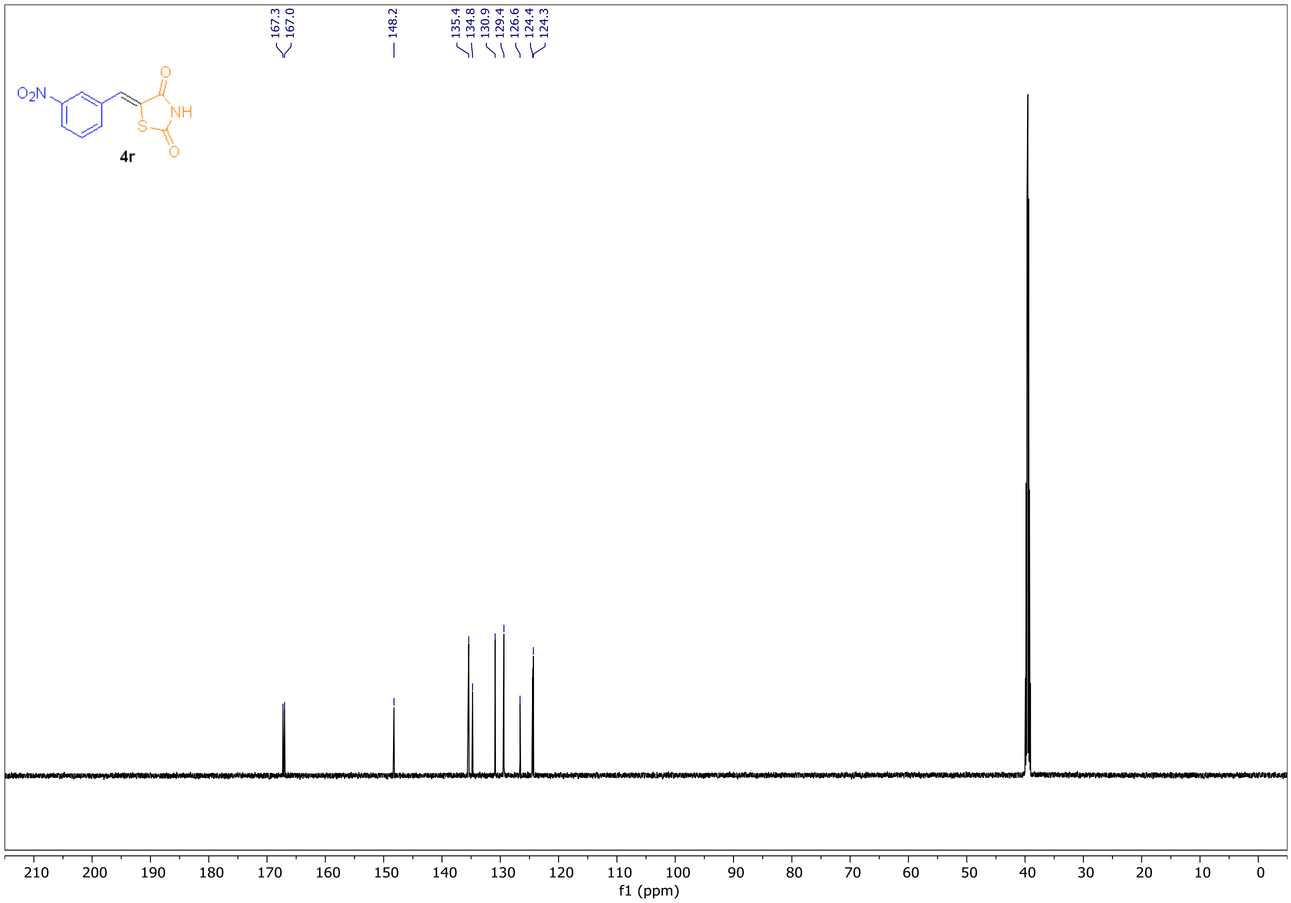

Figure S180. ^{13}C NMR spectrum (151 MHz, DMSO-d_6) of compound **4r**.

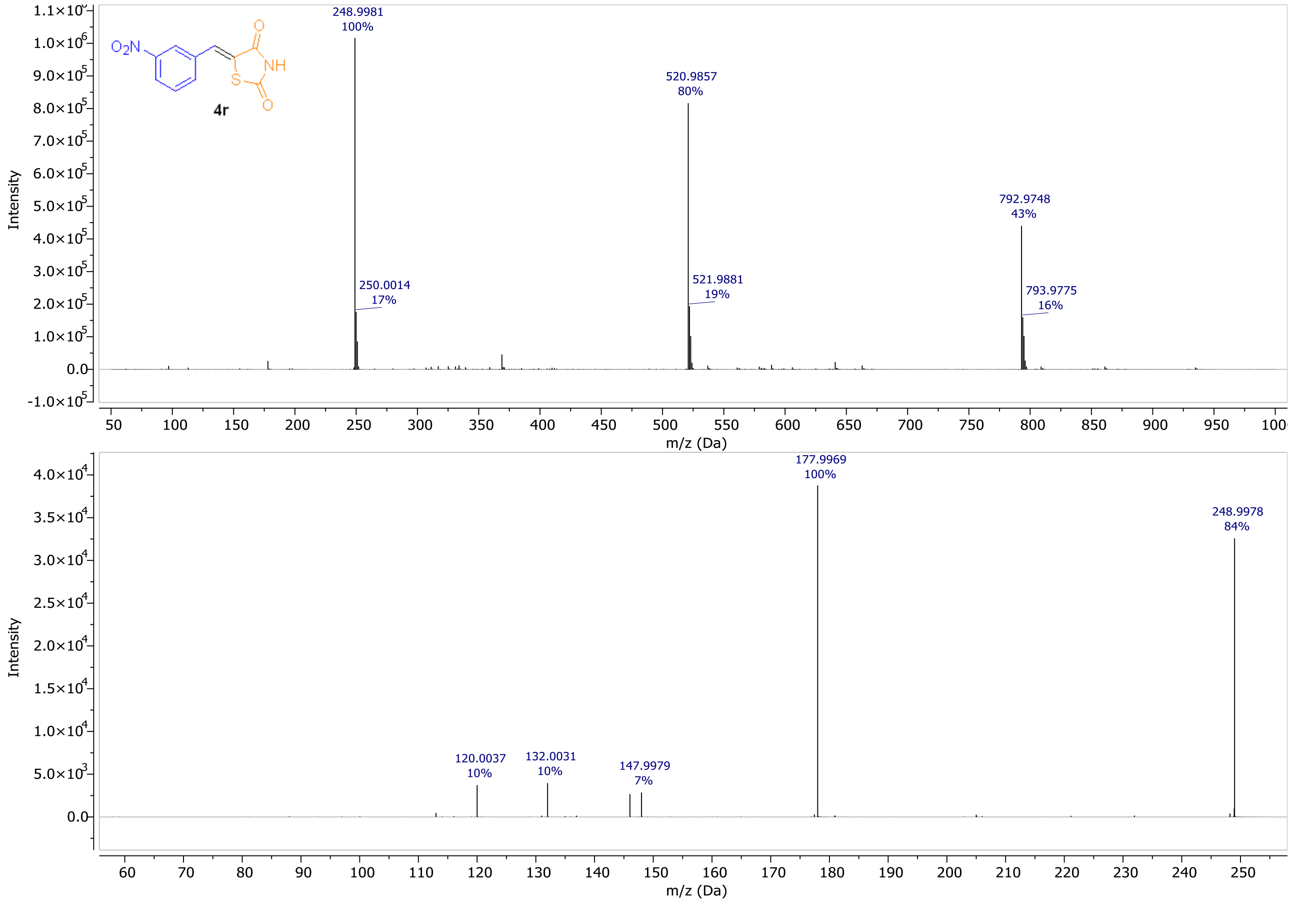

Figure S181. HRMS (ESI-QTOF) of compound **4r** and HRMS/MS for [M−H]$^-$.

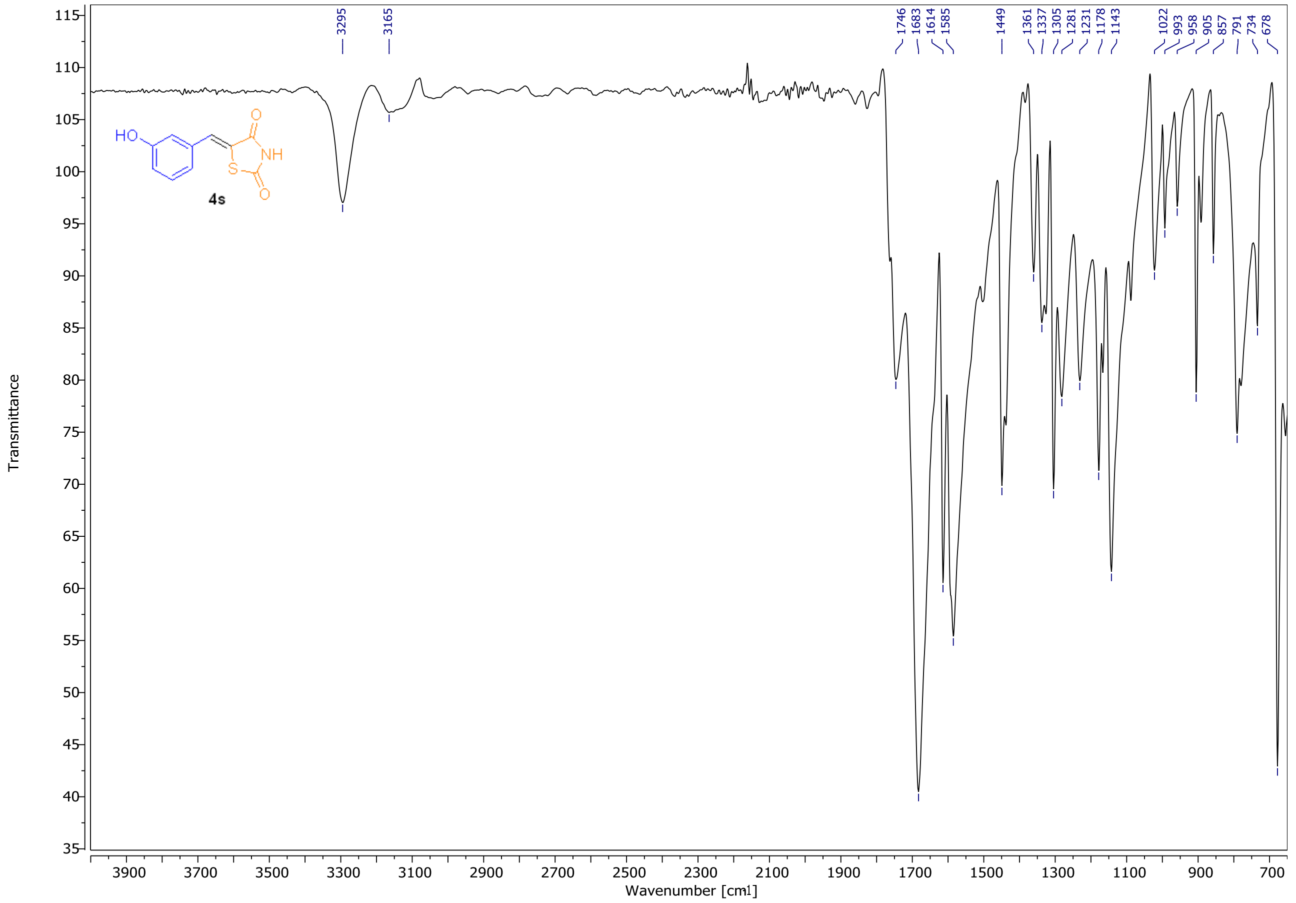

Figure S182. FTIR (ATR) of compound **4s**.

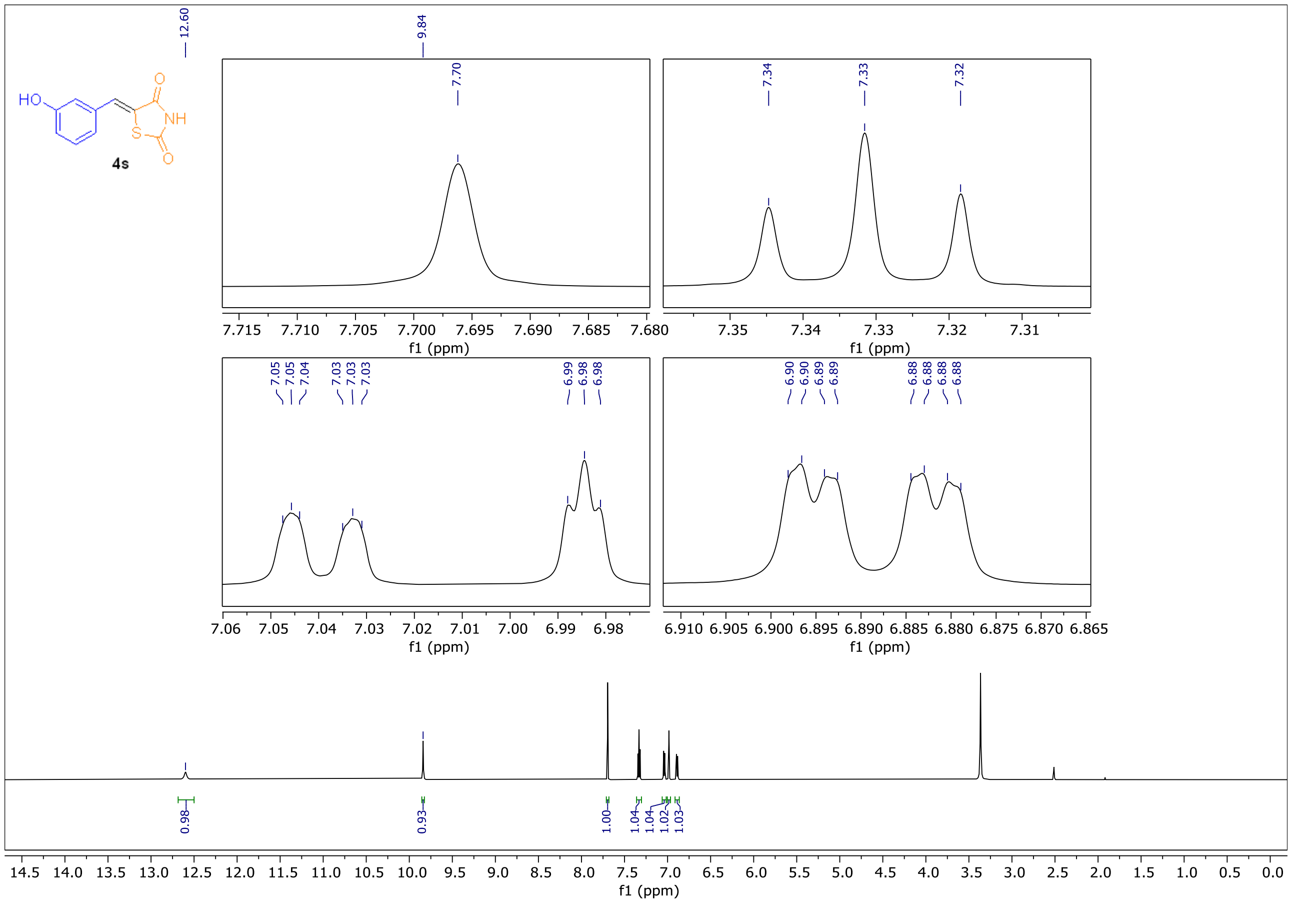

Figure S183. ^{1}H NMR spectrum (600 MHz, DMSO-d_6) of compound **4s**.

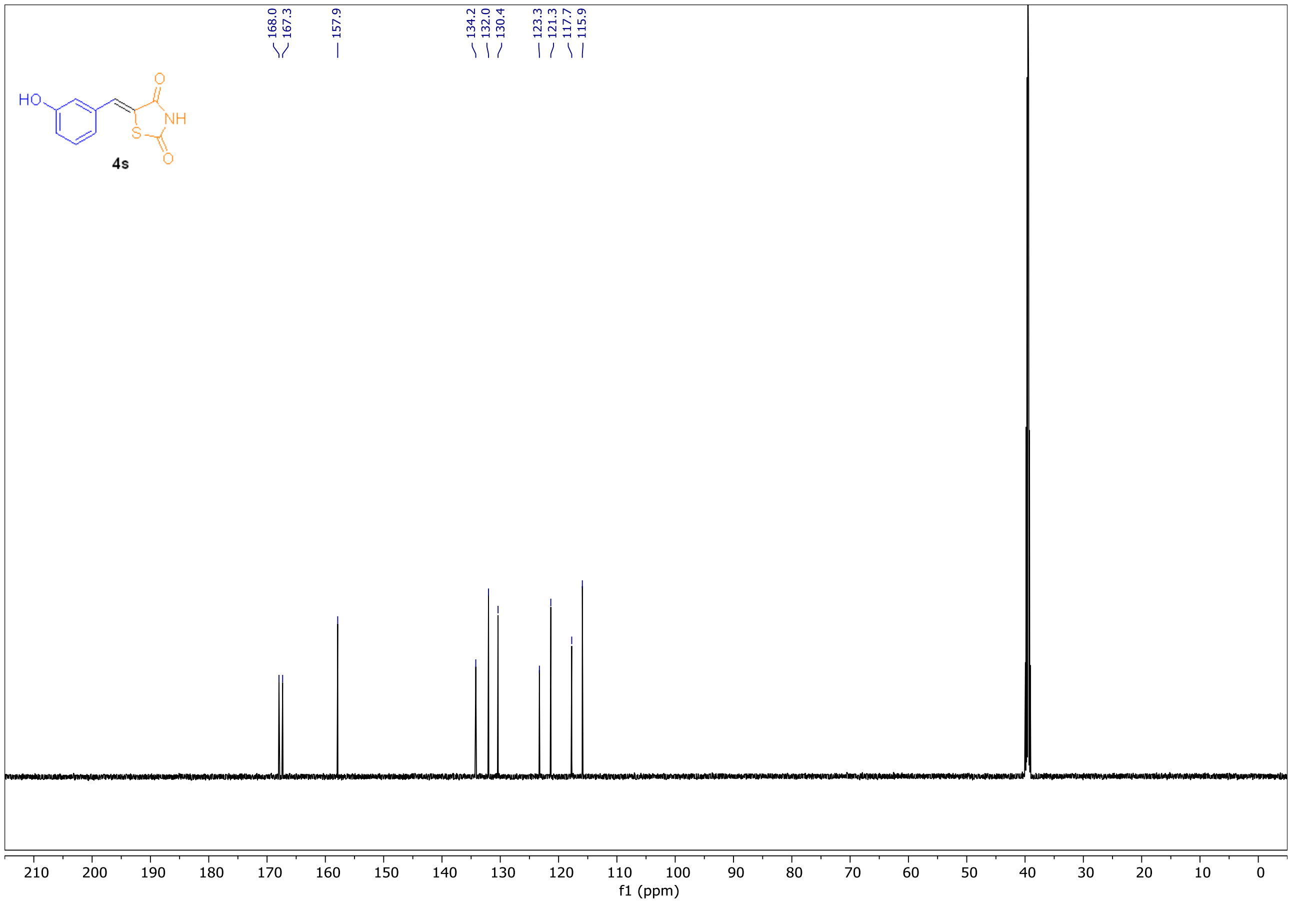

Figure S184. ^{13}C NMR spectrum (151 MHz, DMSO-d_6) of compound **4s**.

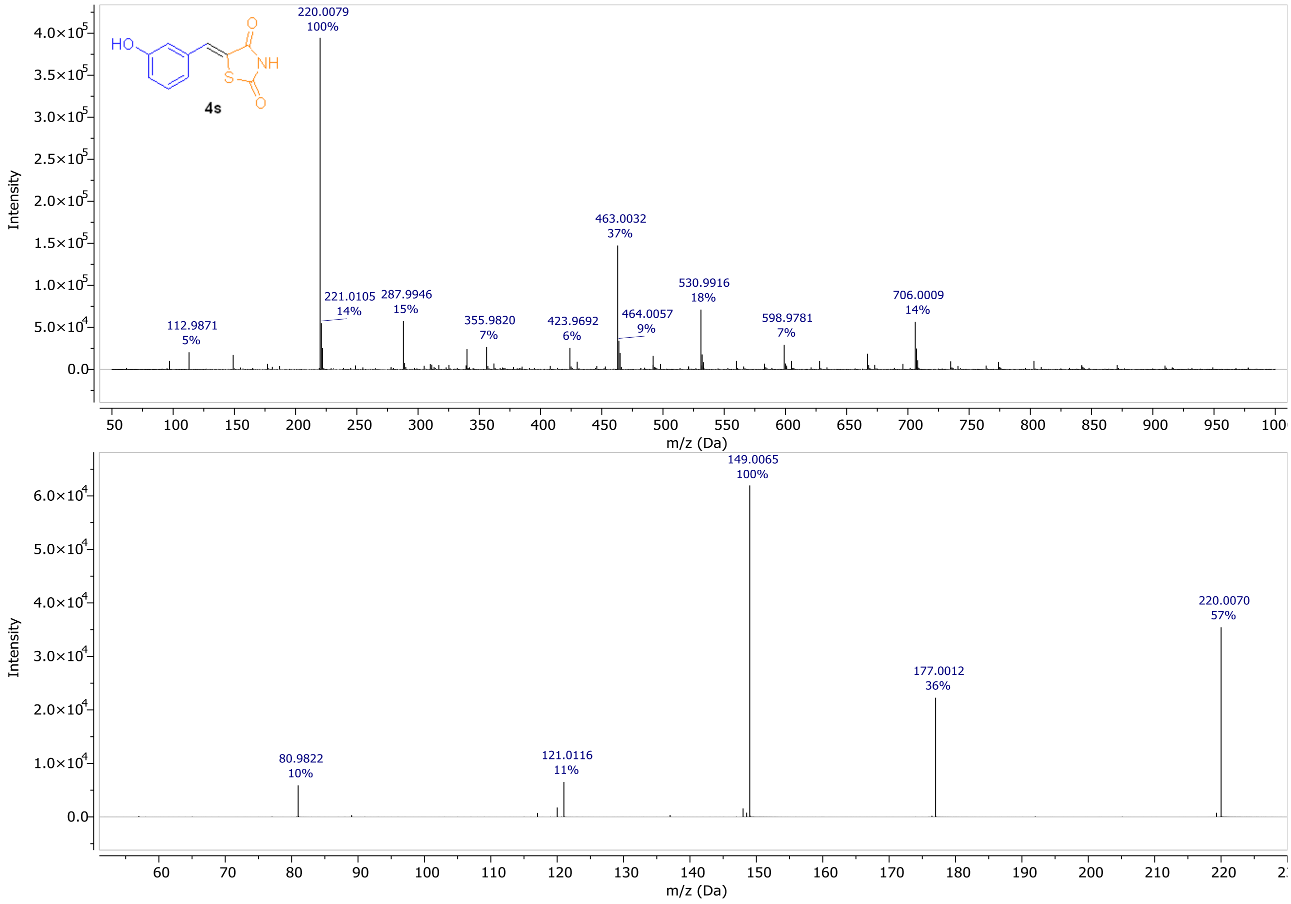

Figure S185. HRMS (ESI-QTOF) of compound **4s** and HRMS/MS for [M−H]⁻.

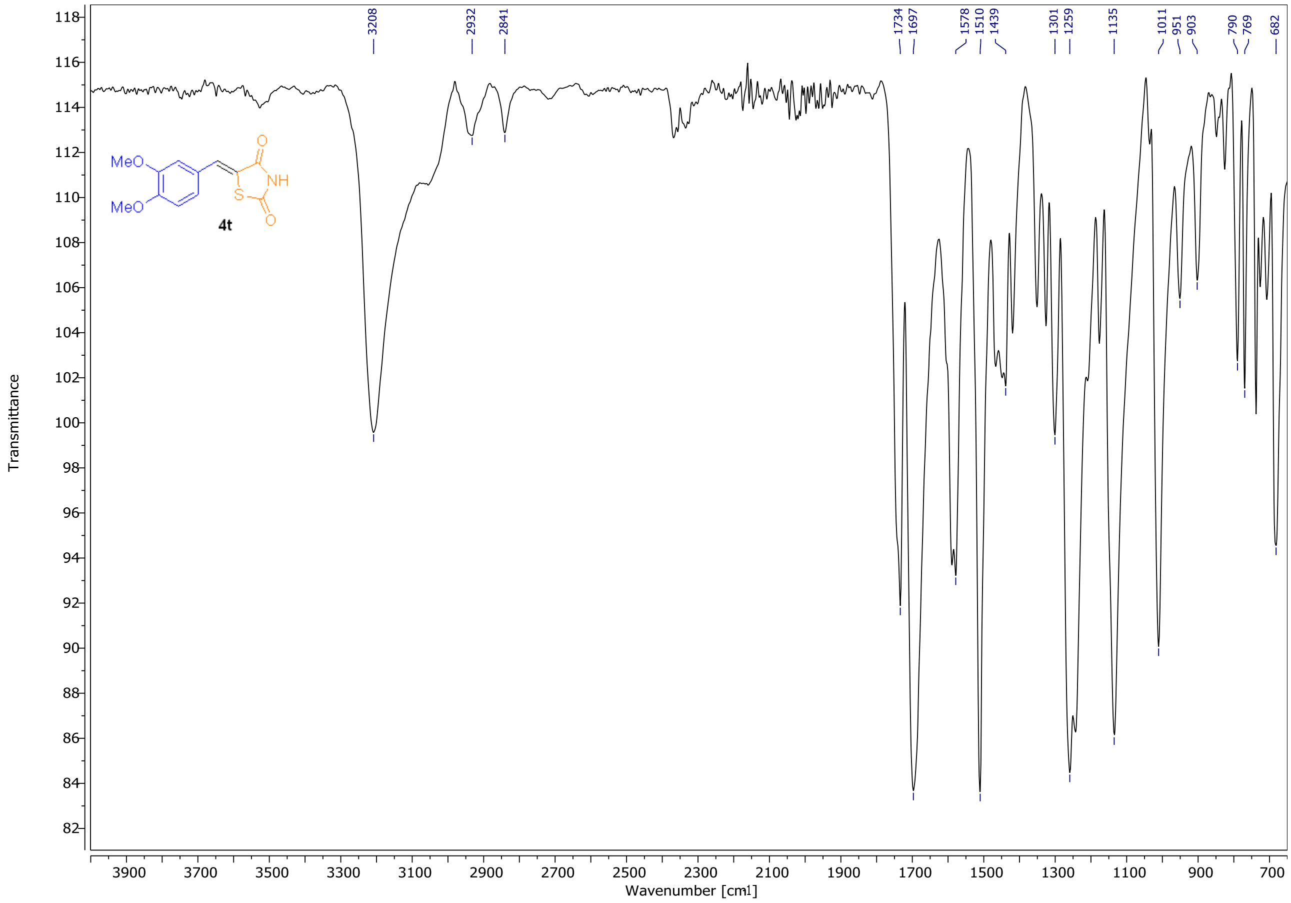

Figure S186. FTIR (ATR) of compound **4t**.

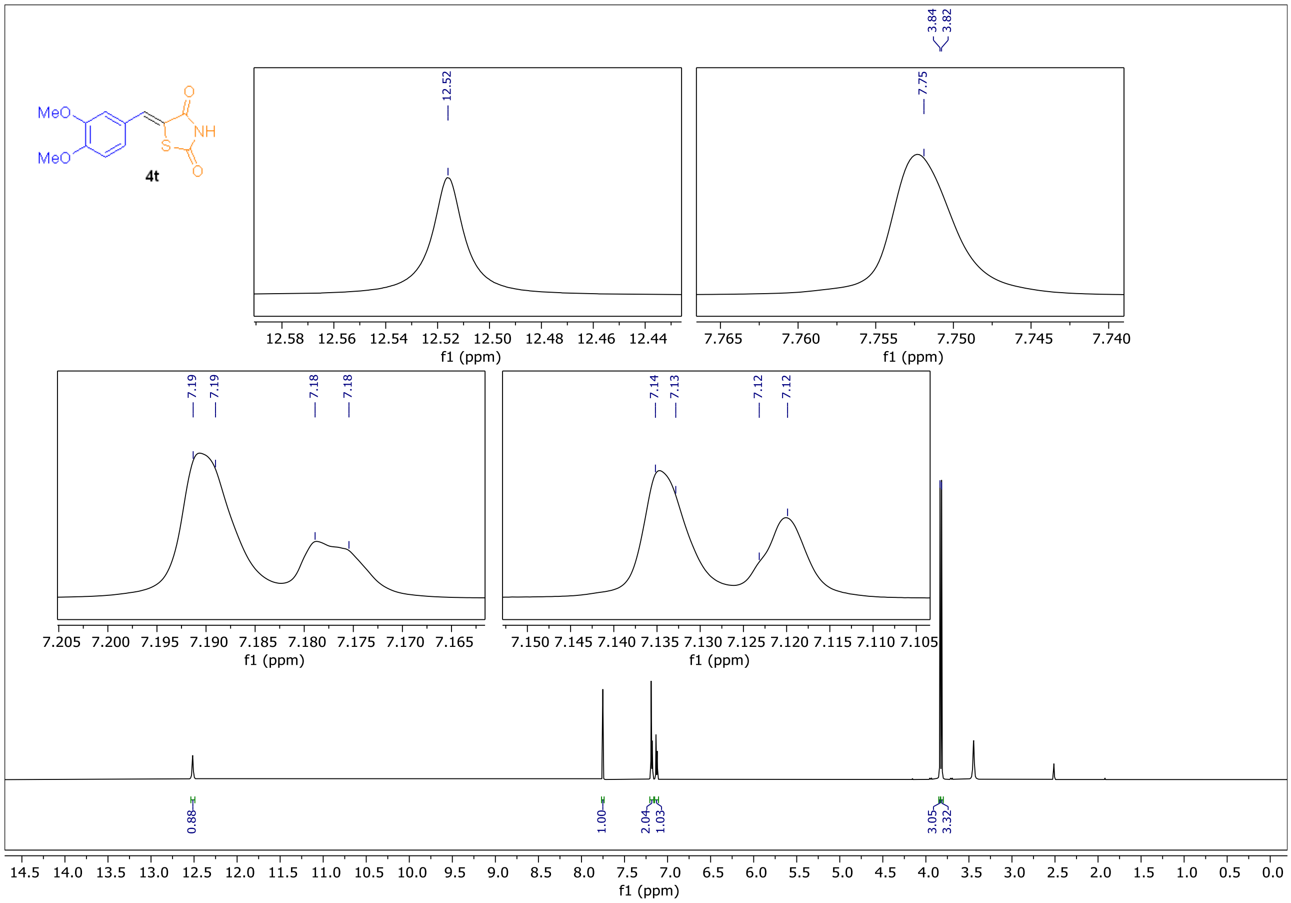

Figure S187. ^{1}H NMR spectrum (600 MHz, DMSO-d_6) of compound **4t**.

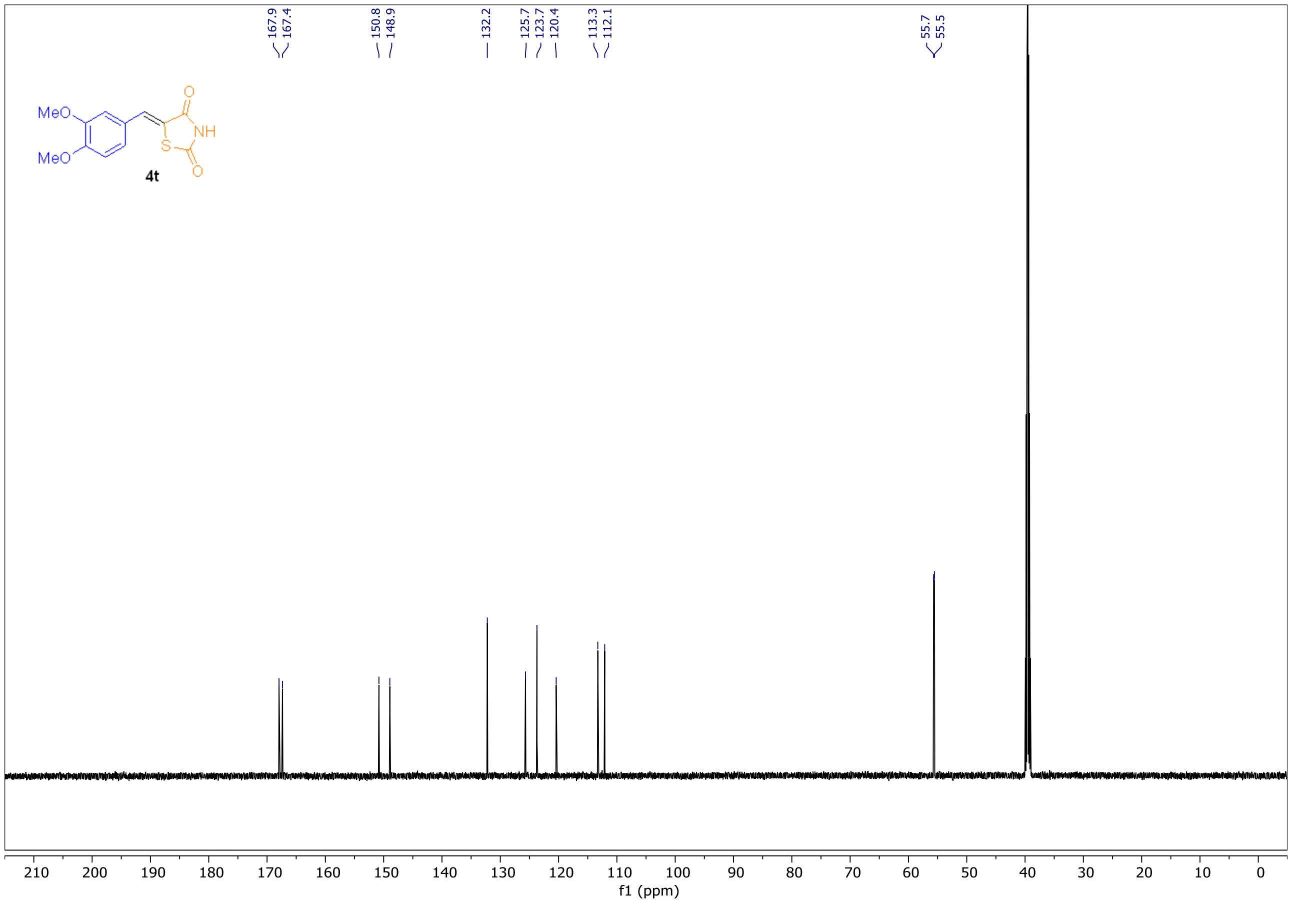

Figure S188. ^{13}C NMR spectrum (151 MHz, DMSO-d_6) of compound **4t**.

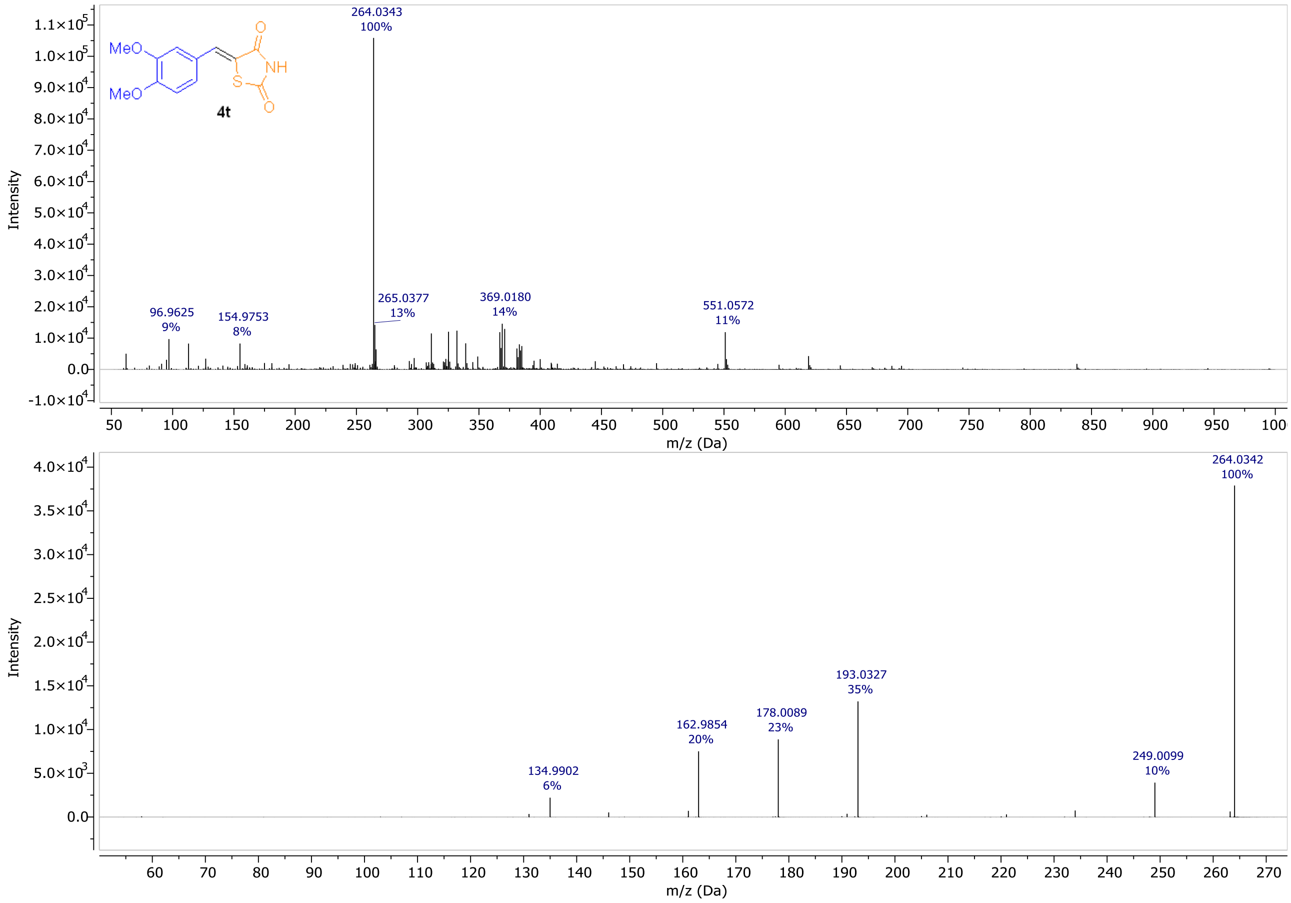

Figure S189. HRMS (ESI-QTOF) of compound **4t** and HRMS/MS for [M−H]⁻.

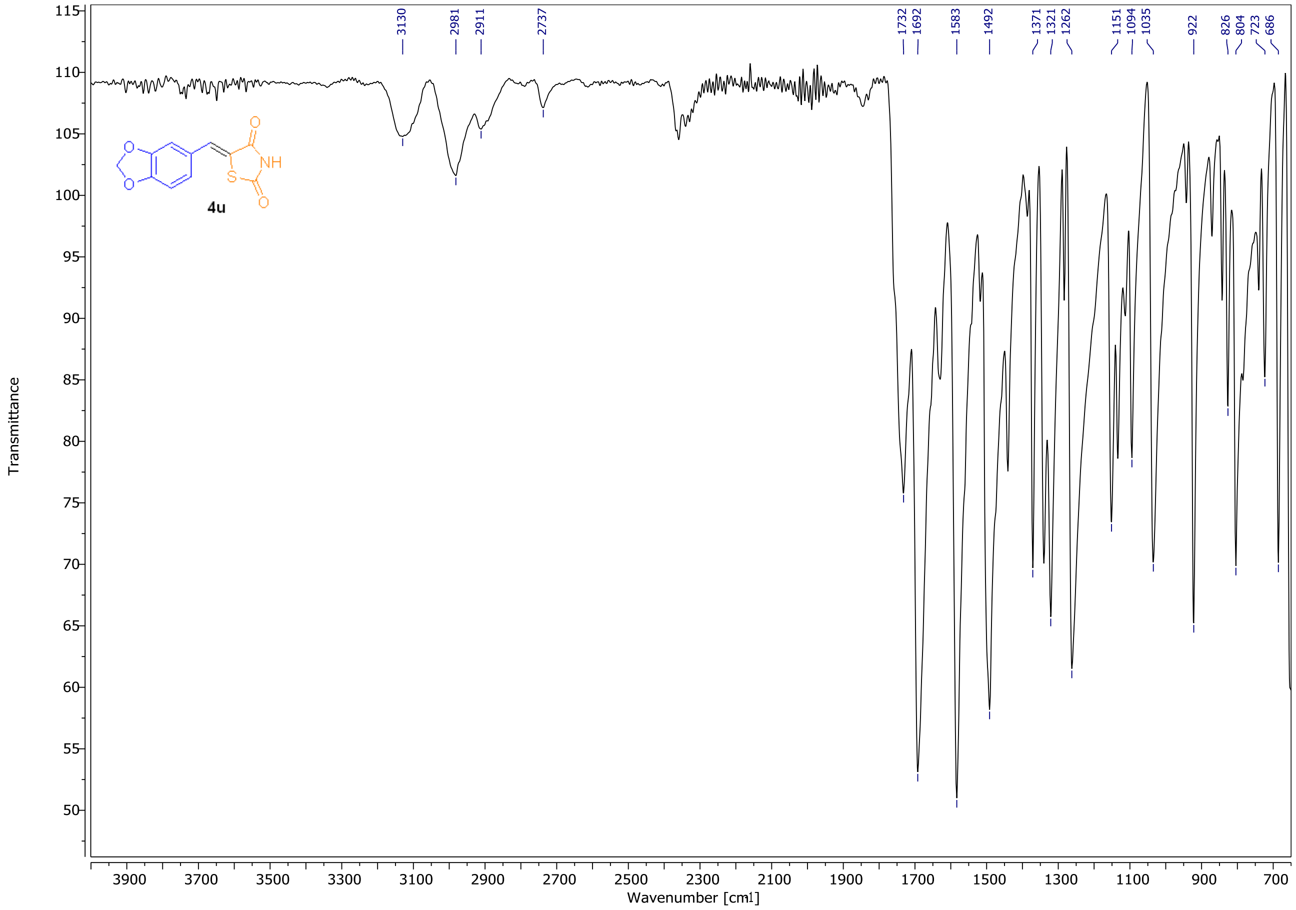

Figure S190. FTIR (ATR) of compound **4u**.

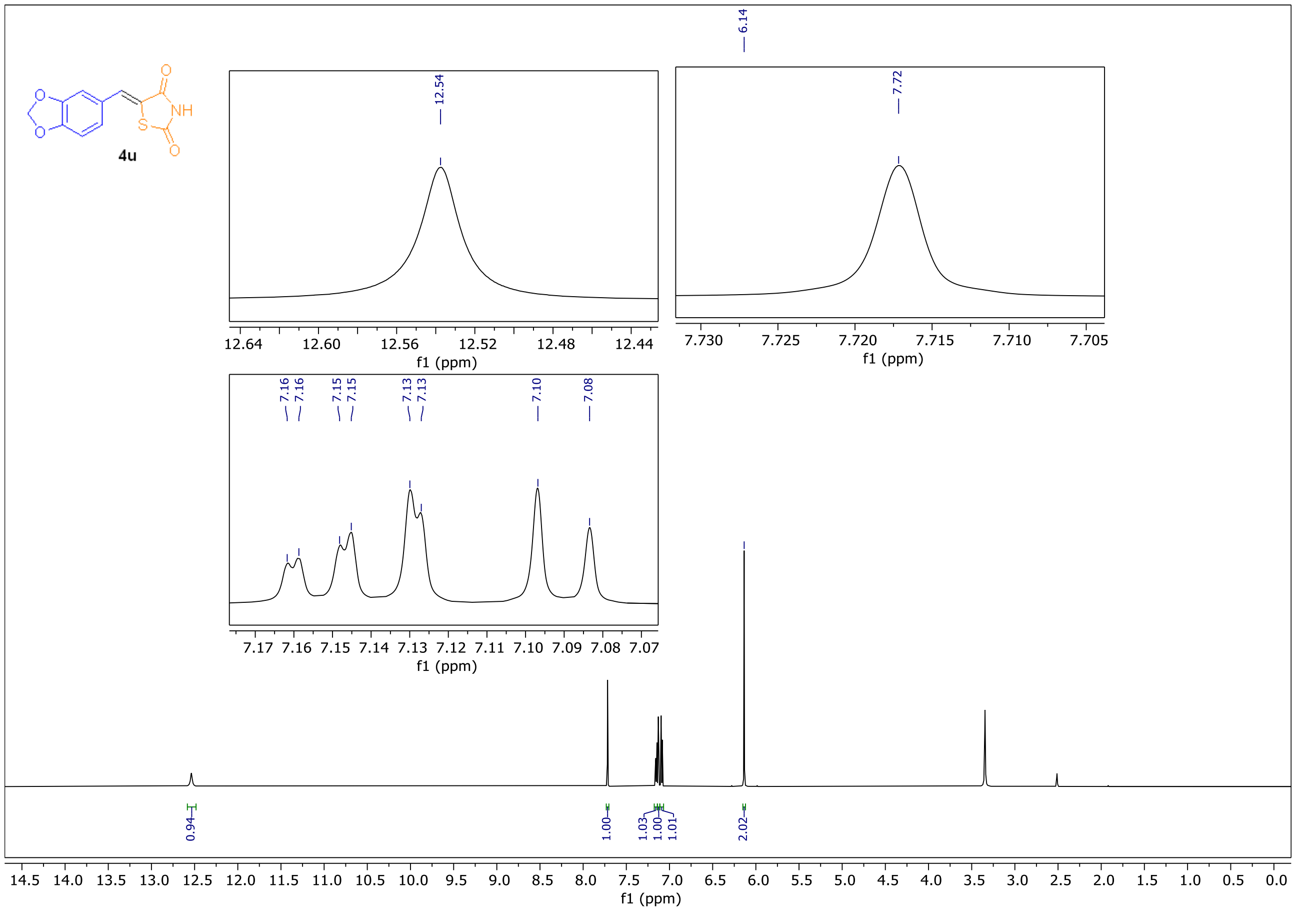

Figure S191. ^{1}H NMR spectrum (600 MHz, DMSO-d_6) of compound **4u**.

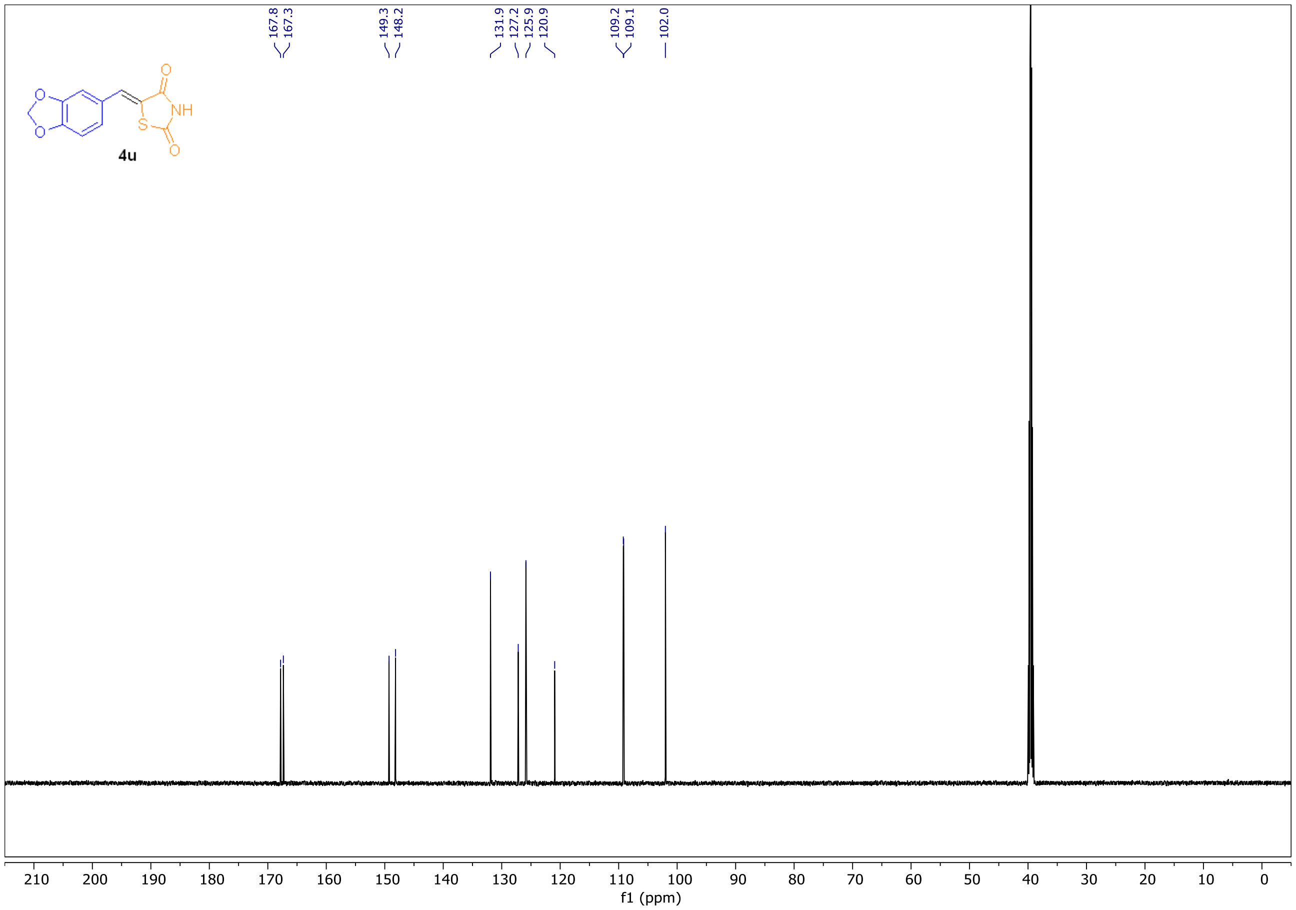

Figure S192. ^{13}C NMR spectrum (151 MHz, DMSO-d_6) of compound **4u**.

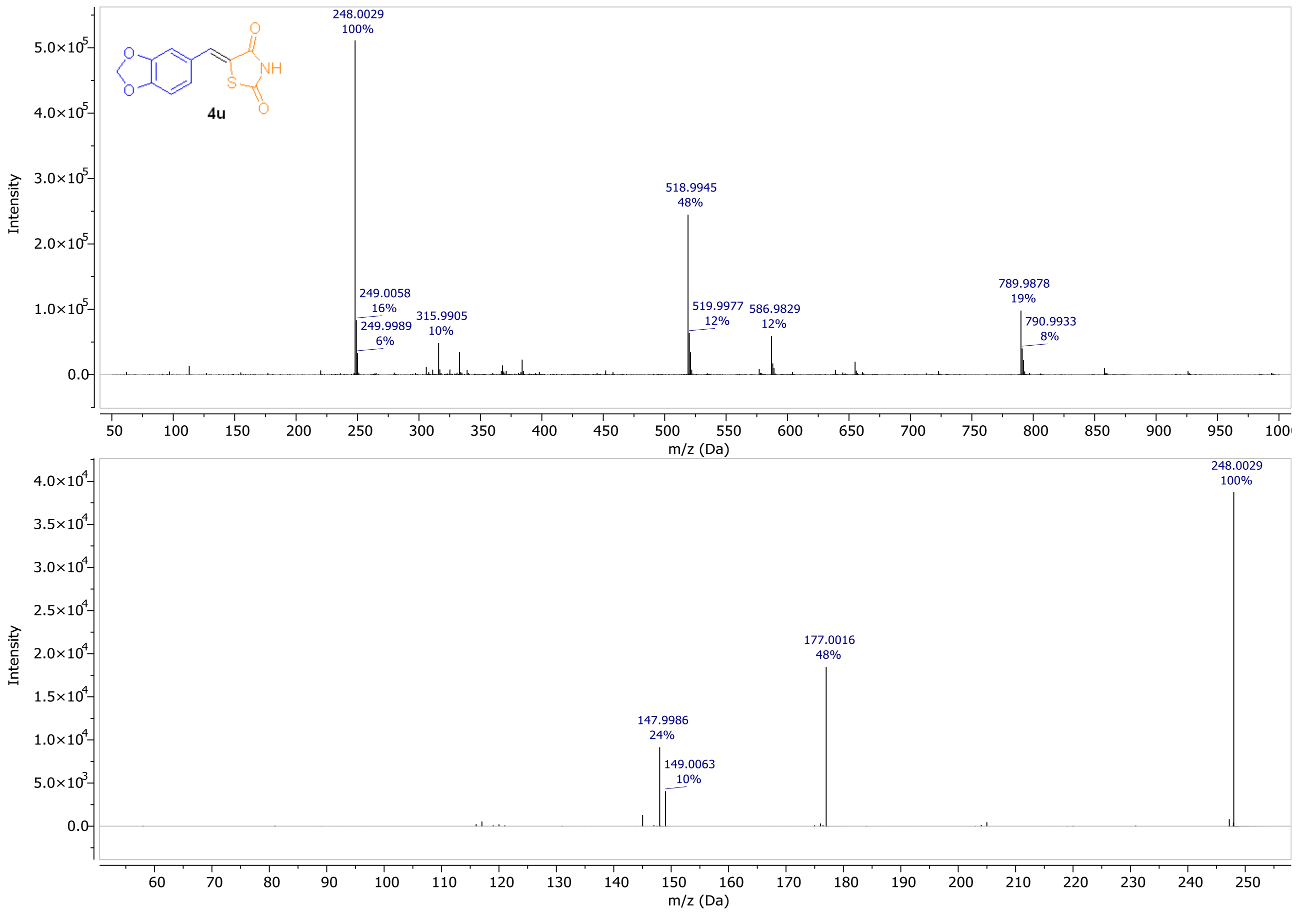

Figure S193. HRMS (ESI-QTOF) of compound **4u** and HRMS/MS for [M−H]$^-$.

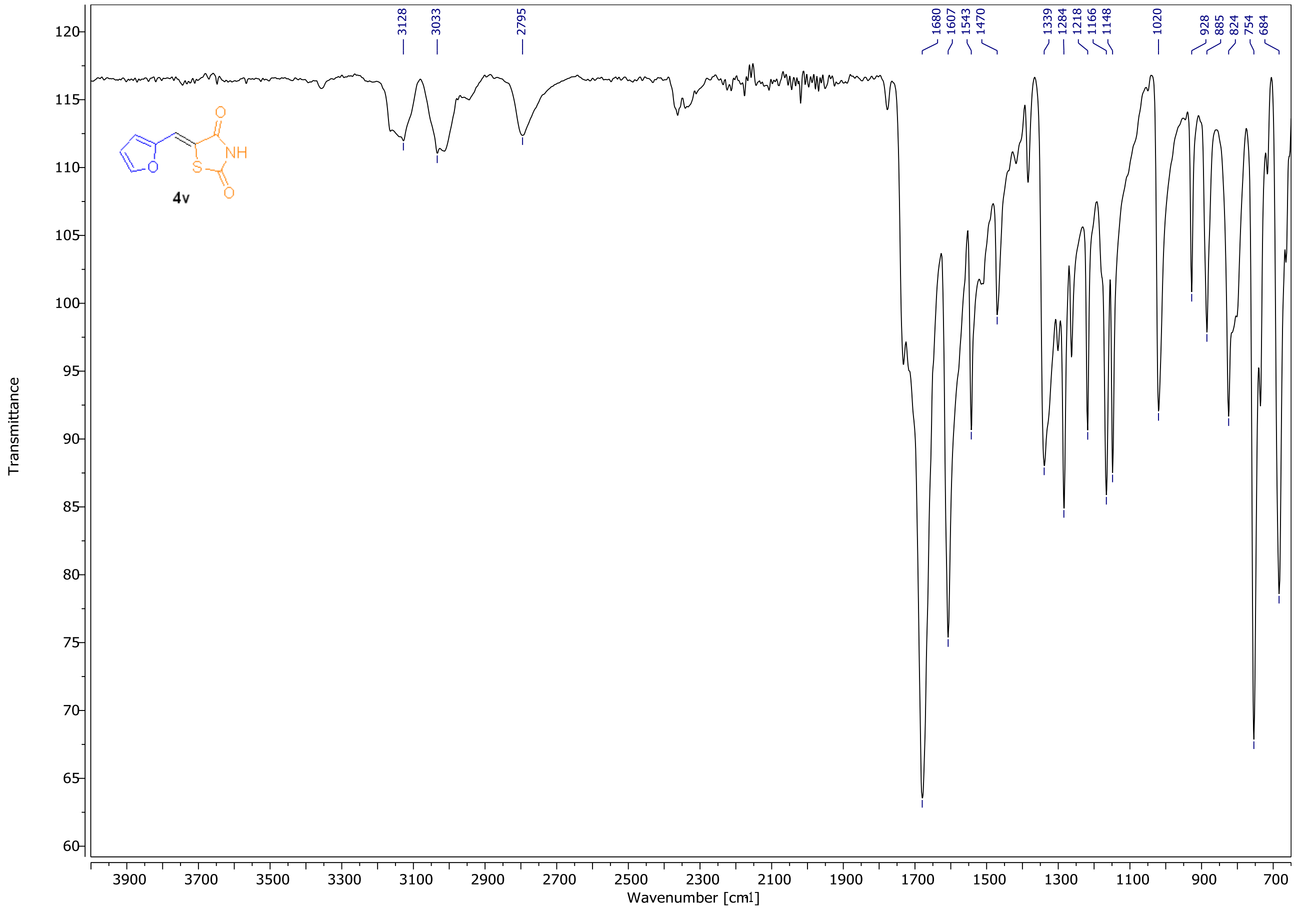

Figure S194. FTIR (ATR) of compound **4v**.

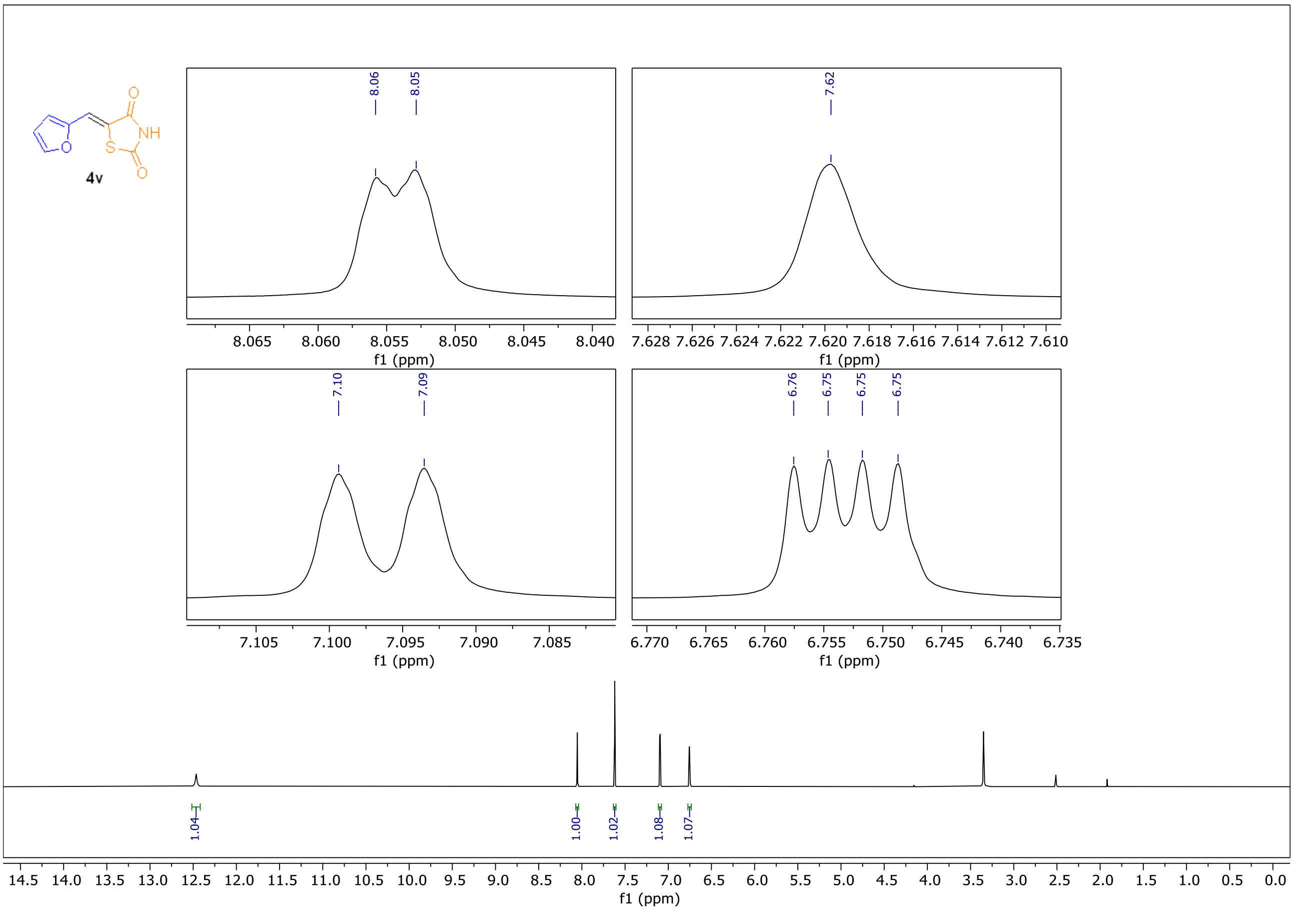

Figure S195. ^{1}H NMR spectrum (600 MHz, DMSO-d_6) of compound **4v**.

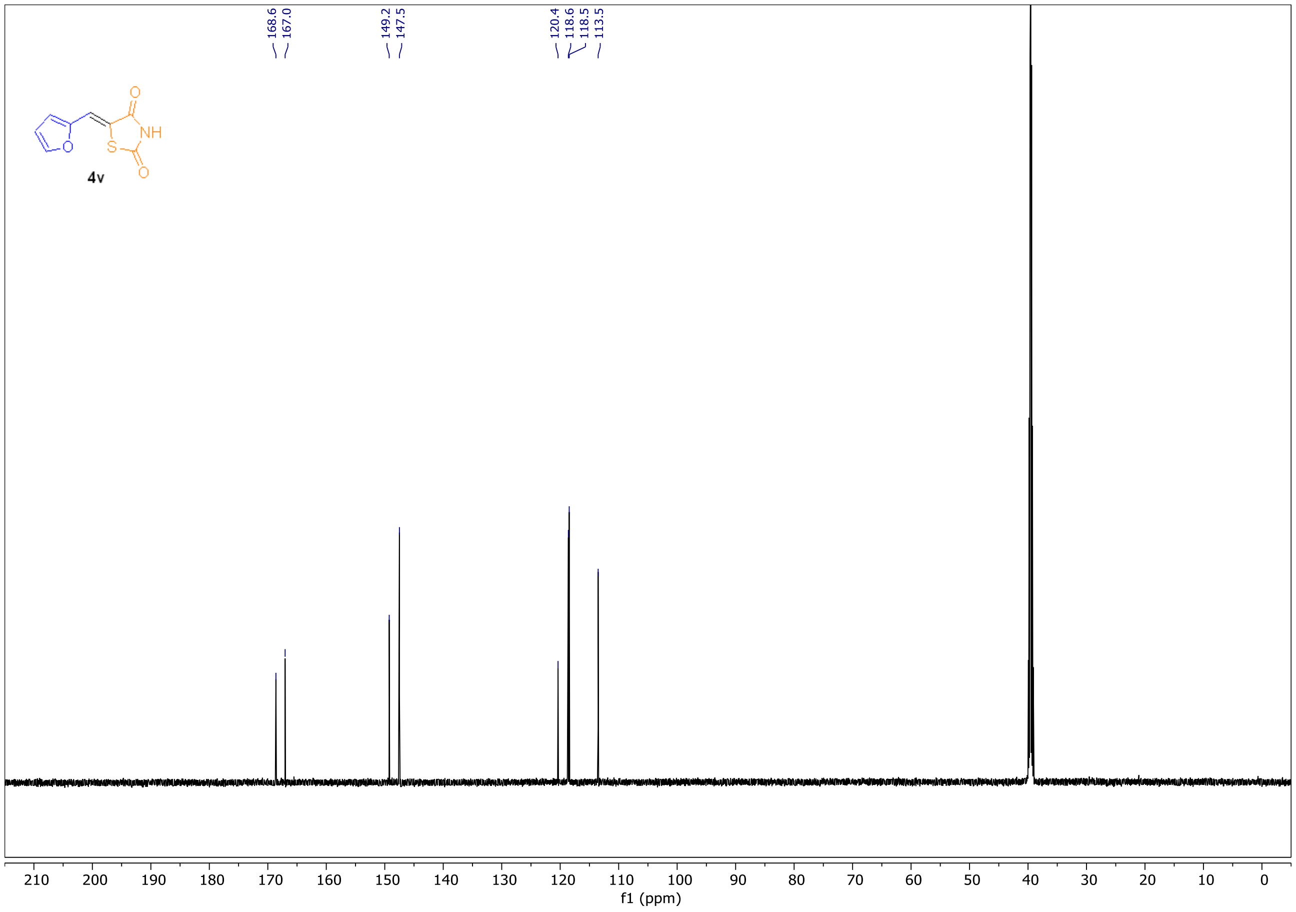

Figure S196. ^{13}C NMR spectrum (151 MHz, DMSO-d_6) of compound **4v**.

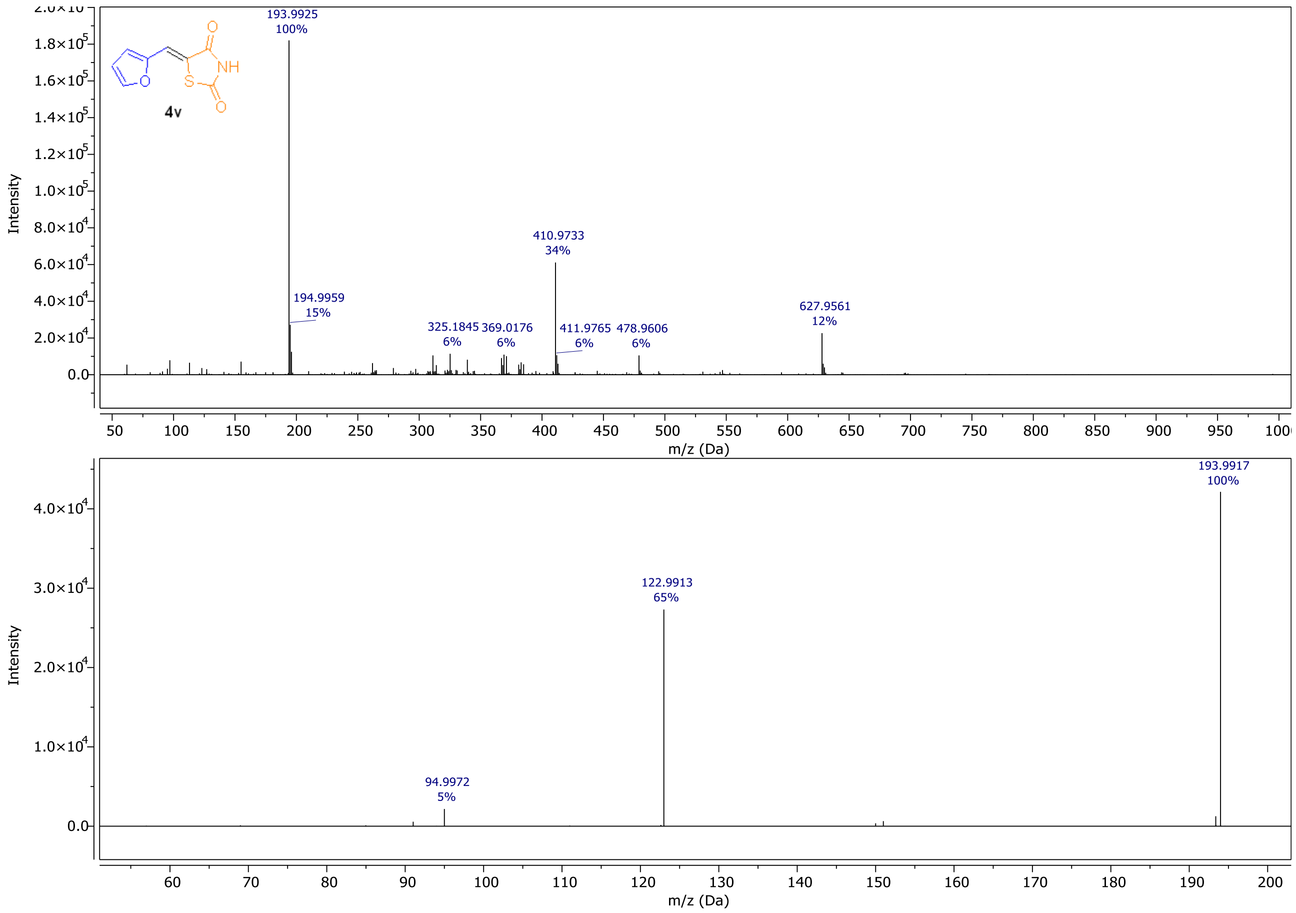

Figure S197. HRMS (ESI-QTOF) of compound **4v** and HRMS/MS for $[M-H]^-$.

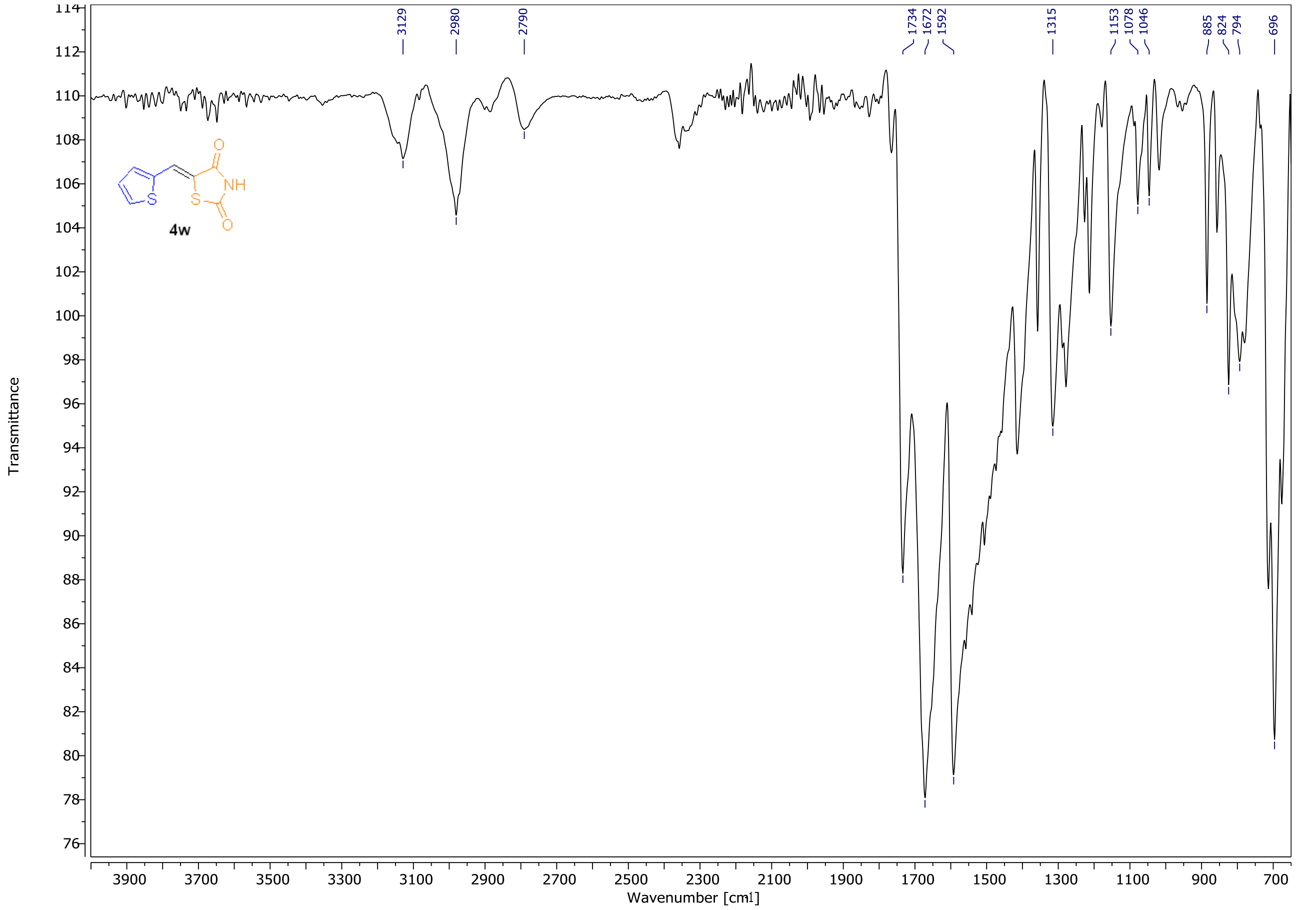

Figure S198. FTIR (ATR) of compound **4w**.

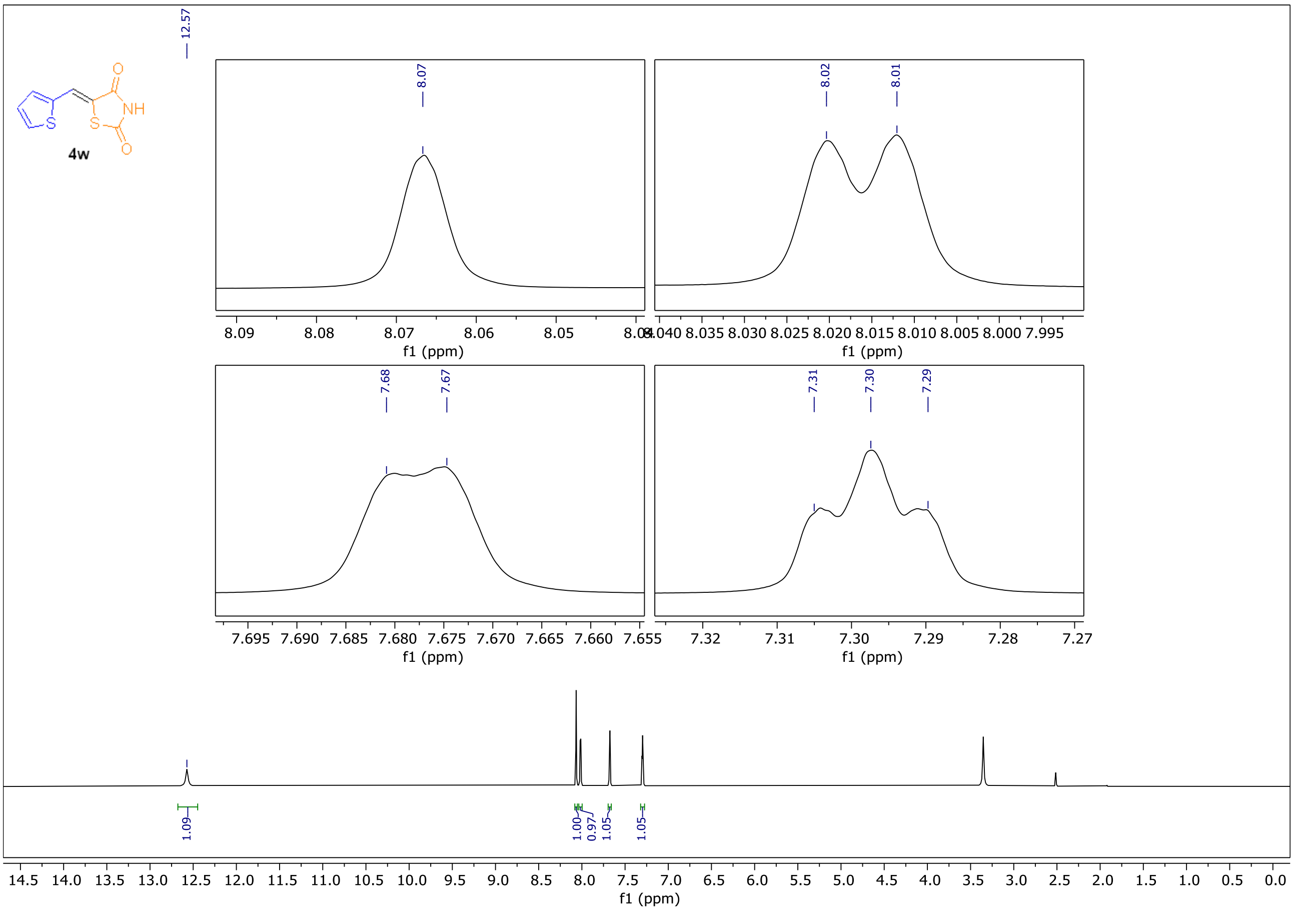

Figure S199. ^{1}H NMR spectrum (600 MHz, DMSO-d_6) of compound **4w**.

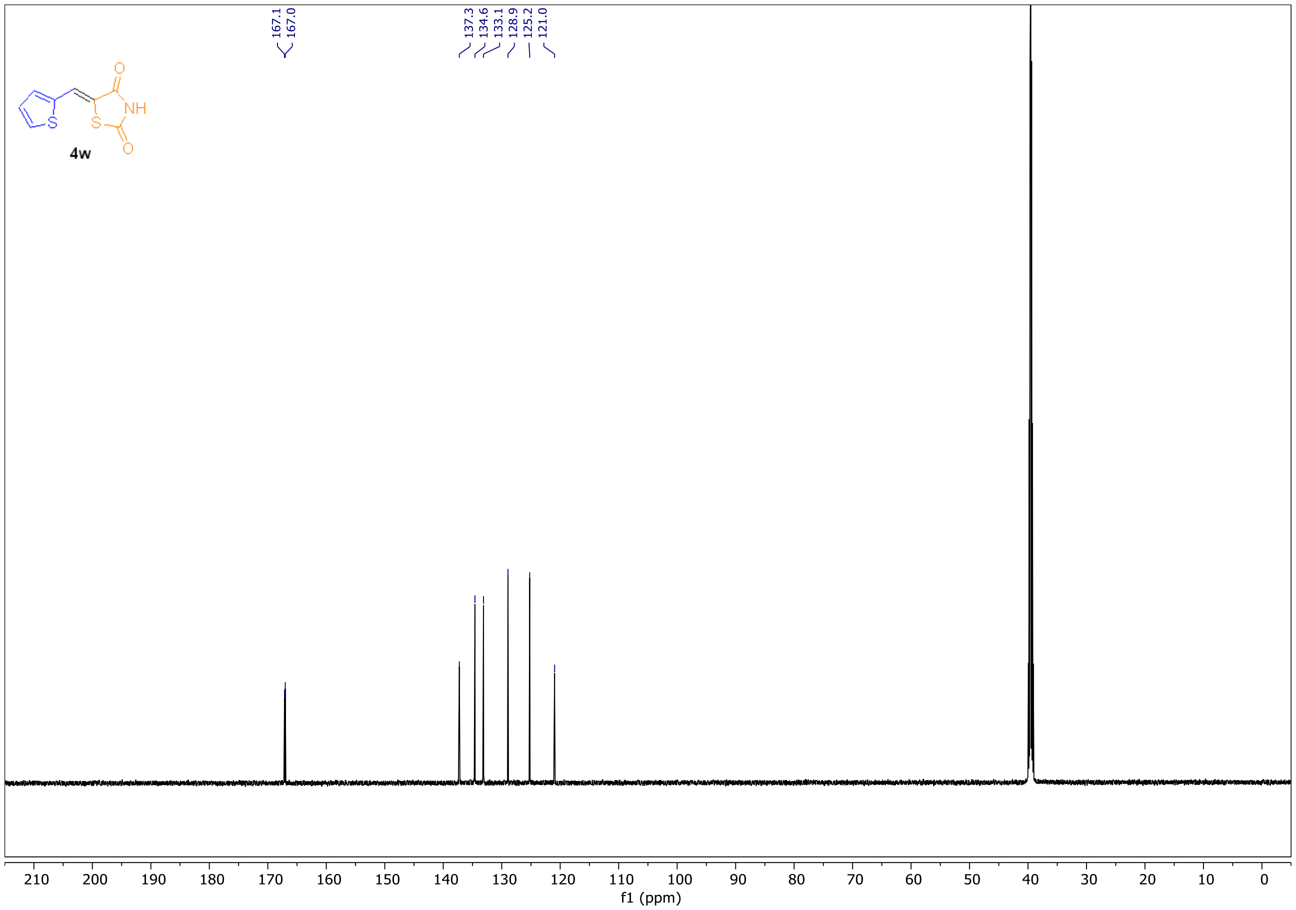

Figure S200. ^{13}C NMR spectrum (151 MHz, DMSO-d_6) of compound **4w**.

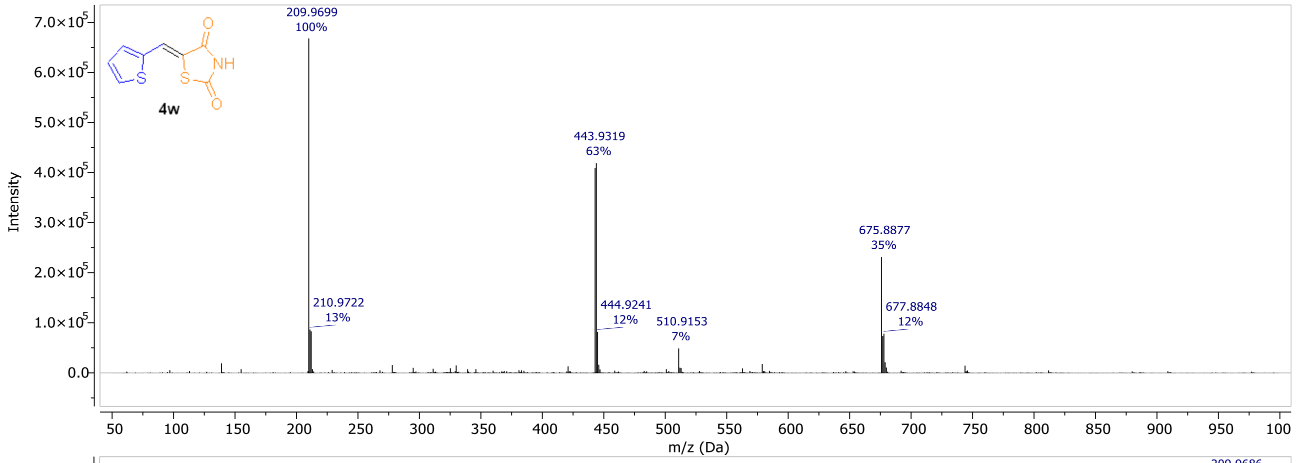

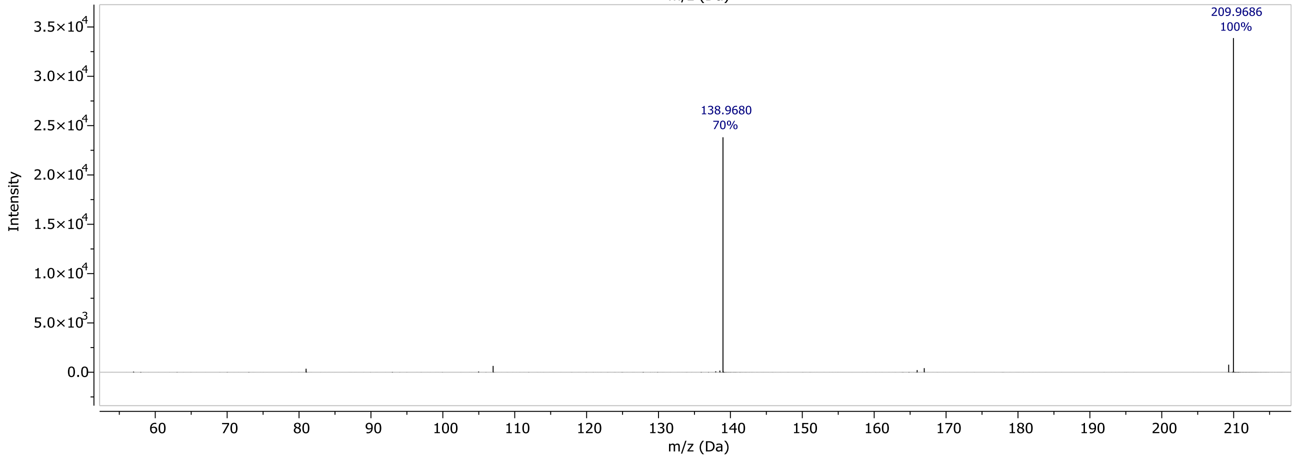

Figure S201. HRMS (ESI-QTOF) of compound **4w** and HRMS/MS for [M−H]⁻.

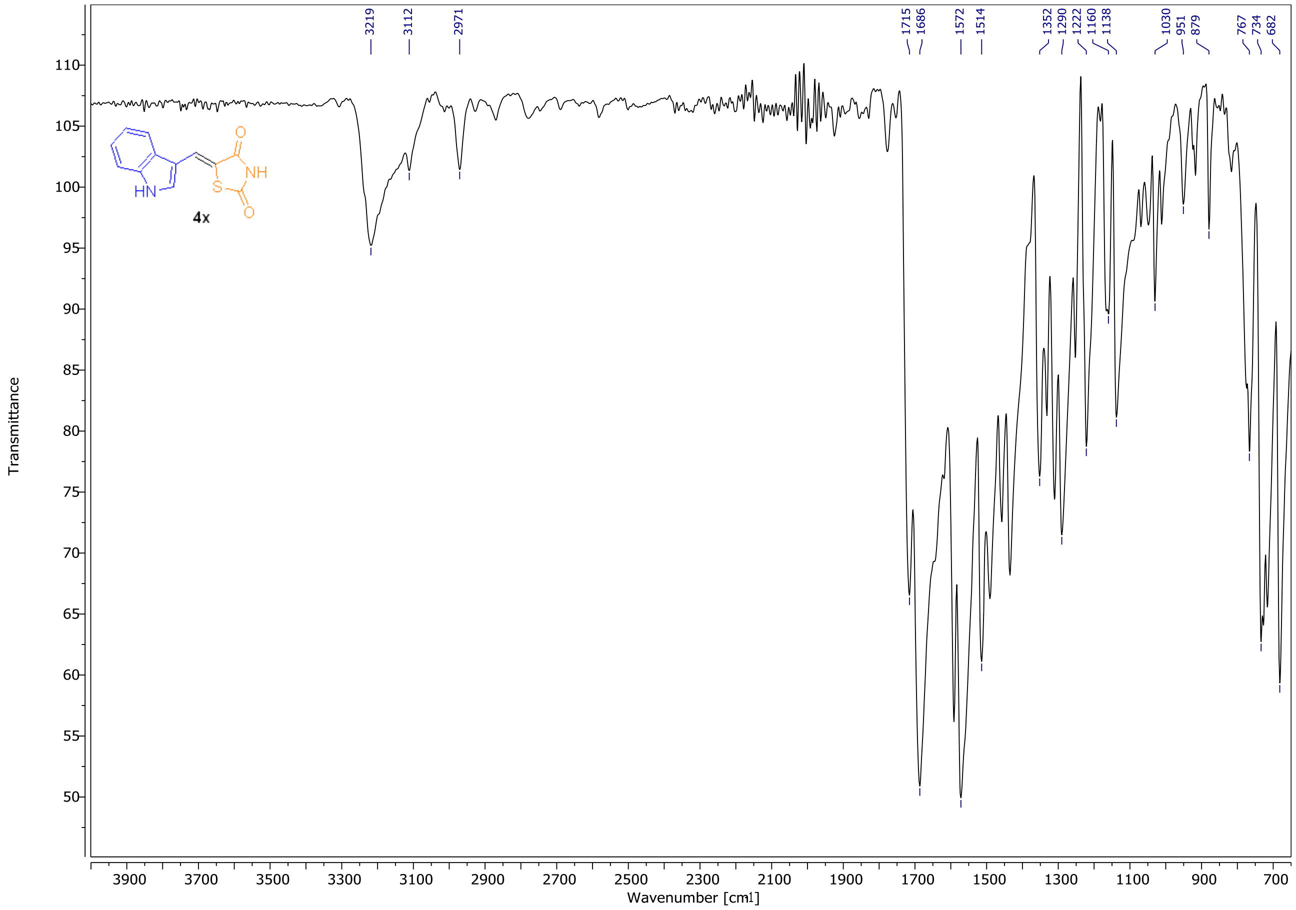

Figure S202. FTIR (ATR) of compound **4x**.

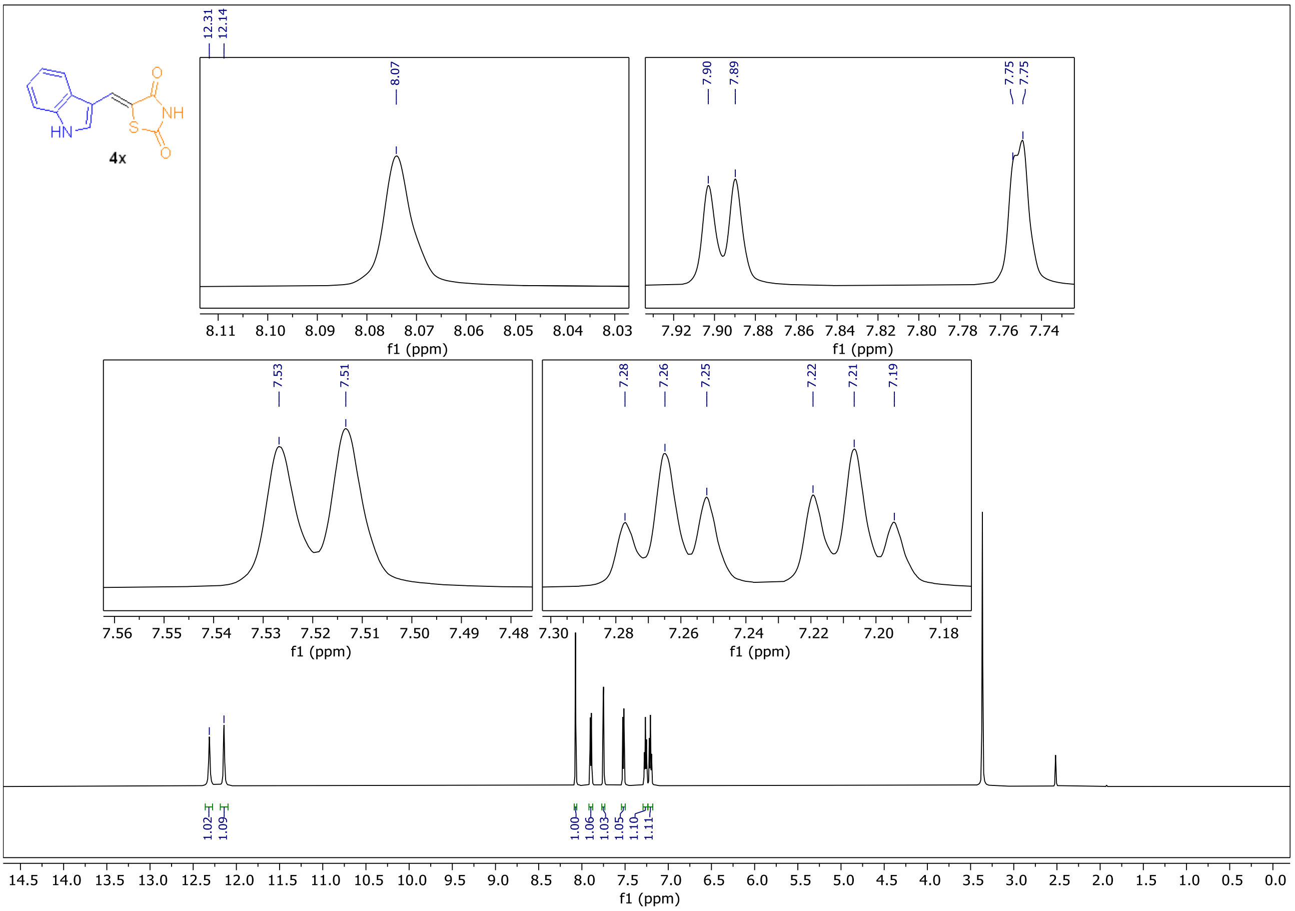

Figure S203. ^{1}H NMR spectrum (600 MHz, DMSO-d_6) of compound **4x**.

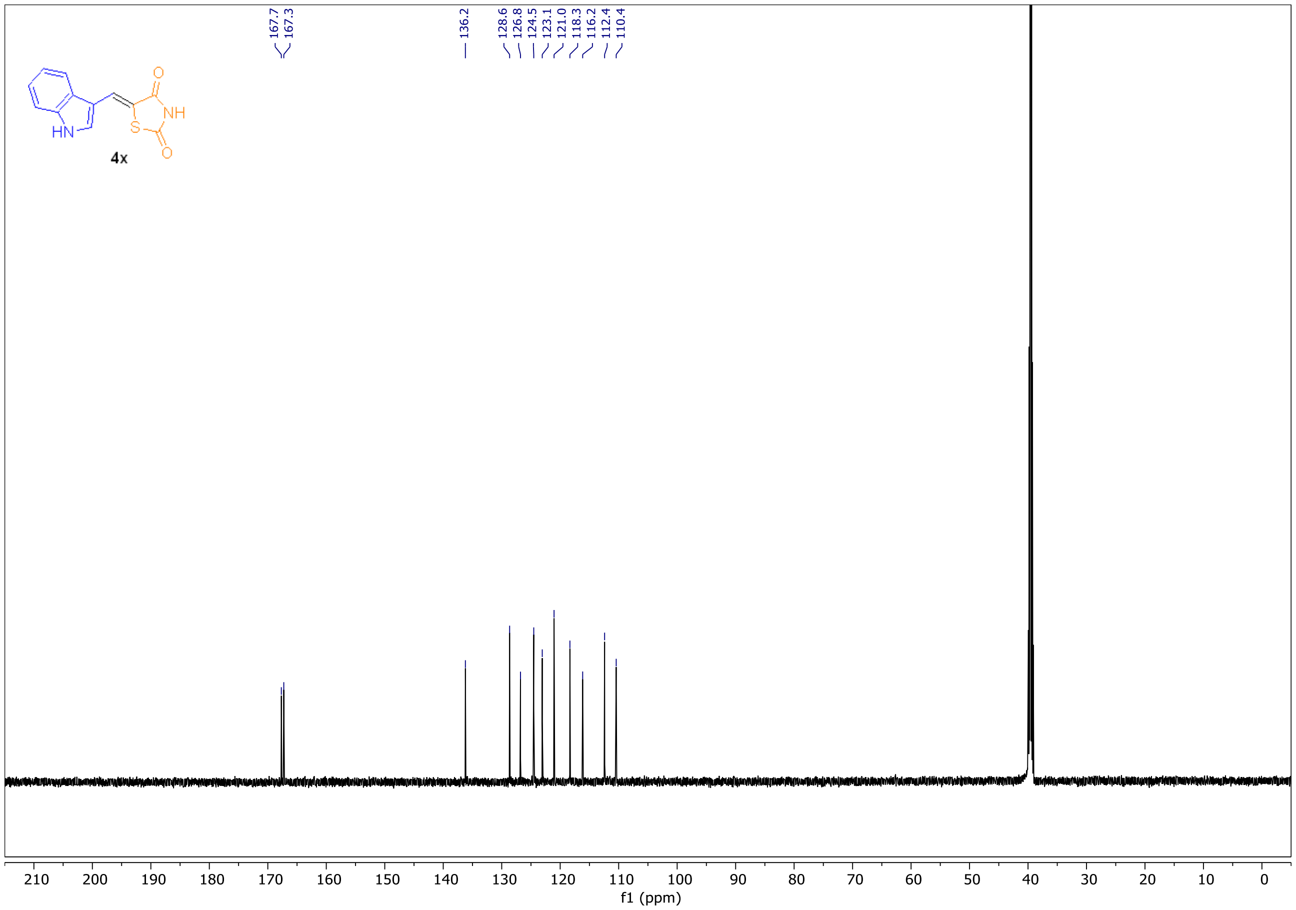

Figure S204. ^{13}C NMR spectrum (151 MHz, DMSO-d_6) of compound **4x**.

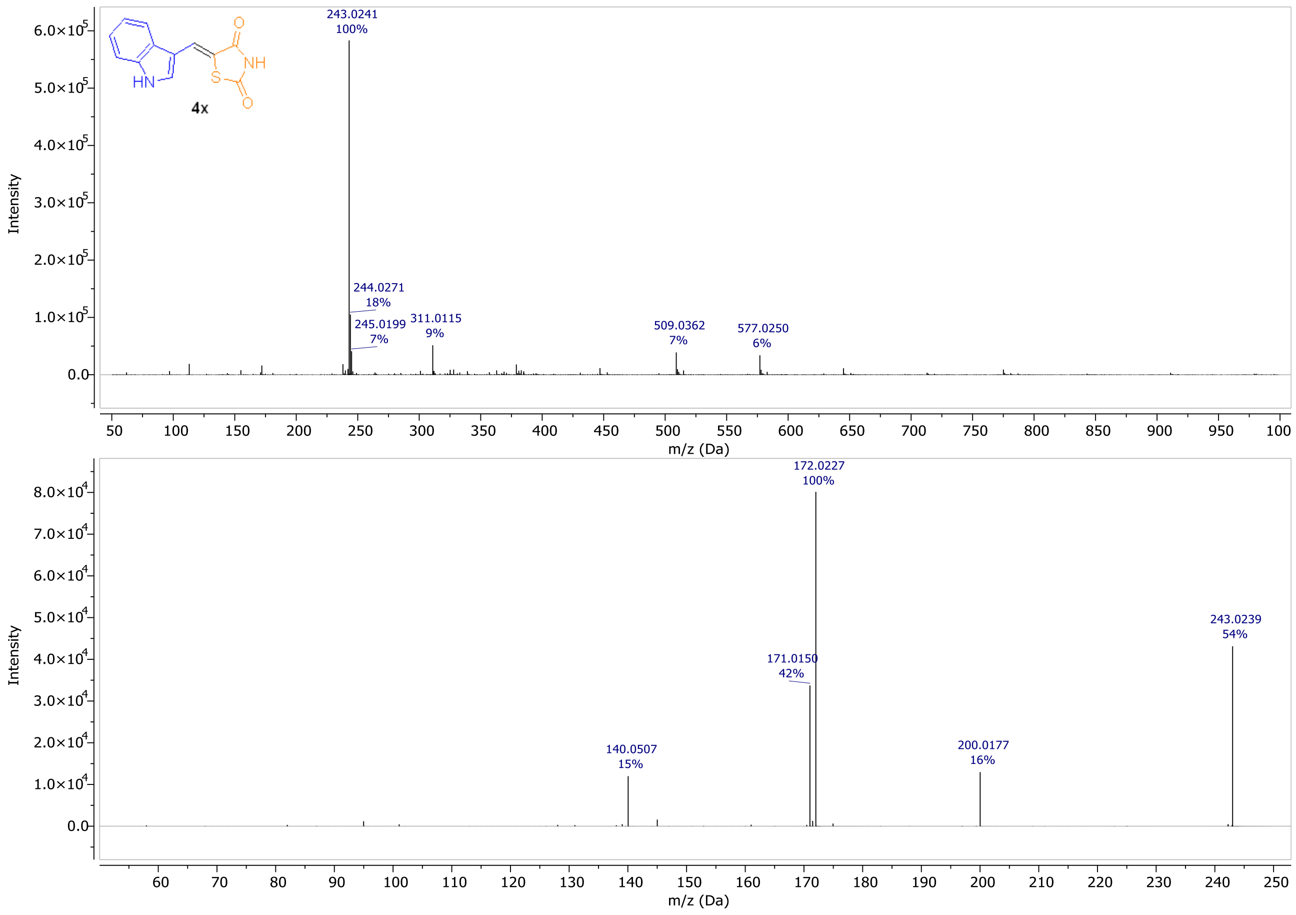

Figure S205. HRMS (ESI-QTOF) of compound **4x** and HRMS/MS for [M−H]$^-$.

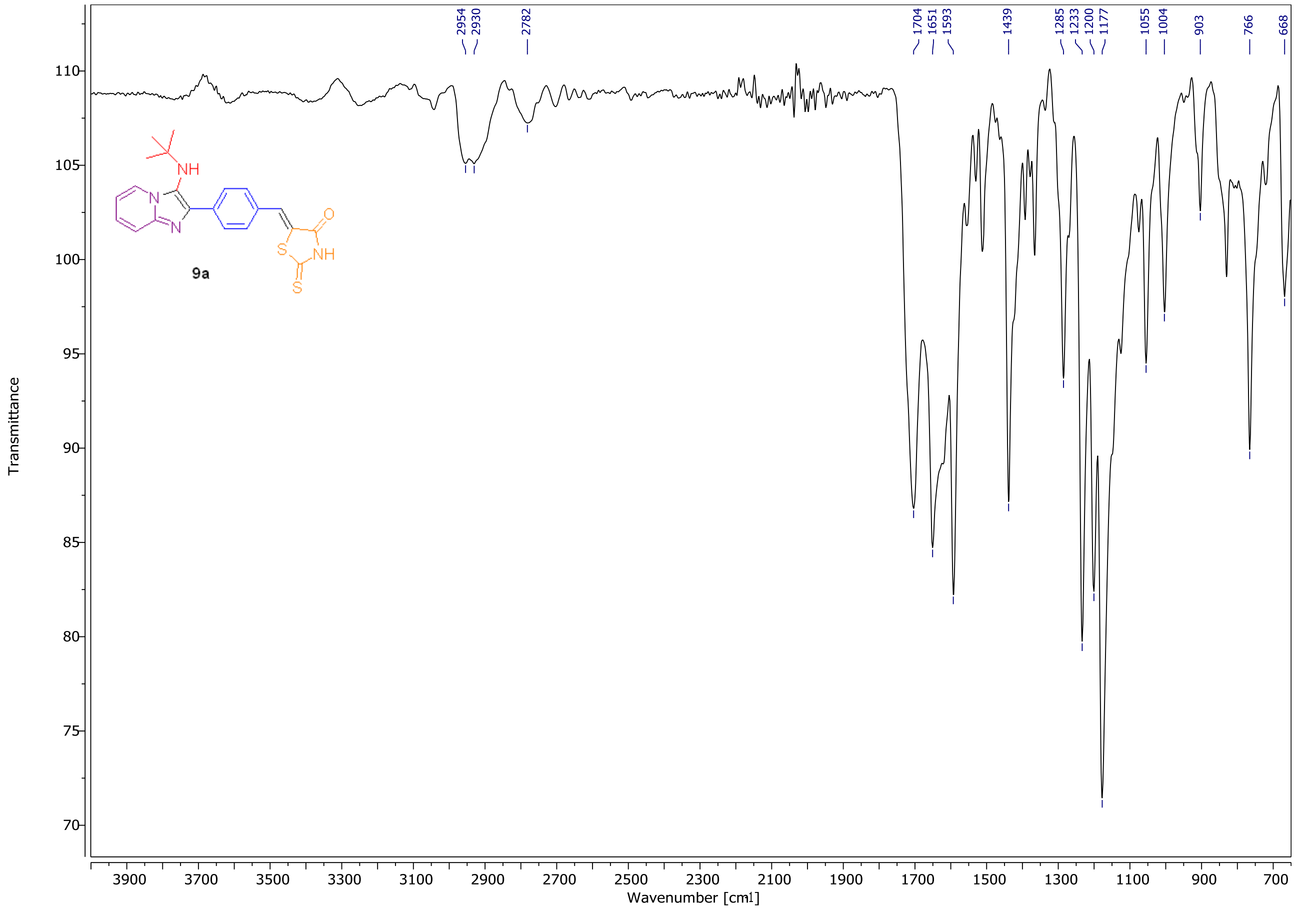

Figure S206. FTIR (ATR) of compound **9a**.

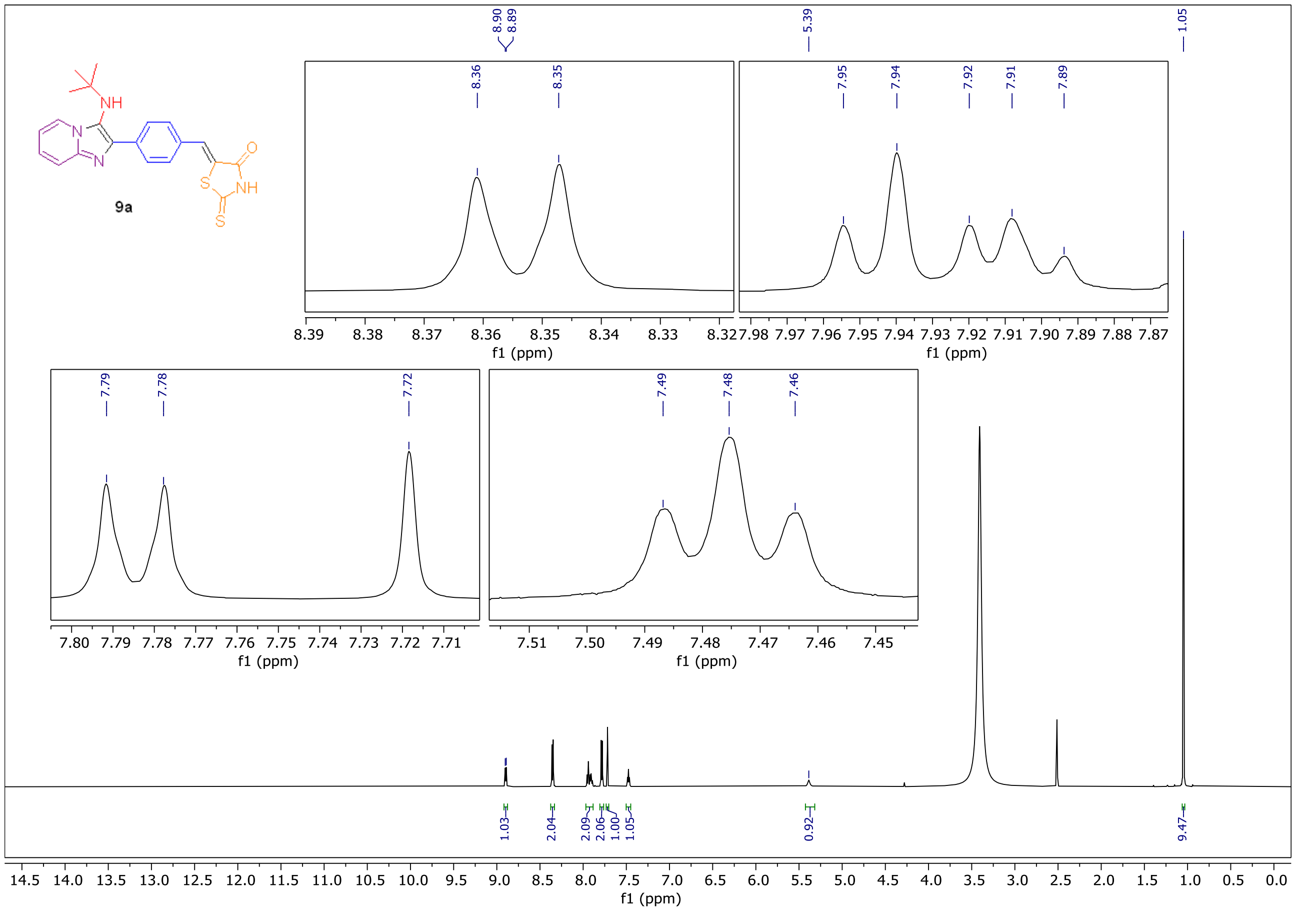

Figure S207. ^{1}H NMR spectrum (600 MHz, DMSO-d_6) of compound **9a**.

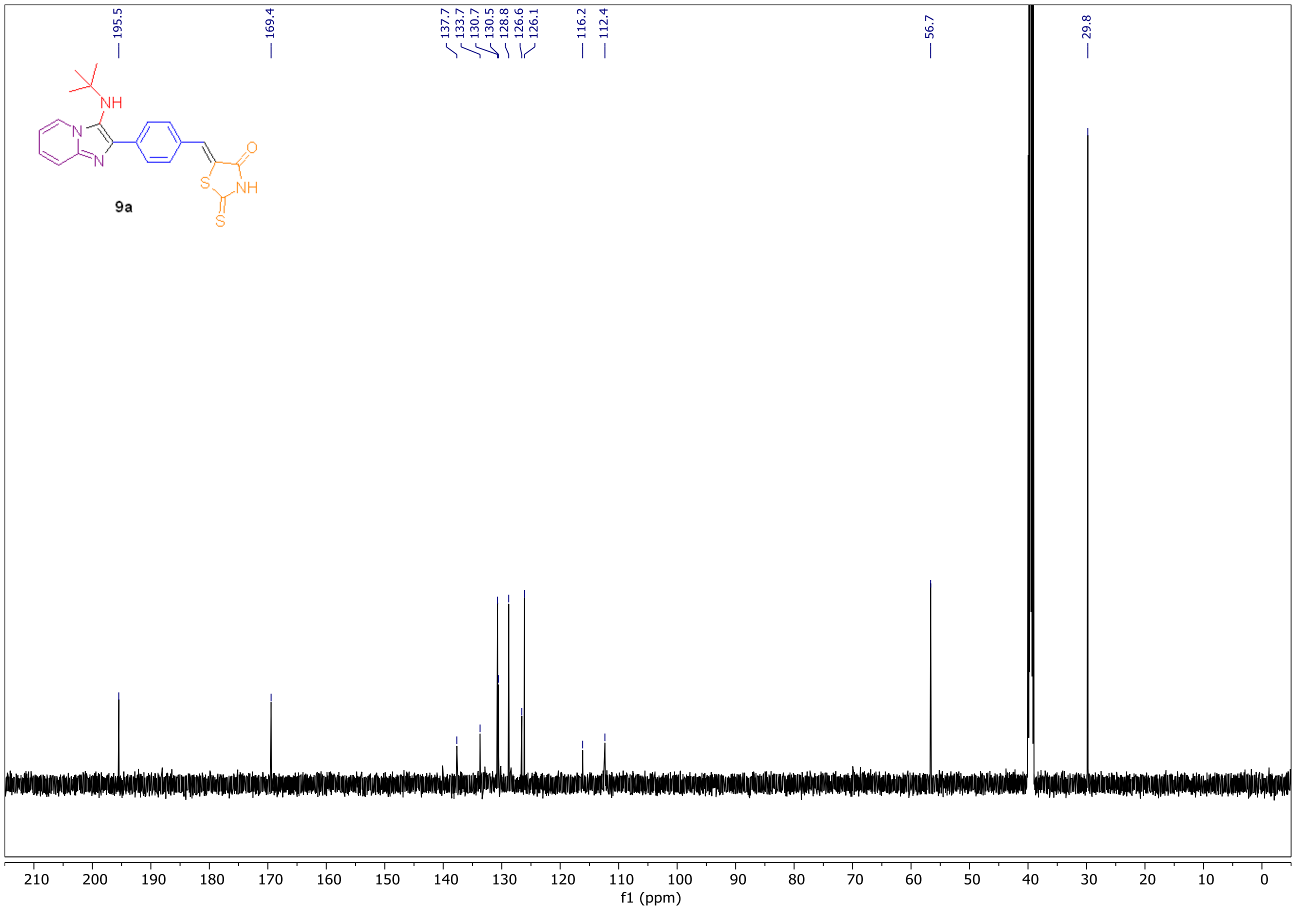

Figure S208. ^{13}C NMR spectrum (151 MHz, DMSO-d_6) of compound **9a**.

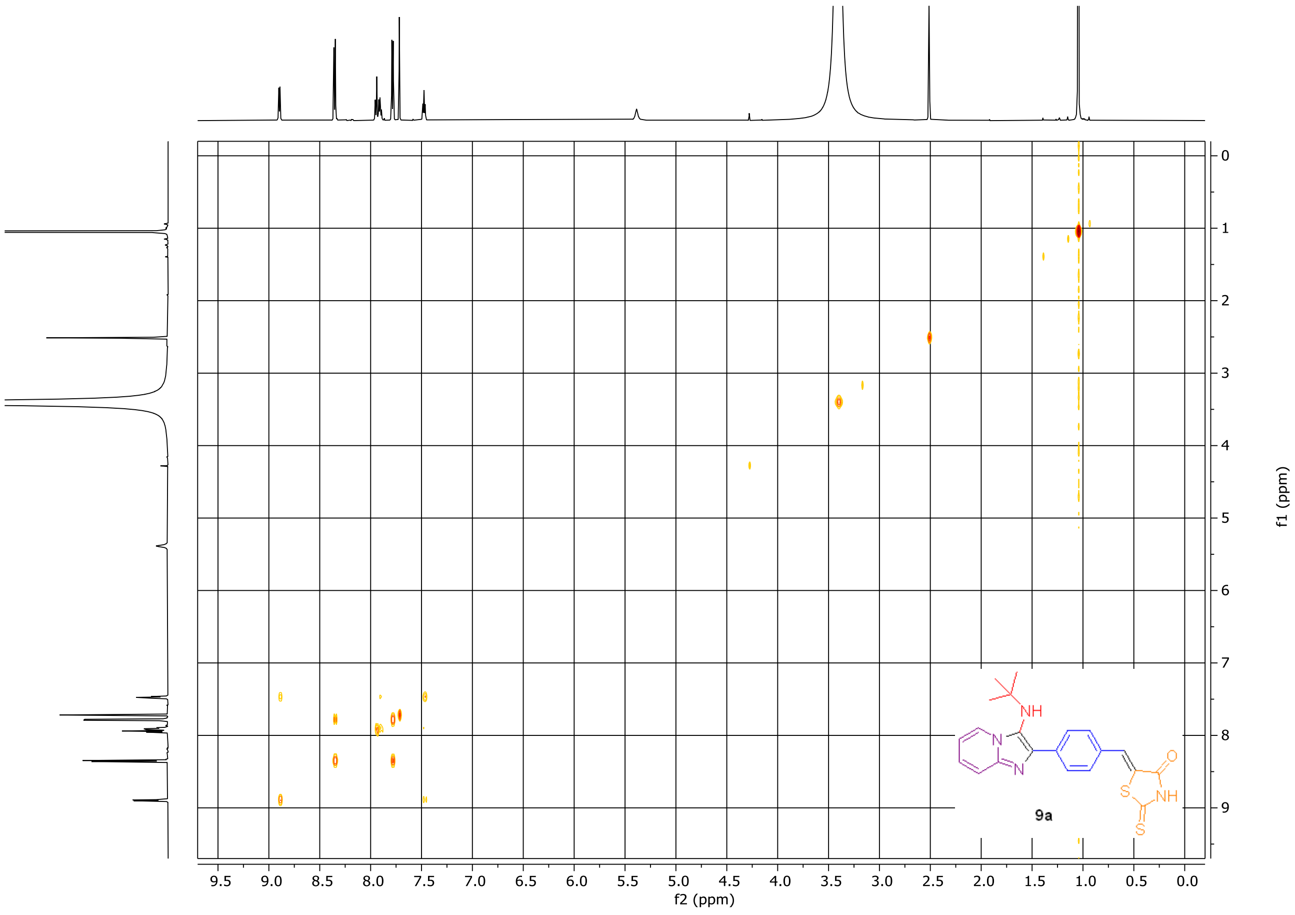

Figure S209. 2D COSY NMR spectrum (600 MHz, DMSO-d_6) of compound **9a**.

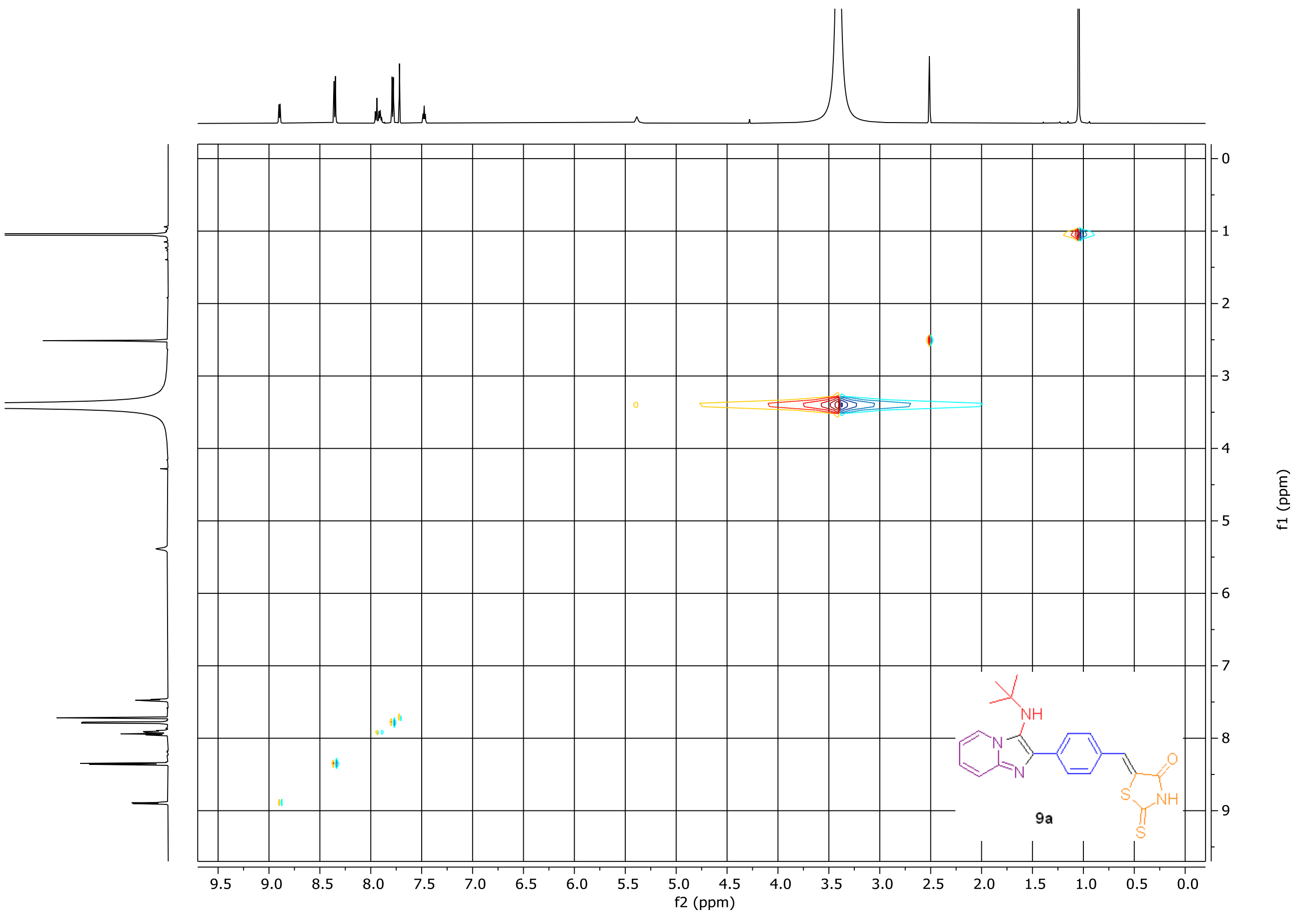

Figure S210. 2D NOESY NMR spectrum (600 MHz, DMSO-d_6) of compound **9a**.

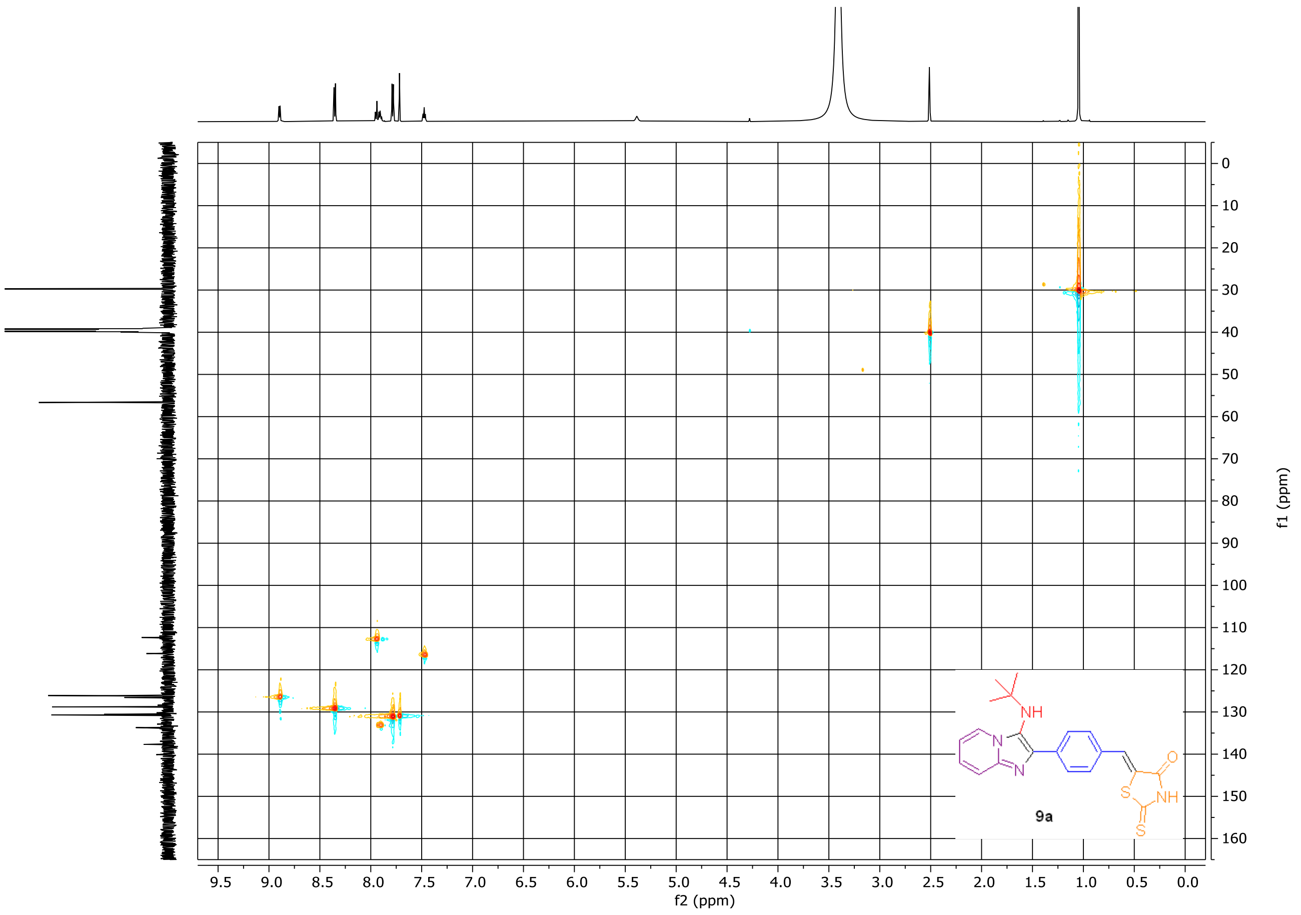

Figure S211. 2D HSQC NMR spectrum (600 MHz, DMSO-d_6) of compound **9a**.

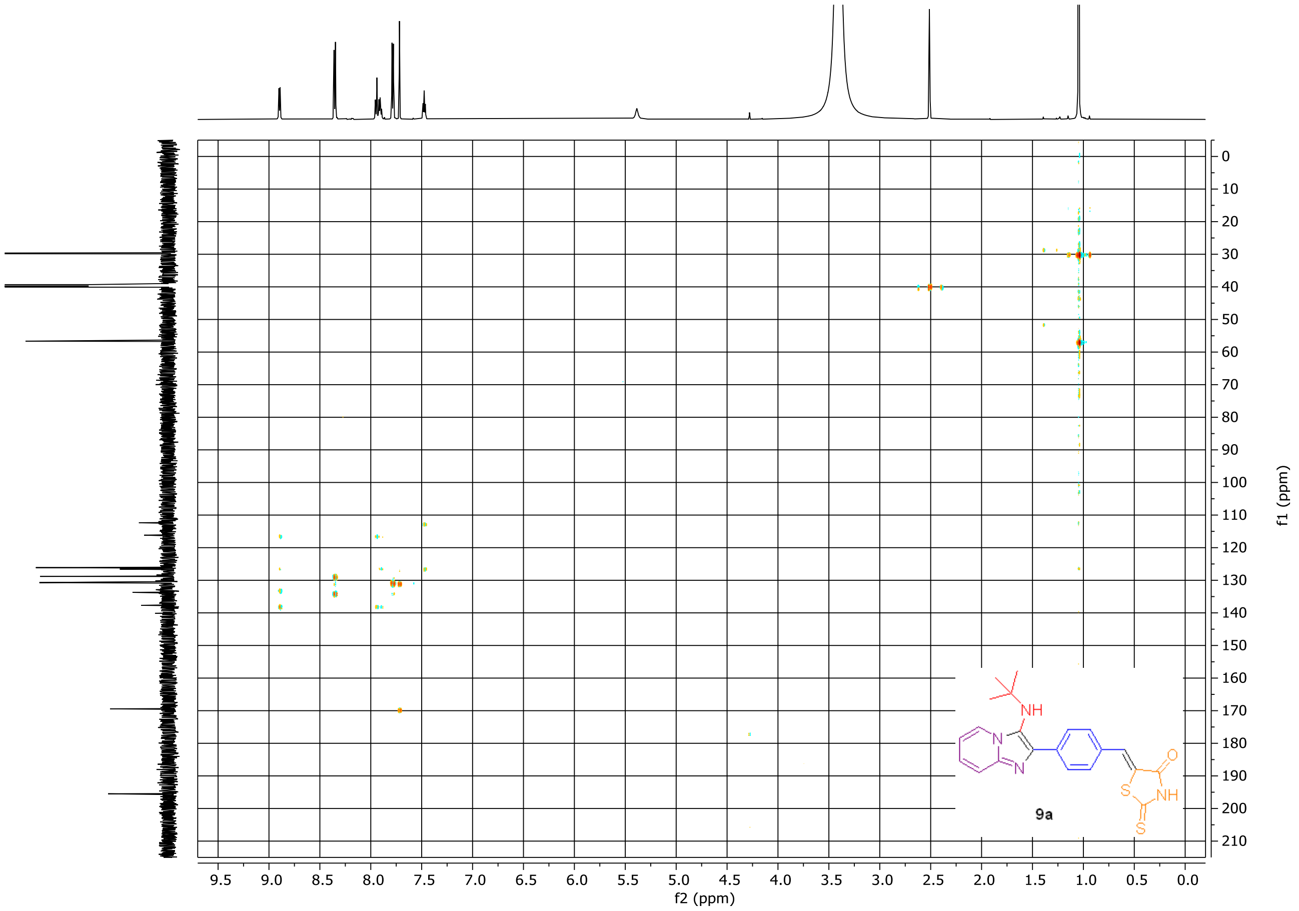

Figure S212. 2D HMBC NMR spectrum (600 MHz, DMSO-d_6) of compound **9a**.

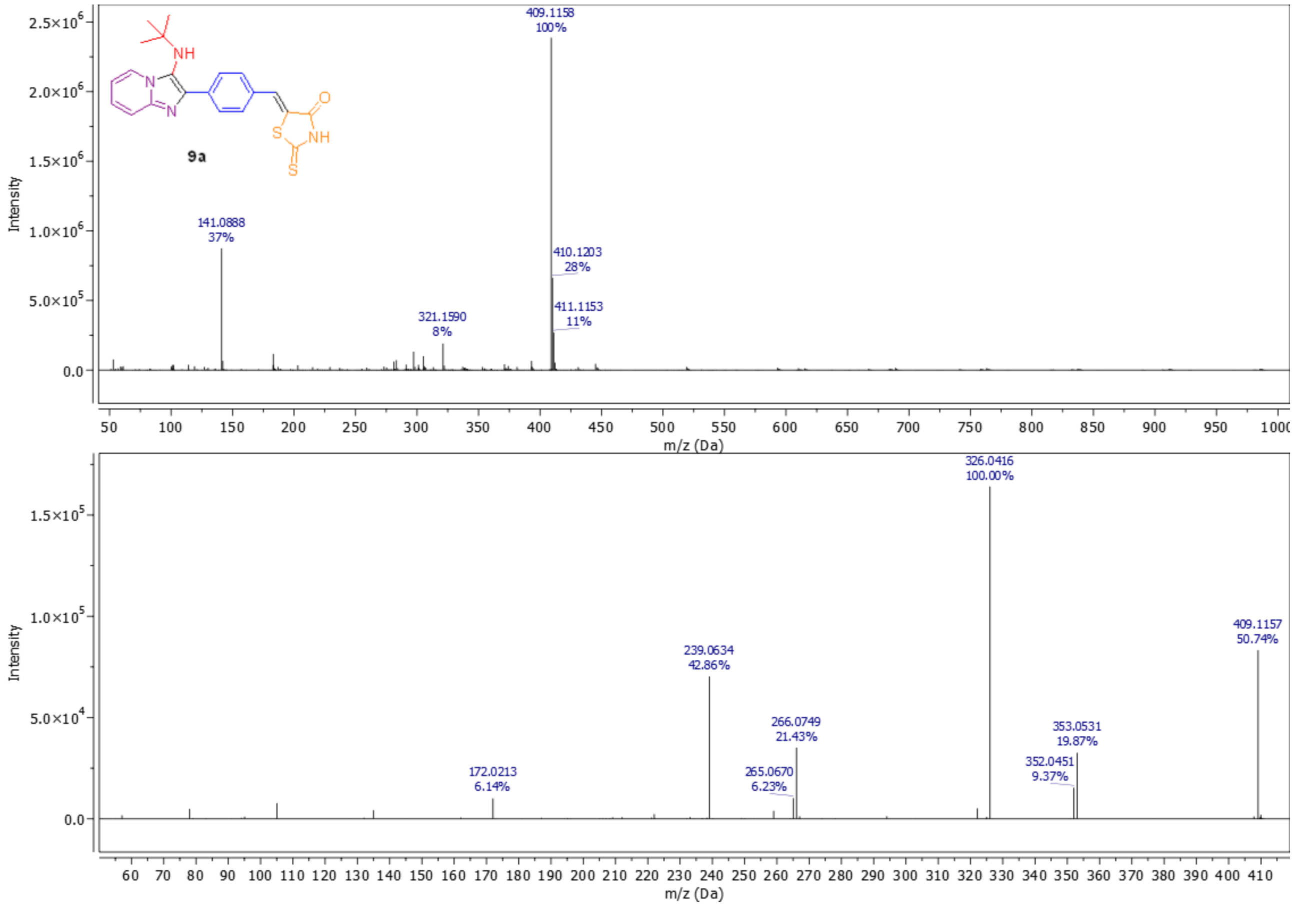

Figure S213. HRMS (ESI-QTOF) of compound **9a** and HRMS/MS for $[M+H]^+$.

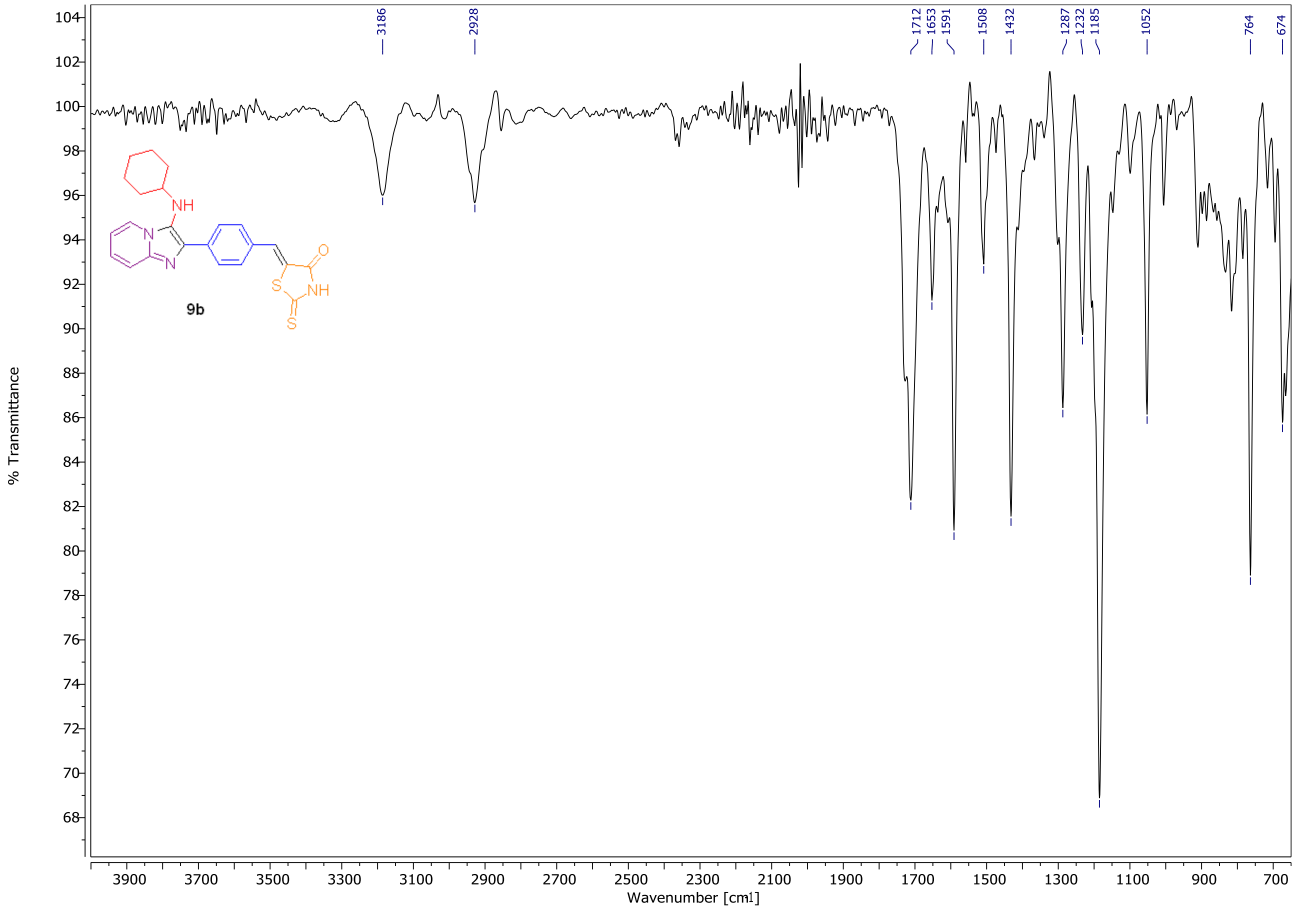

Figure S214. FTIR (ATR) of compound **9b**.

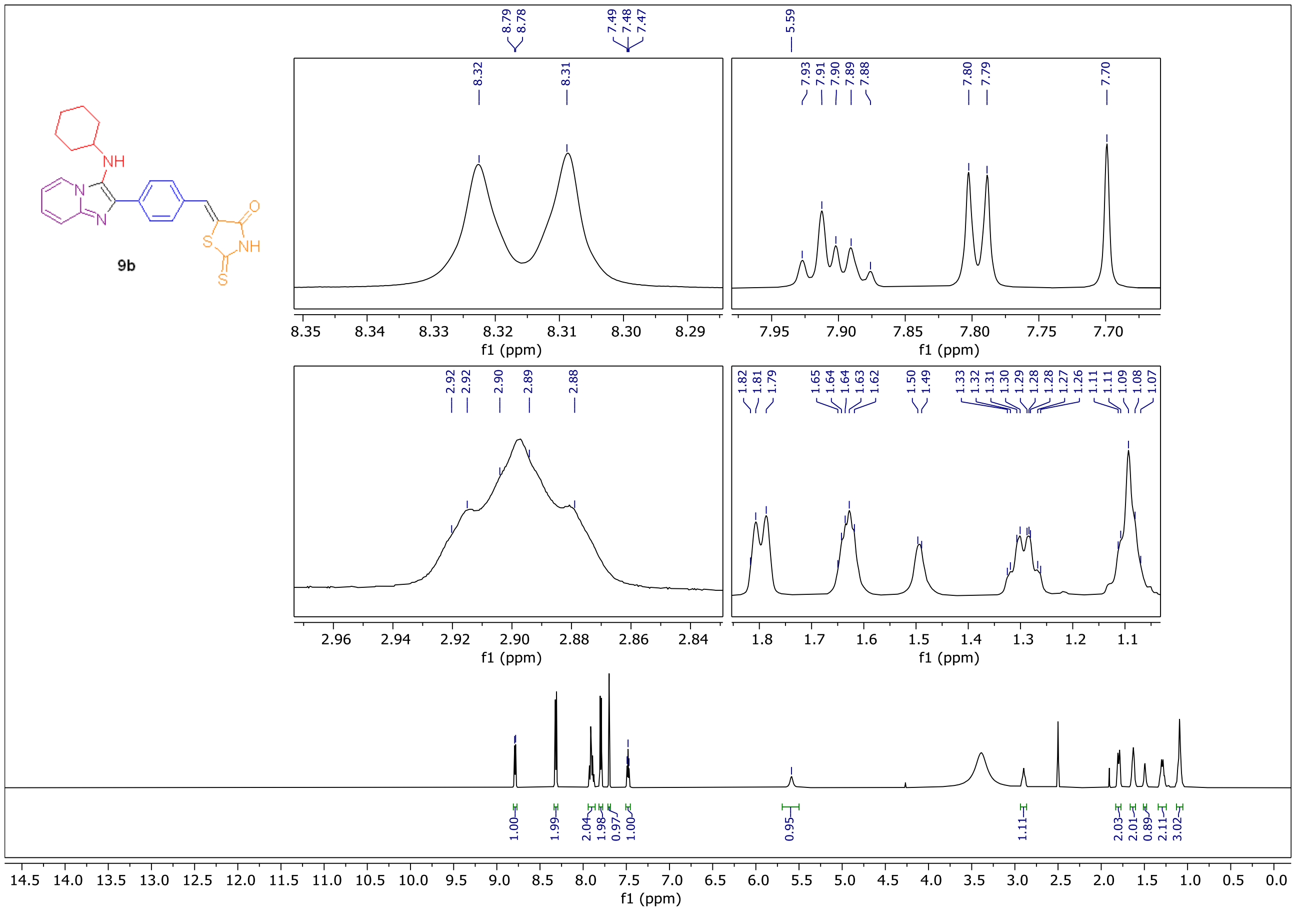

Figure S215. ^{1}H NMR spectrum (600 MHz, DMSO-d_6) of compound **9b**.

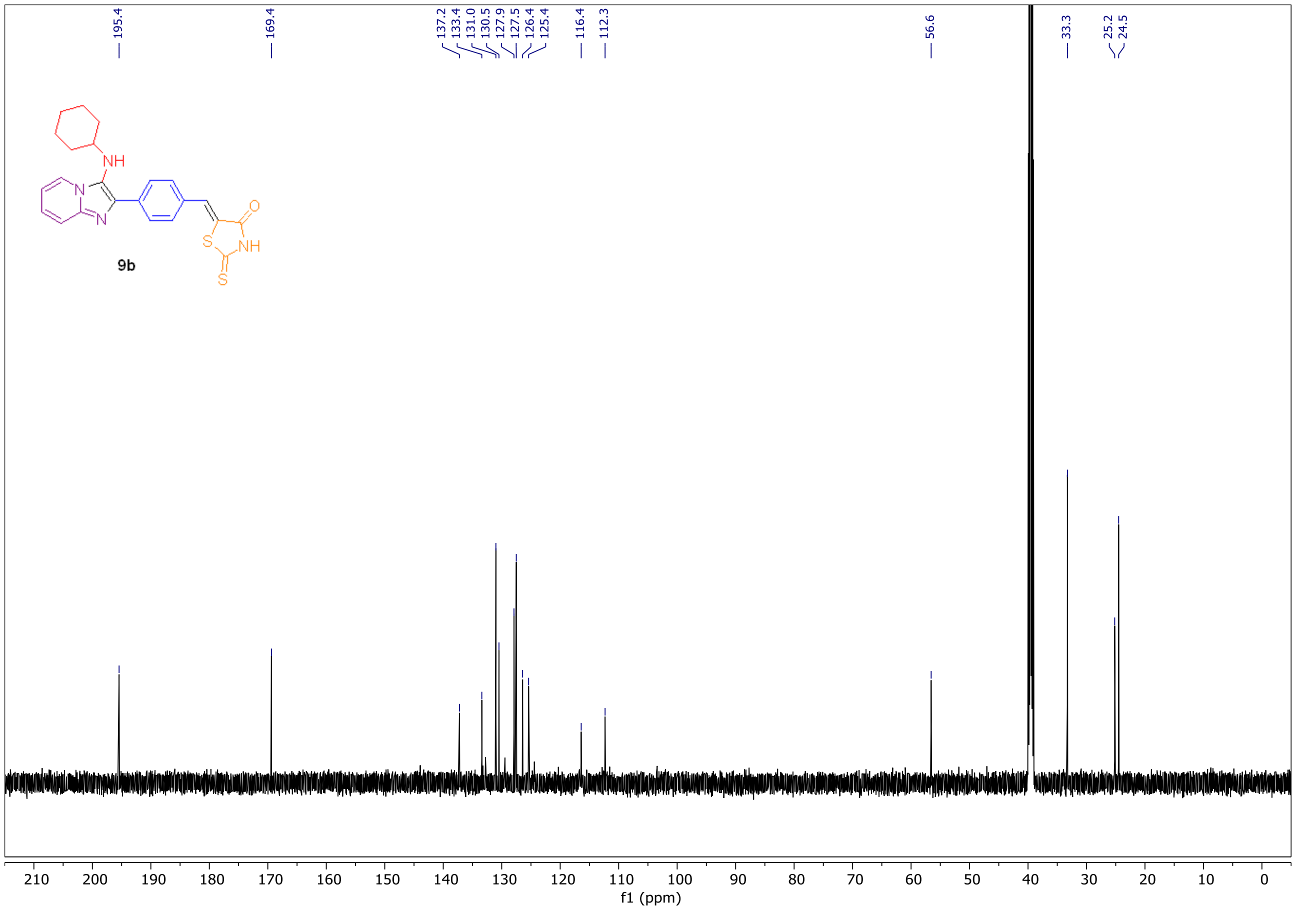

Figure S216. ^{13}C NMR spectrum (151 MHz, DMSO-d_6) of compound **9b**.

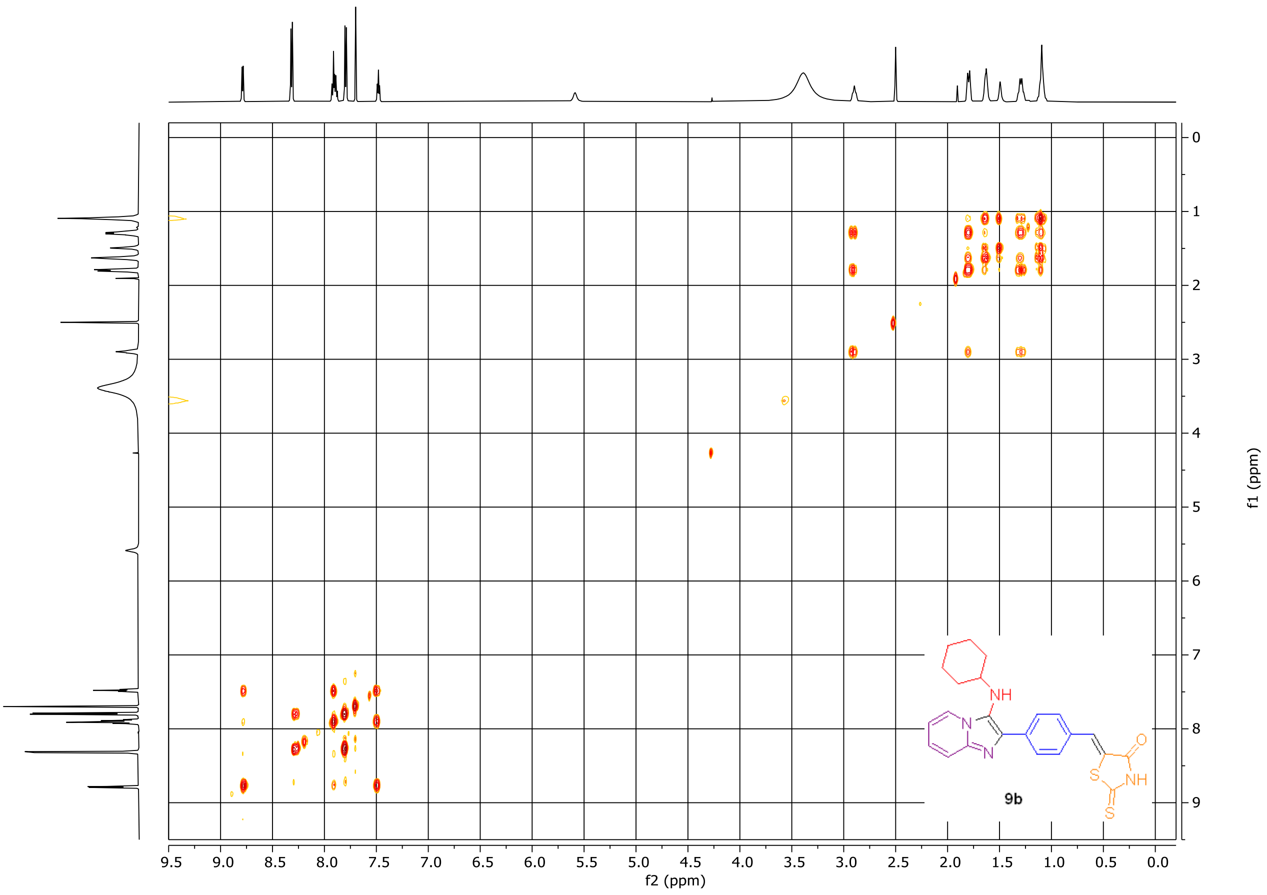

Figure S217. 2D COSY NMR spectrum (600 MHz, DMSO-d_6) of compound **9b**.

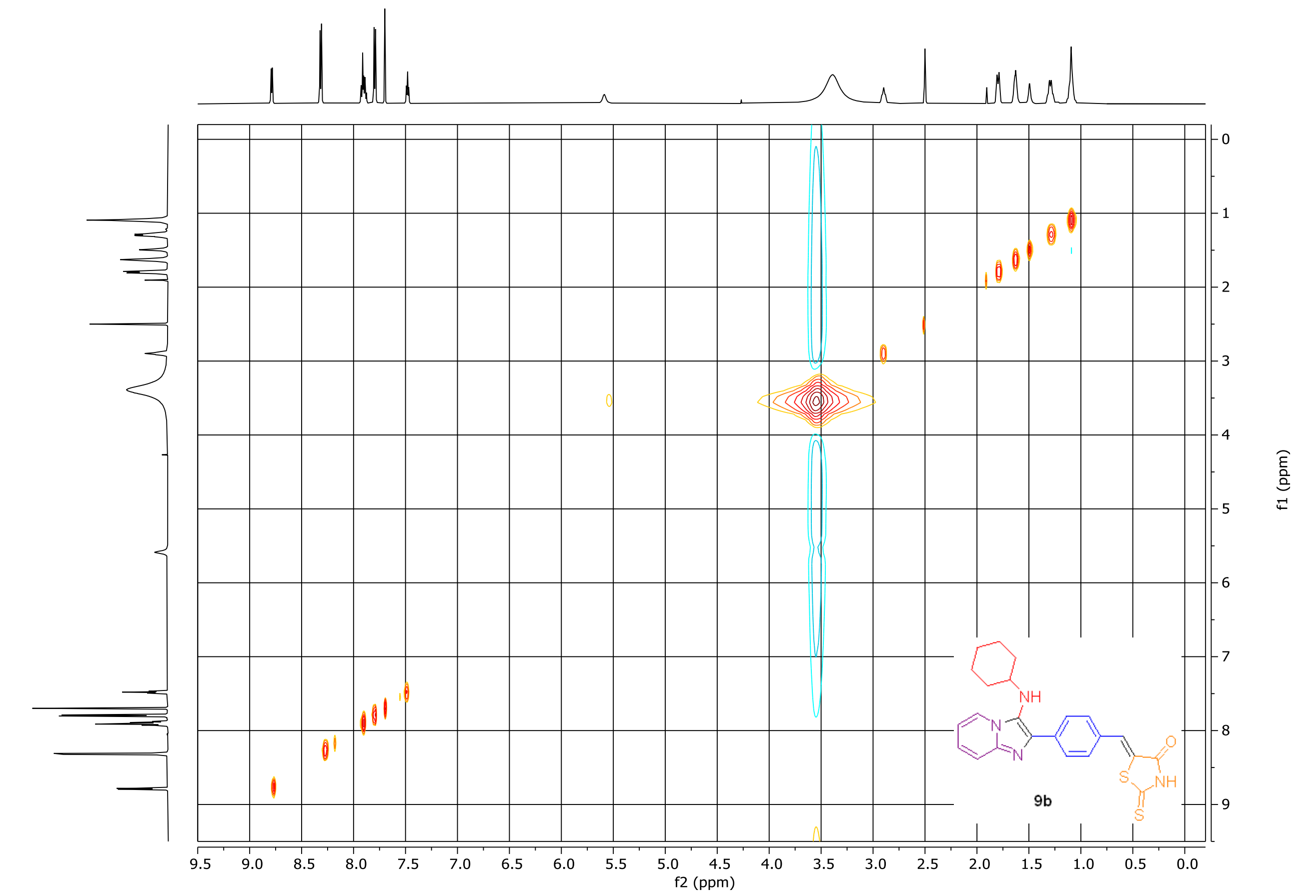

Figure S218. 2D NOESY NMR spectrum (600 MHz, DMSO-d_6) of compound **9b**.

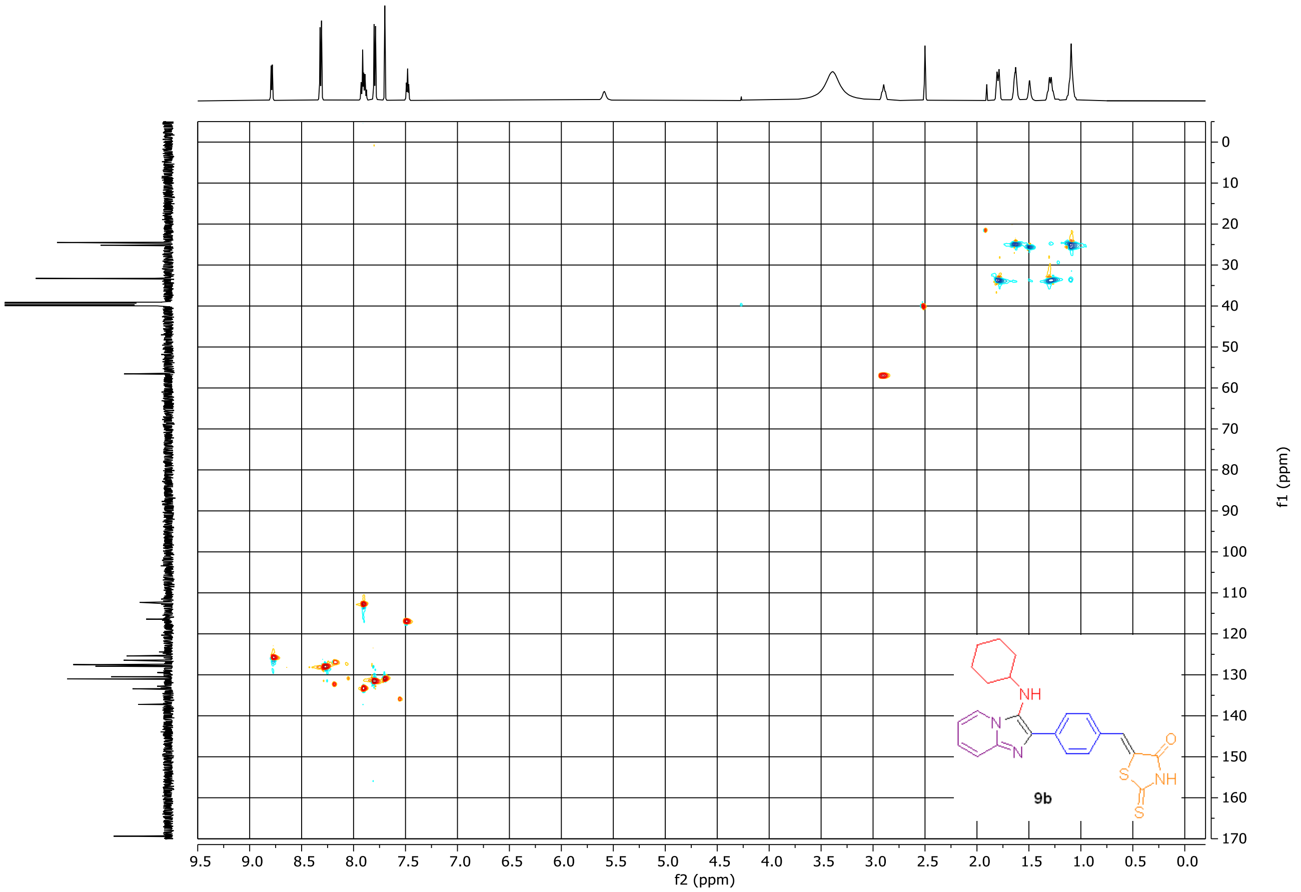

Figure S219. 2D HSQC NMR spectrum (600 MHz, DMSO-d_6) of compound **9b**.

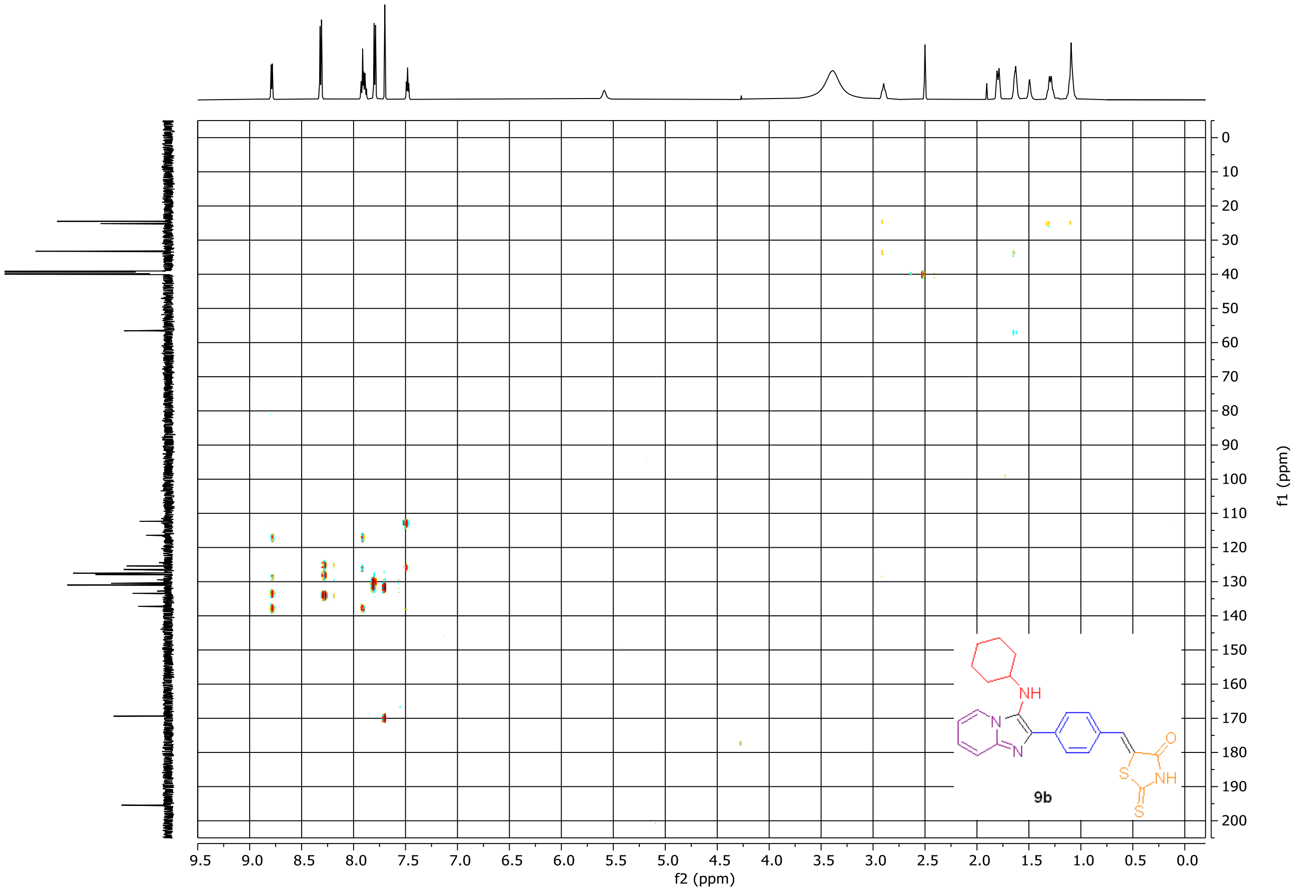

Figure S220. 2D HMBC NMR spectrum (600 MHz, DMSO-d_6) of compound **9b**.

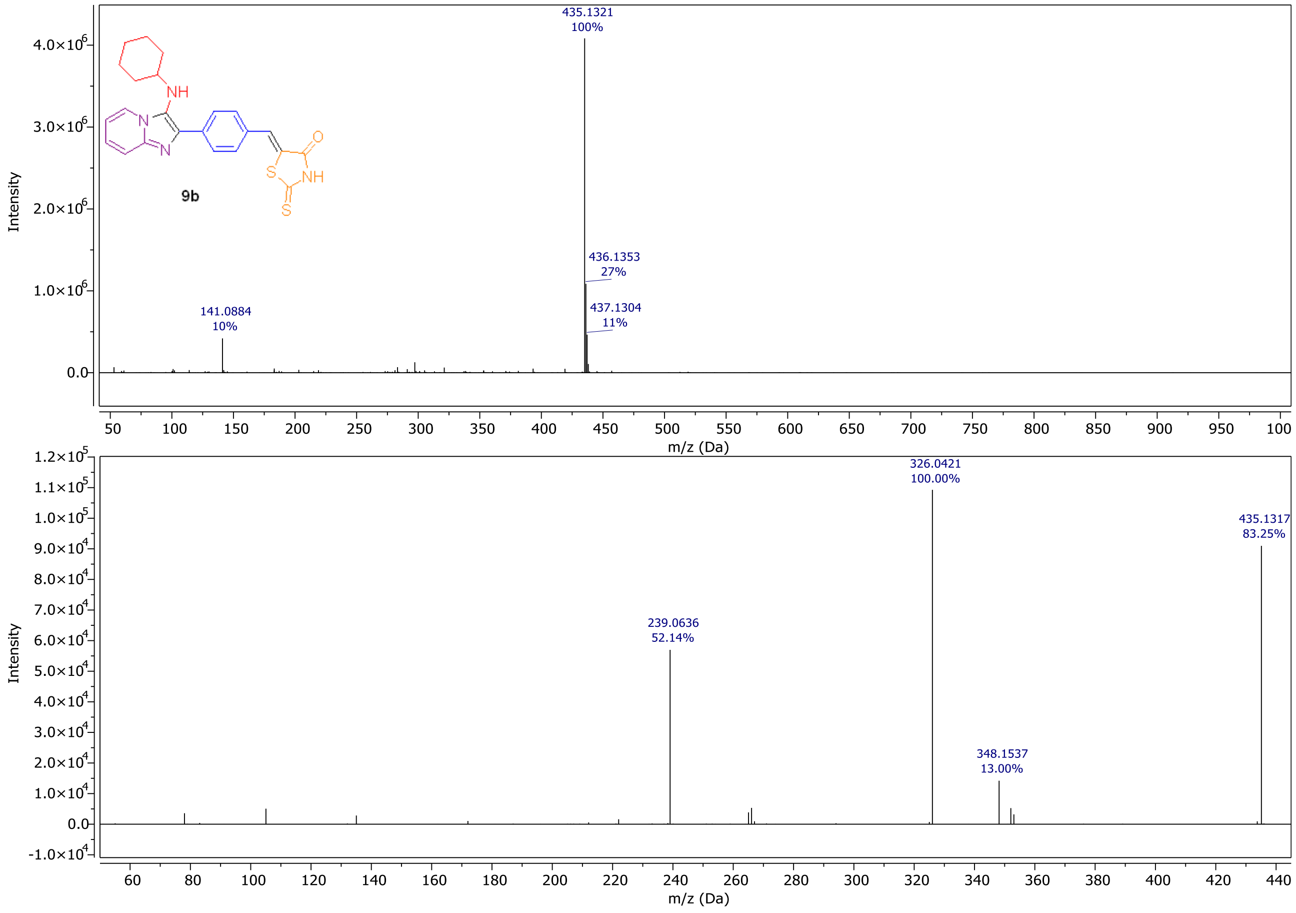

Figure S221. HRMS (ESI-QTOF) of compound **9b** and HRMS/MS for $[M+H]^+$.

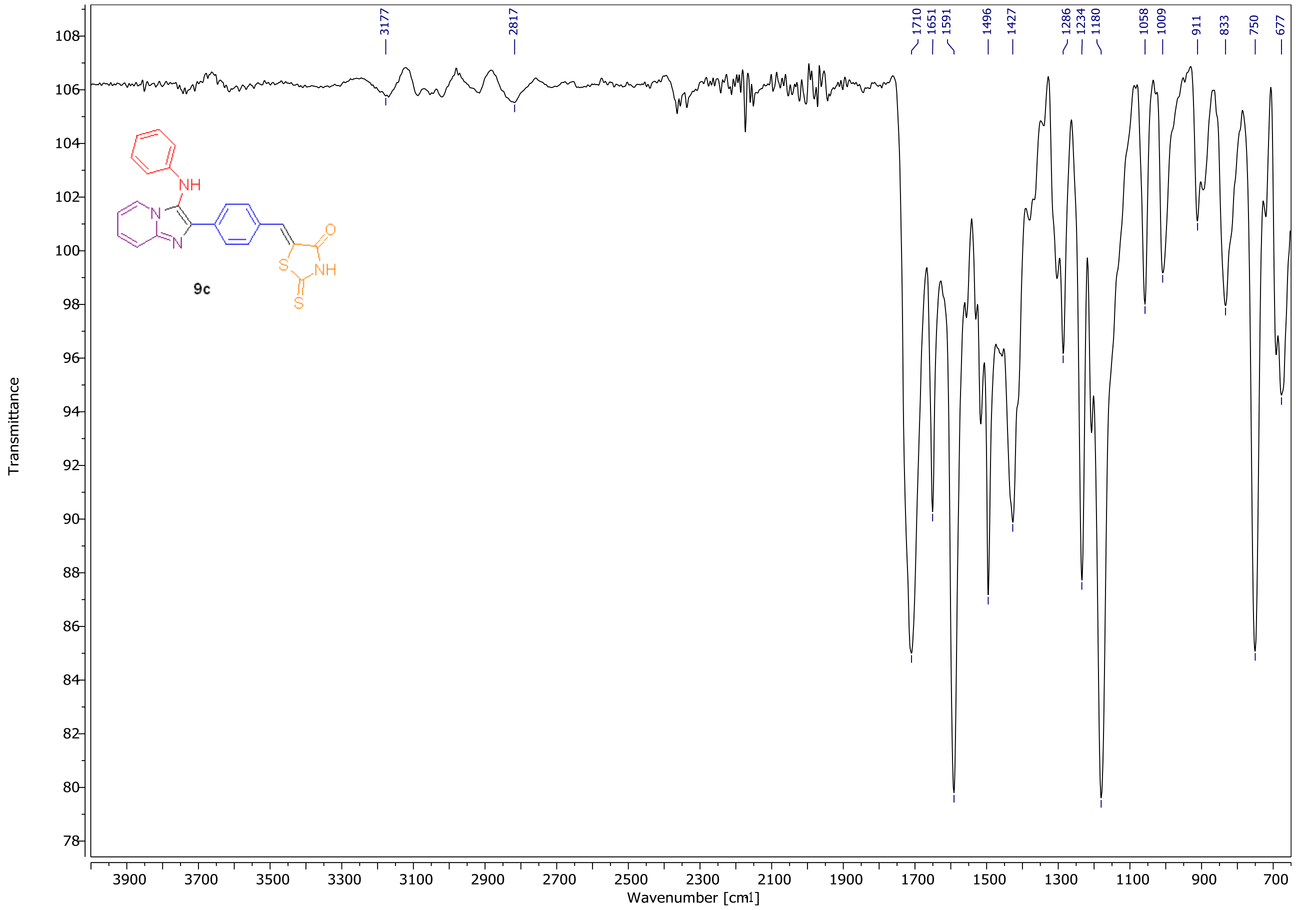

Figure S222. FTIR (ATR) of compound **9c**.

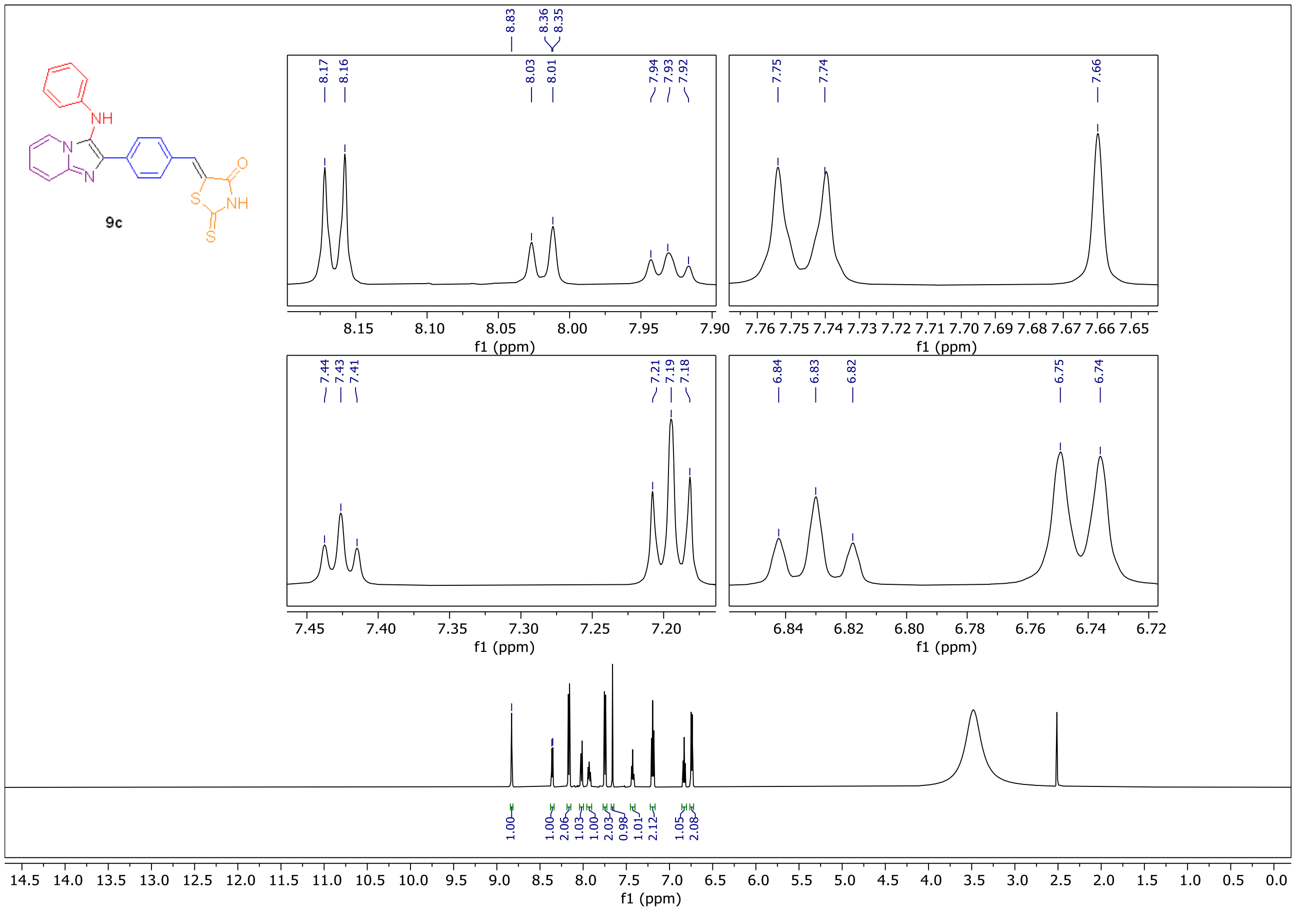

Figure S223. ^{1}H NMR spectrum (600 MHz, DMSO-d_6) of compound **9c**.

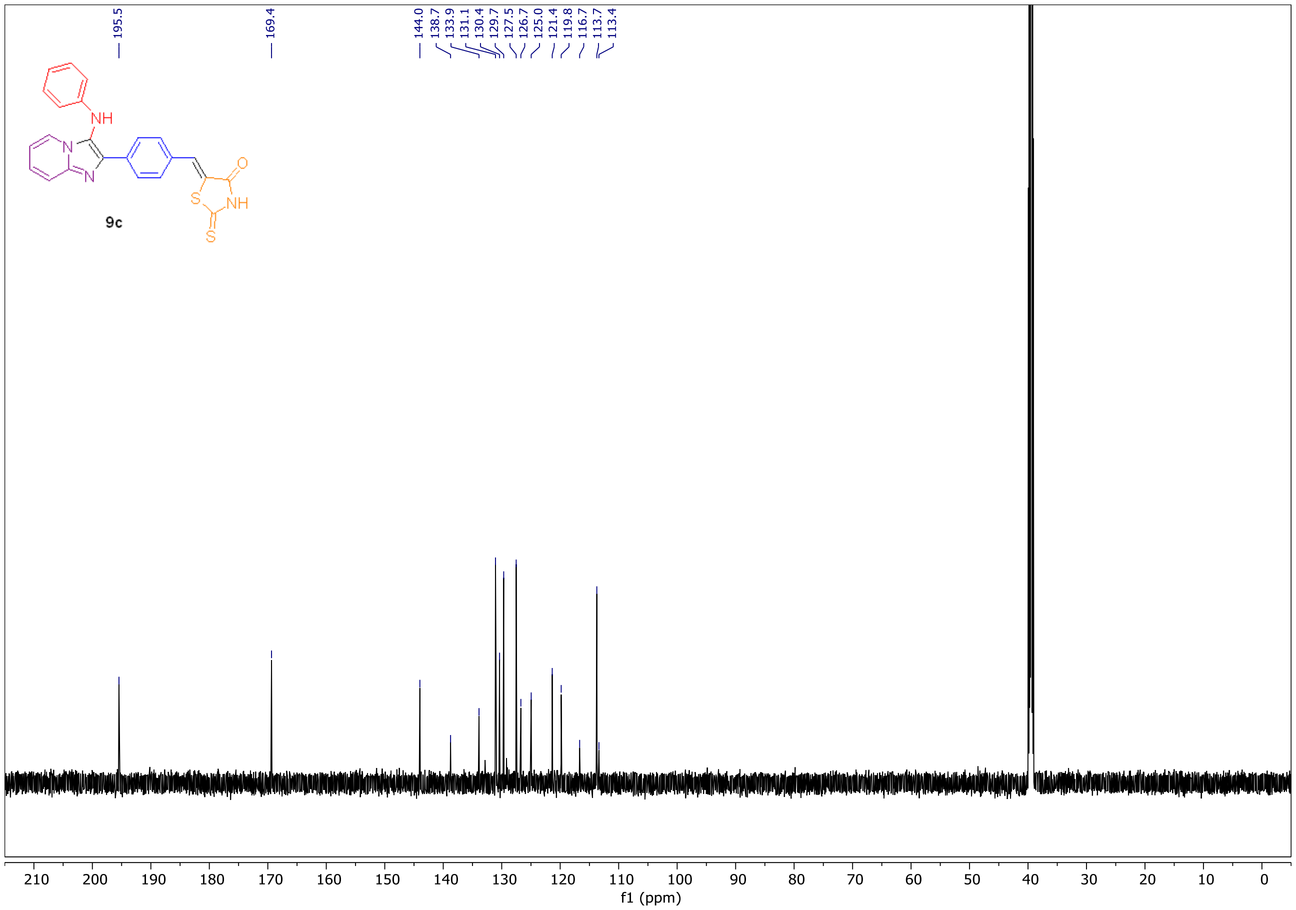

Figure S224. ^{13}C NMR spectrum (151 MHz, DMSO-d_6) of compound **9c**.

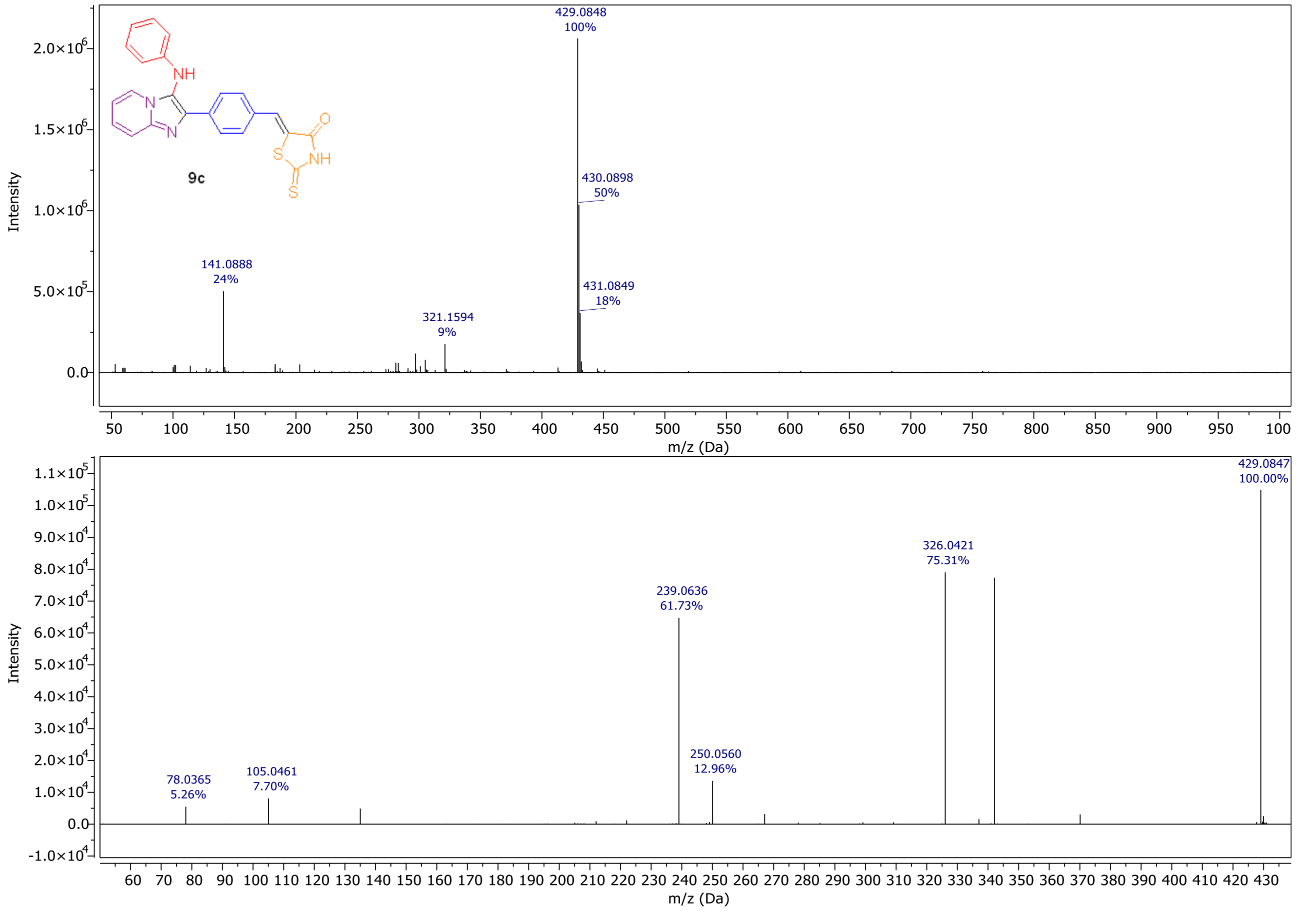

Figure S225. HRMS (ESI-QTOF) of compound **9c** and HRMS/MS for [M+H]$^+$.

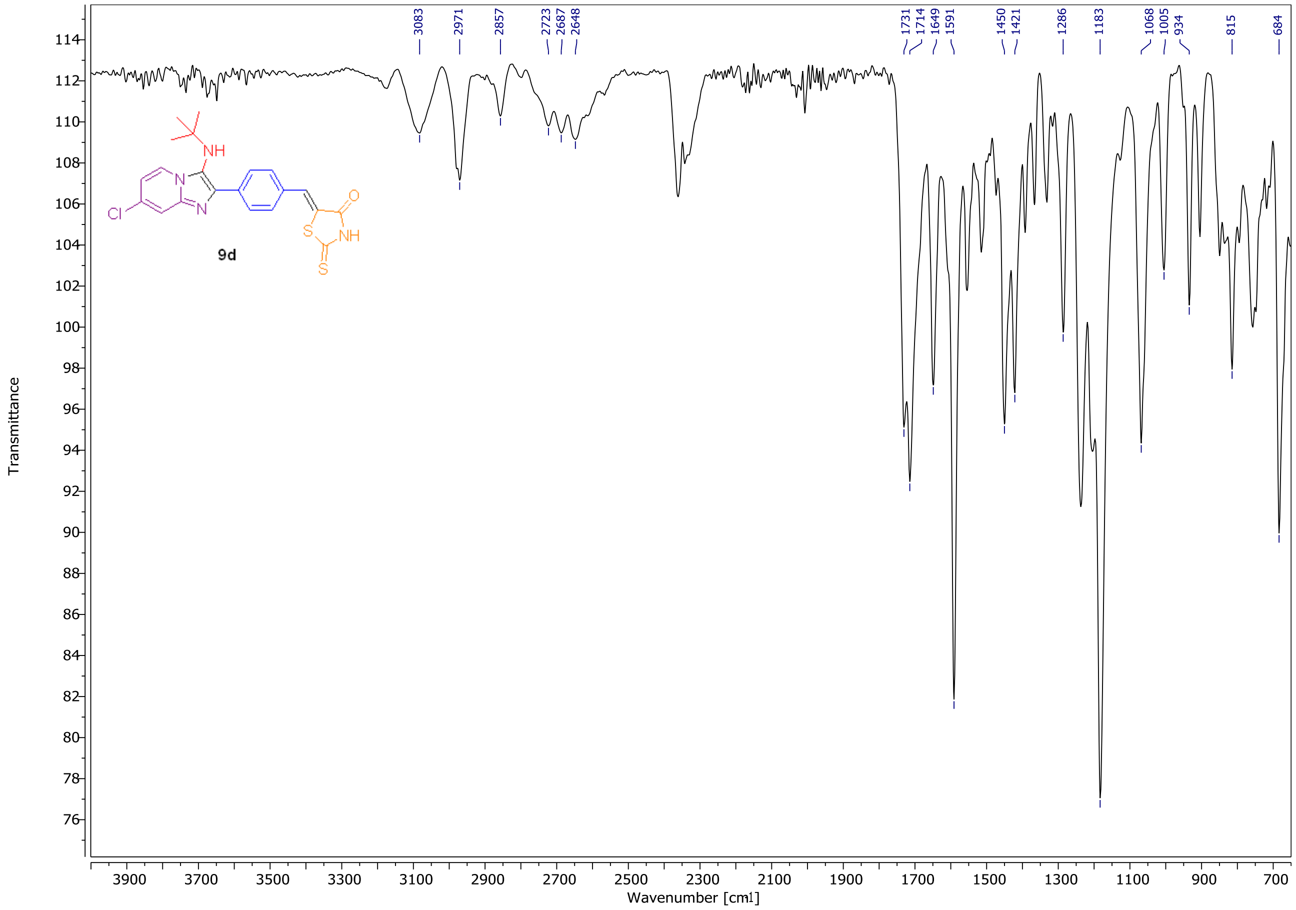

Figure S226. FTIR (ATR) of compound **9d**.

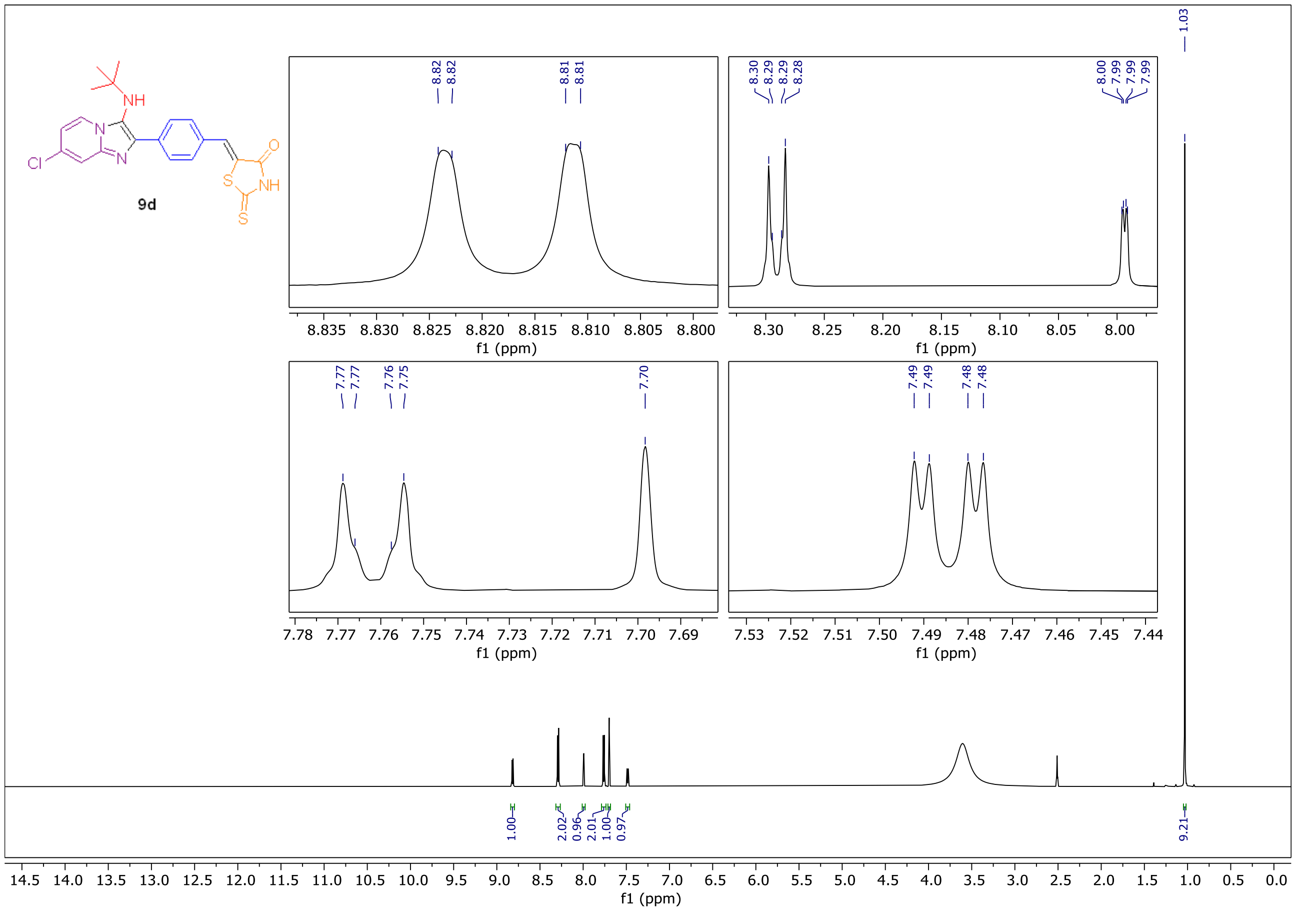

Figure S227. ^{1}H NMR spectrum (600 MHz, DMSO-d_6) of compound **9d**.

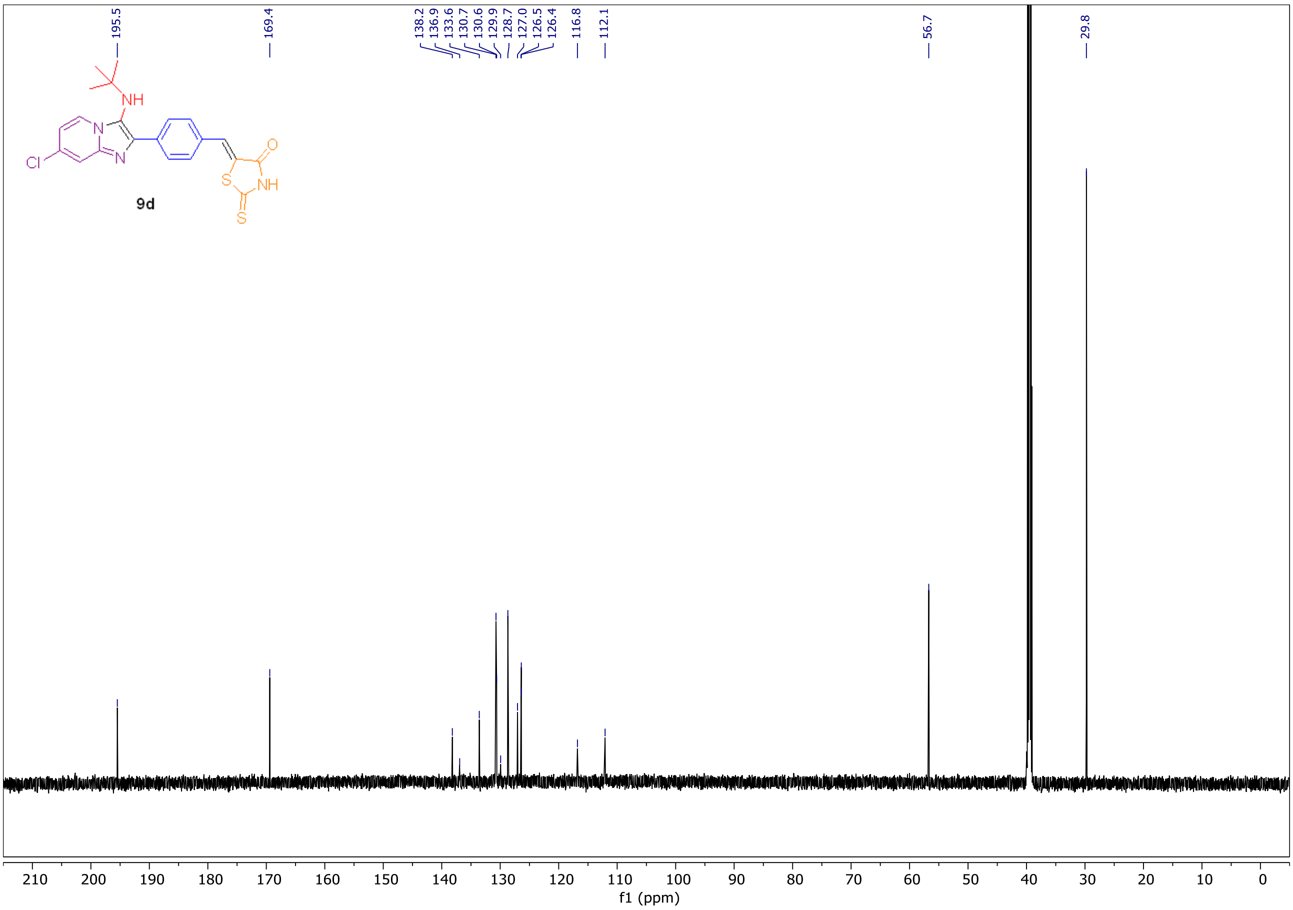

Figure S228. ^{13}C NMR spectrum (151 MHz, DMSO-d_6) of compound **9d**.

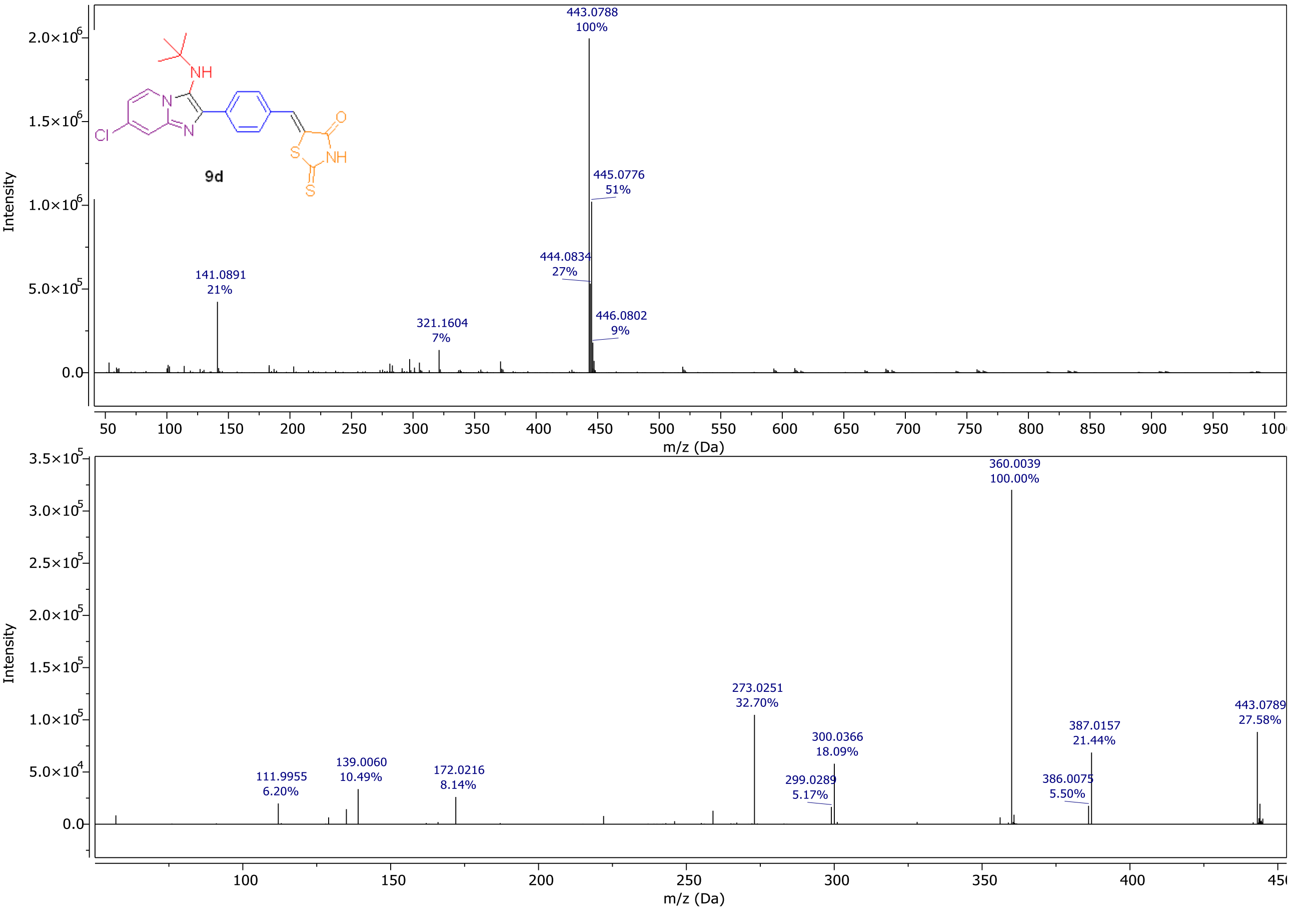

Figure S229. HRMS (ESI-QTOF) of compound **9d** and HRMS/MS for $[M+H]^+$.

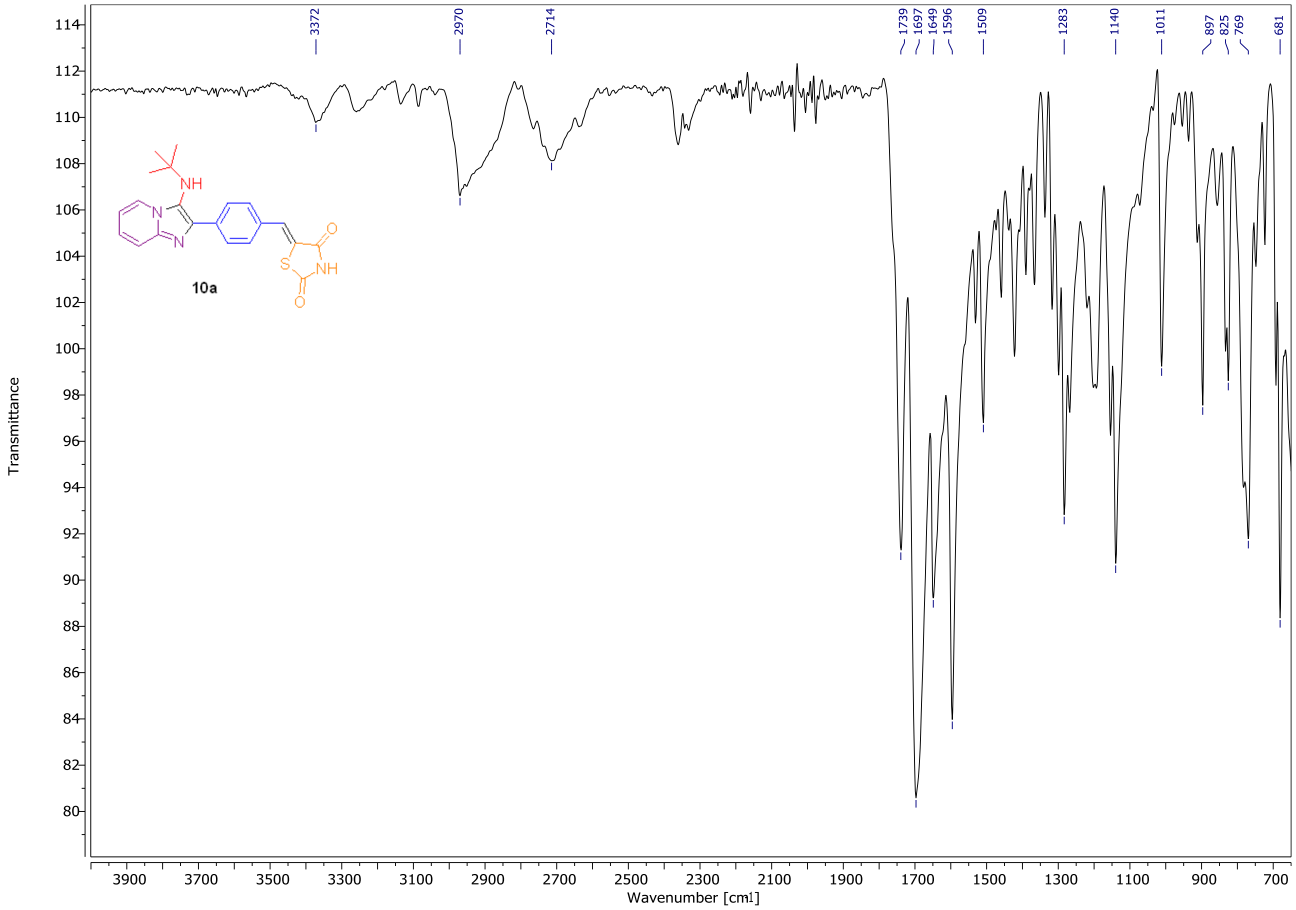

Figure S230. FTIR (ATR) of compound **10a**.

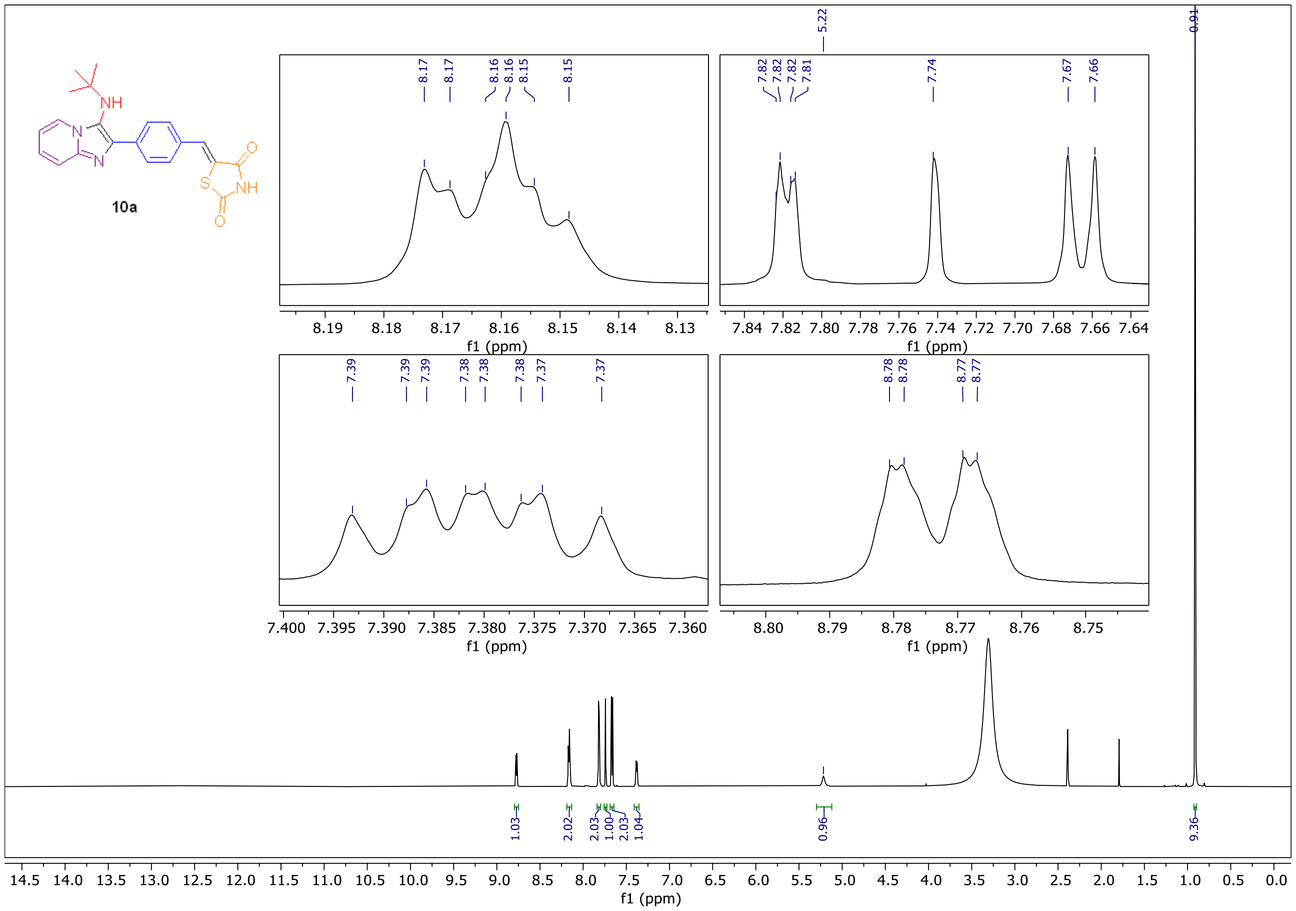

Figure S231. ^{1}H NMR spectrum (600 MHz, DMSO-d_6) of compound **10a**.

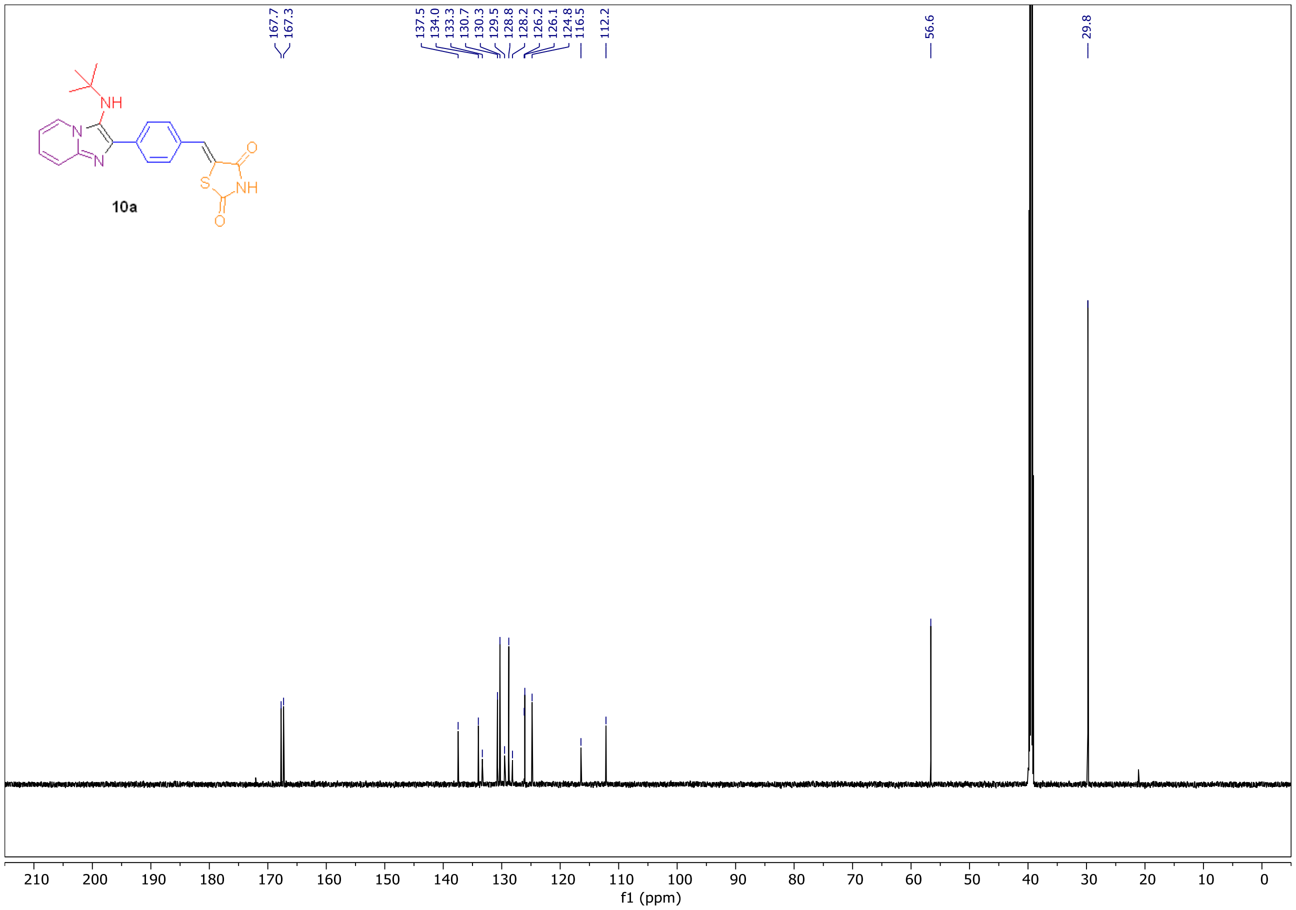

Figure S232. ^{13}C NMR spectrum (151 MHz, DMSO-d_6) of compound **10a**.

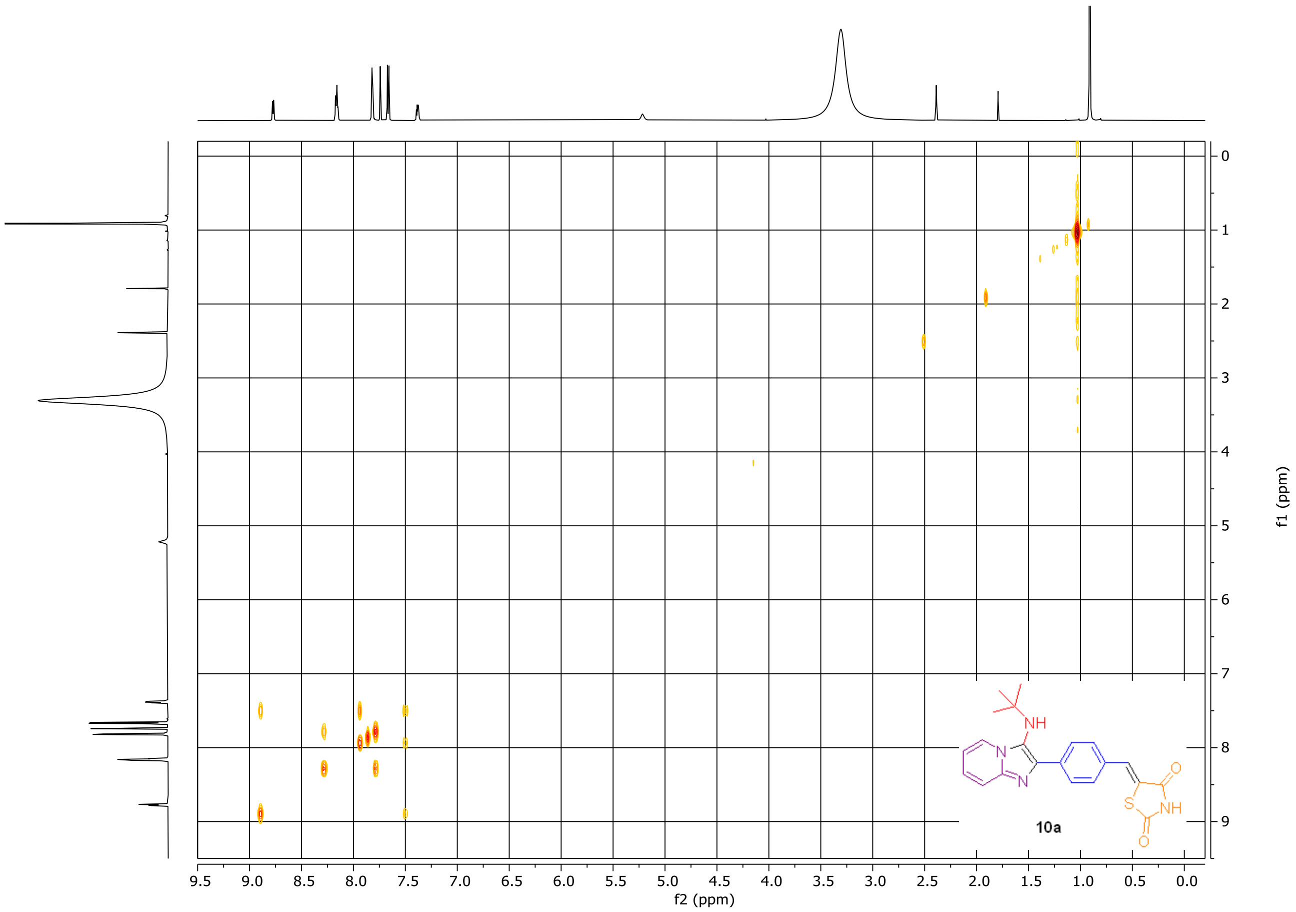

Figure S233. 2D COSY NMR spectrum (600 MHz, DMSO-d_6) of compound **10a**.

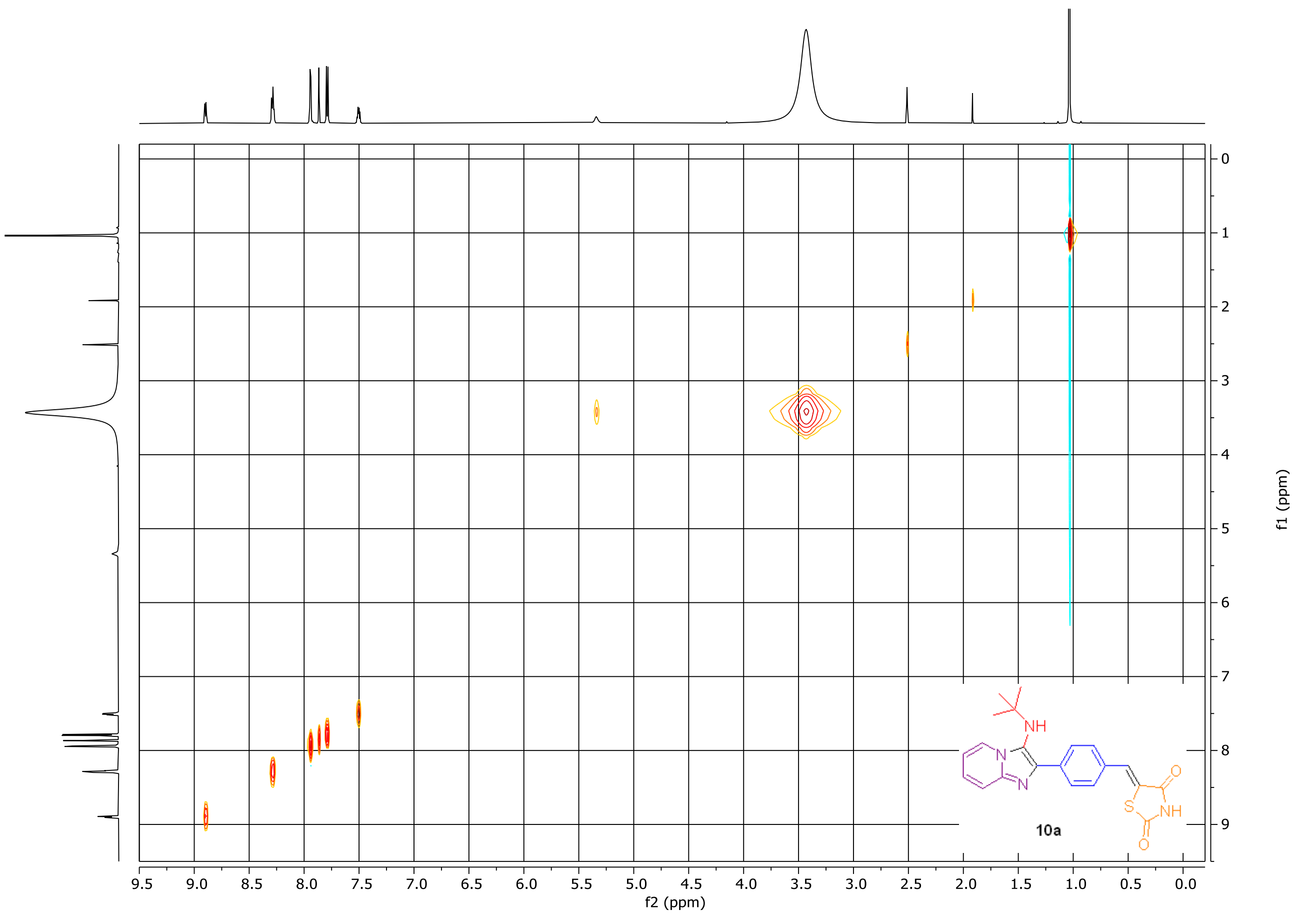

Figure S234. 2D NOESY NMR spectrum (600 MHz, DMSO-d_6) of compound **10a**.

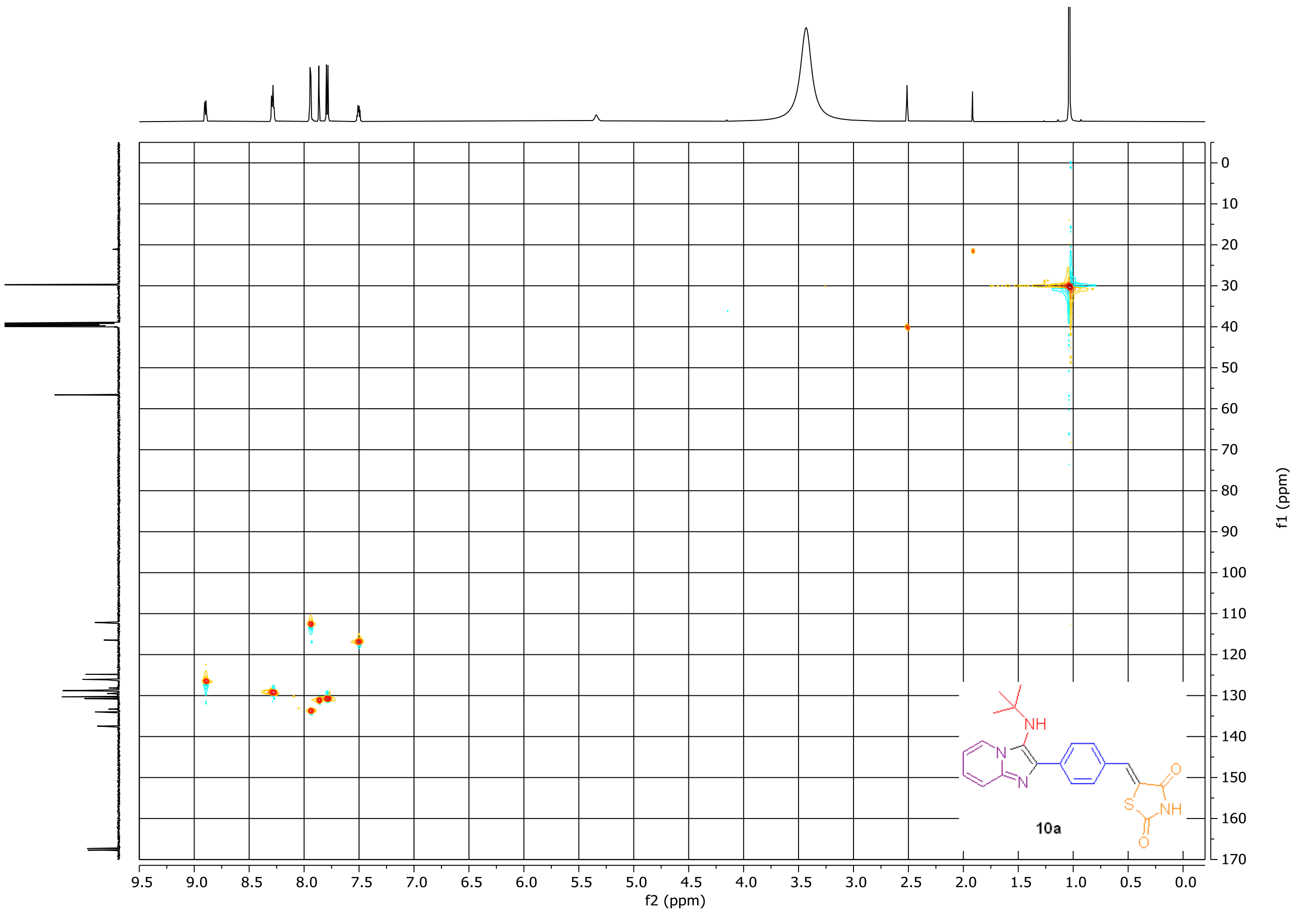

Figure S235. 2D HSQC NMR spectrum (600 MHz, DMSO-d_6) of compound **10a**.

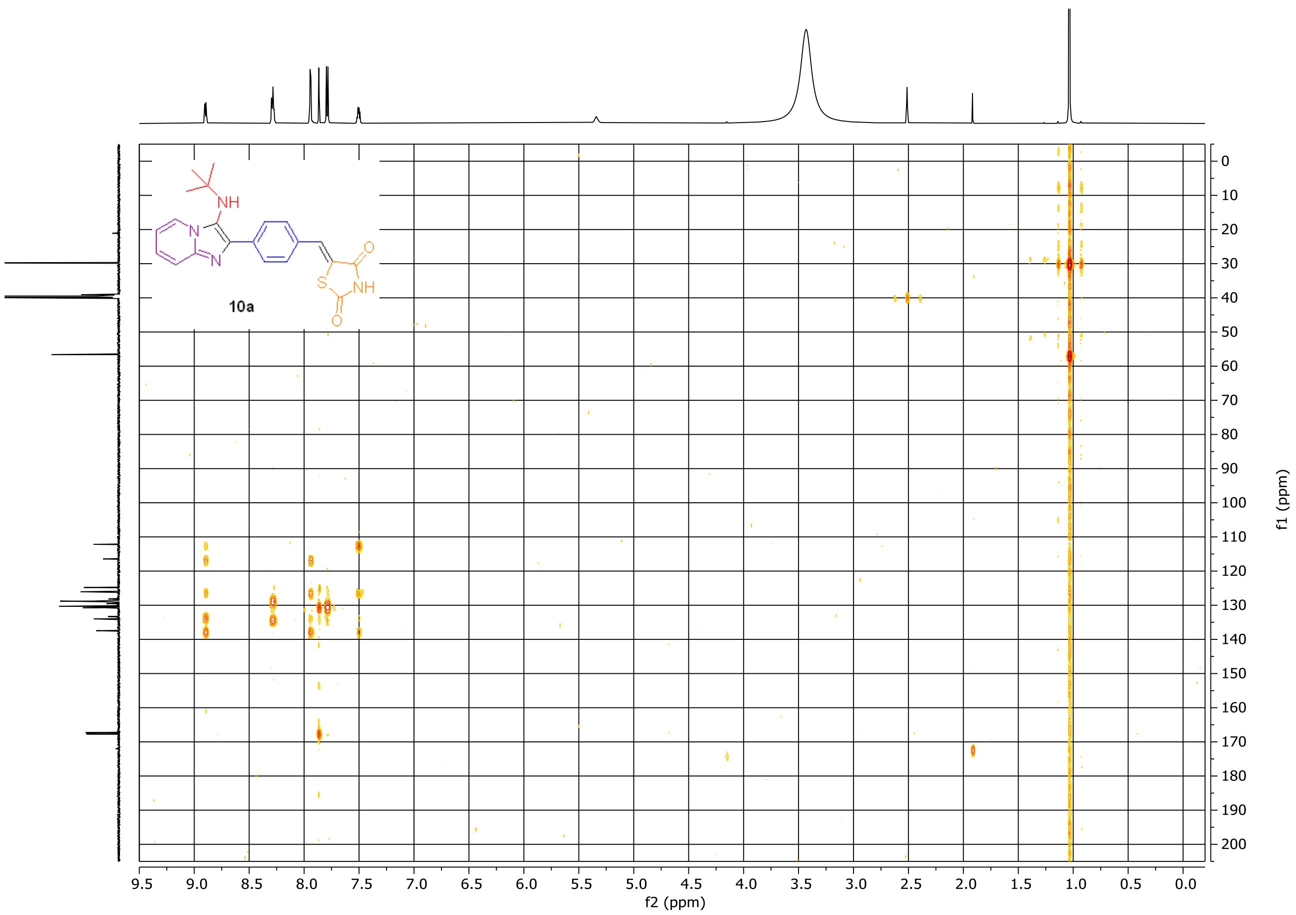

Figure S236. 2D HMBC NMR spectrum (600 MHz, DMSO-d_6) of compound **10a**.

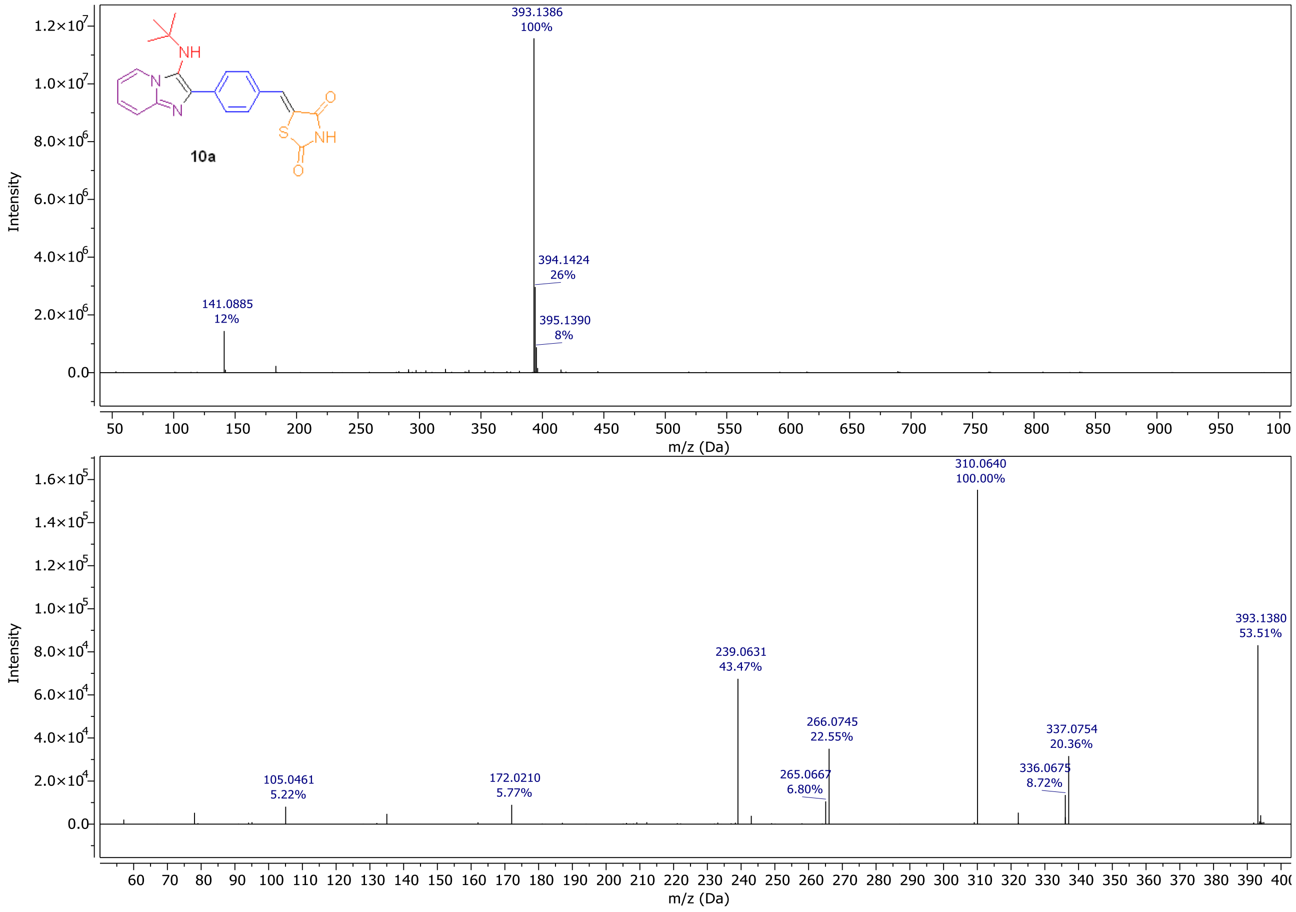

Figure S237. HRMS (ESI-QTOF) of compound **10a** and HRMS/MS for $[M+H]^+$.

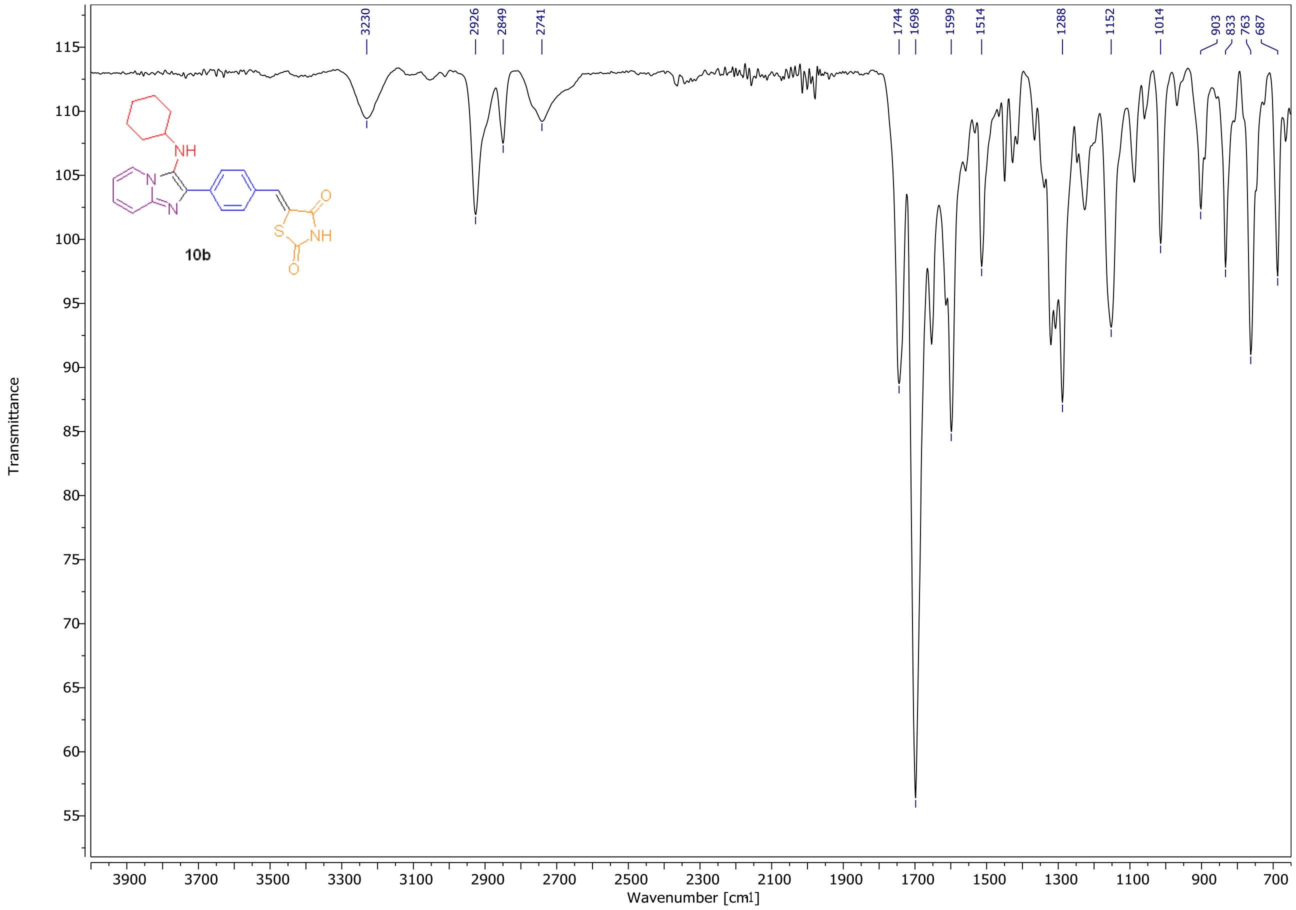

Figure S238. FTIR (ATR) of compound **10b**.

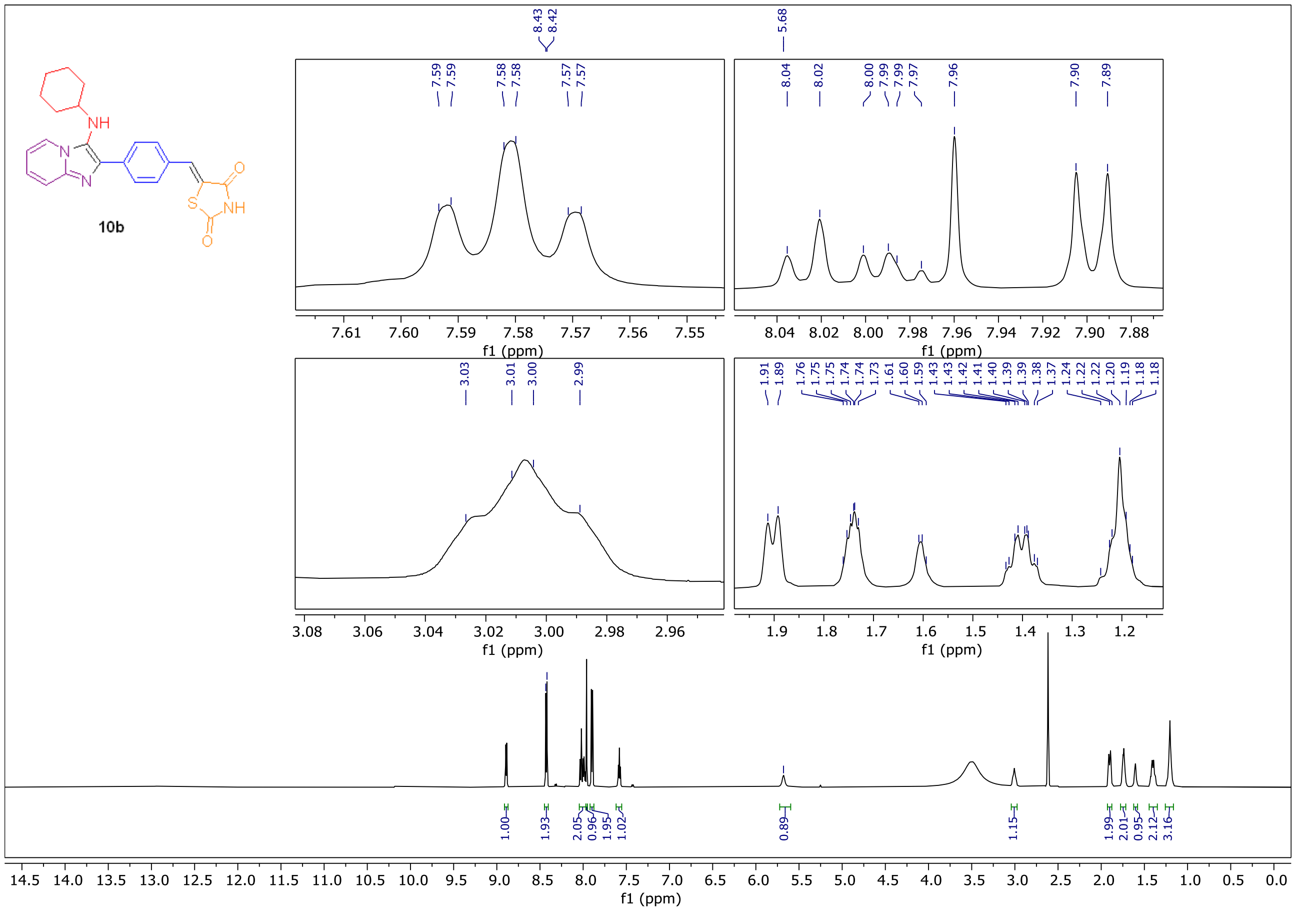

Figure S239. ^{1}H NMR spectrum (600 MHz, DMSO-d_6) of compound **10b**.

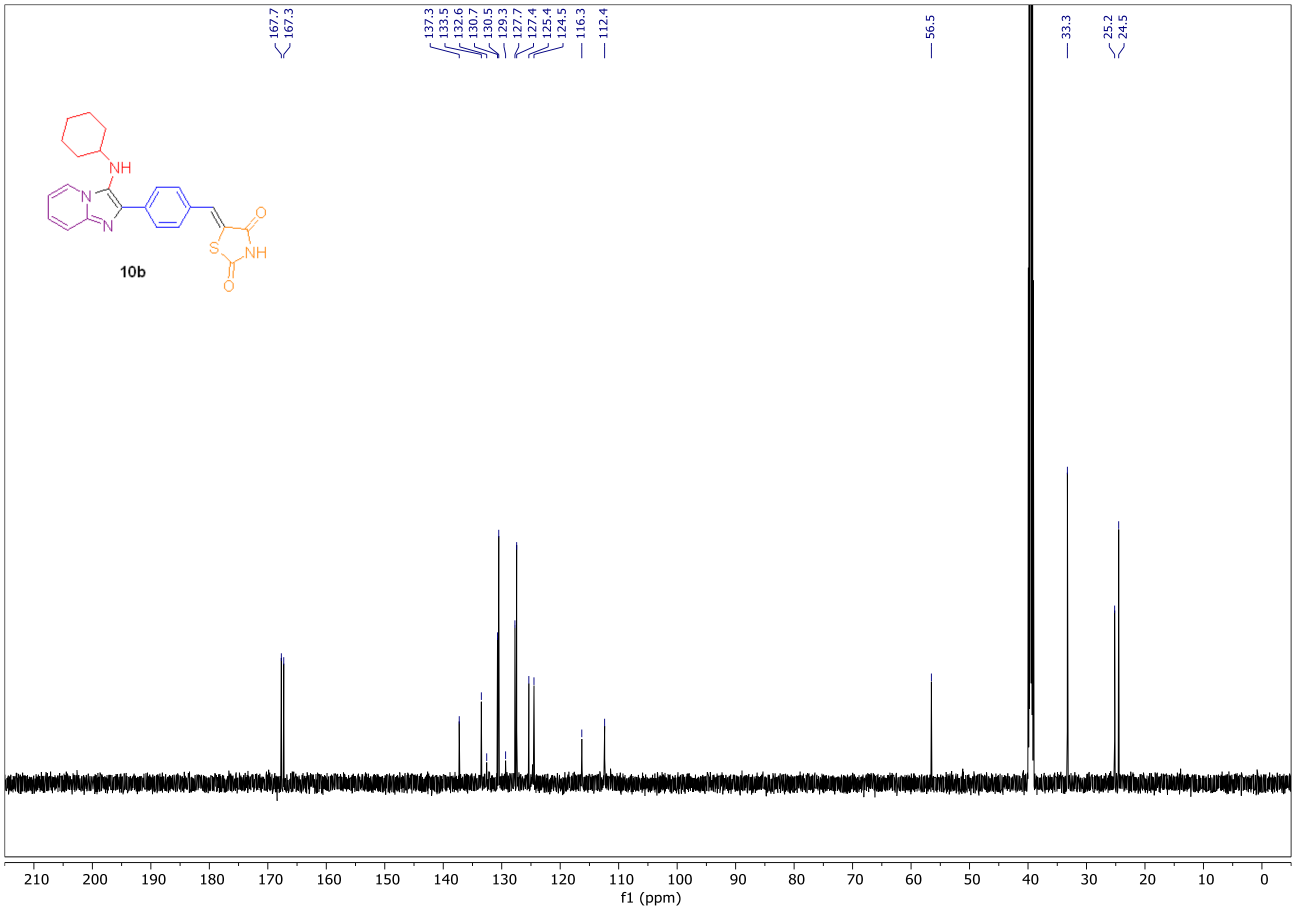

Figure S240. ^{13}C NMR spectrum (151 MHz, DMSO-d_6) of compound **10b**.

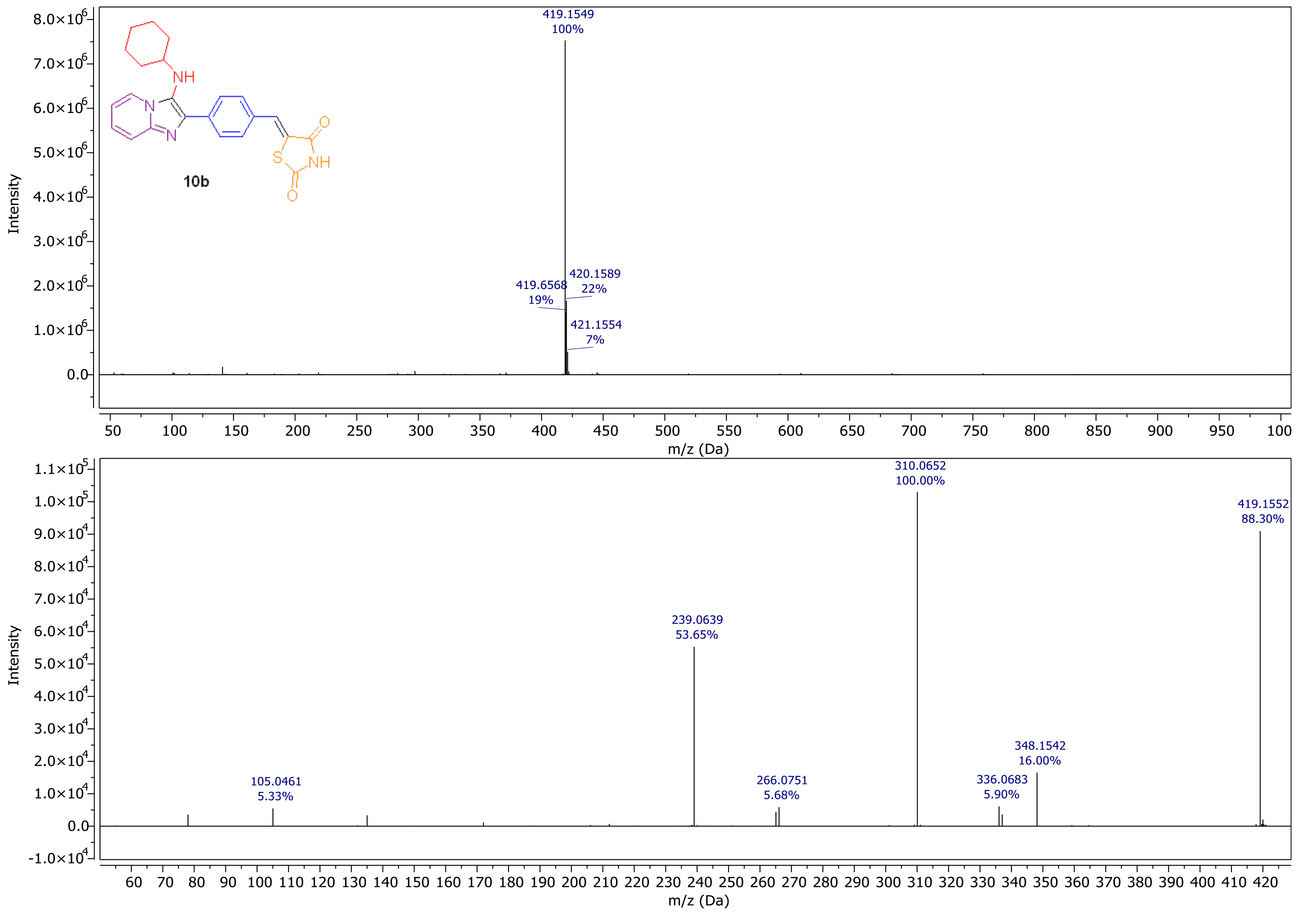

Figure S241. HRMS (ESI-QTOF) of compound **10b** and HRMS/MS for $[M+H]^+$.

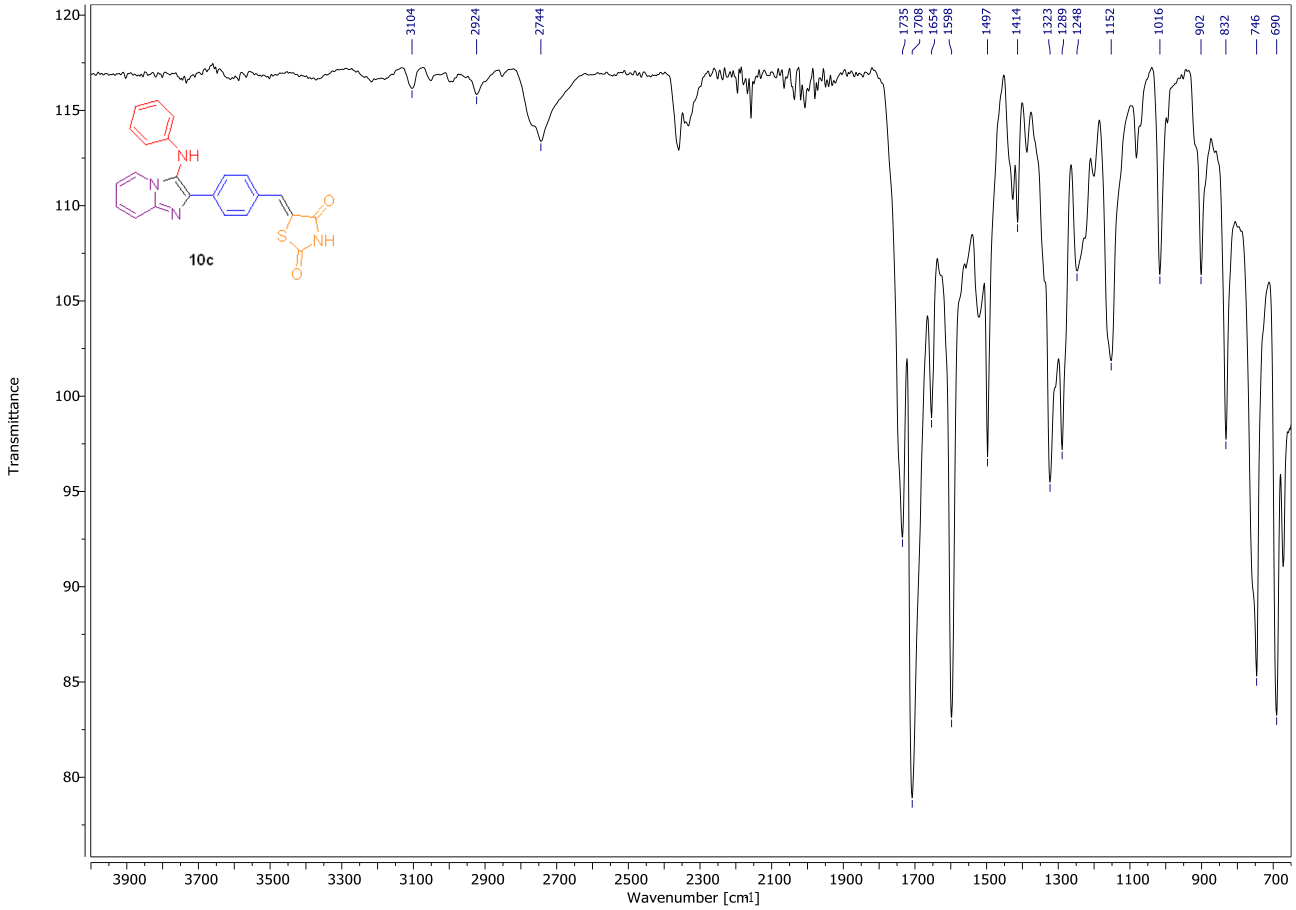

Figure S242. FTIR (ATR) of compound **10c**.

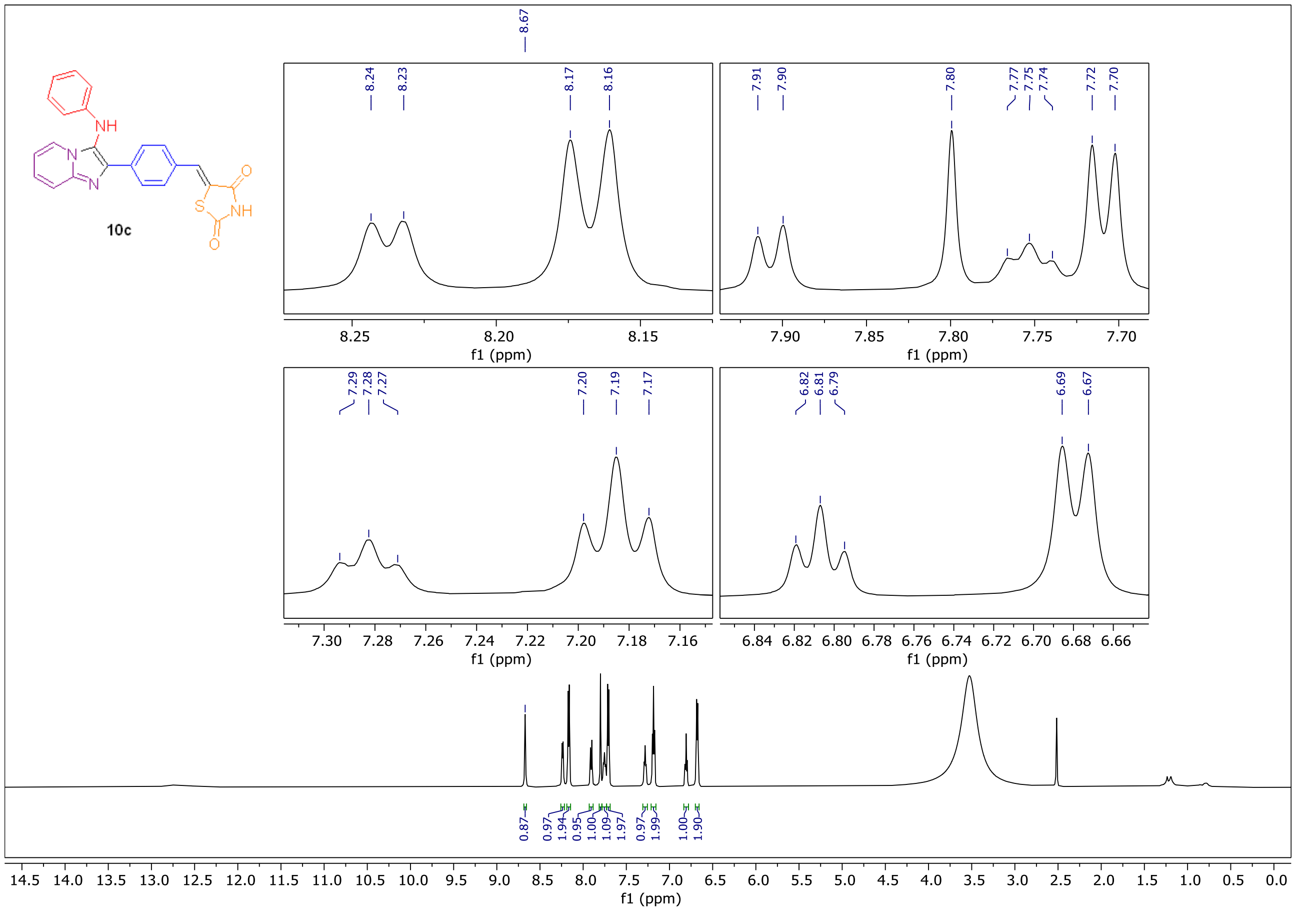

Figure S243. ^{1}H NMR spectrum (600 MHz, DMSO-d_6) of compound **10c**.

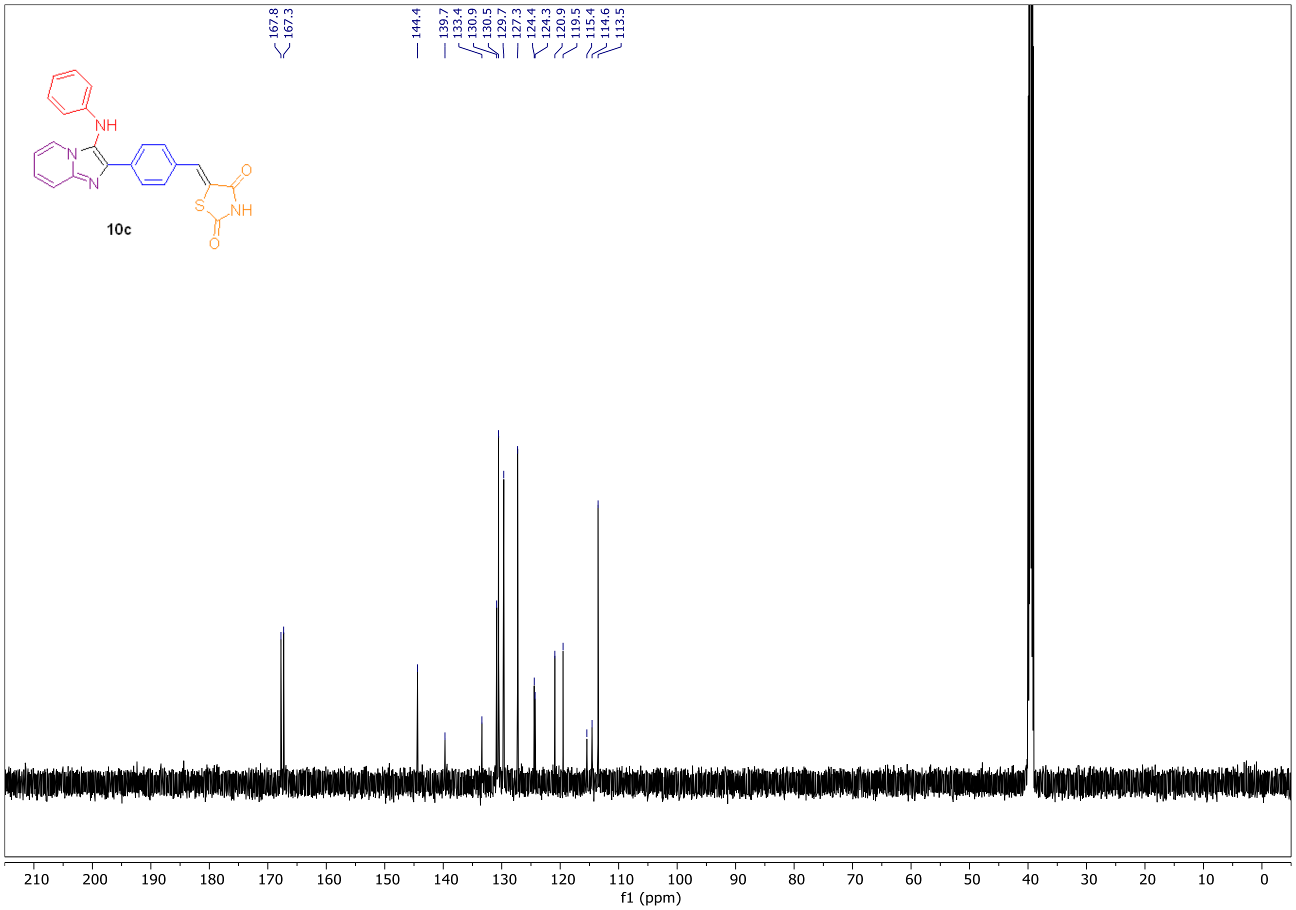

Figure S244. ^{13}C NMR spectrum (151 MHz, DMSO-d_6) of compound **10c**.

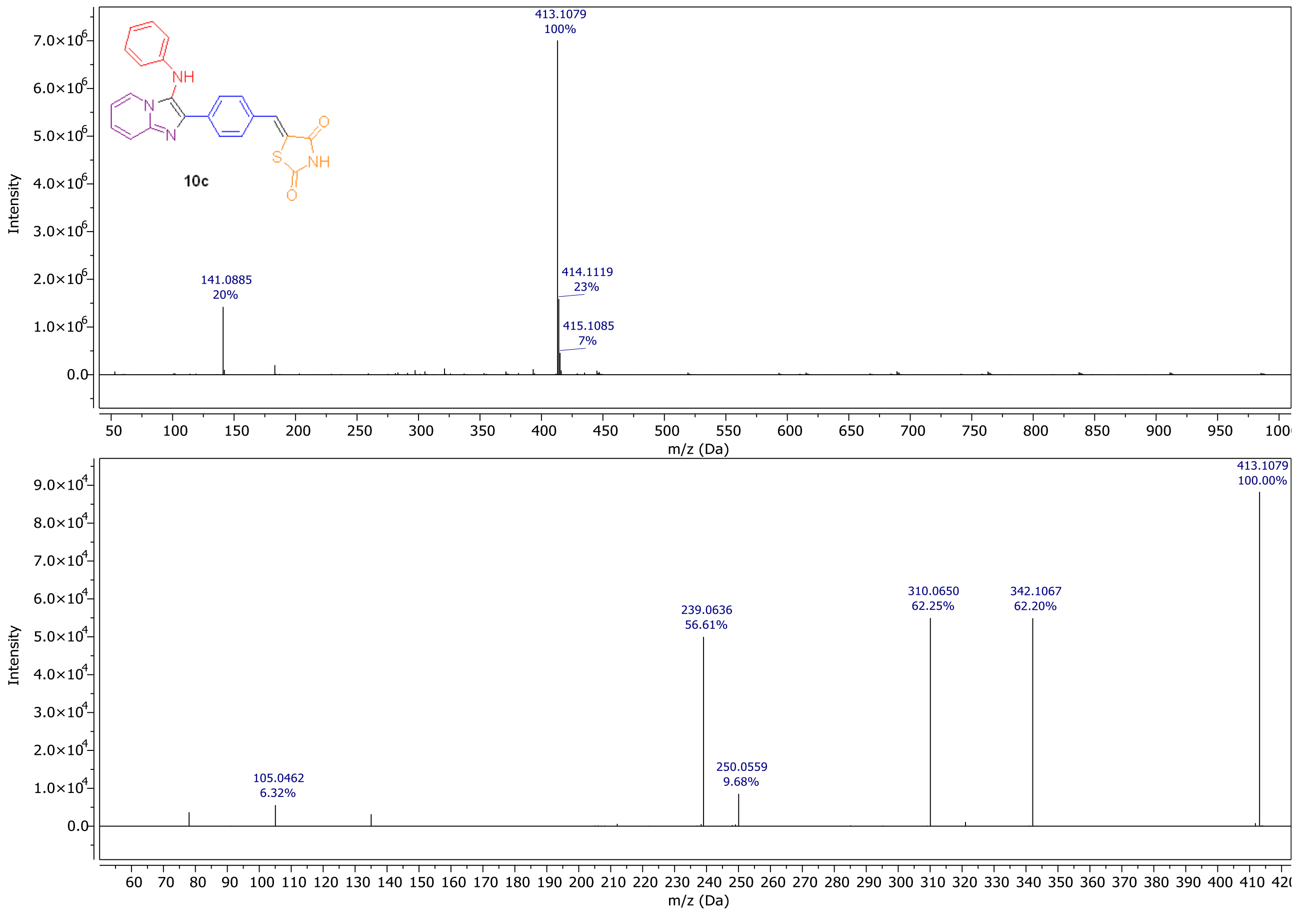

Figure S245. HRMS (ESI-QTOF) of compound **10c** and HRMS/MS for $[M+H]^+$.

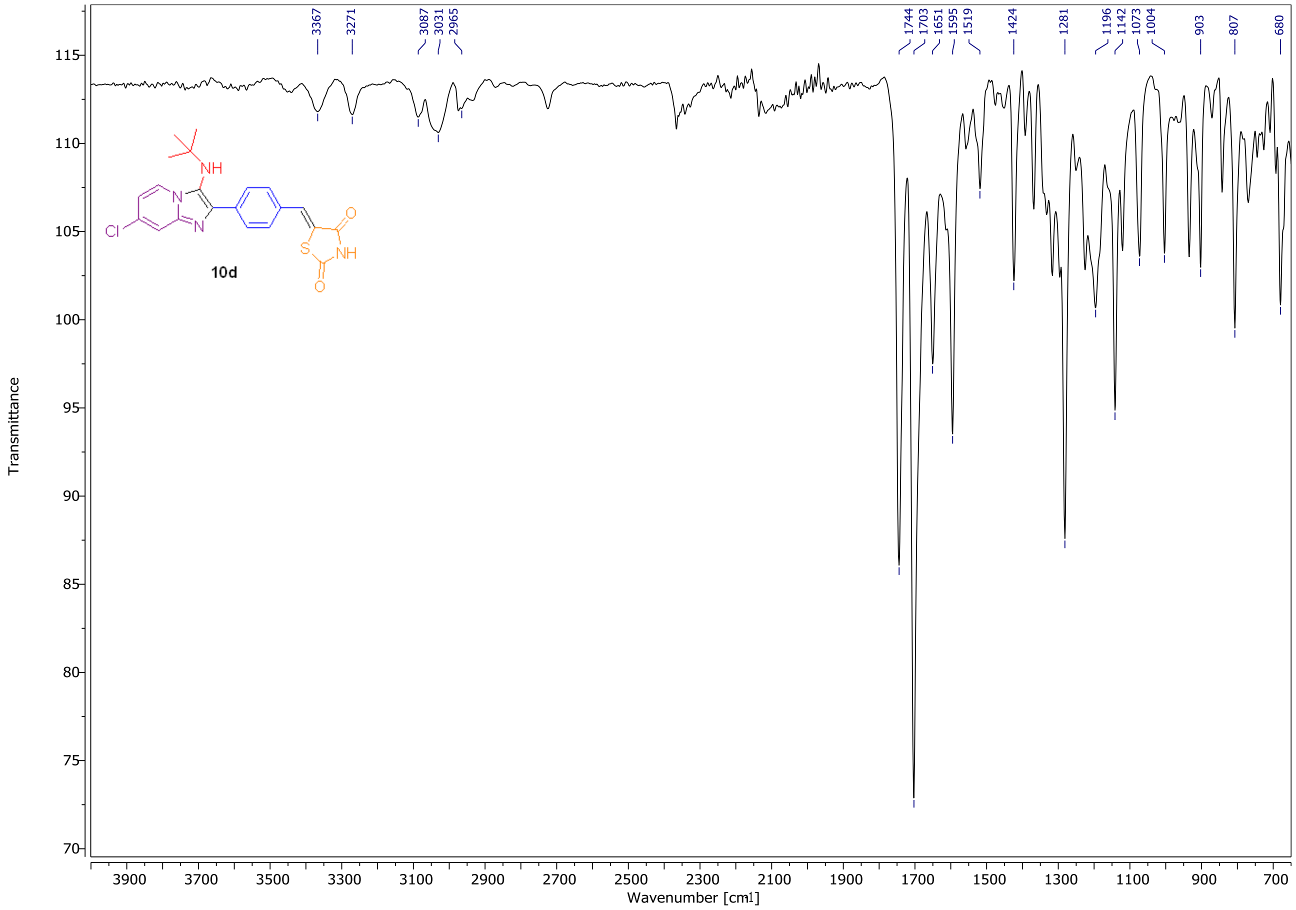

Figure S246. FTIR (ATR) of compound **10d**.

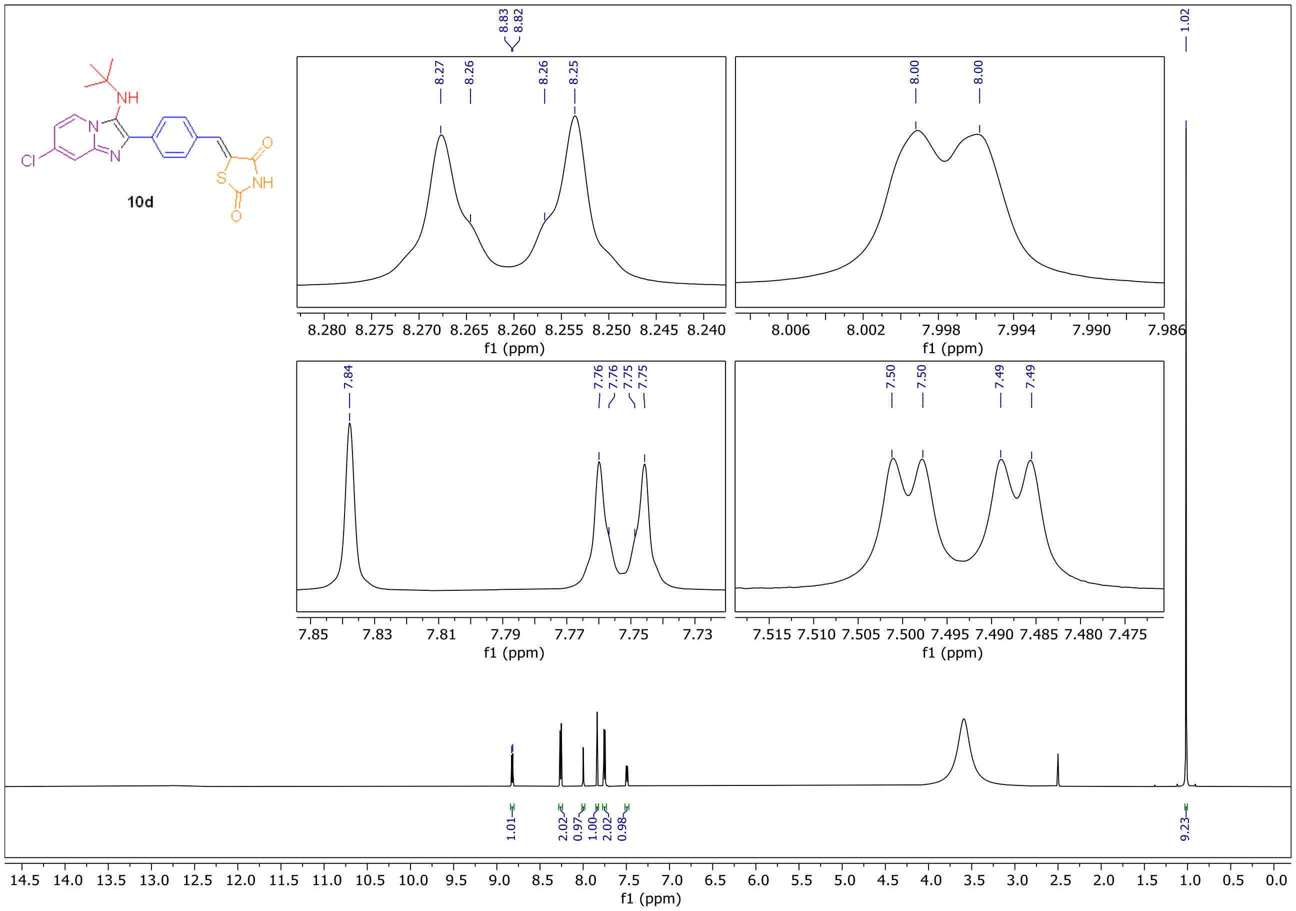

Figure S247. ^{1}H NMR spectrum (600 MHz, DMSO-*d*6) of compound **10d**.

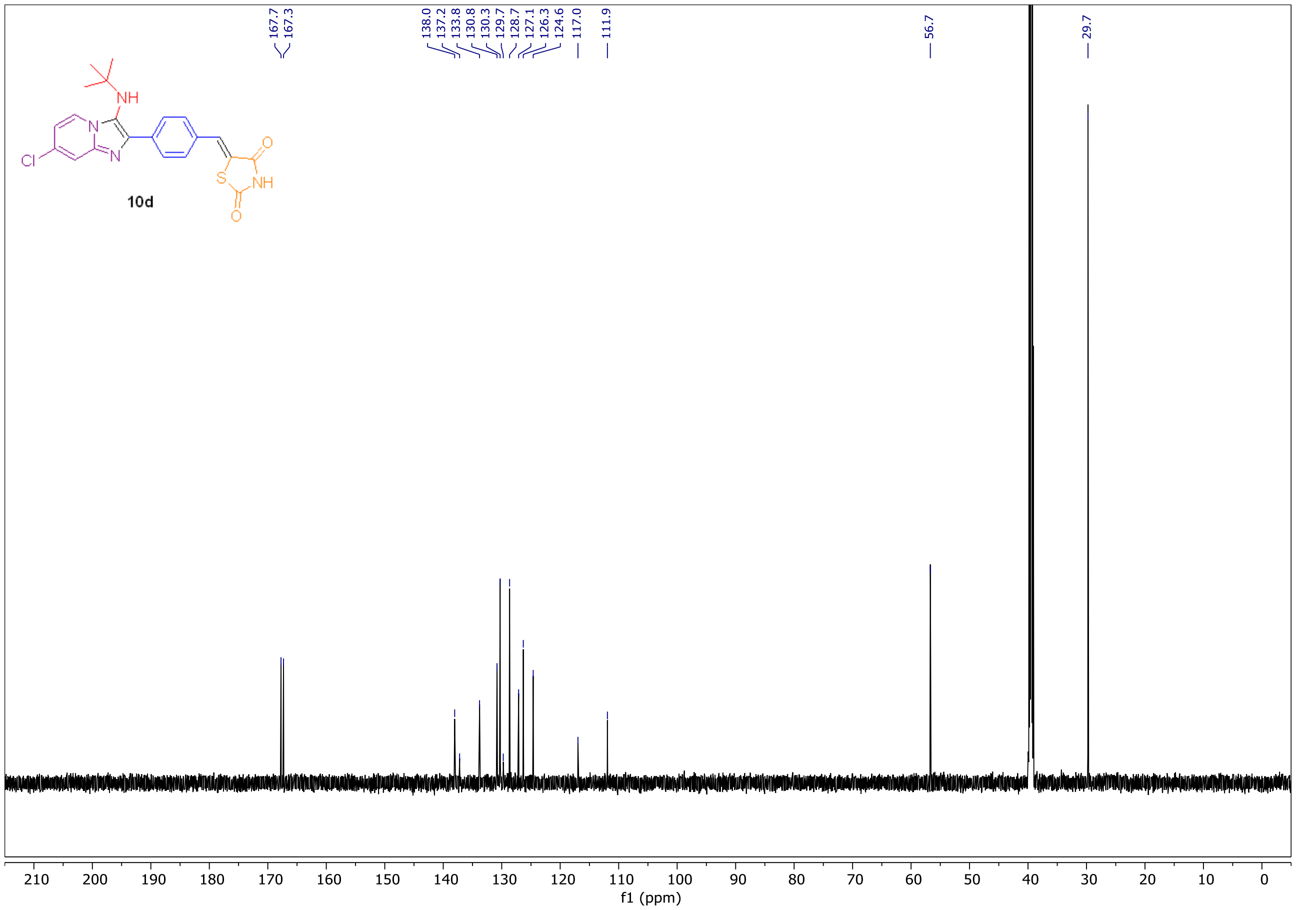

Figure S248. ^{13}C NMR spectrum (151 MHz, DMSO-d_6) of compound **10d**.

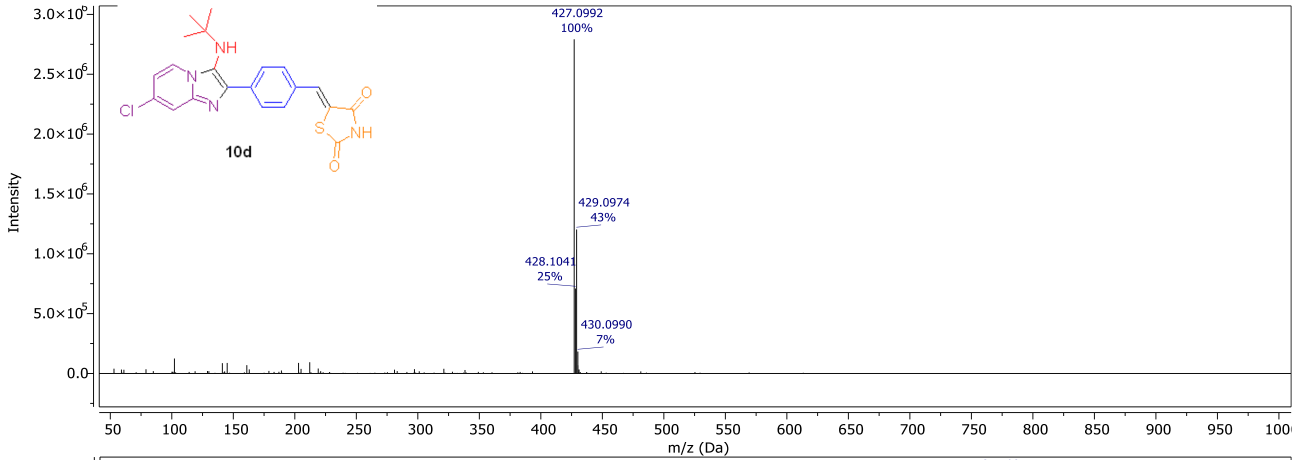

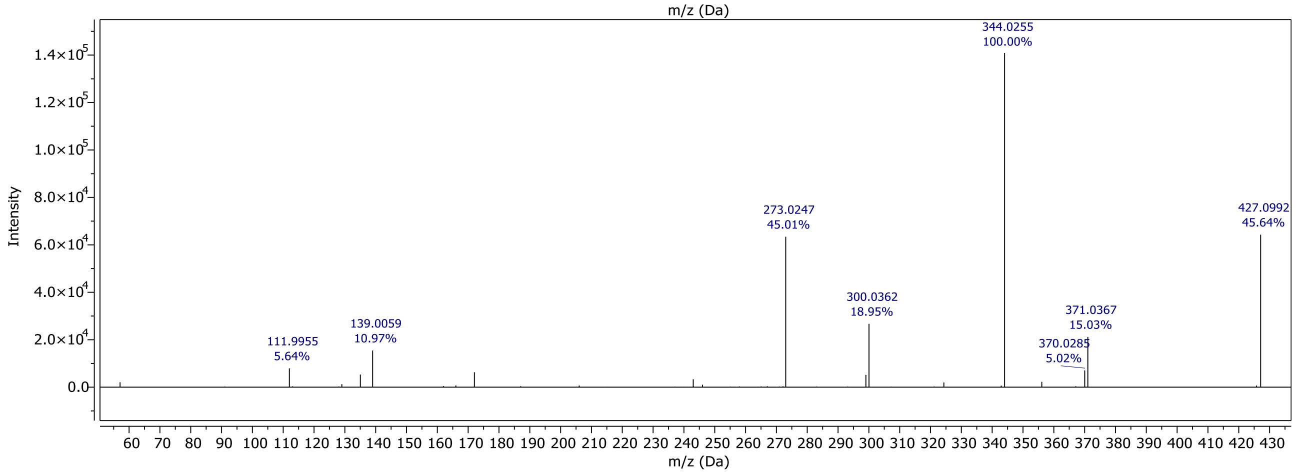

Figure S249. HRMS (ESI-QTOF) of compound **10d** and HRMS/MS for $[M+H]^+$.

4. Crystal structure determination of compounds 3n and 4n

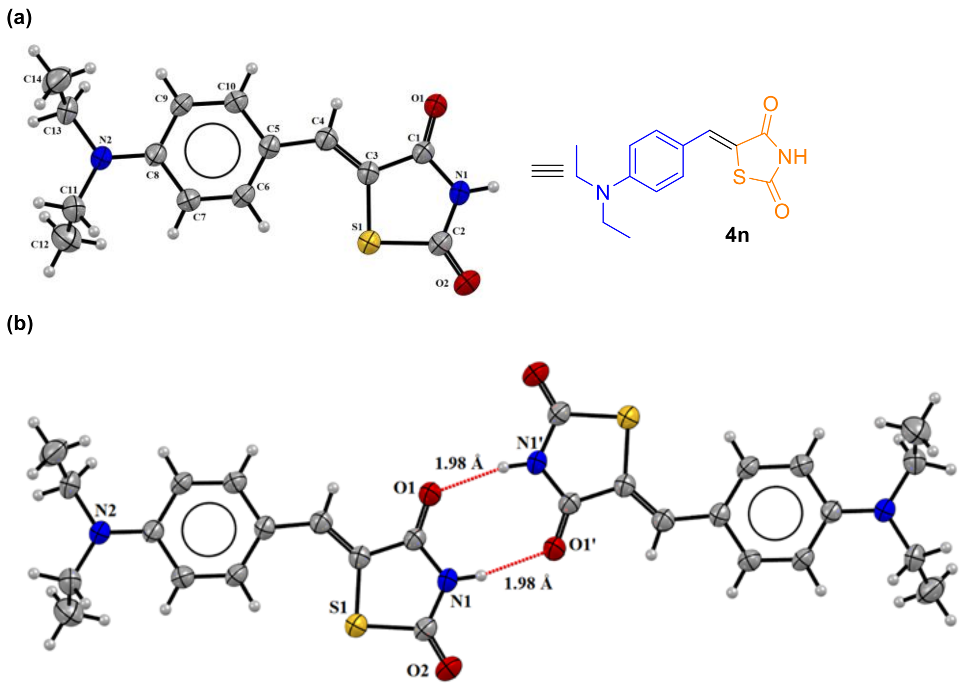

Figure S250. a) Molecular structure of **4n** with crystallographic labeling (50% probability displacement). b) Perspective views of intermolecular hydrogen bonds (dotted lines) of **4n**. (‘) and symmetry operation: −*x, y+1, z+1*.

Table S2. X-ray diffraction data collection and refinement parameters for **3n** and **4n**.

	**3n**	**4n**
Chemical formula	$C_{14}H_{16}N_2OS_2$	$C_{14}H_{16}N_2O_2S$
M (g mol^{-1})	292.41	276.35
Crystal system	Monoclinic	Triclinic
Space group	$P2_1/c$	*P*-1
Unit cell		
a (Å)	10.949(12)	7.787(3)
b (Å)	9.603(11)	9.398(4)
c (Å)	14.589(15)	10.284(4)
α (°)	90	108.485(6)
β (°)	101.18(2)	97.918(7)
γ (°)	90	102.172(7)
V (Å^3)	1505(3)	680.5(5)
Z	4	2
Dc /g cm^{-3}	1.291	1.349
Index ranges	$-13 \le h \le 12$<br>$-11 \le k \le 11$	$-9 \le h \le 9$<br>$-11 \le k \le 11$

	–16 ≤ l ≤ 17	–12 ≤ l ≤ 12
Absorption coefficient /mm^{-1}	0.347	0.237
Absorption correction	multi-scan	multi-scan
Max/min transmission	1.00 / 0.75	0.96 / 0.76
Measured reflections	13977	15980
Independent reflections / R_{int}	2652 / 0.109	2519 / 0.058
Refined parameters	179	175
R_1 (F) / wR_2 (F^2) (I > 2s(I))	0.049 / 0.108	0.035 / 0.082
GooF	0.980	1.057
Largest diff. peak and hole (eÅ^{-3})	0.264 and -0.290	0.194 and -0.183
Deposit number CCDC	2419877	2419878

Table S3. Selected bond lengths (Å) and bond angles (°) for **3n** and **4n**.

**Bond lengths (Å)**			
**3n**		**4n**	
S(1)-C(2)	1.746(4)	S(1)-C(3)	1.753(2)
S(1)-C(3)	1.750(4)	S(1)-C(2)	1.782(2)
S(2)-C(2)	1.629(4)	O(2)-C(2)	1.202(2)
O(1)-C(1)	1.229(4)	O(1)-C(1)	1.221(2)
N(2)-C(8)	1.365(4)	N(2)-C(8)	1.364(2)
N(2)-C(11)	1.458(4)	N(2)-C(11)	1.459(2)
N(2)-C(13)	1.468(5)	N(2)-C(13)	1.468(2)
N(1)-C(2)	1.357(4)	N(1)-C(1)	1.369(2)
N(1)-C(1)	1.390(4)	N(1)-C(2)	1.371(2)
C(12)-C(11)	1.518(5)	C(12)-C(11)	1.500(3)
**Bond angles (°)**			
**3n**		**4n**	
C(2)-S(1)-C(3)	93.04(17)	C(3)-S(1)-C(2)	91.90(8)
C(8)-N(2)-C(11)	122.3(3)	C(8)-N(2)-C(11)	121.99(15)
C(8)-N(2)-C(13)	121.9(3)	C(8)-N(2)-C(13)	121.42(15)
N(1)-C(2)-S(2)	126.4(3)	C(11)-N(2)-C(13)	116.55(15)
N(1)-C(2)-S(1)	109.0(3)	C(1)-N(1)-C(2)	117.79(15)
S(2)-C(2)-S(1)	124.6(2)	C(1)-C(3)-S(1)	109.85(12)
O(1)-C(1)-N(1)	122.4(3)	O(2)-C(2)-N(1)	125.75(17)
O(1)-C(1)-C(3)	127.8(3)	O(2)-C(2)-S(1)	124.89(15)
N(1)-C(1)-C(3)	109.7(3)	N(1)-C(2)-S(1)	109.36(12)
N(2)-C(13)-C(14)	112.7(3)	O(1)-C(1)-N(1)	123.54(16)

5. Photophysical data

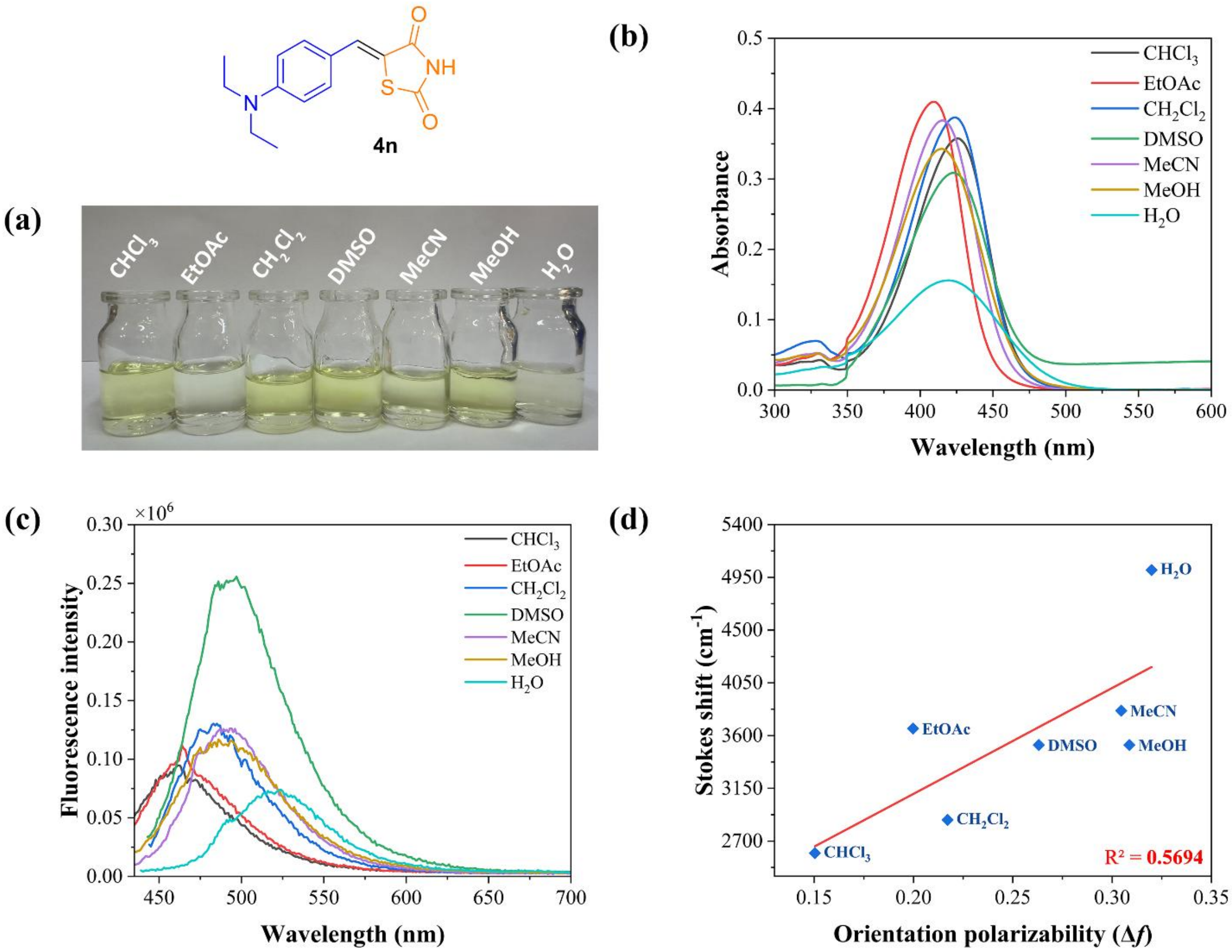

Figure S251. a) Visual impressions of the solvatochromic study at various solvents (10^{-5} M) after excitation with a natural light. b) UV–vis absorption spectra of **4n** in different solvents (10^{-5} M) at room temperature. c) Normalized emission spectra of **4n** in different solvents (10^{-5} M) at room temperature. d) Lippert–Mataga plot showing Stokes shift as a function of solvent orientation polarizability (Δf) for compound **4n**.

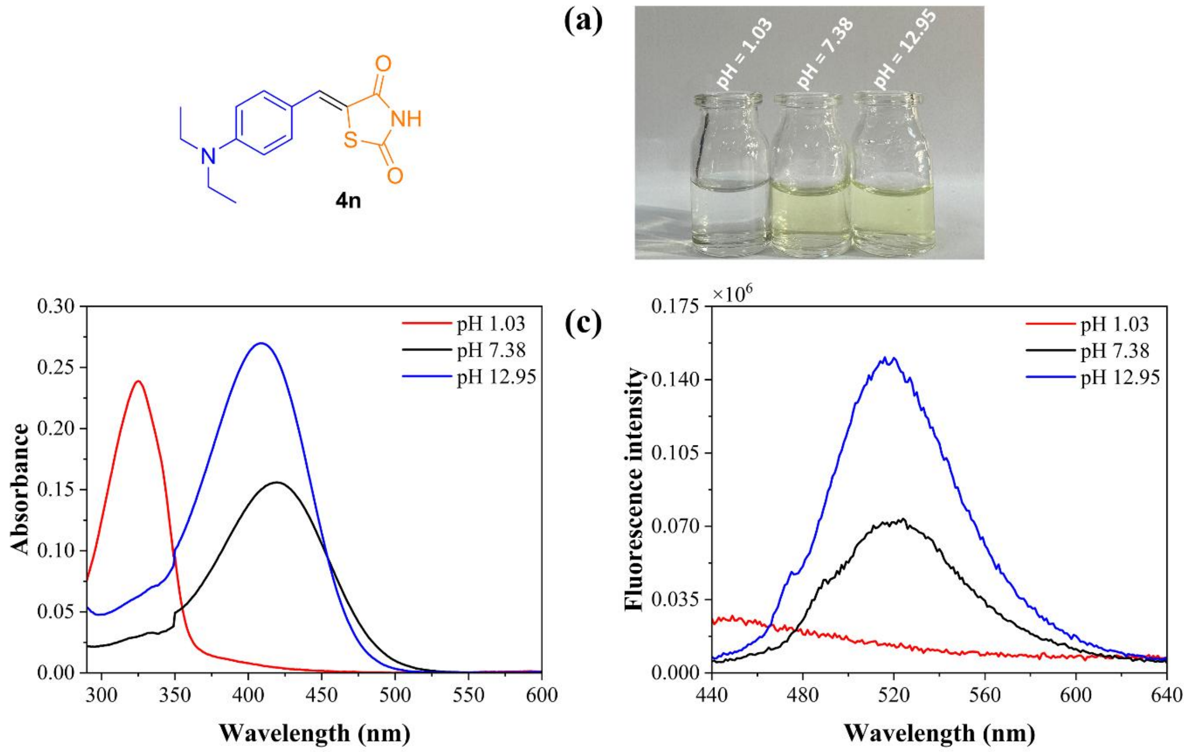

Figure S252. Photophysical study in aqueous solutions under different pH values for compound **4n** (10^{-5} M) at room temperature. a) Visual impressions of the aqueous solution at various pH after excitation with a natural light. b) UV–vis absorption spectra. c) Normalized emission spectra.

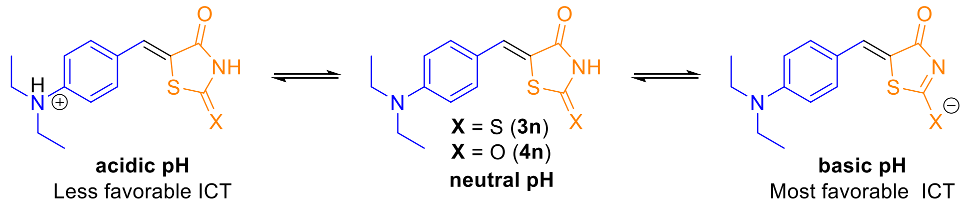

Scheme S2. Possible structures at different pH values for compounds **3n** and **4n**.

6. Benchmarking study

Compounds **3n** and **4n** were studied under nine different functional-basis set combinations (Tables S4 and S5) and the B97-D/def2-TZVPP yielded the lowest mean absolute error (MAE = 2.67 ppm) and was chosen as the method of choice. Atom numbers in the tables below correspond to the numbers prescribed by UCSF Chimera upon building the structure (Figures S253 and S254). Peaks not immediately assigned and quaternary carbon atom shifts were left out of the benchmarking study.

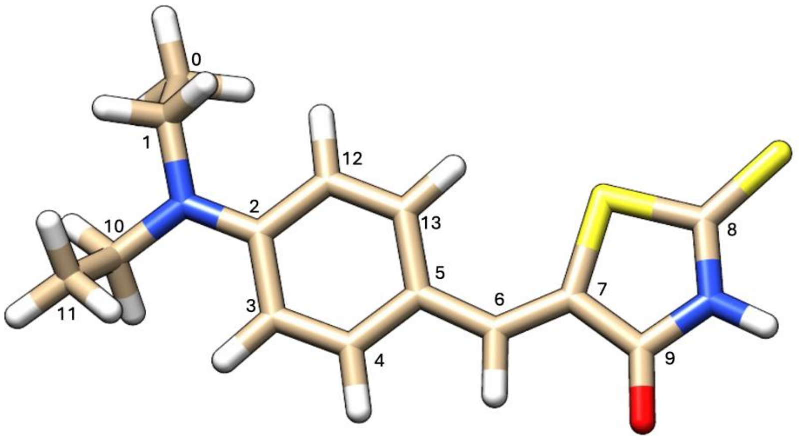

Figure S253. Compound **3n** with labeled atom numbers.

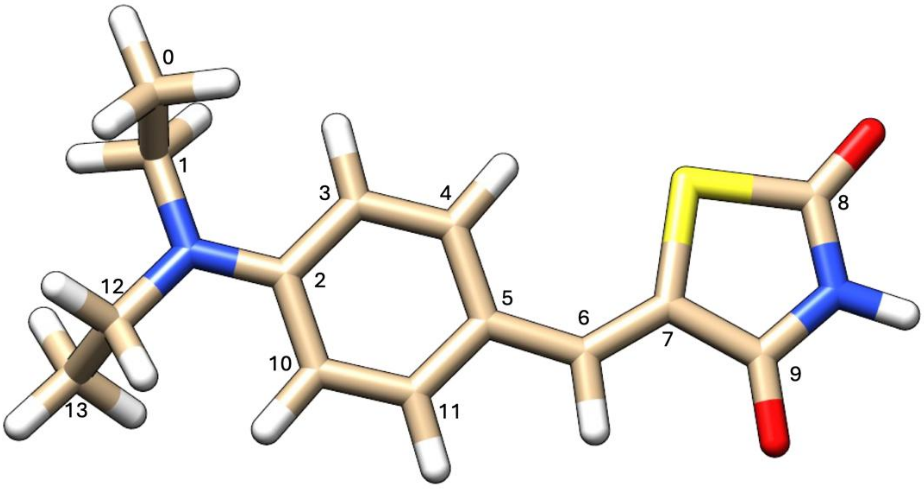

Figure S254. Compound **4n** with labeled atom numbers.

Table S4. Table of benchmarking study of compound **3n** using multiple combinations of density functionals and basis sets.

	Atom	Expt.	B97-D/def2-TZVPP	Error	TPSSh/pcSseg-2	Error	TPSSh/pcSseg-3	Error
CH3	0	12.8	13.23	0.43	14.11	1.31	14.46	1.66
CH2	1	44.7	50.31	5.61	51.35	6.65	51.95	7.25
C	2							
CH	3							
CH	4							
C	5							
CH	6	133	133.82	0.82	142.08	9.08	143.03	10.03
C	7							
C=S	8	195.4	192.72	2.68	204.74	9.34	205.55	10.15
C=O	9	169.9	166.72	3.18	175.57	5.67	176.85	6.95
CH2	10	44.7	50.21	5.51	51.25	6.55	51.91	7.21
CH3	11	12.8	13.24	0.44	14.09	1.29	14.42	1.62
CH	12							
CH	13							
			**MAE**	2.66714286		5.69857143		6.41

	Atom	Expt.	wB97-X D3/def2-TZVPP	Error	wB97-X D3/pcSseg-3	Error	RI-DSD-PBEP86/def2-TZVPP	Error
CH3	0	12.8	12.51	0.29	12.99	0.19	13.22	0.42
CH2	1	44.7	47.73	3.03	49.33	4.63	45.78	1.08
C	2							
CH	3							
CH	4							
C	5							
CH	6	133	151.03	18.03	156.09	23.09	155.24	22.24
C	7							
C=S	8	195.4	219.48	24.08	226.67	31.27	230.47	35.07
C=O	9	169.9	178.16	8.26	183.92	14.02	180.71	10.81
CH2	10	44.7	47.67	2.97	49.21	4.51	45.84	1.14
CH3	11	12.8	12.64	0.16	13.09	0.29	13.3	0.5
CH	12							
CH	13							
			**MAE**	8.11714286		11.1428571		10.18

	Atom	Expt.	RI-DSD-PBEP86 D3BJ/def2-TZVPP	Error	RI-B2PLYP/def2-TZVPP	Error	RI-B2PLYP D3/def2-TZVPP	Error
CH3	0	12.8	13.07	0.27	13.19	0.39	13.06	0.26
CH2	1	44.7	48.9	4.2	47.33	2.63	49.75	5.05
C	2							
CH	3							
CH	4							
C	5							
CH	6	133	140.12	7.12	151.97	18.97	141.72	8.72
C	7							
C=S	8	195.4	203.07	7.67	225.61	30.21	205.25	9.85
C=O	9	169.9	172.5	2.6	179.67	9.77	173.95	4.05
CH2	10	44.7	48.66	3.96	47.35	2.65	49.63	4.93
CH3	11	12.8	13.09	0.29	13.26	0.46	13.02	0.22
CH	12							
CH	13							
			**MAE**	3.73		9.29714286		4.72571429

Table S5. Table of Benchmarking study of compound **4n** using multiple combination of density functionals and basis sets.

	Atom	Expt.	B97-D/def2-TZVPP	Error	TPSSh/pcSseg-2	Error	TPSSh/pcSseg-3	Error
CH3	0	12.5	13.05	0.55	13.95	1.45	14.31	1.81
CH2	1	45	49.84	4.84	50.88	5.88	51.5	6.5
C	2							
CH	3							
CH	4							
C	5							
CH	6	132.9	133.73	0.83	141.62	8.72	142.67	9.77
C	7							
C(=O)-S	8	168.6	170.35	1.75	176.6	8	179.82	11.22
C=O	9	167.9	165.25	2.65	174.1	6.2	175.29	7.39
CH	10							
CH	11							
CH2	12	45	49.75	4.75	50.83	5.83	51.51	6.51
CH3	13	12.5	12.93	0.43	13.82	1.32	14.14	1.64
			**MAE**	2.25714286		5.34285714		6.40571429

	Atom	Expt.	wB97-X D3/def2-TZVPP	Error	wB97-X D3/pcSseg-3	Error	RI-DSD-PBEP86/def2-TZVPP	Error
CH3	0	12.5	12.57	0.65	13.03	0.19	13.32	0.1
CH2	1	45	47.4	1.62	48.97	3.19	45.43	0.35
C	2							
CH	3							
CH	4							
C	5							
CH	6	132.9	149.24	6	154.31	0.93	153.46	1.78
C	7							
C(=O)-S	8	168.6	180.58	49.89	186.56	43.91	184.55	45.92
C=O	9	167.9	176.93	3.78	182.76	2.05	179.57	1.14
CH	10							
CH	11							
CH2	12	45	47.29	47.29	48.9	48.9	45.42	45.42
CH3	13	12.5	12.48	12.48	12.96	12.96	13.1	13.1
			**MAE**	17.3871429		16.0185714		15.4014286

	Atom	Expt.	RI-DSD-PBEP86 D3BJ/def2-TZVPP	Error	RI-B2PLYP/def2-TZVPP	Error	RI-B2PLYP D3/def2-TZVPP	Error
CH3	0	12.5	13	13	13.25	13.25	12.97	12.97
CH2	1	45	48.48	47.48	46.96	45.96	49.13	48.13
C	2							
CH	3							
CH	4							
C	5							
CH	6	132.9	138.62	132.62	150.68	144.68	140.72	134.72
C	7							
C(=O)-S	8	168.6	173.56	165.56	183.33	175.33	176.2	168.2
C=O	9	167.9	170.95	161.95	178.42	169.42	172.33	163.33
CH	10							
CH	11							
CH2	12	45	48.48	36.48	46.93	34.93	49.44	37.44
CH3	13	12.5	12.91	0.09	13.05	0.05	12.84	0.16
			**MAE**	79.5971429		83.3742857		80.7071429
